# Nickel-catalyzed regio- and enantio-selective Markovnikov hydromonofluoroalkylation of 1,3-dienes†

**DOI:** 10.1039/d2sc03958c

**Published:** 2022-10-11

**Authors:** Ling Liao, Ying Zhang, Zhong-Wei Wu, Zhong-Tian Ye, Xue-Xin Zhang, Guangying Chen, Jin-Sheng Yu

**Affiliations:** Shanghai Engineering Research Center of Molecular Therapeutics and New Drug Development, Shanghai Key Laboratory of Green Chemistry and Chemical Processes, East China Normal University Shanghai 200062 China jsyu@chem.ecnu.edu.cn; Key Laboratory of Tropical Medicinal Resource Chemistry of Ministry of Education, Hainan Normal University Haikou 571158 China

## Abstract

A highly enantio- and regio-selective Markovnikov hydromonofluoro(methyl)alkylation of 1,3-dienes was developed using redox-neutral nickel catalysis. It provided a facile strategy to construct diverse monofluoromethyl- or monofluoroalkyl-containing chiral allylic molecules. Notably, this represents the first catalytic asymmetric Markovnikov hydrofluoroalkylation of olefins. The practicability of this methodology is further highlighted by its broad substrate scope, mild base-free conditions, excellent enantio- and regio-selectivity, and diversified product elaborations to access useful fluorinated building blocks.

## Abstract 2

We report a highly enantio- and regio-selective Markovnikov hydromonofluoro(methyl)alkylation of 1,3-dienes using chiral Ni catalysis, allowing access to chiral allylic compounds bearing a CH_2_F, CD_2_F or monofluoroalkyl group at the stereocenter.

## Graphical abstract

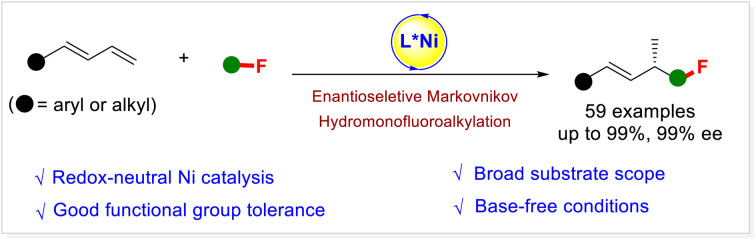

## Introduction

**Scheme 1:**
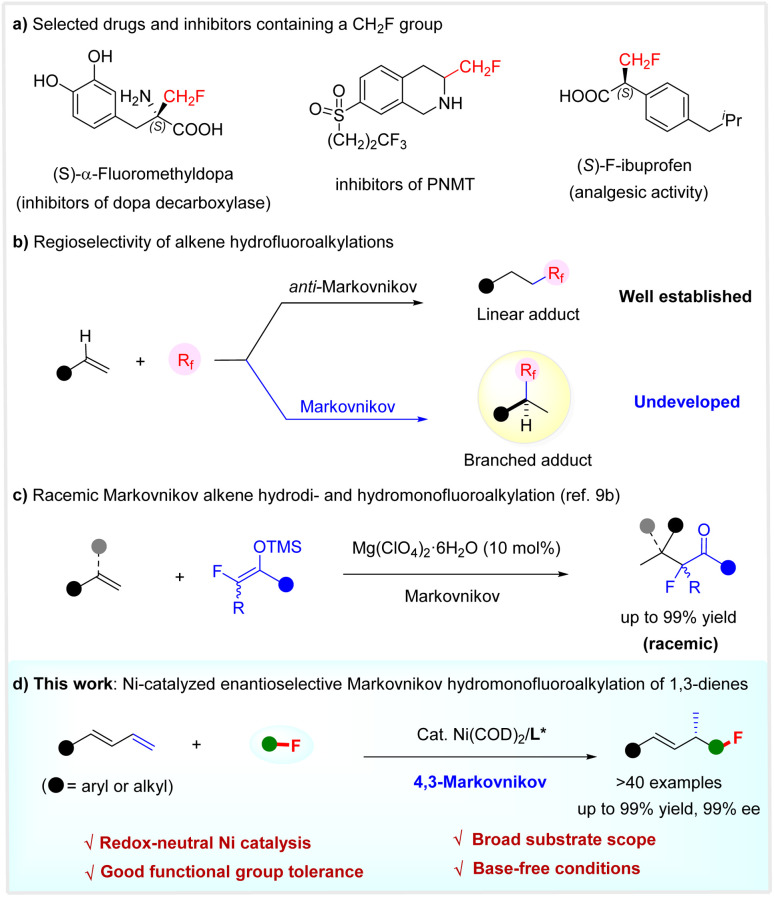
Regioselective hydrofluoroalkylation of alkenes.

The selective introduction of a fluorine or fluoroalkyl moiety into molecules often results in improved physical, chemical, and biological properties.^1^ In particular, the installation of a monofluoromethyl (CH_2_F) group as a bioisostere of various functional groups, such as methyl and hydroxymethyl, has been established as a robust and routine tactic in pharmaceutical chemistry and agrochemistry to tune the properties of bioactive compounds, including bioavailability and metabolic stability.^2^ Typical drugs or inhibitors featuring a CH_2_F unit are shown in Scheme 1a.^3^ However, despite the significant progress made in selective fluoroalkylation,^4^ the efficient incorporation of a CH_2_F group in a highly enantioselective manner remains challenging and very much in demand.^5^

While the hydrofluoroalkylation of alkenes is a powerful strategy to introduce a fluoroalkyl group selectively,^6^ the catalytic enantioselective incorporation of a monofluoroalkyl group is unexplored. Notably, most known alkene hydrofluoroalkylations are based on radical processes, affording linear adducts with anti-Markovnikov regioselectivity.^6,7^ It is considered both interesting and urgent to develop the Markovnikov hydrofluoroalkylation of olefins. This not only offers the potential to develop catalytic enantioselective versions, but would afford branched adducts with a chemical space shape distinct from linear products, which are interesting targets for drug discovery because of the intimate relationship between the shape and their properties of organic molecules (Scheme 1b).^8^ Following our interest in selective fluoroalkylation,^9^ we recently developed the first Markovnikov hydrodi- and hydromonofluoroalkylation of simple alkenes using fluorinated enol silyl ethers, *via* an acid-catalyzed carbocationic process (Scheme 1c).^9*b*^ Herein, we disclose a highly regio- and enantio-selective Markovnikov hydromonofluoro(methyl)alkylation reaction of 1,3-dienes by redox-neutral Ni catalysis (Scheme 1d).

Transition-metal-catalyzed regio- and enantio-selective hydrofunctionalization of 1,3-dienes 1 offers an efficient and atom-economical method to access chiral functionalized allylic compounds from readily available starting materials.^10^ Over the past few years, various highly enantioselective protocols have been used: hydroamination,^11^ hydroalkylation,^12^ hydroarylation,^13^ and hydrosulfonylation,^14^ among others.^15^ Despite the advances made, these reactions mainly rely on using chiral precious metal Pd and Rh catalysts. Since the landmark work of the Zhou group in 2018,^12*c*^ the use of earth-abundant and low-cost chiral Ni catalysts for developing the asymmetric hydrofunctionalization of acyclic 1,3-dienes has gained increasing attention.^12*c*,*e*,*f*,13*b*,*c*^ Despite ongoing achievements, the catalytic enantio- and regio-selective Markovnikov hydromonofluoromethylation of 1,3-dienes to construct functionalized chiral allylic compounds with a CH_2_F at the stereocenter is unexplored.

Inspired by these elegant advances, we speculated that the implementation of catalytic asymmetric 1,3-dienes hydromonofluoromethylation would provide a new direction for enantioselective monofluoromethylation and constitutes a new branch for the hydrofunctionalizations of 1,3-dienes. To reach this goal, the quest for a suitable monofluoromethyl reagent would be the key to success. Among various monofluoromethyl agents, fluorobis(phenyl-sulfonyl)methane (FBSM)^16*a*,*b*^2a proves to be a robust one in developing catalytic enantioselective monofluoromethylation reactions,^5,16^ since the landmark work of Shibata.^16*a*^ On this basis, we determined to use FBSM 2a as a latent monofluoromethyl agent to explore the asymmetric Markovnikov hydromonofluoromethylation of 1,3-dienes 1 under the action of chiral nickel catalysis.

## Results and discussion

### Optimization of the reaction conditions

We commenced this study with 1-phenylbuta-1,3-diene 1a and FBSM 2a as the model substrates, in the presence of Ni(COD)_2_ (10 mol%) and *N*,*N*-diisopropylethylamine (DIPEA) (20 mol%). As shown in Table 1, a series of axially chiral bisphosphine ligands were first investigated. The use of (*S*)-Tol-BINAP afforded the 4,3-Markovnikov adduct 3a in 98% NMR yield with 37% ee within 14 h (entry 1), whilst the bulky (*R*)-DTBM-Segphos L2 afforded product 3a in only 9% NMR yield and 27% ee after 48 h (entry 2).

**Table 1:** Selected conditions for optimization^a^

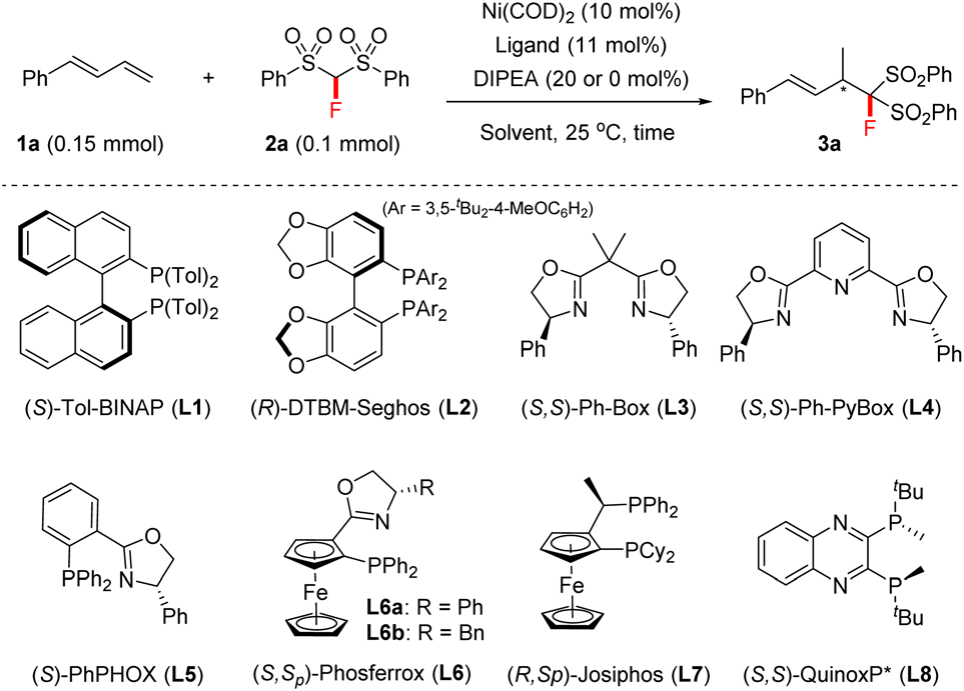						
Entry	Ligand	DIPEA (mol%)	Solvent	Time (h)	Yield^b^ (%)	ee^c^ (%)
1	L1	20	EtOH	14	98	37
2	L2	20	EtOH	48	9	27
3	L3	20	EtOH	24	4	22
4	L4	20	EtOH	24	nr^d^	—
5	L5	20	EtOH	14	79	22
6	L6a	20	EtOH	10	89	68
7	L6a	0	EtOH	10	90	67
8	L6b	0	EtOH	22	50	64
9	L7	0	EtOH	22	50	78
10	L8	0	EtOH	16	95	96
11	L8	0	Toluene	24	84	70
12	L8	0	THF	24	99	86
13	L8	0	CH_2_Cl_2_	24	Trace	—
14	L8	0	MeOH	24	Trace	—
15	L8	0	^i^PrOH	24	Trace	—
16^e^	L8	0	EtOH	72	86	96

a Reaction conditions: 1a (0.15 mmol), 2a (0.1 mmol), Ni(COD)_2_ (10 mol%), ligand (11 mol%), and DIPEA (20 or 0 mol%), run at 25 °C in the indicated solvent (1.0 mL), unless otherwise noted.

b Determined by ^1^H NMR analysis of the crude product using 1,3,5-trimethoxybenzene as the internal standard.

c Determined by chiral HPLC.

d No reaction.

e Run on a 0.25 mmol scale using Ni(COD)_2_ (5 mol%) and L8 (5.5 mol%).

Encouraged by these results, we then tested the performance of chiral bisoxazoline ligands and *P*,*N*-based PHOX (entries 3–6), and found that the use of (*S*,*Sp*)-Ph-Phosferrox L6a could improve the ee of product 3a to 68% (entry 6). Interestingly, base DIPEA proved to be unnecessary in the current reaction. A comparable result was obtained in the absence of DIPEA (entries 6 *vs.* 7). The focus of further optimization was on chiral ferrocene-based chiral ligands, but there was no improvement in the ee values (entry 8, see the ESI† for details). Subsequently, we turned our attention to exploring *P*-chiral phosphine ligands because they usually exhibit distinct chirality-inducing ability.^17^ To our delight, *P*-chiral (*S*,*S*)-QuinoxP*^18^L8, never before used in hydrofunctionalizations of 1,3-dienes, proved to be efficient; it afforded 3a in 95% NMR yield with 96% ee within 16 h (entry 10). An examination of the solvent effect revealed that EtOH was still the best solvent (entries 10 *vs.* 11–15), although the use of THF also afforded the desired product 3a in 99% NMR yield, but with a slightly lower ee (entry 12). Moreover, the use of a 5 mol% Ni catalyst afforded the product 3a in 86% isolated yield with 96% ee, albeit within 72 h (entry 16).

### Evaluation of substrate scope

With the optimized conditions in hand, we explored the generality of this Markovnikov hydromonofluoromethylation in EtOH under the catalysis of a 5 mol% or 10 mol% *P*-chiral (*S*,*S*)-QuinoxP* decorated Ni(COD)_2_ complex (Table 2).

**Table 2:** Scope of hydromonofluoromethylation of dienes with 2 a

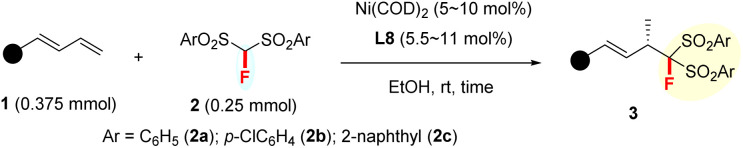						
Entry	1: substituent (●)	2	Time (d)	3	Yield (%)	ee (%)
1	1a: C_6_H_5_	2a	3	3a	86	96
2	1b: 4-MeC_6_H_4_	2a	4	3b	99	94
3	1c: 4-MeOC_6_H_4_	2a	3	3c	97	97
4^b^	1d: 3-MeOC_6_H_4_	2a	3	3d	86	98
5^b^	1e: 2-MeOC_6_H_4_	2a	3	3e	93	98
6	1f: 3,5-MeO_2_C_6_H_3_	2a	3	3f	98	99
7^b^	1g: 4-Me_2_NC_6_H_4_	2a	4	3g	48	93
8	1h: 4-CH_2_=CH(CH_2_)_2_C_6_H_4_	2a	3	3h	68	95
9	1i: 4-CF_3_C_6_H_4_	2a	4	3i	94	97
10	1j: 4-EtO_2_CC_6_H_4_	2a	3	3j	82	96
11	1k: 4-CNC_6_H_4_	2a	3	3k	90	98
12	1l: 4-COMeC_6_H_4_	2a	3	3l	90	98
13	1m: 4-CHOC_6_H_4_	2a	3	3m	71	90
14	1n: 4-ClC_6_H_4_	2a	4	3n	87	97
15	1o: 4-FC_6_H_4_	2a	4	3o	74	99
16^c^	1p: 3-FC_6_H_4_	2a	4	3p	92	96
17^c^	1q: 2-FC_6_H_4_	2a	4	3q	99	94
18	1r: 3,5-(CF_3_)_2_C_6_H_3_	2a	3	3r	49	95
19	1s: 2-naphthyl	2a	3	3s	97	99
20^c^	1t: 1-naphthyl	2a	3	3t	98	91
21^b^	1u: 2-furyl	2a	3	3u	85	93
22^b^	1v: 2-thienyl	2a	3	3v	98	98
23	1w: (*E*)-Ph-CH=CH	2a	5	3w	99	97
24^d^	1x: *n*-C_5_H_11_	2a	3	3x	67	93
25^d^	1y: PhCH_2_CH_2_	2a	5	3y	81	90
26^d^	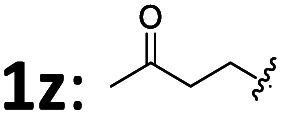	2a	4	3z	69	94
27^b^	1a: C_6_H_5_	2b	5	3aa	94	97
28^b^	1a: C_6_H_5_	2c	5	3ab	99	95
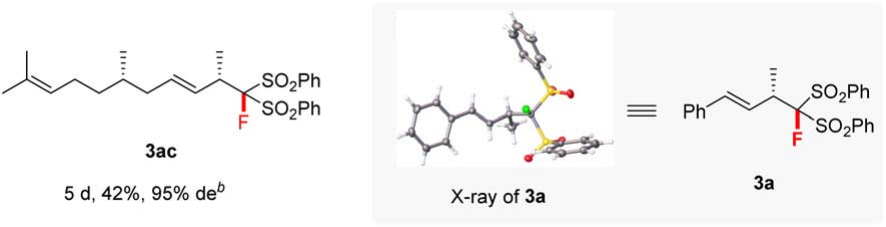						

a Conditions: 1 (0.375 mmol), 2 (0.25 mmol), and EtOH (2.5 mL), at rt, unless otherwise noted; yields of the isolated products are reported; the ee values were determined by chiral HPLC analysis. For 3a, 3c, 3o, and 3s: Ni(COD)_2_ (5 mol%) and L8 (5.5 mol%) were used; for the others: Ni(COD)_2_ (10 mol%) and L8 (11 mol%) were used.

b At 50 °C.

c At 60 °C.

d At 70 °C.

Various aromatic 1,3-dienes with different electron-donating and -withdrawing groups on the aryl ring were viable substrates, affording the corresponding 4,3-Markovnikov adducts 3b–3r in 48–99% yields with 90–99% ee (entries 2–18). The increase in reaction temperature was necessary to ensure full conversion in the cases of *ortho*- or *meta*-substituted aromatic 1,3-dienes. Of note is that the current reaction tolerated various functional groups on the aryl ring of 1,3-dienes: amine (3g), non-conjugated alkene (3h), ester (3j), nitrile (3k), ketone (3l), and aldehyde (3m). Naphthyl-, 2-furyl-, and 2-thienyl-substituted 1,3-dienes all worked smoothly with 2a to afford 3s–3v in excellent yields and ee values (entries 19–22). A conjugated triene was also tolerated; it afforded product 3w in 99% yield and with 97% ee (entry 23). Remarkably, the aliphatic 1,3-dienes, which are generally very challenging in terms of controlling both regio- and enantio-selectivity due to the small steric hindrance of the alkyl group,^12*c*^ proved to be compatible in our reaction system. They afforded the adducts 3x–3z with up to 81% yields and 94% ee at slightly elevated temperatures (entries 24–26). Notably, the ketone functionalities attached in aliphatic 1,3-diene were also compatible well (3z). The differently substituted FBSM 2b and 2c also reacted efficiently with 1-phenylbuta-1,3-diene 1a at 50 °C to afford the targets 3aa (94% yield and 97% ee) and 3ab (99% yield and 95% ee). Furthermore, (*S*)-citronellal-derived alkyl 1,3-diene also reacted smoothly to afford adduct 3ac in moderate yield and with 95% de. X-ray diffraction (XRD) analysis revealed that the absolute configuration of 3a was (*S*). Subsequently, (*S*) was assigned to all other products 3 by analogy.

**Scheme 2:**
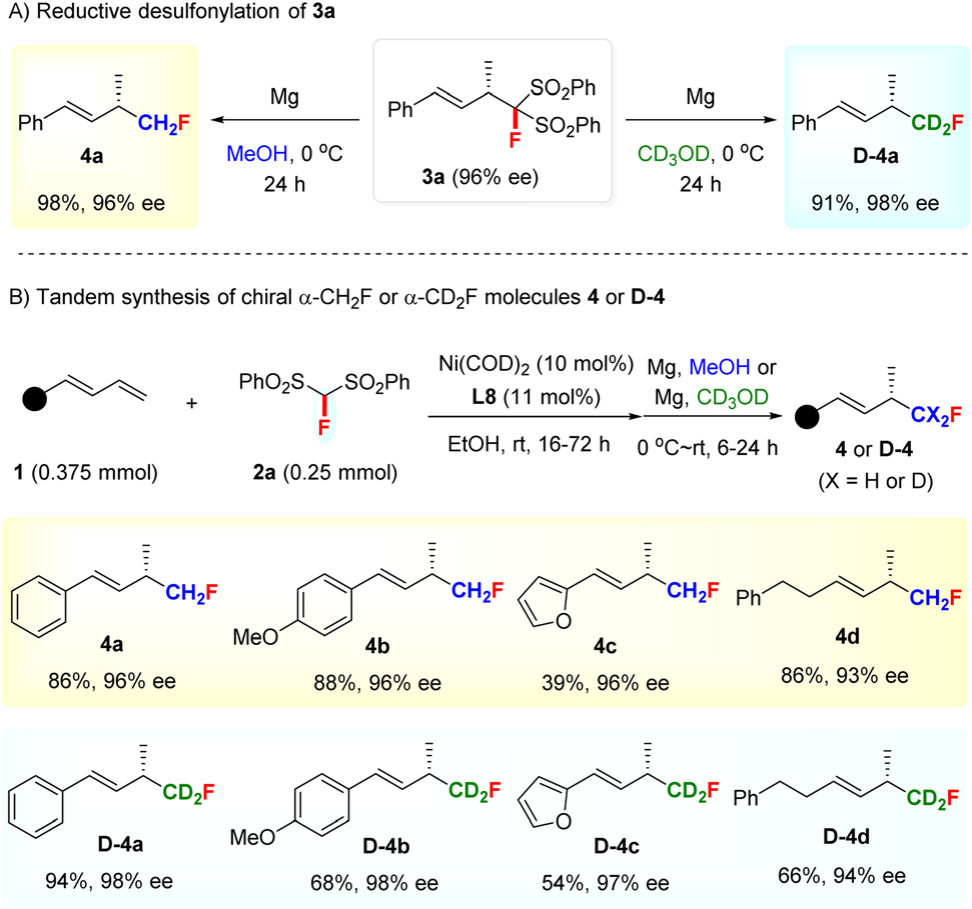
Synthetic applications of hydromonofluoromethylation.

Unsurprisingly, the FBSM adduct 3a could efficiently undergo a reductive desulfonylation to access chiral α-monofluoromethyl (CH_2_F) allylic compound 4a with 96% ee under the action of Mg/MeOH^19^ (Scheme 2A). This result stimulated us to explore the assembly of deuterated monofluoromethyl (CD_2_F)-containing chiral allylic molecules, given that the incorporation of a deuterium atom in the bioactive molecules is emerging as a promising tactic to modulate the bioactivity or pharmacological properties in drug discovery programs since the first deuterated drug, Austedo, was approved by FDA in 2017.^20*a*^ However, while the development of efficient approaches for preparing deuterated compounds is of current interest, the selective introduction of a CD_2_F group into the stereogenic center is still a challenging task and remains unexplored.^21^ To our delight, chiral deuterated allylic product D-4a featuring a CD_2_F group at the stereocenter, difficult to access by other methods, could be obtained smoothly by using CD_3_OD as the solvent in the desulfonylation step.

Furthermore, a tandem Ni-catalyzed asymmetric hydromonofluoro bis(phenylsulfonyl)methylation/reductive desulfonylation sequence was developed for the direct access of α-CH_2_F and α-CD_2_F substituted chiral allylic compounds 4 and D-4 (Scheme 2B). Both aryl- and alkyl-substituted 1,3-dienes were suitable partners for this tandem sequence, as exemplified by the preparation of 4a–4d and D-4a–4d with excellent ee values. It is worth mentioning that the facile synthesis of chiral allylic compounds bearing a CD_2_F-substituted stereocenter justified the use of FBSM as the monofluoromethylation reagent and further highlighted the value of our method.

**Scheme 3:**
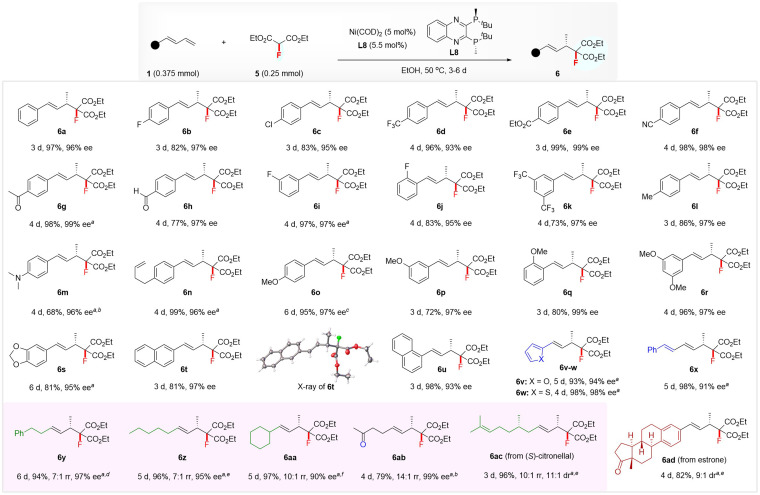
Scope of enantioselective hydromonofluoroalkylation of 1,3-dienes 1 with diethyl fluoromalonate 5. Conditions: 1 (0.375 mmol), 2 (0.25 mmol), Ni(COD)_2_ (5 mol%), and L8 (5.5 mol%) at 50 °C in EtOH (1.5 mL), unless otherwise noted. Yields of isolated products were reported and ee was determined by chiral HPLC analysis. ^*a*^ Using Ni(COD)_2_ (10 mol%) and L8 (11 mol%). ^*b*^ At 70 °C. ^*c*^ At 50–70 °C. ^*d*^ At 60 °C. ^*e*^ At 80 °C. ^*f*^ At 75 °C. The rr indicates the regioselectivity ratio of 4,3-Markovnikov isomer with another isomer, which was determined by ^1^H NMR analysis. The ee value of 6z and 6aa was determined by their derivatives; see the ESI† for details. The dr value of 6ac and 6ad was determined by ^19^F NMR analysis.

The excellent regio- and enantio-selectivity of the above hydromonofluoromethylation inspired us to explore the realization of enantioselective hydromonofluoroalkylation with diethyl fluoromalonate^4*p*,16*d*^5 because of its ability to simultaneously incorporate a fluorine atom and two convertible ester groups,^22^ which allows the construction of functionalized chiral monofluorinated molecules with high structural complexity. After optimization of the reaction conditions (see the ESI† for details), the combination of Ni(COD)_2_ and (*S*,*S*)-QuinoxP* L8 still proved to be an optimal catalytic system.^23^ As illustrated in Scheme 3, the substrate scope was examined by running the reaction in EtOH at 50 °C using 5 mol% of Ni(COD)_2_-ligated (*S*,*S*)-QuinoxP* as the catalyst. Both (hetero)aromatic and aliphatic 1,3-dienes were suitable substrates, affording the 4,3-Markovnikov adducts 6 with excellent regio- and enantio-selectivity. Regardless of the nature and position of the substituent on the phenyl ring of aryl 1,3-dienes, all reacted well with 5 to afford the products 6a–6u in 68–99% yields with 93–99% ee. The XRD analysis of 6t confirmed its absolute configuration to be (*S*), and that of other products was assigned by analogy. Various functional groups, such as ester (6e), nitrile (6f), ketone (6g), aldehyde (6h), amine (6m), and non-conjugated alkene (6n), on the aryl ring of aromatic 1,3-dienes were well-tolerated under this hydromonofluoroalkylation as well. Heteroaromatic 2-furyl- and 2-thienyl-substituted 1,3-dienes, as well as conjugated triene, also afforded the adducts 6v–6x in 93–98% yields and 91–98% ee. Moreover, linear and branched alkyl-substituted 1,3-dienes were tolerated, affording 4,3-Markovnikov adducts 6y–6ab in 79–97% yields and 90–97% ee, with high to excellent regioselectivity, albeit with the generation of a small amount of 4,1-addition isomer in these cases.^13*c*^ The use of a 10 mol% Ni catalyst was required to ensure excellent yields in the case of heteroaryl and alkyl 1,3-dienes. The protocol was also applied to the late-stage hydromonofluoroalkylation of (*S*)-citronellal and estrone derivatives, affording 6ac in 96% yield with 11 : 1 dr and 6ad in 82% yield with 9 : 1 dr.

### Synthetic utility

**Scheme 4:**
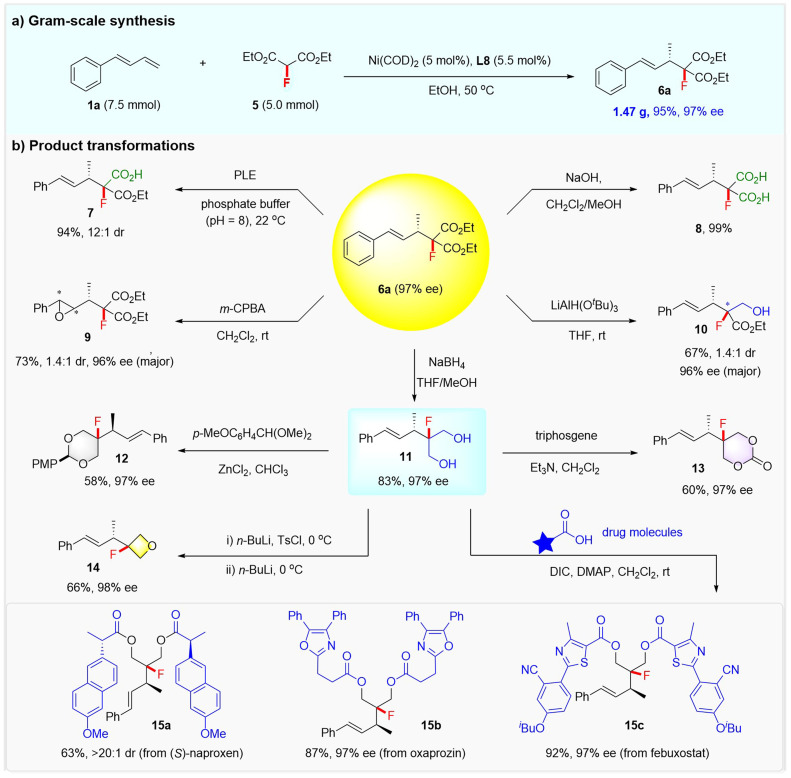
Synthetic utility.

To further highlight the practicality of the reaction, a gram-scale synthesis of product 6a and its synthetic elaborations toward structurally diversified fluorine-containing molecules was conducted. As shown in Scheme 4, starting from 1a (7.5 mmol) and 5 (5 mmol), 1.47 g of 6a could be readily generated in 95% yield and with 97% ee under the standard conditions. The two ester groups of 6a could be selectively hydrolyzed to fluorinated carboxylic acid or dicarboxylic acid, as demonstrated by the synthesis of 7 (94% yield and 12 : 1 dr) *via* a porcine liver esterase (PLE) enabled hydrolytic desymmetrization, and 8 (99% yield) *via* NaOH-mediated hydrolysis. The treatment of 6a with *m*-CPBA led to the epoxidation of the alkenyl and afforded chiral fluorinated epoxide 9 in 73% yield, albeit with modest dr. Compound 6a could also be selectively reduced with LiAl(O^*t*^Bu)_3_ or NaBH_4_, affording a fluorinated hydroxy ester 10 in 67% yield with 1.4 : 1 dr and 96% ee, or a fluorinated diol 11 in 83% yield with 97% ee, respectively.

Notably, diol 11 was readily converted into a synthetically useful fluorinated 1,3-dioxane 12 or 1,3-dioxan-2-one 13 (ref.^24^) under the action of 1-(dimethoxymethyl)-4-methoxybenzene or triphosgene. A fluorinated oxetane 14 was also obtained from diol 11*via* a selective monotosylation and sequential cyclization process. The versatile diol 11 proved to be a very useful linker that can merge two drugs to form complex fluorine-containing molecules, as exemplified by the efficient installation of fluorinated compounds 15a–15c from drugs (*S*)-naproxen, oxaprozin, and febuxostat.

## Conclusions

In summary, we have developed a highly enantioselective Markovnikov regioselective hydromonofluoroalkyl(methyl)ation of 1,3-dienes by using chiral Ni catalysis, allowing access to various functionalized chiral allylic compounds bearing a CH_2_F, CD_2_F or monofluoroalkyl group at the stereocenter. Remarkably, such a methodology provides a new direction for enantioselective monofluoroalkyl(methyl)ation, and it constitutes a new branch of asymmetric 1,3-diene hydrofunctionalizations. Moreover, this represents the first enantio- and regio-selective Markovnikov hydrofluoroalkylation of olefins. The salient features include broad substrate scope for both aromatic and aliphatic 1,3-dienes, excellent enantio- and regio-selectivity, good functional group tolerance, mild base-free conditions, and diverse product transformations. Further studies in our laboratory will focus on elucidating the reaction mechanism^25^ and developing other asymmetric Markovnikov regioselective hydrofluoroalkylation reactions.

## Data availability

All of the experimental data have been included in the ESI.† Crystallographic data can be obtained from the CCDC (2130032 and 2130034).

## Author contributions

L. Liao and Y. Zhang performed the experiments, and collected and analyzed the data; Z.-W. Wu, Z.-T. Ye, and X.-X. Zhang synthesized some of the 1,3-dienes. J.-S. Yu conceived the idea and directed the project; J.-S. Yu and G. Chen co-wrote the manuscript.

## Conflicts of interest

There are no conflicts to declare.

## Supplementary Material

SC-013-D2SC03958C-s001

SC-013-D2SC03958C-s002
