# Supplementary material for: Nickel-catalyzed regio- and enantio-selective Markovnikov hydromonofluoroalkylation of 1,3-dienes
Source: Chem Sci. 2022 Oct 11;13(42):12519–26. doi: 10.1039/d2sc03958c (PMC9629049; doi:10.1039/d2sc03958c)
Supplement: SC-013-D2SC03958C-s001 [file SC-013-D2SC03958C-s001.pdf]

Electronic Supplementary Material (ESI) for Chemical Science.
This journal is © The Royal Society of Chemistry 2022

Nickel-catalyzed regio- and enantioselective Markovnikov hydromonofluoroalkylation of 1,3-dienes

Ling Liao,[a,#] Ying Zhang,[a,#] Zhong-Wei Wu,[a] Zhong-Tian Ye,[a] Xue-Xin Zhang,[a] Guangying Chen,[b] and Jin-Sheng Yu*[a,b]

[a] *Shanghai Engineering Research Center of Molecular Therapeutics and New Drug Development, School of Chemistry and Molecular Engineering, East China Normal University, Shanghai, 200062, China.*

[b] *Key Laboratory of Tropical Medicinal Resource Chemistry of Ministry of Education, Hainan Normal University, Haikou 571158, China.*

[#] L. Liao and Y. Zhang contributed equally to this work.

*E-mail: jsyu@chem.ecnu.edu.cn

Supporting Information

Table of Contents	Page
1. General information	1
2. Selected conditions for reaction optimization	2-3
3. The correction of serial number of 1,3-dienes **1** and their structures	4
4. Enantioselective Markovnikov hydromonofluoromethylation of 1,3-dienes **1** with FBSM **2**	5-22
5. Enantioselective Markovnikov hydromonofluoroalkylation of 1,3-dienes **1** with **5**	23-36
6. Synthetic utility	36-43
7. Mechanistic studies	44-50
8. X-ray crystallographic data of **3a** and **6t**	51-63
9. NMR spectra	64-303
10. HPLC traces	303-376

1. General information

Reactions were monitored by thin layer chromatography (TLC) using UV light or $KMnO_4$ to visualize the course of reaction. Purification of reaction products was carried out by flash chromatography on silica gel. Chemical yields refer to pure isolated substances. The optical rotation $[\alpha]_D$ was measured using Anton Paar MCP 5500. Infrared (IR) spectra were obtained using a SHIMADZU TRT racer-100. The HRMS spectra were measured on Waters GCT Premier™ or Bruker maXis impact spectrometer using electron spray ionization (ESI) method. Chiral HPLC analysis was performed on a Shimadzu LC-20AD instrument using Daicel chiral columns at 25 °C and a mixture of HPLC-grade hexanes and isopropanol as eluent. ^1H, ^{13}C, and ^{19}F NMR were recorded using a Bruker DPX-400 and 500 MHz spectrometer. Chemical shifts were reported in ppm from tetramethylsilane with the solvent resonance as the internal standard. The following abbreviations were used to designate chemical shift multiplicities: s = singlet, d = doublet, t = triplet, q = quartet, m = multiplet.

Unless mentioned, all reactions were performed under N_2 atmosphere. Anhydrous CH_2Cl_2, and THF were prepared by Innovative Technology PS-MD-5. Anhydrous toluene was prepared by distillation over sodium-benzophenone ketyl prior to use. Absolute MeOH, EtOH and ^{i}PrOH were prepared by first dried over anhydrous Na_2SO_4, then treated by Mg chips, distilled and stored under N_2 atmosphere. $Ni(COD)_2$ was purchased from Sigma-Aldrich and used as received. The (*E*)-1,3-dienes **1a-1ad** were prepared according to the corresponding literatures.[1,2] FBSM **2** were prepared following the reported methods.[3] Diethyl fluoromalonate **5** was commercially available and distillation before use.

List of abbreviation:

Entry	Chemical name	Abbreviation
1	Petroleum ether	PE
2	Ethyl acetate	EtOAc
3	Tetrahydrofuran	THF
4	Ethanol	EtOH
5	Methanol	MeOH
6	Dichloromethane	CH_2Cl_2

[1] For synthesis of **1a-1c**, **1i-1j**, **1n-1o** and **1u-1v**: J. S. Marcum, T. N. Cervarich, R. S. Manan, C. C. Roberts, S. J. Meek, *ACS Catal.* **2019**, *9*, 5881. For synthesis of **1d-1h**, **1k**, **1m**, **1p-1t**, and **1ab**: A. Bhowmik, R. A. Fernandes, *Org. Lett*. **2019**, *21*, 9203.

[2] For synthesis of **1l** and **1ad**: H. T. Dang, V. D. Nguyen, H. H. Pham, H. D. Arman, O. V. Larionov, *Tetrahedron* **2019**, *75*, 3258. For synthesis of **1w**: R. Wang, S.-L. Zhang, *RSC Adv*., **2014**, *4*, 39497. For synthesis of **1ac** and **1x**: Y.-B. Li, F. Chen, S.-Q. Zhu and L.-L. Chu, *Org. Chem. Front.*, **2021**, *8*, 2196. For synthesis of **1y**: M.-M. Li, L. Cheng, L.-J. Xiao, J.-H. Xie, Qi-Lin Zhou, *Angew. Chem. Int. Ed*. **2021**, *60*, 2948. For synthesis of **1z**: D. S, Reddy, *Org. Lett*., **2004**, *6*, 3345. For synthesis of **1aa**: K. Duvvuri, K. R. Dewese, M. M. Parsutkar, S. M. Jing, M. M. Mehta, J. C. Gallucci, T. V. RajanBabu, *J. Am. Chem. Soc*. **2019**, *141*, 7365.

[3] a) U. Sankar, S. Mahalakshmi, K. K. Balasubramanian, *Synlett* **2013**, *24*, 1533; b) M. Urban, M. Franc, M. Hofmanová, I. Císařovéb, J. Veselý, *Org. Biomol. Chem.* **2017**, *15*, 9071.

2. Selected conditions for reaction optimization

Table S1: Further optimization of chiral ligands for hydromonofluoromethylation.[a]

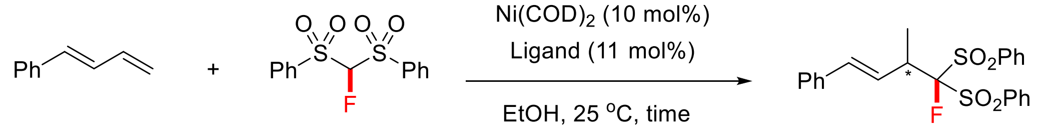

1a (0.15 mmol) **2a** (0.1 mmol) **3a**

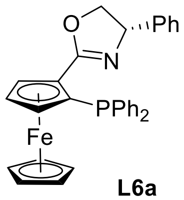

L6a

22 h, 90%, 67% ee

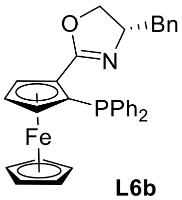

L6b

22 h, 50%, 64% ee

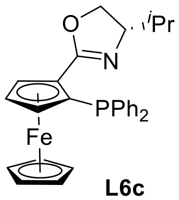

L6c

22 h, 86%, 56% ee

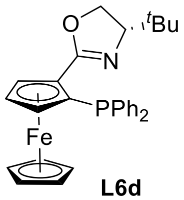

L6d

22 h, 49%, 10% ee

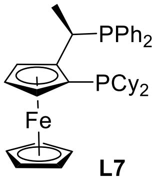

L7

22 h, 50%, 78% ee

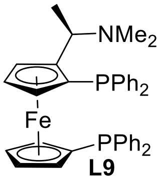

L9

30 h, trace

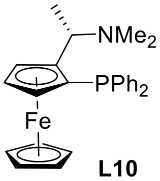

L10

30 h, 40%, 60% ee

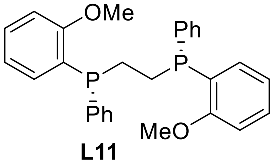

L11

24 h, 6%, 40% ee

[a] Determined by ^{1}H NMR analysis of the crude product using 1,3,5-trimethoxybenzene as internal standard. Ee was determined by chiral HPLC analysis.

Table S2: Selected conditions for optimization of hydromonofluoroalkylation.[a]

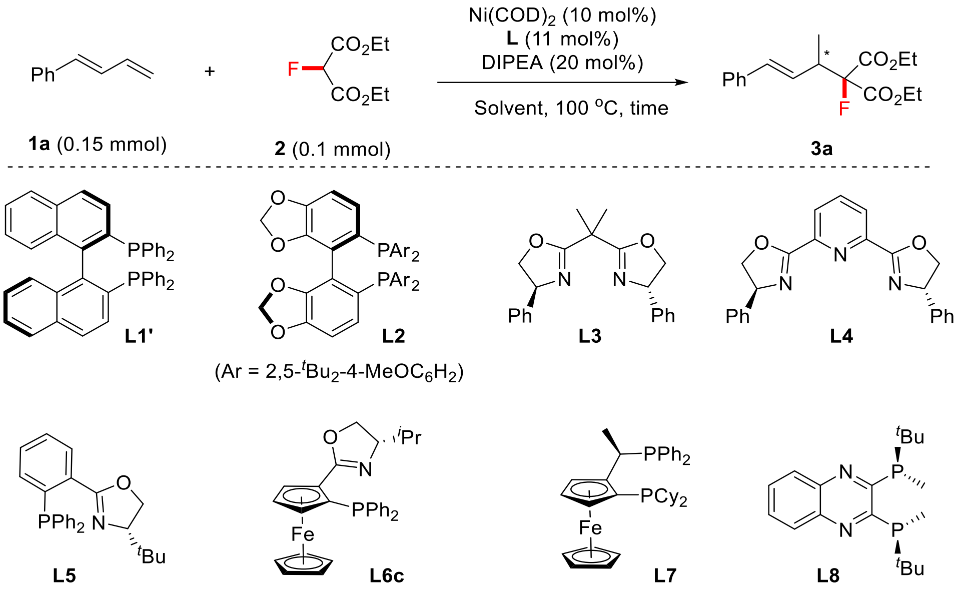

Entry	Ligand	Solvent	Time (h)	Yield (%)	Ee (%)
1	**L1'**	EtOH	4	89	8
2	**L2**	EtOH	8	89	22
3	**L3**	EtOH	36	36	84
4	**L4**	EtOH	21	nr[b]	-
5	**L5**	EtOH	21	89	5
6	**L6c**	EtOH	4	89	68
7	**L7**	EtOH	11	73	23
8	**L8**	EtOH	4	95	92
9[c]	**L8**	EtOH	21	85	97
10[d]	**L8**	EtOH	21	94	96
11[d]	**L8**	THF	21	11	82
12[d]	**L8**	Toluene	21	35	71
13[d]	**L8**	CH_2Cl_2	21	10	80
14[e]	**L8**	EtOH	72	96	96

[a] Reaction conditions: **1a** (0.15 mmol), **2** (0.1 mmol), $Ni(COD)_2$ (10 mol%), ligand (11 mol%), and DIPEA (20 mol%), run at 100 °C in EtOH (1 mL), unless otherwise noted; isolated yield is reported; ee was determined by chiral HPLC analysis. [b] No reaction. [c] At rt. [d] Without the use of DIPEA, at rt. [e] Run on a 0.25 mmol scale using $Ni(COD)_2$ (5 mol%) and **L8** (5.5 mol%) at 50 °C in EtOH (1.5 mL), without DIPEA.

3. The correction of serial number of 1,3-dienes 1 and their structures

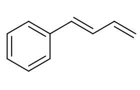

1a

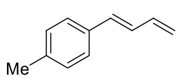

1b

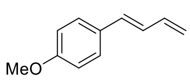

1c

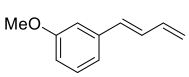

1d

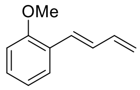

1e

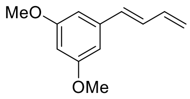

1f

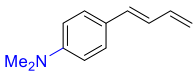

1g

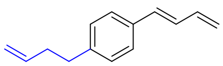

1h

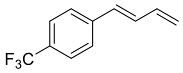

1i

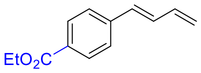

1j

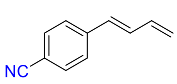

1k

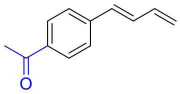

1l

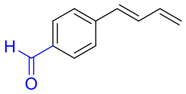

1m

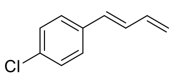

1n

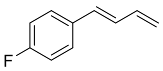

1o

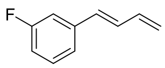

1p

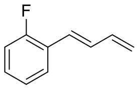

1q

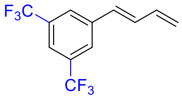

1r

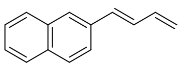

1s

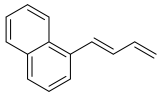

1t

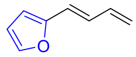

1u

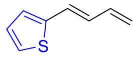

1v

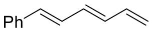

1w

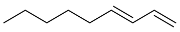

1x

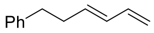

1y

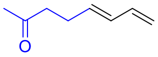

1z

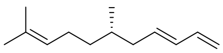

1aa

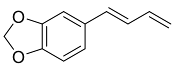

1ab

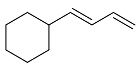

1ac

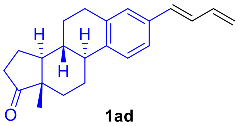

1ad

4. Enantioselective Markovnikov hydromonofluoromethylation of 1,3-dienes with FBSM 2

4.1 General procedure for enantioselective hydromonofluoromethylation of 1,3-dienes with 2

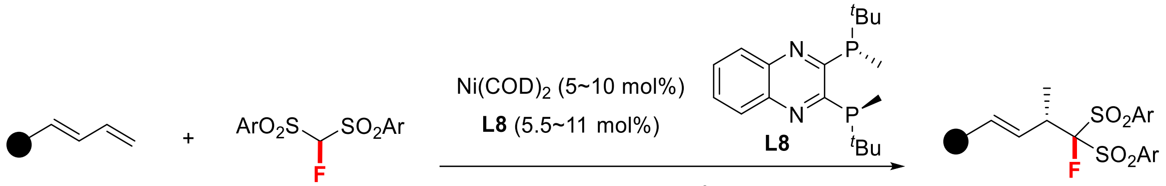

1 (0.375 mmol) **2** (0.25 mmol) **3**

To an oven-dried Schlenk tube equipped with a stirring bar were successively added Ni(COD)$_2$ (3.5 mg, 5 mol%), (*S,S*)-QuinoxP* **L8** (4.6 mg, 5.5 mol%) (or Ni(COD)$_2$ (7.0 mg, 10 mol%), (*S,S*)-QuinoxP* **L8** (9.2 mg, 11 mol%)), 1,3-dienes **1** (0.375 mmol, 1.5 equivs), and FBSM **2** (0.25 mmol), followed by the addition of absolute EtOH (2.5 mL) in a glove box. After it take out from the glove box, the resulting mixture was stirred at 25 °C until full conversion. The reaction was monitored by TLC and GC-MS analysis. After full consumption of **2**, the reaction mixture was concentrated under vacuum to give the crude residue, which was purified by silica gel column chromatography using PE/EtOAc (8:1, v/v) as the eluent to afford the products **3**. (Note: 5 mol% Ni catalyst was used in the cases of product **3a**, **3c**, **3o**, and **3s**; 10 mol% of Ni was used in other cases.) Racemic products **3k**, **3m** and **3z** were prepared using 10 mol% of Ni(COD)$_2$ with a mixed ligand consisting of (*R*,*R*)-quinoxp* and (*S*,*S*)-quinoxp* as the catalyst, and all other racemates **3** were prepared using 10 mol% Ni(COD)$_2$ and 11 mol% dppb.

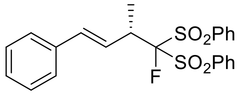

3a

Product **3a** was obtained in 86% yield as a white solid (3 days). m.p. = 136-138 °C; HPLC analysis (Chiralcel OX-H, ^{i}PrOH/hexane = 20/80, 1.0 mL/min, 254 nm; t_r (major) = 17.12 min, t_r (minor) = 19.18 min gave the isomeric composition of the product: 96% ee; $[\alpha]_D^{20}$ = -56.0 (*c* = 0.04, CHCl$_3$); ^{1}H NMR (400 MHz, CDCl$_3$): δ 7.93 (d, *J* = 7.6 Hz, 2H), 7.81 (d, *J* = 7.2 Hz, 2H), 7.70 (t, *J* = 7.6 Hz, 1H), 7.60-7.57 (m, 1H), 7.55-7.51 (m, 2H), 7.44-7.40 (m, 2H), 7.30-7.21 (m, 5H), 6.31-6.22 (m, 2H), 3.53-3.45 (m, 1H), 1.68 (d, *J* = 7.2 Hz, 3H); ^{13}C NMR (100 MHz, CDCl$_3$): δ 136.5, 136.4, 136.0, 135.0, 134.8, 133.7 (d, *J* = 2.0 Hz), 131.0 (d, *J* = 2.0 Hz), 130.8 (d, *J* = 2.0 Hz), 128.8, 128.7, 128.4, 127.8, 126.5, 125.5 (d, *J* = 5.0 Hz), 116.2 (d, *J* = 264.0 Hz), 41.5 (d, *J* = 18.0 Hz), 15.0 (d, *J* = 5.0 Hz); ^{19}F NMR (376 MHz, CDCl$_3$): δ -130.17 (s, 1F); IR (neat): 1448, 1344, 1169, 1151, 1076, 970, 752, 723, 685, 588, 572 cm^{-1}; HRMS (ESI): Exact mass calcd for $C_{23}H_{21}FNaO_4S_2$ [M+Na]$^+$: 467.0758, Found: 467.0769.

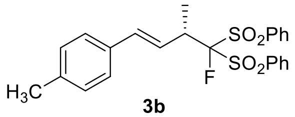

3b

Product **3b** was obtained in 99% yield as a white solid (4 days). m.p. = 118-120 °C; HPLC analysis (Chiralcel OX-H, ^{i}PrOH/hexane = 20/80, 1.0 mL/min, 254 nm; t_r (major) = 18.43 min, t_r (minor) = 20.76min gave the isomeric composition of the product: 94% ee. $[\alpha]_D^{20}$ = -70.9 (c = 0.04, $CHCl_3$); ^{1}H NMR (400 MHz, $CDCl_3$): δ 7.93 (d, J = 8.0 Hz, 2H), 7.80 (d, J = 8.0 Hz, 2H), 7.69 (t, J = 7.2 Hz, 1H), 7.58 (t, J = 7.6 Hz, 1H), 7.52 (t, J = 7.6 Hz, 2H), 7.41 (t, J = 7.6 Hz, 2H), 7.14-7.07 (m, 4H), 6.21-6.20 (m, 2H), 3.51-3.43 (m, 1H), 2.32 (s, 3H), 1.67 (d, J = 7.2 Hz, 3H); ^{13}C NMR (100 MHz, $CDCl_3$): δ 137.6, 136.5, 136.0, 135.0, 134.8, 133.62, 133.60, 131.0 (d, J = 1.8 Hz), 130.7 (d, J = 1.8 Hz), 129.1, 128.8, 128.7, 126.4, 124.4 (d, J = 5.6 Hz), 116.2 (d, J = 264.5 Hz), 41.5 (d, J = 17.8 Hz), 21.1, 15.0 (d, J = 5.0 Hz); ^{19}F NMR (376 MHz, $CDCl_3$): δ -129.94 (s, 1F); IR (neat): 1448, 1339, 1149, 1072, 970, 800, 723, 683, 580, 571 cm^{-1}; HRMS (ESI): Exact mass calcd for $C_{24}H_{23}FNaO_4S_2$ $[M+Na]^+$: 481.0914, Found: 481.0919.

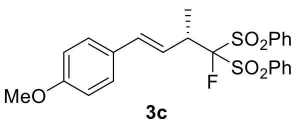

3c

Product **3c** was obtained in 97% yield as a yellow oil (3 days). HPLC analysis (Chiralcel OX-H, ^{i}PrOH/hexane = 20/80, 1.0 mL/min, 254 nm; t_r(major) = 30.87 min, t_r(minor) = 33.36 min gave the isomeric composition of the product: 97% ee. $[\alpha]_D^{20}$ = -161.8 (c = 0.1, $CHCl_3$); ^{1}H NMR (400 MHz, $CDCl_3$): δ 7.93 (d, J = 8.4 Hz, 2H), 7.80 (d, J = 8.4 Hz, 2H), 7.69 (t, J = 7.2 Hz, 1H), 7.58 (t, J = 7.6 Hz, 1H), 7.52 (t, J = 8.0 Hz, 2H), 7.41 (t, J = 8.0 Hz, 2H), 7.17 (d, J = 8.8 Hz, 2H), 6.81 (d, J = 8.4 Hz, 2H), 6.19 (d, J = 15.6 Hz, 1H), 6.09 (dd, J = 15.6, 7.6 Hz, 1H), 3.80 (s, 3H), 3.52-3.43 (m, 1H), 1.67 (d, J = 7.2 Hz, 3H);^{13}C NMR (100 MHz, $CDCl_3$): δ 159.3, 136.5, 135.9, 135.0, 134.7, 133.1 (d, J = 1.4 Hz), 131.0 (d, J = 1.8 Hz), 130.69 (d, J = 1.8 Hz), 129.1, 128.8, 128.6, 127.6, 123.1 (d, J = 5.7 Hz), 116.2 (d, J = 264.3 Hz), 113.8, 55.2, 41.5 (d, J = 17.8 Hz), 15.0 (d, J = 5.0 Hz); ^{19}F NMR (376 MHz, $CDCl_3$): δ -129.95 (s, 1F); IR (neat): 1510, 1448, 1339, 1252, 1167, 1149, 1076, 972, 910, 754, 723, 683, 553 cm^{-1}; HRMS (ESI): Exact mass calcd for $C_{24}H_{23}FNaO_5S_2$ $[M+Na]^+$: 497.0863, Found: 497.0871.

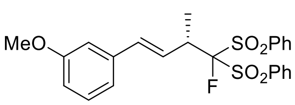

3d

The reaction was carried out at 50 °C for 3 days. Product **3d** was obtained in 86% yield as a white solid. m.p. = 104-106 °C; HPLC analysis (Chiralcel OX-H, ^{i}PrOH/hexane = 20/80, 1.0 mL/min, 254 nm; t_r (major) = 26.92 min, t_r (minor) = 30.99 min gave the isomeric composition of the product: 98% ee. $[\alpha]_D^{20}$ = -35.2 (c = 0.04, $CHCl_3$); ^{1}H NMR (400 MHz, $CDCl_3$): δ 7.93 (d, J = 8.8 Hz, 2H), 7.81 (d, J = 8.4 Hz, 2H), 7.70 (t, J = 7.6 Hz, 1H), 7.59 (t, J = 7.2 Hz, 1H), 7.53 (t, J = 8.0 Hz, 2H), 7.42 (t, J = 8.0 Hz, 2H), 7.20 (t, J = 7.6

Hz, 1H), 6.85 (d, J = 7.6 Hz, 1H), 6.80-6.78 (m, 2H), 6.29-6.19 (m, 2H), 3.81 (s, 3H), 3.53-3.44 (m, 1H), 1.69 (dd, J = 6.8, 0.8 Hz, 3H); ^{13}C NMR (100 MHz, $CDCl_3$): δ 159.6, 137.8, 136.4, 135.9, 135.0, 134.8, 133.6 (d, J = 1.5 Hz), 131.0 (d, J = 1.9 Hz), 130.7 (d, J = 1.9 Hz), 129.4, 128.8, 128.7, 125.8 (d, J = 5.5 Hz), 119.1, 116.1 (d, J = 264.5 Hz), 113.4, 111.8, 55.2, 41.5 (d, J = 17.8 Hz), 14.9 (d, J = 5.0 Hz); ^{19}F NMR (376 MHz, $CDCl_3$): δ -130.21 (s, 1F); IR (neat): 1583, 1448, 1352, 1167, 999, 817, 754, 682, 553 cm^{-1}; HRMS (ESI): Exact mass calcd for $C_{24}H_{23}FNaO_5S_2$ $[M+Na]^+$: 497.0863, Found: 497.0868.

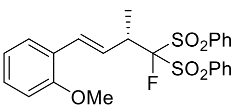

3e

The reaction was carried out at 50 °C for 3 days. Product **3e** was obtained in 93% yield as a colorless oil. HPLC analysis (Chiralcel OX-H, ^{i}PrOH/hexane = 20/80, 1.0 mL/min, 254 nm; t_r (major) = 28.10 min, t_r (minor) = 31.99 min gave the isomeric composition of the product: 98% ee. $[\alpha]_D^{20}$ = -51.4 (c = 0.05, $CHCl_3$); ^{1}H NMR (400 MHz, $CDCl_3$): δ 7.94 (d, J = 8.0 Hz, 2H), 7.82 (d, J = 8.0 Hz, 2H), 7.69 (t, J = 7.6 Hz, 1H), 7.58 (t, J = 8.0 Hz, 1H), 7.53 (t, J = 8.0 Hz, 2H), 7.41 (t, J = 8.4 Hz, 2H), 7.29 (d, J = 7.6 Hz, 1H), 7.21 (t, J = 7.6 Hz, 1H), 6.89 (t, J = 7.6 Hz, 1H), 6.83 (d, J = 8.4 Hz, 1H), 6.62 (d, J = 16.0 Hz, 1H), 6.28 (dd, J = 16.0, 8.0 Hz, 1H), 3.81 (s, 3H), 3.54-3.45 (m, 1H), 1.67 (d, J = 6.8 Hz, 3H); ^{13}C NMR (100 MHz, $CDCl_3$): δ 156.5, 136.6, 136.1, 135.0, 134.7, 131.0 (d, J = 1.8 Hz), 130.8 (d, J = 1.9 Hz), 128.9, 128.8, 128.7, 128.3, 126.9, 125.8 (d, J = 5.5 Hz), 125.5, 120.6, 116.3 (d, J = 264.7 Hz), 110.7, 55.4, 41.9 (d, J = 17.9 Hz), 15.0 (d, J = 5.1 Hz); ^{19}F NMR (376 MHz, $CDCl_3$): δ -130.12 (s, 1F); IR (neat): 1489, 1448, 1340, 1244, 1167, 1151, 1074, 976, 752, 685, 594 cm^{-1}; HRMS (ESI): Exact mass calcd for $C_{24}H_{23}FNaO_5S_2$ $[M+Na]^+$: 497.0863, Found: 497.0873.

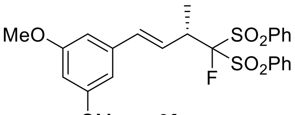

3f

Product **3f** was obtained in 98% yield as a colorless oil (3 days). HPLC analysis (Chiralcel OX-H, ^{i}PrOH/hexane = 20/80, 1.0 mL/min, 254 nm; t_r (major) = 35.41 min, t_r (minor) = 41.05 min gave the isomeric composition of the product: 99% ee. $[\alpha]_D^{20}$ = -148.8 (c = 0.1, $CHCl_3$); ^{1}H NMR (400 MHz, $CDCl_3$): δ 7.92 (d, J = 7.6 Hz, 2H), 7.80 (d, J = 8.4 Hz, 2H), 7.70 (t, J = 7.2 Hz, 1H), 7.60 (t, J = 7.6 Hz, 1H), 7.53 (t, J = 7.6 Hz, 2H), 7.42 (t, J = 8.4 Hz, 2H), 6.40 (d, J = 1.2 Hz, 2H), 6.37 (s, 1H), 6.27-6.16 (m, 2H), 3.78 (s, 6H), 3.53-3.44 (m, 1H), 1.68 (d, J = 6.8 Hz, 3H); ^{13}C NMR (100 MHz, $CDCl_3$): δ 160.8, 138.4, 136.4, 135.9, 135.0, 134.8, 133.7 (d, J= 1.4 Hz), 131.0 (d, J = 1.8 Hz), 130.7 (d, J = 2.0 Hz), 128.8, 128.7, 126.0 (d, J = 5.7 Hz), 116.1 (d, J = 264.4 Hz), 104.6, 110.0, 55.3, 41.4 (d, J = 17.8 Hz), 14.9 (d, J = 5.1 Hz); ^{19}F

NMR (376 MHz, $CDCl_3$): δ -130.15 (s, 1F); IR (neat): 1589, 1448, 1340, 1203, 1149, 1070, 968, 727, 682, 569 cm^{-1}; HRMS (ESI): Exact mass calcd for $C_{25}H_{25}FNaO_6S_2$ $[M+Na]^+$: 527.0969, Found: 527.0971.

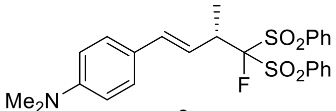

3g

The reaction was carried out at 50 °C for 4 days. Product **3g** was obtained in 48% yield as a yellow oil. HPLC analysis (Chiralcel OX-H, *i*PrOH/hexane = 20/80, 1.0 mL/min, 230 nm; t_r (major) = 34.39 min, t_r (minor) = 37.16 min gave the isomeric composition of the product: 93% ee. $[\alpha]_D^{20}$ = -148.9 (*c* = 0.03, $CHCl_3$); ^1H NMR (400 MHz, $CDCl_3$) δ 7.94 (d, *J* = 8.3 Hz, 2H), 7.80 (d, *J* = 8.3 Hz, 2H), 7.69 (t, *J* = 7.5 Hz, 1H), 7.58 (t, *J* = 7.5 Hz, 1H), 7.53 (t, *J* = 7.9 Hz, 2H), 7.40 (t, *J* = 7.9 Hz, 2H), 7.10 (d, *J* = 8.8 Hz, 2H), 6.62 (d, *J* = 8.8 Hz, 2H), 6.12 (d, *J* = 15.8 Hz, 1H), 5.95 (dd, *J* = 15.8, 7.8 Hz, 1H), 3.49-3.40 (m, 1H), 2.95 (s, 6H), 1.67 (d, *J* = 6.9 Hz, 3H); ^{13}C NMR (100 MHz, $CDCl_3$): δ 150.2, 136.7, 136.1, 134.9, 134.6, 133.7, 131.1 (d, *J* = 1.8 Hz), 130.7 (d, *J* = 1.8 Hz), 128.7, 128.6, 127.5, 124.9, 120.8 (d, *J* = 5.5 Hz), 116.5 (d, *J* = 265.6 Hz), 112.2, 41.6 (d, *J* = 17.9 Hz), 40.4, 15.1 (d, *J* = 4.8 Hz); ^{19}F NMR (376 MHz, $CDCl_3$): δ -129.38 (s, 1F); IR (neat): 1448, 1348, 1166, 1151, 1078, 754, 684, 582, 570 cm^{-1}; HRMS (ESI): Exact mass calcd for $C_{25}H_{27}FNO_4S_2$ $[M+H]^+$: 488.1360, Found: 488.1351.

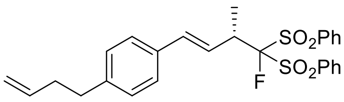

3h

Product **3h** was obtained in 68% yield as a colorless oil (3 days). HPLC analysis (Chiralcel OX-H, *i*PrOH/hexane = 20/80, 1.0 mL/min, 254 nm; t_r (major) = 14.15 min, t_r (minor) = 15.92 min gave the isomeric composition of the product: 95% ee; $[\alpha]_D^{20}$ = -110.4(*c* = 0.07, $CHCl_3$); ^1H NMR (400 MHz, $CDCl_3$) δ 7.93 (d, *J* = 8.5 Hz, 2H), 7.80 (d, *J* = 8.5 Hz, 2H), 7.69 (t, *J* = 7.5 Hz, 1H), 7.58 (t, *J* = 7.5 Hz, 1H), 7.52 (t, *J* = 7.9 Hz, 2H), 7.41 (t, *J* = 7.9 Hz, 2H), 7.20-7.05 (m, 4H), 6.22 (d, *J* = 3.4 Hz, 2H), 5.89-5.79 (m, 1H), 5.06-5.00 (m, 1H), 4.99-4.96 (m, 1H), 3.52-3.45 (m, 1H), 2.71-2.67 (m, 2H), 2.38-2.20 (m, 2H), 1.67 (dd, *J* = 7.1, 1.1 Hz, 3H); ^{13}C NMR (100 MHz, $CDCl_3$): δ 141.6, 137.89, 136.6, 136.0, 135.0, 134.7, 134.1, 133.6 (d, *J* = 1.5 Hz), 131.0 (d, *J* = 1.8 Hz), 130.7 (d, *J* = 2.0 Hz), 128.8, 128.7, 128.5, 126.5, 124.6 (d, *J* = 5.5 Hz), 116.2 (d, *J* = 266.1 Hz), 115.0, 41.6 (d, *J* = 17.9 Hz), 35.4, 35.0, 15.0 (d, *J* = 5.1 Hz); ^{19}F NMR (376 MHz, $CDCl_3$): δ -130.00 (s, 1F); IR (neat): 1639, 1583, 1448, 1338, 1151, 1080, 972, 752, 723, 684, 584, 572 cm^{-1}; HRMS (ESI): Exact mass calcd for $C_{27}H_{28}FO_4S_2$ $[M+H]^+$: 499.1408, Found: 499.1408.

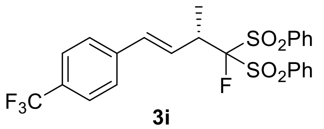

3i

Product **3i** was obtained in 94% yield as a white solid (4 days). m.p. = 102-104 °C; HPLC analysis (Chiralcel OX-H, ^{i}PrOH/hexane = 15/85, 1.0 mL/min, 254 nm; t_r (major) = 15.36 min, t_r (minor) = 17.13 min gave the isomeric composition of the product: 97% ee. $[\alpha]_D^{20}$ = - 104.6 (*c* = 0.08, $CHCl_3$); ^{1}H NMR (400 MHz, $CDCl_3$): δ 7.91 (d, *J* = 7.6 Hz, 2H), 7.80 (d, *J* = 7.6 Hz, 2H), 7.70 (t, *J* = 7.6 Hz, 1H), 7.61 (t, *J* = 7.6 Hz, 1H), 7.55-7.51 (m, 4H), 7.43 (t, *J* = 7.6 Hz, 2H), 7.37 (d, *J* = 8.0 Hz, 2H), 6.46 (dd, *J* = 16, 7.6 Hz, 1H), 6.32 (d, *J* = 15.6 Hz, 1H), 3.58-3.49 (m, 1H), 1.68 (d, *J* = 6.8 Hz, 3H); ^{13}C NMR (100 MHz, $CDCl_3$): δ 139.9, 136.3, 135.8, 135.1, 134.9, 132.3, 131.0 (d, *J* = 1.7 Hz), 130.7 (d, *J* = 1.9 Hz), 129.6 (q, *J* = 24.0 Hz), 128.9, 128.8, 128.4 (d, *J* = 5.9 Hz), 126.7, 125.4 (q, *J* = 3.8 Hz), 124.1(q, *J* = 270.1 Hz) 115.9 (d, *J* = 265.1 Hz), 41.5 (d, *J* = 17.9 Hz), 14.8 (d, *J* = 5.2 Hz); ^{19}F NMR (376 MHz, $CDCl_3$): δ -62.47 (s, 3F), -130.79 (s, 1F); IR (neat): 1614, 1448, 1325, 1167, 1066, 974, 816, 754, 685, 574 cm^{-1}; HRMS (ESI): Exact mass calcd for $C_{24}H_{20}F_4NaO_4S_2$ $[M+Na]^+$: 535.0631, Found: 535.0639.

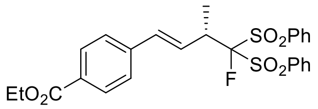

3j

Product **3j** was obtained in 82% yield as a colorless oil (3 days). HPLC analysis (Chiralcel AD-H, ^{i}PrOH/hexane = 20/80, 1.0 mL/min, 254 nm; t_r (major) = 25.55min, t_r (minor) = 23.69 min gave the isomeric composition of the product: 96% ee; $[\alpha]_D^{20}$ = -261.9 (*c* = 0.05, $CHCl_3$); ^{1}H NMR (400 MHz, $CDCl_3$) δ 7.97-7.91 (m, 2H), 7.80 (d, *J* = 8.4 Hz, 2H), 7.70 (t, *J* = 7.6 Hz, 1H), 7.59 (t, *J* = 7.6 Hz, 1H), 7.53 (t, *J* = 7.8 Hz, 2H), 7.42 (t, *J* = 7.8 Hz, 2H), 7.30 (d, *J* = 8.3 Hz, 2H), 6.41 (dd, *J* = 15.9, 7.5 Hz, 1H), 6.31 (d, *J* = 15.9 Hz, 1H), 4.38 (q, *J* = 7.1 Hz, 2H), 3.58-3.50 (m, 1H), 1.69 (d, *J* = 6.9 Hz, 3H), 1.40 (t, *J* = 7.1 Hz, 3H); ^{13}C NMR (100 MHz, $CDCl_3$): 166.3, 140.7, 136.4, 135.9, 135.1, 134.9, 132.7 (d, *J* = 1.5 Hz), 131.0 (d, *J* = 1.8 Hz), 130.7 (d, *J* = 2.0 Hz), 129.7, 129.6, 128.8, 128.7, 128.2 (d, *J* = 5.7 Hz), 126.3, 116.0 (d, *J* = 266.5 Hz), 60.9, 41.4 (d, *J* = 18.0 Hz), 14.8 (d, *J* = 5.3 Hz), 14.3; ^{19}F NMR (376 MHz, $CDCl_3$): δ -130.59 (s, 1F); IR (neat): 1448, 1367, 1168, 1153, 1020, 974, 754, 725, 684, 586, 576 cm^{-1}; HRMS (ESI): Exact mass calcd for $C_{26}H_{26}FO_6S_2$ $[M+H]^+$: 517.1149, Found: 517.1151.

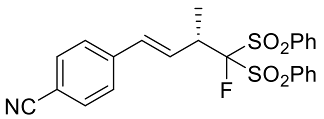

3k

Product **3k** was obtained in 90% yield as a colorless oil (3 days). HPLC analysis (Chiralcel OD-H, ^{i}PrOH/hexane = 20/80, 1.0 mL/min, 230 nm; t_r (major) = 24.09 min, t_r (minor) = 27.25 min gave the isomeric composition of the product: 98% ee; $[\alpha]_D^{20}$ = -277.1 (*c* = 0.03, $CHCl_3$); ^{1}H NMR (400 MHz, $CDCl_3$) δ 7.88 (d, *J* = 8.5 Hz, 2H), 7.80 (d, *J* = 8.5 Hz, 2H), 7.70 (t, *J* = 7.5 Hz, 1H), 7.63-7.56 (m, 3H), 7.52 (t, *J* = 7.8 Hz, 2H), 7.44 (t, *J* = 7.9 Hz, 2H), 7.37 (d, *J* = 8.3 Hz, 2H), 6.55 (dd, *J* = 15.9, 7.7 Hz, 1H), 6.34 (d, *J* = 15.9

Hz, 1H), 3.60-3.51 (m, 1H), 1.68-1.66 (m, 3H); ^{13}C NMR (100 MHz, $CDCl_3$): δ 140.9, 136.0 , 135.7, 135.1, 134.9, 132.2, 131.9 (d, *J* = 1.5 Hz), 130.8 (d, *J* = 1.8 Hz), 130.7 (d, *J* = 2.0 Hz), 129.7 (d, *J* = 5.9 Hz), 128.9, 128.7, 127.0, 118.8, 115.8 (d, *J* = 267.0 Hz), 110.9, 41.4 (d, *J* = 18.1 Hz), 14.8 (d, *J* = 5.3 Hz); ^{19}F NMR (376 MHz, $CDCl_3$): δ -131.00 (s, 1F); IR (neat): 1448, 1313, 1168, 1153, 1078, 974, 754, 723, 684, 586, 569 cm^{-1}; HRMS (ESI): Exact mass calcd for $C_{24}H_{21}FNO_4S_2$ $[M+H]^+$: 470.0891, Found: 470.0889.

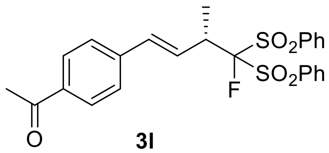

3l

Product **3l** was obtained in 90% yield as a yellow oil (3 days). HPLC analysis (Chiralcel OX-H, ^{i}PrOH/hexane = 40/60, 1.0 mL/min, 254 nm; t_r (major) = 29.43 min, t_r (minor) = 26.82 min gave the isomeric composition of the product: 98% ee; $[\alpha]_D^{20}$ = -192.4 (*c* = 0.04, $CHCl_3$); ^{1}H NMR (400 MHz, $CDCl_3$) δ 7.92-7.87 (m, 4H), 7.80 (d, *J* = 8.5 Hz, 2H), 7.70 (t, *J* = 7.5 Hz, 1H), 7.60 (t, *J* = 7.5 Hz, 1H), 7.53 (t, *J* = 7.9 Hz, 2H), 7.43 (t, *J* = 7.9 Hz, 2H), 7.34 (d, *J* = 8.3 Hz, 2H), 6.47 (dd, *J* = 15.9, 7.7 Hz, 1H), 6.33 (d, *J* = 15.9 Hz, 1H), 3.59-3.50 (m, 1H), 2.59 (s, 3H), 1.69 (dd, *J* = 7.1, 1.2 Hz, 3H); ^{13}C NMR (100 MHz, $CDCl_3$): δ 197.4, 141.0, 136.3, 136.2, 135.8, 135.1, 134.9, 132.6 (d, *J* = 1.6 Hz), 130.9 (d, *J* = 1.8 Hz), 130.7 (d, *J* = 2.0 Hz), 128.8, 128.7, 128.6, 126.5, 116.0 (d, *J* = 266.6 Hz), 41.4 (d, *J* = 18.0 Hz), 26.5, 14.8 (d, *J* = 5.3 Hz); ^{19}F NMR (376 MHz, $CDCl_3$): δ -130.64 (s, 1F); IR (neat): 1448, 1313, 1168, 1153, 1078, 972, 754, 725, 684, 584, 574 cm^{-1}; HRMS (ESI): Exact mass calcd for $C_{25}H_{24}FO_5S_2$ $[M+H]^+$: 487.1044, Found: 487.1041.

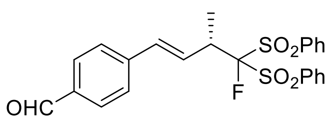

3m

Product **3m** was obtained in 71% yield as a colorless oil (3 days). HPLC analysis (Chiralcel OD-H, ^{i}PrOH/hexane = 20/80, 1.0 mL/min, 254 nm; t_r (major) = 26.84 min, t_r (minor) = 24.33 min gave the isomeric composition of the product: 90% ee; $[\alpha]_D^{20}$ = -72.9676 (*c* = 0.02, $CHCl_3$); ^{1}H NMR (400 MHz, $CDCl_3$) δ 9.98 (s, 1H), 7.91 (d, *J* = 8.5 Hz, 2H), 7.82-7.80 (m, 4H), 7.73-7.68 (m, 1H), 7.63-7.58 (m, 1H), 7.53 (t, *J* = 7.9 Hz, 2H), 7.46-7.42 (m, 4H), 6.54 (dd, *J* = 15.9, 7.8 Hz, 1H), 6.36 (d, *J* = 15.9 Hz, 1H), 3.60-3.51 (m, 1H), 1.69 (dd, *J* = 8.0, 0.8 Hz, 3H); ^{13}C NMR (100 MHz, $CDCl_3$): δ 191.6, 142.5, 136.3, 135.9, 135.6, 135.1, 134.9, 132.5 (d, *J* = 1.5 Hz), 131.0 (d, *J* = 1.8 Hz), 130.8 (d, *J* = 1.9 Hz), 130.0, 129.4 (d, *J* = 5.9 Hz), 128.9, 128.8, 127.0, 116.0 (d, *J* = 266.7 Hz), 41.5 (d, *J* = 18.1 Hz), 14.8 (d, *J* = 5.3 Hz); ^{19}F NMR (376 MHz, $CDCl_3$): δ -130.86 (s, 1F); IR (neat): 1448, 1313, 1168, 1153, 1080, 974, 754, 725, 684, 576, 561 cm^{-1}; HRMS (ESI): Exact mass calcd for $C_{24}H_{22}FO_5S_2$ $[M+H]^+$: 473.0887, Found: 473.0886.

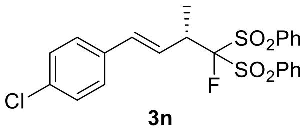
3n

Product **3n** was obtained in 87% yield as a white solid (4 days). m.p. = 99-102 °C; HPLC analysis (Chiralcel OX-H, ^{i}PrOH/hexane = 20/80, 1.0 mL/min, 254 nm; t_r (major) = 17.47 min, t_r (minor) = 20.04 min gave the isomeric composition of the product: 97% ee. $[\alpha]_D^{20}$ = -90.7 (*c* = 0.06, $CHCl_3$); ^1H NMR (400 MHz, $CDCl_3$): δ 7.91 (d, *J* = 8.0 Hz, 2H), 7.80 (d, *J* = 8.0 Hz, 2H), 7.69 (t, *J* = 7.6 Hz, 1H), 7.59 (t, *J* = 7.2 Hz, 1H), 7.52 (t, *J* = 7.6 Hz, 2H), 7.42 (t, *J* = 7.6 Hz, 2H), 7.24 (d, *J* = 8.0 Hz, 2H), 7.18 (d, *J* = 8.4 Hz, 2H), 6.32-6.20 (m, 2H), 3.54-3.46 (m, 1H), 1.67 (d, *J* = 7.2 Hz, 3H); ^{13}C NMR (100 MHz, $CDCl_3$): δ 136.3, 135.8, 135.1, 134.90, 134.86, 133.4, 132.4 (d, *J* = 1.5 Hz), 130.9 (d, *J* = 1.8 Hz), 130.7 (d, *J* = 1.9 Hz), 128.8, 128.7, 128.5, 127.7, 126.2 (d, *J* = 5.7 Hz), 116.0 (d, *J* = 264.7 Hz), 41.4 (d, *J* = 17.9 Hz), 14.8 (d, *J* = 5.1 Hz); ^{19}F NMR (376 MHz, $CDCl_3$): δ -130.46 (s, 1F); IR (neat): 1497, 1448, 1337, 1167, 1088, 972, 752, 682, 586, 532 cm^{-1}; HRMS (ESI): Exact mass calcd for $C_{23}H_{20}ClFNaO_4S_2$ $[M+Na]^+$: 501.0368, Found: 501.0365.

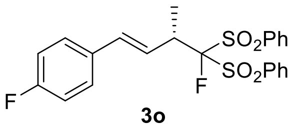
3o

Product **3o** was obtained in 74% yield as a white solid (4 days). m.p. = 76-79 °C; HPLC analysis (Chiralcel OX-H, ^{i}PrOH/hexane = 20/80, 1.0 mL/min, 205 nm; t_r (major) = 19.55 min, t_r (minor) = 22.49 min gave the isomeric composition of the product: 99% ee. $[\alpha]_D^{20}$ = -38.5 (*c* = 0.03, $CHCl_3$); ^1H NMR (400 MHz, $CDCl_3$): δ 7.92 (d, *J* = 8.0 Hz, 2H), 7.81 (d, *J* = 8.0 Hz, 2H), 7.70 (t, *J* = 7.6 Hz, 1H), 7.60 (t, *J* = 7.6 Hz, 1H), 7.53 (t, *J* = 7.6 Hz, 2H), 7.43 (t, *J* = 7.6 Hz, 2H), 7.27-7.21 (m, 1H), 7.03 (d, *J* = 7.6 Hz, 1H), 6.94-6.91 (m, 2H), 6.33-6.21 (m, 2H), 3.55-3.47 (m, 1H), 1.68 (d, *J* = 7.2 Hz, 3H); ^{13}C NMR (100 MHz, $CDCl_3$): δ 162.3 (d, *J* = 245.7 Hz), 135.3, 135.8, 135.0, 134.8, 132.5 (d, *J* = 3.3 Hz), 132.4, 130.9 (d, *J* = 1.8 Hz), 130.7 (d, *J* = 2.0 Hz), 128.7 (d, *J* = 12.3 Hz), 128.0 (d, *J* = 8.0 Hz), 125.24 (d, *J* = 2.3 Hz), 125.18 (d, *J* = 2.3 Hz), 116.0 (d, *J* = 264.5 Hz), 115.3 (d, *J* = 21.5 Hz), 41.4 (d, *J* = 17.9 Hz), 14.9 (d, *J* = 5.1 Hz); ^{19}F NMR (376 MHz, $CDCl_3$): δ -113.95 (s, 1F), -130.40 (s, 1F); IR (neat): 1508, 1448, 1344, 1229, 1151, 1078, 1074, 972, 754, 723, 684, 638, 582 cm^{-1}; HRMS (ESI): Exact mass calcd for $C_{23}H_{20}F_2NaO_4S_2$ $[M+Na]^+$: 485.0663, Found: 485.0672.

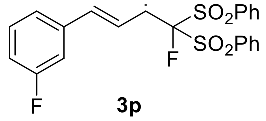
3p

The reaction was carried out at 60 °C for 4 days. Product **3p** was obtained in 92% yield as a colorless oil. HPLC analysis (Chiralcel OX-H, ^{i}PrOH/hexane = 20/80, 1.0 mL/min, 254 nm; t_r (major) = 16.41 min, t_r (minor) = 18.48 min gave the isomeric composition of the product: 96% ee. $[\alpha]_D^{20}$ = -78.8 (*c* = 0.06, $CHCl_3$); ^1H NMR (400 MHz,

CDCl3): δ 7.92 (d, *J* = 8.0 Hz, 2H), 7.81 (d, *J* = 8.0 Hz, 2H), 7.70 (t, *J* = 7.6 Hz, 1H), 7.60 (t, *J* = 7.6 Hz, 1H), 7.53 (t, *J* = 7.6 Hz, 2H), 7.43 (t, *J* = 7.6 Hz, 2H), 7.27-7.21 (m, 1H), 7.03 (d, *J* = 7.6 Hz, 1H), 6.94-6.91 (m, 2H), 6.33-6.21 (m, 2H), 3.55-3.47 (m, 1H), 1.68 (d, *J* = 7.2 Hz, 3H); ^{13}C NMR (100 MHz, $CDCl_3$): δ 162.9 (d, *J* = 243.9 Hz), 138.7 (d, *J* = 7.6 Hz), 136.4, 135.9, 135.1, 134.9, 132.6 (dd, *J* = 4.2, 2.0 Hz), 131.0 (d, *J* = 2.1 Hz), 130.7 (d, *J* = 2.1 Hz), 130.0, 129.9 (d, *J* = 8.4 Hz), 128.8 (d, *J* = 9.8), 127.0 (d, *J* = 5.8 Hz), 122.4 (d, *J* = 2.8 Hz), 116.1 (d, *J* = 264.9 Hz), 114.6 (d, *J* = 21.2 Hz), 112.9 (d, *J* = 21.7 Hz), 41.4 (d, *J* = 17.8 Hz), 14.9 (d, *J* = 5.1 Hz); ^{19}F NMR (376 MHz, $CDCl_3$): δ -113.54 (s, 1F), -130.54 (s, 1F); IR (neat): 1583, 1448, 1346, 1167, 1151, 1078, 974, 754, 684, 571 cm^{-1}; HRMS (ESI): Exact mass calcd for $C_{23}H_{20}F_2NaO_4S_2$ $[M+Na]^+$: 485.0663, Found: 485.0662.

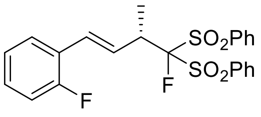

3q

The reaction was carried out at 60 °C for 4 days. Product **3q** was obtained in 99% yield as a colorless oil. HPLC analysis (Chiralcel OX-H, ^{i}PrOH/hexane = 20/80, 1.0 mL/min, 254 nm; t_r (major) = 16.33 min, t_r (minor) = 18.71 min gave the isomeric composition of the product: 94% ee. $[\alpha]_D^{20}$ = -33.55 (*c* = 0.07, $CHCl_3$); ^{1}H NMR (400 MHz, $CDCl_3$): δ 7.92 (d, *J* = 8.0 Hz, 2H), 7.81 (d, *J* = 7.6 Hz, 2H), 7.68 (t, *J* = 7.6 Hz, 1H), 7.57 (t, *J* = 7.6 Hz, 1H), 7.52 (t, *J* = 8.0 Hz, 2H), 7.41 (t, *J* = 7.6 Hz, 2H), 7.36 (t, *J* = 7.6 Hz, 1H), 7.22-7.17 (m, 1H), 7.06 (d, *J* = 7.6 Hz, 1H), 7.01-6.96 (m, 1H), 6.47-6.38 (m, 2H), 3.54-3.46 (m, 1H), 1.68 (d, *J* = 7.2 Hz, 3H); ^{13}C NMR (100 MHz, $CDCl_3$): δ 159.9 (d, *J* = 247.7 Hz), 136.2, 135.8, 134.9 (d, *J* = 22.8 Hz), 130.9 (d, *J* = 1.8 Hz), 130.6 (d, *J* = 1.9 Hz), 129.1 (d, *J* = 8.3 Hz), 128.8, 128.7, 127.8 (t, *J* = 4.8 Hz), 127.3 (d, *J* = 3.5 Hz), 125.8 (d, *J* = 4.0 Hz), 124.2, 124.1, 124.0 (d, *J* = 3.4 Hz), 115.9 (d, *J* = 264.9 Hz), 115.4 (d, *J* = 21.8 Hz), 41.7 (d, *J* = 17.8 Hz), 14.8 (d, *J* = 5.3 Hz); ^{19}F NMR (376 MHz, $CDCl_3$): δ -118.40 (s, 1F), -130.50 (s, 1F); IR (neat): 1487, 1448, 1340, 1230, 1167, 1151, 1080, 972, 752, 685, 592 cm^{-1}; HRMS (ESI): Exact mass calcd for $C_{23}H_{20}F_2NaO_4S_2$ $[M+Na]^+$: 485.0663, Found: 485.0674.

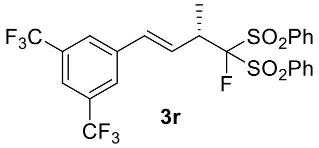

3r

Product **3r** was obtained in 49% yield as a colorless oil (3 days). HPLC analysis (Chiralcel OX-H, ^{i}PrOH/hexane = 20/80, 1.0 mL/min, 254 nm; t_r (major) = 6.86 min, t_r (minor) = 7.28 min gave the isomeric composition of the product: 95% ee. $[\alpha]_D^{20}$ = -10.5 (*c* = 0.02, $CHCl_3$); ^{1}H NMR (300 MHz, $CDCl_3$) δ 7.93 (d, *J* = 8.3 Hz, 2H), 7.82 (d, *J* = 8.3 Hz, 2H), 7.73-7.68 (m, 2H), 7.65-7.7.59 (m, 3H), 7.57-7.52 (m, 2H), 7.47-7.42 (m, 2H), 6.41 (d, *J* = 16.0 Hz, 1H), 6.32 (dd, *J* = 15.9, 6.9 Hz, 1H), 3.68-3.56 (m, 1H), 1.72 (d, *J* = 7.0 Hz, 3H); ^{13}C NMR (100 MHz, $CDCl_3$): δ 138.5, 136.4, 135.9, 135.2, 135.0, 131.9 (q, *J* = 33.3 Hz),

131.0 (d, J = 2.0 Hz), 130.8 (d, J = 2.0 Hz), 129.9 (d, J = 5.8 Hz), 129.5, 128.9 (d, J = 5.0 Hz), 126.3 (d, J = 2.6 Hz), 123.22 (q, J = 272.8 Hz), 121.3-121.1 (m), 115.9 (d, J = 266.6 Hz), 41.19 (d, J = 18.0 Hz), 14.78 (d, J = 5.4 Hz); ^{19}F NMR (376 MHz, $CDCl_3$): δ -62.95 (s, 6F), -131.11 (s, 1F); IR (neat): 1379, 1313, 1278, 11539, 1080, 970, 896, 759, 725, 682 cm^{-1}; HRMS (ESI): Exact mass calcd for $C_{25}H_{19}F_7O_4S_2Na$ [M+Na]$^+$: 603.0505, Found: 603.0508.

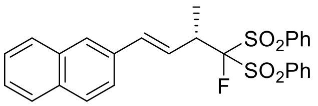
3s

Product **3s** was obtained in 97% yield as a white solid (3 days). m.p. = 124-127 °C; HPLC analysis (Chiralcel OX-H, ^{i}PrOH/hexane = 20/80, 1.0 mL/min, 254 nm; t_r (major) = 24.44 min, t_r (minor) = 27.99 min gave the isomeric composition of the product: 99% ee. $[\alpha]_D^{20}$ = -81.7 (c = 0.04, $CHCl_3$); ^{1}H NMR (400 MHz, $CDCl_3$): δ 7.95 (d, J = 7.6 Hz, 2H), 7.83-7.73 (m, 5H), 7.69 (t, J = 8.0 Hz, 1H), 7.59 (s, 1H), 7.57-7.51 (m, 3H), 7.47-7.37 (m, 5H), 6.44-6.34 (m, 2H), 3.61-3.52 (m, 1H), 1.73 (d, J = 6.8 Hz, 3H); ^{13}C NMR (100 MHz, $CDCl_3$): δ 136.6, 136.1, 135.0, 134.8, 133.9, 133.8 (d, J = 2.0 Hz), 133.4, 133.1, 131.1 (d, J = 2.0 Hz), 130.8 (d, J = 2.0 Hz), 128.8, 128.7, 128.1, 127.9, 127.6, 126.4, 126.3, 125.96, 125.89 (d, J = 5.0 Hz), 123.7, 116.3 (d, J = 264.0 Hz), 41.6 (d, J = 18.0 Hz), 15.0 (d, J = 5.0 Hz); ^{19}F NMR (376 MHz, $CDCl_3$): δ -130.09 (s, 1F); IR (neat): 1448, 1346, 1169, 1151, 1078, 812, 752, 684, 584, 571, 546 cm^{-1}; HRMS (ESI): Exact mass calcd for $C_{27}H_{23}FNaO_4S_2$ [M+Na]$^+$: 517.0914, Found: 517.0927.

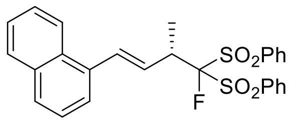
3t

The reaction was carried out at 60 °C for 3 days. Product **3t** was obtained in 98% yield as a colorless oil (3 days). HPLC analysis (Chiralcel OX-H, ^{i}PrOH/hexane = 20/80, 1.0 mL/min, 205 nm; t_r (major) = 21.39 min, t_r (minor) = 25.11 min gave the isomeric composition of the product: 91% ee. $[\alpha]_D^{20}$ = -63.4 (c = 0.07, $CHCl_3$); ^{1}H NMR (400 MHz, $CDCl_3$): δ 7.95 (d, J = 8.0 Hz, 3H), 7.85-7.77 (m, 4H), 7.70 (t, J = 7.6 Hz, 1H), 7.55-7.46 (m, 6H), 7.42 (t, J = 7.6 Hz, 1H), 7.35 (t, J = 7.6 Hz, 2H), 7.06 (d, J = 15.6 Hz, 1H), 6.38-6.32 (m, 1H), 3.69-3.60 (m, 1H), 1.75 (d, J = 6.8 Hz, 3H); ^{13}C NMR (100 MHz, $CDCl_3$): δ 136.4, 136.0, 135.0, 134.7, 134.2, 133.4, 131.2, 131.0 (d, J = 1.7 Hz), 130.9, 130.7 (d, J = 2.0 Hz), 128.8, 128.696 (d, J = 5.3 Hz), 128.695, 128.5, 128.1, 126.0, 125.7, 125.6, 124.2, 123.6, 116.2 (d, J = 265.0 Hz), 41.8 (d, J = 17.9 Hz), 15.1 (d, J = 5.0 Hz); ^{19}F NMR (376 MHz, $CDCl_3$): δ -130.16 (s, 1F); IR (neat): 1448, 1346, 1313, 1167, 1078, 972, 798, 777, 721, 684, 575, 553 cm^{-1}; HRMS (ESI): Exact mass calcd for $C_{27}H_{23}FNaO_4S_2$ [M+Na]$^+$: 517.0914, Found: 517.0915.

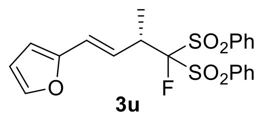

3u

The reaction was carried out at 50 °C for 3 days. Product **3u** was obtained in 85% yield as a yellow solid. m.p. = 100-103 °C; HPLC analysis (Chiralcel OX-H, ^{i}PrOH/hexane = 20/80, 1.0 mL/min, 254 nm; t_r (major) = 21.03 min, t_r (minor) = 24.41 min gave the isomeric composition of the product: 93% ee. $[\alpha]_D^{20}$ = -84.2 (*c* = 0.07, $CHCl_3$); ^1H NMR (400 MHz, $CDCl_3$): δ 7.94 (d, *J* = 8.0 Hz, 2H), 7.78 (d, *J* = 7.6 Hz, 2H), 7.70 (t, *J* = 7.2 Hz, 1H), 7.60-7.51 (m, 3H) 7.39 (t, *J* = 8.0 Hz, 2H), 7.29 (s, 1H), 6.33-6.32 (m, 1H), 6.15 (d, *J* = 3.2 Hz, 1H), 6.08 (d, *J* = 3.2 Hz, 2H), 3.52-3.45 (m, 1H), 1.69 (d, *J* = 7.2 Hz, 3H); ^{13}C NMR (100 MHz, $CDCl_3$): δ 151.9, 142.0, 136.5, 136.0, 135.0, 134.6, 131.1 (d, *J* = 2.0 Hz), 130.7 (d, *J* = 2.0 Hz), 128.8, 128.6, 124.0 (d, *J* = 5.8 Hz), 121.9 (d, *J* = 1.5 Hz), 116.2 (d, *J* = 264.2 Hz), 111.2, 108.3, 40.9 (d, *J* = 17.6 Hz), 14.5 (d, *J* = 5.3 Hz); ^{19}F NMR (376 MHz, $CDCl_3$): δ -130.13 (s, 1F); IR (neat): 1448, 1339, 1169, 1151, 1078, 966, 754, 685, 690, 576 cm^{-1}; HRMS (ESI): Exact mass calcd for $C_{21}H_{19}FNaO_5S_2$ $[M+Na]^+$: 457.0550, Found: 457.0553.

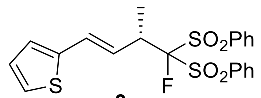

3v

The reaction was carried out at 50 °C for 3 days. Product **3v** was obtained in 98% yield as a yellow solid. m.p. = 111-114 °C; HPLC analysis (Chiralcel OX-H, ^{i}PrOH/hexane = 20/80, 1.0 mL/min, 254 nm; t_r (major) = 22.49 min, t_r (minor) = 25.71 min gave the isomeric composition of the product: 98% ee. $[\alpha]_D^{20}$ = -59.1 (*c* = 0.07, $CHCl_3$); ^1H NMR (400 MHz, $CDCl_3$): δ 7.94 (d, *J* = 8.0 Hz, 2H), 7.79 (d, *J* = 8.0 Hz, 2H), 7.70 (t, *J* = 7.2 Hz, 1H), 7.59 (t, *J* = 7.6 Hz, 1H), 7.53 (t, *J* = 7.6 Hz, 2H), 7.42 (t, *J* = 8.0 Hz, 2H), 7.14 (d, *J* = 5.2 Hz, 1H), 6.92 (t, *J* = 3.6 Hz, 1H), 6.86 (d, *J* = 3.6 Hz, 1H), 6.36 (d, *J* = 7.6 Hz, 1H), 5.98 (dd, *J* = 15.6, 7.6 Hz, 1H), 3.54-3.45 (m, 1H), 1.69 (d, *J* = 7.2 Hz, 3H); ^{13}C NMR (100 MHz, $CDCl_3$): δ 141.3, 136.4, 135.8, 135.0, 134.8, 131.1 (d, *J* = 1.8 Hz), 130.6 (d, *J* = 1.9 Hz), 128.8, 128.7, 127.2, 126.9 (d, *J* = 1.5 Hz), 125.9, 124.8 (d, *J* = 5.6 Hz), 124.6, 116.1 (d, *J* = 264.3 Hz), 41.1 (d, *J* = 17.5 Hz), 14.7 (d, *J* = 5.0 Hz); ^{19}F NMR (376 MHz, $CDCl_3$): δ -129.83 (s, 1F); IR (neat): 1583, 1448, 1344, 1151, 1078, 960, 754, 684, 570 cm^{-1}; HRMS (ESI): Exact mass calcd for $C_{21}H_{19}FNaO_4S_3$ $[M+Na]^+$: 473.0322, Found: 473.0321.

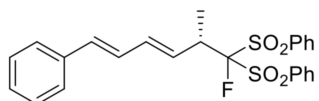

3w

Product **3w** was obtained in 99% yield as a colorless oil (3 days). HPLC analysis (Chiralcel AD-H, ^{i}PrOH/hexane = 20/80, 1.0 mL/min, 254 nm; t_r (major) = 19.29 min, t_r (minor) = 22.40 min gave the isomeric composition of the product: 97% ee. $[\alpha]_D^{20}$ = -69.7 (*c* = 0.06, $CHCl_3$); ^1H NMR (400 MHz, $CDCl_3$): δ 7.91 (d, *J* = 8.0 Hz, 2H), 7.82 (d, *J* = 7.6 Hz, 2H), 7.69 (t, *J* = 7.2 Hz, 1H), 7.63 (t, *J* = 8.0 Hz, 1H), 7.53 (t, *J* = 8.0

Hz, 2H), 7.47 (t, *J* = 7.6 Hz, 2H), 7.37-7.29 (m, 4H), 7.24-7.21 (m, 1H), 6.63 (dd, *J* = 15.6, 10.4 Hz, 1H), 6.45 (d, *J* = 15.6 Hz, 1H), 6.07 (dd, *J* = 15.6, 10.4 Hz, 1H), 5.90 (dd, *J* = 14.8, 8.0 Hz, 1H), 3.46-3.37 (m, 1H), 1.64 (d, *J* = 7.2 Hz, 3H); ^{13}C NMR (100 MHz, $CDCl_3$): δ 136.9, 136.4, 135.9, 135.0, 134.8, 134.2, 132.8, 131.0 (d, *J* = 1.8 Hz), 130.8 (d, *J* = 2.0 Hz), 129.3 (d, *J* = 5.7 Hz), 128.8, 128.7, 128.6, 128.1, 127.6, 126.3, 116.1 (d, *J* = 264.6 Hz), 41.3 (d, *J* = 17.9 Hz), 14.9 (d, *J* = 4.9 Hz); ^{19}F NMR (376 MHz, $CDCl_3$): δ -130.18 (s, 1F); IR (neat): 1448, 1340, 1169, 1151, 1080, 991, 750, 685, 580, 569 cm^{-1}; HRMS (ESI): Exact mass calcd for $C_{25}H_{23}FNaO_4S_2$ $[M+Na]^+$: 493.0914, Found: 493.0916.

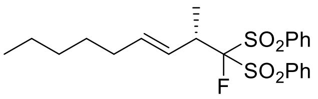

3x

The reaction was carried out at 70 °C for 3 days. Product **3x** was obtained in 67% yield as a yellow oil. HPLC analysis (Chiralcel OD-H, ^{i}PrOH/hexane = 10/90, 1.0 mL/min, 230 nm; t_r (major) = 10.72 min, t_r (minor) = 11.41 min gave the isomeric composition of the product: 93% ee. $[\alpha]_D^{20}$ = -7.2 (*c* = 0.06, $CHCl_3$); ^{1}H NMR (400 MHz, $CDCl_3$): δ 7.90 (d, *J* = 8.4 Hz, 2H), 7.82 (d, *J* = 8.4 Hz, 2H), 7.71-7.64 (m, 2H), 7.54-7.47 (m, 4H), 5.54-5.48 (m, 1H), 5.39-5.32 (m, 1H), 3.31-3.22 (m, 1H), 1.93-1.87 (m, 2H), 1.56 (d, *J* = 6.4 Hz, 3H), 1.30-1.22 (m, 6H), 0.88 (t, *J* = 6.8 Hz, 3H); ^{13}C NMR (100 MHz, $CDCl_3$): δ 136.7, 136.0, 135.7 (d, *J* = 1.4 Hz), 134.9, 134.7, 130.9 (d, *J* = 1.9 Hz), 130.7 (d, *J* = 2.1 Hz), 128.7, 128.6, 125.4 (d, *J* = 5.6 Hz), 116.2 (d, *J* = 263.9 Hz), 41.3 (d, *J* = 17.8 Hz), 32.3, 31.2, 28.5, 22.4, 15.2 (d, *J* = 5.1 Hz), 14.0; ^{19}F NMR (376 MHz, $CDCl_3$): δ -129.78 (s, 1F); IR (neat): 2922, 1583, 1448, 1336, 1151, 972, 754, 723, 682, 570 cm^{-1}; HRMS (ESI): Exact mass calcd for $C_{22}H_{27}FNaO_4S_2$ $[M+Na]^+$: 461.1227, Found: 461.1236.

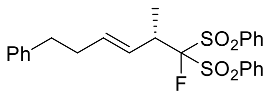

3y

The reaction was carried out at 70 °C for 5 days. Product **3y** was obtained in 81% yield as a white solid. m.p. = 110-113 °C; HPLC analysis (Chiralcel OD-H, ^{i}PrOH/hexane = 20/80, 1.0 mL/min, 205 nm; t_r (major) = 15.14 min, t_r (minor) = 17.59 min gave the isomeric composition of the product: 90% ee. $[\alpha]_D^{20}$ = -19.9 (*c* = 0.1, $CHCl_3$); ^{1}H NMR (400 MHz, $CDCl_3$): δ 7.89 (d, *J* = 8.0 Hz, 2H), 7.80 (d, *J* = 8.0 Hz, 2H), 7.70-7.63 (m, 2H), 7.53-7.45 (m, 4H), 7.29-7.25 (m, 2H), 7.20-7.12 (m, 3H), 5.57 (dd, *J* = 15.6, 8.0 Hz, 1H), 5.41 (dt, *J* = 15.2, 6.4 Hz, 1H), 3.30-3.21 (m, 1H), 2.59 (t, *J* = 7.6 Hz, 2H), 2.26-2.20 (m, 2H), 1.54 (d, *J* = 7.2 Hz, 3H); ^{13}C NMR (100 MHz, $CDCl_3$): δ 141.5, 136.6, 136.0, 134.9, 134.8, 134.5 (d, *J* = 1.5 Hz), 130.9 (d, *J* = 1.9 Hz), 130.7 (d, *J* = 2.0 Hz), 128.7, 128.6, 128.4, 128.2, 126.3 (d, *J* = 5.6 Hz), 125.8, 116.1 (d, *J* = 264.2 Hz), 41.3 (d, *J* = 17.9 Hz), 35.3, 34.0, 15.2 (d, *J* = 5.2 Hz); ^{19}F NMR (376 MHz, $CDCl_3$): δ -

129.93 (s, 1F); IR (neat): 1448, 1339, 1166, 1149, 1078, 972, 752, 683, 576, 561 cm^{-1}; HRMS (ESI): Exact mass calcd for $C_{25}H_{25}FNaO_4S_2$ $[M+Na]^+$: 495.1071, Found: 495.1072.

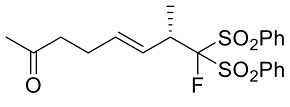

3z

The reaction was carried out at 70 °C for 4 days.Product **3z** was obtained in 69% yield as a colorless oil. HPLC analysis (Chiralcel OX-H, ^{i}PrOH/hexane = 40/60, 1.0 mL/min, 205 nm; t_r (major) = 16.88 min, t_r (minor) = 13.81 min gave the isomeric composition of the product: 94% ee; $[\alpha]_D^{20}$ = -10.7 (*c* = 0.01, $CHCl_3$); ^1H NMR (400 MHz, $CDCl_3$) δ 7.87 (d, *J* = 8.5 Hz, 2H), 7.81 (d, *J* = 8.5 Hz, 2H), 7.72-7.61 (m, 2H), 7.54-7.48 (m, 4H), 5.67-5.61 (m, 1H), 5.46-5.40 (m, 1H), 3.29-3.20 (m, 1H), 2.51-2.40 (m, 2H), 2.25-2.18 (m, 2H), 2.14 (s, 3H), 1.52 (dd, *J* = 7.1, 1.3 Hz, 3H); ^{13}C NMR (100 MHz, $CDCl_3$): δ 207.9, 136.4, 136.0, 134.9, 134.8, 133.4 (d, *J* = 1.4 Hz), 130.8 (d, *J* = 1.8 Hz), 130.7 (d, *J* = 2.1 Hz), 128.8, 128.7, 126.5 (d, *J* = 5.9 Hz), 116.1 (d, *J* = 266.4 Hz), 42.6, 41.2 (d, *J* = 18.0 Hz), 29.9, 26.3, 15.1 (d, *J* = 5.4 Hz); ^{19}F NMR (376 MHz, $CDCl_3$): δ -130.28 (s, 1F); IR (neat): 1448, 1323, 1276, 1107, 1078, 974, 754, 725, 686, 576, 563 cm^{-1}; HRMS (ESI): Exact mass calcd for $C_{21}H_{24}FO_5S_2$ $[M+H]^+$: 439.1044, Found: 439.1040.

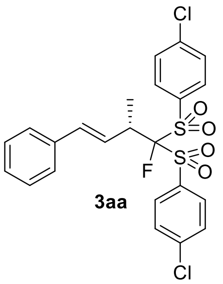

3aa

The reaction was carried out at 50 °C for 5 days. Product **3aa** was obtained in 94% yield as a yellow solid. m.p. = 135-137 °C; HPLC analysis (Chiralcel AD-H, ^{i}PrOH/hexane = 15/85, 1.0 mL/min, 254 nm; t_r (major) = 18.17 min, t_r (minor) = 18.95 min gave the isomeric composition of the product: 97% ee. $[\alpha]_D^{20}$ = -45.5 (*c* = 0.04, $CHCl_3$); ^1H NMR (400 MHz, $CDCl_3$): δ 7.89 (d, *J* = 8.4 Hz, 2H), 7.73 (d, *J* = 8.0 Hz, 2H), 7.53 (d, *J* = 8.4 Hz, 2H), 7.38 (d, *J* = 8.4 Hz, 2H), 7.33-7.29 (m, 2H), 7.26-7.25 (m, 1H), 7.22-7.20 (m, 2H), 6.27 (d, *J* = 16.0 Hz, 1H), 6.13 (dd, *J* = 16.0, 7.6 Hz, 1H), 3.54-3.45 (m, 1H), 1.68 (d, *J* = 7.2 Hz, 3H); ^{13}C NMR (100 MHz, $CDCl_3$): δ 142.3, 142.2, 136.1, 134.8, 134.3, 133.9 (d, *J* = 1.5 Hz), 132.5 (d, *J* = 1.9 Hz), 132.1 (d, *J* = 2.1 Hz), 129.2, 129.1, 128.6, 128.0, 126.4, 124.9 (d, *J* = 5.4 Hz), 116.3 (d, *J* = 264.1 Hz), 41.2 (d, *J* = 17.5 Hz), 14.7 (d, *J* = 5.2 Hz); ^{19}F NMR (376 MHz, $CDCl_3$): δ -130.52 (s, 1F); IR (neat): 2361, 2341, 1576, 1475, 1344, 1168, 1153, 1091, 970, 824, 752, 694, 567 cm^{-1}; HRMS (ESI): Exact mass calcd for $C_{23}H_{19}Cl_2FNaO_4S_2$ $[M+Na]^+$: 534.9978, Found: 534.9973.

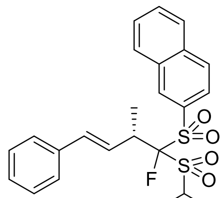

3ab

The reaction was carried out at 50 ºC for 5 days. Product **3ab** was obtained in 99% yield as a yellow solid. HPLC analysis (Chiralcel AD-H, ^{i}PrOH/hexane = 40/60, 1.0 mL/min, 254 nm; t_r (major) = 19.23 min, t_r (minor) = 27.98 min gave the isomeric composition of the product: 95% ee. $[\alpha]_D^{20}$ = -52.2 (*c* = 0.05, $CHCl_3$); ^{1}H NMR (400 MHz, $CDCl_3$): δ 8.42 (s, 1H), 8.24 (s, 1H), 7.82-7.76 (m, 4H), 7.72 (d, *J* = 8.0 Hz, 1H), 7.67-7.53 (m, 6H), 7.47 (t, *J* = 7.6 Hz, 1H), 7.21-7.16 (m, 5H), 6.40-6.30 (m, 2H), 3.75-3.67 (m, 1H), 1.79 (d, *J* = 6.8 Hz, 3H); ^{13}C NMR (100 MHz, $CDCl_3$): δ 136.4, 135.7, 135.6, 133.7, 133.4, 133.2, 133.0, 132.8, 131.6, 131.5, 129.9, 129.8, 129.6, 129.5, 128.7 (d, *J* = 5.6 Hz), 128.4, 127.8, 127.7, 127.6, 127.5, 126.4, 125.7 (d, *J* = 5.6 Hz), 124.7 (d, *J* = 2.6 Hz), 124.4 (d, *J* = 3.2 Hz), 116.6 (d, *J* = 264.2 Hz), 41.5 (d, *J* = 17.8 Hz), 15.2 (d, *J* = 5.0 Hz); ^{19}F NMR (376 MHz, $CDCl_3$): δ -130.00 (s, 1F); IR (neat): 1340, 1167, 1130, 1067, 968, 864, 815, 748, 636, 571 cm^{-1}; HRMS (ESI): Exact mass calcd for $C_{31}H_{25}FNaO_4S_2$ $[M+Na]^+$: 567.1071, Found: 567.1065.

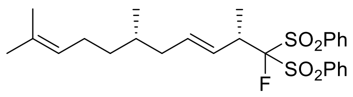

3ac

The reaction was carried out at 50 ºC for 5 days. Product **3ac** was obtained in 42% yield as a colorless oil. HPLC analysis (Chiralcel OX-H, ^{i}PrOH/hexane = 5/95, 1.0 mL/min, 205 nm; t_r(major) = 26.17 min, t_r(minor) = 28.82 min gave the isomeric composition of the product: 95% de. $[\alpha]_D^{20}$ = -6.4 (*c* = 0.03, $CHCl_3$); ^{1}H NMR (400 MHz, $CDCl_3$): δ 7.91-7.88 (m, 2H), 7.83-7.80 (m, 2H), 7.71-7.63 (m, 2H), 7.54-7.46 (m, 4H), 5.56-5.51 (m, 1H), 5.38-5.30 (m, 1H), 5.10-5.06 (m, 1H), 3.31-3.23 (m, 1H), 1.98-1.89 (m, 3H), 1.81-1.74 (m, 1H), 1.68 (s, 3H), 1.60 (s, 3H), 1.58 (dd, *J* = 6.8, 0.8 Hz, 3H), 1.45-1.37 (m, 1H), 1.30-1.23 (m, 1H), 1.14-1.05 (m, 1H), 0.82 (d, *J* = 6.8 Hz, 3H); ^{13}C NMR (100 MHz, $CDCl_3$): δ 136.7, 135.9, 134.9, 134.7, 134.2 (d, *J* = 1.4 Hz), 131.1, 130.9 (d, *J* = 1.9 Hz), 130.8 (d, *J* = 2.0 Hz), 128.8, 128.6, 126.8 (d, *J* = 5.5 Hz), 124.7, 116.1 (d, *J* = 264.0 Hz), 41.6 (d, *J* = 17.9 Hz), 39.7, 36.5, 32.4, 25.7, 25.5, 19.3, 17.6, 15.4 (d, *J* = 5.0 Hz); ^{19}F NMR (376 MHz, $CDCl_3$): δ -129.60 (s, 1F); IR (neat): 2962, 1448, 1348, 1217, 1168, 1080, 754, 725, 648, 578 cm^{-1}; HRMS (ESI): Exact mass calcd for $C_{26}H_{33}FNaO_4S_2$ $[M+Na]^+$: 515.1697, Found: 515.1708.

4.2 Transformations of product 3a

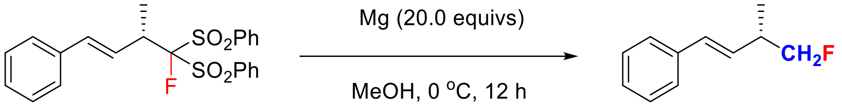

To a solution of product **3a** (44.4 mg, 0.1 mmol) in absolute MeOH (1.0 mL) was added activated Mg (20 equivs) in one portion at 0 °C under N_2 atmosphere. The reaction was stirred at 0 °C until full consumption of **3a** monitored by TLC and GC-MS analysis (about 12 h). After adding water (3 mL) and dilute HCl (3 mL, 2 N), the reaction mixture was extracted with CH_2Cl_2 (5 mL × 3). The combined organic layers were dried over Na_2SO_4, and concentrated under reduced pressure to afford the crude residue, which was purified by flash chromatography with PE as the eluent was used as the eluent) to provide the desired product **4a** in 98% yield as a colorless oil. HPLC analysis (Chiralcel OD-H, ^{i}PrOH/hexane = 0/100, 1.0 mL/min, 254 nm; t_r (major) = 17.71 min, t_r (minor) = 13.90 min gave the isomeric composition of the product: 96% ee; $[\alpha]_D^{20}$ = -1.3 (*c* = 0.02, $CHCl_3$); ^{1}H NMR (400 MHz, $CDCl_3$): δ 7.37-7.35 (m, 2H), 7.32-7.28 (m, 2H), 7.25-7.20 (m, 1H), 6.58 (d, *J* = 16.0 Hz, 1H), 6.13 (dd, *J* = 16.0, 7.6 Hz, 1H), 4.46-4.44 (m, 1H), 4.34-4.24 (m, 1H), 2.81-2.67 (m, 1H), 1.16 (d, *J* = 6.8 Hz, 3H); ^{13}C NMR (100 MHz, $CDCl_3$): δ 137.2, 130.7, 130.5 (d, *J* = 6.6 Hz), 128.5, 127.3, 126.1, 87.3 (d, *J* = 171.3 Hz), 37.9 (d, *J* = 18.9 Hz), 15.8 (d, *J* = 6.1 Hz); ^{19}F NMR (376 MHz, CDCl3): δ -219.64 (s, 1F); IR (neat): 2966, 1490, 1215, 1047, 966, 752, 721, 692, 678, 669 cm-1; HRMS (EI): Exact mass calcd for $C_{11}H_{13}F$ $[M]^+$: 164.0998, Found: 164.0996.

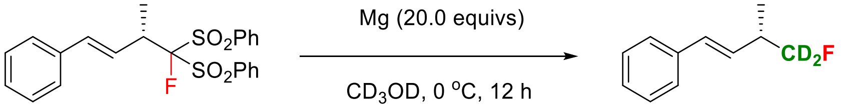

To a solution of product **3a** (44.4 mg, 0.1 mmol) in absolute CD_3OD (1.0 mL) was added activated Mg (48.6 mg, 20 equivs) in one portion at 0 °C under N_2 atmosphere. The reaction was stirred at 0 °C until full consumption of **3a** monitored by TLC and GC-MS analysis (about 12 h). After adding water (3 mL) and dilute HCl (3 mL), the reaction mixture was extracted with CH_2Cl_2 (5 mL × 3). The combined organic layers were dried over Na_2SO_4, and concentrated under reduced pressure to afford the crude residue, which was purified by flash chromatography with PE as the eluent was used as the eluent) to provide the desired product **D-4a** in 91% yield as a colorless oil. HPLC analysis (Chiralcel OD-H, ^{i}PrOH/hexane = 0/100, 1.0 mL/min, 254 nm; t_r (major) = 12.31 min, t_r (minor) = 15.24 min gave the

isomeric composition of the product: 98% ee; $[\alpha]_D^{20}$ = -2.8 (c = 0.02, $CHCl_3$); ^{1}H NMR (400 MHz, $CDCl_3$): δ 7.37-7.34 (m, 2H), 7.32-7.28 (m, 2H), 7.24-7.20 (m, 1H), 6.47 (d, J = 14.8 Hz, 1H), 6.13 (dd, J = 16.0, 8.4 Hz, 1H), 2.78-2.67 (m, 1H), 1.16 (dd, J = 6.8 Hz, 5.6 Hz, 3H); ^{13}C NMR (100 MHz, $CDCl_3$): δ 137.2, 130.7, 130.5 (d, J = 7.0 Hz), 128.5, 127.3, 126.1, 86.6 (dt, J = 169 Hz, 22.0 Hz), 37.7 (d, J = 20.0 Hz), 15.8 (d, J = 6.0 Hz); ^{19}F NMR (376 MHz, $CDCl_3$): δ -220.94~-221.01 (m, 1F); IR (neat): 1494, 1448, 1078, 958, 744, 721, 692 cm^{-1}; HRMS (EI): Exact mass calcd for $C_{11}H_{11}D_2F$ $[M]^+$: 166.1126, Found: 166.1121.

4.3 General procedure for the tandem synthesis of α-monofluoromethyl allylic compounds

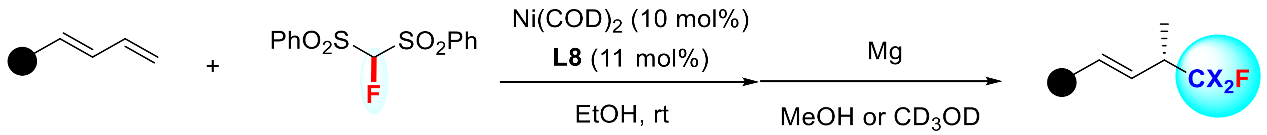

1 (0.375 mmol) **2a** (0.25 mmol) X = H or D **4** or **D-4**

To an oven-dried Schlenk tube equipped with a stirring bar were successively added $Ni(COD)_2$ (7.0 mg, 10 mol%), (S,S)-QuinoxP* **L8** (9.2 mg, 11 mol%), 1,3-dienes **1** (0.375 mmol, 1.5 equivs), and FBSM **2a** (78.6 mg, 0.25 mmol), followed by the addition of absolute EtOH (2.5 mL) in a glove box. After it take out from the glove box, the resulting mixture was stirred at 25 °C until full conversion of 2 indicated by TLC or GC-MS analysis. The reaction mixture was concentrated under vacuum, and the residue was filtrated over a short pad of silica gel to give the crude product **3**, which was used for the next reductive desulfonylation step.

To a solution of above crude products **3** in absolute **MeOH** (2.5 mL) or **CD_3OD** (2.5 mL) was added activated Mg (48.6 mg, 20 equivs) in one portion at 0 °C under N_2 atmosphere. The reaction was stirred at 0 °C (**4a**, **4d** and **D-4a**) or rt (**4b**, **4c, D-4b, D-4c and D-4d**) until full consumption of **3** monitored by TLC and GC-MS analysis (about 12 h). After adding water (3 mL) and dilute HCl (3 mL, 2 N), the reaction mixture was extracted with CH_2Cl_2 (10 mL × 2). The combined organic layers were dried over Na_2SO_4, and concentrated under reduced pressure. The crude residue was purified by flash chromatography with PE as the eluent (in the case of **4b** and **D-4b**, PE/CH_2Cl_2 (10:1, v/v) was used as the eluent) to provide the desired **CH_2F-** or **CD_2F-**containing products **4** or **D-4**.

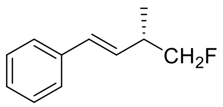

4a

Product **4a** was obtained in 86% yield from **1a** as a colorless oil. HPLC analysis (Chiralcel OD-H, ^{i}PrOH/hexane = 0/100, 1.0 mL/min, 254 nm; t_r (major) = 17.71 min, t_r (minor) = 13.90 min gave the isomeric composition of the product: 96% ee. The NMR spectra of **4a** is consistent with that of the product obtained via one-step desulfonylation reaction in Section 4.2.

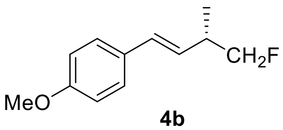

4b

Product **4b** was obtained in 88% yield from **1c** as a colorless oil. HPLC analysis (Chiralcel OD-H, ^{i}PrOH/hexane = 0.1/99.9, 1.0 mL/min, 254 nm; t_r (major) = 18.29 min, t_r (minor) = 17.22 min) gave the isomeric composition of the product: 96% ee. $[\alpha]_D^{20}$ = -8.3 (c = 0.06, $CHCl_3$); ^{1}H NMR (400 MHz, $CDCl_3$): δ 7.31-7.24 (m, 2H), 6.86-6.82 (m, 2H), 6.41 (d, J = 16.0 Hz, 1H), 5.97 (dd, J = 16.0, 7.2 Hz, 1H), 4.44-4.34 (m, 1H), 4.32-4.22 (m, 1H), 3.79 (s, 3H), 2.78-2.63 (m, 1H), 1.15 (dd, J = 6.8, 1.2 Hz, 3H); ^{13}C NMR (100 MHz, $CDCl_3$): δ 158.9, 130.1, 130.0, 128.2 (d, J = 6.7 Hz), 127.2, 113.9, 87.4 (d, J = 171.1 Hz), 55.2, 37.8 (d, J = 18.9 Hz), 15.9 (d, J = 5.9 Hz); ^{19}F NMR (376 MHz, $CDCl_3$): δ -219.34 (s, 1F); IR (neat): 2964, 1606, 1510 1246, 1033, 966, 910, 806, 750, 669, 532 cm^{-1}; HRMS (ESI): Exact mass calcd for $C_{12}H_{16}OF$ $[M+H]^+$: 195.1185, Found: 195.1179.

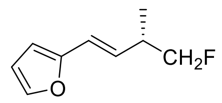

4c

Product **4c** was obtained in 39% yield from **1n** as a colorless oil. HPLC analysis (Chiralcel OD-H, ^{i}PrOH/hexane = 0/100, 1.0 mL/min, 254 nm; t_r (major) = 8.96 min, t_r (minor) = 8.36 min) gave the isomeric composition of the product: 96% ee. $[\alpha]_D^{20}$ = -3.2 (c = 0.03, $CHCl_3$); ^{1}H NMR (400 MHz, $CDCl_3$): δ 7.32 (d, J = 2.0 Hz, 1H), 6.36-6.35 (m, 1H), 6.32-6.28 (m, 1H), 6.19 (d, J = 3.2 Hz, 1H), 6.08 (dd, J = 16.0, 0.8 Hz, 1H), 4.44-4.22 (m, 2H), 2.76-2.65 (m, 1H), 1.69 (dd, J = 6.8, 0.8 Hz, 3H); ^{13}C NMR (100 MHz, $CDCl_3$): δ 152.7, 141.6, 129.3 (d, J = 7.0 Hz), 119.3, 111.2, 107.1, 84.1 (d, J = 117.0 Hz), 37.6 (d, J = 19.0 Hz), 15.7 (d, J = 6.0 Hz); ^{19}F NMR (376 MHz, $CDCl_3$): δ -219.35 (s, 1F); IR (neat): 2933, 1215, 1115, 981, 962, 883, 796, 667, 594 cm^{-1}; HRMS (ESI): Exact mass calcd for $C_9H_{12}F$ $[M+H]^+$: 155.0872, Found: 155.0866.

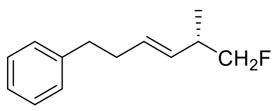

4d

Product **4d** was obtained in 86% yield from **1r** as a colorless oil. HPLC analysis (Chiralcel OD-H + OD-H, ^{i}PrOH/hexane = 0/100, 1.0 mL/min, 205 nm; t_r (major) = 18.81 min, t_r (minor) = 20.60 min gave the isomeric composition of the product: 93% ee. $[\alpha]_D^{20}$ = -4.4 (c = 0.06, $CHCl_3$); ^{1}H NMR (400 MHz, $CDCl_3$): δ 7.30-7.26 (m, 2H), 7.20-7.16 (m, 3H), 5.60-5.52 (m, 1H), 5.36-5.29 (m, 1H), 4.32-4.09 (m, 2H), 2.69-2.65 (m, 2H), 2.56-

2.45 (m, 1H), 2.35-2.29 (m, 2H), 1.01 (dd, J = 6.8, 1.2 Hz, 3H); ^{13}C NMR (100 MHz, $CDCl_3$): δ 141.8, 131.0 (d, J = 6.9 Hz), 130.7, 128.5, 128.2, 125.7, 87.6 (d, J = 170.5 Hz), 37.4 (d, J = 18.8 Hz), 35.9, 34.5, 15.9 (d, J = 5.9 Hz); ^{19}F NMR (376 MHz, $CDCl_3$): δ -219.30 (s, 1F); IR (neat): 2927, 1454, 1388, 1215, 1006, 970, 908, 750, 698, 667, 570 cm^{-1}; HRMS (EI): Exact mass calcd for $C_{13}H_{17}F$ [M]$^+$: 192.1306, Found: 192.1309.

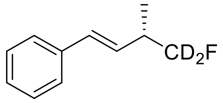

D-4a

Product **D-4a** was obtained in 94% yield from **1a** as a colorless oil. HPLC analysis (Chiralcel OD-H, ^{i}PrOH/hexane = 0/100, 1.0 mL/min, 254 nm; t_r (major) = 12.31 min, t_r (minor) = 15.24 min gave the isomeric composition of the product: 98% ee. The NMR spectra of **D-4a** is consistent with that of the product obtained via one-step desulfonylation reaction in Section 4.2.

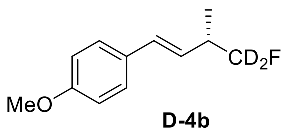

D-4b

Product **D-4b** was obtained in 68% yield from **1c** as a colorless oil. HPLC analysis (Chiralcel IC, ^{i}PrOH/hexane = 1/100, 1.0 mL/min, 254 nm; t_r (major) = 5.71 min, t_r (minor) = 5.14 min) gave the isomeric composition of the product: 98% ee. $[\alpha]_D^{20}$ = -0.4 (c = 0.02, $CHCl_3$); ^{1}H NMR (400 MHz, $CDCl_3$): δ 7.30-7.28 (m, 2H), 6.85-6.83 (m, 2H), 6.44-6.40 (m, 1H), 5.98 (dd, J = 16.0, 7.6 Hz, 1H), 3.80 (s, 3H), 2.76-2.65 (m, 1H), 1.15 (dd, J = 6.8, 1.2 Hz, 3H); ^{13}C NMR (100 MHz, $CDCl_3$): δ 159.0, 130.1, 130.0, 128.2 (d, J = 7.0 Hz), 127.3, 113.9, 87.4 (m), 55.3, 37.6 (d, J = 19.0 Hz), 15.9 (d, J = 5.0 Hz) (Note: the peak for CD_2F is missed in the ^{13}C NMR spectrum, possibly due to low intensity); ^{19}F NMR (376 MHz, $CDCl_3$): δ -220.69~-220.77 (m, 1F); IR (neat): 1606, 1510, 1463, 1246, 1174, 1033, 964, 904, 804, 727, 650, 530 cm^{-1}; HRMS (EI): Exact mass calcd for $C_{12}H_{13}D_2OF$ [M]$^+$: 196.1230, Found: 196.1227.

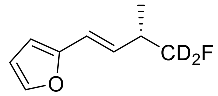

D-4c

Product **D-4c** was obtained in 54% yield from **1n** as a colorless oil. HPLC analysis (Chiralcel OD-H, ^{i}PrOH/hexane = 0/100, 1.0 mL/min, 254 nm; t_r (major) = 8.51 min, t_r (minor) = 9.12 min) gave the isomeric composition of the product: 97% ee. $[\alpha]_D^{20}$ = -1.3 (c = 0.03, $CHCl_3$); ^{1}H NMR (400 MHz, $CDCl_3$): δ 7.32 (s, 1H), 6.36-6.34 (m, 1H), 6.30 (d, J = 15.6 Hz, 1H), 6.19 (d, J = 3.2 Hz, 1H), 6.08 (dd, J = 16.4, 8.8 Hz, 1H), 2.72-2.65 (m, 1H), 1.14 (d, J = 6.8Hz, 3H); ^{13}C NMR (100 MHz, $CDCl_3$): δ 152.7, 141.6, 129.2 (d, J = 7.0 Hz), 119.3, 111.2, 107.1, 86.4 (dt, J = 169 Hz, 29 Hz), 37.4 (d, J = 19.0 Hz), 15.6 (d, J = 6.0 Hz); ^{19}F NMR (376 MHz, $CDCl_3$): δ -220.68~-220.75 (m, 1F); IR (neat): 1681, 1660, 1460, 1275, 952, 883, 800,734, 665, 594 cm^{-1}; HRMS (EI): Exact mass calcd for $C_9H_9D_2FO$ [M]$^+$: 156.0916, Found: 156.0914.

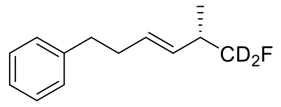

D-4d

Product **D-4d** was obtained in 66% yield from **1r** as a colorless oil. HPLC analysis (Chiralcel OD-H+OD-H, ^{i}PrOH/hexane = 0/100, 1.0 mL/min, 220 nm; t_r (major) = 16.54 min, t_r (minor) = 17.63 min gave the isomeric composition of the product: 94% ee. $[\alpha]_D^{20}$ = -0.9 (*c* = 0.04, $CHCl_3$); ^{1}H NMR (400 MHz, $CDCl_3$): δ 7.29-7.24 (m, 2H), 7.19-7.16 (m, 3H), 5.60-5.52 (m, 1H), 5.36-5.29 (m, 1H), 2.67 (t, *J* = 7.2 Hz, 2H), 2.54-2.43 (m, 1H), 2.35-2.29 (m, 2H), 1.01 (dd, *J* = 5.6, 1.2 Hz, 3H); ^{13}C NMR (100 MHz, $CDCl_3$): δ 141.8, 131.0 (d, *J* = 7.0 Hz), 130.7, 128.5, 128.2, 125.7, 86.8 (dt, *J* = 169, 23 Hz), 37.2 (d, *J* = 18.0 Hz), 35.9, 34.5, 15.9 (d, *J* = 6.0 Hz); ^{19}F NMR (376 MHz, $CDCl_3$): δ -220.59~-220.67 (m, 1F); IR (neat): 1496, 1454, 1062, 952, 906, 731 698 cm^{-1}; HRMS (EI): Exact mass calcd for $C_{13}H_{15}D_2F$ $[M]^+$: 194.1437, Found: 194.1434.

5. Enantioselective Markovnikov hydromonofluoroalkylation of 1,3-dienes 1 with 5

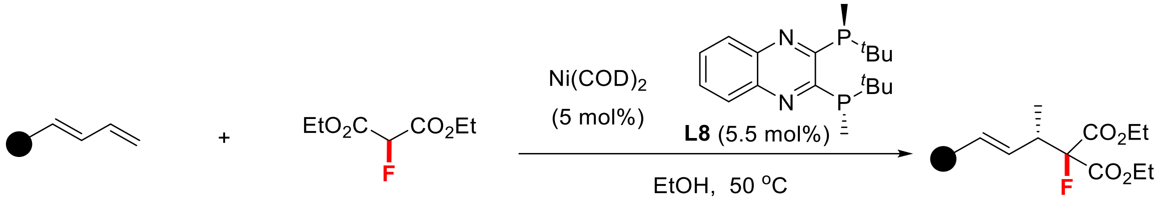

1 **5** **6**

To an oven-dried Schlenk tube equipped with a stirring bar were successively added Ni(COD)$_2$ (3.5 mg, 0.0125 mmol, 5 mol%), (*S,S*)-QuinoxP* **L8** (4.6 mg, 0.01375 mmol, 5.5 mol%), 1,3-dienes **1** (0.375 mmol, 1.5 equivs), and diethyl fluoromalonate **5** (44.5 mg, 0.25 mmol), followed by the addition of absolute EtOH (1.5 mL) in a glove box. After it take out from the glove box, the resulting mixture was stirred at room temperature for 5 min, and then stirred at 50 °C until full conversion. The reaction was monitored by TLC and GC-MS analysis. After full consumption of **5**, the mixture was concentrated under vacuum to give the crude residue, which was purified by silica gel column chromatography (PE/EA = 10:1, v/v) to afford products **6**. Racemic products **6h**, **6x** and **6ab** were prepared using 10 mol% of Ni(COD)$_2$ with a mixed ligand consisting of (*R*,*R*)-quinoxp* and (*S*,*S*)-quinoxp* as the catalyst, and all other racemates **6** were prepared using 10 mol% Ni(COD)$_2$ and 11 mol% dppb.

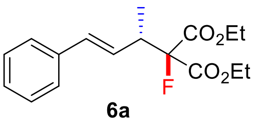

6a

Product **6a** was obtained in 97% yield as colorless oil (3 days). HPLC analysis (Chiralcel AD-H, ^{i}PrOH/hexane = 2/98, 1.0 mL/min, 254 nm; t_r(minor) = 10.97 min, t_r(major) = 12.50 min) gave the isomeric composition of the product: 96% ee; $[\alpha]_D^{20}$ = -84.7 (*c* = 0.51, $CHCl_3$); ^{1}H NMR (400 MHz, $CDCl_3$): δ 7.29-7.17 (m, 5H), 6.46 (d, *J* = 16.0 Hz, 1H), 6.07 (dd, *J* = 15.6, 9.2 Hz, 1H), 4.27 (q, *J* = 7.2 Hz, 2H), 4.20–4.15 (m, 2H), 3.42–3.29 (m, 1H), 1.28 (t, *J* = 7.2 Hz, 3H), 1.19-1.15 (m, 6H); ^{13}C NMR (100 MHz, $CDCl_3$): δ 165.56 (d, *J* = 25.3 Hz), 165.55 (d, *J* = 25.7 Hz), 136.67, 133.09, 128.51, 127.67, 126.85 (d, *J* = 2.7 Hz), 126.36, 97.08 (d, *J* = 203.9 Hz), 62.65, 62.48, 42.73 (d, *J* = 20.4 Hz), 14.70 (d, *J* = 4.4 Hz), 14.06, 14.03; ^{19}F NMR (376 MHz, $CDCl_3$): δ -178.21 (s, 1F). IR (ATR): 1747, 1369, 1232, 1097, 968, 858, 746, 694 cm^{-1}; HRMS (ESI): Exact mass calcd for $C_{17}H_{21}FNaO_4$ $[M+Na]^+$: 331.1316, Found: 331.1309.

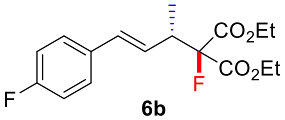

6b

Product **6b** was obtained in 82% yield as colorless oil (3 days). HPLC analysis (Chiralcel AD-H, ^{i}PrOH/hexane = 2/98, 1.0 mL/min, 254 nm; t_r(minor) = 14.32 min, t_r(major) = 18.78 min) gave the isomeric composition of the product: 97% ee; $[\alpha]_D^{20}$ = -28.4 (*c* = 0.50, $CHCl_3$); ^{1}H NMR (400 MHz, $CDCl_3$): δ 7.31–7.28 (m, 2H), 7.00–6.96 (m,

2H) 6.47 (d, J = 15.6 Hz, 1H), 6.04 (dd, J = 16.4, 9.2 Hz, 1H), 4.32 (q, J = 7.2 Hz, 2H), 4.22 (q, J = 7.2 Hz, 2H), 3.44–3.30 (m, 1H), 1.33 (t, J = 7.2 Hz, 3H), 1.24-1.20 (m, 6H); ^{13}C NMR (100 MHz, $CDCl_3$): δ 165.54 (d, J = 25.5 Hz, 2C), 162.37 (d, J = 245.5 Hz), 132.82 (d, J = 3.3 Hz), 131.87, 127.85 (d, J = 7.9 Hz), 126.64 (t, J = 2.4 Hz), 97.03 (d, J = 204.1 Hz), 62.66, 62.47, 42.65 (d, J = 20.3 Hz), 14.66 (d, J = 4.3 Hz), 14.06, 14.02; ^{19}F NMR (376 MHz, $CDCl_3$): δ -178.21 (s, 1F), -114.33 (s, 1F); IR (ATR): 1747, 1369, 1226, 1159, 1097, 970, 858, 821 cm^{-1}; HRMS (ESI): Exact mass calcd for $C_{17}H_{20}F_2NaO_4$ $[M+Na]^+$: 349.1222, Found: 349.1219

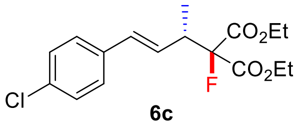

6c

Product **6c** was obtained in 83% yield as colorless oil (3 days). HPLC analysis (Chiralcel AD-H, ^{i}PrOH/hexane = 2/98, 1.0 mL/min, 254 nm; t_r (minor) = 12.05 min, t_r (major) = 15.76 min) gave the isomeric composition of the product: 95% ee; $[\alpha]_D^{20}$ = -40.0 (c = 0.52, $CHCl_3$); ^{1}H NMR (400 MHz, $CDCl_3$): δ 7.26 (s, 4H), 6.45 (d, J = 16.0 Hz, 1H), 6.10 (dd, J = 16.0, 9.2 Hz, 1H), 4.32 (q, J = 7.2 Hz, 2H), 4.22 (q, J = 7.2 Hz, 2H), 3.45–3.32 (m, 1H), 1.33 (t, J = 7.2 Hz, 3H), 1.23-1.20 (m, 6H); ^{13}C NMR: (100 MHz, $CDCl_3$): δ 165.51 (d, J = 25.6 Hz), 165.49 (d, J = 25.3 Hz), 135.14, 133.35, 131.87, 128.69, 127.60 (d, J = 1.8 Hz), 127.56, 96.96 (d, J = 204.2 Hz), 62.71, 62.51, 42.66 (d, J = 20.3 Hz), 14.61 (d, J = 4.3 Hz), 14.07, 14.03; ^{19}F NMR (376 MHz, $CDCl_3$): δ -178.19 (s, 1F); IR (ATR): 1747, 1369, 1232, 1163, 1093, 1039, 970, 810 cm^{-1}; HRMS (ESI): Exact mass calcd for $C_{17}H_{20}ClFNaO_4$ $[M+Na]^+$: 365.0926, Found: 365.0920.

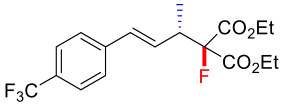

6d

Product **6d** was obtained in 96% yield as colorless oil (4 days). HPLC analysis (Chiralcel AD-H, ^{i}PrOH/hexane = 2/98, 1.0 mL/min, 254 nm; t_r(minor) = 10.68 min, t_r(major) = 13.89 min) gave the isomeric composition of the product: 93% ee; $[\alpha]_D^{20}$ = -47.4 (c = 0.71, $CHCl_3$); ^{1}H NMR (400 MHz, $CDCl_3$): δ 7.55 (d, J = 8.0 Hz, 2H), 7.43 (d, J = 8.0 Hz, 2H), 6.54 (d, J = 15.6 Hz, 1H), 6.23 (dd, J = 16.0, 9.2 Hz, 1H), 4.33 (q, J = 7.2 Hz, 2H), 4.23 (q, J = 7.2 Hz, 2H), 3.49–3.34 (m, 1H), 1.33 (t, J = 7.2 Hz, 3H), 1.24-1.20 (m, 6H); ^{13}C NMR (100 MHz, $CDCl_3$): δ 165.40 (d, J = 25.8 Hz), 165.35 (d, J = 25.4 Hz), 140.08, 131.73, 129.72 (d, J = 2.6 Hz), 129.45 (q, J = 32.1 Hz), 126.48, 125.44 (q, J = 3.5 Hz), 124.07 (q, J = 270.1 Hz), 96.78 (d, J = 204.2 Hz), 62.69, 62.50, 42.55 (d, J = 20.5 Hz), 14.47 (d, J = 3.7 Hz), 13.99, 13.94; ^{19}F NMR (376 MHz, $CDCl_3$): δ -177.95 (s, 1F), -62.55 (s, 3F); IR (ATR): 1749, 1616, 1323, 1234, 1163, 1066, 824, 737 cm^{-1}; HRMS (ESI): Exact mass calcd for $C_{18}H_{20}F_4NaO_4$ $[M+Na]^+$: 399.1190, Found: 399.1181.

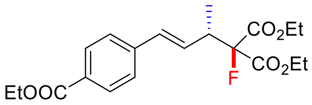

6e

Product **6e** was obtained in 99% yield as colorless oil (3 days). HPLC analysis (Chiralcel OJ-H, ^{i}PrOH/hexane = 15/85, 0.5 mL/min, 230 nm; t_r(minor) = 24.88 min, t_r(major) = 25.40 min) gave the isomeric composition of the product: 99% ee; $[\alpha]_D^{20}$ = -65.45 (c = 0.91, $CHCl_3$); ^{1}H NMR (400 MHz, $CDCl_3$): δ 7.96 (d, J = 8.4 Hz, 2H), 7.38 (d, J = 8.4 Hz, 2H), 6.54 (d, J = 16.0 Hz, 1H), 6.24 (dd, J = 16.0, 9.2 Hz, 1H), 4.39-4.30 (m, 4H), 4.22 (q, J = 6.8 Hz, 2H), 3.48–3.34 (m, 1H), 1.39 (t, J = 7.2 Hz, 3H), 1.33 (t, J = 7.2 Hz, 3H) 1.23-1.19 (m, 6H); ^{13}C NMR (100 MHz, $CDCl_3$): δ 166.32, 165.48 (d, J = 25.6 Hz), 165.44 (d, J = 25.2 Hz), 140.96, 132.27, 129.85, 129.60 (d, J = 2.6 Hz), 129.54, 126.19, 96.87 (d, J = 204.4 Hz), 62.73, 62.54, 60.91, 42.70 (d, J = 20.4 Hz), 14.54 (d, J = 4.3 Hz), 14.30, 14.05, 14.02; ^{19}F NMR (376 MHz, $CDCl_3$): δ -177.93 (s, 1F); IR (ATR): 1751, 1716, 1608, 1367, 1276, 1234, 1178, 765 cm^{-1}; HRMS (ESI): Exact mass calcd for $C_{20}H_{26}FO_6$ $[M+H]^+$: 381.1708, Found: 381.1703.

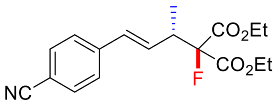

6f

Product **6f** was obtained in 98% yield as colorless oil (4 days). HPLC analysis (Chiralcel OJ-H, ^{i}PrOH/hexane = 5/95, 1.0 mL/min, 230 nm; t_r(minor) = 37.15 min, t_r(major) = 42.76 min) gave the isomeric composition of the product: 98% ee; $[\alpha]_D^{20}$ = -69.5 (c = 0.91, $CHCl_3$); ^{1}H NMR (400 MHz, $CDCl_3$): δ 7.57 (d, J = 8.4 Hz, 2H), 7.40 (d, J = 8.4 Hz, 2H), 6.52 (d, J = 15.6 Hz, 1H), 6.26 (dd, J = 15.6, 8.8 Hz, 1H), 4.32 (q, J = 7.2 Hz, 2H), 4.25-4.20 (m, 2H), 3.46–3.36 (m, 1H), 1.32 (t, J = 7.2 Hz, 3H), 1.23-1.19 (m, 6H); ^{13}C NMR (100 MHz, $CDCl_3$): δ 165.37 (d, J = 25.6 Hz), 165.29 (d, J = 25.3 Hz), 141.07, 132.38, 131.49, 131.12 (d, J = 2.8 Hz), 126.83, 118.78, 111.01, 96.66 (d, J = 204.5 Hz), 62.77, 62.57, 42.50 (d, J = 20.4 Hz), 14.45 (d, J = 4.3 Hz), 14.04, 14.00; ^{19}F NMR (376 MHz, $CDCl_3$): δ -177.60 (s, 1F); IR (ATR): 2225, 1749, 1604, 1369, 1097, 1041, 858, 819 cm^{-1}; HRMS (ESI): Exact mass calcd for $C_{18}H_{21}FNO_4$ $[M+ H]^+$: 334.1449, Found: 334.1450.

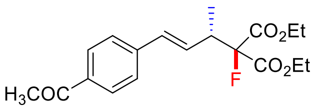

6g

The reaction was carried out using 10 mol% of chiral Ni catalyst. Product **6g** was obtained in 98% yield as colorless oil (4 days). HPLC analysis (Chiralcel AD-H, ^{i}PrOH/hexane = 5/95, 1.0 mL/min, 230 nm; t_r(minor) = 21.54 min, t_r(major) = 19.75 min) gave the isomeric composition of the product: 99% ee; $[\alpha]_D^{20}$ = -72.41 (c = 0.93, $CHCl_3$); ^{1}H NMR (400 MHz, $CDCl_3$): δ 7.88 (d, J = 8.4 Hz, 2H), 7.40 (d, J = 8.4 Hz, 2H), 6.54 (d, J = 15.6 Hz, 1H), 6.26 (dd, J = 16.0, 9.2 Hz, 1H), 4.32 (q, J = 7.2 Hz, 2H), 4.25-4.20 (m, 2H), 3.48–3.36 (m, 1H), 2.58 (s, 3H), 1.33 (t, J = 7.8 Hz, 3H), 1.23-1.20 (m, 6H); ^{13}C NMR (100 MHz, $CDCl_3$): δ 197.45, 165.46 (d, J = 25.5 Hz), 165.41 (d, J = 25.4 Hz), 141.26, 136.21, 132.13, 130.00 (d,

J = 2.7 Hz), 128.72, 126.43, 96.83 (d, J = 204.4 Hz), 62.73, 62.54, 42.68 (d, J = 20.4 Hz), 26.53, 14.53 (d, J = 4.3 Hz), 14.05, 14.01; ^{19}F NMR (376 MHz, $CDCl_3$): δ -177.75 (s, 1F); IR (ATR): 2981, 1749, 1602, 1411, 1359, 1269, 1041, 972 cm^{-1}; HRMS (ESI): Exact mass calcd for $C_{19}H_{24}FO_5$ $[M+H]^+$: 351.1602, Found: 351.1598.

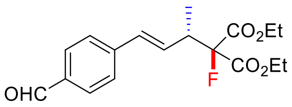

6h

Product **6h** was obtained in 77% yield as colorless oil (4 days). HPLC analysis (Chiralcel OJ-H, ^{i}PrOH/hexane = 25/75, 1.0 mL/min, 230 nm; t_r(minor) = 11.95 min, t_r(major) = 15.43 min) gave the isomeric composition of the product: 97% ee; $[\alpha]_D^{20}$ = -102.11 (c = 0.75, $CHCl_3$); ^{1}H NMR (400 MHz, $CDCl_3$): δ 9.97 (s, 1H), 7.81 (d, J = 8.4 Hz, 2H), 7.48 (d, J = 8.0 Hz, 2H), 6.57 (d, J = 16.0 Hz, 1H), 6.31 (dd, J = 15.6, 8.8 Hz, 1H), 4.33 (q, J = 7.2 Hz, 2H), 4.26-4.21 (m, 2H), 3.48–3.37 (m, 1H), 1.33 (t, J = 7.2 Hz, 3H), 1.24-1.20 (m, 6H); ^{13}C NMR (100 MHz, $CDCl_3$): δ 191.59, 165.44 (d, J = 25.6 Hz), 165.38 (d, J = 25.4 Hz), 142.64, 135.56, 132.07, 130.81 (d, J = 2.7 Hz), 130.09, 126.86, 96.78 (d, J = 204.4 Hz), 62.77, 62.57, 42.66 (d, J = 20.5 Hz), 14.51 (d, J = 4.2 Hz), 14.06, 14.02; ^{19}F NMR (376 MHz, $CDCl_3$): δ -177.71 (s, 1F); IR (ATR): 1749, 1697, 1602, 1271, 1166, 1041, 858, 738 cm^{-1}; HRMS (ESI): Exact mass calcd for $C_{18}H_{22}FO_5$ $[M+H]^+$: 337.1446, Found: 337.1444.

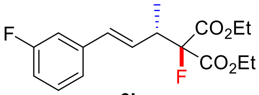

6i

The reaction was carried out using 10 mol% of chiral Ni catalyst. Product **6i** was obtained in 97% yield as colorless oil (4 days). HPLC analysis (Chiralcel AD-H, ^{i}PrOH/hexane = 2/98, 1.0 mL/min, 254 nm; t_r(minor) = 10.41 min, t_r(major) = 15.91 min) gave the isomeric composition of the product: 97% ee; $[\alpha]_D^{20}$ = -101.9 (c = 0.71, $CHCl_3$); ^{1}H NMR (400 MHz, $CDCl_3$): δ 7.28–7.22 (m, 1H), 7.09 (d, J = 8.0 Hz, 1H), 7.03 (d, J = 10.0 Hz, 1H), 6.92 (t, J = 7.6 Hz, 1H), 6.47 (d, J = 15.6 Hz, 1H), 6.13 (dd, J = 16.0 , 9.2 Hz, 1H), 4.32 (q, J = 6.8 Hz, 2H), 4.23 (q, J = 6.8 Hz, 2H), 3.44-3.33 (m, 1H), 1.33 (t, J = 6.8 Hz, 3H), 1.24-1.20 (m, 6H); ^{13}C NMR (100 MHz, $CDCl_3$): δ 165.47 (d, J = 25.6 Hz, 2C), 163.04 (d, J = 243.9 Hz), 138.99 (d, J = 7.6 Hz), 132.00 (d, J = 2.6 Hz), 129.97 (d, J = 8.4 Hz), 128.37 (d, J = 2.8 Hz), 122.21 (d, J = 2.8 Hz), 114.48 (d, J = 21.2 Hz), 112.82 (d, J = 21.7 Hz), 96.90 (d, J = 204.2 Hz), 62.70, 62.52, 42.56 (d, J = 20.3 Hz), 14.57 (d, J = 4.2 Hz), 14.05, 14.01; ^{19}F NMR (376 MHz, $CDCl_3$): δ -178.08 (s, 1F), -113.52 (s, 1F); IR (ATR): 1747, 1583, 1446, 1230, 1097, 941, 779, 686 cm^{-1}; HRMS (ESI): Exact mass calcd for $C_{17}H_{20}F_2NaO_4$ $[M+Na]^+$: 349.1222, Found: 349.1214.

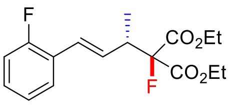
6j

Product **6j** was obtained in 83% yield as colorless oil (4 days). HPLC analysis (Chiralcel AD-H, ^{i}PrOH/hexane = 2/98, 1.0 mL/min, 230 nm; t_r (minor) = 11.63 min, t_r (major) = 12.27 min) gave the isomeric composition of the product: 95% ee; $[\alpha]_D^{20}$ = -66.2 (c = 0.49, $CHCl_3$); ^{1}H NMR (400 MHz, $CDCl_3$): δ 7.42 (t, J = 8.0 Hz, 1H), 7.22-7.17 (m, 1H), 7.09-6.98 (m, 2H), 6.69 (d, J = 16.4 Hz, 1H), 6.19 (dd, J = 16.0, 9.2 Hz, 1H), 4.33 (q, J = 7.2 Hz, 2H), 4.24 (q, J = 7.2 Hz, 2H), 3.47-3.36 (m, 1H), 1.33 (t, J = 6.4 Hz, 3H), 1.26-1.21 (m, 6H); ^{13}C NMR (100 MHz, $CDCl_3$): δ 165.49 (d, J = 25.3 Hz), 165.45 (d, J = 26.0 Hz), 160.07 (d, J = 247.6 Hz), 129.47 (dd, J = 25.3, 3.0 Hz), 128.99 (d, J = 8.4 Hz), 127.23 (d, J = 3.6 Hz), 125.44 (d, J = 3.8 Hz), 124.41 (d, J = 12.3 Hz), 124.06 (d, J = 3.6 Hz), 115.59 (d, J = 21.9 Hz), 96.95 (d, J = 203.5 Hz), 62.67, 62.54, 43.00 (d, J = 20.4 Hz), 14.57 (d, J = 4.0 Hz), 14.00, 13.97; ^{19}F NMR (376 MHz, $CDCl_3$): δ -178.11 (s, 1F), -118.54 (s, 1F); IR (ATR): 1749, 1487, 1456, 1271, 1230, 1041, 972, 756 cm^{-1}; HRMS (ESI): Exact mass calcd for $C_{17}H_{20}F_2NaO_4$ $[M+Na]^+$: 349.1222, Found: 349.1213.

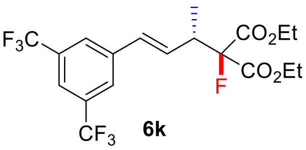
6k

Product **6k** was obtained in 73% yield as colorless oil (4 days). HPLC analysis (Chiralcel AD-H, ^{i}PrOH/hexane = 5/95, 1.0 mL/min, 230 nm; t_r(minor) = 3.97 min, t_r(major) = 7.34 min) gave the isomeric composition of the product: 97% ee; $[\alpha]_D^{20}$ = -30.66 (c = 0.99, $CHCl_3$); ^{1}H NMR (400 MHz, $CDCl_3$): δ 7.74 (s, 3H), 6.58 (d, J = 16.0 Hz, 1H), 6.31 (dd, J = 15.6, 8.8 Hz, 1H), 4.34 (q, J = 7.2 Hz, 2H), 4.25 (q, J = 6.8 Hz, 2H), 3.49–3.39 (m, 1H), 1.34 (t, J = 7.2 Hz, 3H), 1.26-1.22 (m, 6H); ^{13}C NMR (100 MHz, $CDCl_3$): δ 165.40 (d, J = 25.5 Hz), 165.28 (d, J = 25.3 Hz), 138.72, 131.68 (q, J = 33.1 Hz), 131.40 (d, J = 2.8 Hz), 130.43, 126.15, 123.23 (q, J = 271.1 Hz), 121.18-121.14 (m), 96.68 (d, J = 204.7 Hz), 62.85, 62.67, 42.41 (d, J = 20.5 Hz), 14.48 (d, J = 4.2 Hz), 14.03; ^{19}F NMR (376 MHz, $CDCl_3$): δ -177.44 (s, 1F), -63.04 (s, 6F); IR (ATR): 1753, 1381, 1278, 1178, 1134, 1043, 970, 682 cm^{-1}; HRMS (ESI): Exact mass calcd for $C_{19}H_{20}F_7O_4$ $[M+H]^+$: 445.1244, Found: 445.1241.

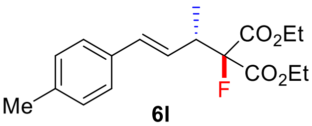
6l

Product **6l** was obtained in 86% yield as colorless oil (3 days). HPLC analysis (Chiralcel AD-H, ^{i}PrOH/hexane = 2/98, 1.0 mL/min, 254 nm; t_r (minor) = 9.67 min, t_r (major) = 11.08 min) gave the isomeric composition of the product: 97% ee; $[\alpha]_D^{20}$ = -121.1 (c = 0.84, $CHCl_3$); ^{1}H NMR (400 MHz, $CDCl_3$): δ 7.22 (d, J = 7.6 Hz, 2H), 7.09 (d, J = 7.6 Hz, 2H), 6.47 (d, J = 16.0 Hz, 1H), 6.06 (dd, J = 16.0, 9.2 Hz, 1H), 4.32 (q, J = 7.2 Hz, 2H), 4.21 (q, J = 6.8 Hz, 2H), 3.44-3.30 (m, 1H), 2.32 (s, 3H), 1.33 (t, J = 8.8 Hz, 3H), 1.23-1.19 (m, 6H); ^{13}C NMR (100 MHz, $CDCl_3$): δ 165.57 (d, J = 25.4 Hz), 165.52 (d, J = 25.7 Hz), 137.43,

133.86, 132.91, 129.15,126.22, 125.73 (d, J = 2.6 Hz), 97.09 (d, J = 203.8 Hz), 62.55, 62.37, 42.71 (d, J = 20.3 Hz), 21.07, 14.67 (d, J = 4.3 Hz), 14.01, 13.96; ^{19}F NMR (376 MHz, $CDCl_3$): δ -178.28 (s, 1F); IR (ATR): 1747, 1446, 1232, 1271, 1232, 1039, 970, 804 cm^{-1}; HRMS (ESI): Exact mass calcd for $C_{18}H_{23}FNaO_4$ $[M+Na]^+$: 345.1473, Found: 345.1475.

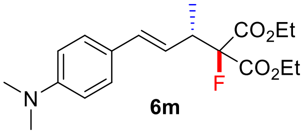

The reaction was carried out at 70 ºC using 10 mol% of chiral Ni catalyst. Product **6m** was obtained in 68% yield as colorless oil (4 days). HPLC analysis (Chiralcel OJ-H, ^{i}PrOH/hexane = 5/95, 1.0 mL/min, 230 nm; t_r (minor) = 58.72 min, t_r (major) = 44.60 min) gave the isomeric composition of the product: 96% ee; $[\alpha]_D^{20}$ = -22.49 (c = 0.61, $CHCl_3$); ^{1}H NMR (400 MHz, $CDCl_3$): δ 7.22 (d, J = 8.8 Hz, 2H), 6.65 (d, J = 8.4 Hz, 2H), 6.41 (d, J = 15.6 Hz, 1H), 5.90 (dd, J = 15.6, 9.2 Hz, 1H), 4.31 (q, J = 7.2 Hz, 2H), 4.24-4.19 (m, 2H), 3.39-3.31 (m, 1H), 2.94 (s, 6H), 1.33 (t, J = 7.2 Hz, 3H), 1.24-1.18 (m, 6H); ^{13}C NMR (100 MHz, $CDCl_3$): δ 165.78 (d, J = 25.5 Hz), 165.66 (d, J = 25.8 Hz), 150.14, 132.94, 127.32, 125.21, 122.27 (d, J = 2.5 Hz), 112.31, 97.39 (d, J = 203.5 Hz), 62.54, 62.38, 42.96 (d, J = 20.3 Hz), 40.46, 14.93 (d, J = 4.4 Hz), 14.09, 14.03; ^{19}F NMR (376 MHz, $CDCl_3$): δ -178.54 (s, 1F); IR (ATR): 2980, 1749, 1608, 1521, 1446, 1355, 1039 cm^{-1}; HRMS (ESI): Exact mass calcd for $C_{19}H_{27}FNO_4$ $[M+H]^+$: 352.1919, Found: 352.1911.

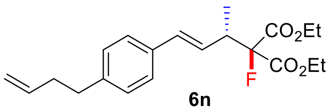

The reaction was carried out using 10 mol% of chiral Ni catalyst. Product **6n** was obtained in 99% yield as colorless oil (4 days). HPLC analysis (Chiralcel IF, ^{i}PrOH/hexane = 5/95, 0.5 mL/min, 230 nm; t_r(minor) = 13.14 min, t_r(major) = 14.44 min) gave the isomeric composition of the product: 96% ee; $[\alpha]_D^{20}$ = -60.07 (c = 0.84, $CHCl_3$); ^{1}H NMR (400 MHz, $CDCl_3$): δ 7.25 (d, J = 6.4 Hz, 2H), 7.11 (d, J = 8.4 Hz, 2H), 6.47 (d, J = 16.0 Hz, 1H), 6.07 (q, J = 6.4 Hz, 1H), 5.87-5.80 (m, 1H), 5.05-4.96 (m, 2H), 4.32 (q, J = 7.2 Hz, 2H), 4.25-4.19 (m, 2H), 3.43-3.32 (m, 1H), 2.68 (t, J = 7.2 Hz, 2H), 2.38-2.32 (m, 2H), 1.33 (t, J = 7.6 Hz, 3H), 1.24-1.19 (m, 6H); ^{13}C NMR (100 MHz, $CDCl_3$): δ 165.62 (d, J = 25.4 Hz), 165.56 (d, J = 25.7 Hz), 141.50, 137.92, 134.37, 132.92, 128.59, 126.32, 126.01 (d, J = 2.7 Hz), 114.96, 97.12 (d, J = 203.8 Hz), 62.60, 62.43, 42.73 (d, J = 20.3 Hz), 35.34, 35.04, 14.73 (d, J = 4.3 Hz), 14.05, 14.01; ^{19}F NMR (376 MHz, $CDCl_3$): δ -178.22 (s, 1F); IR (ATR): 2980, 1749, 1514, 1369, 1271, 1041, 970, 860 cm^{-1}; HRMS (ESI): Exact mass calcd for $C_{21}H_{28}FO_4$ $[M+H]^+$: 363.1966, Found: 363.1963.

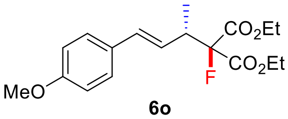
6o

Product **6o** was obtained in 95% yield as colorless oil (50-70 °C, 6 days). HPLC analysis (Chiralcel OX-H, ^{i}PrOH/hexane = 1/99, 1.0 mL/min, 254 nm; t_r(minor) = 22.39 min, t_r(major) = 20.38 min) gave the isomeric composition of the product: 97% ee; $[\alpha]_D^{20}$ = -77.9 (*c* = 0.50, $CHCl_3$); ^1H NMR (400 MHz, $CDCl_3$): δ 7.27 (d, *J* = 7.2 Hz, 2H), 6.84-6.82 (m, 2H), 6.44 (d, *J* = 16.0 Hz, 1H), 5.97 (dd, *J* = 16.0, 9.2 Hz, 1H), 4.32 (q, *J* = 6.8 Hz, 2H), 4.22 (q, *J* = 7.2 Hz, 2H), 3.80 (s, 3H), 3.43-3.29 (m, 1H), 1.33 (t, *J* = 6.8 Hz, 3H), 1.24-1.19 (m, 6H); ^{13}C NMR (100 MHz, $CDCl_3$): δ 166.15 (d, *J* = 25.5 Hz), 165.60 (d, *J* = 26.0 Hz), 159.27, 132.48, 129.47, 127.53, 124.56 (d, *J* = 2.7 Hz), 113.92, 97.21 (d, *J* = 203.9 Hz), 62.60, 62.42, 55.25, 42.79 (d, *J* = 20.3 Hz), 14.78 (d, *J* = 4.3 Hz), 14.07, 14.02; ^{19}F NMR (376 MHz, $CDCl_3$): δ -178.47 (s, 1F); IR (ATR): 1747, 1512, 1246, 1174, 1032, 968, 856, 820 cm^{-1}; HRMS (ESI): Exact mass calcd for $C_{18}H_{23}FNaO_5$ $[M+Na]^+$: 361.1422, Found: 361.1419.

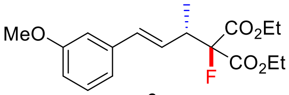
6p

Product **6p** was obtained in 72% yield as colorless oil (3 days). HPLC analysis (Chiralcel OJ-H, ^{i}PrOH/hexane = 2/98, 1.0 mL/min, 254 nm; t_r(minor) = 54.85 min, t_r(major) = 39.30 min) gave the isomeric composition of the product: 97% ee; $[\alpha]_D^{20}$ = -66.2 (*c* = 0.49, $CHCl_3$); ^1H NMR (400 MHz, $CDCl_3$): δ 7.23-7.19 (m, 1H), 6.93 (d, *J* = 7.6 Hz, 1H), 6.86 (s, 1H), 6.78 (d, *J* = 7.6 Hz, 1H), 6.48 (d, *J* = 16.0 Hz, 1H), 6.11 (dd, *J* = 15.6, 9.2 Hz, 1H), 4.33 (q, *J* = 7.2 Hz, 2H), 4.23 (q, *J* = 7.2 Hz, 2H), 3.81 (s, 3H), 3.46-3.31 (m, 1H), 1.33 (t, *J* = 7.2 Hz, 3H), 1.25-1.20 (m, 6H); ^{13}C NMR (100 MHz, $CDCl_3$): δ 165.57 (d, *J* = 25.4 Hz), 165.53 (d, *J* = 25.7 Hz), 159.78, 138.12, 133.02, 129.48, 127.16 (d, *J* = 2.7 Hz), 119.06, 113.36, 111.64, 97.01 (d, *J* = 204.1 Hz), 62.65, 62.48, 55.21, 42.43 (d, *J* = 20.3 Hz), 14.68 (d, *J* = 4.3 Hz), 14.06, 14.02; ^{19}F NMR (376 MHz, $CDCl_3$): δ -178.24 (s, 1F); IR (ATR): 1747, 1580, 1230, 1157, 1039, 775, 734, 690 cm^{-1}; HRMS (ESI): Exact mass calcd for $C_{18}H_{23}FNaO_5$ $[M+Na]^+$: 361.1422, Found: 361.1419.

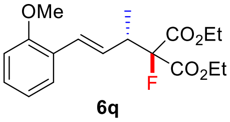
6q

Product **6q** was obtained in 80% yield as colorless oil (3 days). HPLC analysis (Chiralcel OX-H, ^{i}PrOH/hexane = 2/98, 1.0 mL/min, 254 nm; t_r(minor) = 13.1 min, t_r(major) = 12.1 min) gave the isomeric composition of the product: 99% ee; $[\alpha]_D^{20}$ = -15.3 (*c* = 0.3, $CHCl_3$); ^1H NMR (400 MHz, $CDCl_3$): δ 7.39 (d, *J* = 7.6 Hz, 1H), 7.23-7.19 (m, 1H), 6.91-6.83 (m, 3H), 6.08 (dd, *J* = 16.0, 8.8 Hz, 1H), 4.32 (q, *J* = 7.2 Hz, 2H), 4.23 (q, *J* = 11.2 Hz, 2H), 3.82 (s, 3H), 3.46-3.35 (m, 1H), 1.33 (t, *J* = 6.8 Hz, 3H), 1.26-1.20 (m, 6H); ^{13}C NMR (100 MHz, $CDCl_3$): δ 165.63 (d, *J* = 25.4 Hz), 165.53 (d, *J* = 26.0 Hz), 156.54, 128.67, 127.86, 127.17 (d, *J* = 2.6 Hz), 126.68, 125.74, 120.55, 110.71, 97.19 (d, *J* = 203.1 Hz), 62.52, 62.40, 55.30, 43.10 (d, *J* = 20.3

Hz), 14.68 (d, J = 4.4 Hz), 13.97, 13.91; ^{19}F NMR (376 MHz, $CDCl_3$): δ -178.33 (s, 1F); IR (ATR): 1749, 1290, 1271, 1244, 1103, 1097, 1029, 752 cm^{-1}; HRMS (ESI): Exact mass calcd for $C_{18}H_{23}FNaO_5$ $[M+Na]^+$: 361.1422, Found: 361.1414.

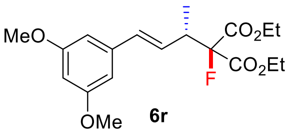

6r

Product **6r** was obtained in 96% yield as colorless oil (4 days). HPLC analysis (Chiralcel AD-H, ^{i}PrOH/hexane = 2/98, 1.0 mL/min, 254 nm; t_r(minor) = 17.24 min, t_r(major) = 35.06 min) gave the isomeric composition of the product: 97% ee; $[\alpha]_D^{20}$ = -55.8 (c = 0.50, $CHCl_3$); ^{1}H NMR (400 MHz, $CDCl_3$): δ 6.48 (s, 2H), 6.43 (d, J = 15.6 Hz, 1H), 6.36 (s, 1H), 6.09 (dd, J = 15.6, 9.2 Hz, 1H), 4.32 (q, J = 6.8 Hz, 2H), 4.22 (q, J =7.2 Hz, 2H), 3.79 (s, 6H), 3.45-3.30 (m, 1H), 1.33 (t, J = 6.8 Hz, 3H), 1.25-1.20 (m, 6H); ^{13}C NMR (100 MHz, $CDCl_3$): δ 165.55 (d, J = 25.3 Hz), 165.51 (d, J = 25.8 Hz), 160.87, 138.67, 133.13, 127.31 (d, J = 2.6 Hz), 104.45, 99.99, 97.05 (d, J = 204.0 Hz), 62.67, 62.51, 55.33, 42.68 (d, J = 20.4 Hz), 14.68 (d, J = 4.3 Hz), 14.08, 14.02; ^{19}F NMR (376 MHz, $CDCl_3$): δ -178.30 (s, 1F); IR (ATR): 1747, 1591, 1265, 1205, 1153, 968, 734, 702 cm^{-1}; HRMS (ESI): Exact mass calcd for $C_{19}H_{25}FNaO_6$ $[M+Na]^+$: 391.1527, Found: 391.1534.

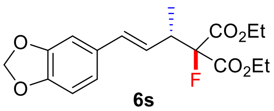

6s

The reaction was carried out using 10 mol% of chiral Ni catalyst. Product **6s** was obtained in 81% yield as colorless oil (6 days). HPLC analysis (Chiralcel AD-H, ^{i}PrOH/hexane = 2/98, 1.0 mL/min, 254 nm; t_r(minor) = 20.39 min, t_r(major) = 24.24 min) gave the isomeric composition of the product: 95% ee; $[\alpha]_D^{20}$ = -64.0 (c = 0.52, $CHCl_3$); ^{1}H NMR (400 MHz, $CDCl_3$): δ 6.87 (s, 1H), 6.76-6.70 (m, 2H), 6.40 (d, J = 15.6 Hz, 1H), 5.96-5.90 (m, 3H), 4.31 (q, J = 6.8 Hz, 2H), 4.21 (q, J = 8.0 Hz, 2H), 3.39-3.28 (m, 1H), 1.31 (t, J = 6.8 Hz, 3H), 1.23-1.17 (m, 6H); ^{13}C NMR (100 MHz, $CDCl_3$): δ 165.56 (d, J = 25.2 Hz), 165.52 (d, J = 25.7 Hz), 147.95, 147.24, 132.62, 131.07, 124.92 (d, J = 2.6 Hz), 120.98, 108.16, 105.61, 101.03, 97.11 (d, J = 204.1 Hz), 62.63, 62.45, 42.68 (d, J = 20.3 Hz), 14.71 (d, J = 4.4 Hz), 14.07, 14.00; ^{19}F NMR (376 MHz, $CDCl_3$): δ -178.58 (s, 1F); IR (ATR): 1745, 1489, 1444, 1247, 1093, 927, 858, 792 cm^{-1}; HRMS (ESI): Exact mass calcd for $C_{18}H_{21}FNaO_6$ $[M+Na]^+$: 375.1214, Found: 375.1216.

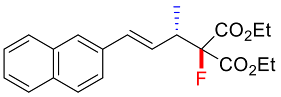

6t

Product **6t** was obtained in 81% yield as a white solid (3 days), m.p. = 58-60 °C; HPLC analysis (Chiralcel AD-H, ^{i}PrOH/hexane = 2/98, 1.0 mL/min, 230 nm; t_r (minor) = 14.34 min, t_r (major) = 17.98 min) gave the isomeric composition of the product: 97% ee; $[\alpha]_D^{20}$ = -66.7 (c = 0.26, $CHCl_3$); ^{1}H NMR (400 MHz, $CDCl_3$): δ

7.80-7.76 (m, 3H), 7.69 (s, 1H), 7.55 (d, J = 8.4 Hz, 1H), 7.48–7.41 (m, 2H), 6.67 (d, J = 16.0 Hz, 1H), 6.26 (dd, J = 15.6, 6.8 Hz, 1H), 4.34 (q, J = 6.8 Hz, 2H), 4.28 (q, J = 6.8 Hz, 2H) , 3.53–3.38 (m, 1H), 1.34 (t, J = 7.2 Hz, 3H), 1.26–1.20 (m, 6H); ^{13}C NMR (100 MHz, CDCl$_3$): δ 165.56 (d, J = 25.4 Hz, 2C), 134.09, 133.48, 133.17, 132.99, 128.14, 127.90, 127.60, 127.21 (d, J = 2.6 Hz), 126.23 (d, J = 1.4 Hz), 125.86, 123.48, 97.09 (d, J = 204.1 Hz), 62.63, 62.46, 42.85 (d, J = 20.4 Hz), 14.71 (d, J = 4.4 Hz), 14.03, 13.99; ^{19}F NMR (376 MHz, CDCl$_3$): δ -178.20 (s, 1F). IR (ATR): 1747, 1367, 1265, 1230, 1095, 966, 813, 734 cm^{-1}; HRMS (ESI): Exact mass calcd for $C_{21}H_{23}FNaO_4$ [M+Na]$^+$: 381.1473, Found: 381.1474.

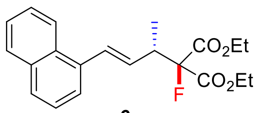

6u

Product **6u** was obtained in 98% yield as colorless oil (3 days). HPLC analysis (Chiralcel AD-H, ^{i}PrOH/hexane = 2/98, 1.0 mL/min, 230 nm; t$_r$(minor) = 11.51 min, t$_r$(major) = 13.05 min) gave the isomeric composition of the product: 93% ee; $[\alpha]_D^{20}$ = -26.3 (c = 0.71, CHCl$_3$); ^{1}H NMR (400 MHz, CDCl$_3$): δ 8.06 (d, J = 8.0 Hz, 1H), 7.85-7.83 (m, 1H), 7.77 (d, J = 8.4 Hz, 1H), 7.55-7.41 (m, 4H), 7.28 (d, J = 16.4 Hz, 1H), 6.16 (dd, J = 15.6, 9.2 Hz, 1H), 4.35 (q, J = 10.8 Hz, 2H), 4.27-4.21 (m, 2H), 3.61-3.48 (m, 1H), 1.36 (t, J = 7.2 Hz, 3H), 1.29 (d, J = 6.8 Hz, 3H), 1.23 (t, J = 7.2 Hz, 3H); ^{13}C NMR (100 MHz, CDCl$_3$): δ 165.69 (d, J = 25.7 Hz), 165.62 (d, J = 25.5 Hz), 134.46, 133.53, 131.06, 130.54, 130.23 (d, J = 2.6 Hz), 128.51, 128.03, 126.02, 125.74, 125.59, 124.04, 123.65, 97.19 (d, J = 204.2 Hz), 62.70, 62.57, 43.01 (d, J = 20.3 Hz), 14.72 (d, J = 4.3 Hz), 14.05 (s, 2C); ^{19}F NMR (376 MHz, CDCl$_3$): δ -178.35 (s, 1F); IR (ATR): 1745, 1456, 1367, 1228, 1099, 968, 858, 771 cm^{-1}; HRMS (ESI): Exact mass calcd for $C_{21}H_{23}FNaO_4$ [M+Na]$^+$: 381.1473, Found: 381.1479.

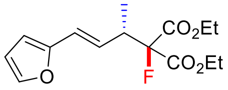

6v

The reaction was carried out using 10 mol% of chiral Ni catalyst. Product **6v** was obtained in 93% yield as colorless oil (5 days). HPLC analysis (Chiralcel AD-H, ^{i}PrOH/hexane = 2/98, 1.0 mL/min, 254 nm; t$_r$ (minor) = 11.37 min, t$_r$ (major) = 13.60 min) gave the isomeric composition of the product: 94% ee; $[\alpha]_D^{20}$ = -73.06 (c = 0.99, CHCl$_3$); ^{1}H NMR (400 MHz, CDCl$_3$): δ 7.32 (s, 1H), 6.34-6.29 (m, 2H), 6.20 (s, 1H), 6.06 (dd, J = 16.0, 9.2 Hz, 1H), 4.32 (q, J = 7.2 Hz, 2H), 4.24 (q, J = 6.8 Hz, 2H), 3.40-3.29 (m, 1H), 1.32 (t, J = 6.8 Hz, 3H), 1.26-1.18 (m, 6H); ^{13}C NMR (100 MHz, CDCl$_3$): δ 165.54 (d, J = 25.5 Hz), 165.49 (d, J = 25.6 Hz),152.13, 141.98, 125.43 (d, J = 2.8 Hz), 121.38, 111.14, 107.87, 96.90 (d, J = 204.4 Hz), 62.64, 62.49, 42.29 (d, J = 20.4 Hz), 14.51 (d, J = 4.4 Hz), 13.99, 13.96; ^{19}F NMR (376 MHz, CDCl$_3$): δ -178.14 (s, 1F); IR (ATR): 1747, 1232, 1095, 1037, 962, 929, 738. 596 cm^{-1}; HRMS (ESI): Exact mass calcd for $C_9H_{12}F$

[M+H]: 299.1217, Found: 299.1286.

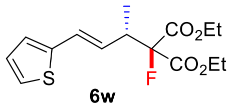

6w

The reaction was carried out using 10 mol% of chiral Ni catalyst. Product **6w** was obtained in 98% yield as yellowish liquid (4 days); HPLC analysis (Chiralcel AD-H, ^{i}PrOH/hexane = 2/98, 1.0 mL/min, 230 nm; t_r(minor) =13.51 min, t_r (major) = 16.71 min) gave the isomeric composition of the product: 98% ee; $[\alpha]_D^{20}$ = -5.23 (c = 0.15, $CHCl_3$); ^{1}H NMR (400 MHz, $CDCl_3$): δ 7.14 (d, J = 5.2 Hz, 1H), 6.95-6.92 (m, 2H), 6.62 (d, J = 15.6 Hz, 1H), 5.95 (dd, J = 16.0, 9.2 Hz, 1H), 4.32 (q, J = 7.2 Hz, 2H), 4.24 (q, J = 7.2 Hz, 2H), 3.42–3.27 (m, 1H), 1.32 (t, J = 7.2 Hz, 3H), 1.26–1.19 (m, 6H); ^{13}C NMR (100 MHz, $CDCl_3$): δ 165.53 (d, J = 25.3 Hz, 2C), 141.67, 127.25, 126.38 (d, J = 2.6 Hz), 126.22, 125.69, 124.38, 96.95 (d, J = 204.4 Hz), 62.68, 62.53, 42.56 (d, J = 20.6 Hz), 14.58 (d, J = 4.4 Hz), 14.05, 14.02; ^{19}F NMR (376 MHz, $CDCl_3$): δ -178.11 (s, 1F); IR (ATR): 1749, 1463, 1269, 1165, 1095, 958, 856, 698 cm^{-1}; HRMS (ESI): Exact mass calcd for $C_{15}H_{19}FNaO_4S$ $[M+Na]^+$: 337.0880, Found: 337.0877.

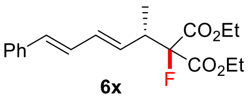

6x

The reaction was carried out by using 10 mol% of chiral Ni catalyst. Product **6x** was obtained in 98% yield as colorless oil (5 days). HPLC analysis (Chiralcel AD-H, ^{i}PrOH/hexane =1 /99, 1.0 mL/min, 254 nm; t_r (minor) = 30.74 min, t_r (major) = 36.71 min) gave the isomeric composition of the product: 91% ee; $[\alpha]_D^{20}$ = -57.1 (c = 0.50, $CHCl_3$); ^{1}H NMR (400 MHz, $CDCl_3$): δ 7.38-7.29 (m, 4H), 7.24-7.20 (m, 1H), 6.71 (dd, J = 16.0, 10.8 Hz, 1H), 6.50 (d, J = 3.9 Hz, 1H) , 6.31 (dd, J = 15.2, 10.9 Hz, 1H), 5.71 (dd, J = 15.2, 9.2 Hz, 1H), 4.32 (q, J = 7.2 Hz, 2H), 4.29-4.23 (m, 2H), 3.38-3.27 (m, 1H), 1.33 (t, J = 7.2 Hz, 3H), 1.27 (t, J = 7.2 Hz, 3H), 1.17 (d, J = 6.8 Hz, 3H); ^{13}C NMR (100 MHz, $CDCl_3$): δ 165.56 (d, J = 25.5 Hz), 165.53 (d, J = 25.7 Hz), 137.05, 133.54, 132.54, 130.83 (d, J = 2.8 Hz), 128.59, 128.27, 127.59, 126.32, 96.99 (d, J = 203.9 Hz), 62.65, 62.51, 42.45 (d, J = 20.5 Hz), 14.58 (d, J = 4.3 Hz), 14.11, 14.03; ^{19}F NMR (376 MHz, $CDCl_3$): δ -178.43 (s, 1F); IR (ATR): 1747, 1265, 1236, 1161, 989, 858, 734, 692 cm^{-1}; HRMS (ESI): Exact mass calcd for $C_{19}H_{23}FNaO_4$ $[M+Na]^+$: 357.1473, Found: 357.1475.

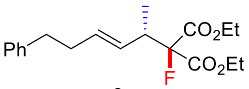

6y

The reaction was carried out at 60 °C using 10 mol% of chiral Ni catalyst. Product **6y** was obtained in 94% yield as colorless oil (6 days), The ^{19}F NMR analysis of the crude mixture revealed that the regioselective ratio was 7:1. HPLC analysis (Chiralcel AD-H, ^{i}PrOH/hexane = 1/99, 1.0 mL/min, 210 nm; t_r (minor) = 12.90 min, t_r (major) = 13.93 min) gave the isomeric composition of the product: 97% ee; $[\alpha]_D^{20}$ = -34.9 (c = 0.90, $CHCl_3$); ^{1}H NMR

(400 MHz, $CDCl_3$): δ 7.29-7.26 (m, 2H), 7.19-7.14 (m, 3H), 5.67-5.59 (m, 1H), 5.41 (dd, J = 15.6 Hz, 8.8 Hz, 1H), 4.30 (q, J = 7.2 Hz, 2H), 4.21 (q, J = 7.2 Hz, 2H), 3.24-3.10 (m, 1H), 2.64 (t, J = 8.0 Hz, 2H), 2.33-2.27 (m, 2H), 1.33-1.25 (m, 6H), 1.08 (d, J = 6.8 Hz, 3H); ^{13}C NMR (100 MHz, $CDCl_3$): 165.66 (d, J = 25.4 Hz), 165.58 (d, J = 26.0 Hz), 141.53, 133.36, 128.32, 128.24, 127.75 (d, J = 2.6 Hz), 125.78, 97.17 (d, J = 203.2 Hz), 62.47, 62.27, 42.18 (d, J = 20.4 Hz), 35.61, 34.12, 14.64 (d, J = 4.3 Hz), 14.02, 13.97; ^{19}F NMR (376 MHz, $CDCl_3$): δ -178.92 (s, 1F); IR (ATR): 1747, 1454, 1234, 1163, 1028, 970, 798, 698 cm^{-1}; HRMS (ESI): Exact mass calcd for $C_{19}H_{25}FNaO_4$ $[M+Na]^+$: 359.1629, Found: 359.1625.

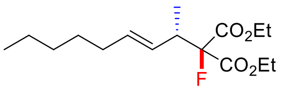

6z

The reaction was carried out at 80 °C using 10 mol% of chiral Ni catalyst. Product **6z** was obtained in 96% yield as colorless oil (5 days). The ^{19}F NMR analysis of the crude mixture revealed that the regioselective ratio was 7:1. The isomeric composition of **6s** was determined to be 95% ee by HPLC analysis of its derivative **6s'**. $[\alpha]_D^{20}$ = -32.7 (c = 0.95, $CHCl_3$); ^1H NMR (400 MHz, $CDCl_3$): 5.61-5.54 (m, 1 H), 5.34 (dd, J = 15.2, 8.8 Hz, 1H), 4.32-4.21 (m, 4 H), 3.24-3.09 (m, 1H), 1.98-1.93 (m, 2H), 1.33-1.24 (m, 12H), 1.08 (d, J = 6.8 Hz, 3H), 0.87 (t, J = 7.2 Hz, 3H); ^{13}C NMR (100 MHz, $CDCl_3$): δ 165.77 (d, J = 25.5 Hz), 165.67 (d, J = 25.9 Hz), 134.55, 126.93 (d, J = 2.6 Hz), 97.4 (d, J = 203.2 Hz), 62.49, 62.28, 42.35 (d, J = 20.2 Hz), 32.39, 31.25, 28.87, 22.46, 14.77 (d, J = 4.3 Hz), 14.05, 14.01, 14.00; ^{19}F NMR (376 MHz, $CDCl_3$): δ -179.19 (s, 1F); IR (ATR): 1751, 1267, 1240, 1168, 1041, 974, 738, 704 cm^{-1}; HRMS (ESI): Exact mass calcd for $C_{16}H_{27}FNaO_4$ $[M+Na]^+$: 325.1786, Found: 325.1778.

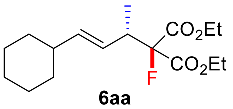

6aa

The reaction was carried out at 75 °C using 10 mol% of chiral Ni catalyst. Product **6aa** was obtained in 97% yield as colorless oil (5 days). The ^{19}F NMR analysis of the crude mixture revealed that the regioselective ratio was 10:1. The isomeric composition of **6t** was determined to be 90% ee by HPLC analysis of its derivative **6t'**; $[\alpha]_D^{20}$ = -25.5 (c = 0.49, $CHCl_3$); ^1H NMR (400 MHz, $CDCl_3$): δ 5.51 (dd, J = 14.4, 6.8 Hz, 1H), 5.29 (dd, J = 15.6, 9.2 Hz, 1H), 4.29 (q, J = 7.2 Hz , 2H), 4.22 (q, J = 7.2 Hz , 2H), 3.20-3.05 (m, 1H), 1.90-1.87 (m, 1H), 1.70-1.61 (m, 5H), 1.33-1.25 (m, 9H), 1.10-1.02 (m, 5H); ^{13}C NMR (100 MHz, $CDCl_3$): δ 165.77 (d, J = 25.5 Hz), 165.63 (d, J = 26.3 Hz), 140.27, 124.60 (d, J = 2.7 Hz), 97.45 (d, J = 202.7 Hz), 62.48, 62.27, 42.45 (d, J = 20.2 Hz), 40.56, 32.85, 32.77, 26.06, 25.90, 25.88, 14.79 (d, J = 4.2 Hz), 14.07, 14.01; ^{19}F NMR (376 MHz, $CDCl_3$): δ -179.36 (s, 1F); IR (ATR): 2924, 2380, 2349, 1747, 1448, 1230, 1031, 970 cm^{-1}; HRMS (ESI): Exact mass calcd for $C_{17}H_{27}FNaO_4$ $[M+Na]^+$: 337.1786, Found:

337.1778.

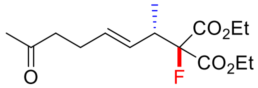
6ab

The reaction was carried out at 70 °C using 10 mol% of chiral Ni catalyst. Product **6ab** was obtained in 79% yield as colorless oil (4 days). The ^{19}F NMR analysis of the crude mixture revealed that the regioselective ratio was 22:1. HPLC analysis (Chiralcel IF, ^{i}PrOH/hexane = 10/90, 1.0 mL/min, 230 nm; t_r (minor) = 7.87 min, t_r (major) = 8.15 min) gave the isomeric composition of the product: 99% ee; $[\alpha]_D^{20}$ = -12.47 (*c* = 0.51, $CHCl_3$); ^1H NMR (400 MHz, $CDCl_3$): δ 5.61-5.54 (m, 1H), 5.38 (dd, *J* = 15.6, 6.8 Hz, 1H), 4.31-4.20 (m, 4H), 3.22-3.08 (m, 1H), 2.46 (t, *J* = 7.2 Hz, 2H), 2.24 (q, *J* = 7.6 Hz, 2H), 2.12 (s, 3H), 1.32-1.26 (m, 6H), 1.07 (d, *J* = 7.2 Hz, 3H); ^{13}C NMR (100 MHz, $CDCl_3$): δ 207.82, 165.61 (d, *J* = 26.1 Hz), 165.55 (d, *J* = 25.4 Hz), 132.40, 128.14 (d, *J* = 2.7 Hz), 97.10 (d, *J* = 203.0 Hz), 62.53, 62.33, 42.94, 42.06 (d, *J* = 20.4 Hz), 29.87, 26.46, 14.59 (d, *J* = 4.3 Hz), 14.05, 13.99; ^{19}F NMR (376 MHz, $CDCl_3$): δ -178.71 (s, 1F); IR (ATR): 2981, 1747, 1714, 1446, 1367, 1161, 1039, 858 cm^{-1}; HRMS (ESI): Exact mass calcd for $C_{15}H_{24}FO_5$ $[M+H]^+$: 303.1602, Found: 303.1600.

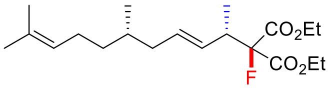
6ac

The reaction was carried out at 80 °C using 10 mol% of chiral Ni catalyst. Product **6ac** was obtained in 96% yield as colorless oil (3 days). ^{19}F NMR analysis of the crude reaction mixture revealed that dr value was 11:1; $[\alpha]_D^{20}$ = -13.6 (*c* = 0.51, $CHCl_3$); ^1H NMR (400 MHz, $CDCl_3$): 5.60-5.53 (m,1H), 5.34 (dd, *J* = 15.2, 8.8 Hz, 1H), 5.09-5.06 (m, 1H), 4.29 (q, *J* = 7.2 Hz , 2H), 4.23 (q, *J* = 7.2 Hz , 2H), 3.24-3.13 (m, 1H), 2.02-1.90 (m, 3H), 1.85-1.78 (m, 1H), 1.67 (s, 3H), 1.59 (s, 3H), 1.46-1.42 (m, 1H), 1.33-1.26 (m, 8H), 1.8 (d, *J* = 6.8 Hz, 3H), 0.83 (d, *J* = 6.4 Hz, 3H); ^{13}C NMR (100 MHz, $CDCl_3$): δ 165.77 (d, *J* = 25.4 Hz), 165.68 (d, *J* = 25.9 Hz), 132.94, 131.12, 128.23 (d, *J* = 2.7 Hz), 124.72, 97.27 (d, *J* = 203.4 Hz), 62.25, 62.30, 42.45 (d, *J* = 1.0 Hz), 39.75, 36.57, 32.53, 25.68. 25.55, 19.18, 17.59, 14.85 (d, *J* = 4.3 Hz), 14.03, 14.01; ^{19}F NMR (376 MHz, $CDCl_3$): δ -179.07 (s, 1F); IR (ATR): 1749, 1456, 1367, 1267, 1230, 1041, 972, 860 cm^{-1}; HRMS (ESI): Exact mass calcd for $C_{20}H_{33}FNaO_4$ $[M+Na]^+$: 379.2255, Found: 379.2249.

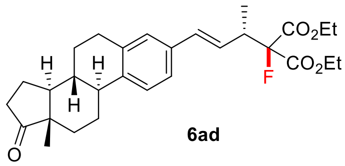
6ad

The reaction was carried out at 80 °C using 10 mol% of chiral Ni catalyst. Product **6ad** was obtained in 82% yield as colorless oil (4 days). ^{19}F NMR analysis of the crude reaction mixture revealed that dr value was 9:1; $[\alpha]_D^{20}$ = +67.0 (*c* = 0.50, $CHCl_3$); ^1H NMR (400 MHz, $CDCl_3$): δ 7.22 (d, *J* = 8.0

Hz, 1H), 7.12-7.08 (m, 2H), 6.45 (d, J = 16.0 Hz, 1H), 6.07 (dd, J = 15.6, 9.2 Hz, 1H), 4.32 (q, J = 7.2 Hz, 2H), 4.26-4.18 (m, 2H), 3.43-3.32 (m,1H), 2.91-2.88 (m, 2H), 2.54-2.47 (m, 1H), 2.42-2.39 (m, 1H), 2.30-2.25 (m, 1H), 2.19-1.94 (m, 5H), 1.63-1.47 (m, 5H), 1.33 (t, J = 7.2 Hz, 3H), 1.25-1.18 (m, 6H), 0.91 (s, 3H); ^{13}C NMR (100 MHz, $CDCl_3$): δ 220.80, 165.60 (d, J = 25.4 Hz), 165.52 (d, J = 25.8 Hz), 139.43, 136.59, 134.29, 132.78, 126.82, 126.20 (d, J = 2.7 Hz), 125.50, 123.93, 97.10 (d, J = 203.7 Hz), 62.63, 62.44, 50.46, 47.94, 44.40, 42.73 (d, J = 20.2 Hz), 38.11, 35.82, 31.55, 29.31, 26.44, 25.68, 21.55, 14.77 (d, J = 4.3 Hz), 14.10, 14.03, 13.81; ^{19}F NMR (376 MHz, $CDCl_3$): δ -178.53 (s, 1F); IR (ATR): 1737, 1454, 1267, 1234, 1039, 819, 734, 665cm^{-1}; HRMS (ESI): Exact mass calcd for $C_{29}H_{37}FNaO_5$ $[M+Na]^+$: 507.2517, Found: 507.2524.

General procedure for synthesizing the derivatives 6z' and 6aa' of product 6z and 6aa

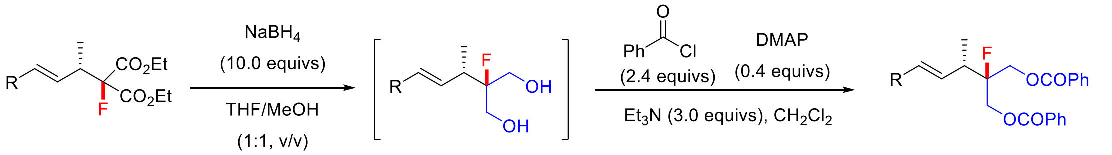

6z: R = ^{n}C$_5$H$_{11}$
6aa: R = ^{c}Hexyl

6z': R = ^{n}C$_5$H$_{11}$, 46%, 95% ee
6aa': R = ^{c}Hexyl, 42%, 90% ee

To a stirred solution of product **6z** or **6aa** (0.1 mmol) in a mixed solvent of anhydrous THF/MeOH (1.0 mL, 1:1, v/v) was added $NaBH_4$ slowly at 0 °C, the resulting mixture was then stirred at rt for overnight. The reaction mixture was quenched with H_2O (5 mL) and extracted with CH_2Cl_2 (10 mL × 2). The combined organic layers were dried over Na_2SO_4, the reaction mixture was concentrated under reduced pressure to give the residue, which was filtered over a short pad of silica gel eluting with PE/EtOAc (1/1, v/v) to afford the crude diols, which was used directly for next step.

To a solution of the above diol in CH_2Cl_2 was added DMAP (0.4 equiv), Et_3N (3.0 equivs) and benzoyl chloride (2.4 equivs) at 0 °C. The reaction mixture was warmed to rt and stirred overnight. After quenching with H_2O (5 mL), the resulting mixture was extracted with CH_2Cl_2 (10 mL × 2). The combined organic layers were dried over Na_2SO_4, and concentrated under reduced pressure. The obtained residue was purified by silica gel column chromatography using PE/EtOAc (20/1, v/v) as the elute to deliver the product **6z'** or **6aa'** as colorless oil.

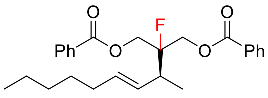

6z'

Product **6z'** was obtained in 46% yield from **6z** via the sequential $NaBH_4$ reduction and benzoyl protection. HPLC analysis (Chiralpak IF, ^{i}PrOH/hexane = 1/99, 0.5 mL/min, 230 nm; t_r (minor) = 22.54 min, t_r (major) = 24.25 min) gave the isomeric composition of the product: 95% ee; $[\alpha]_D^{20}$ = -108.3 (c = 0.48, $CHCl_3$); ^{1}H NMR (400

MHz, $CDCl_3$): δ 8.04 (d, J = 8.4 Hz, 4H), 7.59-7.56 (m, 2H), 7.46-7.42 (m, 4H), 5.62-5.55 (m, 1H), 5.45 (dd, J = 15.6, 8.4 Hz, 1H), 4.68-4.51 (m, 4H), 2.92-2.80 (m, 1H), 2.02-1.97 (m, 2H), 1.32-1.21 (m, 9H), 0.86 (t, J = 6.8 Hz, 3H); ^{13}C NMR (100 MHz, $CDCl_3$): δ 165.92 (s, 2C), 134.00, 133.27, 129.69, 129.53 (d, J = 3.5 Hz), 128.47, 128.24 (d, J = 6.1 Hz), 96.01 (d, J = 179.8 Hz), 63.94 (d, J = 27.5 Hz), 63.78 (d, J = 27.9 Hz), 40.40 (d, J = 21.1 Hz), 32.51, 31.35, 28.86, 22.45, 14.48 (d, J = 4.7 Hz), 14.01; ^{19}F NMR (376 MHz, $CDCl_3$): δ -172.54 (s, 1F); IR (ATR): 1724, 1452, 1265, 1176, 1070, 1028, 978, 707 cm^{-1}; HRMS (ESI): Exact mass calcd for $C_{26}H_{31}FNaO_4$ $[M+Na]^+$: 449.2099, Found: 449.2108.

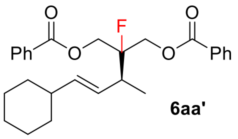

6aa'

Product **6aa'** was obtained in 42% yield from **6aa** via the sequential $NaBH_4$ reduction and benzoyl protection. HPLC analysis (Chiralcel OZ-H, ^{i}PrOH/hexane = 1/99, 1.0 mL/min, 230 nm; t_r (minor) = 12.49 min, t_r (major) = 14.37 min) gave the isomeric composition of the product: 90% ee; $[\alpha]_D^{20}$ = -25.3 (c = 0.35, $CHCl_3$); ^{1}H NMR (400 MHz, $CDCl_3$): δ 8.05-8.03 (m, 4H), 7.59-7.55 (m, 2H), 7.46-7.42 (m, 4H), 5.52 (dd, J = 15.6, 10.2 Hz, 1H), 5.40 (dd, J = 15.6, 8.4 Hz, 1H), 4.67-4.51 (m, 4H), 2.88-2.79 (m, 1H), 1.95-1.87 (m, 1H), 1.69-1.60 (m, 5H), 1.34-1.05 (m, 8H); ^{13}C NMR (100 MHz, $CDCl_3$): δ 165.91 (s, 2C), 139.73, 133.26 (d, J = 1.3 Hz), 129.70, 129.54 (d, J = 4.6 Hz), 128.46, 125.82 (d, J = 6.1 Hz), 96.03 (d, J = 179.8 Hz), 63.97 (d, J = 27.4 Hz), 63.71 (d, J = 28.2 Hz), 63.57, 40.64, 40.44 (d, J = 21.0 Hz), 32.84, 32.79, 26.06, 25.94, 14.47 (d, J = 4.6 Hz); ^{19}F NMR (376 MHz, $CDCl_3$): δ -172.54 (s, 1F); IR (ATR): 1722, 1450, 1176, 1070, 1026, 974, 767, 686 cm^{-1}; HRMS (ESI): Exact mass calcd for $C_{27}H_{31}FNaO_4$ $[M+Na]^+$: 461.2099, Found: 461.2097.

6. Synthetic utility

6.1 Gram-scale synthesis of monofluoroalkylated product 6a

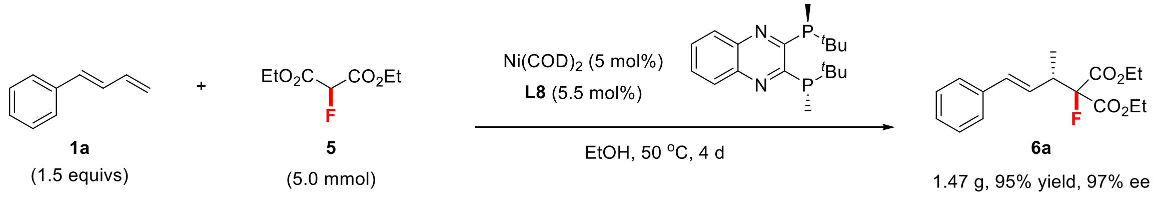

To an oven-dried Schlenk tube equipped with a stirring bar were successively added $Ni(COD)_2$ (69.0 mg, 0.25 mmol, 5 mol%), (S,S)-QuinoxP* **L8** (91.9 mg, 0.275 mmol, 5.5 mol%), 1-phenyl-1,3-butadiene **1** (976.6 mg, 7.5 mmol), and diethyl fluoromalonate **5** (890.8 mg, 5.0 mmol), followed by the addition of absolute EtOH (30 mL) in a glove box. After it take out from the glove box, the resulting

mixture was stirred at room temperature for 5 min, and then stirred at 50 °C until full conversion. The reaction was monitored by TLC and GC-MS analysis. After full consumption of **5**, the reaction mixture was concentrated under vacuum to remove the solvent EtOH. The crude residue was purified by silica gel column chromatography (PE/EA = 10:1, v/v) to afford product **6a** as a colorless oil (1.47 g, 95% yield). HPLC analysis (Chiralcel AD-H, ^{i}PrOH/hexane = 2/98, 1.0 mL/min, 230 nm; t_r(minor) = 11.1 min, t_r(major) = 13.1 min) gave the isomeric composition of the product: 97% ee.

6.2 Transformations of product 6a

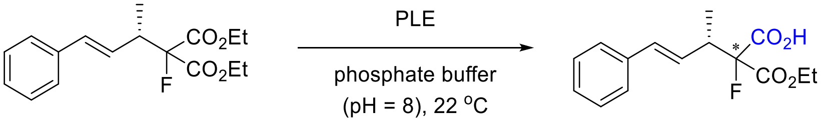

6a (0.2 mmol) **7**

To a solution of **6a** (308.5 mg, 1.0 mmol, 1.0 equiv) in a mixed solvent of phosphate butter (20 mL, pH = 8) and DMSO (6 mL) was added pig liver esterase (PLE) (20 mg, 200 units), the reaction mixture was then stirred at 22 °C. After full conversion of **6a** by TLC analysis, HCl (2 N) was added to make the pH of the solution to 3. The aqueous layers were extracted with CH_2Cl_2 (20 mL × 3). The combined organic phases were washed with brine (20 mL), dried over Na_2SO_4 and concentrated under the reduced pressure to afford the crude product. The ^{1}H and ^{19}F NMR analysis revealed that the dr value was 12:1. The crude residue was purified by flash column chromatography using CH_2Cl_2/MeOH (10:1 to 5:1, v/v) as the eluent to afford the desired product **7** (263.5 mg, 94% yield) as colorless oil; $[\alpha]_D^{20}$ = -60.9 (*c* = 0.51, $CHCl_3$); ^{1}H NMR (400 MHz, $CDCl_3$): δ 7.33-7.21 (m, 5H), 6.52 (d, *J* = 16.0 Hz, 1H), 6.12 (dd, *J* = 16.0, 9.2 Hz, 1H), 4.29 (q, *J* = 7.2 Hz, 2H), 3.42-3.31 (m, 1H), 1.29 (t, *J* = 7.2 Hz, 3H), 1.19 (d, *J* = 6.8 Hz, 3H); ^{13}C NMR (100 MHz, $CDCl_3$): δ 168.67 (d, *J* = 28.7 Hz), 165.84 (d, *J* = 26.0 Hz), 136.62, 133.35, 128.50, 127.70, 126.44, 97.05 (d, *J* = 203.5 Hz), 63.03, 42.73 (d, *J* = 20.6 Hz), 14.63 (d, *J* = 3.9 Hz), 13.94; ^{19}F NMR (376 MHz, $CDCl_3$): δ -176.84 (s, 1F); IR (ATR): 1749, 1296, 1267, 1043, 1014, 1014, 750, 694 cm^{-1}; HRMS (ESI): Exact mass calcd for $C_{15}H_{17}FNaO_4$ $[M+Na]^+$: 303.1003, Found: 303.0989.

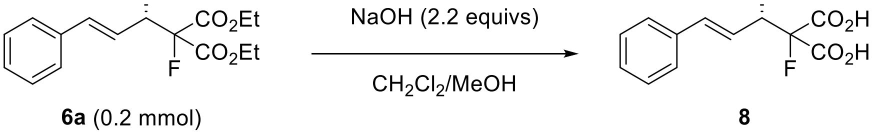

6a (0.2 mmol) **8**

Compound **6a** (61.6 mg, 0.2 mmol, 1.0 equiv) was dissolved in a mixed solvent of MeOH (0.2 mL)

and CH_2Cl_2 (1.2 mL), followed by the addition of NaOH (17.6 mg, 0.44 mmol, 2.2 equivs). The reaction mixture was stirred at rt until full conversion of 3a (ca. 1 h). Then water and HCl (2 N) were added, the resulting mixture was extracted with EtOAc (10 mL × 3). The combined organic phases were successively washed with brine and water (10 mL, each), and then dried over Na_2SO_4, filtrated, and concentrated under vacuum to afford the analytically pure product **8** (49.9 mg, 99% yield) as white solid; m.p. = 151-154 °C; $[\alpha]_D^{20}$ = -3.6 (*c* = 0.52, $CHCl_3$). ^{1}H NMR (400 MHz, MeOD-*d*$_4$): δ 7.35 (d, *J* = 7.6 Hz, 2H), 7.28 (t, *J* = 7.2 Hz, 2H), 7.22-7.18 (m, 1H), 6.56 (d, *J* = 16.0 Hz, 1H), 6.15 (dd, *J* = 15.6, 8.8 Hz, 1H), 3.45-3.34 (m, 1H), 1.21 (d, *J* = 6.8 Hz, 3H); ^{13}C NMR (100 MHz, MeOD-*d*$_4$): δ 167.81 (d, *J* = 26.3 Hz), 167.65 (d, *J* = 25.8 Hz), 136,97, 132.73, 128.17, 127.24, 126.88 (d, *J* = 2.5 Hz), 125.97, 97.13 (d, *J* = 199.2 Hz), 42.13 (d, *J* = 20.4 Hz), 13.69 (d, *J* = 4.2 Hz); ^{19}F NMR (376 MHz, MeOD-*d*$_4$): δ -178.78 (s, 1F); IR (ATR): 1732, 1448, 1159, 1132, 1014, 970, 746, 694 cm^{-1}; HRMS (ESI): Exact mass calcd for $C_{13}H_{13}FNaO_4$ $[M+Na]^+$: 275.0690, Found: 275.0686.

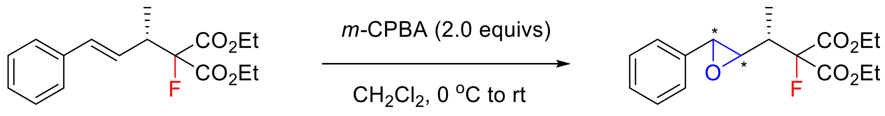

6a (0.2 mmol) **9**

To a stirred solution of **6a** (61.6 mg, 0.2 mmol, 1.0 equiv) in CH_2Cl_2 (2 mL) was added *m*-CPBA (69.0 mg, 0.4 mmol, 2.0 equivs) at 0 °C. After being stirred at rt for 24 h, the reaction mixture was quenched with saturated NH_4Cl (aq., 5 mL) and extracted with CH_2Cl_2 (10 mL × 2). The combined organic layers were dried over Na_2SO_4, and concentrated under reduced pressure to give the residue, which was purified by flash column chromatography (PE/EtOAc, 9/1, v/v) to afford the fluorinated epoxide **9** in 73% yield as white solid (47.3 mg); m.p. = 130-133 °C; ^{1}H NMR analysis of the crude residue revealed that the *dr* value was 1.4:1; HPLC analysis (Chiralcel AD-H, ^{i}PrOH/hexane = 2/98, 1.0 mL/min, 230 nm; major diastereomer: t_r(major) = 18.74 min, t_r(minor) = 16.23 min) gave the isomeric composition of the major diastereomer: 96% ee; $[\alpha]_D^{20}$ = -16.7 (*c* = 0.52, $CHCl_3$); ^{1}H NMR analysis for the mixture diastereomers (400 MHz, $CDCl_3$): δ 7.35-7.21 (m, 5H), 4.37-4.19 (m, 4H), 3.79-3.66 (m, 1H), 3.11-3.00 (m, 1H), 2.66-2.50 (m, 1H), 1.35-1.25 (m, 6H), 1.18-1.14 (m, 3H); ^{13}C NMR for mixture diastereomers (100 MHz, $CDCl_3$): δ 165.51 (d, *J* = 14.6 Hz), 165.48 (d, *J* = 19.3 Hz), 165.24, 164.98, 136.67, 136.58, 128.47, 128.36, 128.34, 125.61, 125.53, 95.65 (d, *J* = 204.6 Hz), 95.47 (d, *J* = 203.1 Hz), 62.93, 62.92, 62.85, 62.80, 61.81 (d, *J* = 3.6 Hz), 61.17 (d, *J* = 3.2 Hz), 59.51, 57.58, 41.94 (d, *J* = 20.6 Hz), 41.38 (d, *J* = 19.7 Hz), 13.98 (d, *J* = 2.0 Hz), 13.73, 11.33 (d, *J* = 4.1 Hz), 10.88 (d, *J* = 3.6 Hz); ^{19}F NMR for mixture diastereomers (376 MHz, $CDCl_3$): δ -176.59 (s), -178.46 (s); IR

(ATR): 2384, 2349, 1751, 1369, 1155, 1043, 752, 698 cm^{-1}; HRMS (ESI): Exact mass calcd for $C_{17}H_{21}OF_5Na$ $[M+Na]^+$: 347.1265, Found: 347.1263.

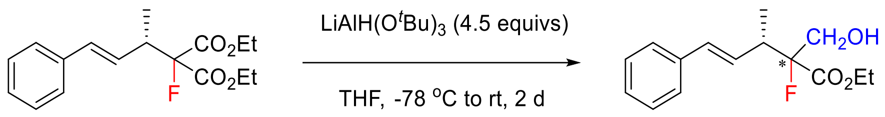

6a (0.2 mmol) **10**

To a stirred solution of **6a** (61.6 mg, 0.2 mmol, 1.0 equiv) in THF (2 mL) was added dropwise $LiAl(OtBu)_3$ (1.0 M in THF, 900 uL) at -78 °C. The resulting mixture was then warmed to rt and stirred at rt for 2 days. After quenching with 10% $KHSO_4$ (aq., 5 mL), the solution was extracted with CH_2Cl_2 (10 mL × 2). The combined organic layers were dried over Na_2SO_4, and concentrated under the reduced pressure. The residue was purified by flash column chromatography (PE/EtOAc, 1/1, v/v) to afford α-allylated α-fluoro-β-hydroxyl ester **10** in 67% yield as light yellow liquid (34.5 mg); ^{19}F NMR analysis of the crude mixture revealed that the *dr* value was 1.4:1; HPLC analysis (Chiralcel AD-H, ^{i}PrOH/hexane = 1/99, 1.0 mL/min, 254 nm; major diastereomer: t_r(major) = 57.76 min, t_r(minor) = 16.37 min; minor diastereomer: t_r (major) = 35.26 min, t_r (minor) = 42.96 min) gave the isomeric composition of major diastereomer: 96% ee, gave the isomeric composition of minor diastereomer: 95% ee; $[\alpha]_D^{20}$ = -31.5 (*c* = 0.60, $CHCl_3$); ^{1}H NMR for the mixture diastereomers (400 MHz, $CDCl_3$): δ 7.35-7.23 (m, 5H), 6.49-6.41 (m, 1H), 6.19-6.09 (m, 1H), 4.35-4.23 (m, 2H), 3.99-3.87 (m, 2H), 2.95-2.82 (m, 1H), 2.14 (br, 1H), 1.34 (t, *J* = 7.2 Hz, 1H), 1.26 (t, *J* = 7.2 Hz, 2H), 1.20-1.15 (m, 3H); ^{13}C NMR for the mixture diastereomers (100 MHz, $CDCl_3$): δ 169.88 (d, *J* = 25.8 Hz), 169.83 (d, *J* = 25.6 Hz), 136.75, 136.62, 132.47, 132.08, 128.58, 128.52, 128.28 (d, *J* = 5.1 Hz), 127.67, 127.63, 127.59, 126.30, 126.28, 99.79 (d, *J* = 190.9 Hz), 99.68 (d, *J* = 191.1 Hz), 66.07 (d, *J* = 23.3 Hz), 65.19 (d, *J* = 23.5 Hz), 61.89, 61.74, 41.70, 41.48, 41.27, 15.29 (d, *J* = 4.5Hz), 14.48 (d, *J* = 3.4 Hz), 14.23; ^{19}F NMR for the mixture diastereomers (376 MHz, $CDCl_3$): δ -183.59 (s), -179.11 (s); IR (ATR): 1747, 1367, 1238, 1095, 1037, 958, 856, 696 cm^{-1}; HRMS (ESI): Exact mass calcd for $C_{15}H_{19}FNaO_3$ $[M+Na]^+$: 289.1210, Found: 289.1219.

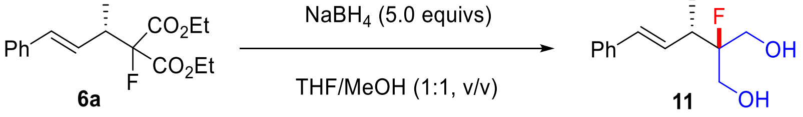

6a **11**

(1.0 mmol, 97% ee) 83%, 97% ee

To a stirred solution of **6a** (308.5 mg, 1.0 mmol, 1.0 equiv) in a mixed solvent of THF (5 mL) and

MeOH (5 mL) was slowly added $NaBH_4$ at 0 °C. The resulting suspension was stirred at rt for overnight. After quenching with H_2O (20 mL), the solution was extracted with CH_2Cl_2 (20 mL × 3). The combined organic layers were successively washed with saturated NH_4Cl (aq., 10 mL) and brine (10 mL), and then dried over Na_2SO_4 and concentrated under reduced pressure. The obtained residue was purified by flash column chromatography using PE/EtOAc (1/1, v/v) as the eluent to afford fluorinated diol **11** (186.6 mg, 83% yield) as a white solid, m.p. = 97-100 °C; HPLC analysis (Chiralcel OJ-H, ^{i}PrOH/hexane = 25/70, 1.0 mL/min, 254 nm; t_r (major) = 7.03 min, t_r (minor) = 7.76 min) gave the isomeric composition of the product: 97% ee; $[\alpha]_D^{20}$ = -13.6 (c = 0.42, $CHCl_3$); ^{1}H NMR (400 MHz, $CDCl_3$): δ 7.37-7.31 (m, 4H), 7.23-7.21 (m, 1H), 6.47 (d, J = 16.0 Hz, 1H), 6.19 (dd, J = 16.0, 8.8 Hz, 1H), 3.96-3.80 (m, 4H), 2.90-2.82 (m, 1H), 1.94 (s, br, 2H), 1.20 (d, J = 6.8 Hz, 3H); ^{13}C NMR (100 MHz, $CDCl_3$): δ 136.92, 131.63, 129.33 (d, J = 5.9 Hz), 128.57, 127.50, 126.21, 98.64 (d, J = 175.5 Hz), 63.65 (d, J = 25.4 Hz, 2C), 40.05 (d, J = 20.9 Hz), 14.33 (d, J = 5.2 Hz); ^{19}F NMR (376 MHz, $CDCl_3$): δ -177.69 (s, 1F); IR (ATR): 3334, 1448, 1265, 1055, 1024, 916, 748, 692 cm^{-1}; HRMS (ESI): Exact mass calcd for$C_{13}H_{17}FNaO_2$ $[M+Na]^+$: 247.1105, Found: 247.1107.

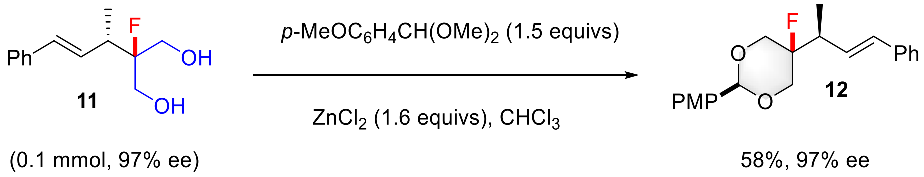

To a stirred solution of **11** (22.4 mg, 0.1 mmol, 1.0 equiv) and 1-(dimethoxymethyl)-4-methoxybezene (27.3 mg, 0.15 mmol, 1.5 equivs) in $CHCl_3$ (1 mL) was added $ZnCl_2$ (21.8 mg, 0.16 mmol, 1.6 equivs). The reaction mixture was allowed to stir at room temperature until full conversion of **11**. The solvent was removed under reduced pressure, and the residue was purified by silica gel column chromatography (PE/EtOAc = 5:1, v/v) to afford **12** (19.8 mg, 58% yield) as a white solid, m.p. = 121-124 °C; HPLC analysis (Chiralcel OX-H, ^{i}PrOH/hexane = 10/90, 1.0 mL/min, 254 nm; t_r(minor) = 29.19 min, t_r(major) = 16.45 min) gave the isomeric composition of the product: 97% ee; $[\alpha]_D^{20}$ = -13.9 (c = 0.49, $CHCl_3$); ^{1}H NMR (400 MHz, $CDCl_3$): δ 7.44 (d, J = 8.8 Hz, 2H), 7.38-7.31 (m, 4H), 7.27-7.23 (m, 1H), 6.88 (d, J = 8.8 Hz, 2H), 6.44 (d, J = 15.6 Hz, 1H), 6.16 (dd, J = 15.6, 9.2 Hz, 1H), 5.40 (s, 1H), 4.35-4.22 (m, 2H), 4.01-3.82 (m, 2H), 3.79 (s, 3H), 2.50-2.40 (m, 1H), 1.19 (d, J = 6.8 Hz, 3H); ^{13}C NMR (100 MHz, $CDCl_3$): δ 160.09, 136.56, 132.19, 130.03, 128.62, 128.27 (d, J = 4.3 Hz), 127.70, 127.46, 126.25, 113.59, 100.76, 89.66 (d, J = 183.7 Hz), 71.56 (d, J = 22.4 Hz), 71.38 (d, J = 22.3 Hz), 55.27, 41.50 (d, J = 20.5Hz), 13.96 (d, J = 4.0 Hz); ^{19}F NMR (376 MHz, $CDCl_3$): δ -177.35

(s, 1F). IR (ATR): 2924, 2852, 1517, 1247, 1035, 977, 738, 696 cm^{-1}; HRMS (ESI): Exact mass calcd for $C_{21}H_{24}FO_3$ $[M+H]^+$: 343.1704, Found: 343.1702.

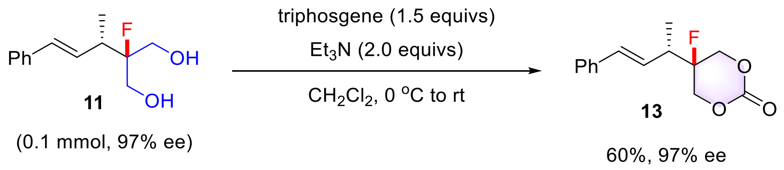

To a stirred solution of **11** (22.4 mg, 0.1 mmol, 1.0 equiv) in CH_2Cl_2 (1 mL) was successively added Et_3N (20.2 mg, 0.2 mmol) and triphosgene (44.5 mg, 0.15 mmol) at -30 °C. The reaction mixture was naturally warmed to rt and stirred overnight. After full consumption of **11** by TLC analysis, the reaction mixture was directly purified by flash column chromatography using PE/EtOAc (2/1, v/v) as the eluent to afford 1,3-dioxan-2-one **13** (15.1 mg, 60% yield) as a white solid; m.p. = 115-118 °C; HPLC analysis (Chiralcel AD-H, ^{i}PrOH/hexane = 30/70, 1.0 mL/min, 254 nm; t_r(minor) = 7.41 min, t_r (major) = 8.38 min) gave the isomeric composition of the product: 97% ee; $[\alpha]_D^{20}$ = -4.3 (*c* = 0.34, $CHCl_3$); ^1H NMR (400 MHz, $CDCl_3$): δ 7.37-7.26 (m, 5H), 6.49 (d, *J* = 15.6 Hz, 1H), 6.08 (dd, *J* = 15.6, 9.2 Hz, 1H), 4.55-4.34 (m, 4H), 2.69-2.60 (m, 1H), 1.24 (d, *J* = 6.8 Hz, 3H); ^{13}C NMR (100 MHz, $CDCl_3$): δ 147.40, 135.82, 133.70, 128.73, 128.24, 126.38, 125.97 (d, *J* = 4.2 Hz), 89.32 (d, *J* = 182.0 Hz), 72.05 (d, *J* = 21.4 Hz), 71.63 (d, *J* = 24.4 Hz), 41.01 (d, *J* = 20.9 Hz), 14.17 (d, *J* = 4.0 Hz); ^{19}F NMR (376 MHz, $CDCl_3$): δ -175.22 (s, 1F); IR (ATR): 1753, 1456, 1184, 1087, 1020, 831, 752, 692 cm^{-1}; HRMS (ESI): Exact mass calcd for $C_{14}H_{15}FNaO_3$ $[M+Na]^+$: 273.0897, Found: 273.0895.

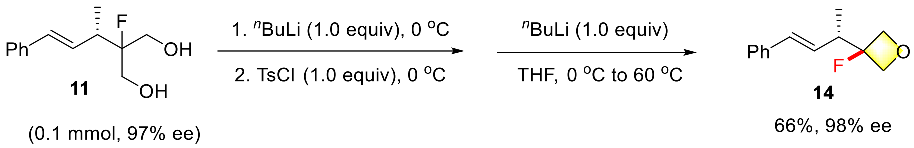

To a solution of **11** (56.1 mg, 0.25 mmol) in THF (1.0 mL) was added ^{n}BuLi (100 μL, 2.5 M in hexanes, 1.0 equiv.) at 0 °C. After being stirred at 0 °C for 30 min, a solution of *p*-toluenesulfonyl chloride (47.7 mg, 0.25 mmol, 1.0 equiv) in the THF (1.0 mL) was added. The resulting mixture was stirred at 0 °C for additional 1 h, and ^{n}BuLi (100 μL, 2.5 M in hexanes, 1.0 equiv) was then added. after being stirred at 60 °C for 6 h, the reaction mixture was cooled and diluted with Et_2O (10 mL) and H_2O (10 mL). The mixture was separated via a separating funnel, and the aqueous layer was extracted with Et_2O (2 x 10 mL). The combined organic layers were dried over Na_2SO_4, filtered and concentrated. The obtained residue was purified by silica gel column chromatography (hexanes/EtOAc = 9:1, v/v) to yield

fluorinated oxetane **14** as colorless oil. HPLC analysis (Chiralcel OD-H, ^{i}PrOH/hexane = 10/90, 1.0 mL/min, 254 nm; t_r(minor) = 6.59 min, t_r(major) = 9.01 min) gave the isomeric composition of the product: 98% ee; $[\alpha]_D^{20}$ = -1.5 (*c* = 0.31, $CHCl_3$); ^1H NMR (400 MHz, $CDCl_3$): 7.38-7.36 (m, 2H), 7.33-7.29 (m, 2H), 7.25-7.22 (m, 1H), 6.53 (d, *J* = 15.6 Hz, 1H), 6.15 (dd, *J* = 15.6, 8.4 Hz, 1H), 4.80-4.69 (m, 2H), 4.64-4.57 (m, 2H), 2.94-2.81 (m, 1H), 1.17 (d, *J* = 6.8 Hz, 3H); ^{13}C NMR (100 MHz, $CDCl_3$): δ 137.46, 133.07, 129.26, 128.46 (d, *J* = 3.8 Hz), 128.29, 126.96, 97.48 (d, *J* = 210.1 Hz), 80.31 (d, *J* = 24.3 Hz), 80.23 (d, *J* = 24.2 Hz), 42.95 (d, *J* = 22.3 Hz), 14.12 (d, *J* = 4.6 Hz); ^{19}F NMR (376 MHz, $CDCl_3$): δ -158.95 (s, 1F); IR (ATR): 2924, 1494, 1450, 1249, 974, 881, 748, 692 cm^{-1}; HRMS (EI): Exact mass calcd for $C_{13}H_{15}FO$ $[M]^+$: 206.1101, Found: 206.1105

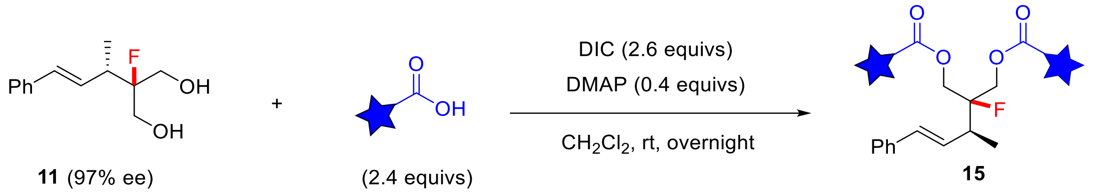

To a solution of **8** (11.2 mg, 0.05 mmol) and carboxylic acid-based drug (0.12 mmol, 2.4 equivs) in CH_2Cl_2 (0.6 mL) were added DMAP (6.1 mg, 0.02 mmol) and DIC (16.0 mg, 0.13 mmol). The resulting suspension was stirred at rt for overnight. After adding H_2O (5 mL), the reaction mixture was extracted with CH_2Cl_2 (10 mL × 3). The combined organic layers were dried over Na_2SO_4, and concentrated under reduced pressure to give the residue, which was purified by flash column chromatography (PE/EtOAc, 4/1, v/v) to afford the corresponding drug derivatives **15**.

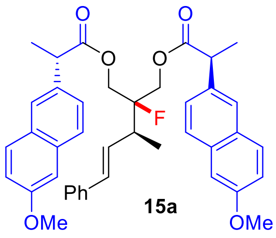

63%, >20:1 dr

Product **15a** was prepared by following the above procedure from *S*-naproxen, and isolated as a foamy solid (20.4 mg, 63% yield), ^{19}F NMR analysis of the crude mixture revealed that the dr value was >20:1; $[\alpha]_D^{20}$ = -33.4 (*c* = 0.43, $CHCl_3$); ^1H NMR (400 MHz, $CDCl_3$): δ 7.72-7.61 (m, 6H), 7.35-7.32 (m, 2H), 7.24-7.09 (m, 9H), 5.96-5.83 (m, 2H), 4.22-4.02 (m, 4H), 3.91 (s, 3H), 3.90 (s, 3H), 3.85-3.78 (m, 2H), 2.29-2.20 (m, 1H), 1.56-1.51 (m, 6H), 0.91 (d, *J* = 6.8 Hz, 3H); ^{13}C NMR (100 MHz, $CDCl_3$): δ 173.69, 173.64, 157.73, 157.66, 136.63, 135.16, 135.08, 133.77, 133.71, 132.04, 129.30, 129.24, 128.87 (d, *J* = 4.0 Hz), 128.43, 127.47, 127.25, 127.16, 126.17, 126.13, 126.06, 126.04, 125.96, 119.13, 119.02, 105.58, 95.79 (d, *J* = 180.0 Hz), 63.54 (d, *J* = 27.4 Hz), 63.39 (d, *J* = 30.4 Hz), 55.26, 45.34, 45.28, 40.00 (d, *J* = 20.5 Hz), 18.05, 17.84, 14.08 (d, *J* = 5.1 Hz); ^{19}F NMR (376 MHz, $CDCl_3$): δ -174.68 (s, 1F); IR (ATR): 1737, 1606, 1392, 1265, 1031, 925, 812,

694; HRMS (ESI): Exact mass calcd for $C_{41}H_{41}FNaO_6$ $[M+Na]^+$: 671.2779, Found: 671.2783.

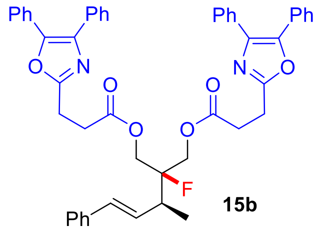

15b

87%, 97% ee

Product **15b** was prepared by following the above procedure from oxaprozin, and isolated as a foamy solid (33.8 mg, 87% yield). HPLC analysis (Chiralcel AD-H, ^{i}PrOH/hexane = 10/90, 1.0 mL/min, 230 nm; t_r (major) = 63.73 min, t_r (minor) = 60.28 min) gave the isomeric composition of the product: 97% ee; $[\alpha]_D^{20}$ = -56.4 (*c* = 0.46, $CHCl_3$); $[\alpha]_D^{20}$ = -36.0 (*c* = 0.51, $CHCl_3$); ^1H NMR (400 MHz, $CDCl_3$): δ 7.63-7.55 (m, 8H), 7.36-7.20 (m, 17H), 6.38 (d, *J* = 16.0 Hz, 1H), 6.11 (dd, *J* = 16.0, 8.8 Hz, 1H), 4.45-4.24 (m, 4H), 3.18-3.13 (m, 4H), 2.97-2.92 (m, 4H), 2.85-2.76 (m, 1H), 1.14 (d, *J* = 7.2 Hz, 3H); ^{13}C NMR (100 MHz, $CDCl_3$): δ 171.24, 161.42, 145.47, 136.66, 135.12, 132.36, 128.91, 128.62, 128.52, 128.46, 128.04, 127.85, 127.58, 126.49, 126.26, 95.66 (d, *J* = 180.1 Hz), 63.41 (d, *J* = 27.5 Hz), 62.96 (d, *J* = 28.8 Hz), 40.14 (d, *J* = 20.9 Hz), 30.85, 23.35, 14.11 (d, *J* = 4.4 Hz); ^{19}F NMR (376 MHz, $CDCl_3$): δ -173.90 (s, 1F); IR (ATR): 2382, 2349, 1743, 1219, 1155, 1026, 763, 692; HRMS (ESI): Exact mass calcd for $C_{49}H_{43}FN_2NaO_6$ $[M+Na]^+$: 797.2997, Found: 797.3004.

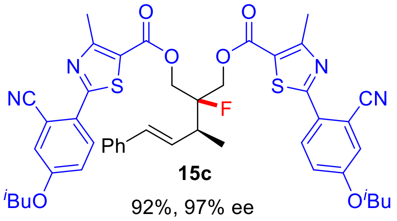

15c

92%, 97% ee

Product **15c** was prepared by following the above procedure from febuxostat, and isolated as a foamy solid (37.8 mg, 92% yield). HPLC analysis (Chiralcel AD-H, ^{i}PrOH/hexane = 10/90, 1.0 mL/min, 254 nm; t_r(minor) = 45.49 min, t_r(major) = 52.32 min) gave the isomeric composition of the product: 97% ee; $[\alpha]_D^{20}$ = -130.3 (*c* = 0.48, $CHCl_3$); ^1H NMR (400 MHz, $CDCl_3$): δ 8.11-8.01 (m, 4H), 7.36-7.34 (m, 2H), 7.31-7.23 (m, 3H), 7.01-6.97 (m, 2H), 6.54 (d, *J* = 16.0 Hz, 1H), 6.23 (dd, *J* = 15.6, 8.4 Hz, 1H), 4.67-4.52 (m, 4H), 3.90 (d, *J* = 6.4 Hz, 4H), 3.04-2.96 (m, 1H), 2.74 (s, 6H), 2.24-2.18 (m, 2H), 1.34 (d, *J* = 7.2 Hz, 3H), 1.10 (d, *J* = 8.4 Hz, 12H); ^{13}C NMR (100 MHz, $CDCl_3$): δ 167.77, 162.61, 162.28, 161.24, 161.12, 161.08, 136.55, 132.60, 132.10, 132.07, 128.58, 127.80, 126.31, 125.68 (d, *J* = 2.7 Hz), 120.53, 120.44, 115.29, 115.27, 112.59, 112.56, 102.96, 95.50 (d, *J* = 180.7 Hz), 75.71, 64.16 (d, *J* = 28.4 Hz), 63.69 (d, *J* = 20.9 Hz), 41.11 (d, *J* = 20.8 Hz), 28.15, 19.03, 17.53, 14.34 (d, *J* = 4.8 Hz); ^{19}F NMR (376 MHz, $CDCl_3$): δ -171.53 (s, 1F); IR (ATR): 1716, 1604, 1508, 1371, 1253, 1085, 1012, 754; HRMS (ESI): Exact mass calcd for $C_{45}H_{45}FN_4NaO_6S_2$ $[M+Na]^+$: 843.2657, Found: 843.2666.

7. Mechanistic studies

To gain some insight into the reaction mechanism, we conducted the following experiments (Scheme S1A-B). First, (*Z*)-1-phenylbutadiene ((*Z*)-**1a**) was subjected to the current hydromonofluoromethylation condition (Scheme S1A). Although the reaction rate for the *Z* isomer was much slower than that of the *E* isomer, only (*E*)-product **3a** was produced in 46% yield with 96% ee, suggesting a π-allylnickel species. The *Z* isomer did not appreciably generate the *E* isomer under the reaction conditions. Second, when EtOD was used as the solvent, the occurrence of H-D scrambling in diene **1a** in the absence of **2a** (eq 1, Scheme S1B), and 45% D (1.34 D) incorporation into the Me group of **3a** under the model reaction (eq 2), revealed that a Ni-H intermediate was generated from the solvent EtOH and that insertion of the terminal double bond of the diene into the Ni-H bond was reversible. Third, the use of deuterated FBSM **D-2a** in this reaction led to the deuterium (0.7 D) incorporation at the C4 positions of product **3a** in nonprotonic solvent THF, suggested that the hydrogen in the FBSM might be a partial source of the proton, though no D incorporation observed in protonic EtOH (eq 3). This result, together with 45% D incorporation at the methyl group using EtOD as the solvent (eq 2), demonstrated that the proton in the product mainly comes from both EtOH and FBSM.

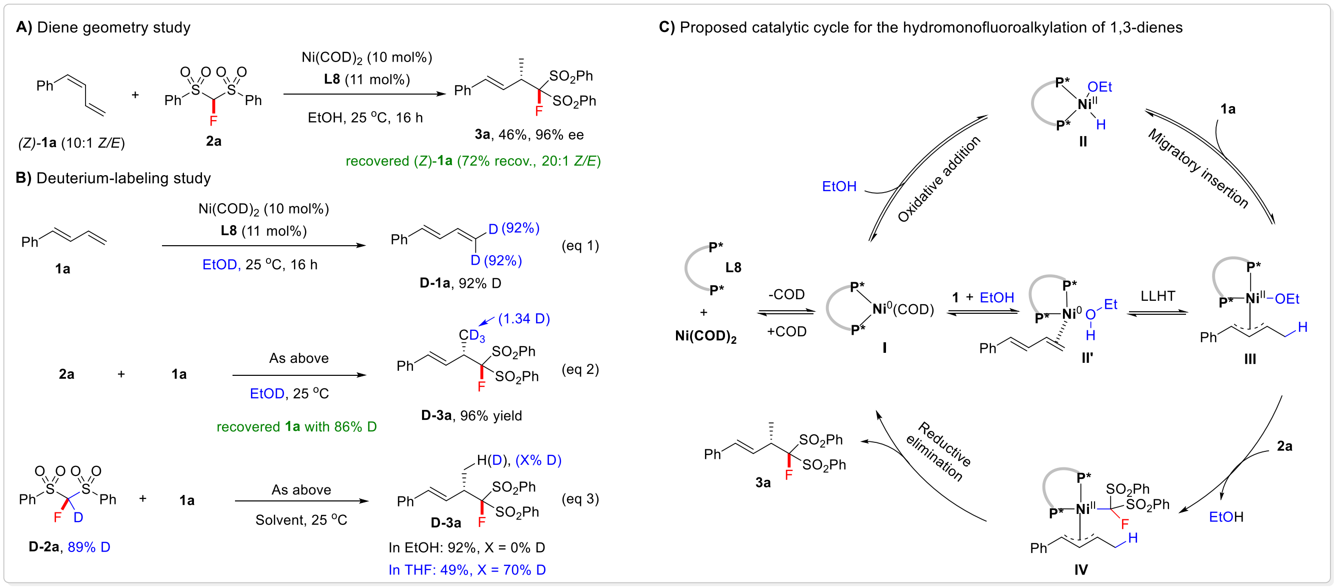

Scheme S1. Preliminary mechanistic investigations and a proposed mechanism.

Based on our preliminary mechanistic investigations, and literature precedents,[4] we propose a catalytic cycle shown in Scheme S1C. One of the possible pathway is that the oxidative addition of EtOH to Ni$^{(0)}$ species **I** forms Ni$^{(II)}$-H intermediate **II**. The migration insertion of diene **1a** into Ni$^{(II)}$-H

[4] (a) Cheng, L.; Li, M.-M.; Xiao, L.-J.; Xie, J.-H.; Zhou, Q.-L. *J. Am. Chem. Soc.* **2018**, *140*, 11627. (b) Xiao, L.-J.; Cheng, L.; Feng, W.-M.; Li, M.-L.; Xie, J.-H.; Zhou, Q.-L. *Angew. Chem. Int. Ed.* **2018**, *57*, 461. (c) Li, Z.-Q.; Fu, Y.; Deng, R.; Tran, V. T.; Gao, Y.; Liu, P.; Engle, K. M. *Angew. Chem. Int. Ed.* **2020**, *59*, 23306.

bond of **II** delivers π-allyl Ni$^{(II)}$ complex **III**, which might undergo a ligand exchange to give the species **IV** and EtOH. Subsequent reductive elimination of **IV** affords the target **3a** and regenerates the Ni$^{(0)}$ species **I**. Another pathway to form π-allyl Ni$^{(II)}$ **III** involving a LLHT process was also possible.[4a]

7.1 Diene geometry study

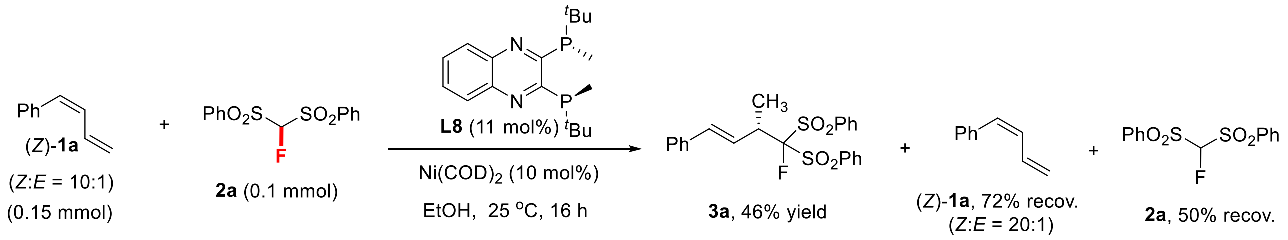

To an oven-dried Schlenk tube equipped with a stirring bar were successively added Ni(COD)$_2$ (2.8 mg, 10 mol%), (*S,S*)-QuinoxP* **L8** (3.7 mg, 11 mol%), 1,3-diene (*Z*)-**1a** (*Z:E* = 10:1, 19.5 mg, 0.15 mmol, 1.5 equivs), and FBSM **2a** (31.4 mg, 0.1 mmol), followed by the addition of absolute EtOH (1.0 mL) in a glove box. After it take out from the glove box, the resulting mixture was stirred at 25 °C for 16 h. The reaction mixture was concentrated under vacuum to give the crude residue, which was purified by silica gel column chromatography using PE/EtOAc (8:1 to 3:1, v/v) as the eluent to afford the product **3a** with 46% yield (21.1 mg), recovery the 72% of 1,3-diene (*Z*)-**1a** (14.1 mg) with 20:1 *Z/E* and 50% of **2a** (15.8 mg); HPLC analysis (Chiralcel OX-H, ^{i}PrOH/hexane = 20/80, 1.0 mL/min, 254 nm; t_r(minor) = 17.88 min, t_r(major) = 20.30 min) gave the isomeric composition of **3a**: 96% ee.

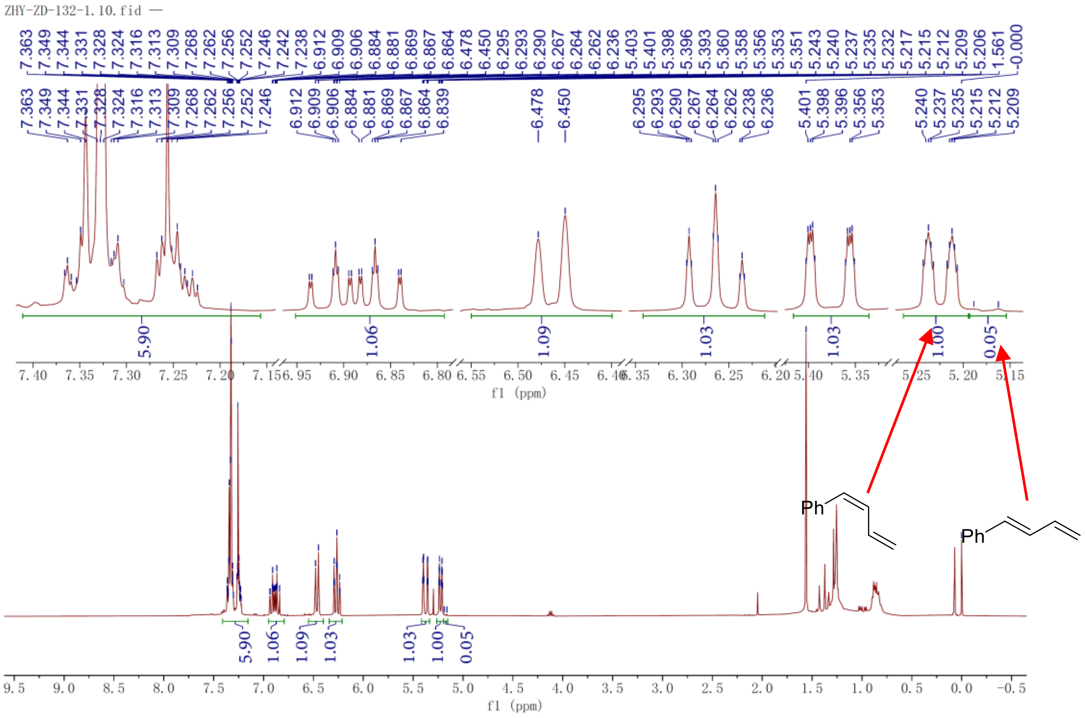

Figure S1. The ^{1}H NMR spectrum of the recovered 1,3-diene (*Z*)-**1a** with 20:1 *Z/E*.

7.2 Deuterium labeling study

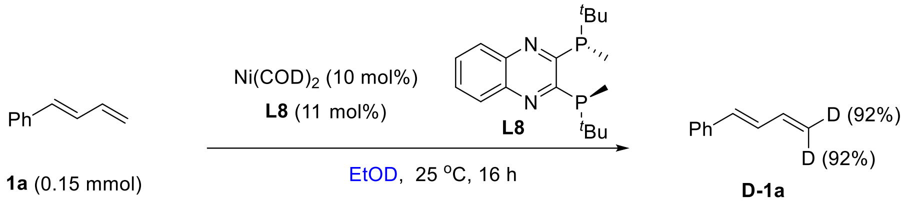

To an oven-dried Schlenk tube equipped with a stirring bar were successively added Ni(COD)$_2$ (2.8 mg, 10 mol%), (*S,S*)-QuinoxP* **L8** (3.7 mg, 11 mol%) and 1,3-dienes **1a** (19.5 mg, 0.15 mmol, 1.5 equivs) followed by the addition of absolute **EtOD** (1.0 mL) in a glove box. After it take out from the glove box, the resulting mixture was stirred at 25 °C for 16 h. The reaction mixture was concentrated under vacuum to give the crude residue, which was purified by silica gel column chromatography to recovery the deuterated 1,3-diene **D-1a** with 92% D incorporation (18.5 mg, 95% recovery).

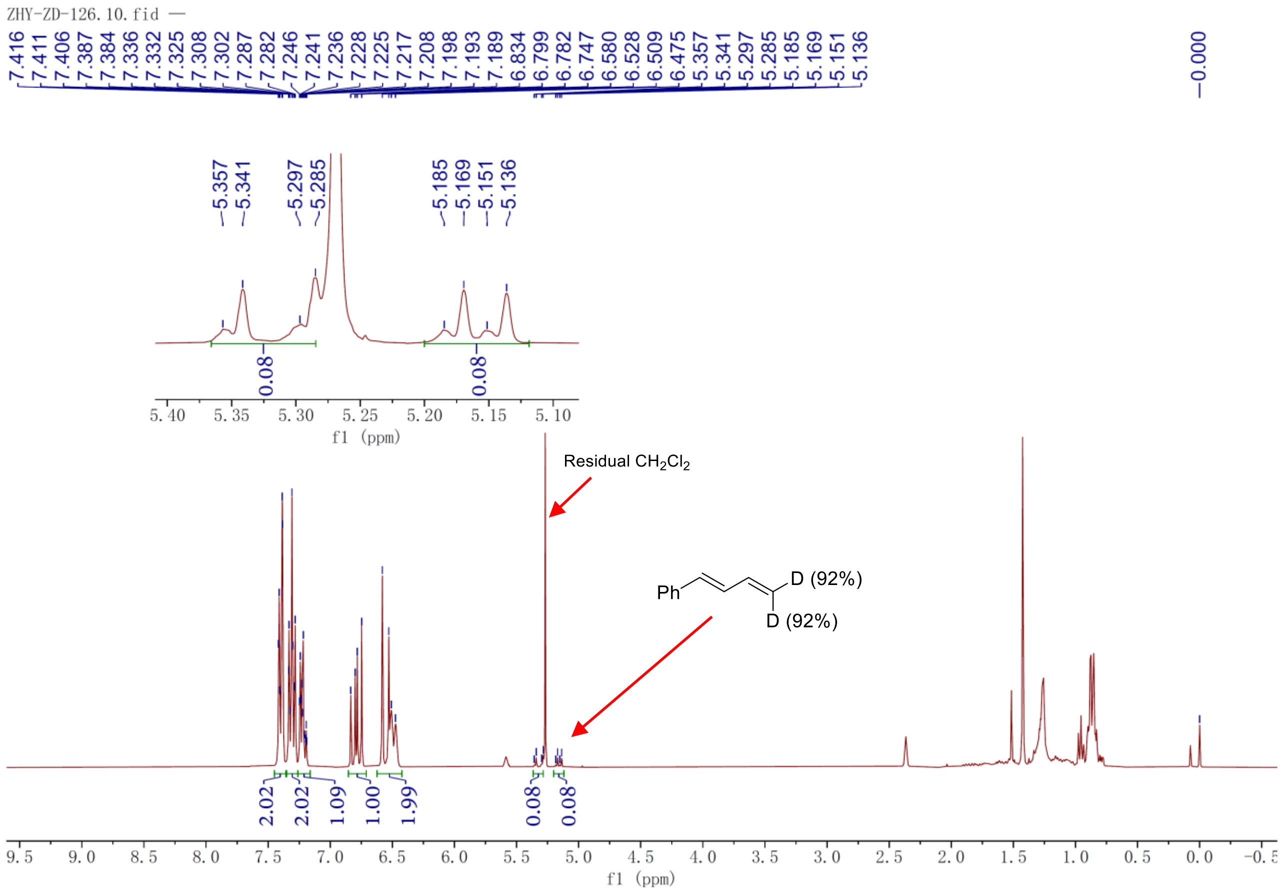

Figure S2. The deuterium incorporation ratio of the recovered 1,3-diene (*Z*)-**1a**.

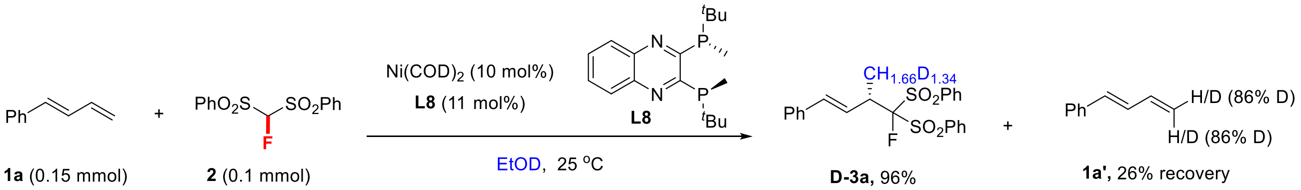

To an oven-dried Schlenk tube equipped with a stirring bar were successively added Ni(COD)$_2$ (2.8 mg, 10 mol%), (*S,S*)-QuinoxP* **L8** (3.7 mg, 11 mol%), 1,3-diene **1a** (19.5 mg, 0.15 mmol, 1.5 equivs), and FBSM **2** (31.4 mg, 0.1 mmol), followed by the addition of absolute EtOD (1.0 mL) in a glove box. After it take out from the glove box, the resulting mixture was stirred at 25 °C for 16 h. The reaction mixture was concentrated under vacuum to give the crude residue, which was purified by silica gel column chromatography using PE/EtOAc (8:1 to 6:1, v/v) as the eluent to afford the product **D-3a** in 96% yield (42.8 mg). ^{1}H NMR (400 MHz, $CDCl_3$): δ 7.95-7.92 (m, 2H), 7.82-7.80 (m, 2H), 7.72-7.68 (m, 1H), 7.61-7.57 (m, 1H), 7.56-7.51 (m, 2H), 7.44-7.39 (m, 2H), 7.31-7.21 (m, 5H), 6.31-6.22 (m, 2H), 3.52–3.45 (m, 1H), 1.69-1.64 (m, 1.66H).

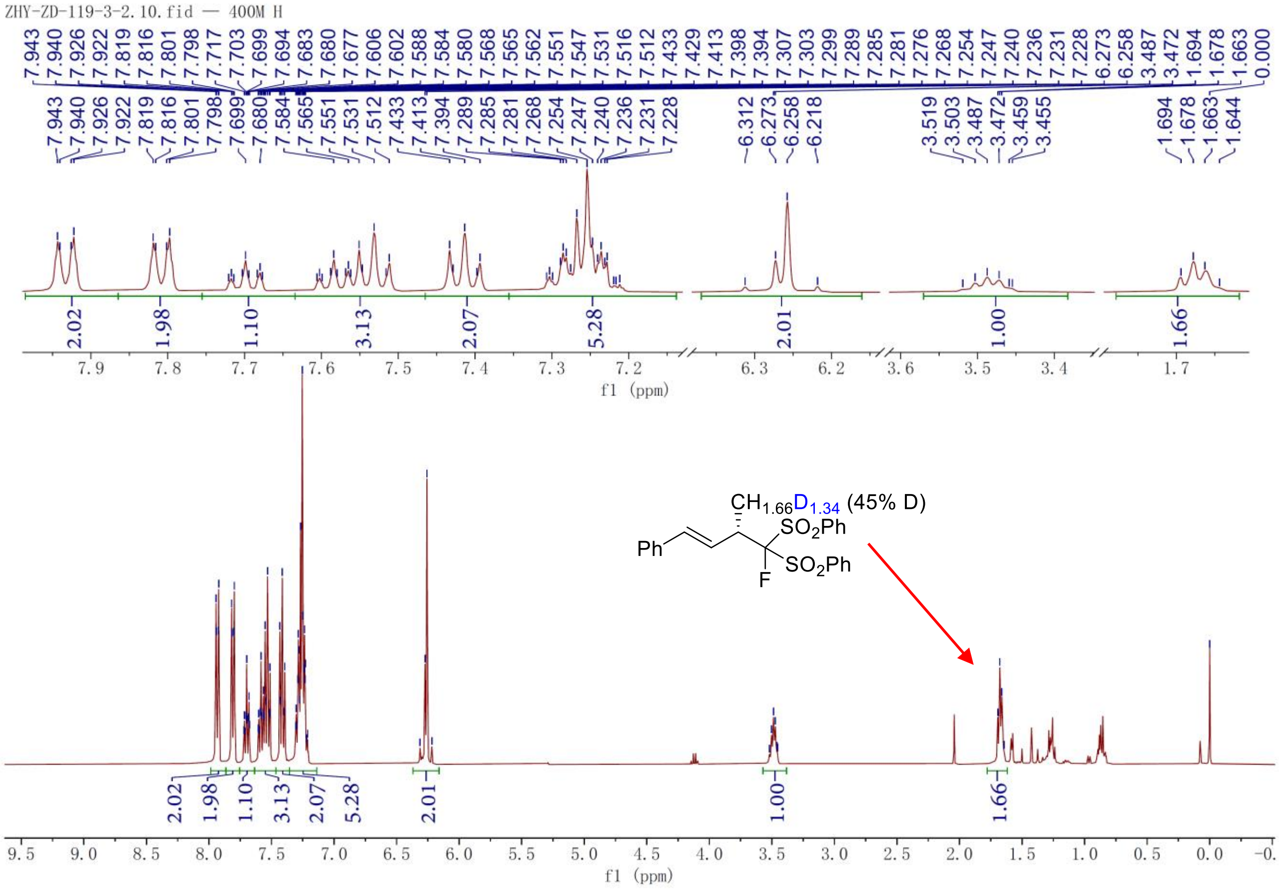

Figure S3. The product **3a** with 45% D (1.34 D) incorporation at the methyl group (in EtOD).

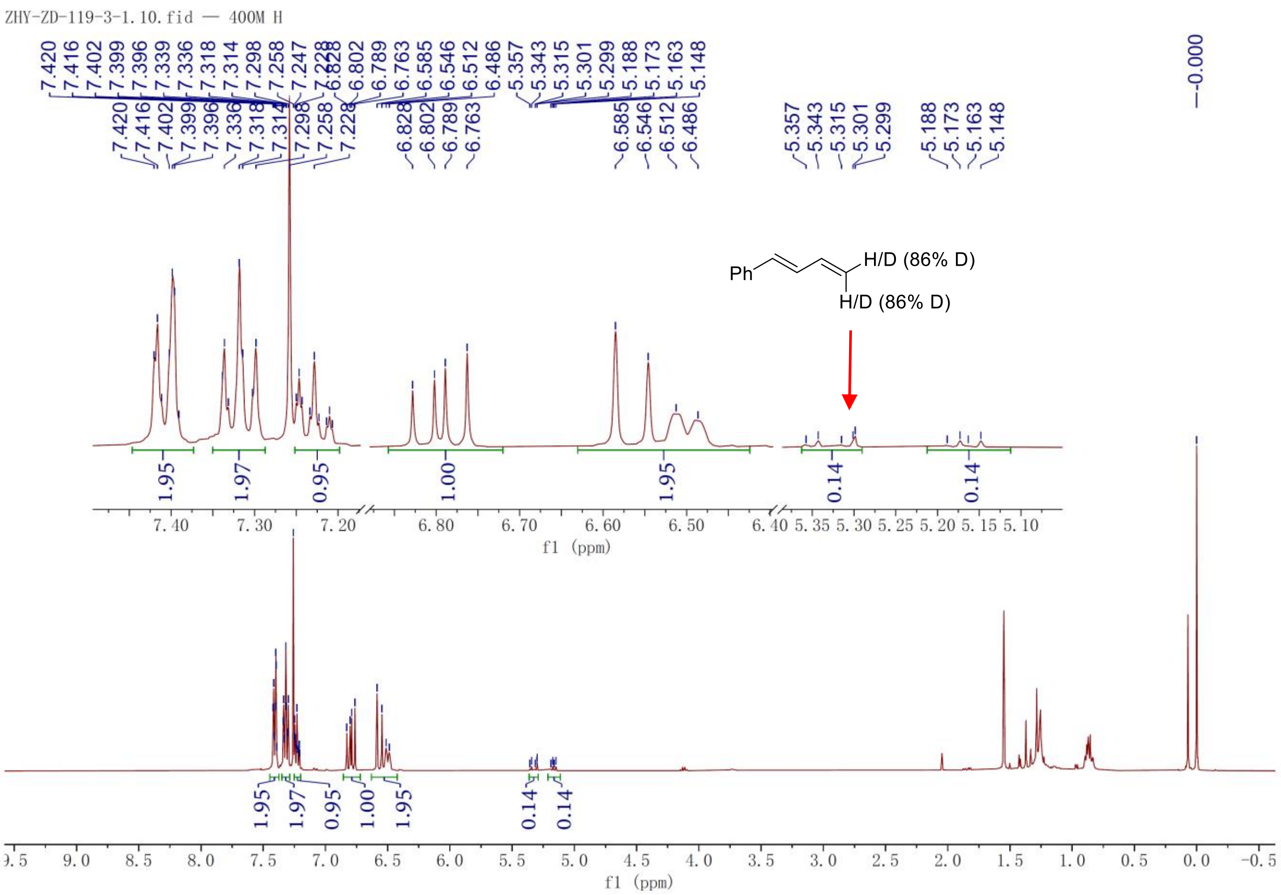

Figure S4. The recovered 1,3-diene **1a** with 86% D incorporation.

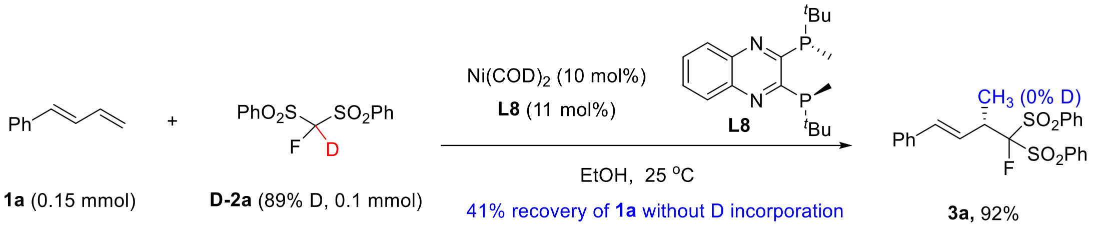

To an oven-dried Schlenk tube equipped with a stirring bar were successively added Ni(COD)$_2$ (2.8 mg, 10 mol%), (*S,S*)-QuinoxP* **L8** (3.7 mg, 11 mol%), 1,3-diene **1a** (19.5 mg, 0.15 mmol, 1.5 equivs), and deuterated FBSM **D-2a** (31.4 mg, 0.1 mmol, 89% D), followed by the addition of absolute EtOH (1.0 mL) in a glove box. After it take out from the glove box, the resulting mixture was stirred at 25 °C for 16 h. The reaction mixture was concentrated under vacuum to give the crude residue, which was purified by silica gel column chromatography using PE/EtOAc (8:1 to 3:1, v/v) as the eluent to afford the product **3a** in 92% yield (40.3 mg) without D incorporation, and recovery 41% of **1a** (9.4 mg) without D incorporation.

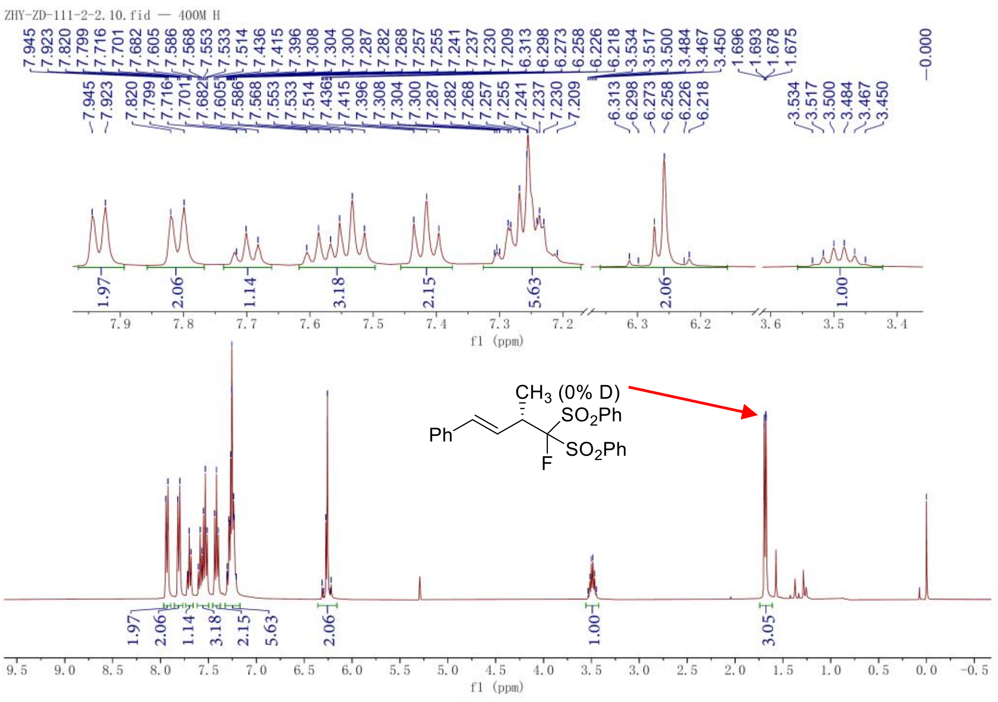

Figure S5. The ^{1}H NMR spectrum of the obtained product **3a** with 0% D incorporation.

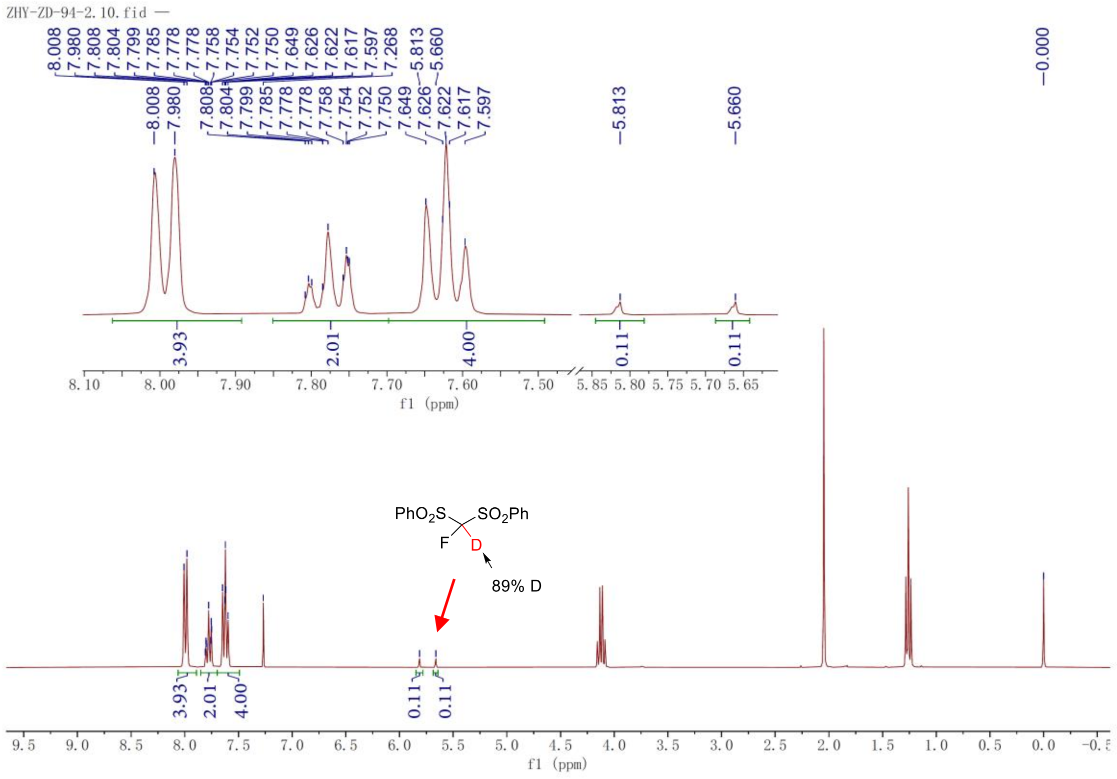

Figure S6. The ^{1}H NMR spectrum of the deuterated FBSM **D-2a** with 89% D incorporation.

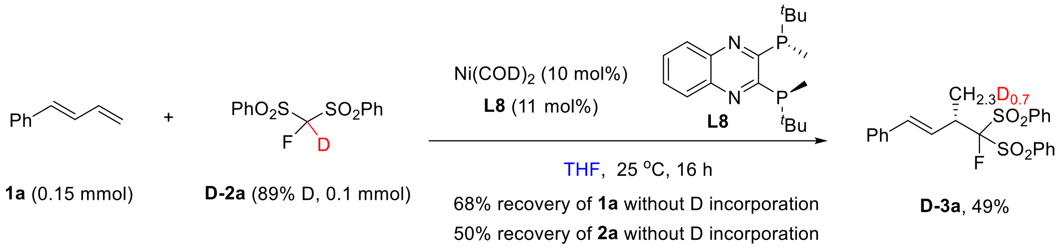

To an oven-dried Schlenk tube equipped with a stirring bar were successively added $Ni(COD)_2$ (2.8 mg, 10 mol%), (*S,S*)-QuinoxP* **L8** (3.7 mg, 11 mol%), 1,3-diene **1a** (19.5 mg, 0.15 mmol, 1.5 equivs), and deuterated FBSM **D-2a** (31.4 mg, 0.1 mmol), followed by the addition of anhydrous THF (1.0 mL) in a glove box. After it take out from the glove box, the resulting mixture was stirred at 25 °C for 16 h. The reaction mixture was concentrated under vacuum to give the crude residue, which was purified by silica gel column chromatography using PE/EtOAc (8:1 to 3:1, v/v) as the eluent to afford the product **D-3a** in 49% yield (21.6 mg) with 23% D incorporation, and recovery 68% of **1a** (13.6 mg) and 50% of **2a** (15.1 mg) without D incorporation. ^1H NMR (400 MHz, $CDCl_3$): δ 7.95-7.92 (m, 2H), 7.82-7.80 (m, 2H), 7.72-7.68 (m, 1H), 7.61-7.57 (m, 1H), 7.56-7.51 (m, 2H), 7.44-7.40 (m, 2H), 7.31-7.21 (m, 5H), 6.31-6.22 (m, 2H), 3.52–3.45 (m, 1H), 1.70-1.66 (m, 2.3H).

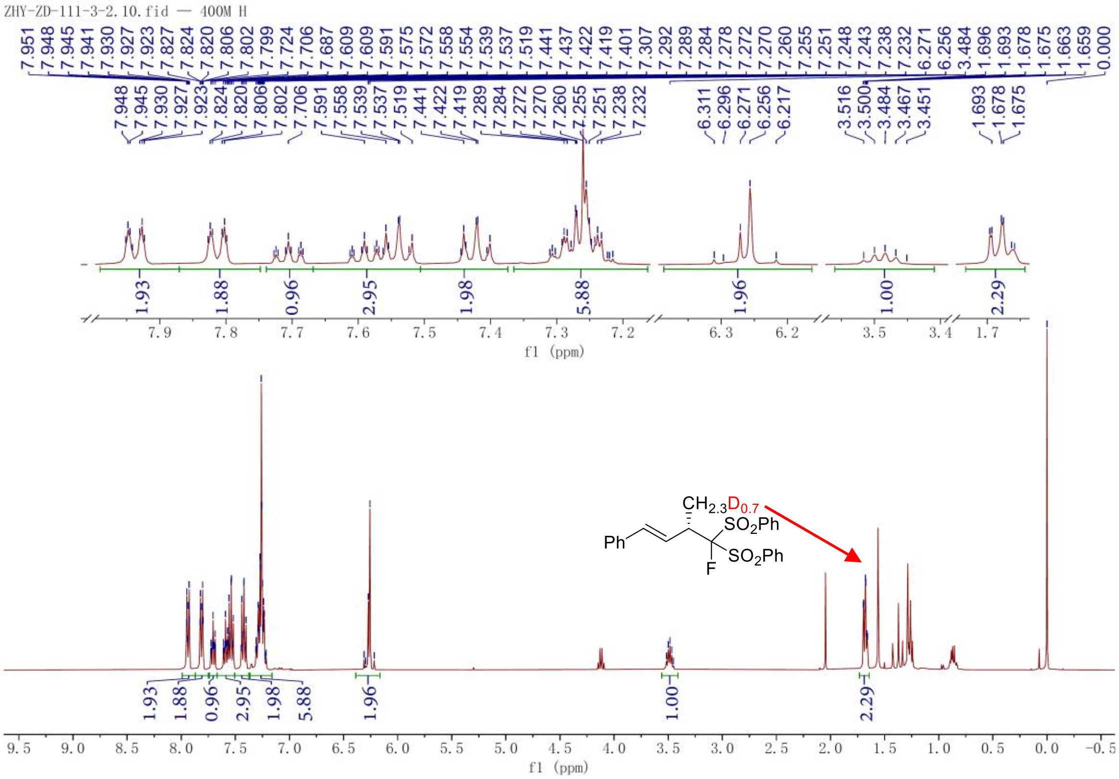

Figure S7. The ^1H NMR spectrum of the obtained **D-3a** with 23% D incorporation.

8. X-ray crystallographic data of 3a and 6t

Data intensity of **3a**[5] was collected using a 'Bruker APEX-II CCD' diffractometer at 150.00(10) K. Data collection and reduction were done by using Olex2 and the structure was solved with the ShelXS structure solution program using direct methods and refined by full-matrix least-squares on F^2 with anisotropic displacement parameters for non-H atoms using SHELX-97. Hydrogen atoms were added at their geometrically idea positions and refined isotropically. Crystal data for **3a**: $C_{23}H_{21}FO_4S_2$, T = 173.00(10) K, trigonal, $P2_12_12_1$, a = 7.55430(10) Å, b = 10.05800(10) Å, c = 28.1125(2) Å, α = 90°, β = 90°, γ = 90°, V = 2136.02(4) $Å^3$. Z = 4, ρ_{calc} = 1.382 g/cm^3. 46149 reflections collected, 3742 [Rint = 0.0374, Rsigma = 0.0133] independent reflections, R_1 = 0.0223, wR_2 = 0.0585 (I>=2σ (I)), R_1 = 0.0223, wR_2 = 0.0586 (all data), GOF = 1.097, and 0.010(3) parameters.

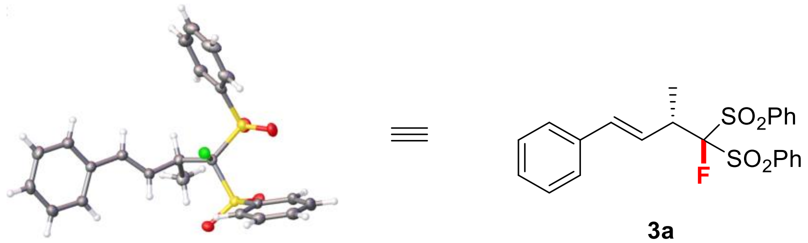

3a

Table S3. Crystal data and structure refinement for **3a.**

Identification code	**3a**
Empirical formula	$C_{23}H_{21}FO_4S_2$
Formula weight	444.52
Temperature/K	173.00(10)
Crystal system	orthorhombic
Space group	$P2_12_12_1$
a/Å	7.55430(10)
b/Å	10.05800(10)
c/Å	28.1125(2)
α/°	90
β/°	90
γ/°	90

[5] Supplementary crystallographic data have been deposited at Cambridge Crystallographic Data Center (CCDC number: 2130034).

Volume/Å^3	2136.02(4)
Z	4
ρ calcg/cm^3	1.382
μ /mm^{-1}	2.572
F(000)	928.0
Crystal size/mm^3	0.36 × 0.28 × 0.12
Radiation	CuKα (λ = 1.54184)
2Θ range for data collection/°	6.288 to 134.128
Index ranges	-9 ⩽ h ⩽ 9, -12 ⩽ k ⩽ 12, -33 ⩽ l ⩽ 33
Reflections collected	46149
Independent reflections	3742 [R_{int} = 0.0374, R_{sigma} = 0.0133]
Data/restraints/parameters	3742/0/272
Goodness-of-fit on F^2	1.097
Final R indexes [I>=2σ (I)]	R_1 = 0.0223, wR_2 = 0.0585
Final R indexes [all data]	R_1 = 0.0223, wR_2 = 0.0586
Largest diff. peak/hole / e Å^{-3}	0.20/-0.26
Flack parameter	0.010(3)

Table S4. Fractional atomic coordinates (×10^4) and equivalent isotropic displacement parameters (Å^2×10^3) for **3a**. U(eq) is defined as one third of the trace of the orthogonalized Uij tensor.

**Atom**	***x***	***y***	***z***	**U(eq)**
S1	5202.4(7)	5867.5(5)	6562.2(2)	23.42(12)
S2	7987.6(6)	4284.4(5)	6023.9(2)	24.73(12)
F1	4639.8(14)	3845.9(11)	6003.2(4)	23.2(2)
O1	3794(2)	5640.2(15)	6895.9(5)	30.5(3)
O2	6789(2)	6532.3(15)	6704.5(6)	35.0(4)
O3	7827(2)	5354.2(15)	5688.2(6)	36.7(4)
O4	9393.6(19)	4285.9(17)	6364.6(6)	33.8(3)
C1	1645(3)	-609(2)	6656.1(7)	27.3(4)
C2	74(3)	-1278(2)	6744.9(8)	31.5(5)

Atom	*x*	*y*	*z*	U(eq)
C3	-1199(3)	-712(2)	7033.7(8)	31.5(5)
C4	-876(3)	519(2)	7237.6(8)	32.9(5)
C5	680(3)	1191(2)	7146.6(7)	27.3(4)
C6	1974(3)	642.8(19)	6849.8(6)	21.9(4)
C7	3664(3)	1311(2)	6747.1(7)	23.3(4)
C8	4070(3)	2569(2)	6832.6(7)	24.0(4)
C9	5894(3)	3135(2)	6748.1(7)	23.0(4)
C10	5859(2)	4187(2)	6349.0(6)	20.8(4)
C11	4247(3)	6694.9(19)	6072.1(7)	23.9(4)
C12	2487(3)	6442(2)	5965.3(7)	25.4(4)
C13	1673(3)	7169(2)	5607.5(8)	31.3(5)
C14	2618(4)	8139(2)	5367.7(8)	35.8(5)
C15	4360(3)	8399(2)	5482.9(9)	38.0(6)
C16	5204(3)	7675(2)	5834.7(8)	30.6(5)
C17	8062(3)	2778(2)	5702.3(7)	23.9(4)
C18	7316(3)	2718(3)	5248.7(8)	34.5(5)
C19	7479(3)	1558(3)	4990.3(8)	41.6(6)
C20	8355(3)	478(3)	5179.6(9)	39.7(6)
C21	9070(3)	543(2)	5630.0(8)	37.8(5)
C22	8938(3)	1696(2)	5895.0(7)	29.9(5)
C23	6724(3)	3635(2)	7213.5(7)	32.4(5)

Table S5. Anisotropic Displacement Parameters ($Å^2×10^3$) for **3a**. The Anisotropic displacement factor exponent takes the form: $-2\pi^2[h^2a^{*2}U_{11}+2hka^*b^*U_{12}+\ldots]$.

Atom	U_{11}	U_{22}	U_{33}	U_{23}	U_{13}	U_{12}
S1	25.2(2)	18.3(2)	26.8(2)	-3.52(18)	2.92(18)	-3.5(2)
S2	20.0(2)	23.2(2)	31.0(2)	1.67(19)	5.63(18)	-2.78(19)
F1	21.0(5)	25.1(6)	23.4(5)	-2.8(4)	-2.2(4)	-3.4(4)
O1	36.1(8)	26.8(8)	28.7(7)	-2.8(6)	12.1(6)	0.7(7)

Atom	U_{11}	U_{22}	U_{33}	U_{23}	U_{13}	U_{12}
O2	34.3(8)	25.5(7)	45.2(9)	-8.4(7)	-6.3(7)	-9.1(7)
O3	37.5(9)	28.4(8)	44.3(9)	12.6(7)	16.9(8)	1.9(7)
O4	21.7(7)	36.3(8)	43.3(8)	-4.4(7)	-1.7(6)	-6.7(7)
C1	29.2(10)	23.8(10)	28.7(10)	-3.7(8)	4.7(8)	-0.9(9)
C2	36.1(11)	23.8(10)	34.7(11)	-3.7(8)	1.1(10)	-8.4(10)
C3	27.3(10)	30.8(11)	36.4(11)	7.2(9)	3.4(9)	-7.9(10)
C4	33.7(12)	29.2(11)	35.9(11)	4.3(9)	12.9(10)	1.6(10)
C5	31.4(11)	21.9(10)	28.7(10)	-2.3(8)	7.5(9)	-2.0(9)
C6	25.5(10)	20.2(9)	20.2(9)	1.1(7)	0.3(7)	-1.1(9)
C7	24.3(10)	20.8(9)	24.9(9)	-1.8(8)	3.3(8)	-0.1(8)
C8	25.3(11)	22.1(10)	24.5(10)	-0.2(8)	6.0(8)	-1.9(8)
C9	26.1(10)	19.9(9)	23.0(9)	-1.3(8)	0.1(8)	-1.1(8)
C10	19.2(9)	20.7(9)	22.6(9)	-3.0(8)	0.1(7)	-4.9(8)
C11	24.9(10)	17.2(9)	29.4(10)	-1.0(8)	7.2(8)	0.0(8)
C12	26.8(10)	20.5(9)	28.9(10)	-3.2(8)	6.3(8)	-2.5(8)
C13	30.6(12)	30.6(11)	32.7(11)	-4.2(9)	1.4(9)	3.8(10)
C14	46.8(14)	29.8(11)	30.7(11)	2.6(9)	7.8(10)	10.4(10)
C15	43.9(14)	26.7(11)	43.4(13)	9.7(10)	20.0(11)	4.5(11)
C16	*24.2(*11)	23.5(10)	44.1(12)	2.5(9)	12.0(9)	-0.5(9)
C17	18.8(9)	29.4(11)	23.3(9)	0.5(8)	4.5(8)	1.2(9)
C18	26.3(11)	49.7(14)	27.6(10)	1.3(10)	-0.1(9)	10.3(11)
C19	28.2(12)	66.9(17)	29.7(11)	-14.8(11)	-1.9(9)	5.0(12)
C20	34.2(13)	43.3(14)	41.7(13)	-14.7(11)	7.9(10)	-1.8(11)
C21	44.4(14)	31.2(12)	37.7(12)	-0.7(10)	8.2(11)	8.1(11)
C22	30.2(11)	34.2(12)	25.1(10)	1.7(9)	2.4(8)	5.5(10)
C23	39.2(12)	32.9(11)	25.1(10)	-0.2(9)	-6.6(9)	-4.3(10)

Table S6. Bond Lengths for **3a**

Atom	Atom	Length/Å	Atom	Atom	Length/Å
S1	O1	1.4366(15)	C7	C8	1.324(3)
S1	O2	1.4299(16)	C8	C9	1.509(3)
S1	C10	1.860(2)	C9	C10	1.543(3)
S1	C11	1.764(2)	C9	C23	1.535(3)
S2	O3	1.4364(16)	C11	C12	1.387(3)
S2	O4	1.4301(16)	C11	C16	1.393(3)
S2	C10	1.8522(19)	C12	C13	1.387(3)
S2	C17	1.765(2)	C13	C14	1.384(3)
F1	C10	1.382(2)	C14	C15	1.381(4)
C1	C2	1.386(3)	C15	C16	1.384(3)
C1	C6	1.395(3)	C17	C18	1.395(3)
C2	C3	1.382(3)	C17	C22	1.384(3)
C3	C4	1.386(3)	C18	C19	1.380(4)
C4	C5	1.380(3)	C19	C20	1.379(4)
C5	C6	1.399(3)	C20	C21	1.378(4)
C6	C7	1.471(3)	C21	C22	1.382(3)

Table S7. Bond Angles for **3a**.

Atom	Atom	Atom	Angle/°	Atom	Atom	Atom	Angle/°
O1	S1	C10	105.26(9)	C23	C9	C10	113.68(16)
O1	S1	C11	106.40(10)	S2	C10	S1	110.03(10)
O2	S1	O1	120.83(10)	F1	C10	S1	105.94(13)
O2	S1	C10	106.94(9)	F1	C10	S2	104.15(11)
O2	S1	C11	109.94(10)	F1	C10	C9	110.63(15)
C11	S1	C10	106.62(9)	C9	C10	S1	113.18(13)
O3	S2	C10	106.88(10)	C9	C10	S2	112.34(14)
O3	S2	C17	108.00(10)	C12	C11	S1	118.35(16)
O4	S2	O3	120.12(10)	C12	C11	C16	121.6(2)
O4	S2	C10	108.33(9)	C16	C11	S1	119.74(17)

Atom	Atom	Atom	Angle/°	Atom	Atom	Atom	Angle/°
O4	S2	C17	108.68(10)	C11	C12	C13	119.0(2)
C17	S2	C10	103.60(9)	C14	C13	C12	119.7(2)
C2	C1	C6	121.4(2)	C15	C14	C13	120.7(2)
C3	C2	C1	120.1(2)	C14	C15	C16	120.5(2)
C2	C3	C4	119.3(2)	C15	C16	C11	118.4(2)
C5	C4	C3	120.7(2)	C18	C17	S2	119.50(17)
C4	C5	C6	120.84(19)	C22	C17	S2	119.30(16)
C1	C6	C5	117.68(19)	C22	C17	C18	121.1(2)
C1	C6	C7	119.40(18)	C19	C18	C17	118.8(2)
C5	C6	C7	122.89(18)	C20	C19	C18	120.4(2)
C8	C7	C6	127.05(19)	C21	C20	C19	120.3(2)
C7	C8	C9	122.93(19)	C20	C21	C22	120.5(2)
C8	C9	C10	110.96(16)	C21	C22	C17	118.9(2)
C8	C9	C23	111.23(17)				

Table S8. Hydrogen Atom Coordinates (Å×10^4) and Isotropic Displacement Parameters (Å^2×10^3) for **3a**.

Atom	*x*	*y*	*z*	U(eq)
H1	2496.23	-1004.13	6463.34	33
H2	-122.54	-2109	6609.76	38
H3	-2261.18	-1151.63	7090.69	38
H4	-1717.41	896.25	7437.96	40
H5	871.36	2019.52	7284.48	33
H7	4541.12	794.26	6607	28
H8	3193.51	3128.6	6950.05	29
H9	6636.66	2401.37	6634.82	28
H12	1860.92	5794.91	6131.21	30
H13	497.07	7004.86	5529.08	38
H14	2072.41	8620.45	5126.34	43

H15	4971.13	9065.2	5322.76	46
H16	6382.2	7839.4	5910.77	37
H18	6718.69	3446.06	5122.95	41
H19	6996.26	1504.35	4686.62	50
H20	8462.99	-299.1	5002.42	48
H21	9645.67	-194	5756.44	45
H22	9430.28	1744.13	6197.79	36
H23A	5985.23	4313.54	7348.78	49
H23B	7875.93	3995.34	7149.09	49
H23C	6828.96	2909.33	7433.42	49
H1	2496.23	-1004.13	6463.34	33
H2	-122.54	-2109	6609.76	38

Data intensity of **6t**[6] was collected using a 'Bruker APEX-II CCD' diffractometer at 150.00(10) K. Data collection and reduction were done by using Olex2 and the structure was solved with the ShelXS structure solution program using direct methods and refined by full-matrix least-squares on F^2 with anisotropic displacement parameters for non-H atoms using SHELX-97. Hydrogen atoms were added at their geometrically idea positions and refined isotropically. Crystal data for **6t**: $C_{21}H_{23}FO_4$, T = 173.00(10) K, trigonal, $P2_1$, a = 8.0920(2) Å, b = 5.94840(10) Å, c = 19.5057(4) Å, α = 90°, β = 98.051(2)°, γ = 90°, V = 929.64(3) Å^3. Z = 2, ρ_{calc} = 1.280 g/cm^3. 18308 reflections collected, 3278 [R(int) = 0.0550, R(sigma) = 0.0357] independent reflections, R_1 = 0.0289, wR_2 = 0.0721 (I = 2σ(I), final), R_1 = 0.0312, wR_2 = 0.0733 (all data), GOF = 1.044, and 0.03(9) parameters.

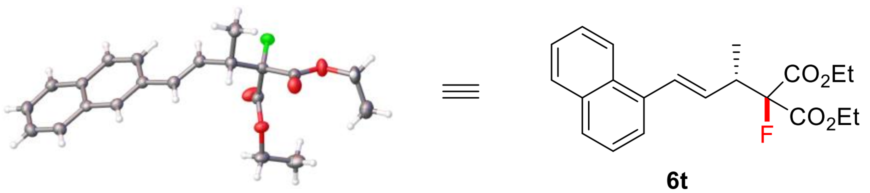

6t

Table S9. Crystal data and structure refinement for **6t.**

Identification code	**6t**
Empirical formula	$C_{21}H_{23}FO_4$

[6] Supplementary crystallographic data have been deposited at Cambridge Crystallographic Data Center (CCDC number: 2130032).

Formula weight	358.39
Temperature/K	173.00(10)
Crystal system	monoclinic
Space group	$P2_1$
a/Å	8.0920(2)
b/Å	5.94840(10)
c/Å	19.5057(4)
α /°	90
β /°	98.051(2)
γ /°	90
Volume/$Å^3$	929.64(3)
Z	2
ρ_{calc}/g/cm^3	1.280
μ /mm-1	0.778
F(000)	380.0
Crystal size/mm3	0.32 × 0.28 × 0.16
Radiation	CuKα (λ = 1.54184)
2Θ range for data collection/°	9.158 to 134.104
Index ranges	-9 ≤ h ≤ 9, -7 ≤ k ≤ 7, -23 ≤ l ≤ 23
Reflections collected	18308
Independent reflections	3278 [R(int) = 0.0550, R(sigma) = 0.0357]
Data/restraints/parameters	3278/1/239
Goodness-of-fit on F_2	1.044
Final R indexes [I>=2σ (I)]	R_1 = 0.0289, wR_2 = 0.0721
Final R indexes [all data]	R_1 = 0.0312, wR_2 = 0.0733
Largest diff. peak/hole / e $Å^{-3}$	0.14/-0.12
Flack parameter	0.03(9)

Table S10. Fractional atomic coordinates (×10[4]) and equivalent isotropic displacement parameters (Å[2]×10[3]) for **6t**. U(eq) is defined as one third of the trace of the orthogonalized Uij tensor.

**Atom**	***x***	***y***	***z***	**U(eq)**
F1	6893.7(15)	7041.7(19)	8581.6(6)	32.6(3)
O1	4801(2)	2064(3)	8734.8(7)	38.2(4)
O2	6782(2)	3848(3)	9464.5(7)	40.4(4)
O3	9348(2)	5042(3)	8072.7(9)	44.4(4)
O4	7961.3(17)	1766(2)	7999.6(7)	30.6(3)
C1	8037(2)	8740(3)	4784.6(10)	25.2(4)
C2	8750(3)	9676(4)	4225.1(10)	29.3(4)
C3	8643(3)	8582(4)	3607.3(11)	32.4(5)
C4	7801(3)	6503(4)	3513.3(10)	33.7(5)
C5	7112(3)	5542(4)	4041.7(10)	29.5(5)
C6	7219(2)	6621(3)	4695.6(9)	24.6(4)
C7	6546(2)	5658(3)	5261.8(10)	25.3(4)
C8	6673(2)	6702(3)	5896.7(10)	25.9(4)
C9	7477(3)	8841(3)	5973.1(10)	28.6(5)
C10	8133(3)	9811(3)	5440.2(10)	29.4(5)
C11	5994(3)	5599(3)	6469.1(10)	27.8(4)
C12	5894(3)	6442(3)	7092.5(10)	28.4(4)
C13	5172(3)	5225(4)	7657.5(10)	27.9(4)
C14	6499(3)	4937(3)	8296.4(10)	26.0(4)
C15	5889(3)	3431(3)	8850.5(10)	28.3(4)
C16	6461(4)	2411(5)	10043.5(11)	45.3(6)
C17	7660(4)	495(5)	10119.0(13)	54.1(7)
C18	8125(3)	3955(3)	8109.9(10)	27.0(4)
C19	9489(3)	564(4)	7901.6(12)	36.3(5)
C20	10552(3)	174(5)	8581.0(14)	47.5(6)
C21	3667(3)	6519(4)	7855.4(11)	36.7(5)

Table S11. Anisotropic Displacement Parameters ($Å^2 \times 10^3$) for **6t**. The Anisotropic displacement factor exponent takes the form: $-2\pi^2[h^2a^{*2}U_{11}+2hka^*b^*U_{12}+\ldots]$.

Atom	U_{11}	U_{22}	U_{33}	U_{23}	U_{13}	U_{12}
F1	38.0(7)	24.2(6)	34.3(6)	-5.3(5)	0.9(5)	-1.5(5)
O1	38.9(9)	41.0(9)	34.9(8)	3.0(7)	6.4(7)	-9.9(8)
O2	48.0(10)	47.4(10)	24.1(7)	3.6(6)	-0.8(6)	-9.0(8)
O3	31.8(9)	30.5(8)	73.8(11)	6.9(8)	17.3(8)	-0.7(7)
O4	29.0(8)	24.3(7)	39.0(7)	-0.4(6)	6.4(6)	2.1(6)
C1	22.6(11)	24.4(10)	27.8(10)	2.3(8)	1.1(8)	2.0(8)
C2	27.5(11)	26.8(10)	33.4(11)	2.7(8)	3.7(8)	0.5(9)
C3	31.4(12)	38.3(12)	28.8(10)	7.0(9)	9.1(9)	3.9(9)
C4	34.3(12)	38.5(12)	28.8(10)	-3.9(9)	6.4(8)	1.6(10)
C5	27.8(11)	29.9(11)	30.7(10)	-3.7(8)	4.1(8)	-1.2(9)
C6	18.7(10)	26.5(10)	27.8(9)	-0.5(8)	0.2(7)	3.0(8)
C7	21.2(10)	23.9(10)	30.3(10)	0.5(8)	1.4(8)	-1.3(8)
C8	22.5(10)	26.5(10)	27.9(9)	0.3(8)	1.4(7)	0.8(8)
C9	31.6(12)	27.8(10)	25.7(10)	-3.1(8)	1.4(8)	-3.2(9)
C10	29.3(11)	26.0(10)	31.6(10)	-1.6(8)	0.1(8)	-3.5(9)
C11	26.8(11)	27.0(10)	29.2(10)	1.4(8)	2.5(8)	-1.6(9)
C12	27.2(11)	28.2(10)	28.9(10)	1.4(8)	1.2(8)	0.7(8)
C13	27.9(11)	29.7(10)	25.9(10)	0.3(8)	2.8(8)	0.6(9)
C14	30.5(11)	21.8(10)	25.7(9)	-2.7(7)	3.4(8)	-1.7(8)
C15	29.6(11)	30.4(11)	25.3(9)	0.1(8)	5.8(8)	3.3(9)
C16	59.6(17)	53.4(15)	23.2(10)	7.8(10)	6.8(10)	-1.4(12)
C17	67.4(19)	56.3(16)	38.6(13)	10.2(12)	7.5(12)	4.1(14)
C18	28.9(12)	24.3(10)	27.7(10)	4.8(8)	4.2(8)	-0.5(9)
C19	35.9(13)	32.2(11)	43.8(12)	2.7(9)	16.6(10)	7.8(10)
C20	34.6(14)	42.8(14)	62.5(16)	-8.5(12)	-2.5(11)	10.3(11)
C21	30.1(12)	44.7(14)	35.2(11)	2.3(10)	3.9(9)	7.4(10)

Table S12. Bond Lengths for **6t**.

Atom	Atom	Length/Å	Atom	Atom	Length/Å
F1	C14	1.389(2)	C6	C7	1.419(3)
O1	C15	1.196(3)	C7	C8	1.376(3)
O2	C15	1.333(2)	C8	C9	1.427(3)
O2	C16	1.468(3)	C8	C11	1.467(3)
O3	C18	1.193(3)	C9	C10	1.360(3)
O4	C18	1.323(2)	C11	C12	1.328(3)
O4	C19	1.463(3)	C12	C13	1.504(3)
C1	C2	1.418(3)	C13	C14	1.537(3)
C1	C6	1.423(3)	C13	C21	1.535(3)
C1	C10	1.421(3)	C14	C15	1.538(3)
C2	C3	1.361(3)	C14	C18	1.529(3)
C3	C4	1.411(3)	C16	C17	1.490(4)
C4	C5	1.364(3)	C19	C20	1.494(3)
C5	C6	1.420(3)			

Table S13. Bond Angles for **6t**.

Atom	Atom	Atom	Angle/°	Atom	Atom	Atom	Angle/°
C15	O2	C16	116.79(18)	C11	C12	C13	124.68(19)
C18	O4	C19	115.78(17)	C12	C13	C14	110.40(16)
C2	C1	C6	119.10(18)	C12	C13	C21	110.29(17)
C2	C1	C10	122.67(19)	C21	C13	C14	109.95(16)
C10	C1	C6	118.21(18)	F1	C14	C13	108.65(15)
C3	C2	C1	120.8(2)	F1	C14	C15	108.79(15)
C2	C3	C4	120.2(2)	F1	C14	C18	106.41(16)
C5	C4	C3	120.67(19)	C13	C14	C15	112.22(16)
C4	C5	C6	120.6(2)	C18	C14	C13	112.13(15)
C5	C6	C1	118.63(18)	C18	C14	C15	108.43(16)

Atom	Atom	Atom	Angle/°	Atom	Atom	Atom	Angle/°
C7	C6	C1	119.04(17)	O1	C15	O2	126.04(19)
C7	C6	C5	122.33(19)	O1	C15	C14	124.31(18)
C8	C7	C6	121.99(19)	O2	C15	C14	109.63(17)
C7	C8	C9	118.15(19)	O2	C16	C17	109.8(2)
C7	C8	C11	119.63(18)	O3	C18	O4	126.1(2)
C9	C8	C11	122.22(18)	O3	C18	C14	123.78(19)
C10	C9	C8	121.28(19)	O4	C18	C14	110.16(17)
C9	C10	C1	121.31(19)	O4	C19	C20	110.63(18)
C12	C11	C8	127.3(2)				

Table S14. Torsion angles for **6t.**

A	B	C	Angle/°	A	B	C	Angle/°
C15	O2	C16	116.79(18)	C11	C12	C13	124.68(19)
C18	O4	C19	115.78(17)	C12	C13	C14	110.40(16)
C2	C1	C6	119.10(18)	C12	C13	C21	110.29(17)
C2	C1	C10	122.67(19)	C21	C13	C14	109.95(16)
C10	C1	C6	118.21(18)	F1	C14	C13	108.65(15)
C3	C2	C1	120.8(2)	F1	C14	C15	108.79(15)
C2	C3	C4	120.2(2)	F1	C14	C18	106.41(16)
C5	C4	C3	120.67(19)	C13	C14	C15	112.22(16)
C4	C5	C6	120.6(2)	C18	C14	C13	112.13(15)
C5	C6	C1	118.63(18)	C18	C14	C15	108.43(16)
C7	C6	C1	119.04(17)	O1	C15	O2	126.04(19)
C7	C6	C5	122.33(19)	O1	C15	C14	124.31(18)
C8	C7	C6	121.99(19)	O2	C15	C14	109.63(17)
C7	C8	C9	118.15(19)	O2	C16	C17	109.8(2)
C7	C8	C11	119.63(18)	O3	C18	O4	126.1(2)
C9	C8	C11	122.22(18)	O3	C18	C14	123.78(19)
C10	C9	C8	121.28(19)	O4	C18	C14	110.16(17)

A	B	C	Angle/°	A	B	C	Angle/°
C9	C10	C1	121.31(19)	O4	C19	C20	110.63(18)
C12	C11	C8	127.3(2)				

Table S15. Hydrogen Atom Coordinates (Å×10^4) and Isotropic Displacement Parameters (Å^2×10^3) for **6t**.

Atom	*x*	*y*	*z*	U(eq)
H2	9297.33	11052.32	4280.76	35
H3	9126.68	9206.96	3246.04	39
H4	7714.83	5781.89	3086.77	40
H5	6566.71	4166.92	3972.85	35
H7	6003.54	4278.72	5202.18	30
H9	7552.85	9586.71	6395.43	34
H10	8655.4	11201.97	5506.2	35
H11	5588.67	4146.71	6386.45	33
H12	6300.4	7888.39	7186.17	34
H13	4798.03	3733.77	7487.5	33
H16A	5327.58	1842.35	9959.1	54
H16B	6583.73	3282.44	10467.9	54
H17A	7501.08	-401.83	9705.83	81
H17B	7469.85	-411.09	10507.61	81
H17C	8780.91	1064.38	10192.06	81
H19A	10107.66	1434.84	7602.83	4
H19B	9201.3	-867.88	7678.19	44
H20A	11538.32	-636.24	8507.51	71
H20B	9937.88	-686.64	8876.6	71
H20C	10863.38	1592.77	8795.42	71
H21A	4028.6	7952.91	8046.32	55
H21B	3169.85	5674.2	8192.91	55
H21C	2860.27	6738.45	7450.75	55

9. NMR spectra

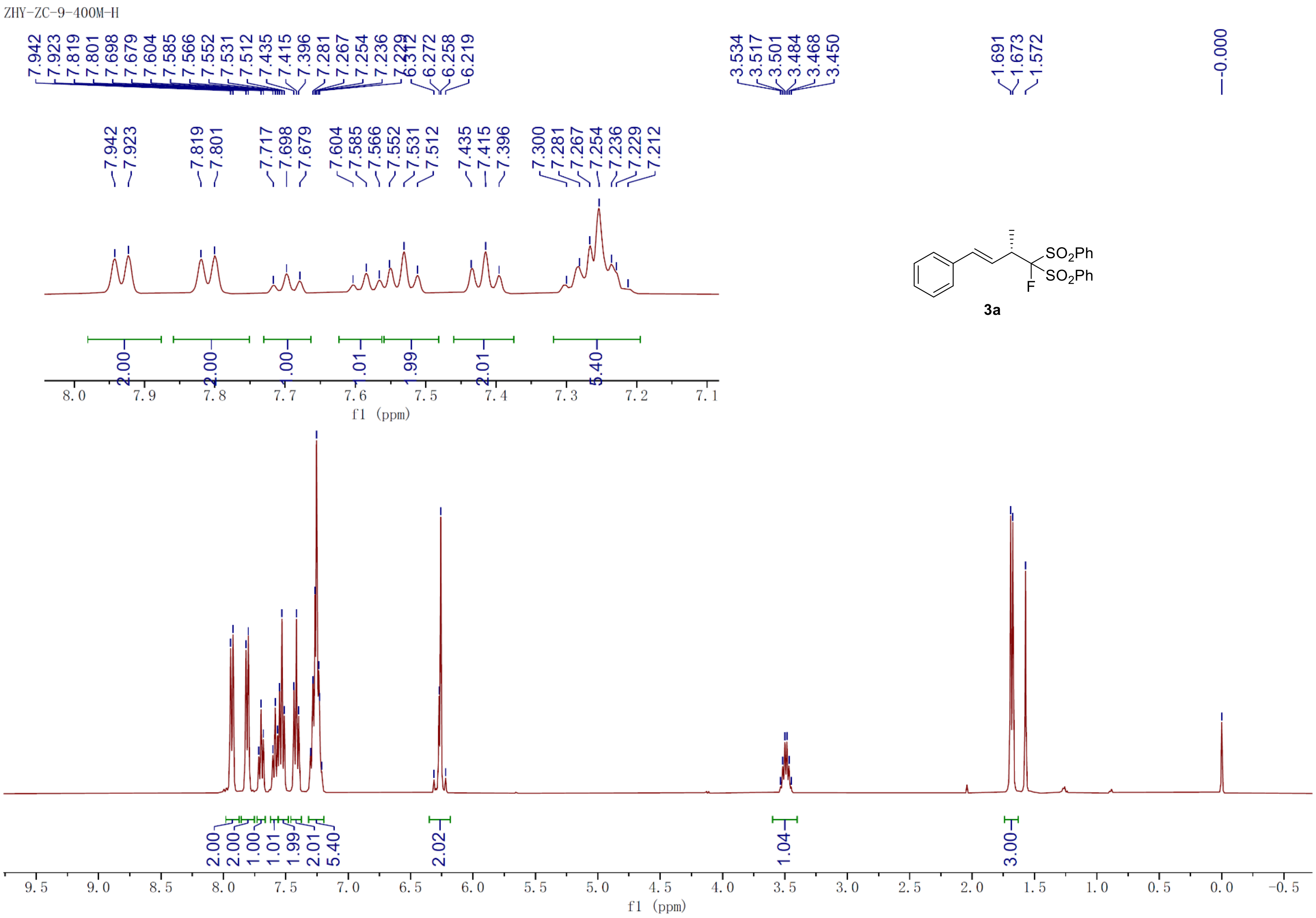

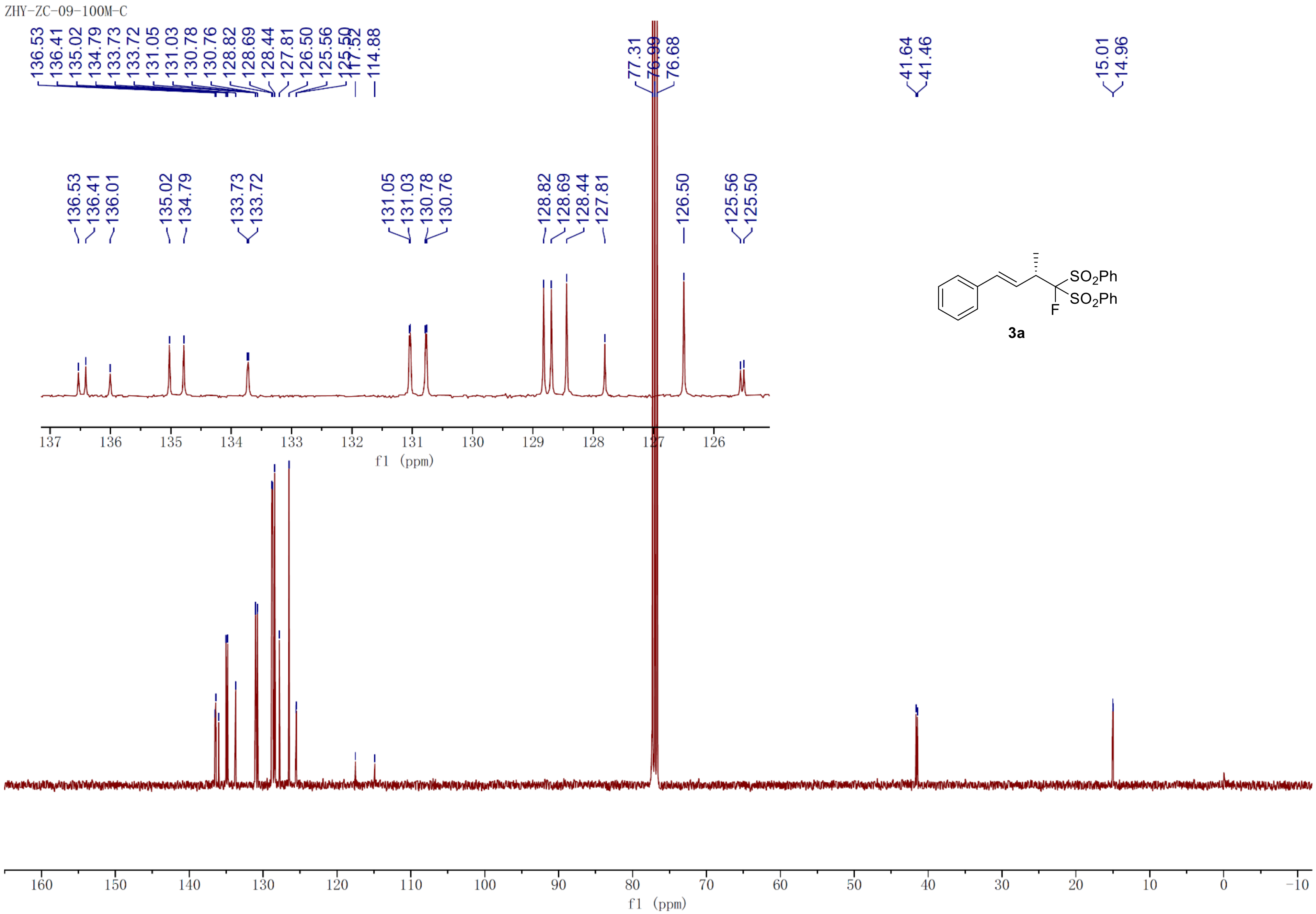
ZHY-ZC-09-100M-C
136.53
136.41
135.02
134.79
133.73
133.72
131.05
131.03
130.78
130.76
128.82
128.69
128.44
127.81
126.50
125.56
125.50
117.52
114.88
77.31
76.99
76.68
41.64
41.46
15.01
14.96
136.53
136.41
136.01
135.02
134.79
133.73
133.72
131.05
131.03
130.78
130.76
128.82
128.69
128.44
127.81
126.50
125.56
125.50
f1 (ppm)
SO2Ph
SO2Ph
F
3a
f1 (ppm)

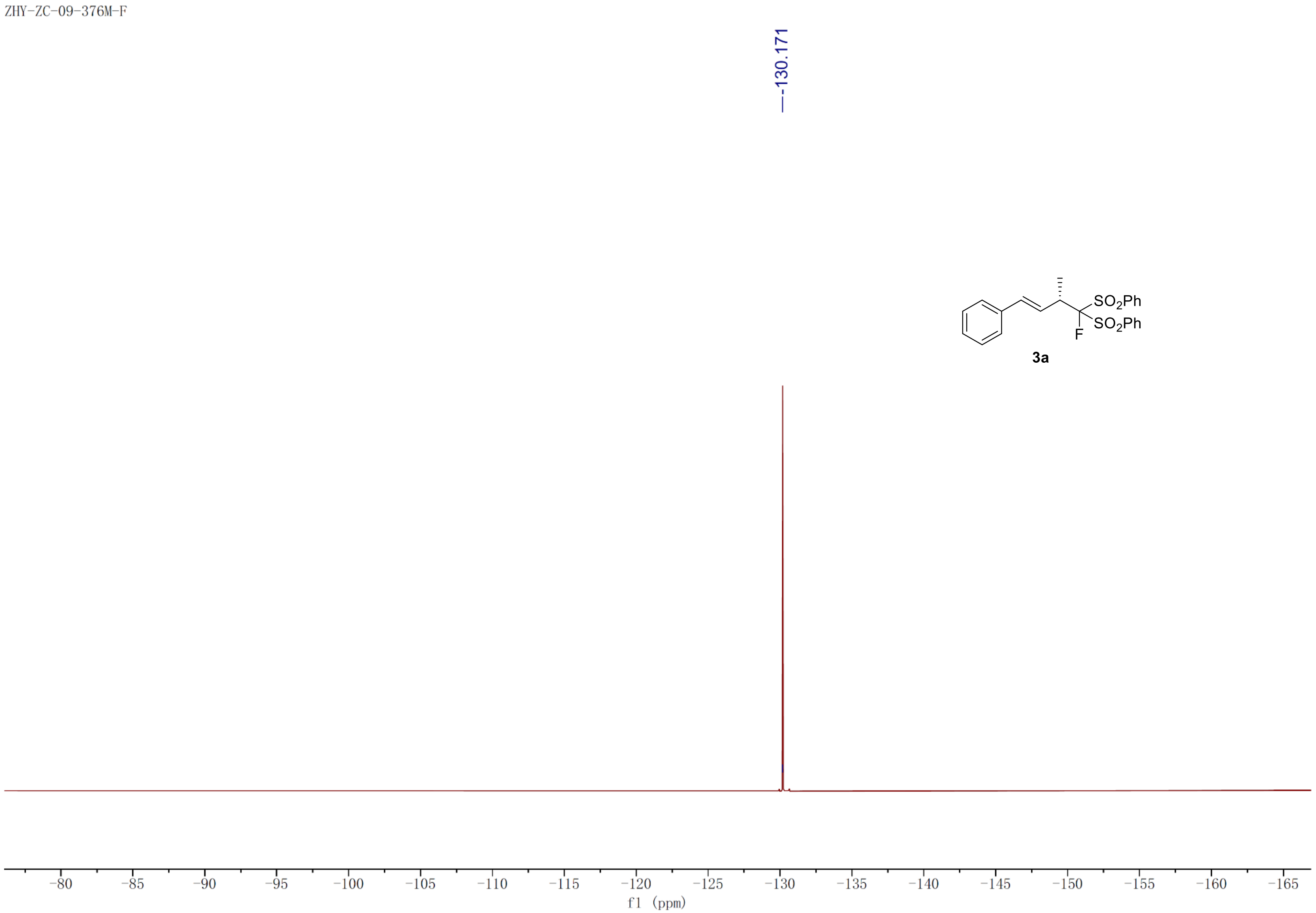
ZHY-ZC-09-376M-F
-130.171
SO2Ph
SO2Ph
F
3a
−80 −85 −90 −95 −100 −105 −110 −115 −120 −125 −130 −135 −140 −145 −150 −155 −160 −165
f1 (ppm)

ZHY-ZC-98-400M-H

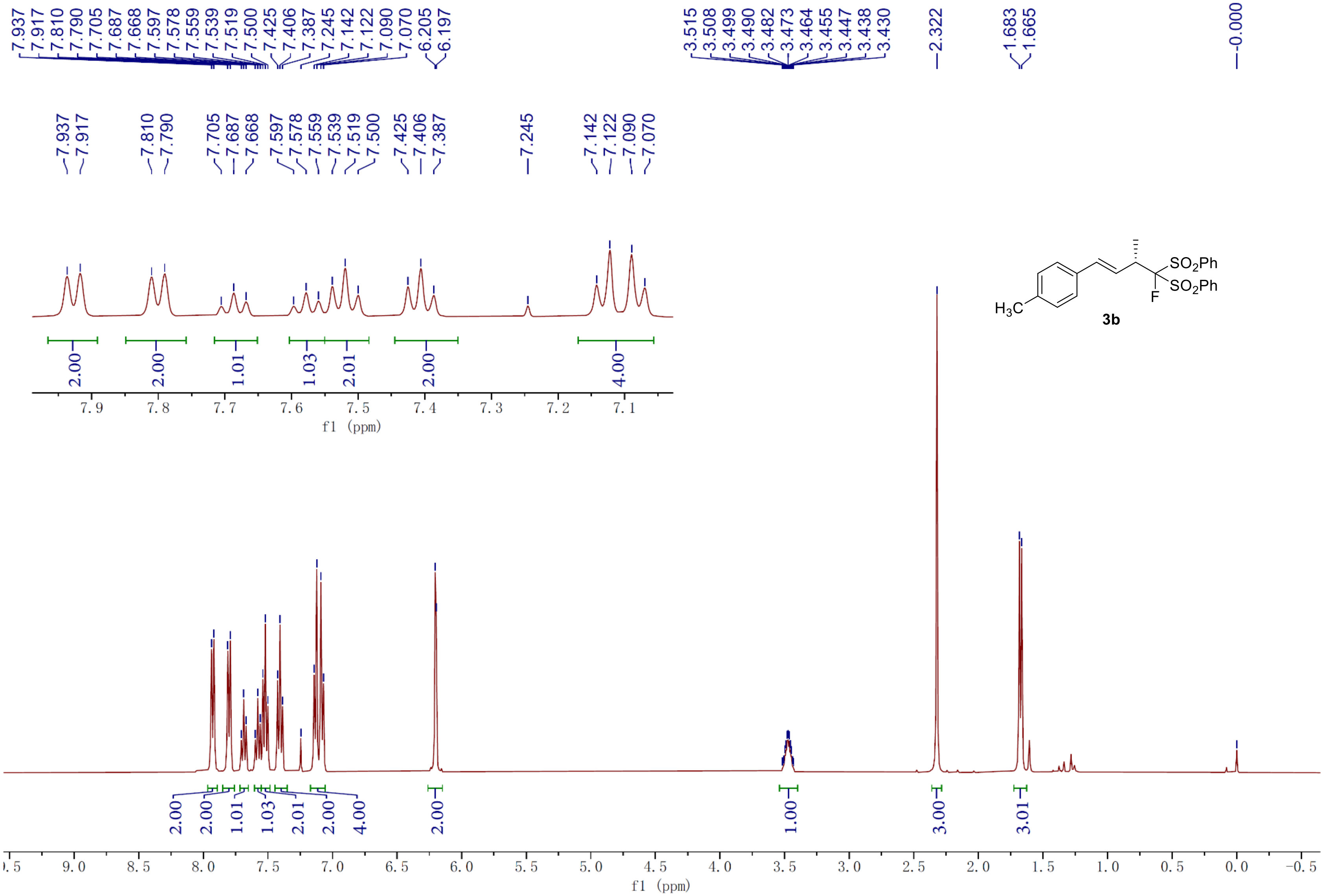

3b

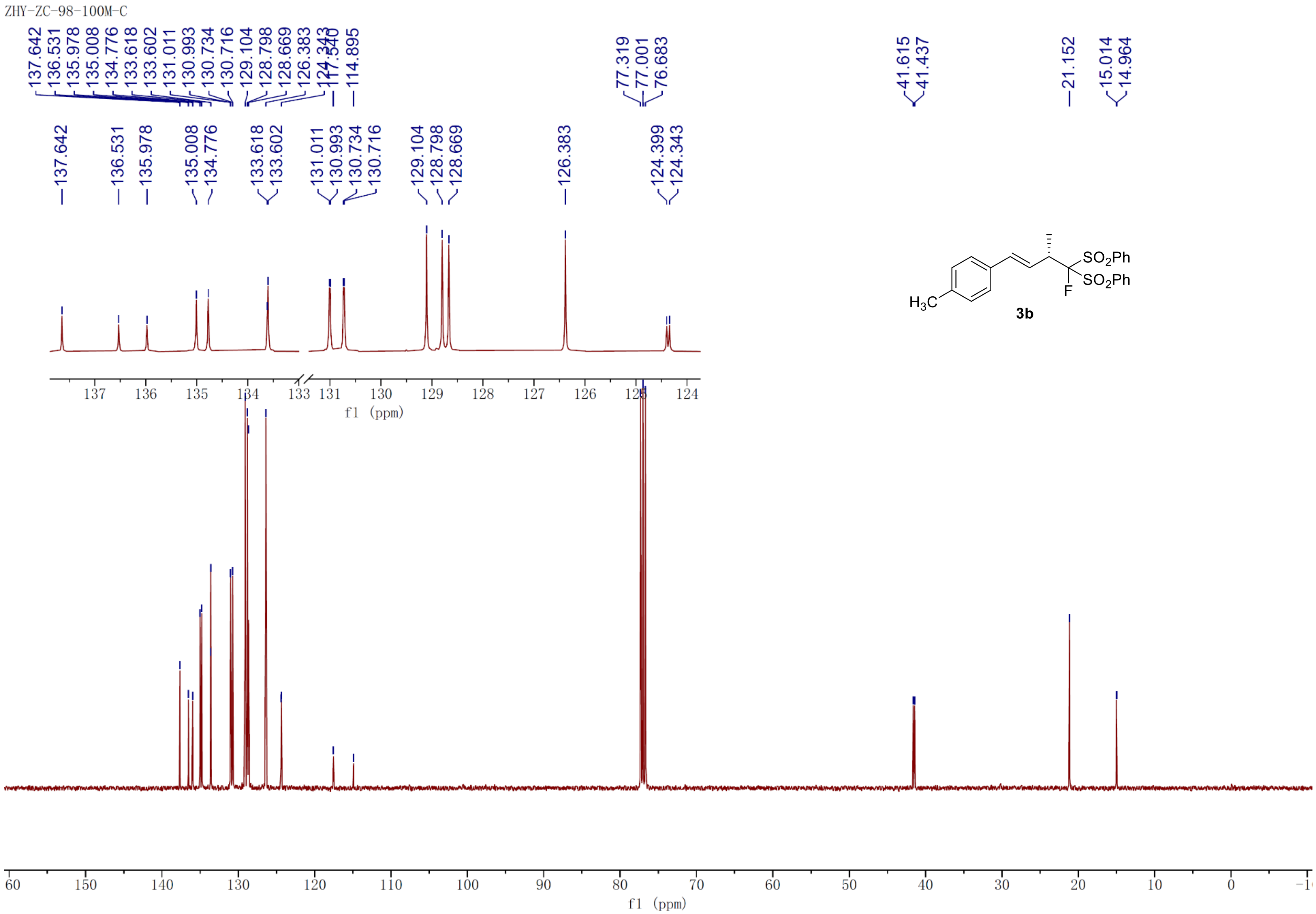
ZHY-ZC-98-100M-C
137.642
136.531
135.978
135.008
134.776
133.618
133.602
131.011
130.993
130.734
130.716
129.104
128.798
128.669
126.383
124.343
117.540
114.895
77.319
77.001
76.683
41.615
41.437
21.152
15.014
14.964
124.399
124.343
f1 (ppm)
SO_2Ph
SO_2Ph
F
H_3C
3b
f1 (ppm)

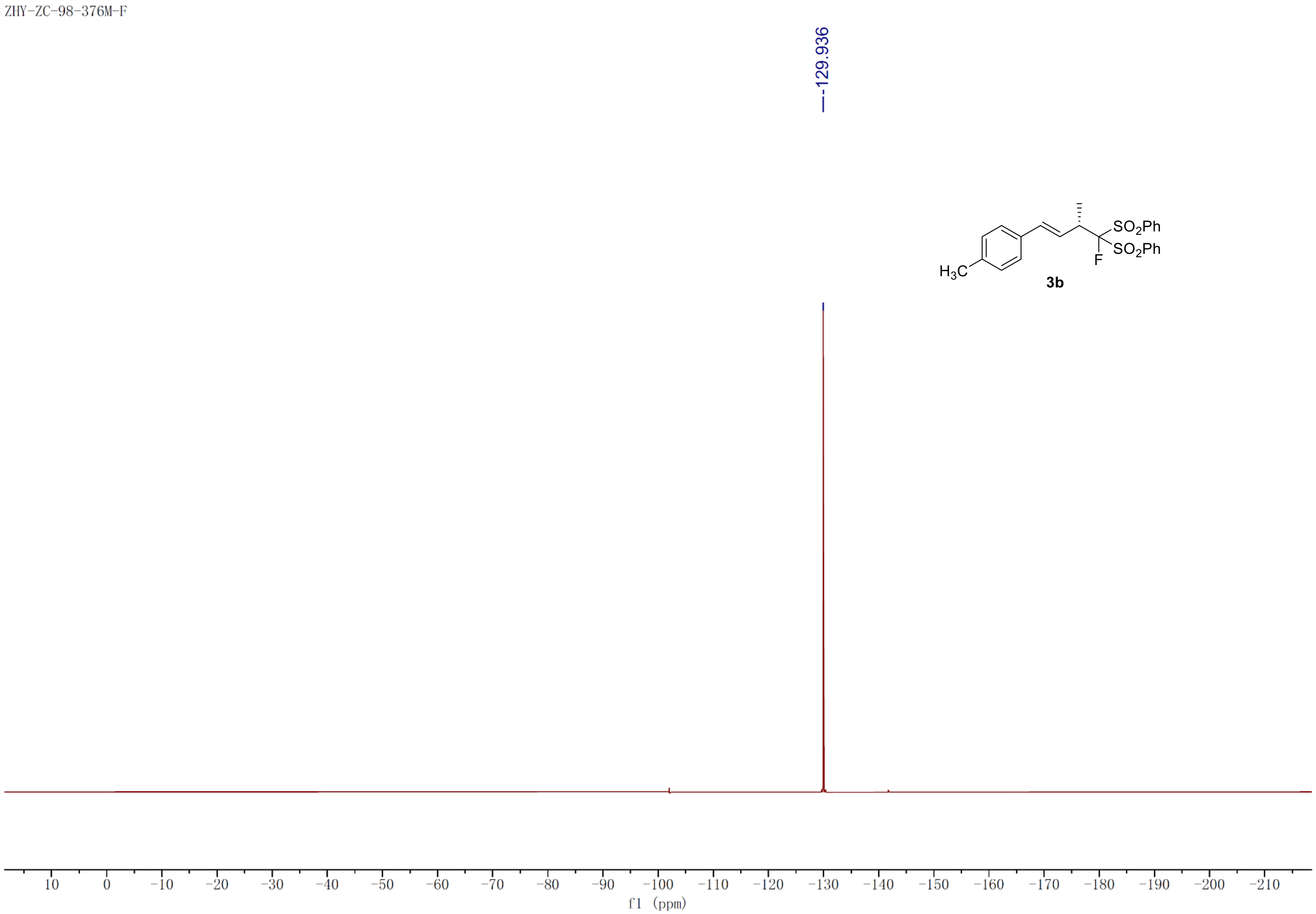
ZHY-ZC-98-376M-F
-129.936
H3C
SO2Ph
SO2Ph
F
3b
10 0 −10 −20 −30 −40 −50 −60 −70 −80 −90 −100 −110 −120 −130 −140 −150 −160 −170 −180 −190 −200 −210
f1 (ppm)

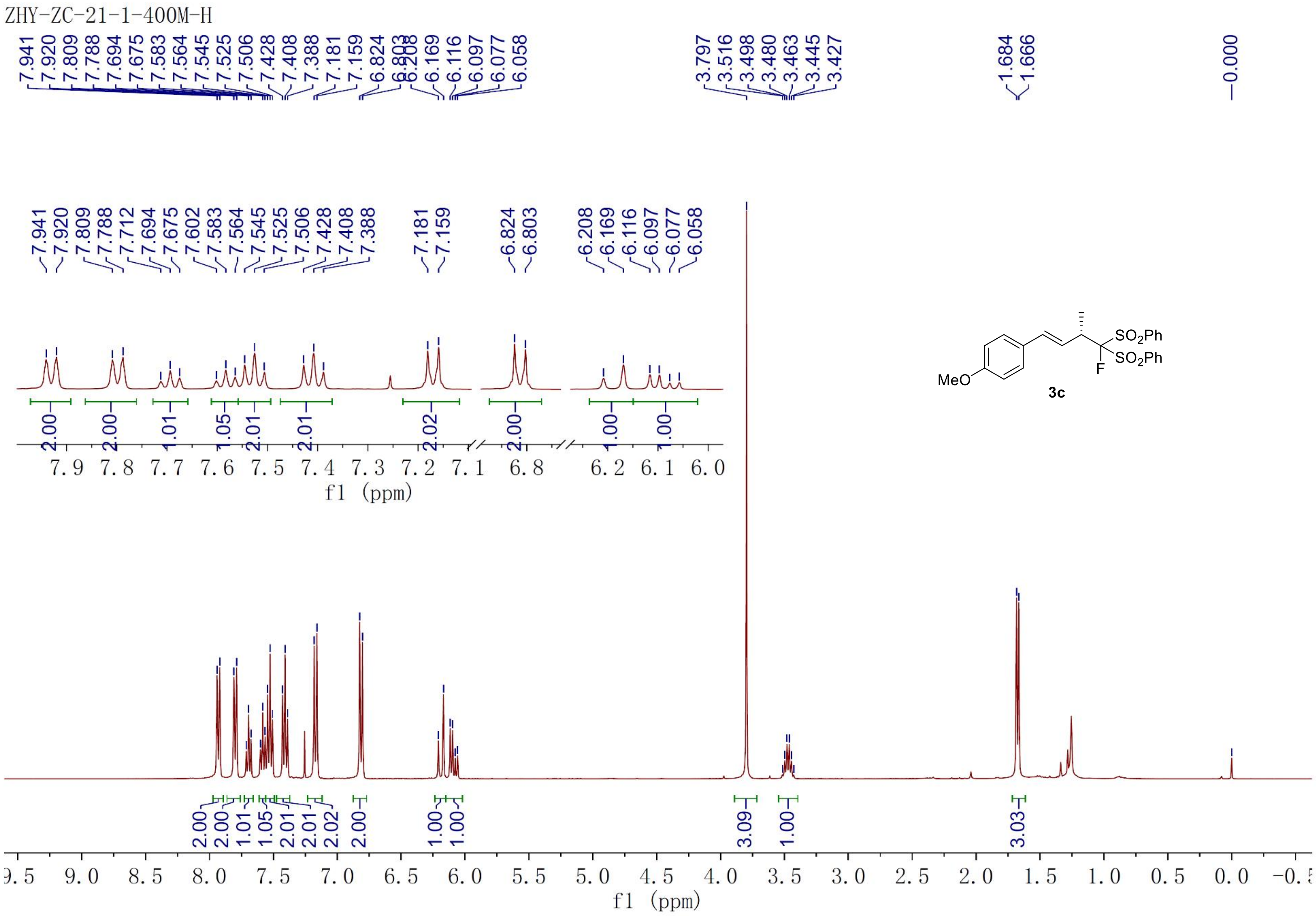
ZHY-ZC-21-1-400M-H
f1 (ppm)
SO2Ph
SO2Ph
F
MeO
3c

ZHY-ZC-21-1-100M-C

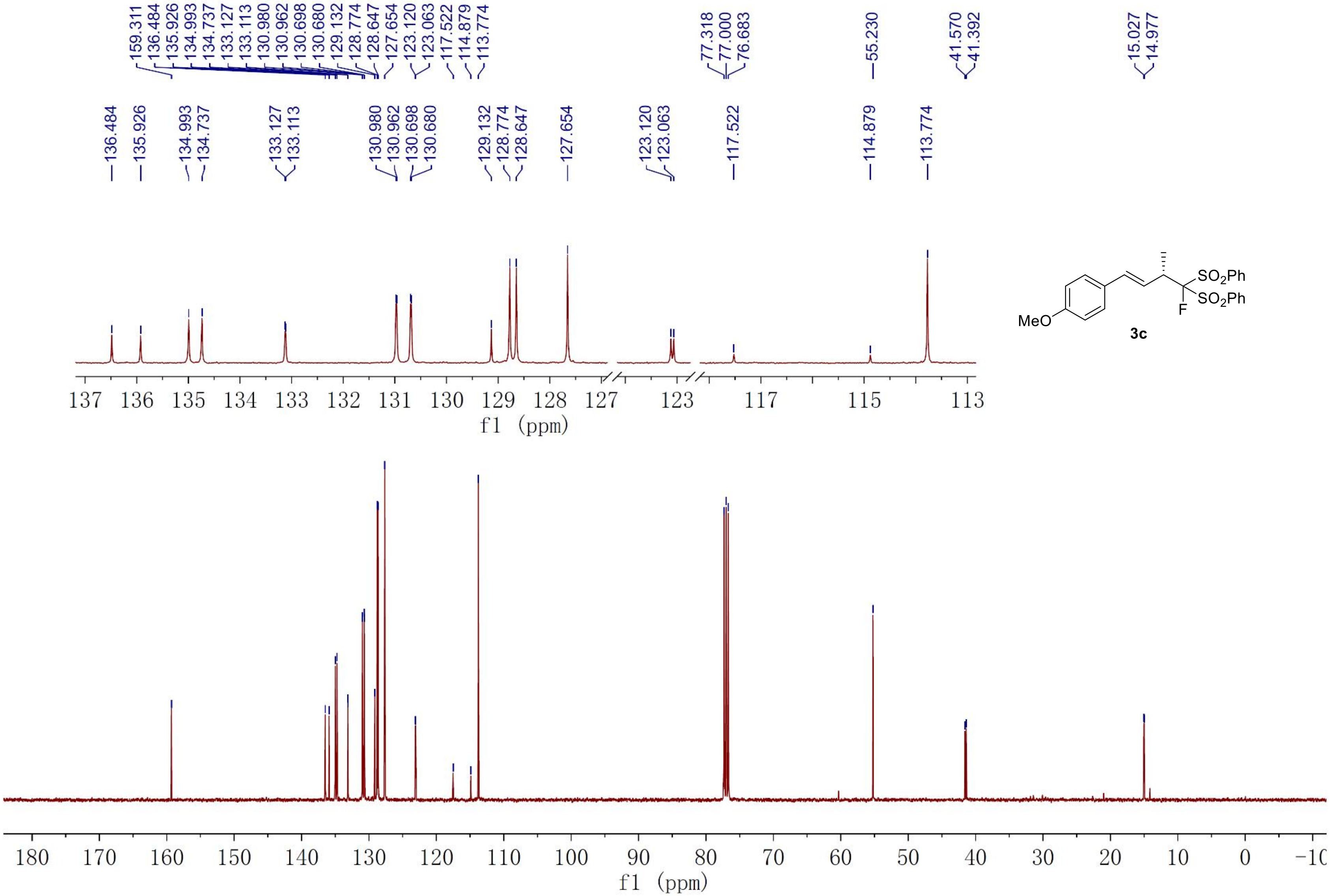

ZHY-ZC-21-1-376M-F

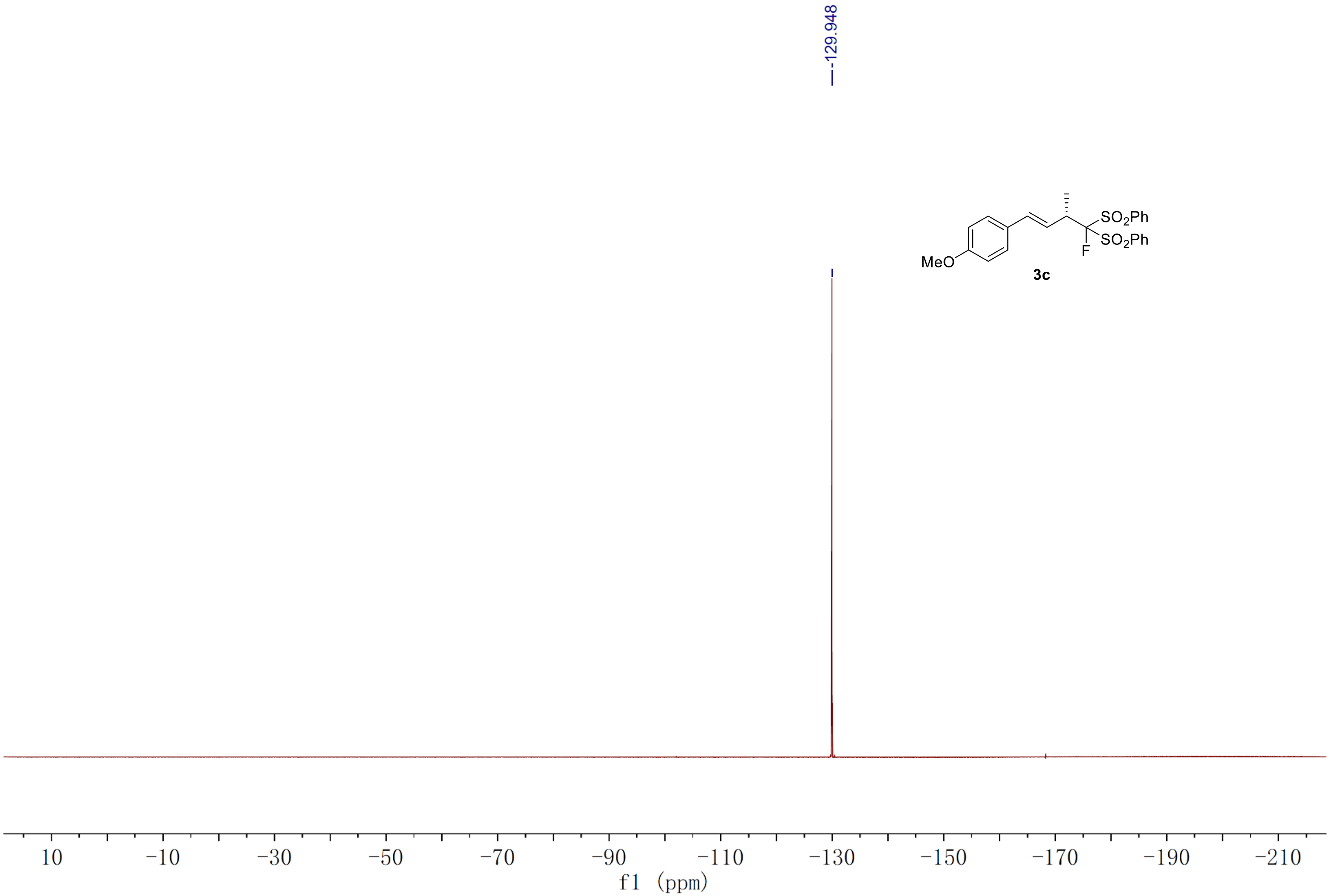

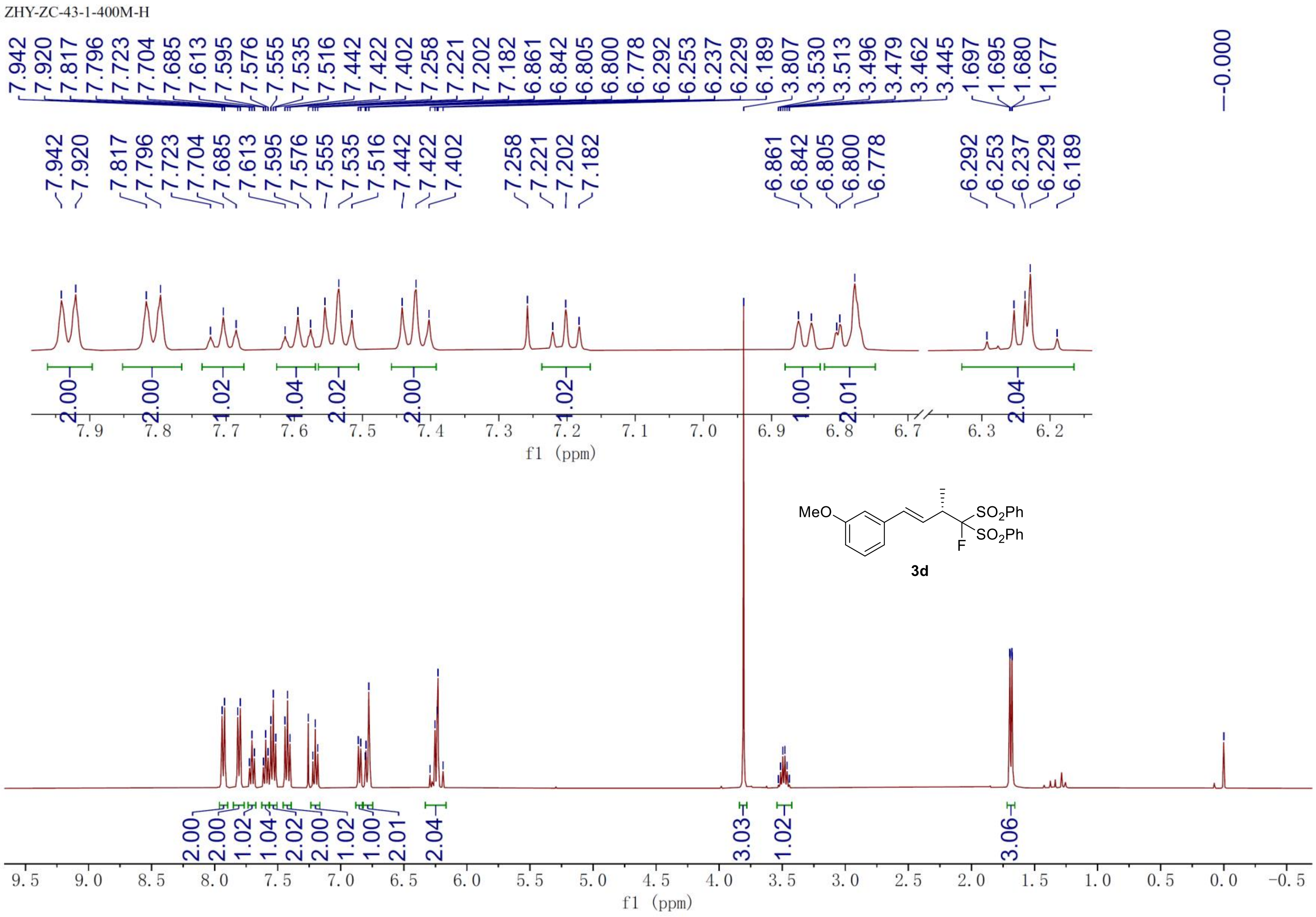
ZHY-ZC-43-1-400M-H
7.942 7.920 7.817 7.796 7.723 7.704 7.685 7.613 7.595 7.576 7.555 7.535 7.516 7.442 7.422 7.402 7.258 7.221 7.202 7.182 6.861 6.842 6.805 6.800 6.778 6.292 6.253 6.237 6.229 6.189 3.807 3.530 3.513 3.496 3.479 3.462 3.445 1.697 1.695 1.680 1.677
-0.000
2.00 2.00 1.02 1.04 2.02 2.00 1.02 1.00 2.01 2.04
f1 (ppm)
MeO
SO2Ph
SO2Ph
F
3d
2.00 2.00 1.02 1.04 2.02 2.00 1.02 1.00 2.01 2.04 3.03 1.02 3.06
f1 (ppm)

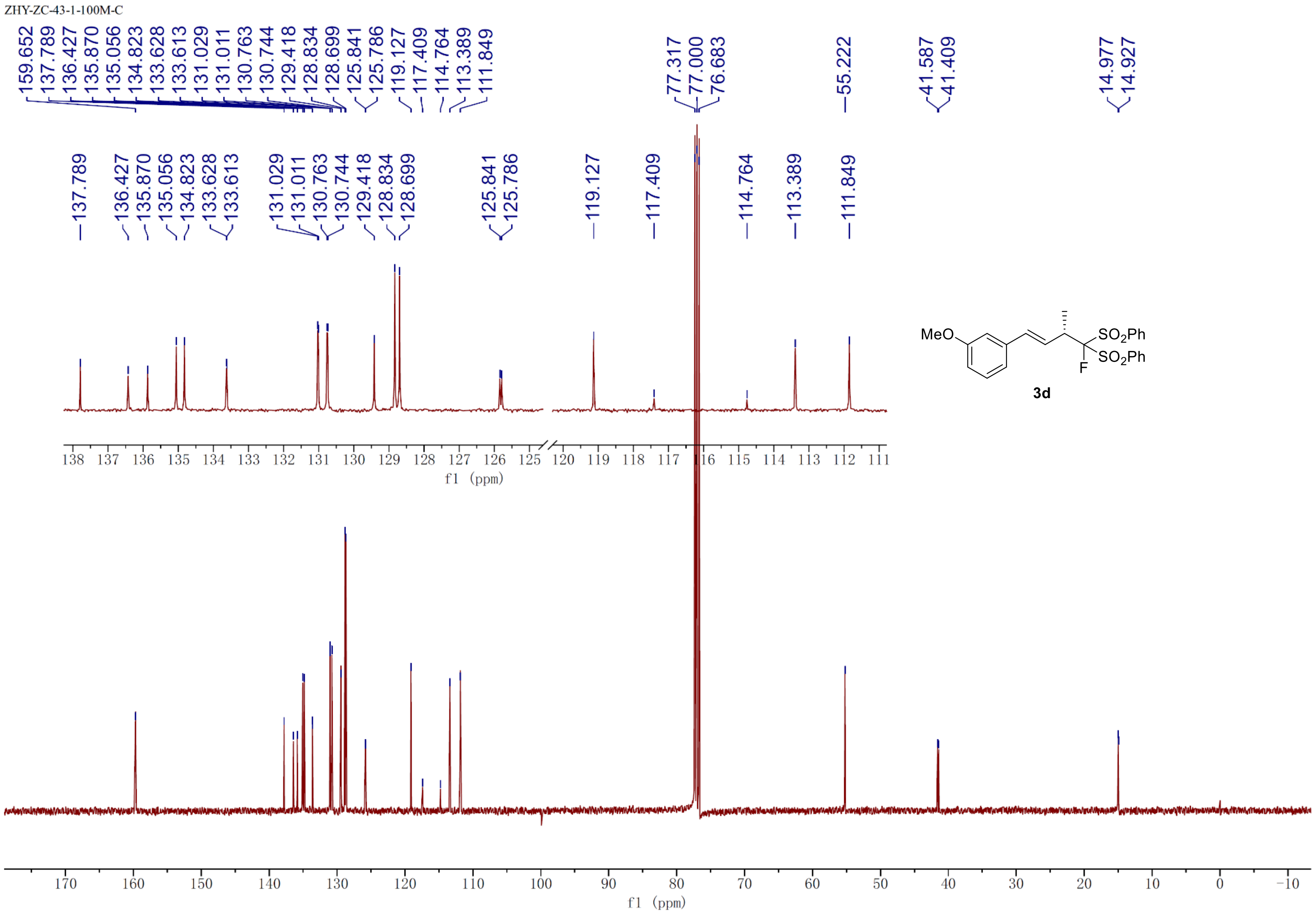
ZHY-ZC-43-1-100M-C
159.652
137.789
136.427
135.870
135.056
134.823
133.628
133.613
131.029
131.011
130.763
130.744
129.418
128.834
128.699
125.841
125.786
119.127
117.409
114.764
113.389
111.849
77.317
77.000
76.683
55.222
41.587
41.409
14.977
14.927
MeO
SO2Ph
SO2Ph
F
3d
f1 (ppm)
f1 (ppm)

ZHY-ZC-43-1-376M-F

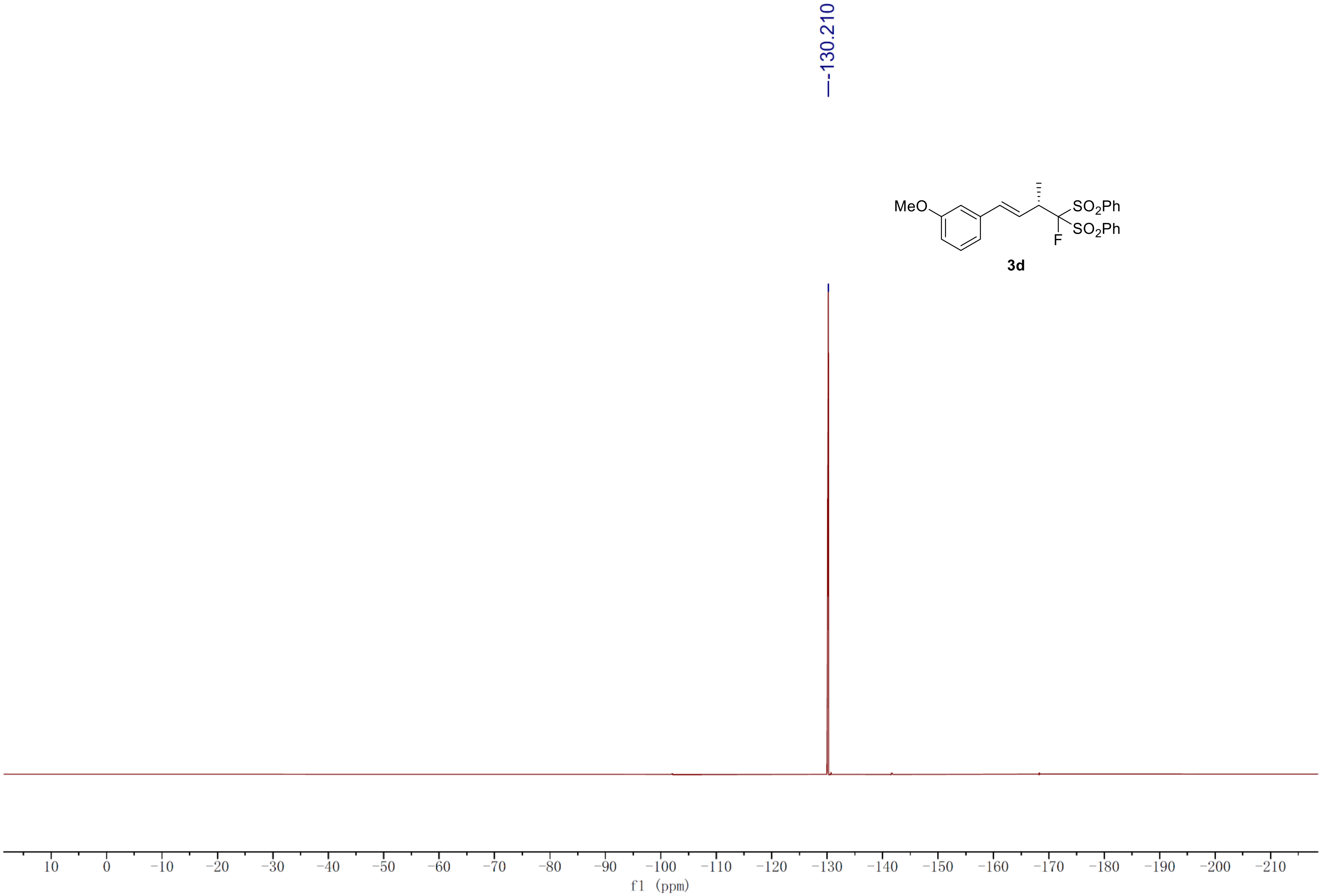

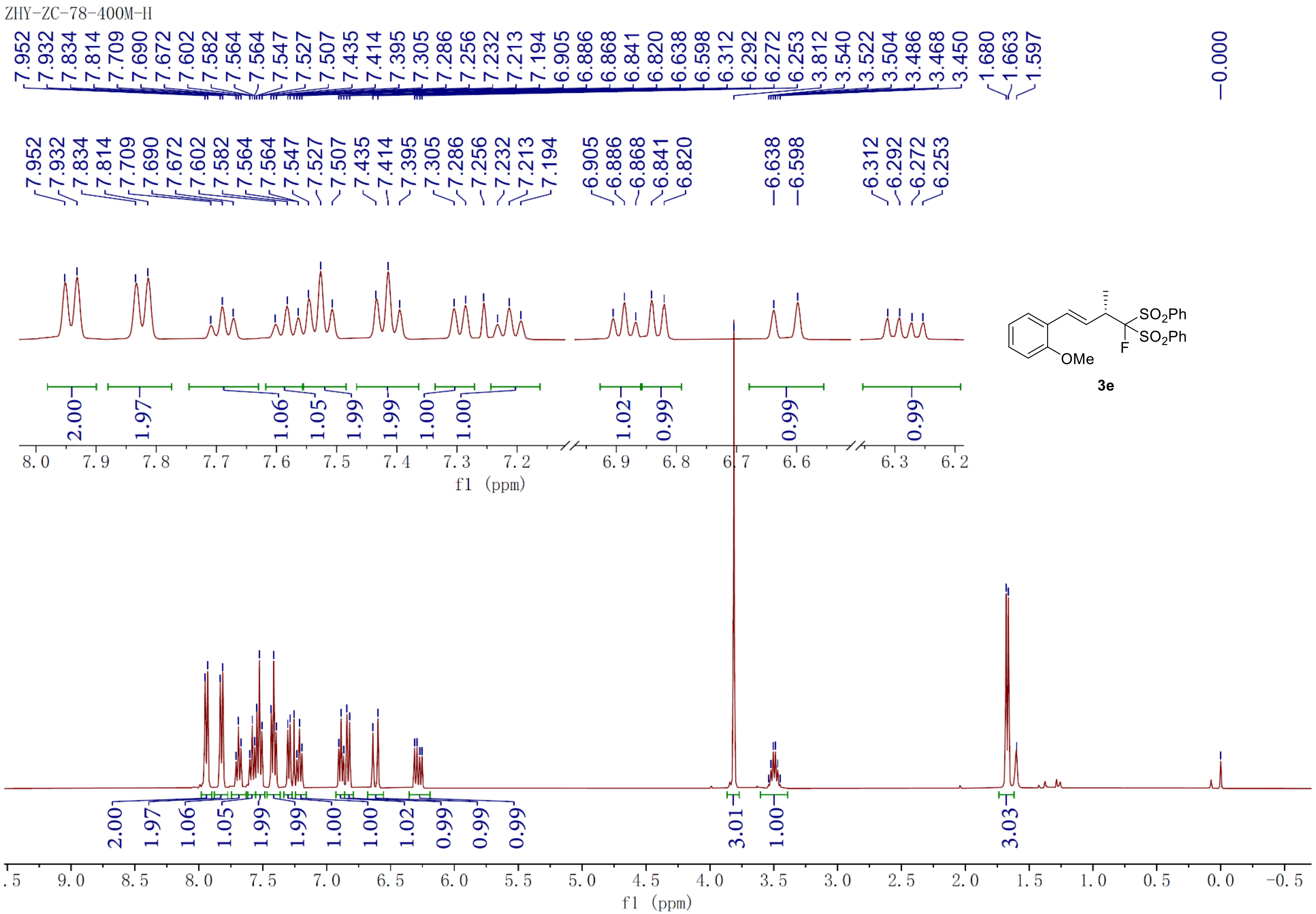

3e

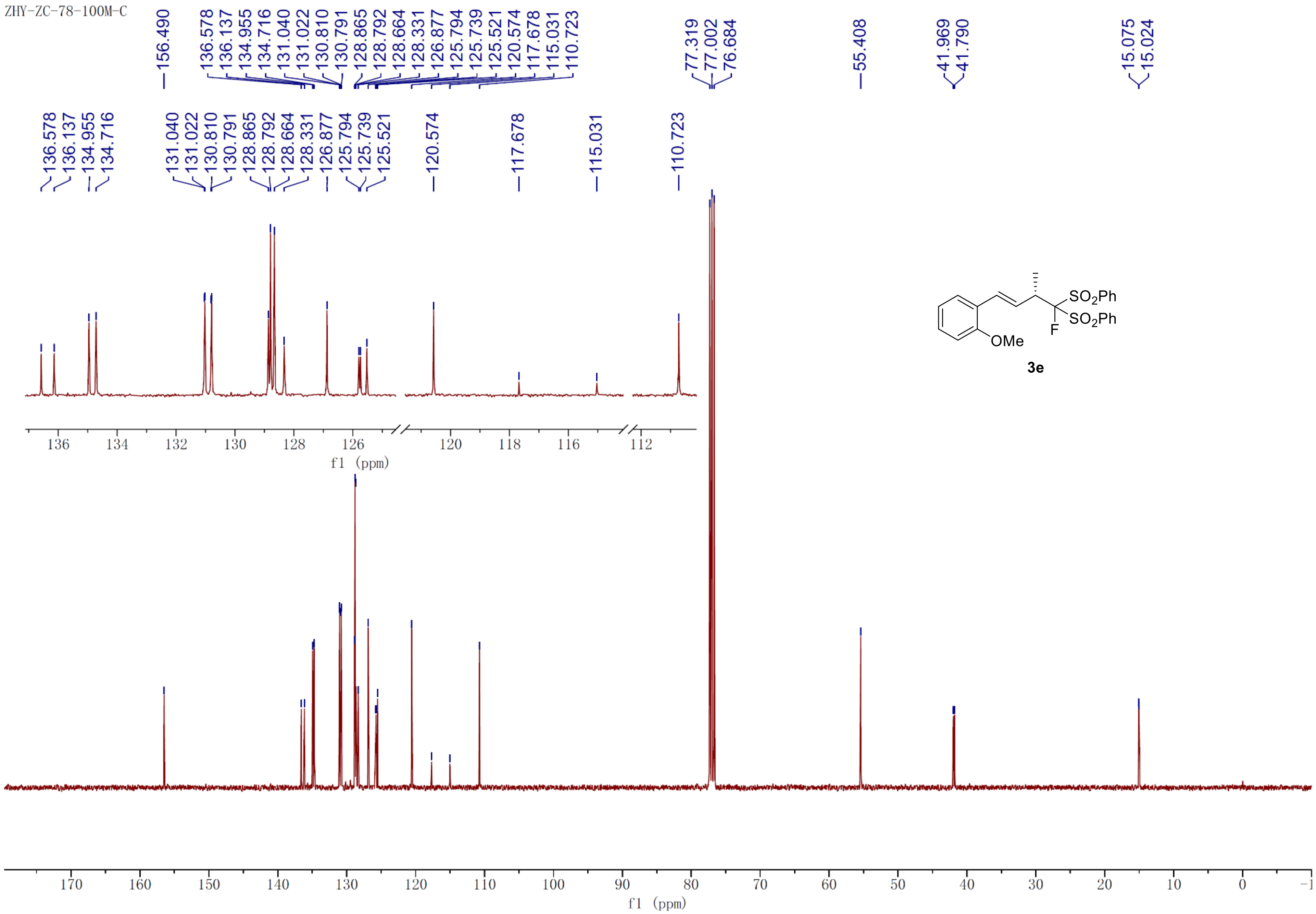
ZHY-ZC-78-100M-C
156.490
136.578
136.137
134.955
134.716
131.040
131.022
130.810
130.791
128.865
128.792
128.664
128.331
126.877
125.794
125.739
125.521
120.574
117.678
115.031
110.723
77.319
77.002
76.684
55.408
41.969
41.790
15.075
15.024
SO2Ph
SO2Ph
F
OMe
3e
f1 (ppm)

ZHY-ZC-78-376M-F

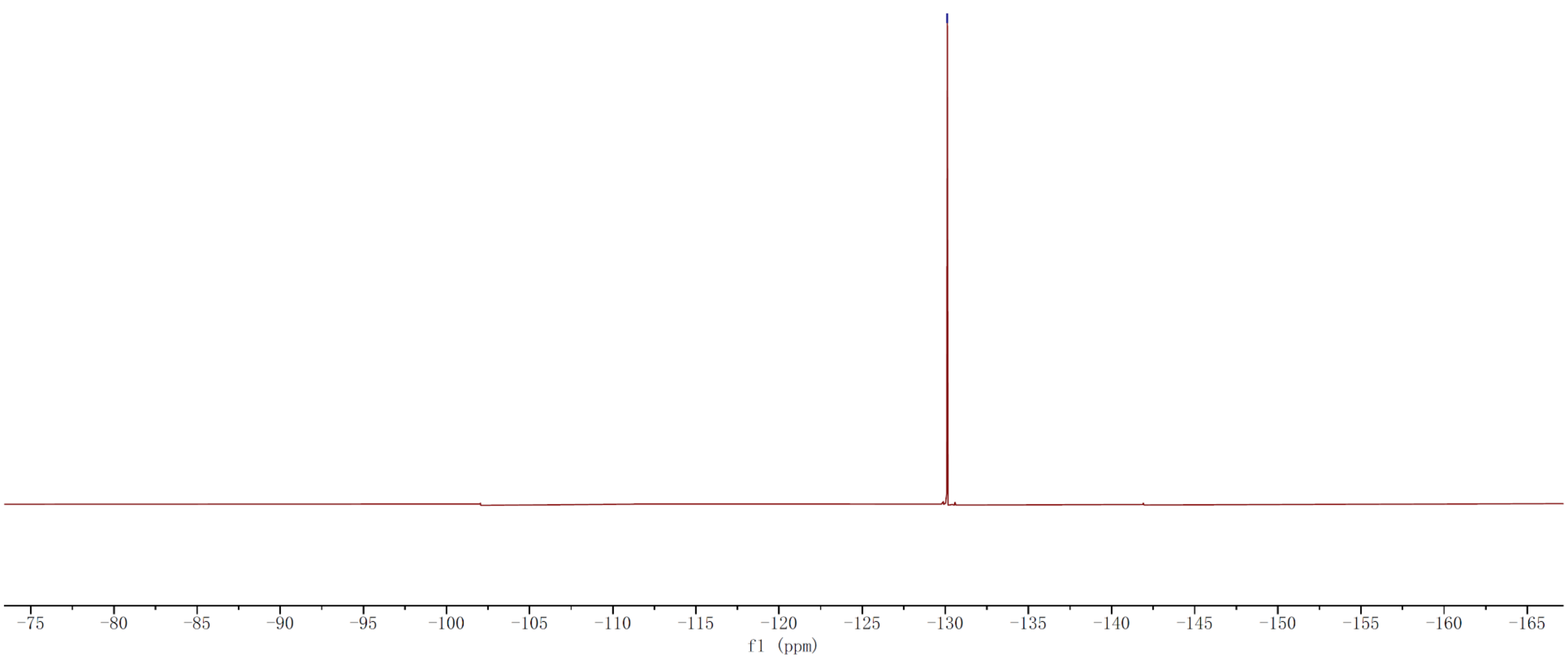

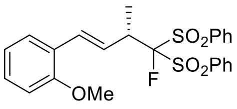

3e

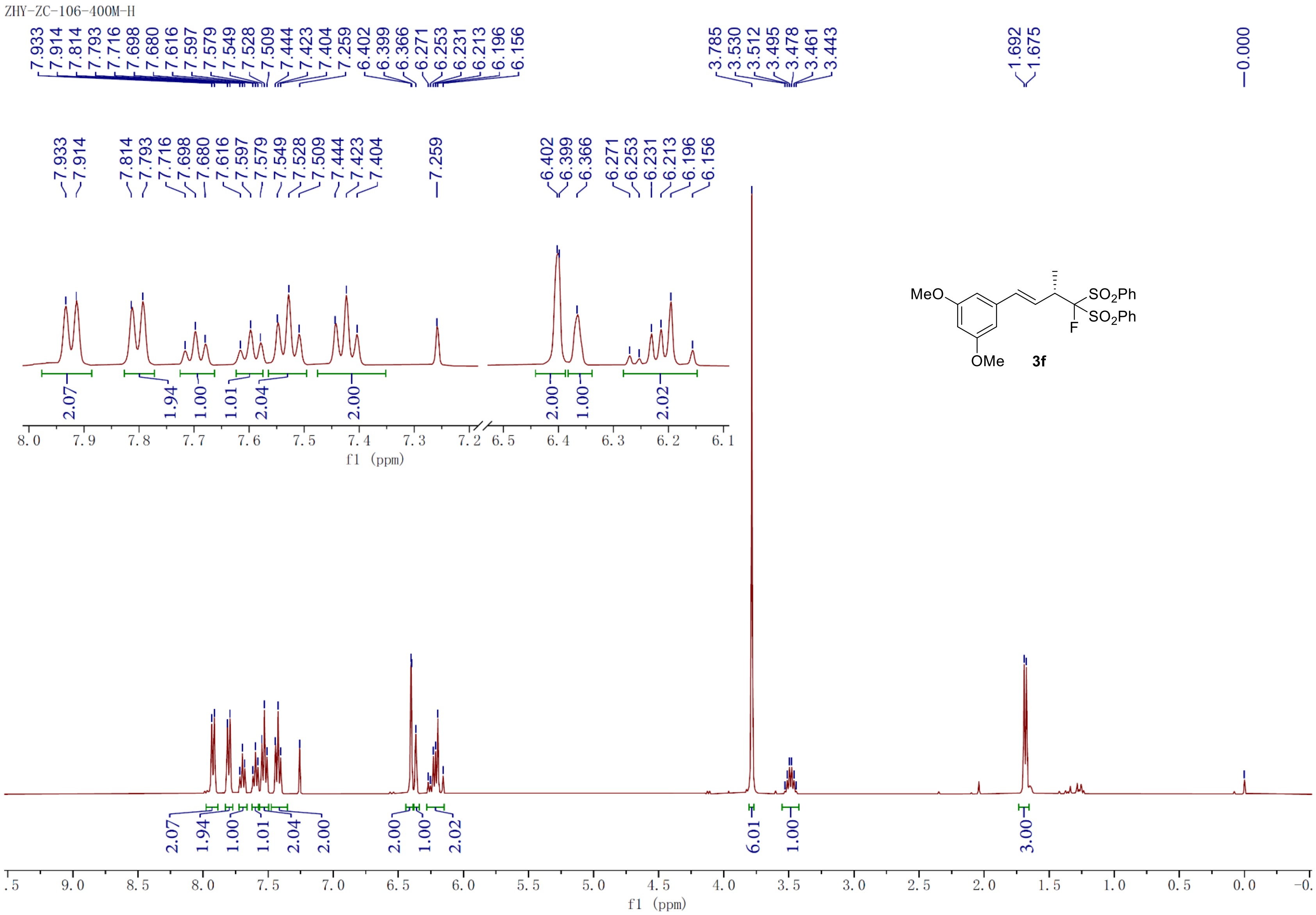
ZHY-ZC-106-400M-H
7.933 7.914 7.814 7.793 7.716 7.698 7.680 7.616 7.597 7.579 7.549 7.528 7.509 7.444 7.423 7.404 7.259 6.402 6.399 6.366 6.271 6.253 6.231 6.213 6.196 6.156
3.785 3.530 3.512 3.495 3.478 3.461 3.443
1.692 1.675
0.000
MeO
SO2Ph
SO2Ph
F
OMe
3f
2.07 1.94 1.00 1.01 2.04 2.00 2.00 1.00 2.02
6.01 1.00 3.00
f1 (ppm)

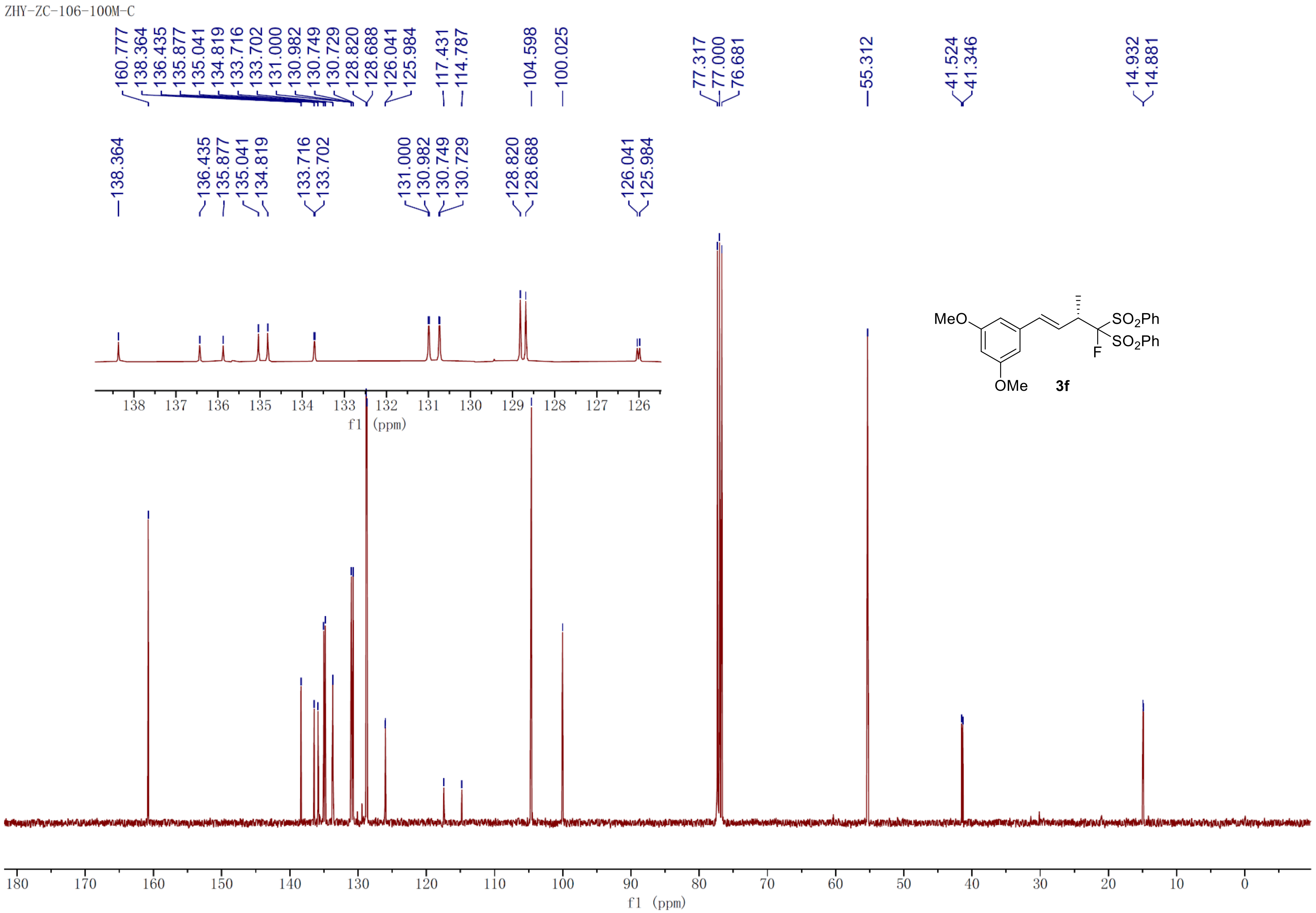
ZHY-ZC-106-100M-C
160.777
138.364
136.435
135.877
135.041
134.819
133.716
133.702
131.000
130.982
130.749
130.729
128.820
128.688
126.041
125.984
117.431
114.787
104.598
100.025
77.317
77.000
76.681
55.312
41.524
41.346
14.932
14.881
MeO
OMe
SO2Ph
SO2Ph
F
3f
f1 (ppm)

ZHY-ZC-106-376M-F

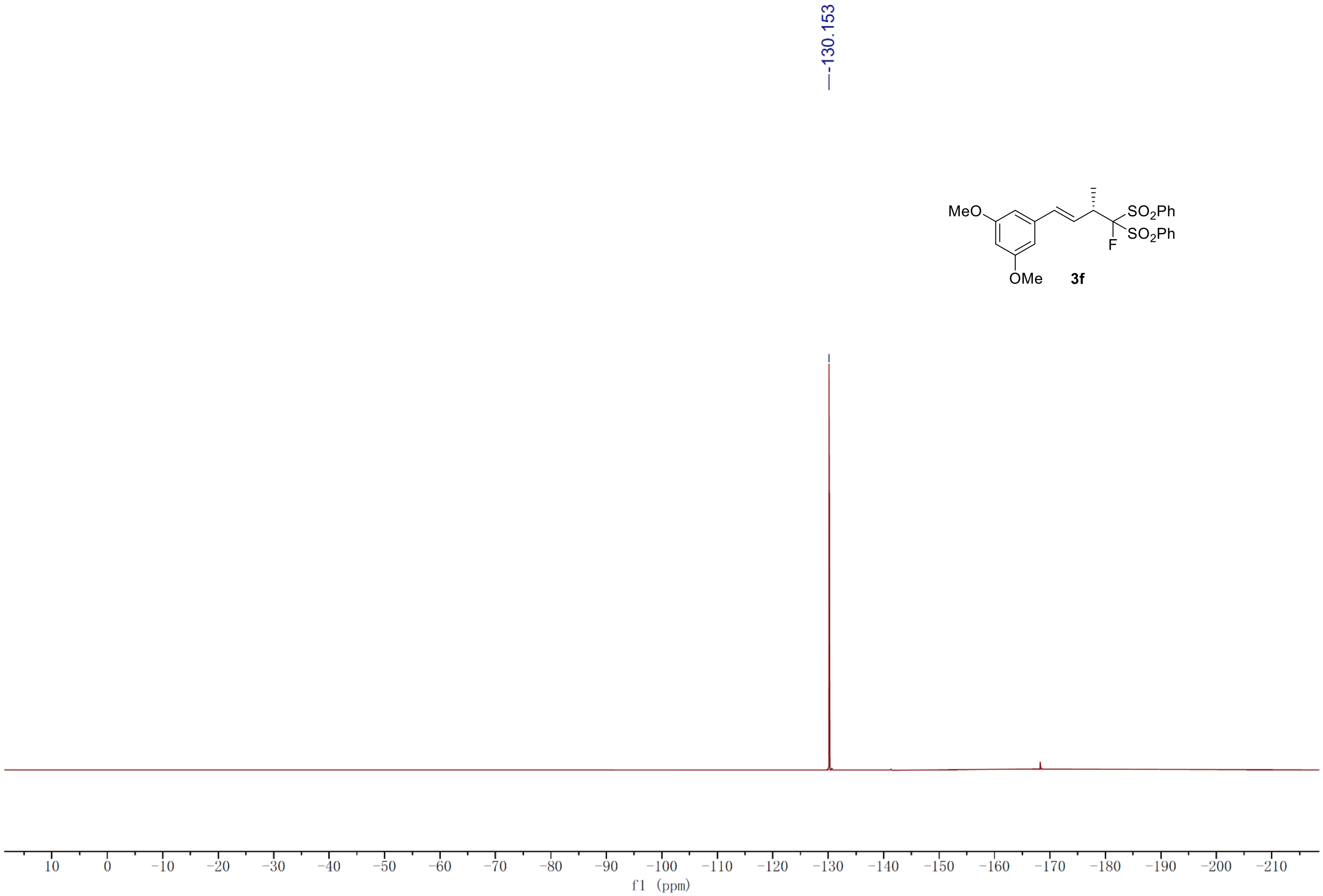

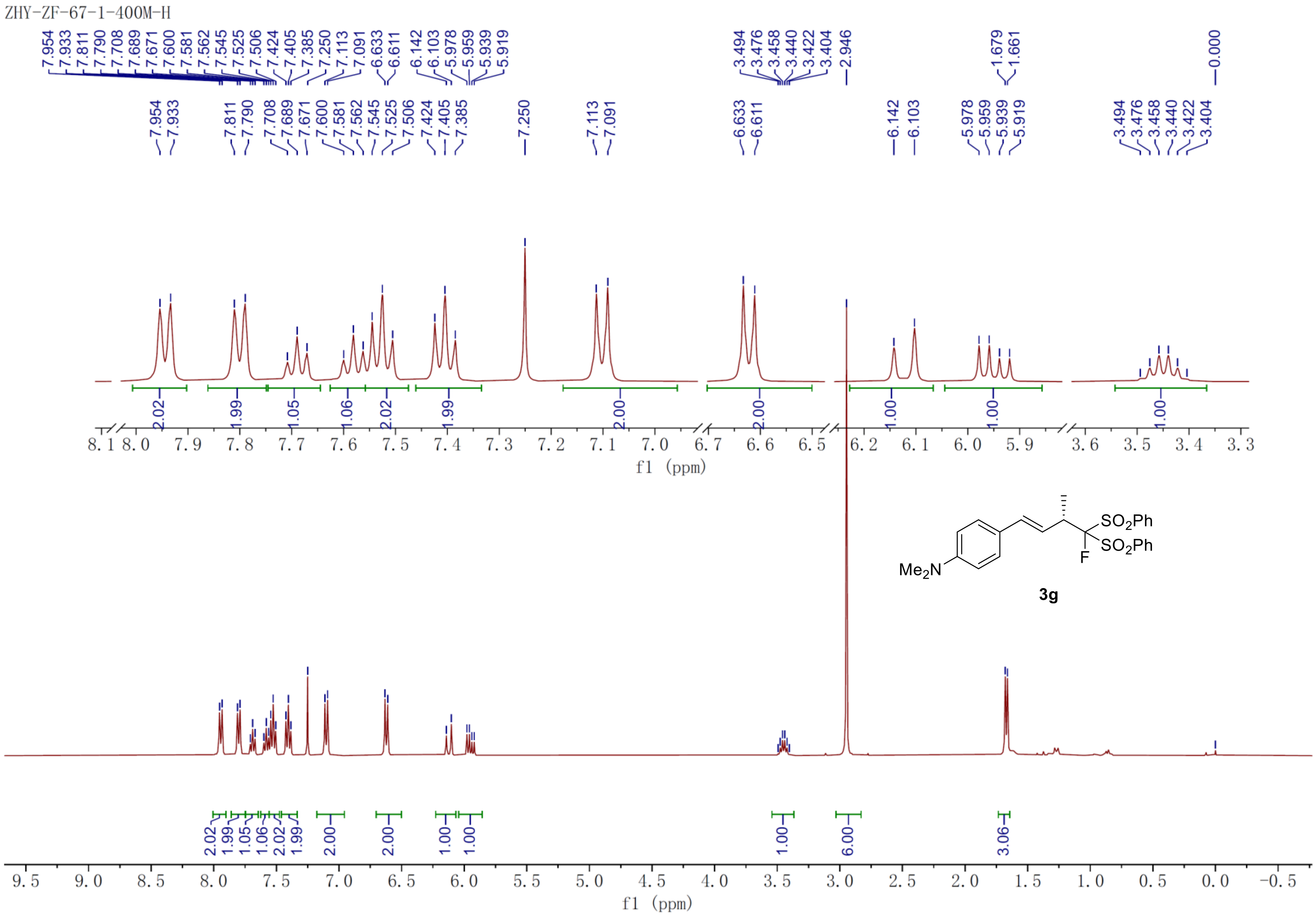
ZHY-ZF-67-1-400M-H
7.954 7.933 7.811 7.790 7.708 7.689 7.671 7.600 7.581 7.562 7.545 7.525 7.506 7.424 7.405 7.385 7.250 7.113 7.091 6.633 6.611 6.142 6.103 5.978 5.959 5.939 5.919
3.494 3.476 3.458 3.440 3.422 3.404 2.946
1.679 1.661
0.000
SO2Ph
SO2Ph
F
Me2N
3g
f1 (ppm)

ZHY-ZF-67-1-100M-C

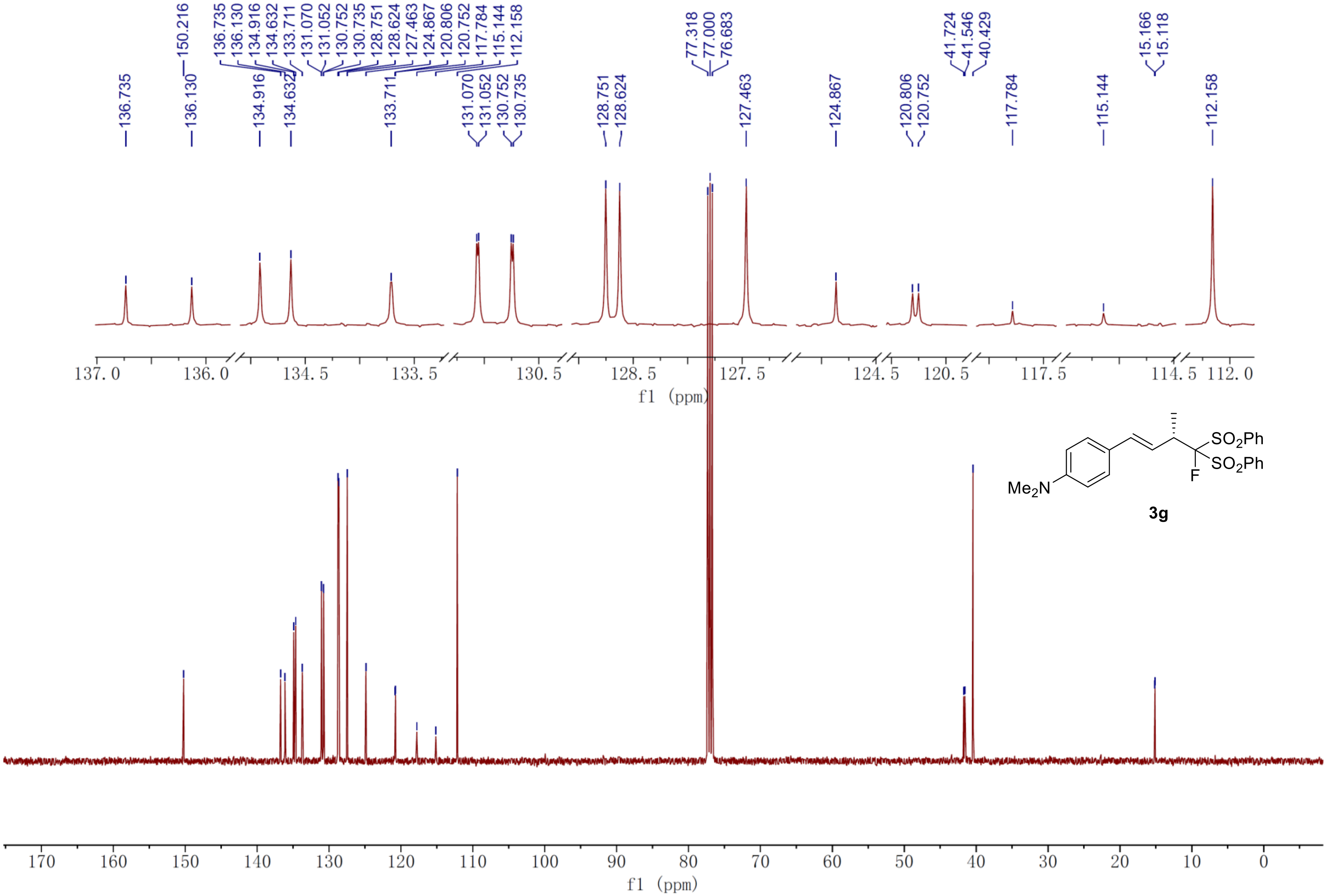

ZHY-ZF-67-1-376M-F

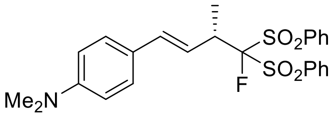

3g

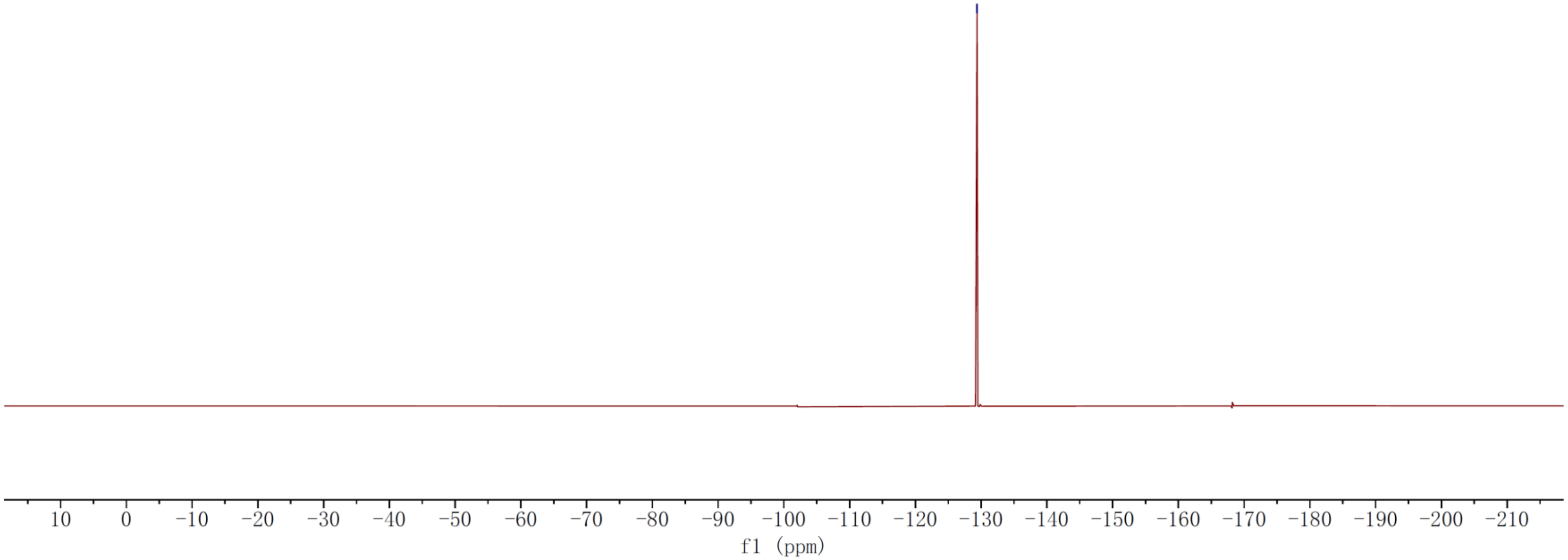

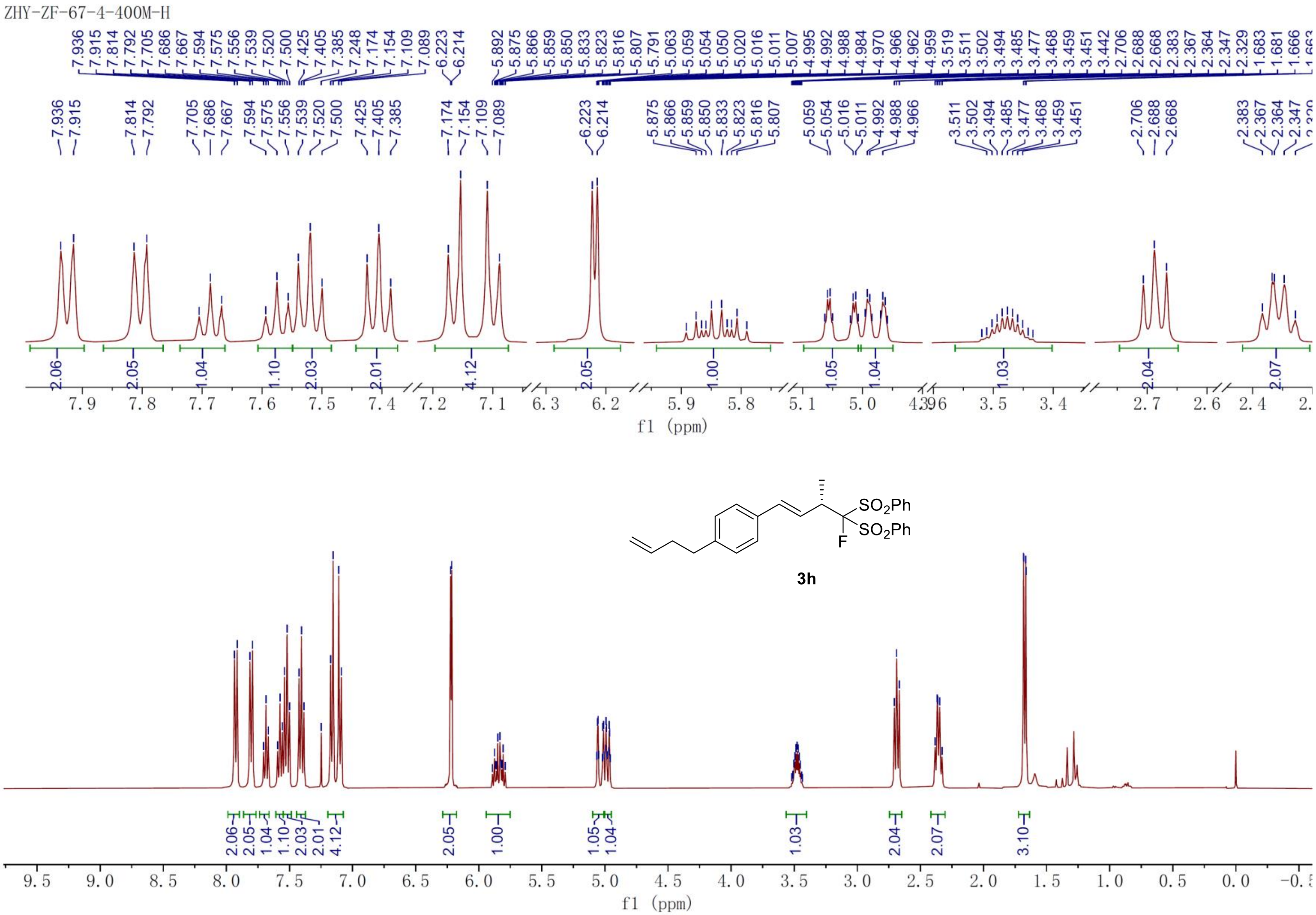

3h

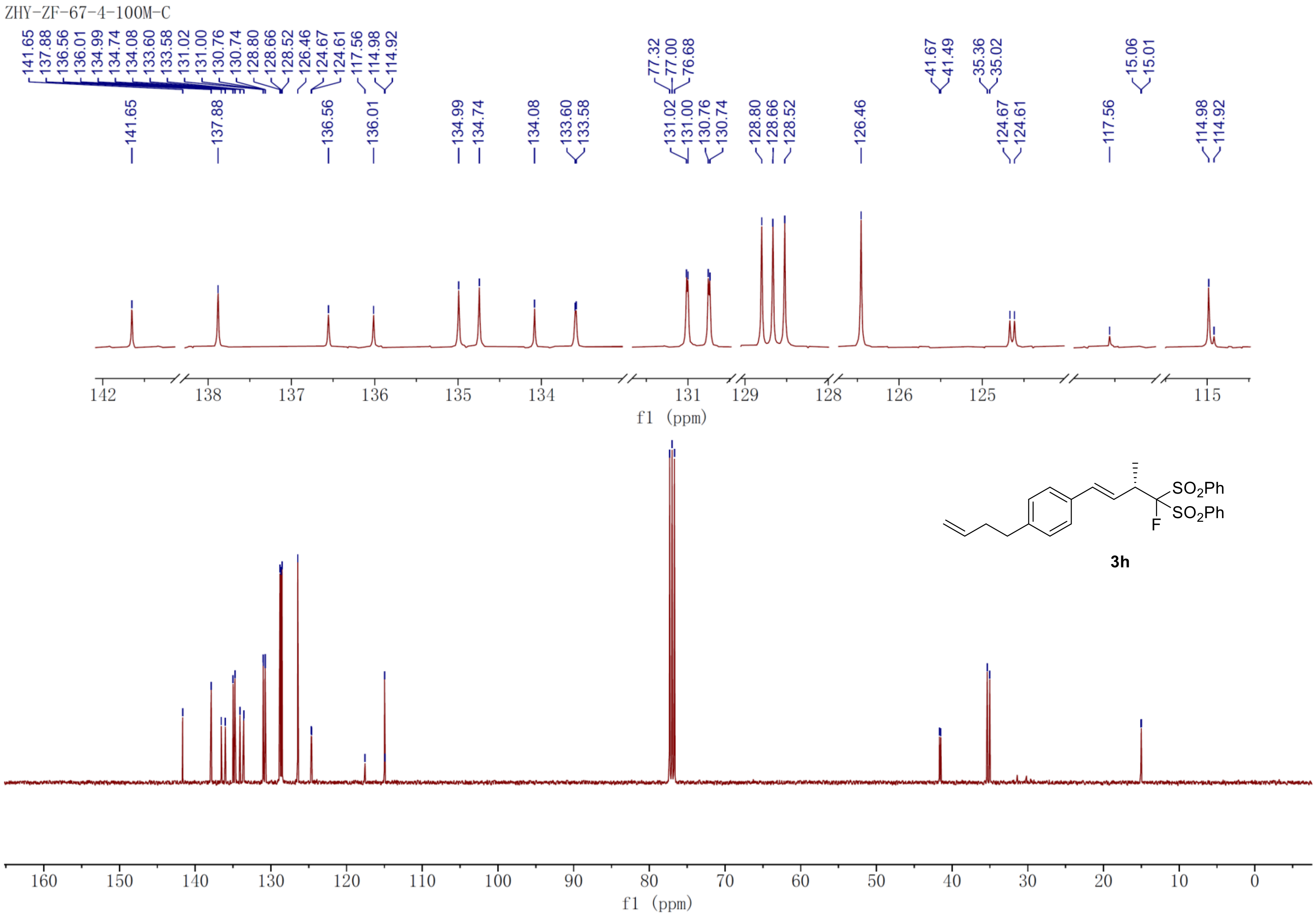
ZHY-ZF-67-4-100M-C
141.65
137.88
136.56
136.01
134.99
134.74
134.08
133.60
133.58
131.02
131.00
130.76
130.74
128.80
128.66
128.52
126.46
124.67
124.61
117.56
114.98
114.92
77.32
77.00
76.68
41.67
41.49
35.36
35.02
15.06
15.01
f1 (ppm)
f1 (ppm)
SO2Ph
SO2Ph
F
3h

ZHY-ZF-67-4-376M-F

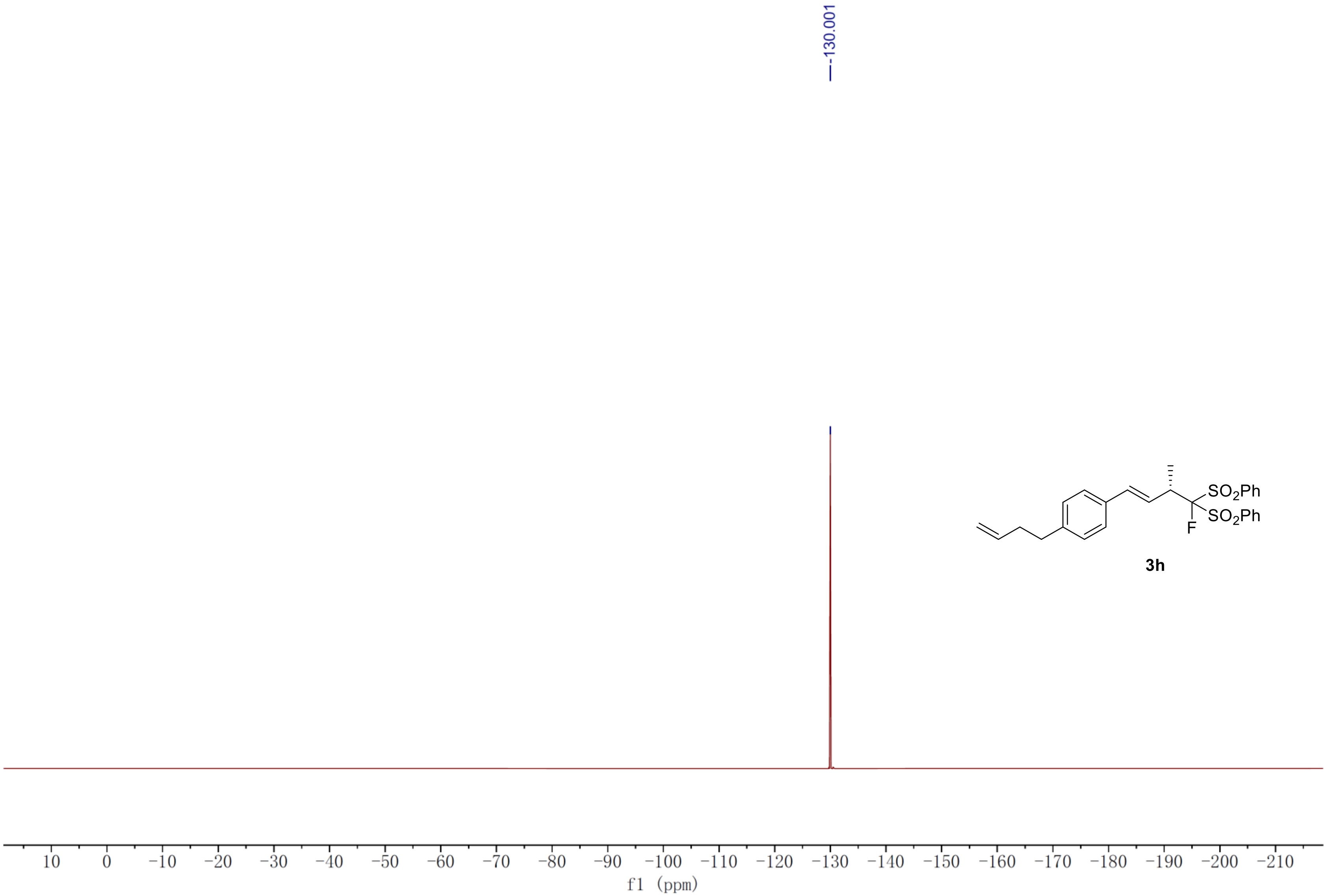

3h

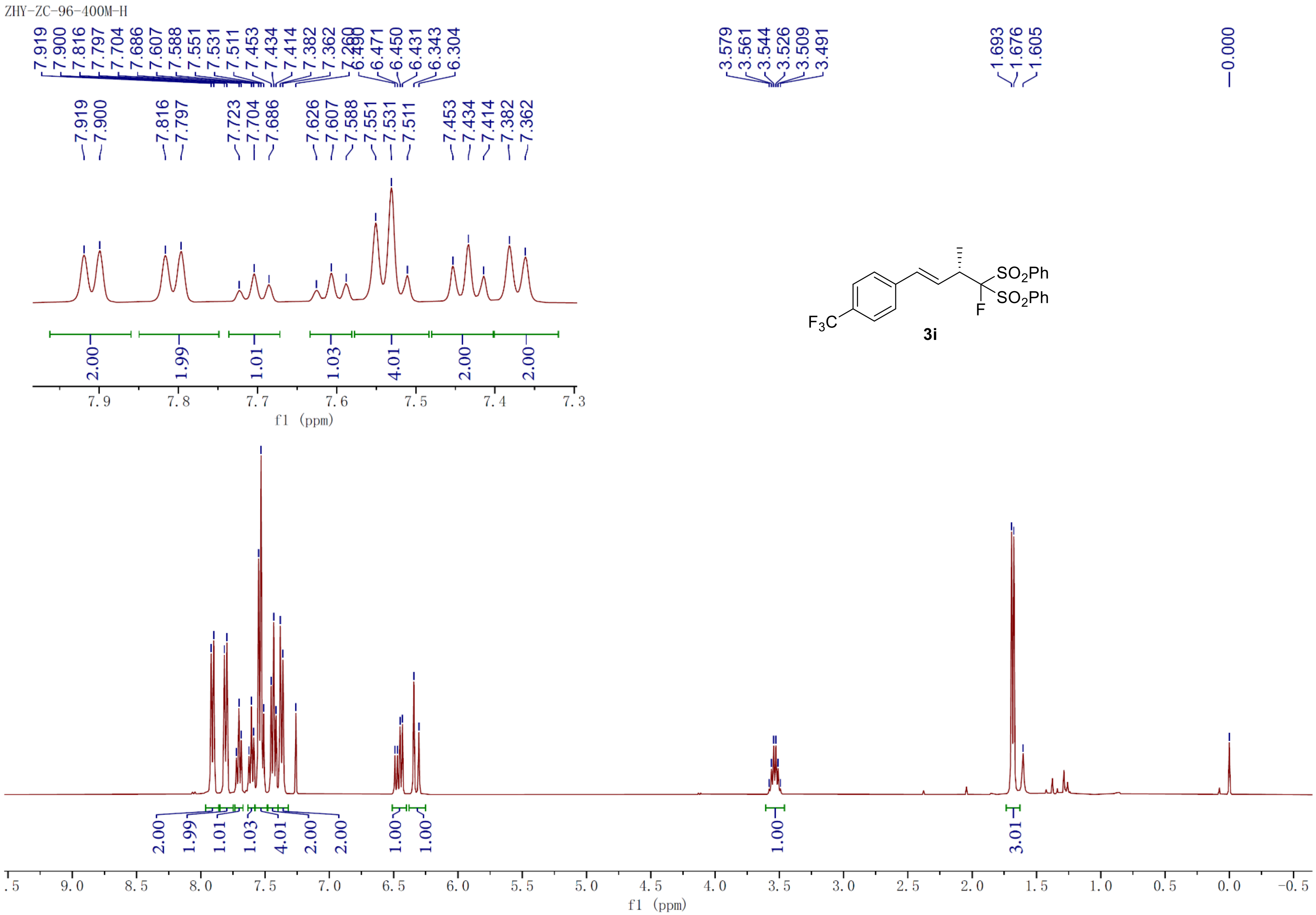
ZHY-ZC-96-400M-H
7.919
7.900
7.816
7.797
7.704
7.686
7.607
7.588
7.551
7.531
7.511
7.453
7.434
7.414
7.382
7.362
7.260
6.490
6.471
6.450
6.431
6.343
6.304
3.579
3.561
3.544
3.526
3.509
3.491
1.693
1.676
1.605
0.000
7.723
7.626
SO2Ph
SO2Ph
F
F3C
3i
2.00
1.99
1.01
1.03
4.01
2.00
2.00
1.00
1.00
1.00
3.01
f1 (ppm)

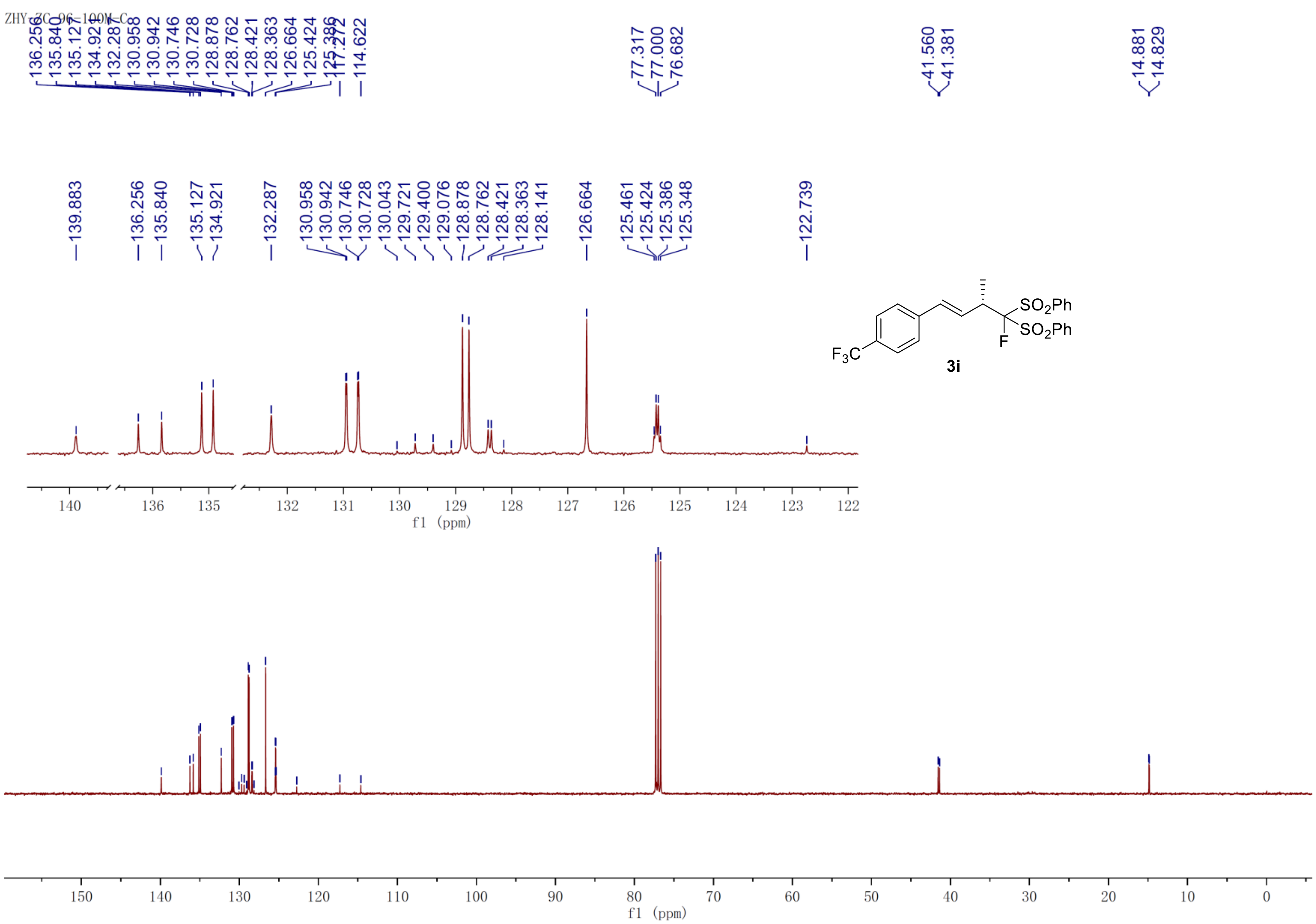
ZHY-ZC-96-100M-C
F$_3$C
SO$_2$Ph
SO$_2$Ph
F
3i
f1 (ppm)
f1 (ppm)

ZHY-ZC-96-376M-F

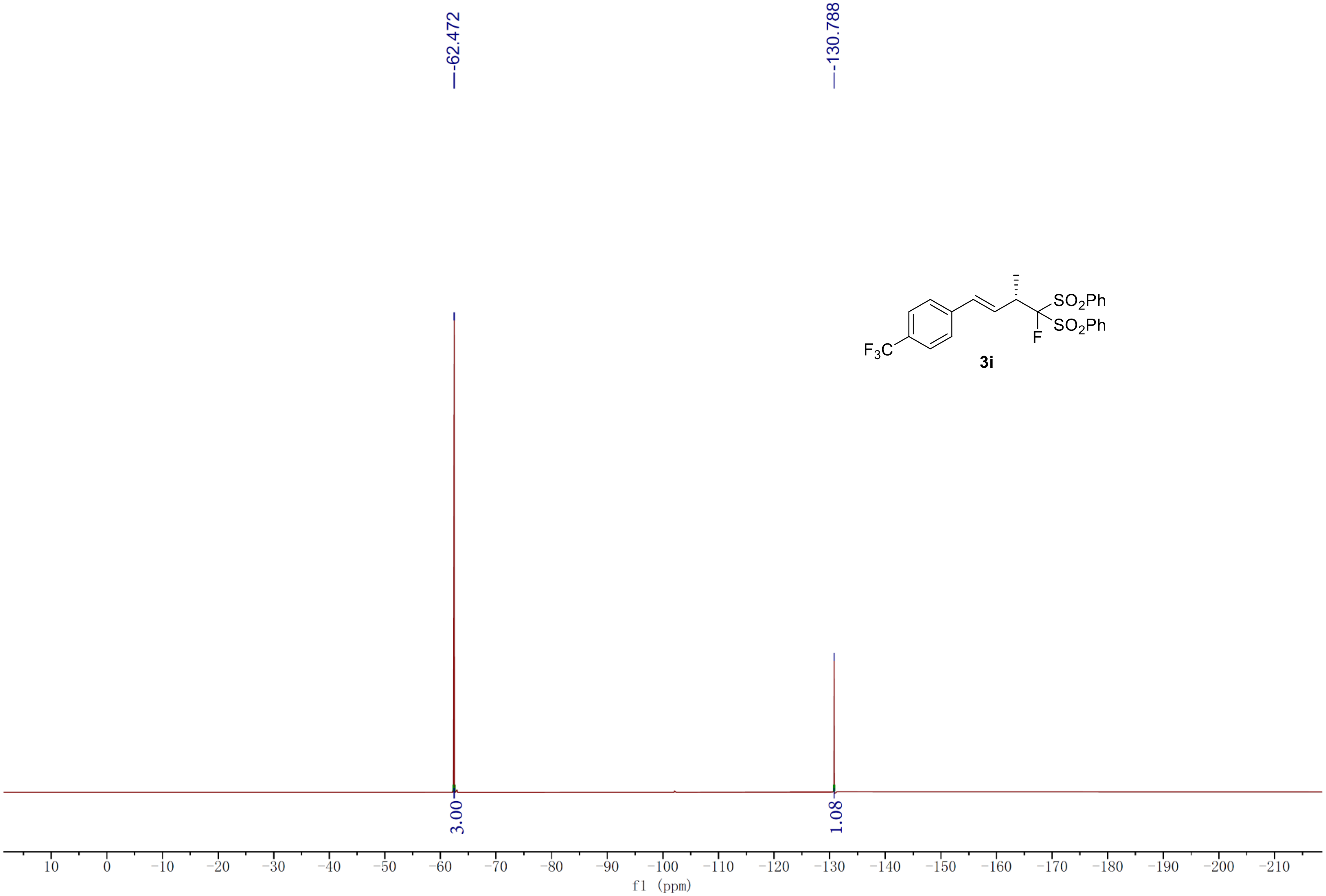

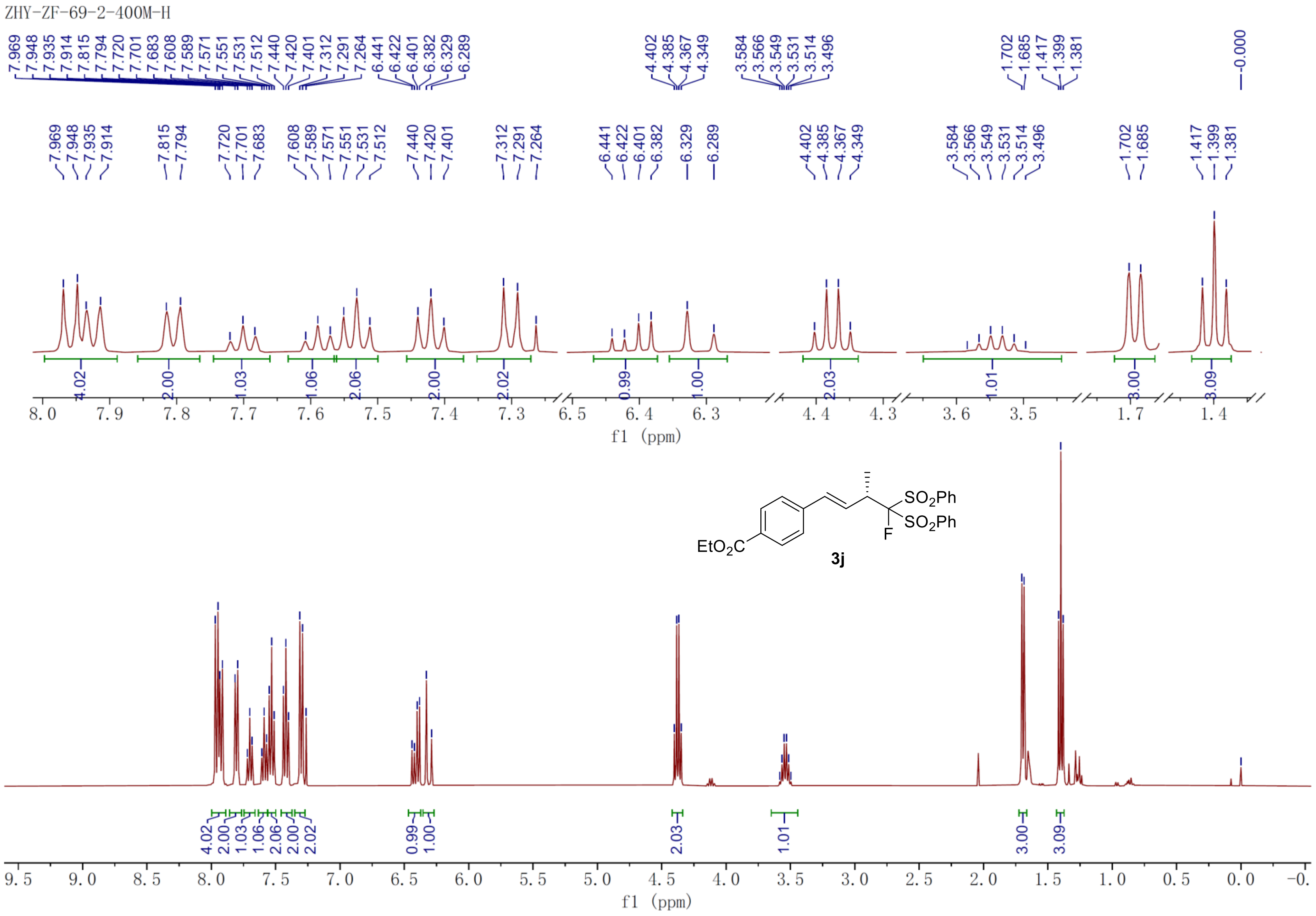
ZHY-ZF-69-2-400M-H
EtO2C
SO2Ph
SO2Ph
F
3j
f1 (ppm)

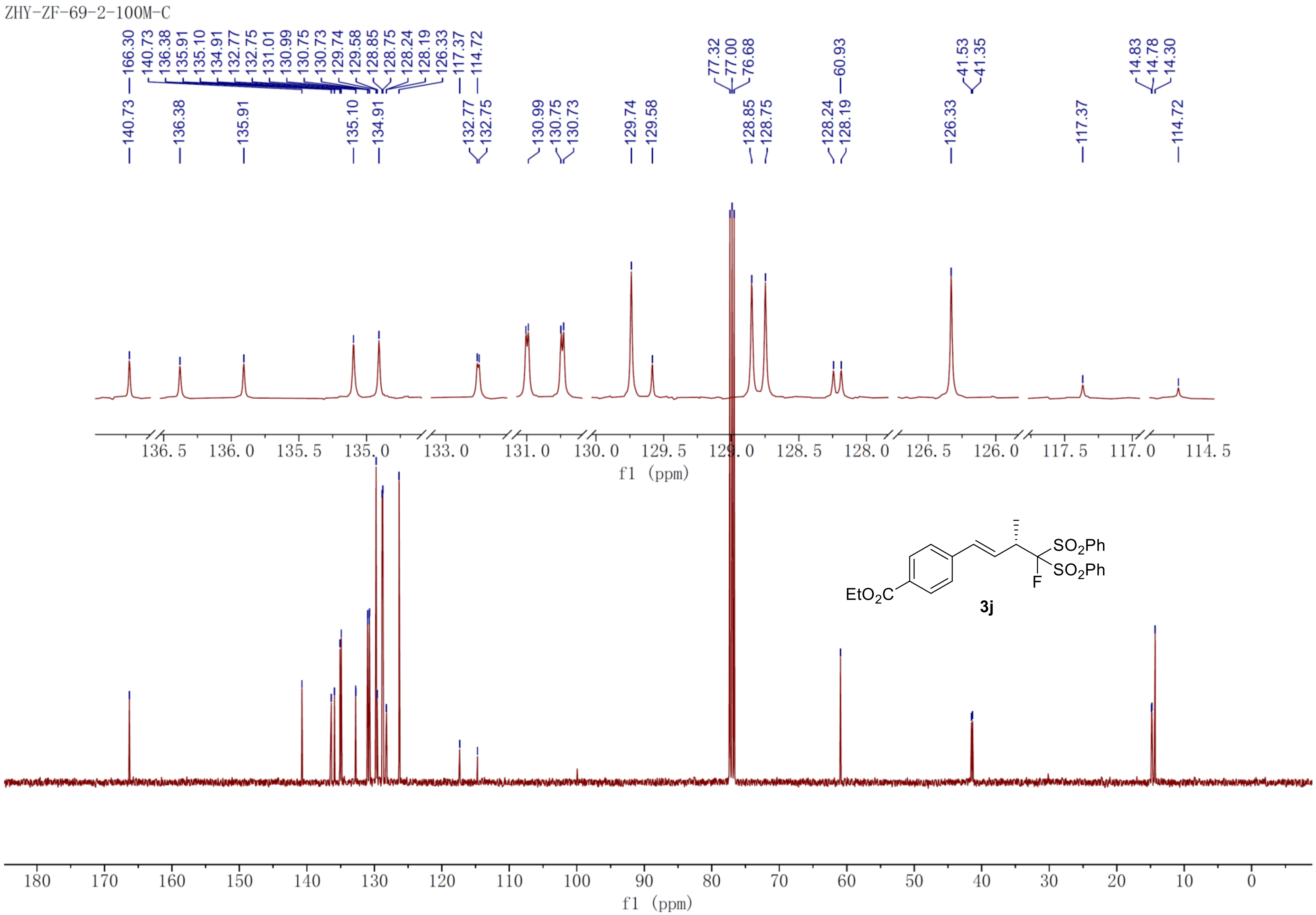
ZHY-ZF-69-2-100M-C
166.30
140.73
136.38
135.91
135.10
134.91
132.77
132.75
131.01
130.99
130.75
130.73
129.74
129.58
128.85
128.75
128.24
128.19
126.33
117.37
114.72
77.32
77.00
76.68
60.93
41.53
41.35
14.83
14.78
14.30
f1 (ppm)
SO2Ph
SO2Ph
F
EtO2C
3j

ZHY-ZF-69-2-376M-F

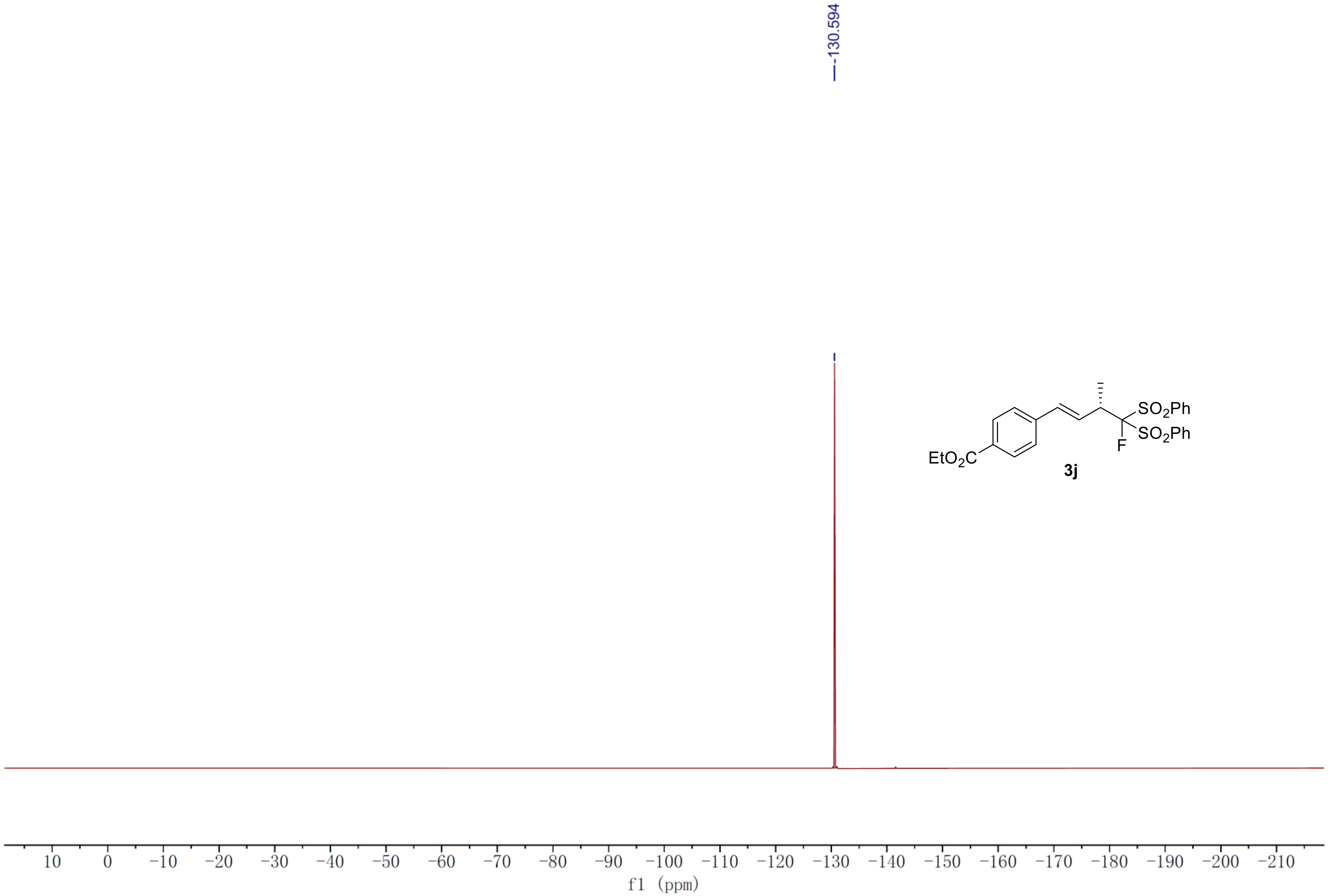

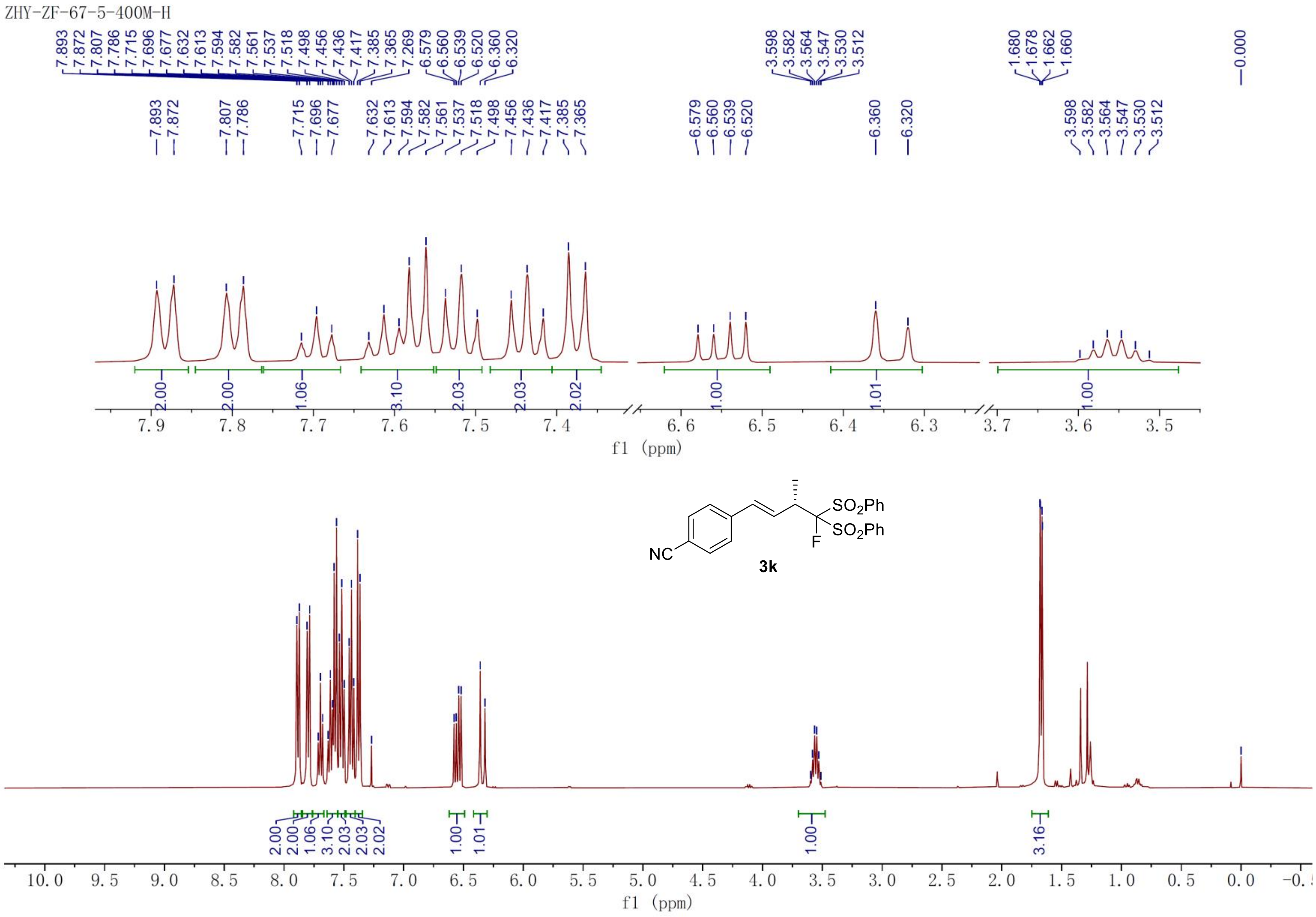
ZHY-ZF-67-5-400M-H
7.893
7.872
7.807
7.786
7.715
7.696
7.677
7.632
7.613
7.594
7.582
7.561
7.537
7.518
7.498
7.456
7.436
7.417
7.385
7.365
7.269
6.579
6.560
6.539
6.520
6.360
6.320
3.598
3.582
3.564
3.547
3.530
3.512
1.680
1.678
1.662
1.660
0.000
2.00
2.00
1.06
3.10
2.03
2.03
2.02
1.00
1.01
1.00
3.16
f1 (ppm)
SO2Ph
SO2Ph
F
NC
3k

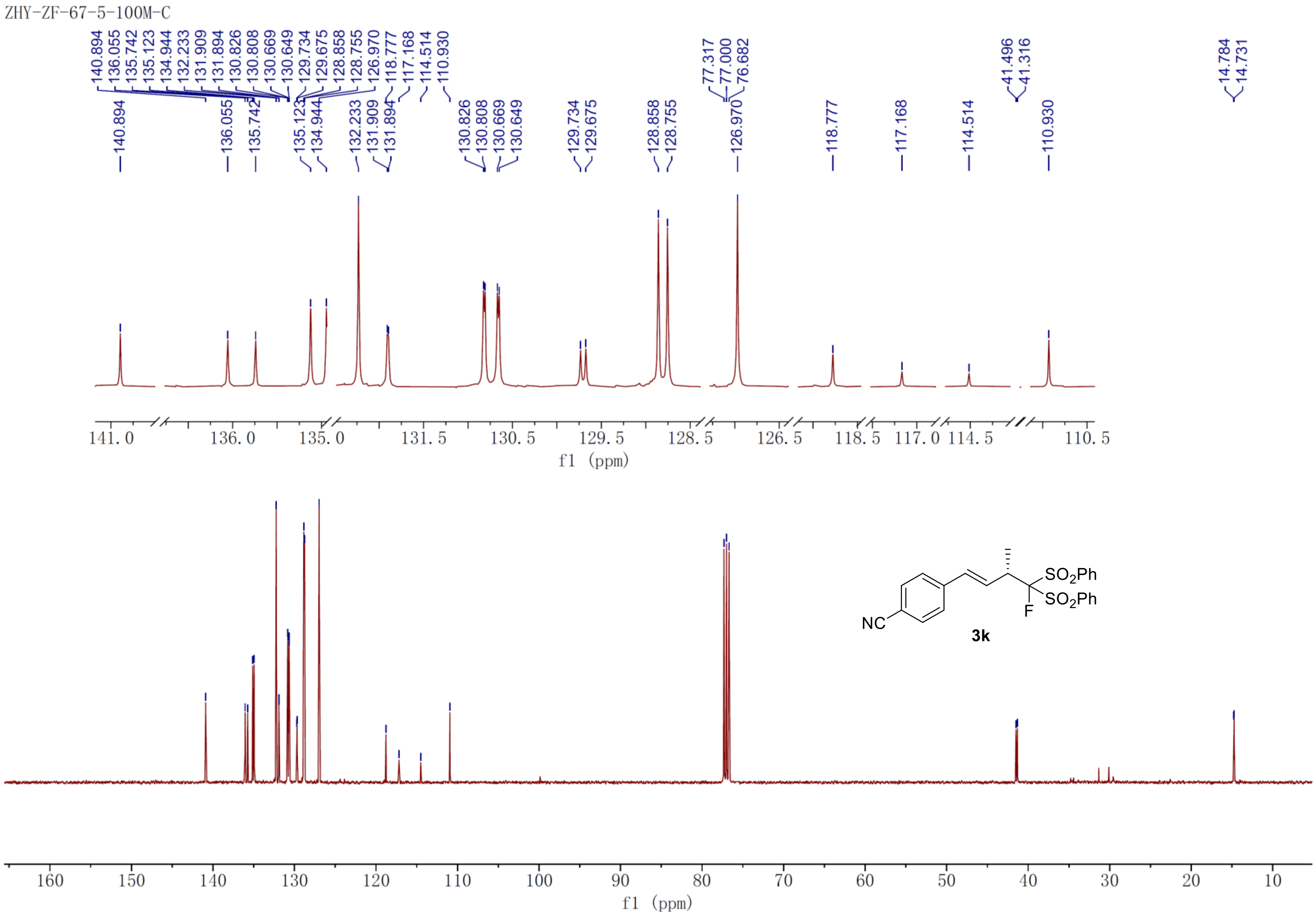
ZHY-ZF-67-5-100M-C
140.894
136.055
135.742
135.123
134.944
132.233
131.909
131.894
130.826
130.808
130.669
130.649
129.734
129.675
128.858
128.755
126.970
118.777
117.168
114.514
110.930
77.317
77.000
76.682
41.496
41.316
14.784
14.731
f1 (ppm)
SO2Ph
SO2Ph
F
NC
3k

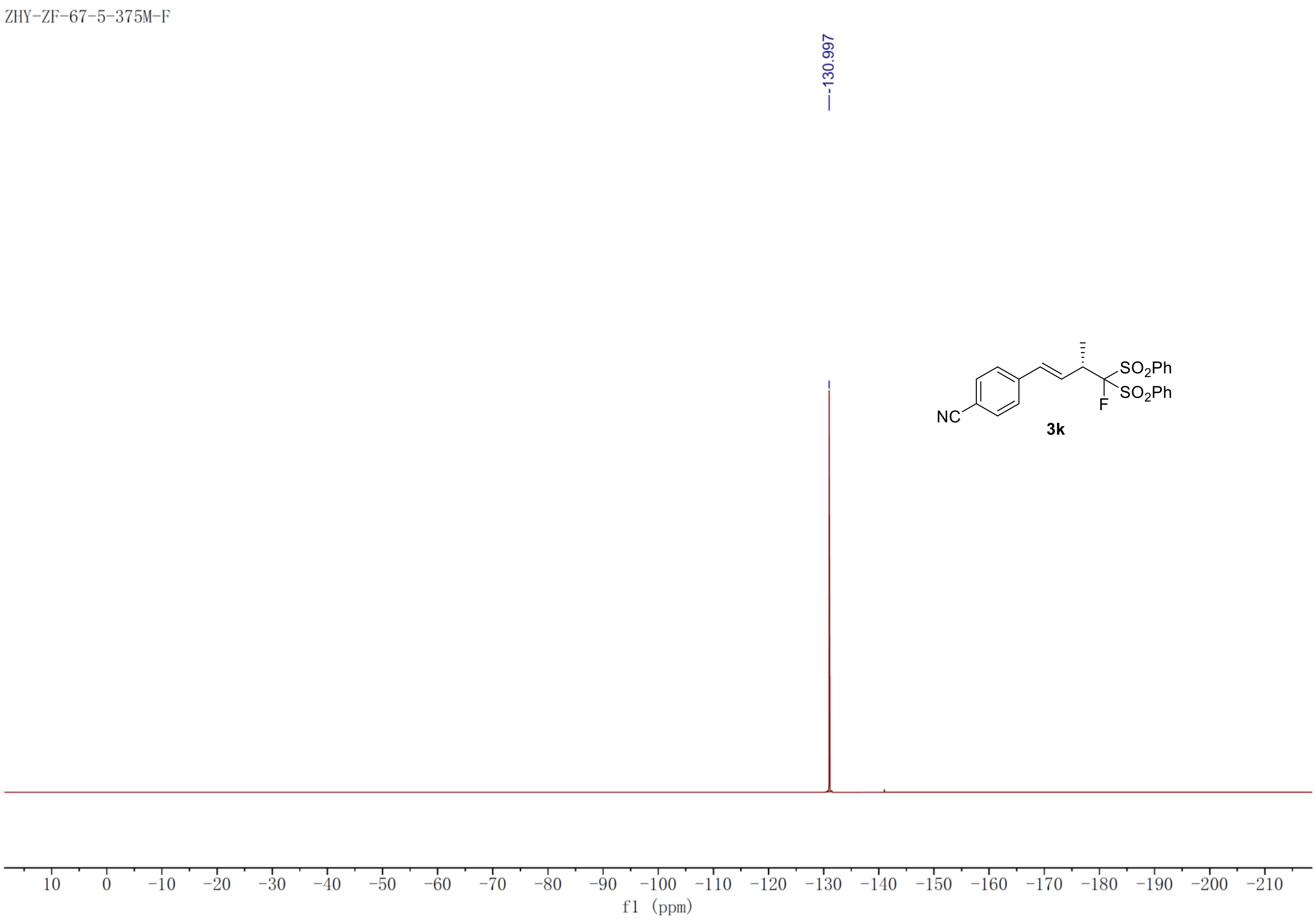
ZHY-ZF-67-5-375M-F
-130.997
NC
SO2Ph
SO2Ph
F
3k
10 0 −10 −20 −30 −40 −50 −60 −70 −80 −90 −100 −110 −120 −130 −140 −150 −160 −170 −180 −190 −200 −210
f1 (ppm)

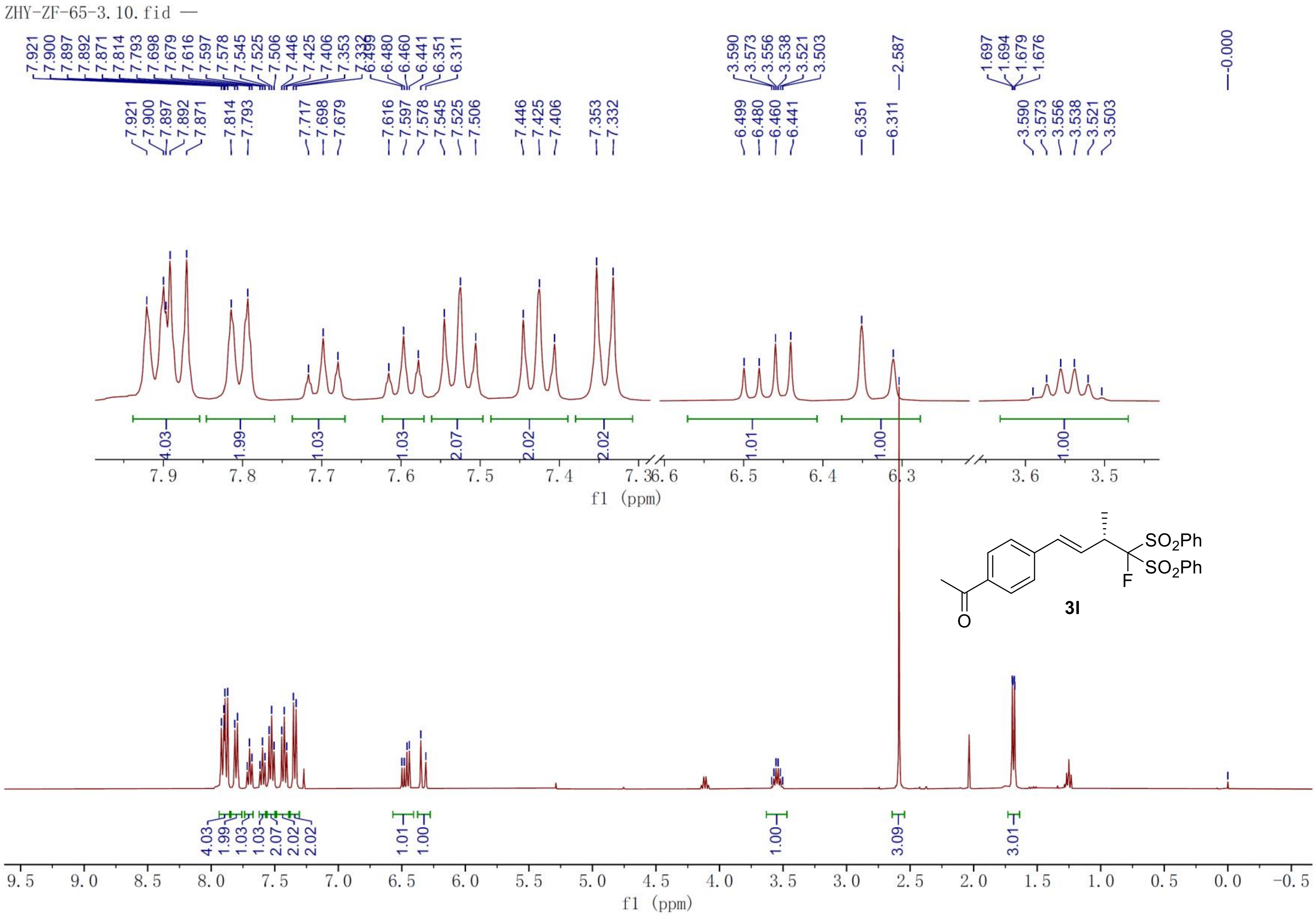
ZHY-ZF-65-3.10.fid
7.921 7.900 7.897 7.892 7.871 7.814 7.793 7.717 7.698 7.679 7.616 7.597 7.578 7.545 7.525 7.506 7.446 7.425 7.406 7.353 7.332
6.499 6.480 6.460 6.441 6.351 6.311
3.590 3.573 3.556 3.538 3.521 3.503
2.587
1.697 1.694 1.679 1.676
0.000
4.03 1.99 1.03 1.03 2.07 2.02 2.02 1.01 1.00 1.00 3.09 3.01
f1 (ppm)
SO2Ph
SO2Ph
F
O
3l

ZHY-ZF-67-3-100M-C

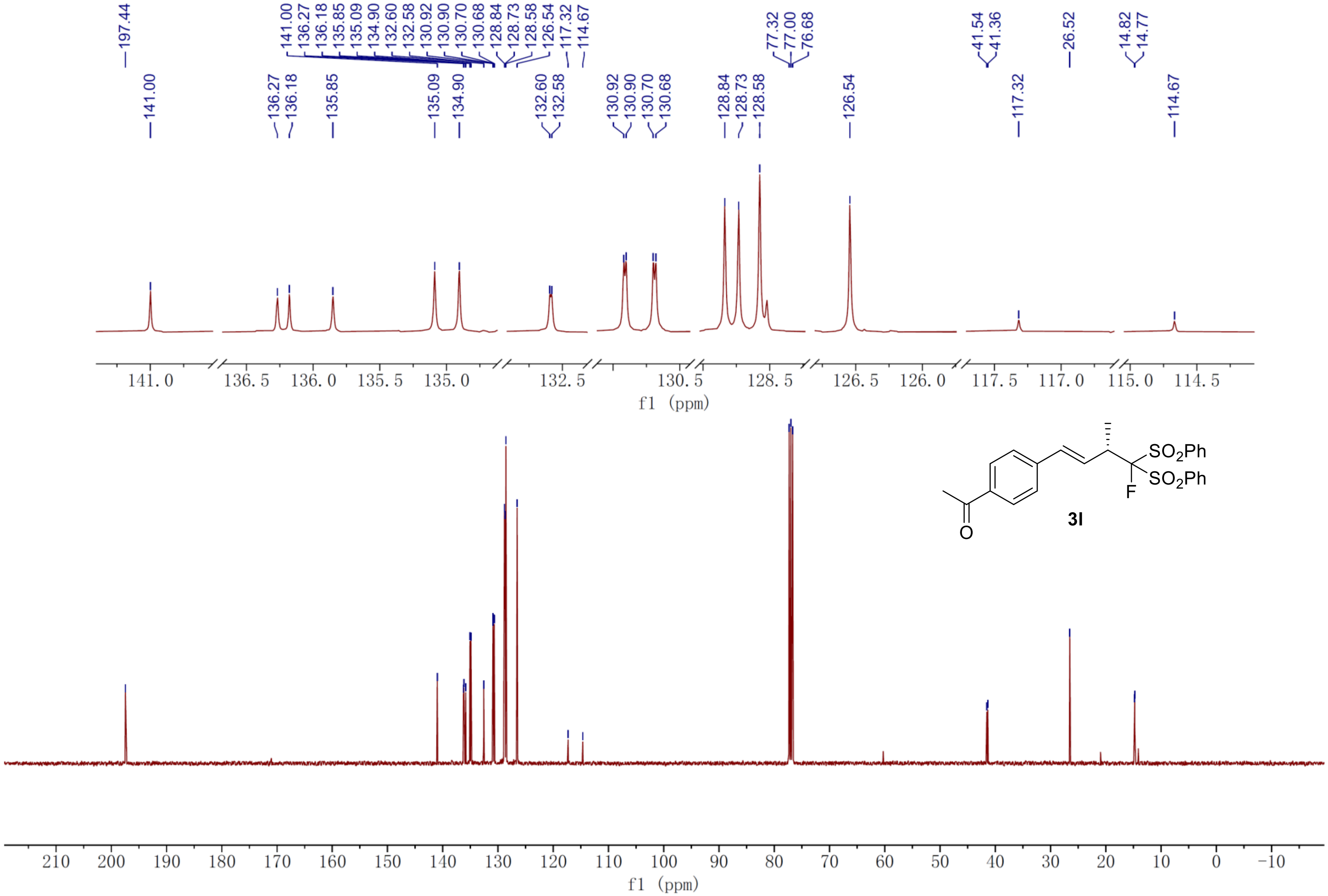

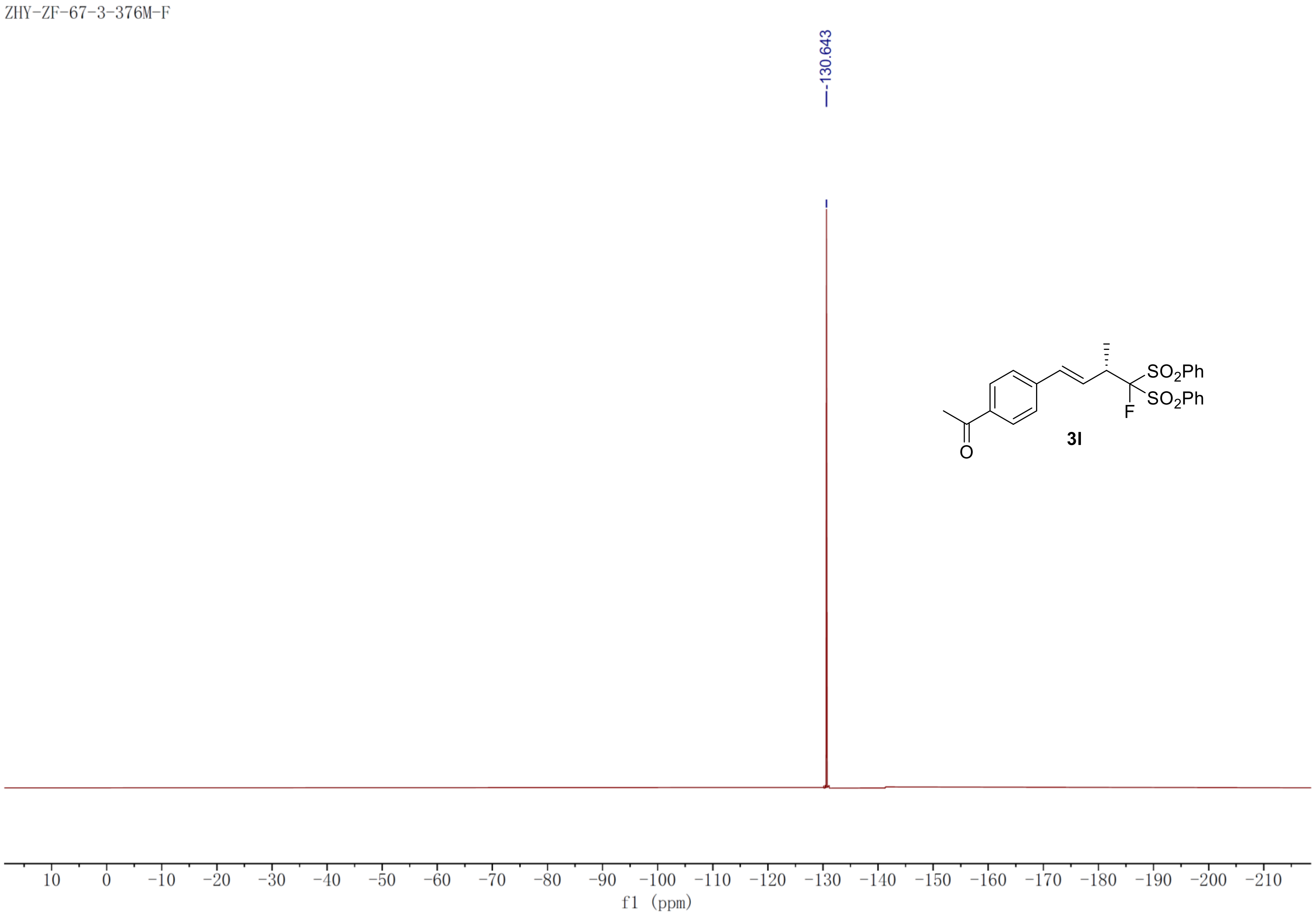
ZHY-ZF-67-3-376M-F
-130.643
SO2Ph
SO2Ph
F
O
3l
10 0 −10 −20 −30 −40 −50 −60 −70 −80 −90 −100 −110 −120 −130 −140 −150 −160 −170 −180 −190 −200 −210
f1 (ppm)

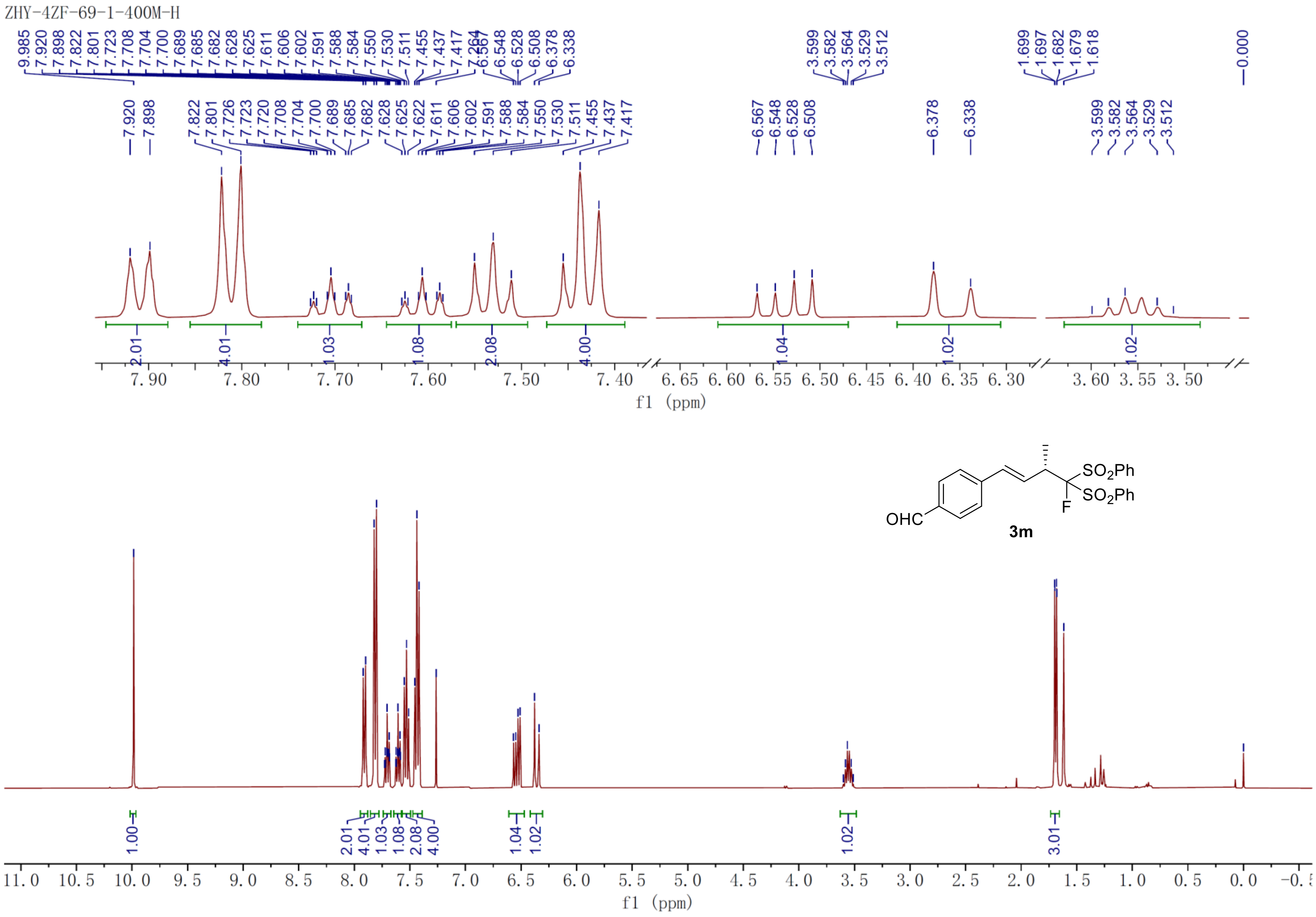
ZHY-4ZF-69-1-400M-H
SO2Ph
SO2Ph
F
OHC
3m
f1 (ppm)

ZHY-4ZF-69-1-100M-C

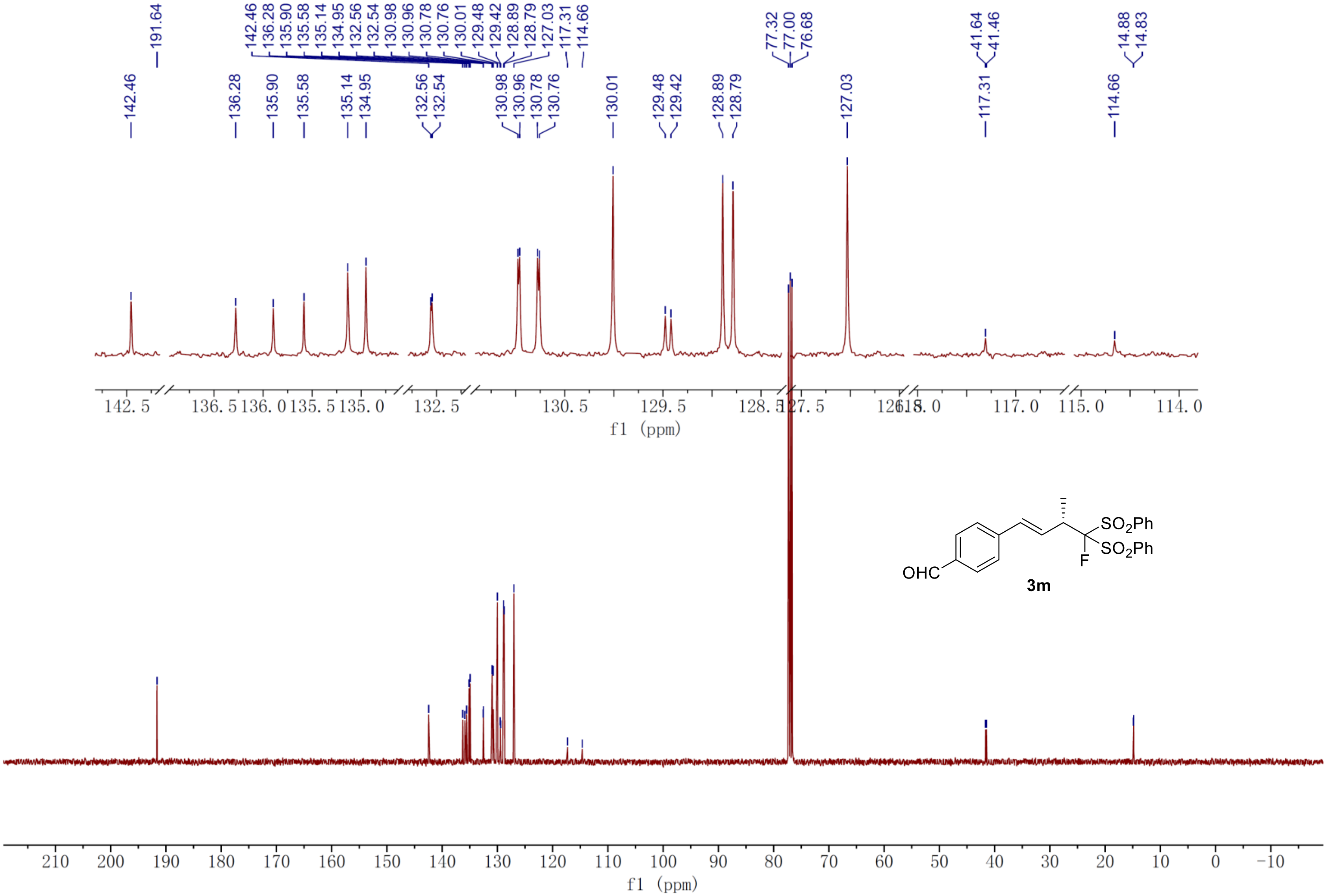

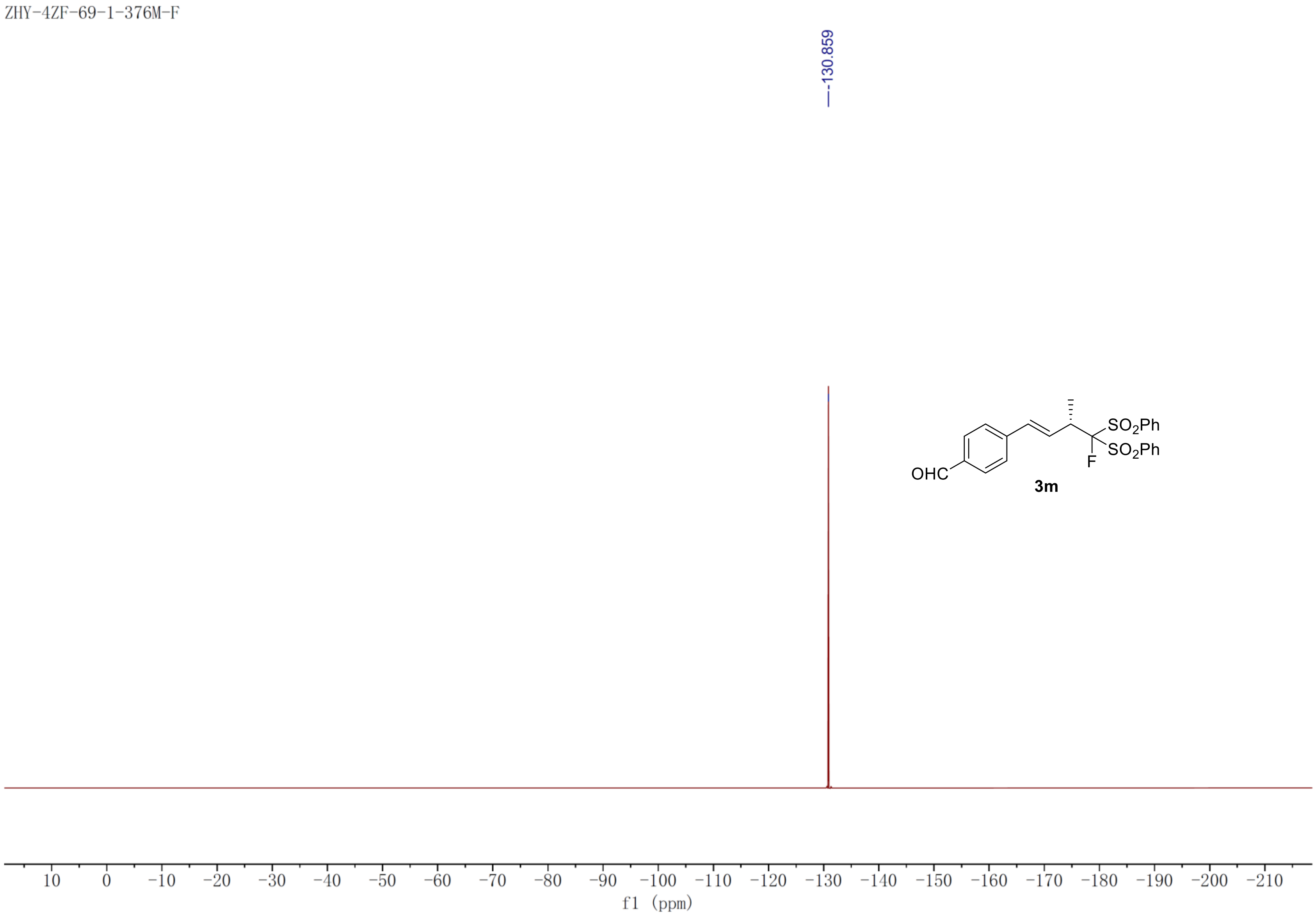
ZHY-4ZF-69-1-376M-F
-130.859
SO2Ph
SO2Ph
F
OHC
3m
10 0 −10 −20 −30 −40 −50 −60 −70 −80 −90 −100 −110 −120 −130 −140 −150 −160 −170 −180 −190 −200 −210
f1 (ppm)

ZHY-ZC-105-400M-H

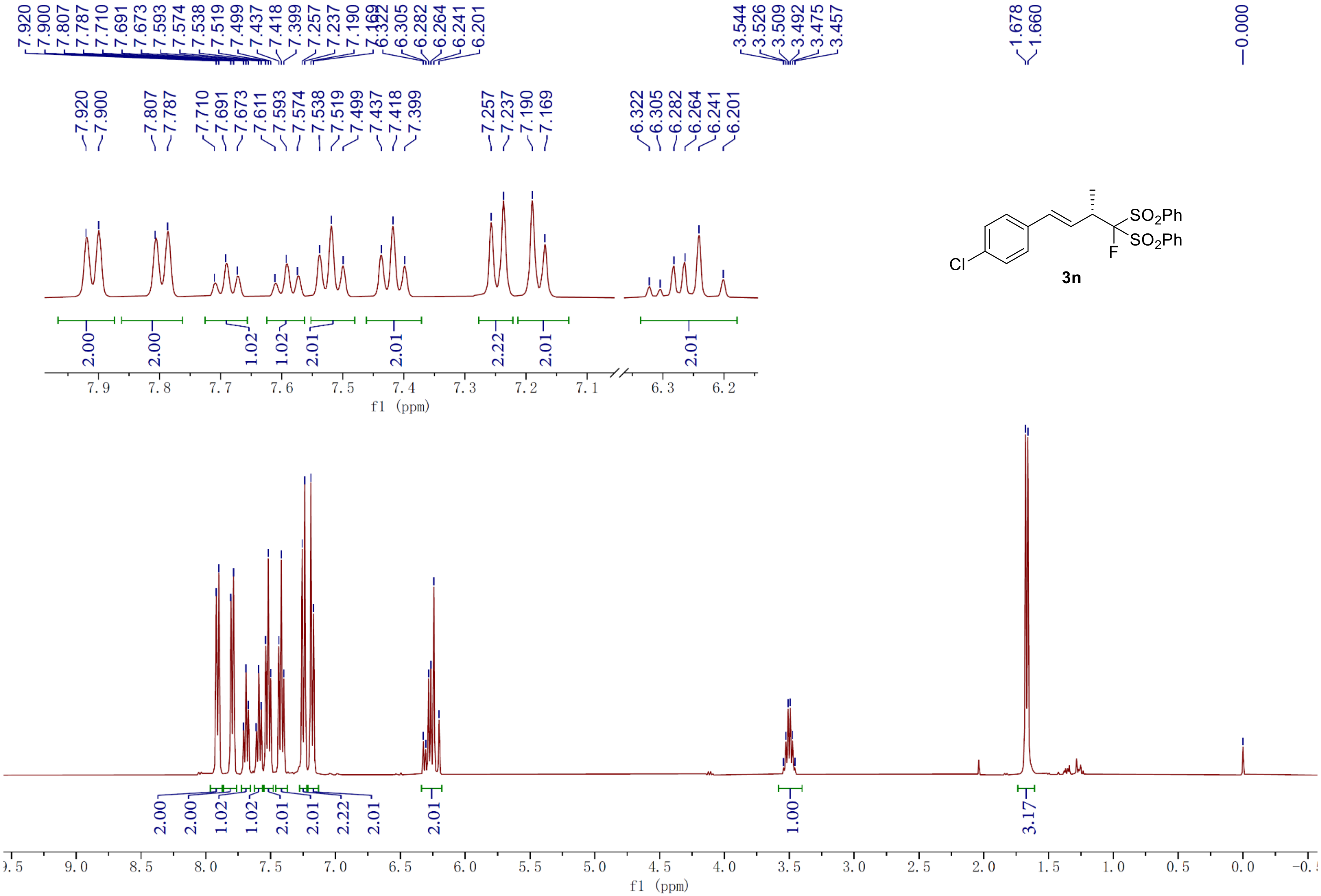

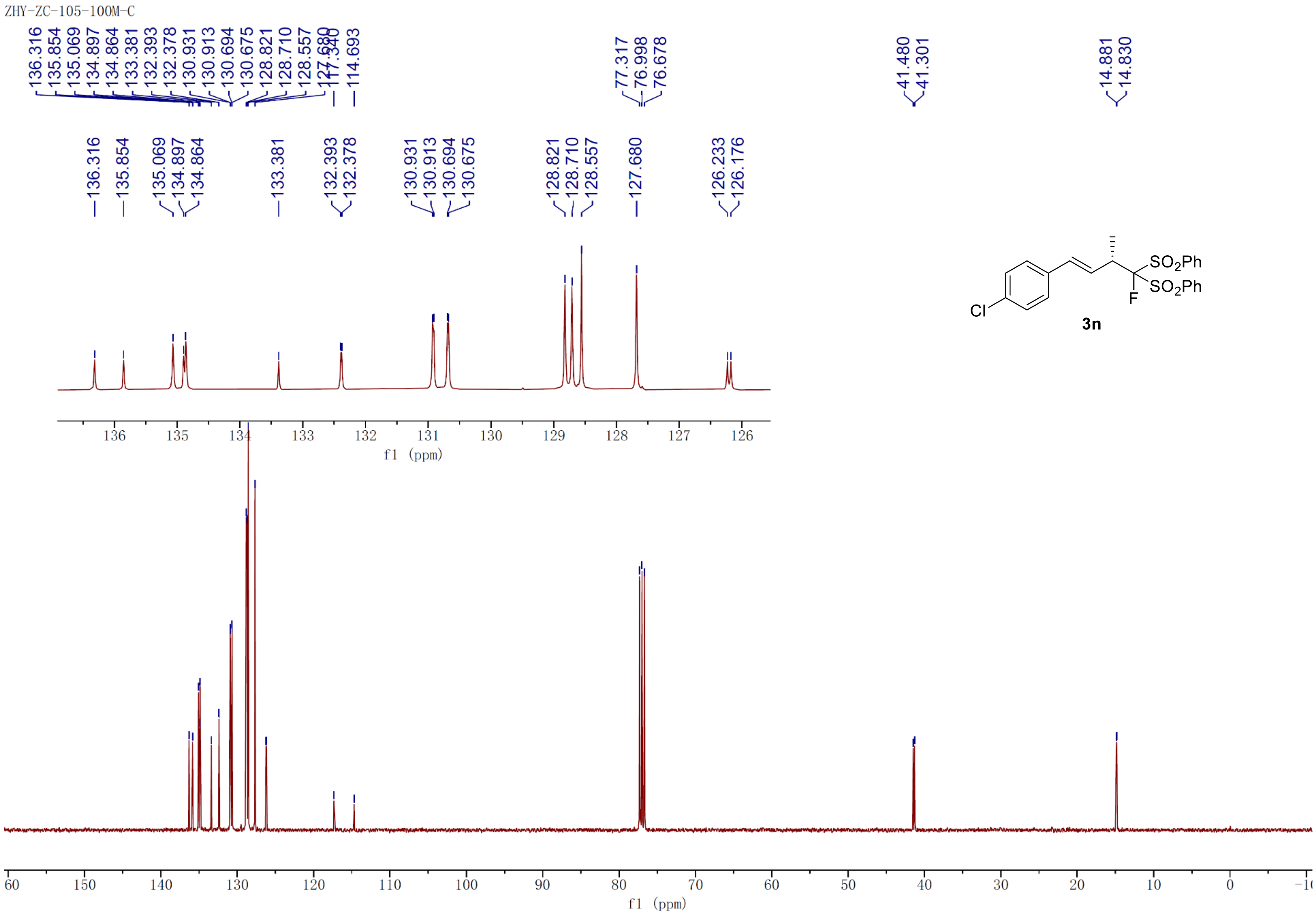
ZHY-ZC-105-100M-C
136.316
135.854
135.069
134.897
134.864
133.381
132.393
132.378
130.931
130.913
130.694
130.675
128.821
128.710
128.557
127.680
117.340
114.693
77.317
76.998
76.678
41.480
41.301
14.881
14.830
126.233
126.176
f1 (ppm)
SO2Ph
SO2Ph
F
Cl
3n

ZHY-ZC-105-376M-F

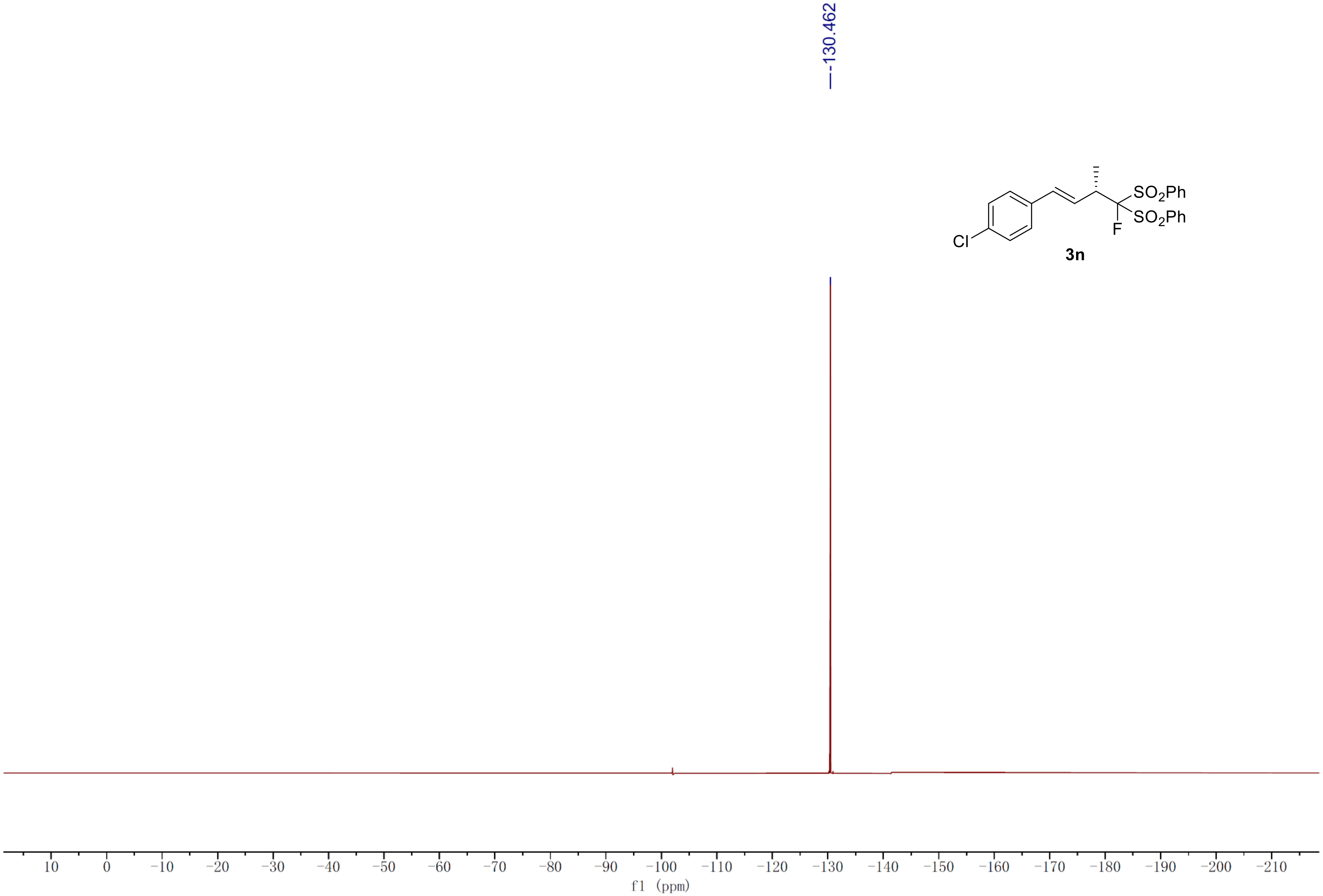

3n

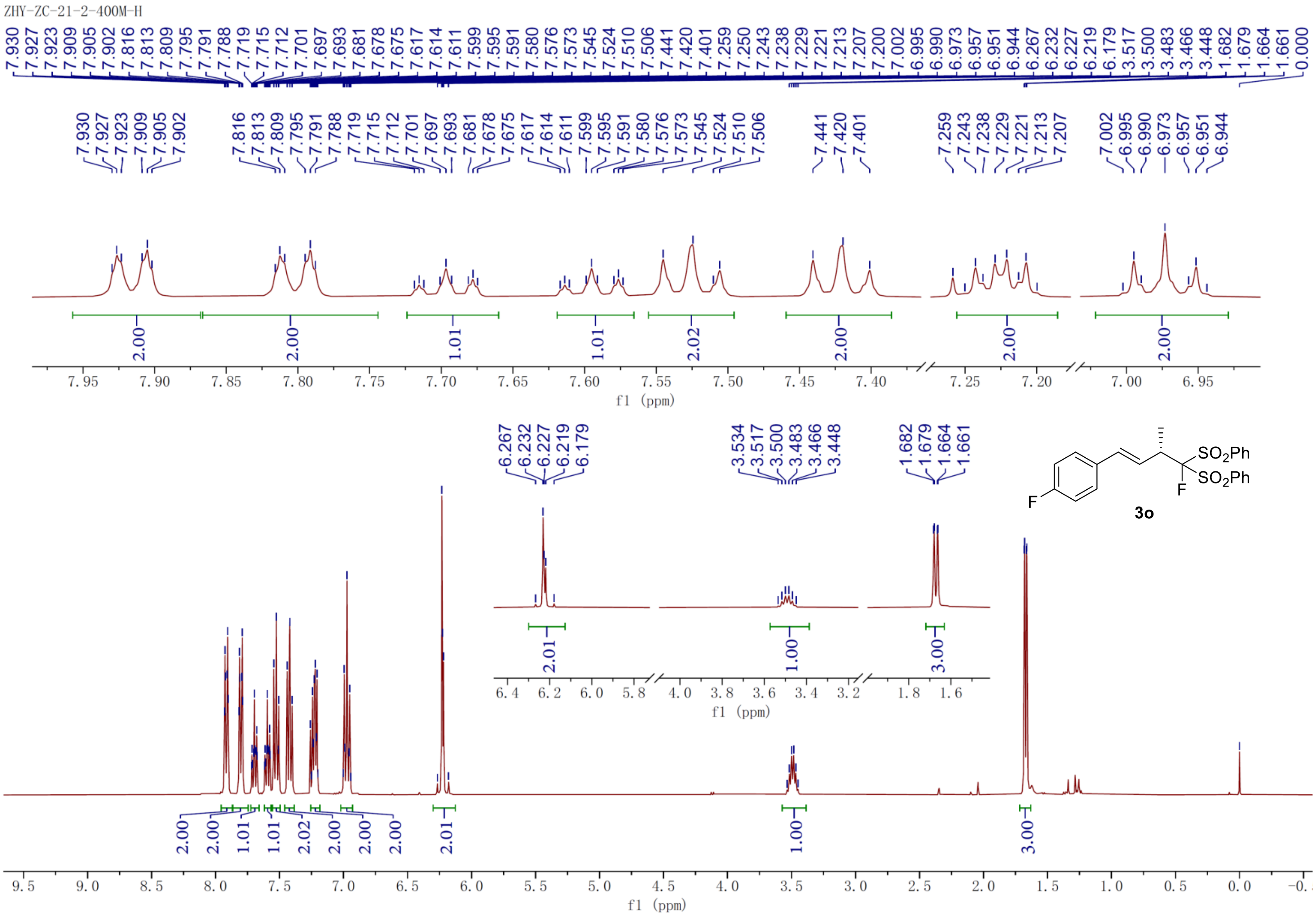
ZHY-ZC-21-2-400M-H
SO2Ph
SO2Ph
F
F
3o
f1 (ppm)

ZHY-ZC-21-2-100M-C

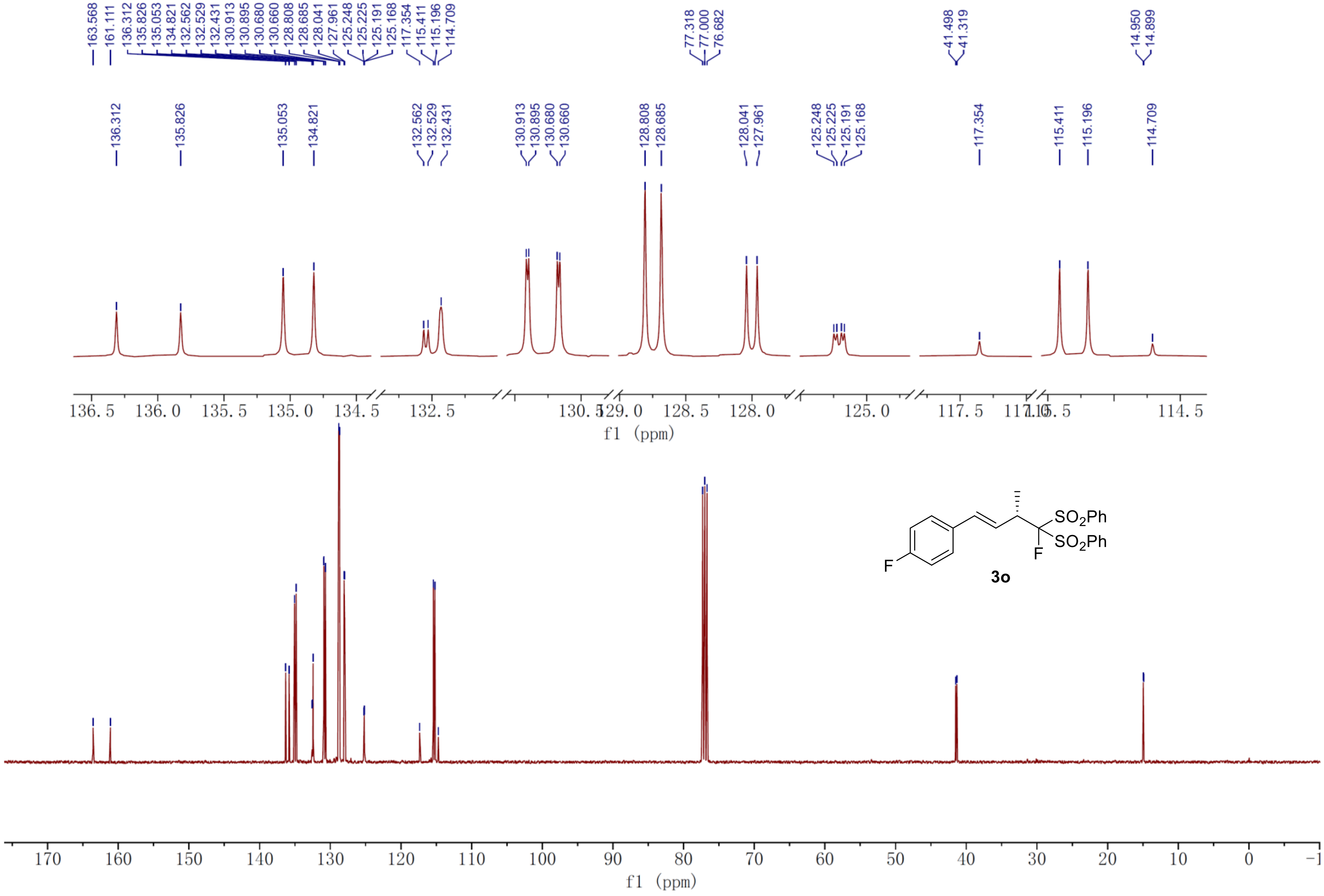

ZHY-ZC-21-2-376M-F

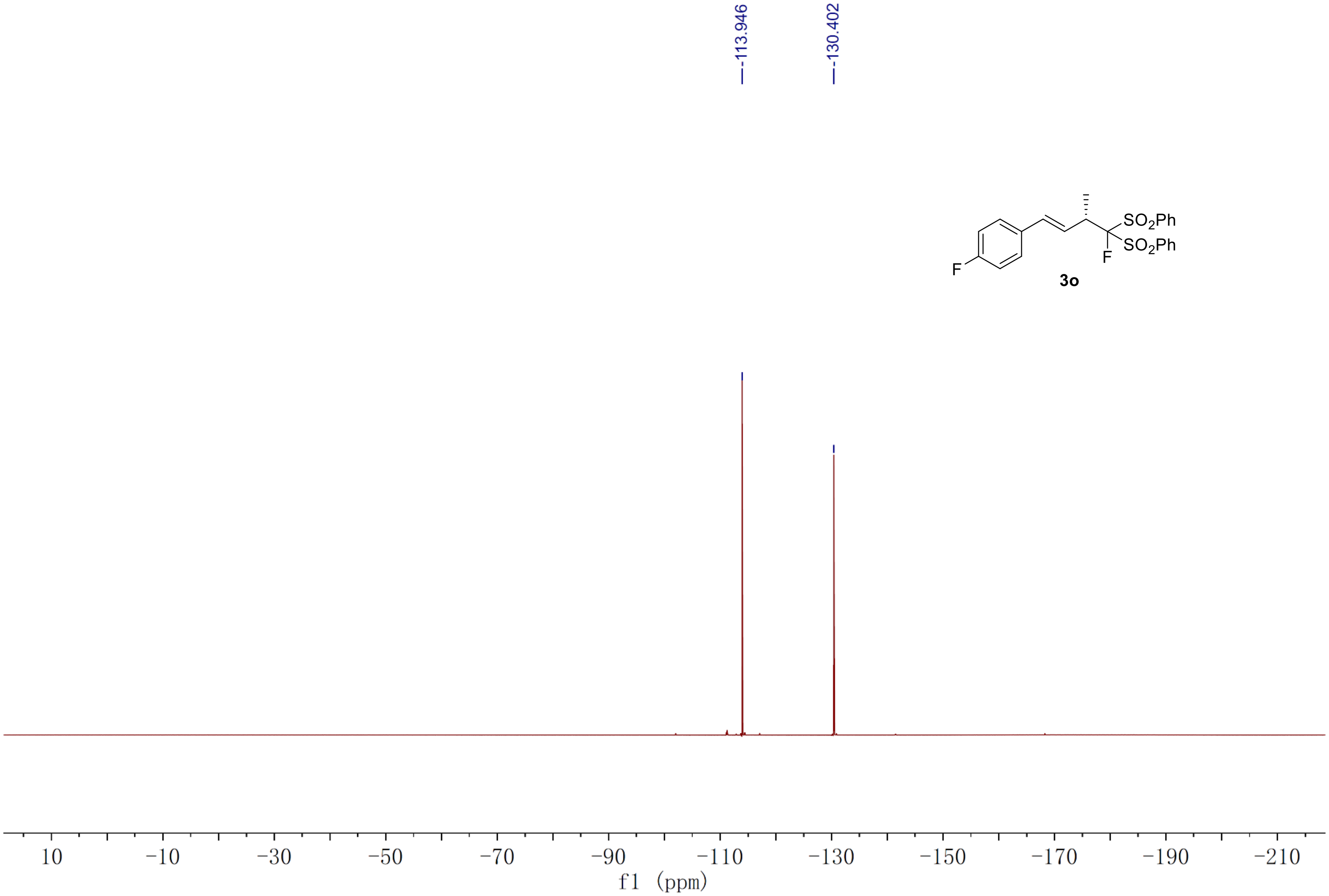

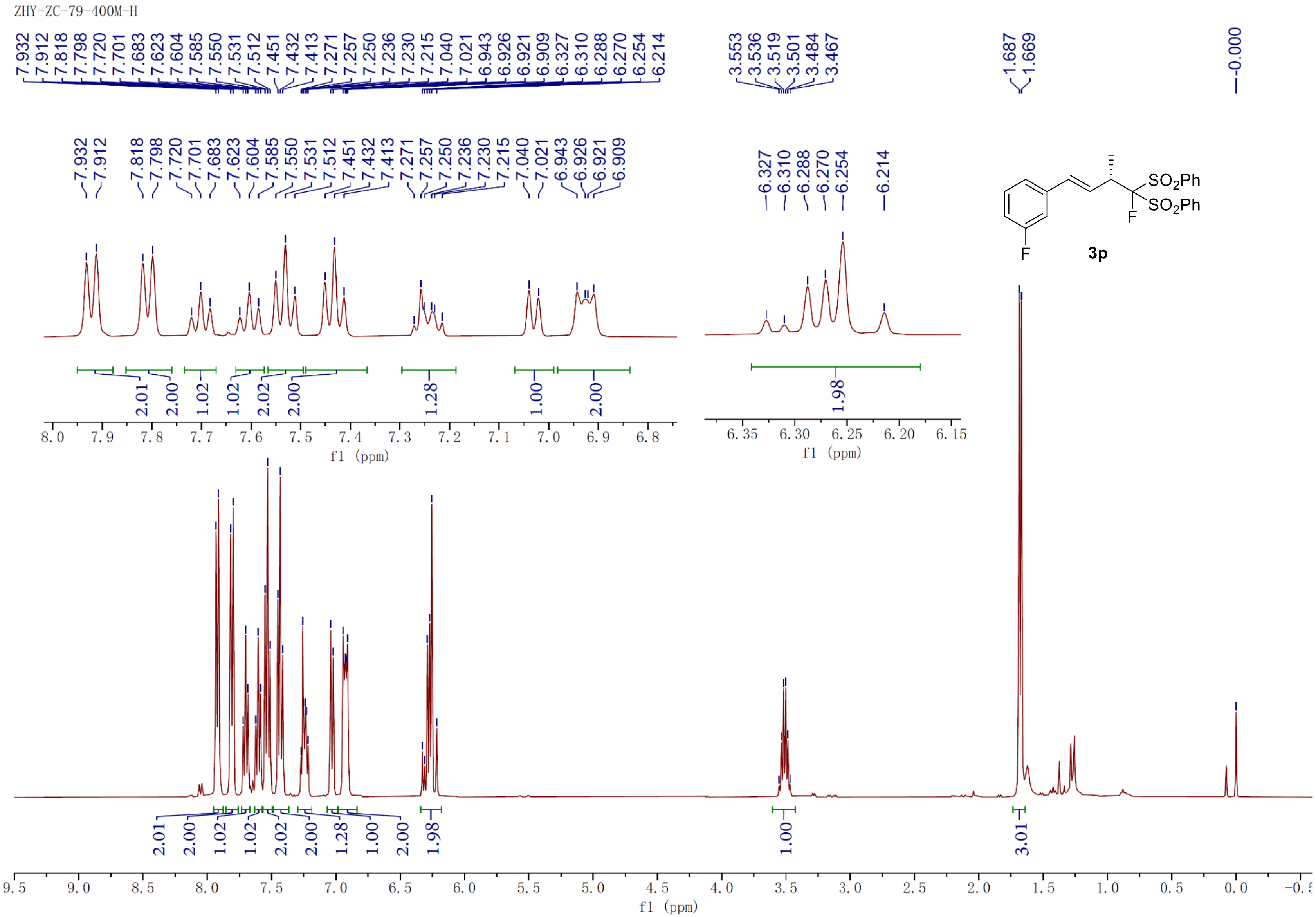
ZHY-ZC-79-400M-H
SO2Ph
SO2Ph
F
F
3p
f1 (ppm)

ZHY-ZC-79-100M-C

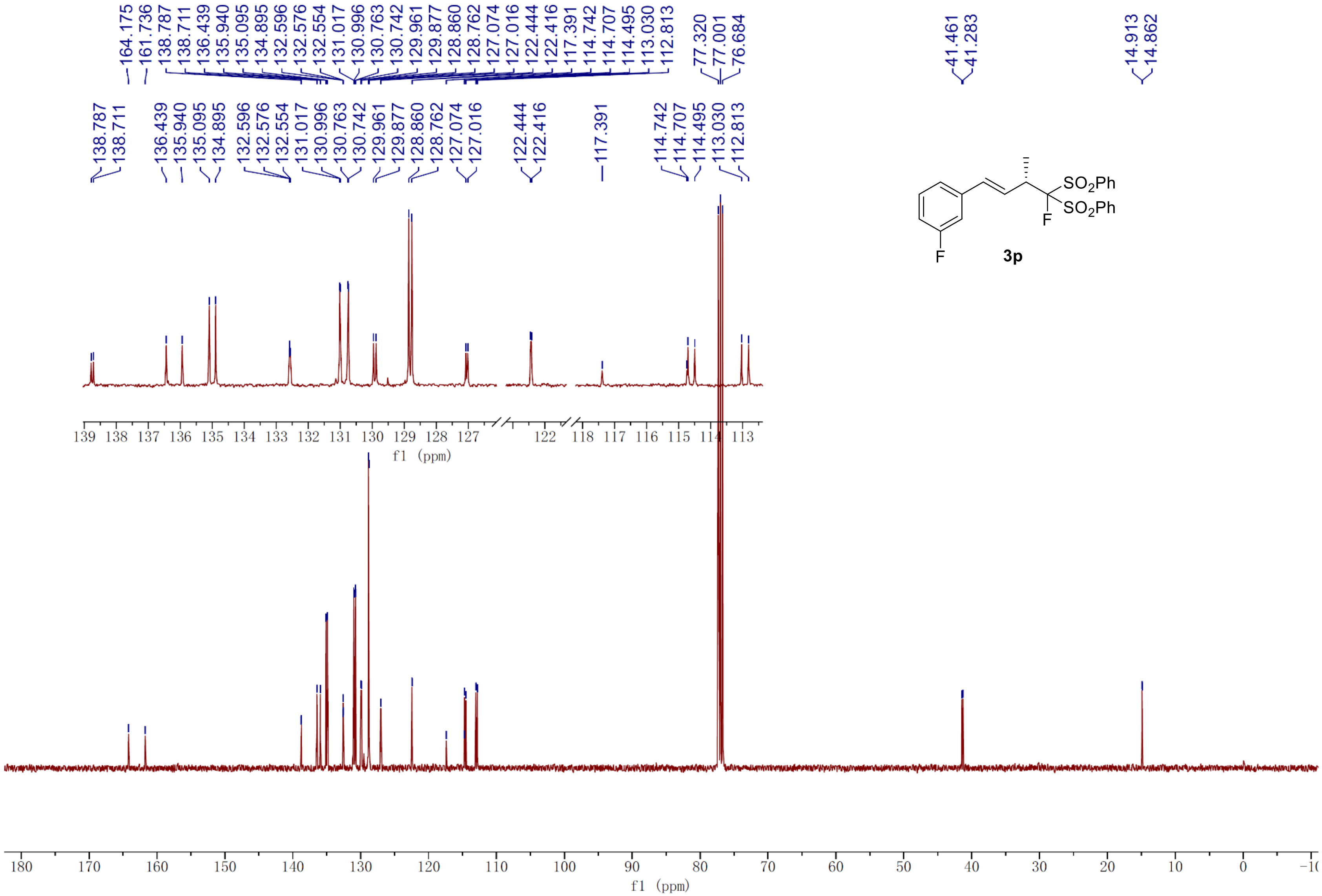

3p

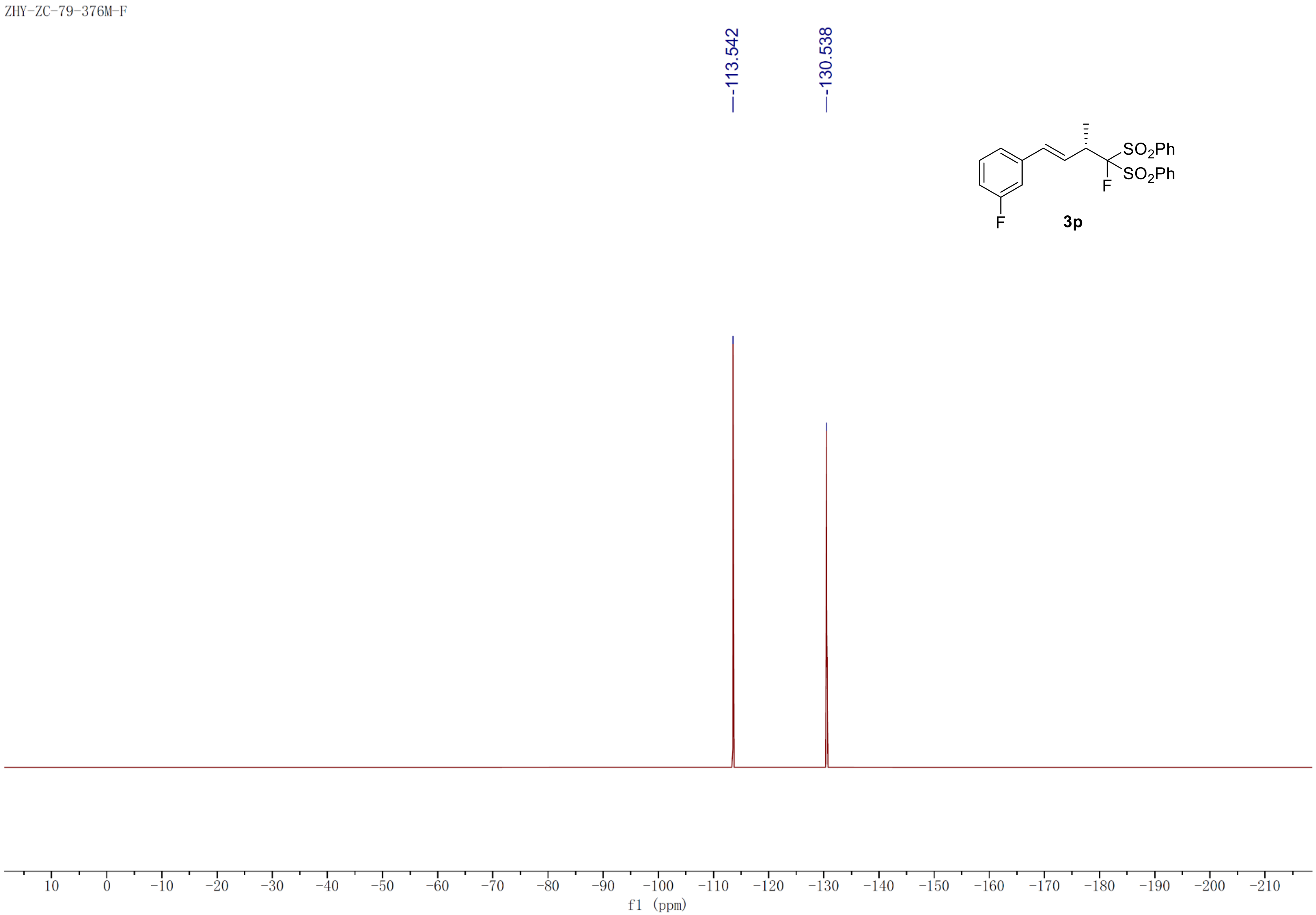
ZHY-ZC-79-376M-F
-113.542
-130.538
SO2Ph
SO2Ph
F
F
3p
10 0 −10 −20 −30 −40 −50 −60 −70 −80 −90 −100 −110 −120 −130 −140 −150 −160 −170 −180 −190 −200 −210
f1 (ppm)

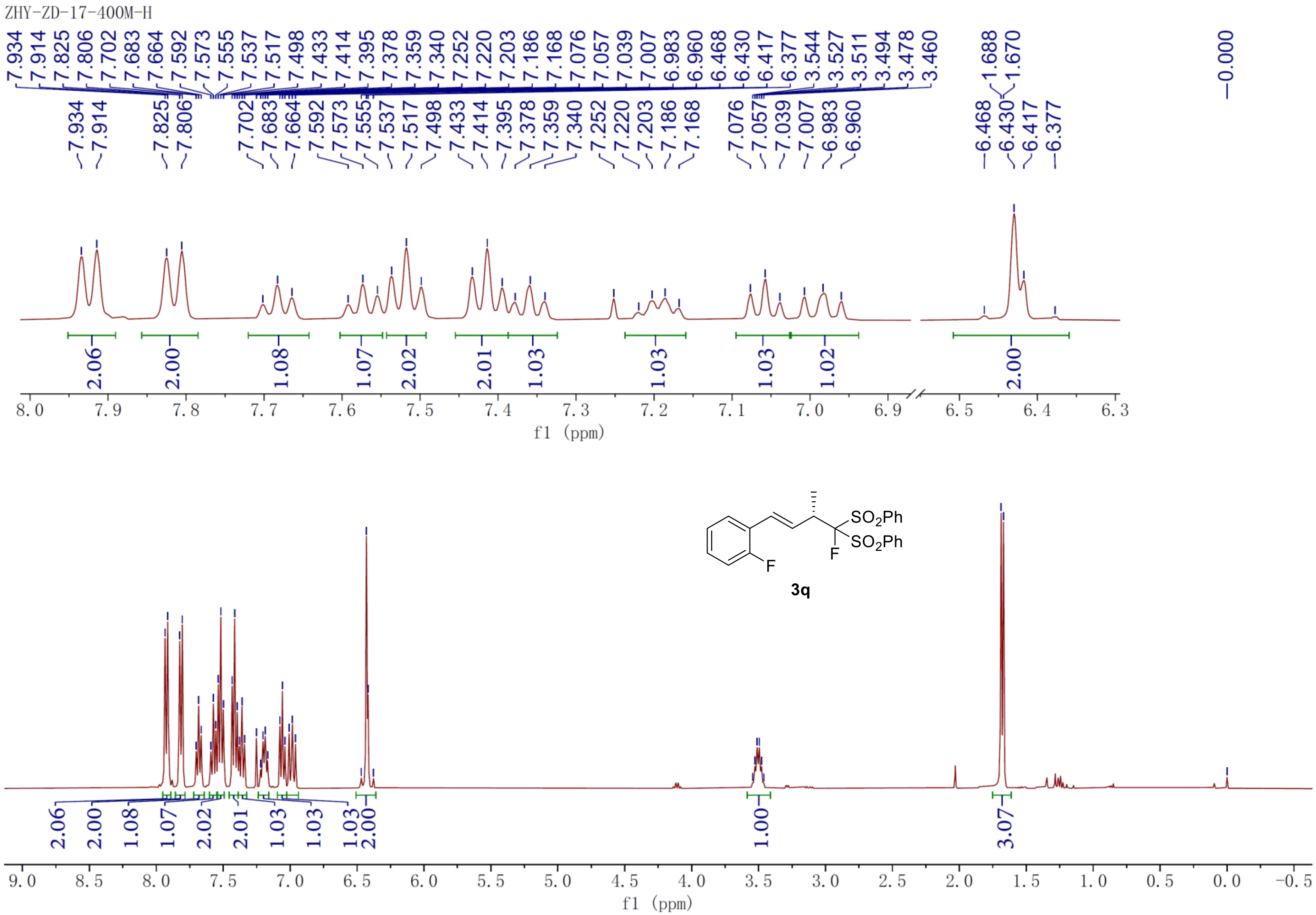
ZHY-ZD-17-400M-H
f1 (ppm)
SO2Ph
SO2Ph
F
F
3q
f1 (ppm)

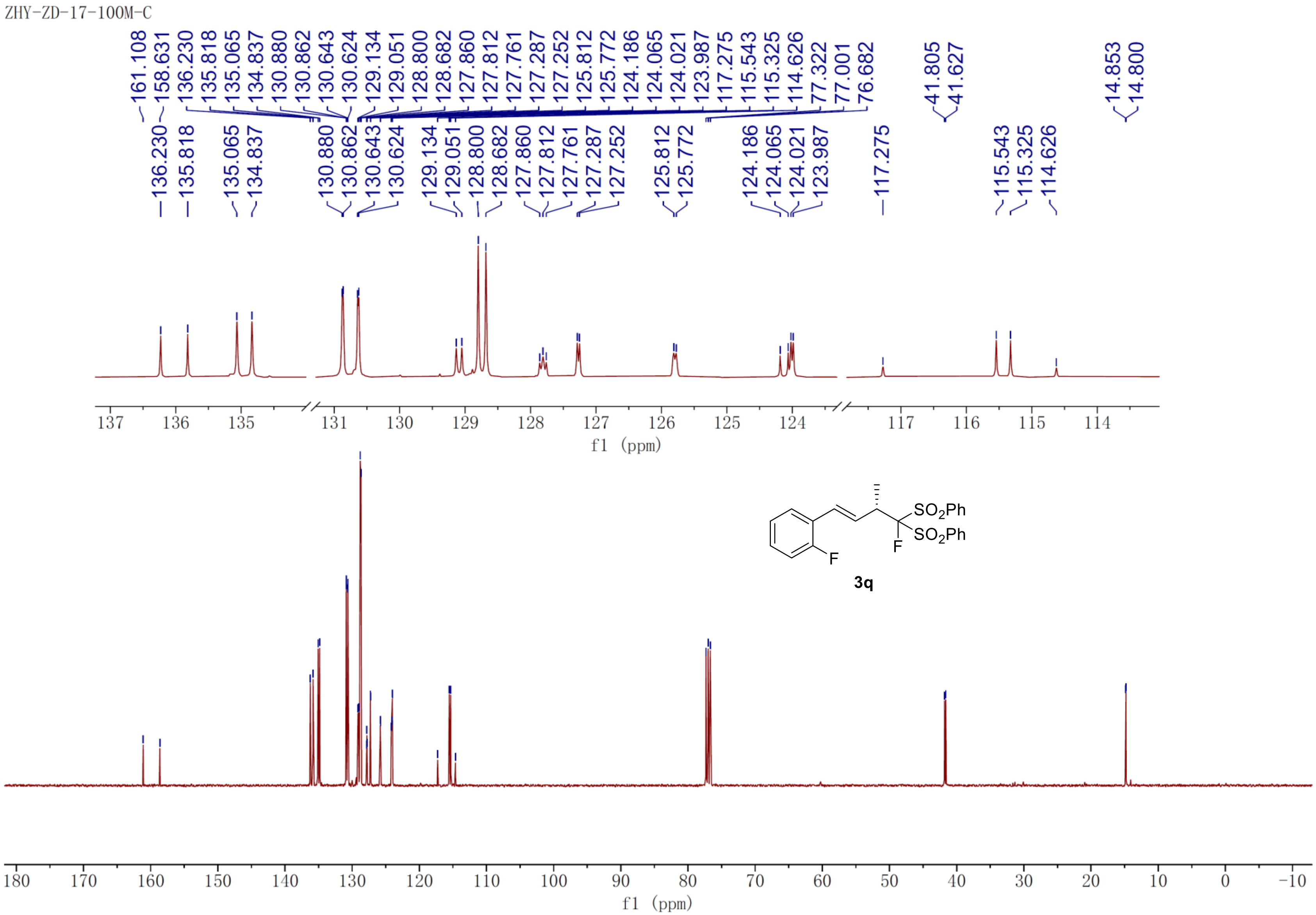
ZHY-ZD-17-100M-C
161.108
158.631
136.230
135.818
135.065
134.837
130.880
130.862
130.643
130.624
129.134
129.051
128.800
128.682
127.860
127.812
127.761
127.287
127.252
125.812
125.772
124.186
124.065
124.021
123.987
117.275
115.543
115.325
114.626
77.322
77.001
76.682
41.805
41.627
14.853
14.800
f1 (ppm)
SO2Ph
SO2Ph
F
F
3q

ZHY-ZD-17-376M-F

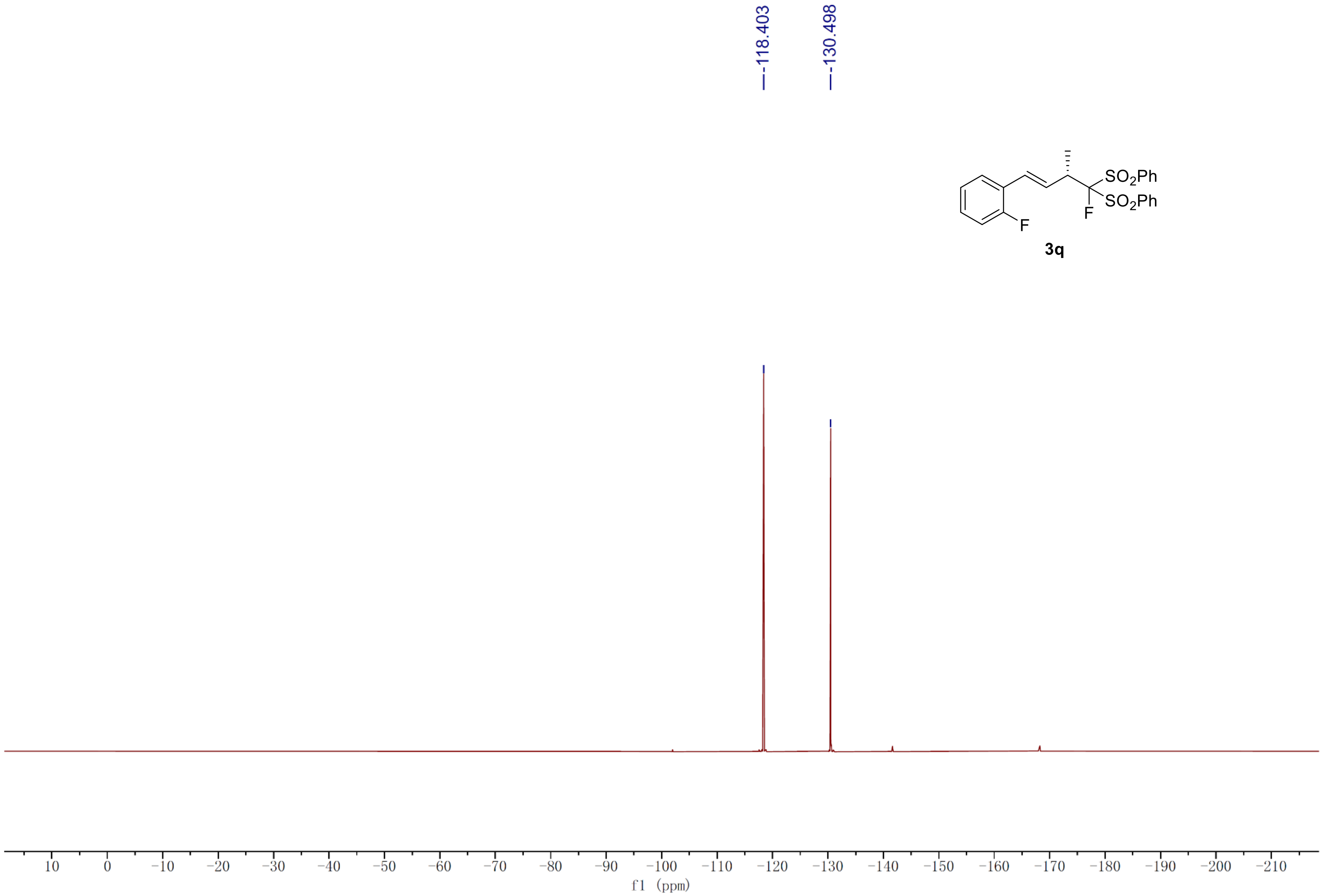

ZHY-ZF-65-2-300M-H

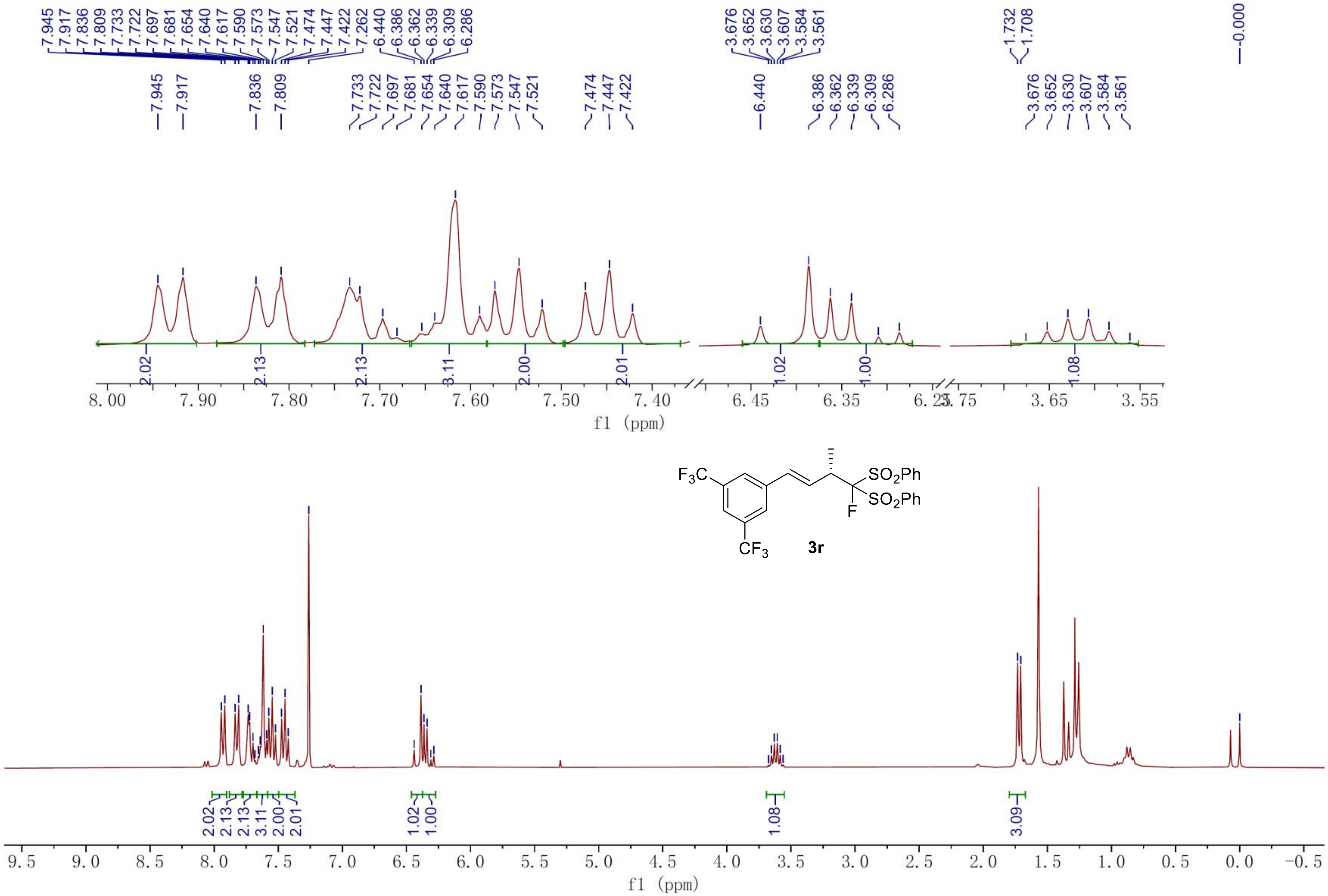

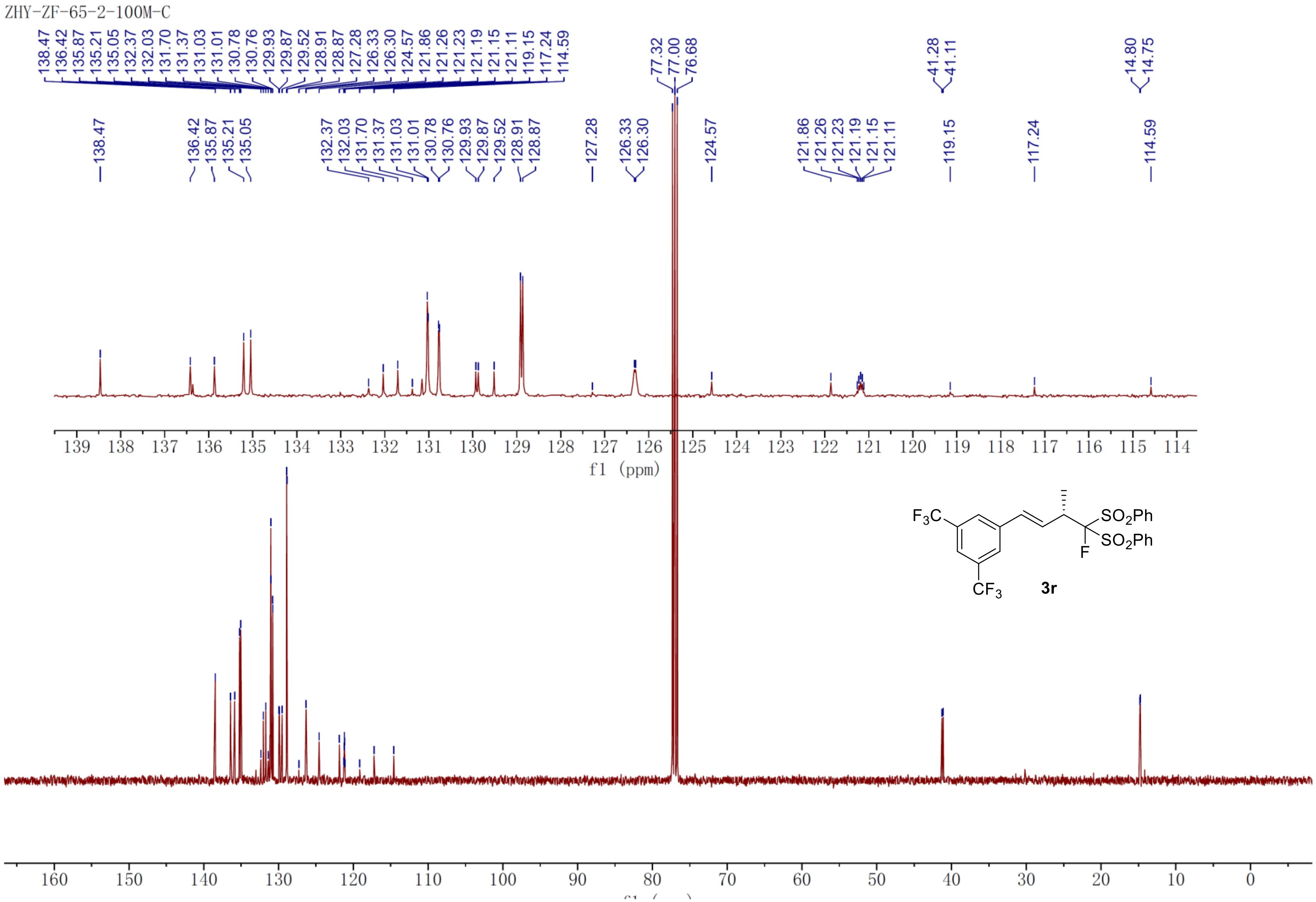
ZHY-ZF-65-2-100M-C
f1 (ppm)
F3C
SO2Ph
SO2Ph
F
CF3
3r

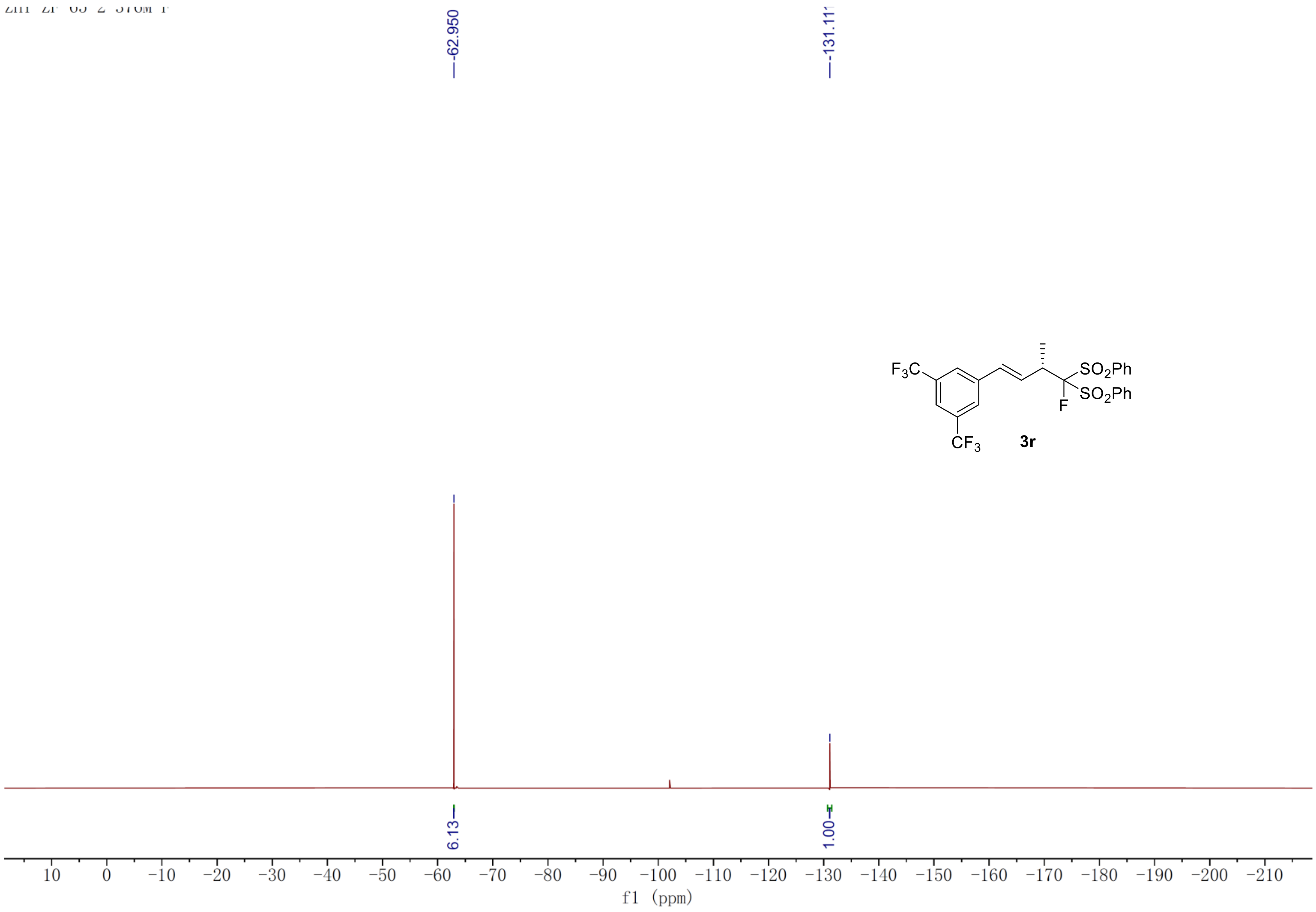

-62.950
6.13
1.00
F3C
SO2Ph
SO2Ph
F
CF3
3r
10 0 −10 −20 −30 −40 −50 −60 −70 −80 −90 −100 −110 −120 −130 −140 −150 −160 −170 −180 −190 −200 −210
f1 (ppm)

ZHY-ZC-26-400M-H

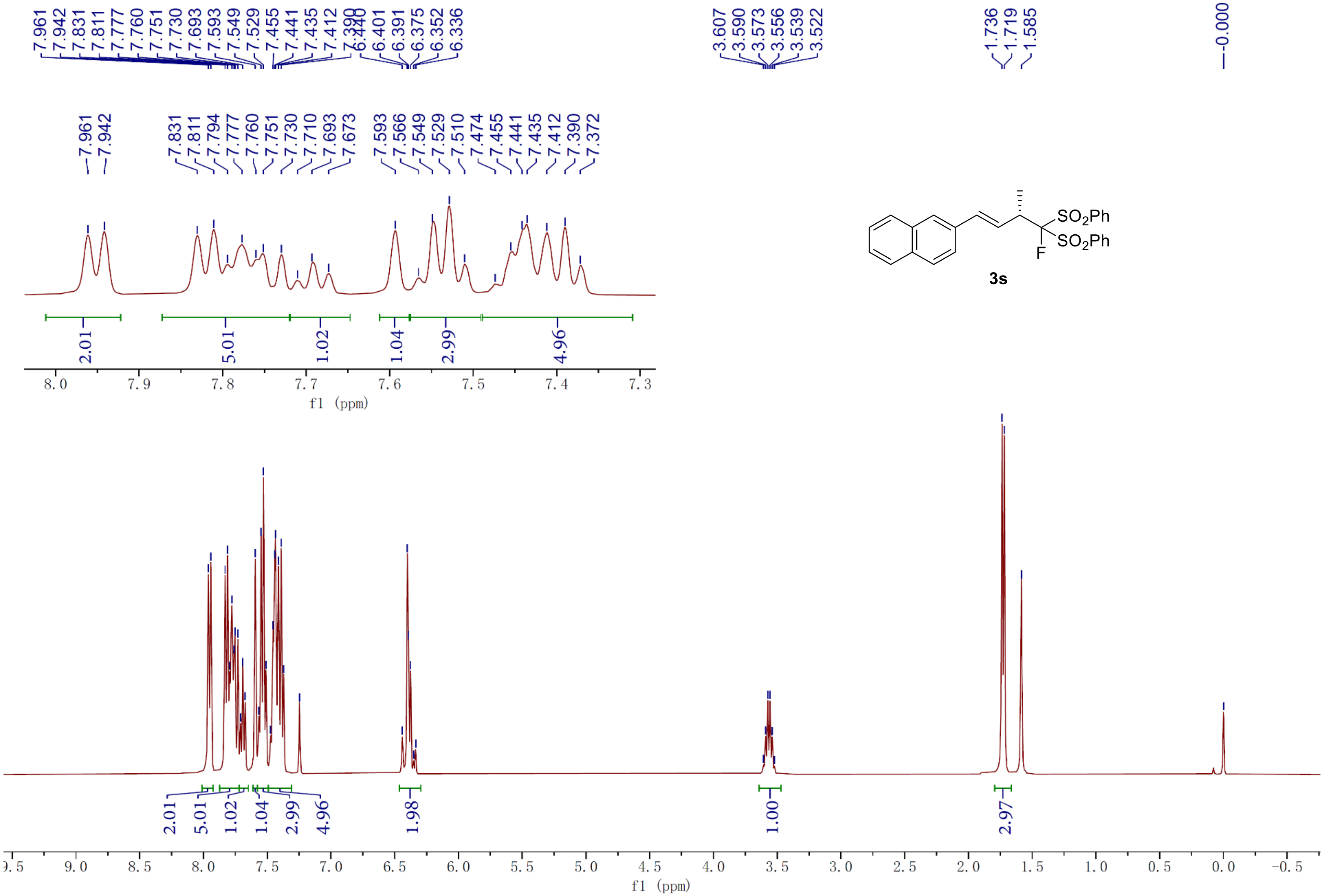

3s

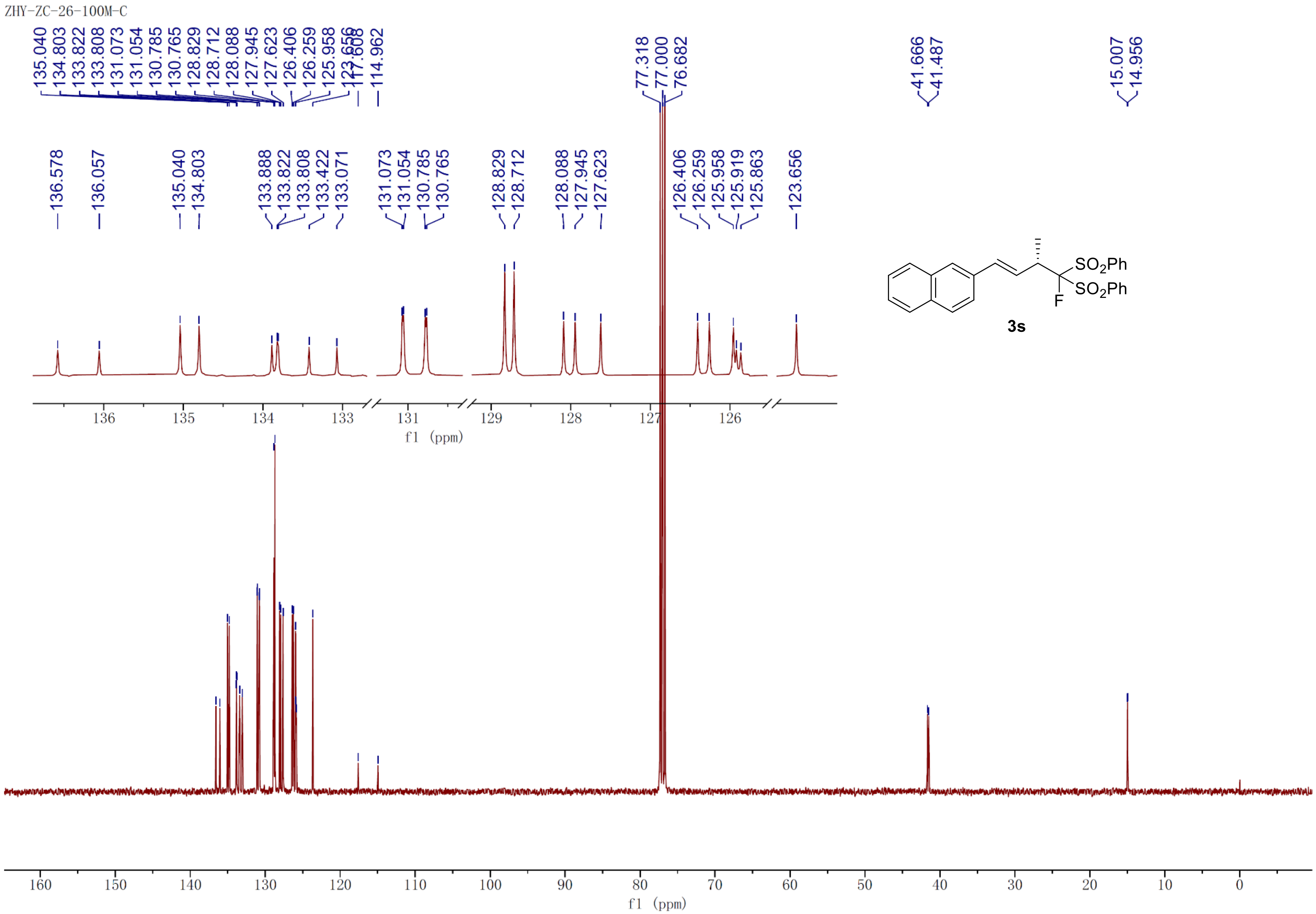
ZHY-ZC-26-100M-C
135.040
134.803
133.822
133.808
131.073
131.054
130.785
130.765
128.829
128.712
128.088
127.945
127.623
126.406
126.259
125.958
123.656
117.608
114.962
77.318
77.000
76.682
41.666
41.487
15.007
14.956
136.578
136.057
133.888
133.422
133.071
125.919
125.863
SO2Ph
SO2Ph
F
3s
f1 (ppm)

ZHY-ZC-26-376M-F

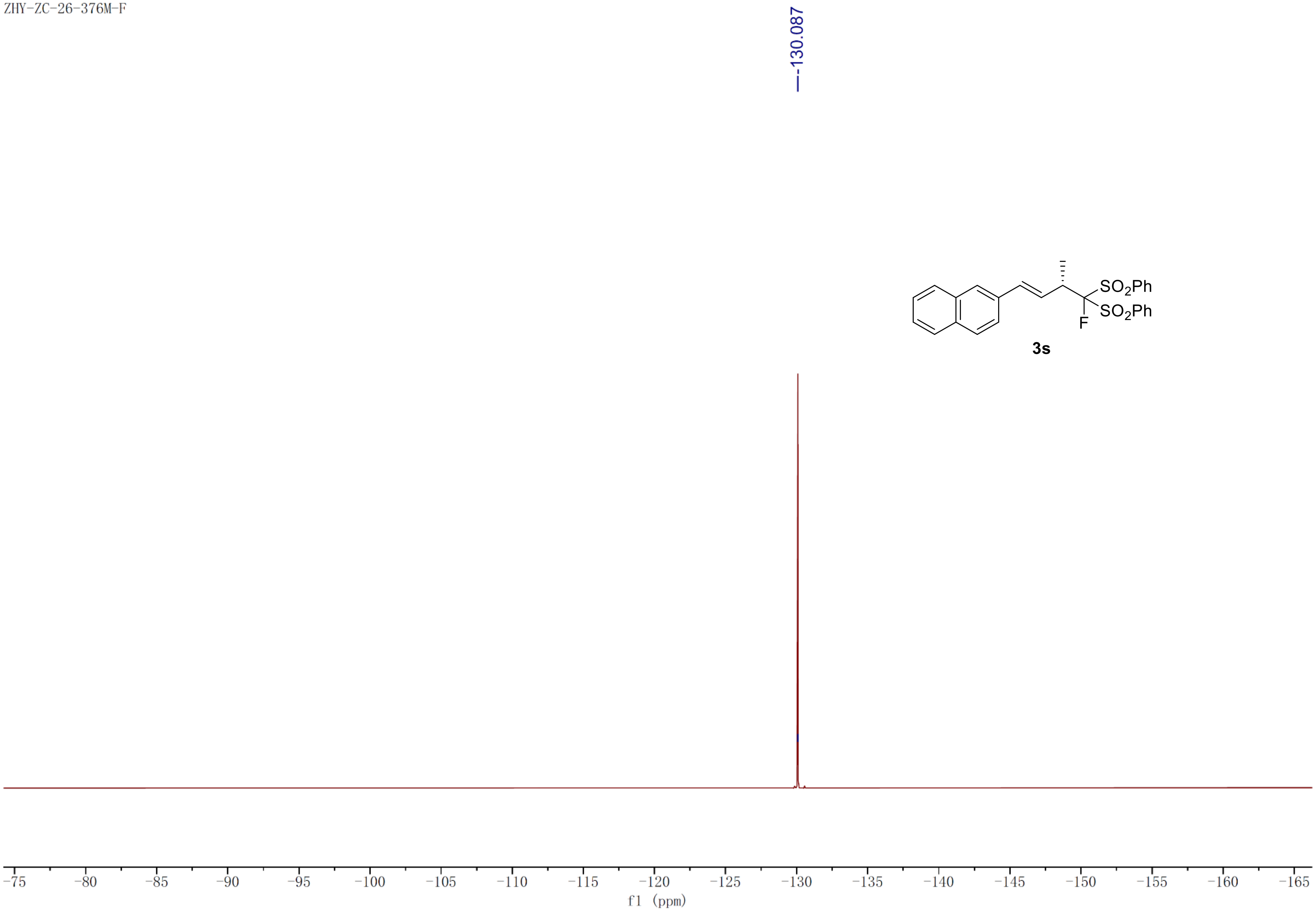

3s

ZHY-ZD-26-400M-H

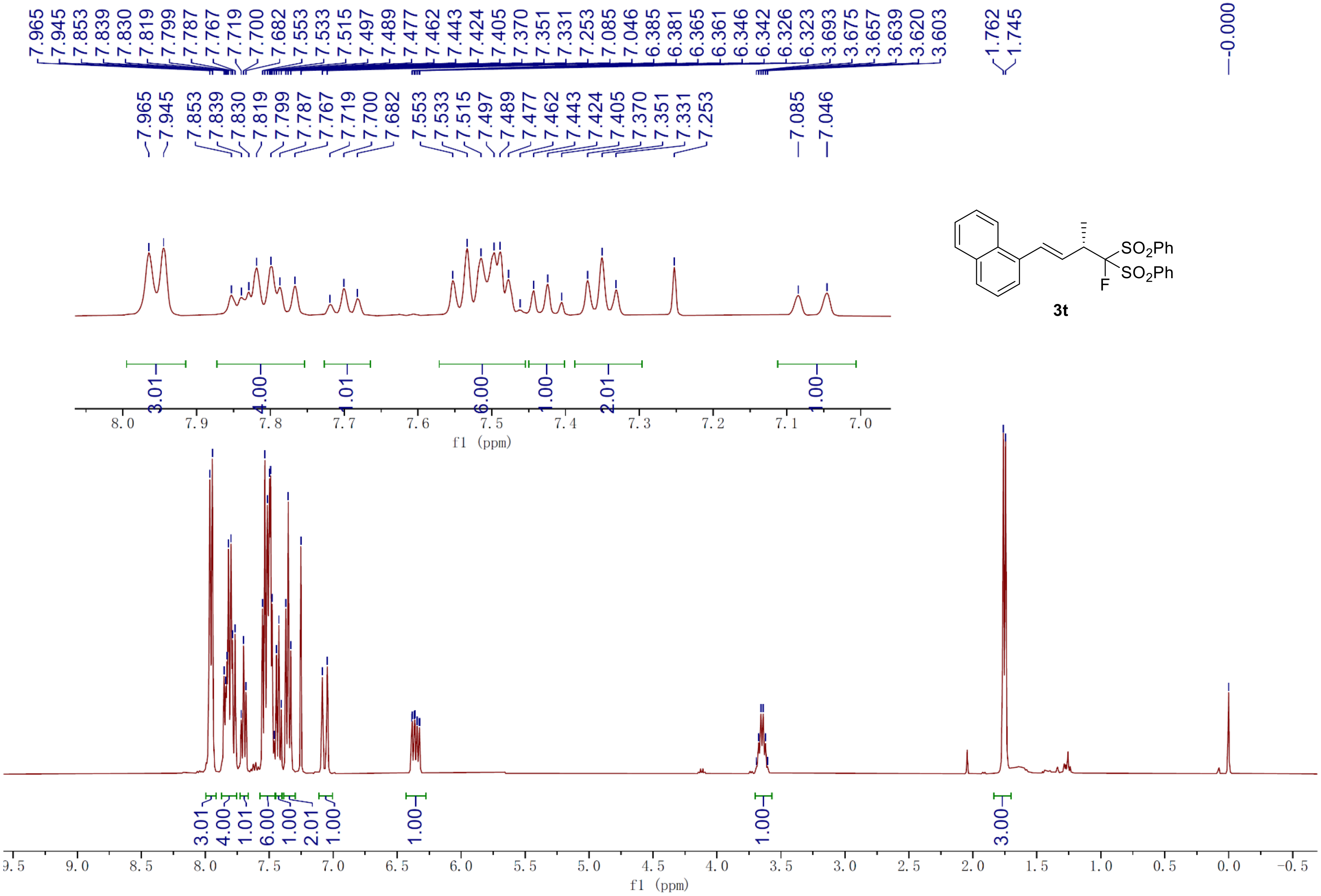

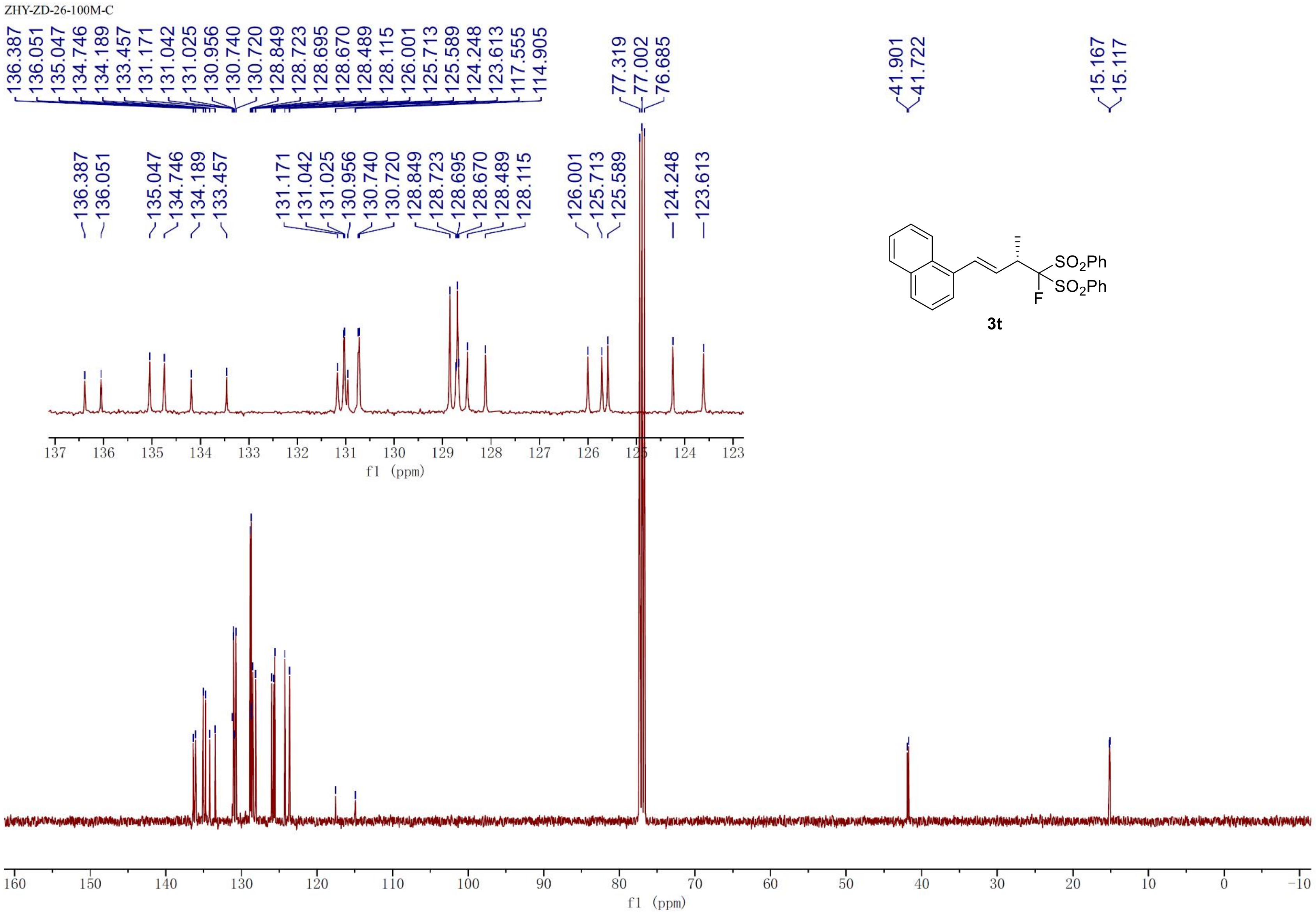

ZHY-ZD-26-100M-C
136.387
136.051
135.047
134.746
134.189
133.457
131.171
131.042
131.025
130.956
130.740
130.720
128.849
128.723
128.695
128.670
128.489
128.115
126.001
125.713
125.589
124.248
123.613
117.555
114.905
77.319
77.002
76.685
41.901
41.722
15.167
15.117
SO2Ph
SO2Ph
F
3t
f1 (ppm)

ZHY-ZD-26-376M-F

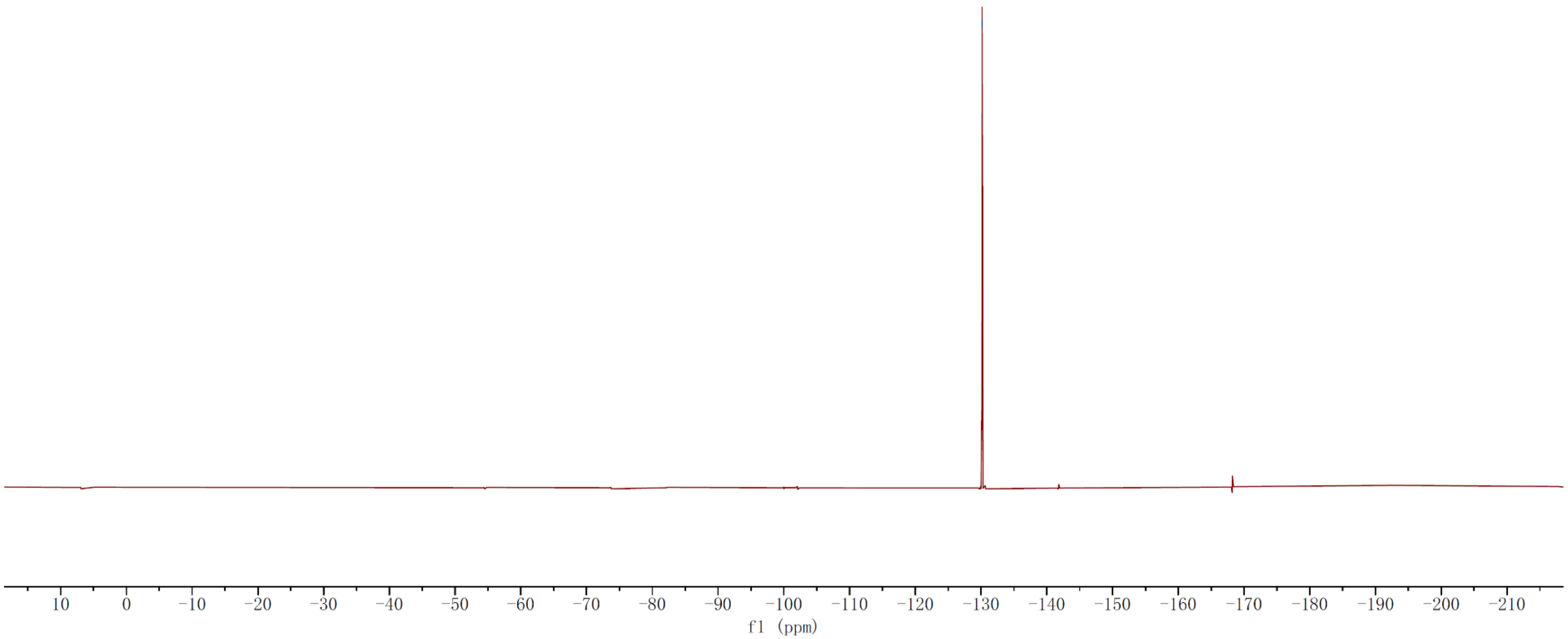

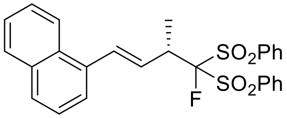

3t

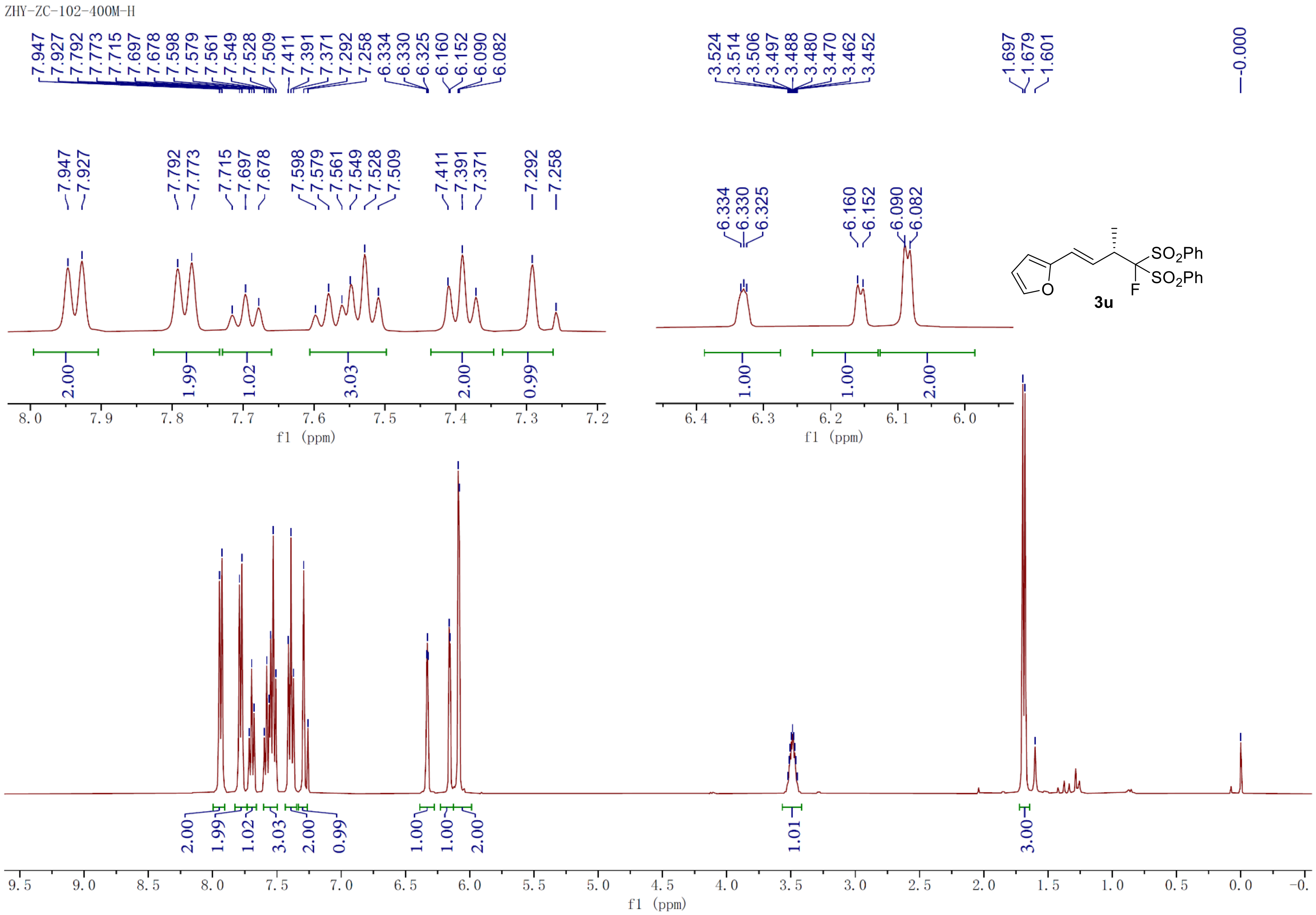
ZHY-ZC-102-400M-H
7.947
7.927
7.792
7.773
7.715
7.697
7.678
7.598
7.579
7.561
7.549
7.528
7.509
7.411
7.391
7.371
7.292
7.258
6.334
6.330
6.325
6.160
6.152
6.090
6.082
3.524
3.514
3.506
3.497
3.488
3.480
3.470
3.462
3.452
1.697
1.679
1.601
-0.000
2.00
1.99
1.02
3.03
2.00
0.99
1.00
1.00
2.00
1.01
3.00
SO2Ph
SO2Ph
F
O
3u
f1 (ppm)

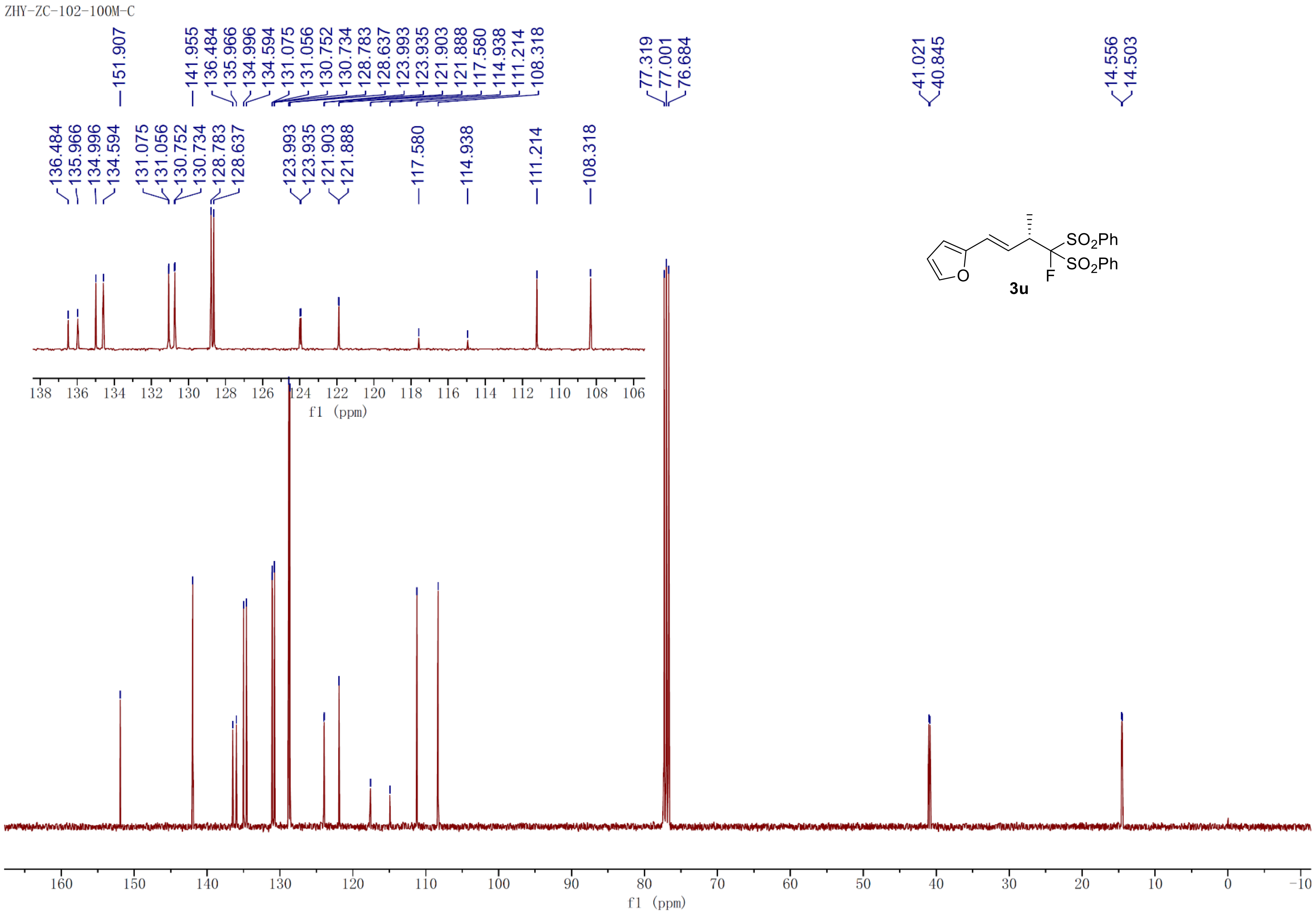
ZHY-ZC-102-100M-C
151.907
141.955
136.484
135.966
134.996
134.594
131.075
131.056
130.752
130.734
128.783
128.637
123.993
123.935
121.903
121.888
117.580
114.938
111.214
108.318
77.319
77.001
76.684
41.021
40.845
14.556
14.503
SO2Ph
SO2Ph
F
O
3u
f1 (ppm)

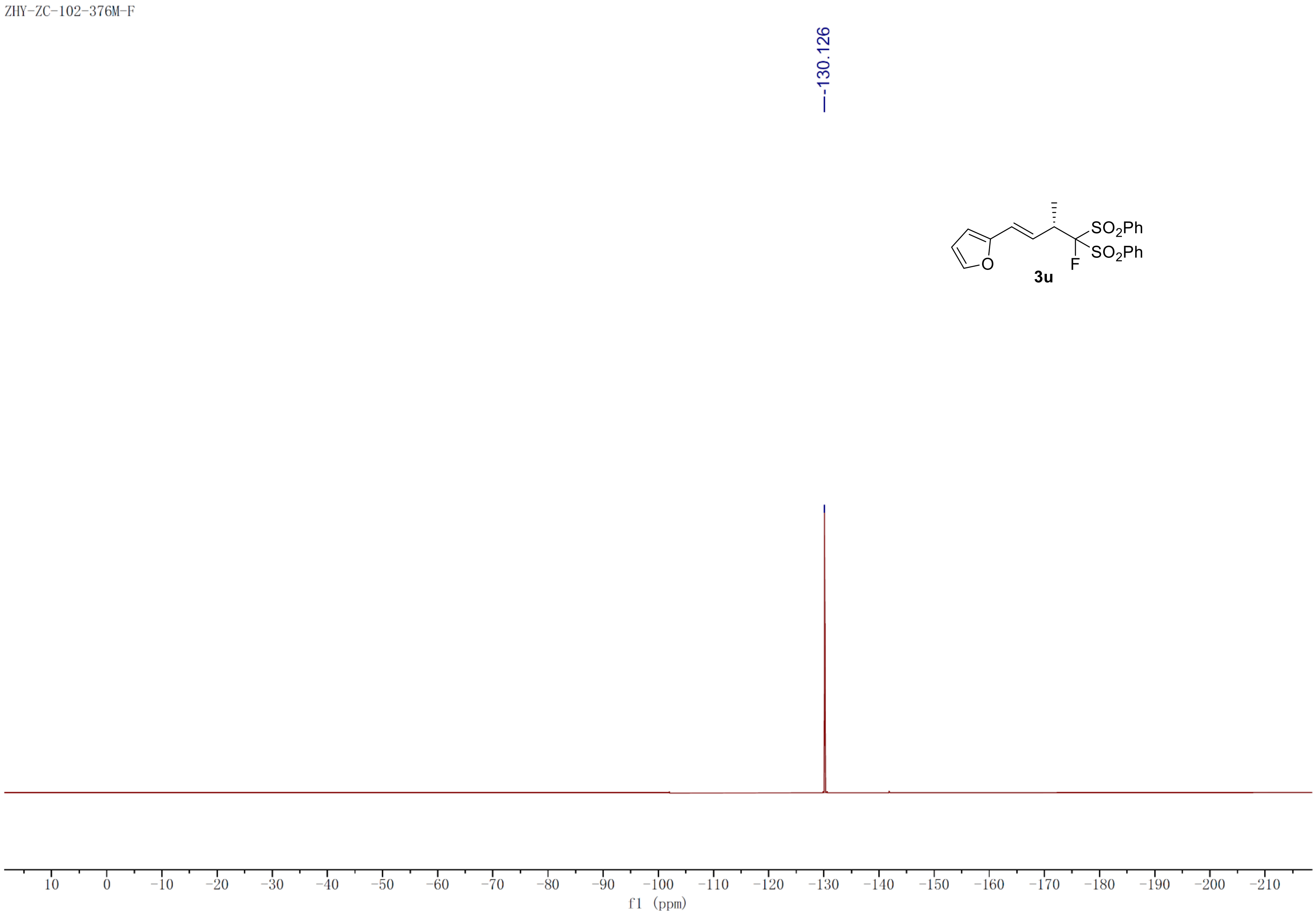
ZHY-ZC-102-376M-F
-130.126
SO2Ph
SO2Ph
F
O
3u
10 0 −10 −20 −30 −40 −50 −60 −70 −80 −90 −100 −110 −120 −130 −140 −150 −160 −170 −180 −190 −200 −210
f1 (ppm)

ZHY-ZC-119-400M-H

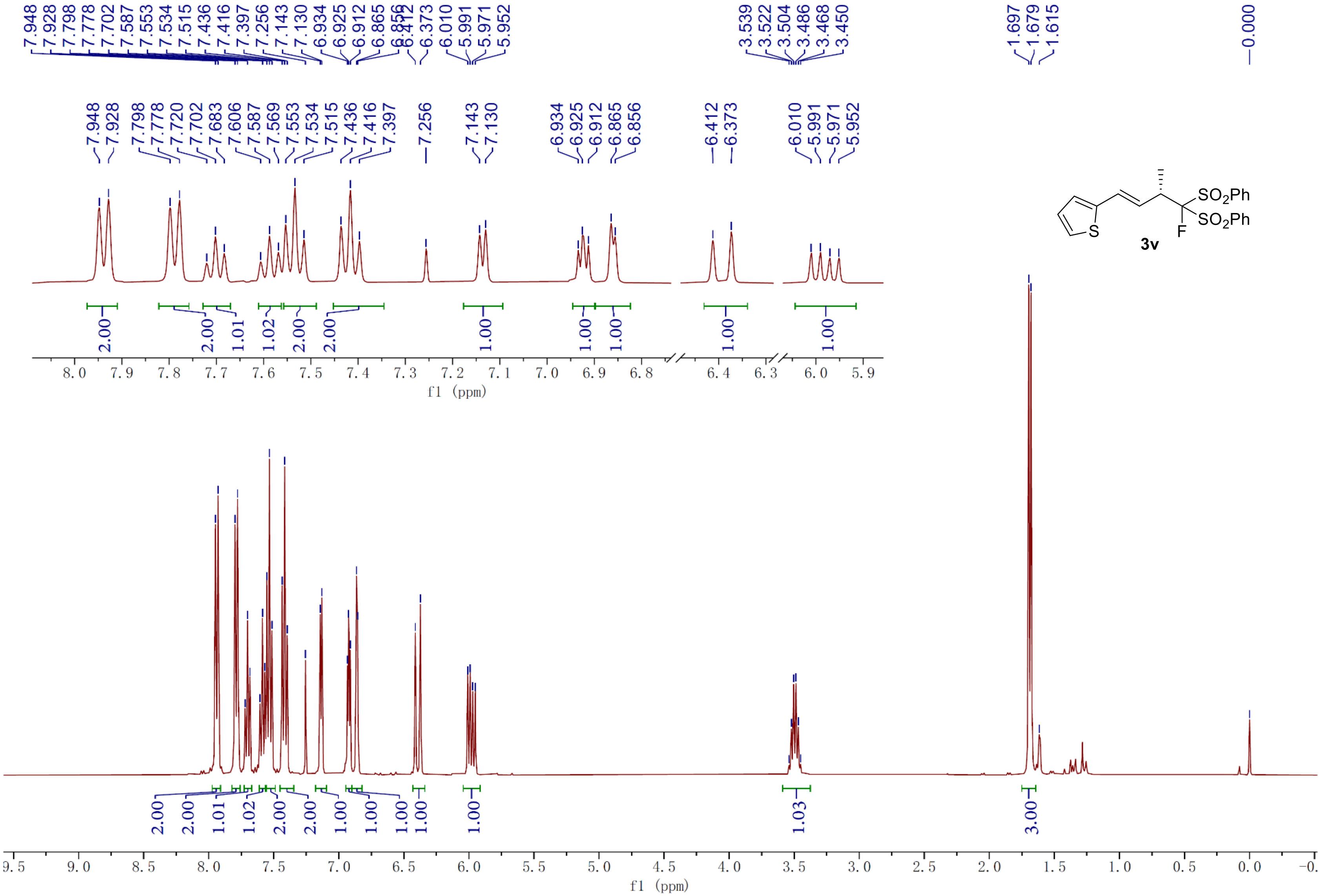

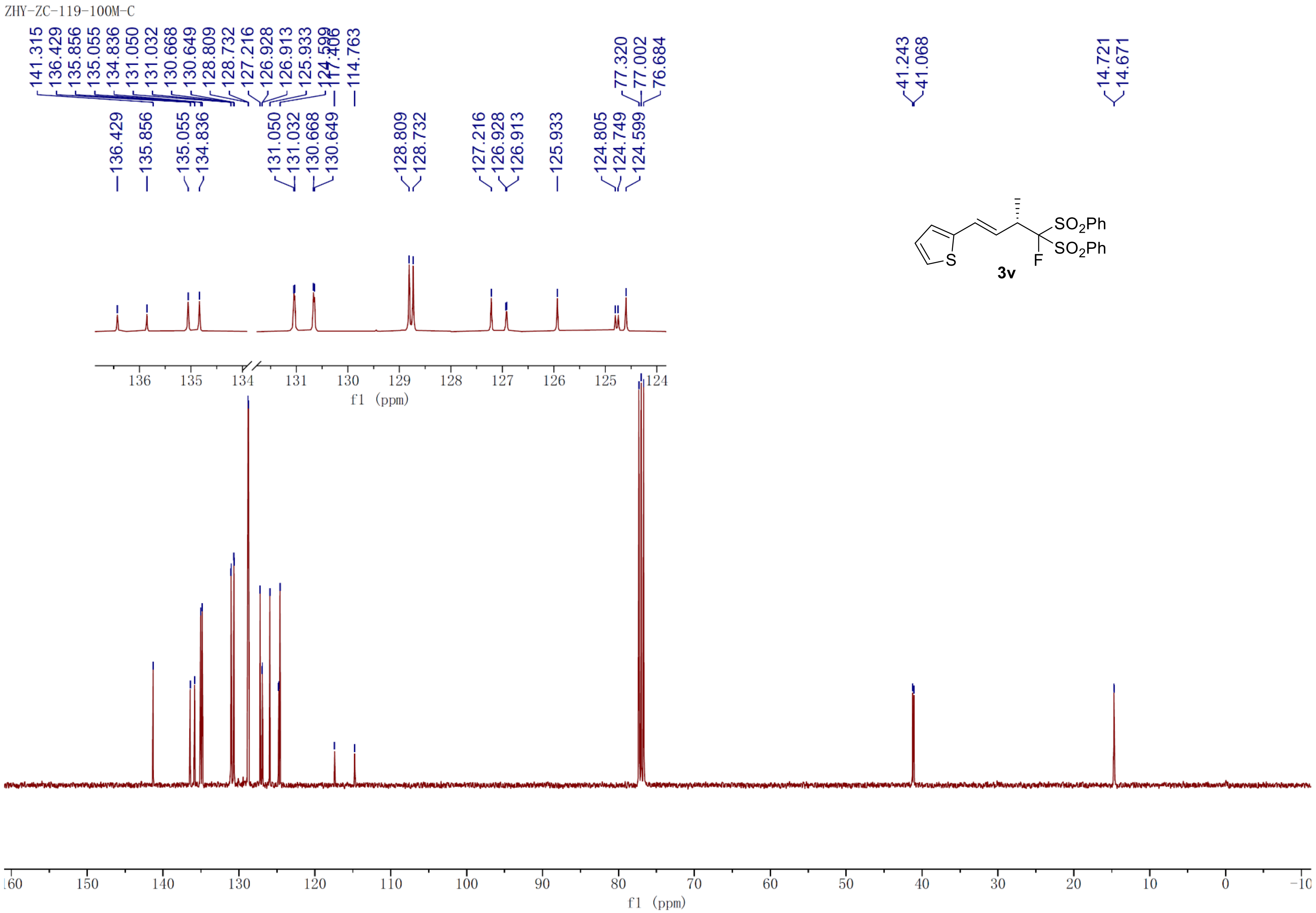
ZHY-ZC-119-100M-C
141.315
136.429
135.856
135.055
134.836
131.050
131.032
130.668
130.649
128.809
128.732
127.216
126.928
126.913
125.933
124.599
117.406
114.763
77.320
77.002
76.684
41.243
41.068
14.721
14.671
124.805
124.749
SO2Ph
SO2Ph
F
S
3v
f1 (ppm)

ZHY-ZC-119-376M-F

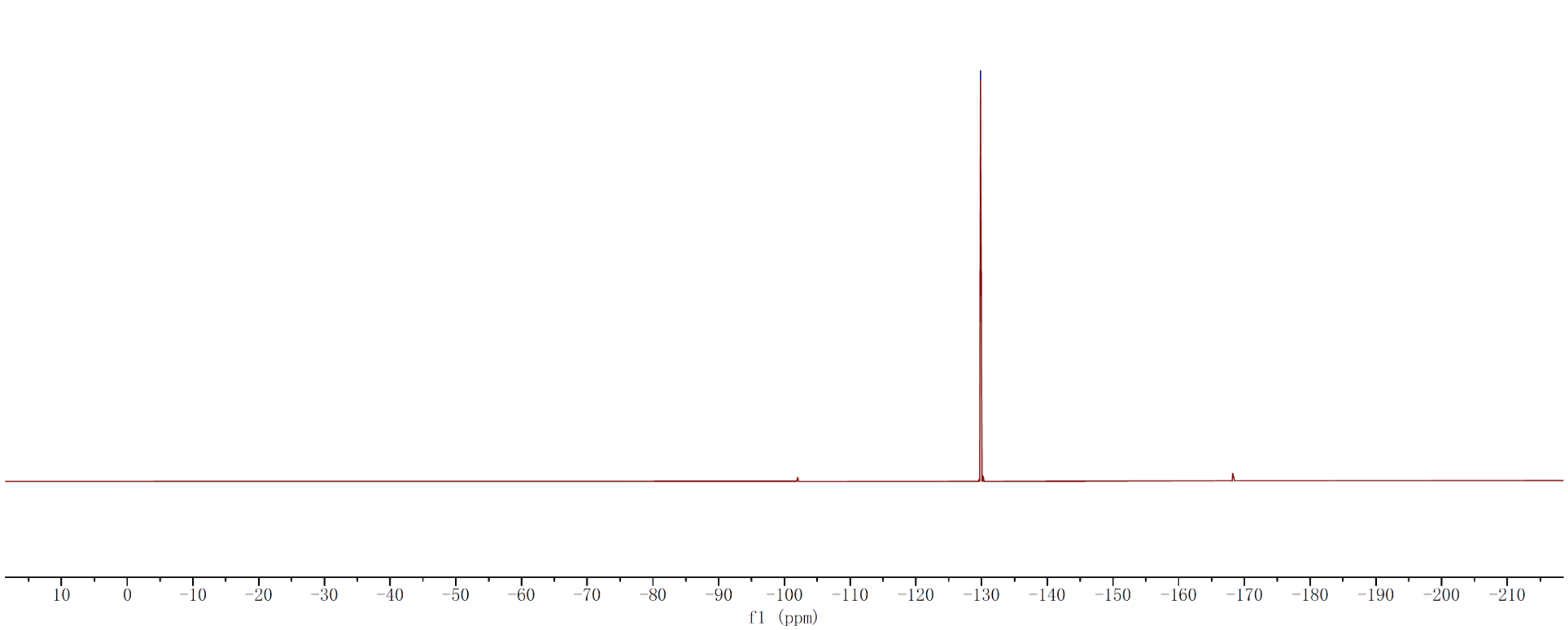

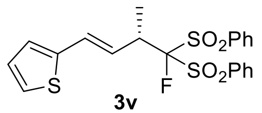

3v

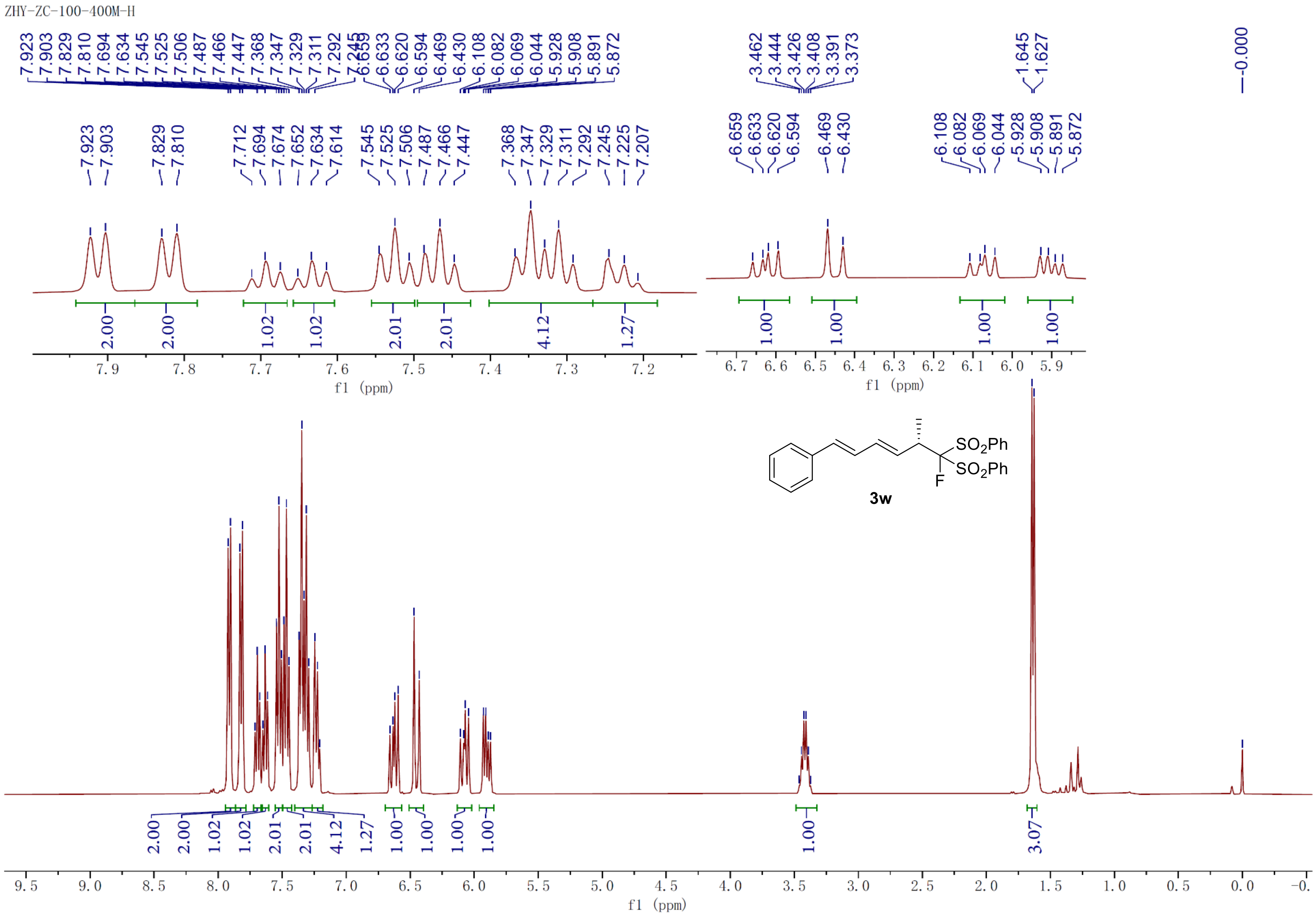
ZHY-ZC-100-400M-H
SO2Ph
SO2Ph
F
3w
f1 (ppm)

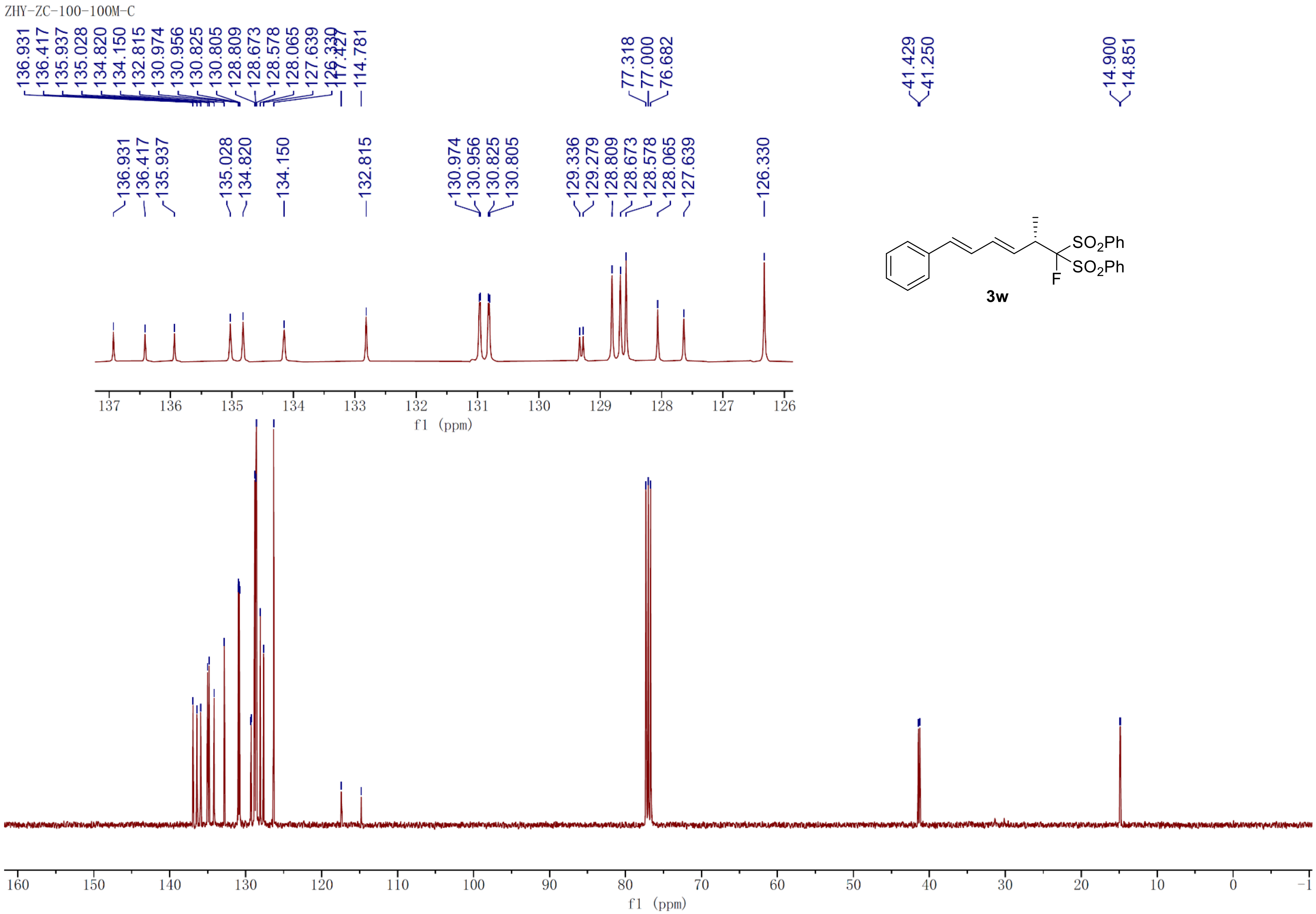
ZHY-ZC-100-100M-C
136.931
136.417
135.937
135.028
134.820
134.150
132.815
130.974
130.956
130.825
130.805
128.809
128.673
128.578
128.065
127.639
126.330
117.427
114.781
77.318
77.000
76.682
41.429
41.250
14.900
14.851
129.336
129.279
SO2Ph
SO2Ph
F
3w
f1 (ppm)

ZHY-ZC-100-376M-F

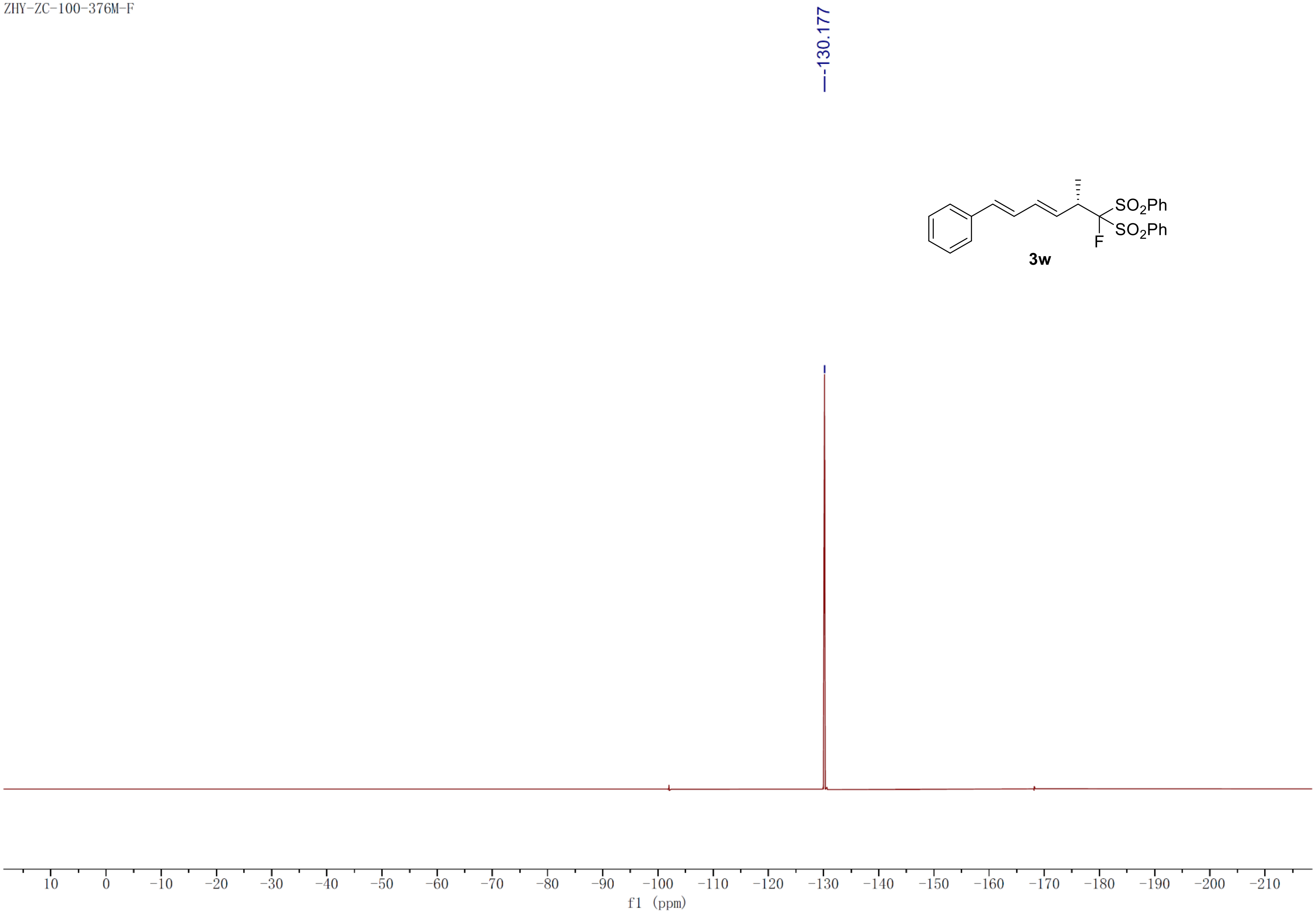

ZHY-ZC-126-400M-H

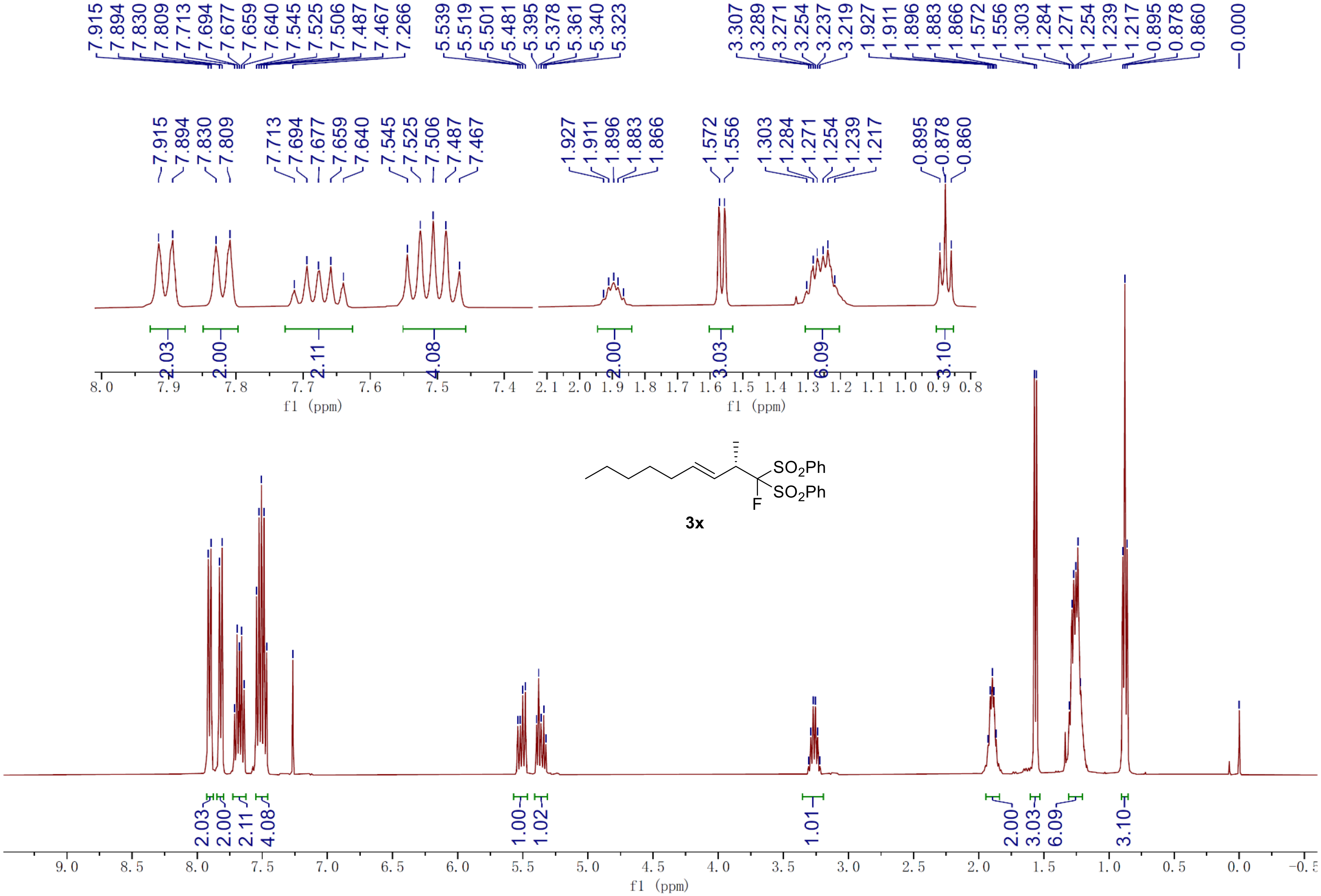

ZHY-ZC-126-100M-C

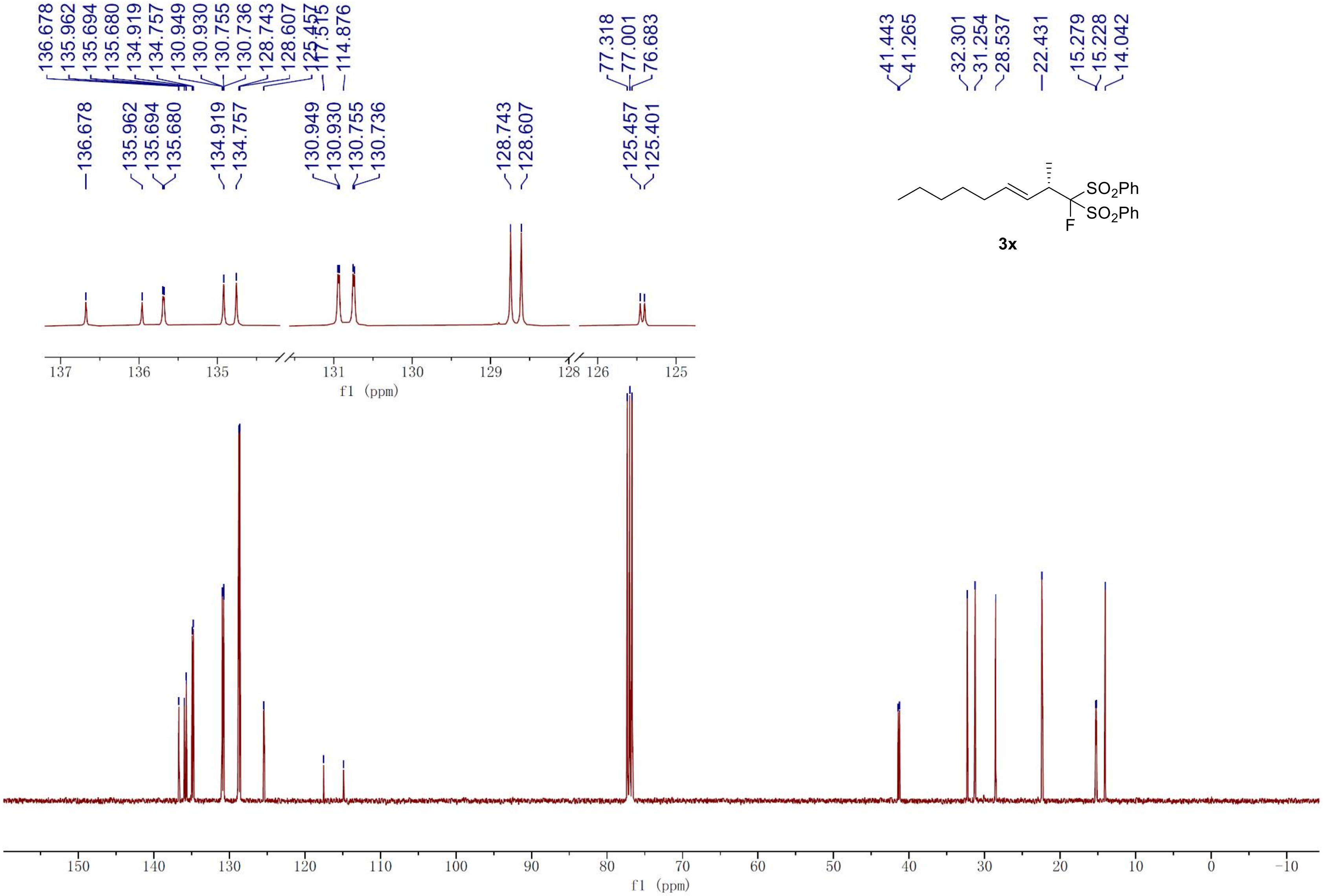

3x

ZHY-ZC-126-376M-F

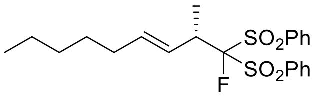

3x

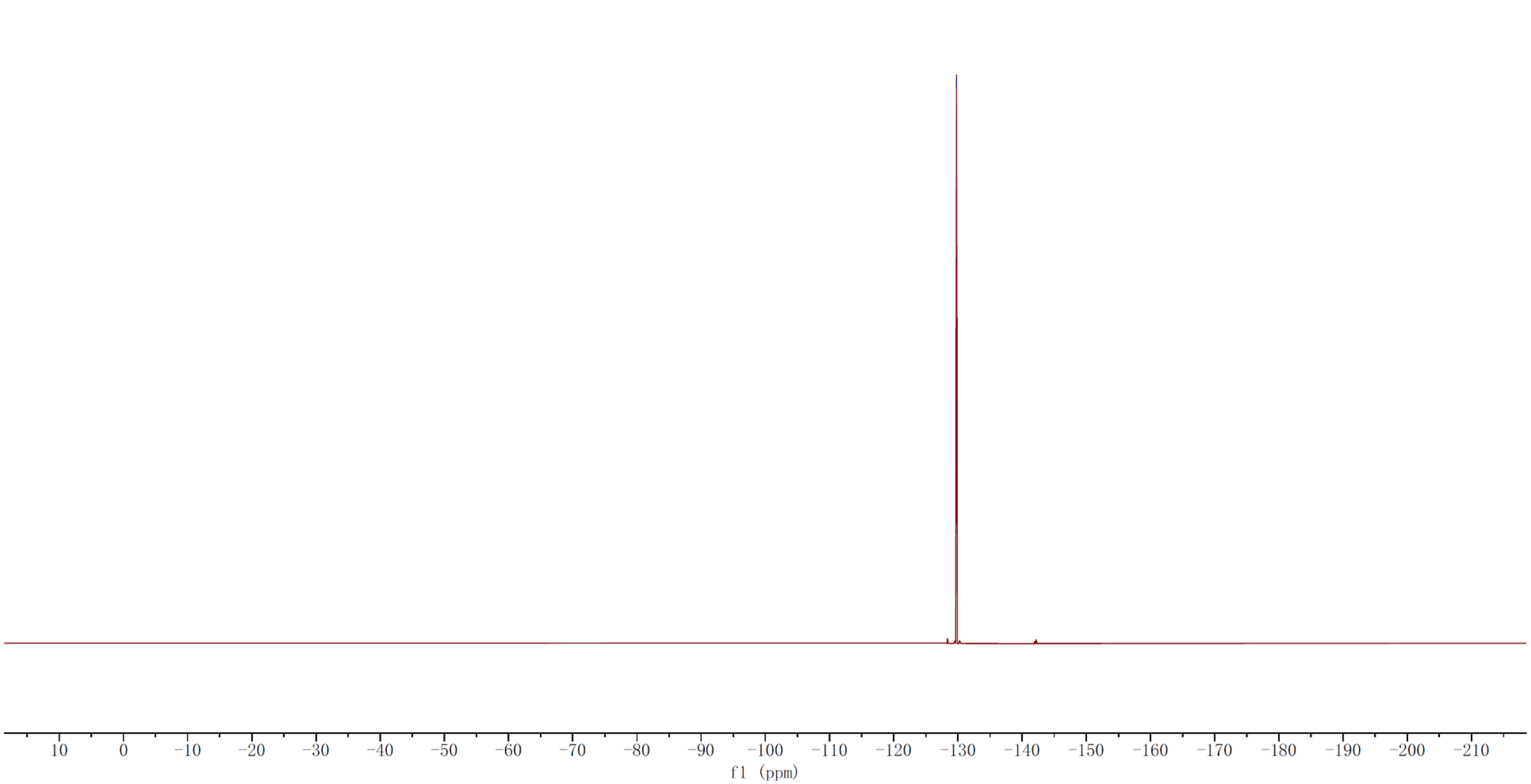

ZHY-ZC-135-400M-H

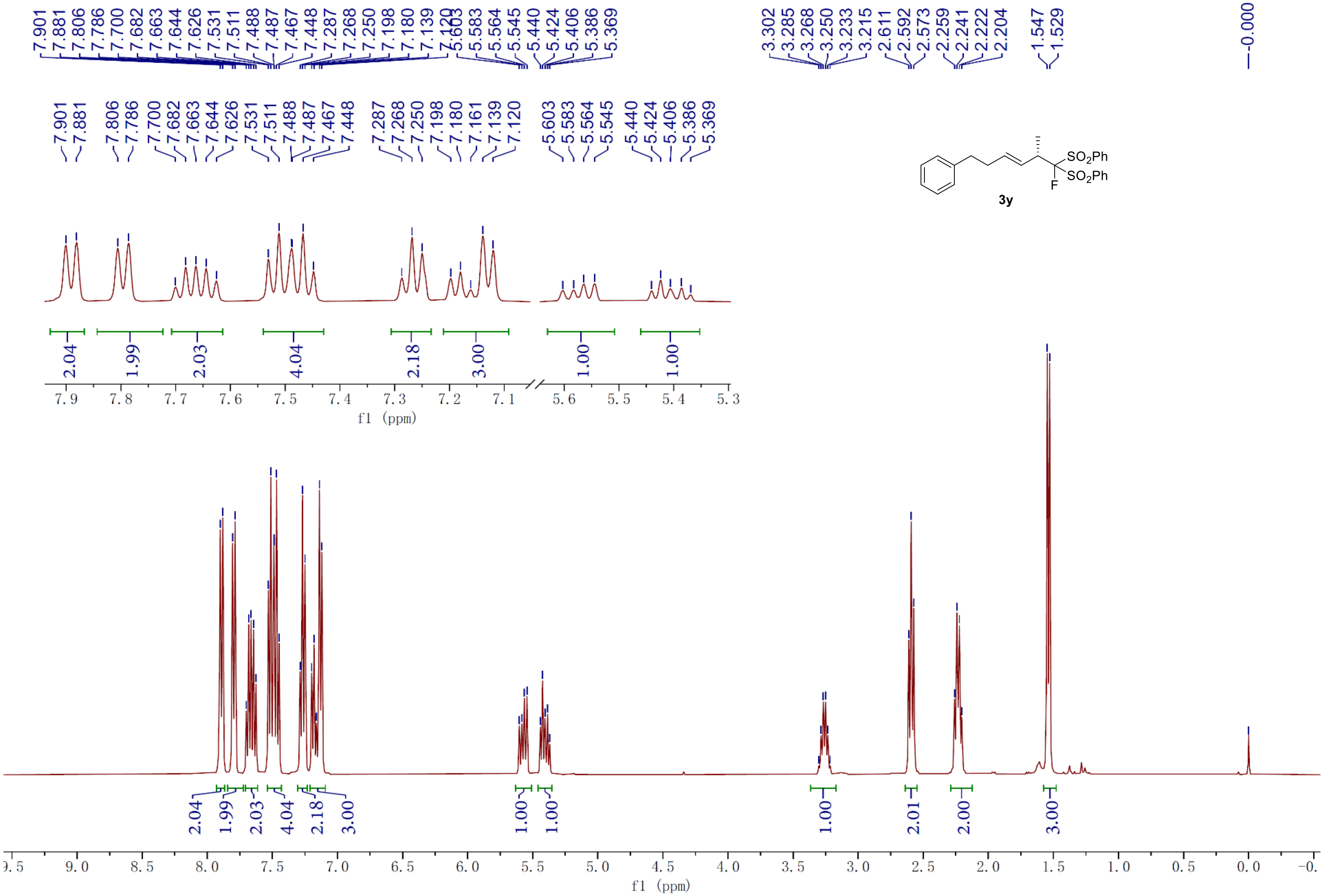

3y

ZHY-ZC-135-100M-C

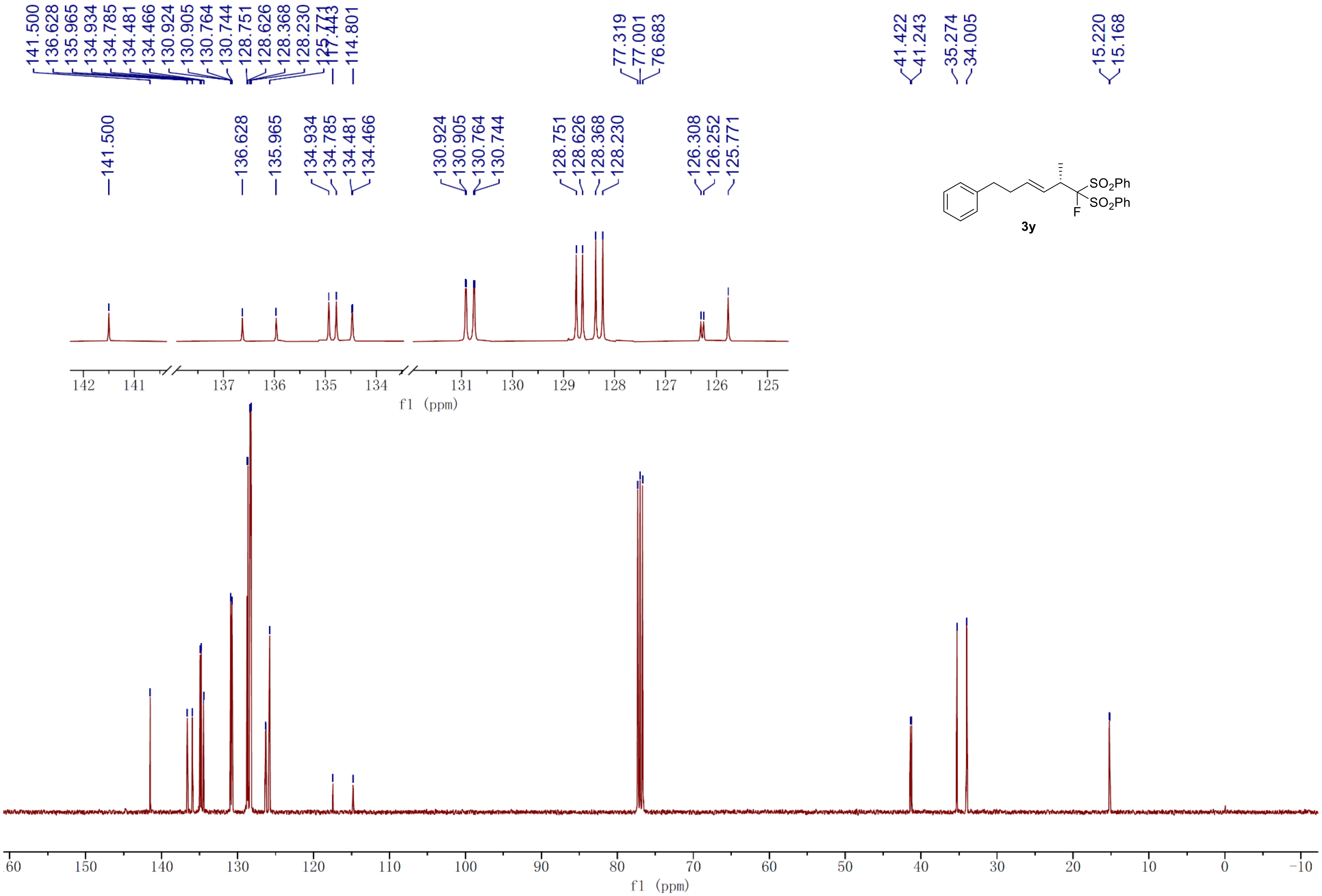

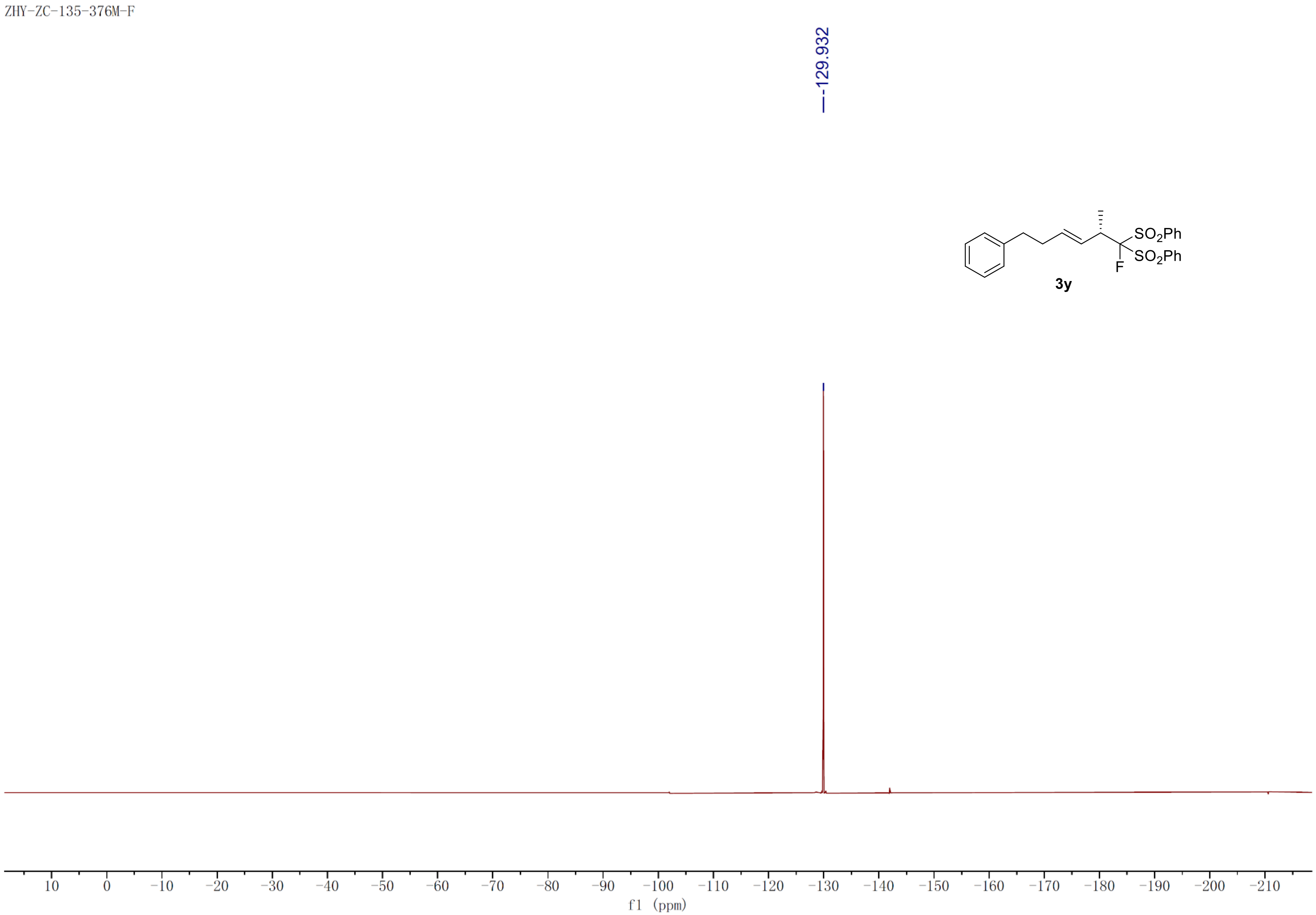
ZHY-ZC-135-376M-F
-129.932
SO2Ph
SO2Ph
F
3y
10
0
-10
-20
-30
-40
-50
-60
-70
-80
-90
-100
-110
-120
-130
-140
-150
-160
-170
-180
-190
-200
-210
f1 (ppm)

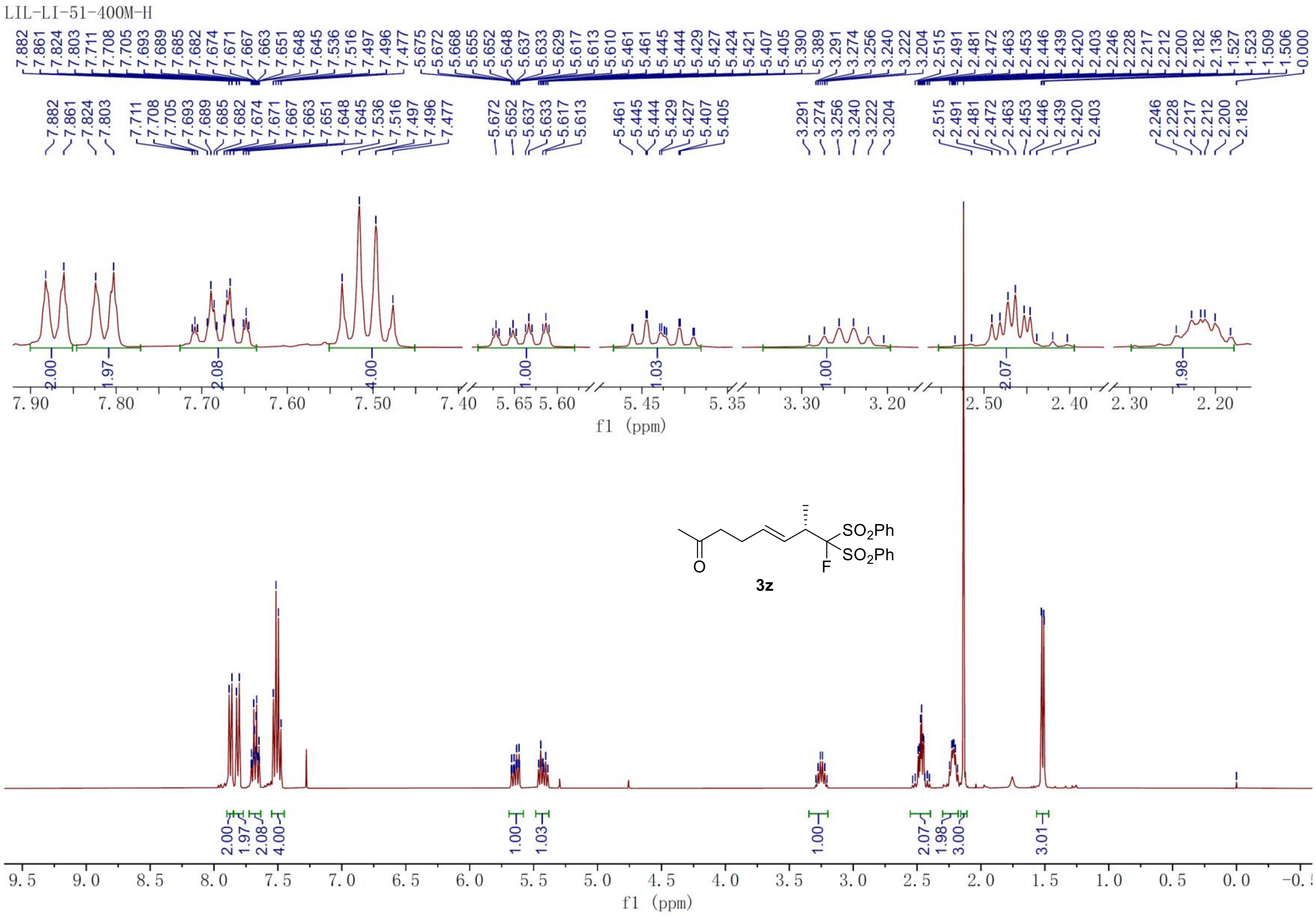
LIL-LI-51-400M-H
f1 (ppm)
SO2Ph
SO2Ph
F
O
3z

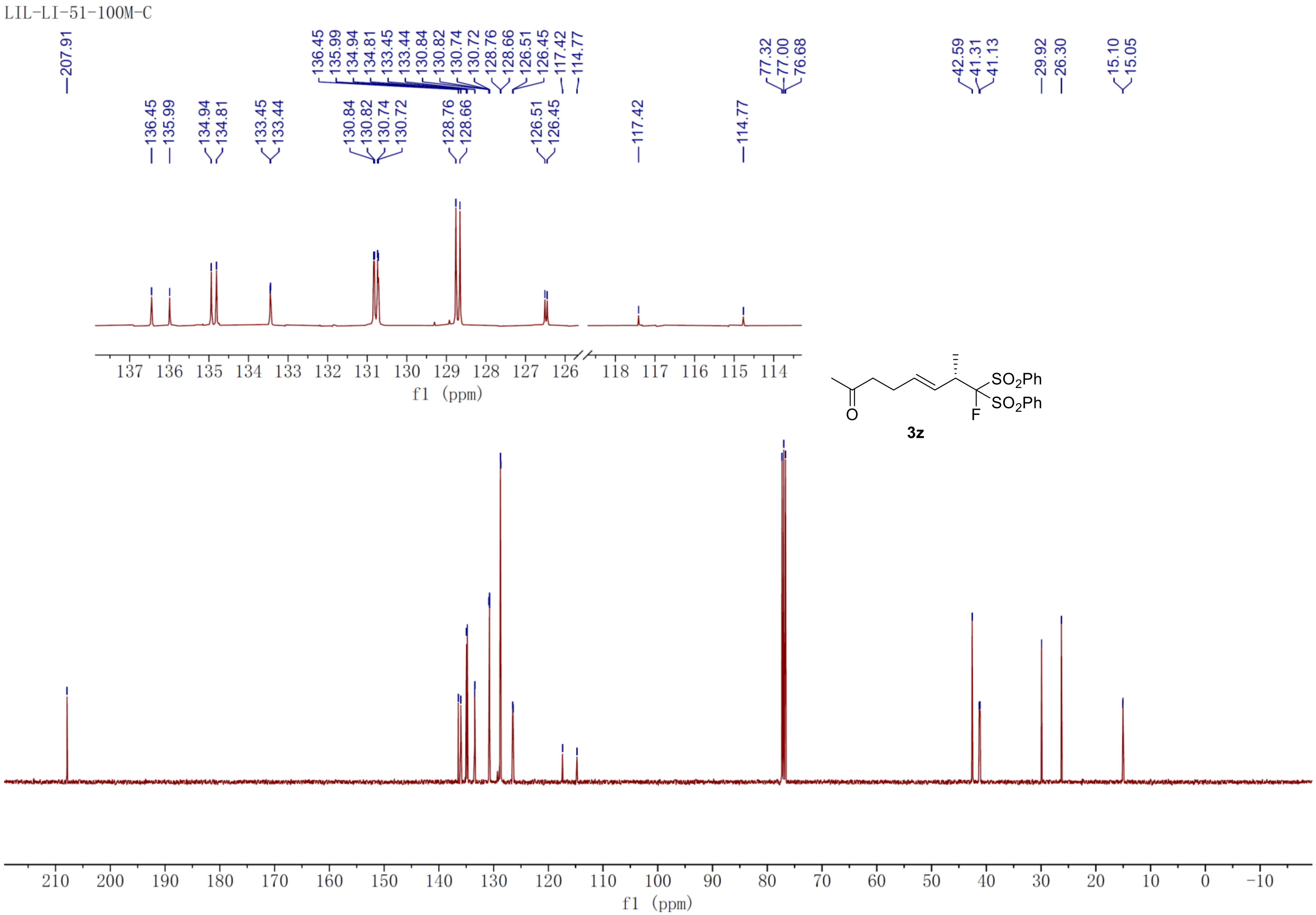
LIL-LI-51-100M-C
207.91
136.45
135.99
134.94
134.81
133.45
133.44
130.84
130.82
130.74
130.72
128.76
128.66
126.51
126.45
117.42
114.77
77.32
77.00
76.68
42.59
41.31
41.13
29.92
26.30
15.10
15.05
f1 (ppm)
SO2Ph
SO2Ph
O
F
3z

LIL-LI-51-376M-F

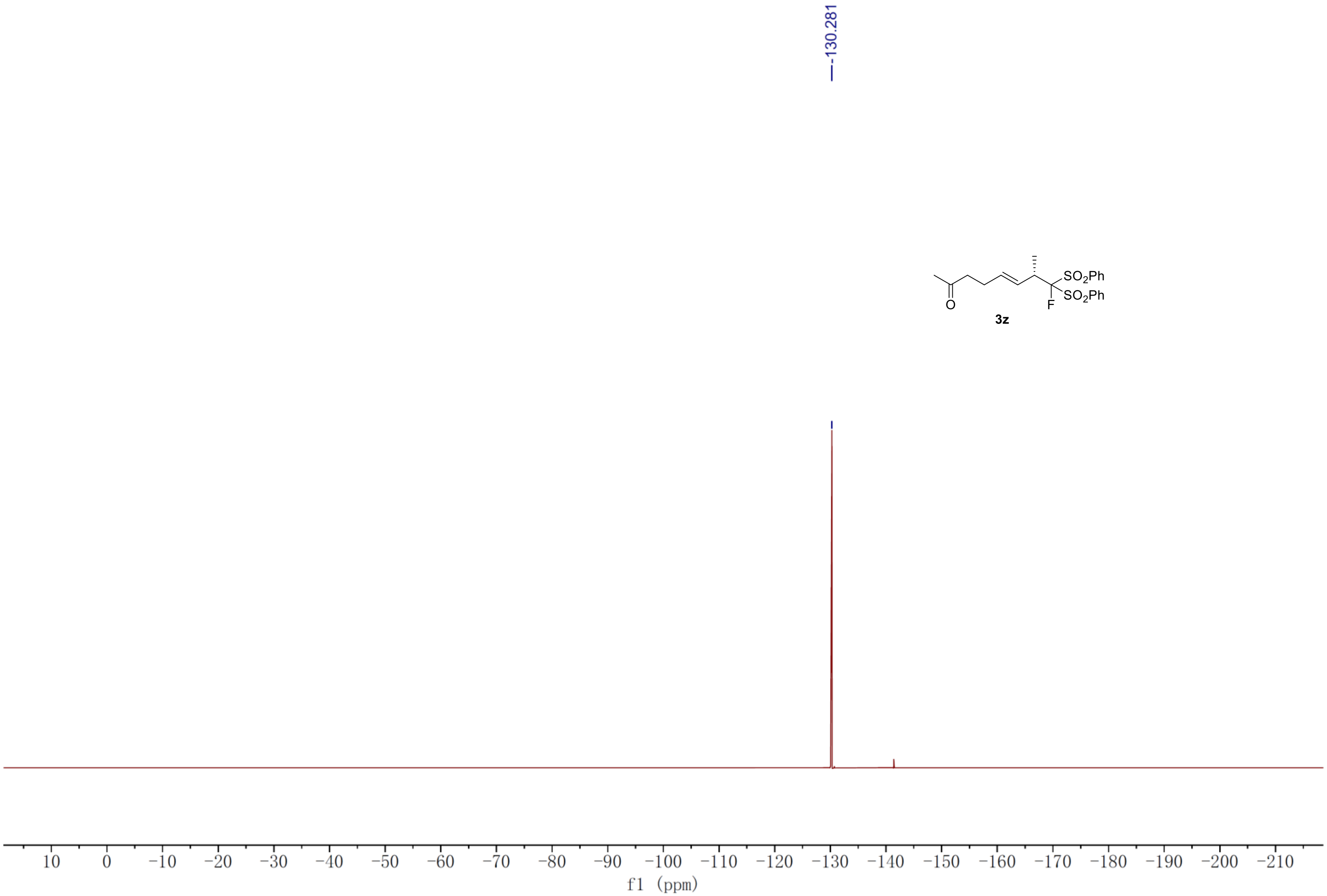

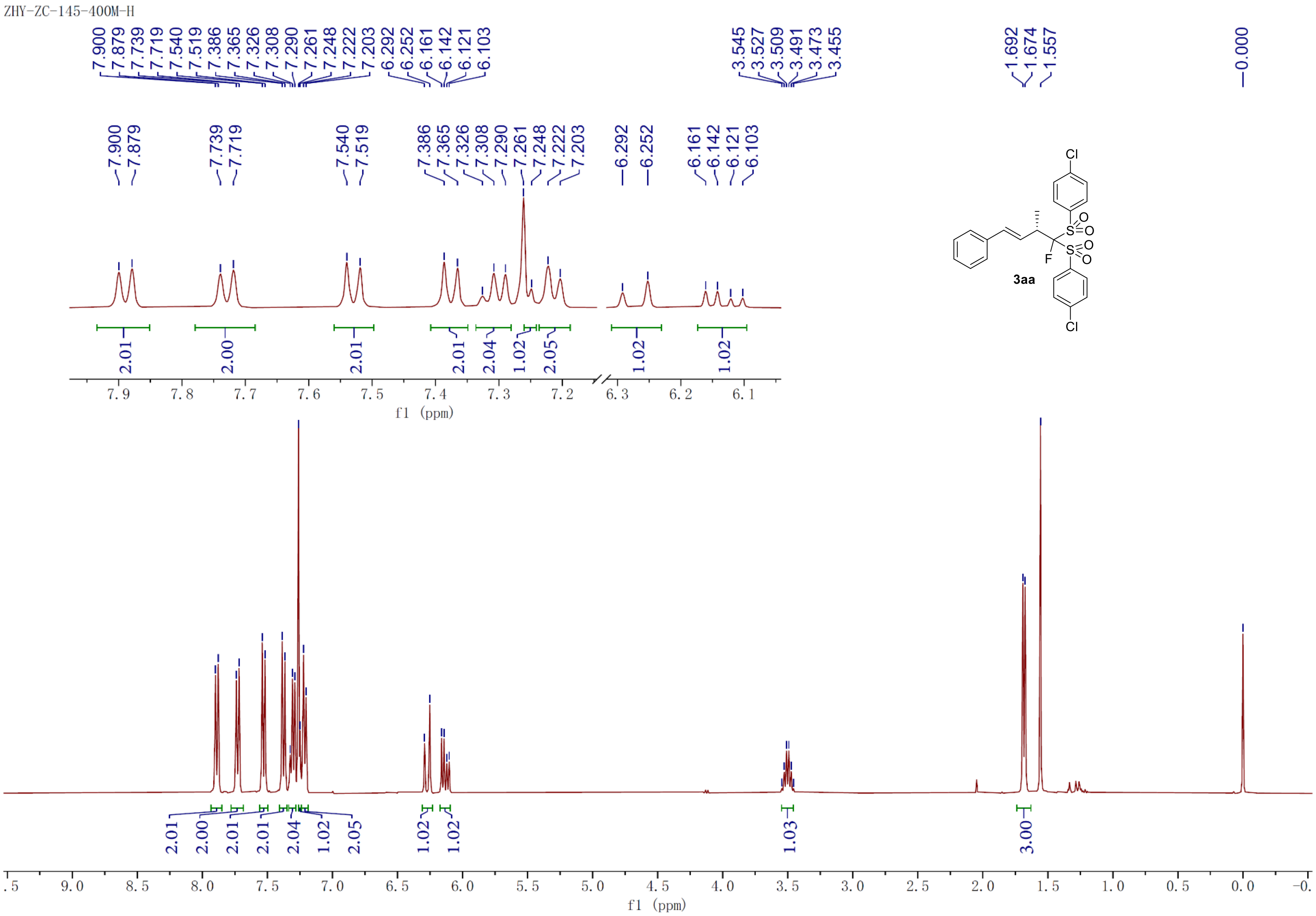
ZHY-ZC-145-400M-H
3aa
f1 (ppm)

ZHY-ZC-145-100M-C

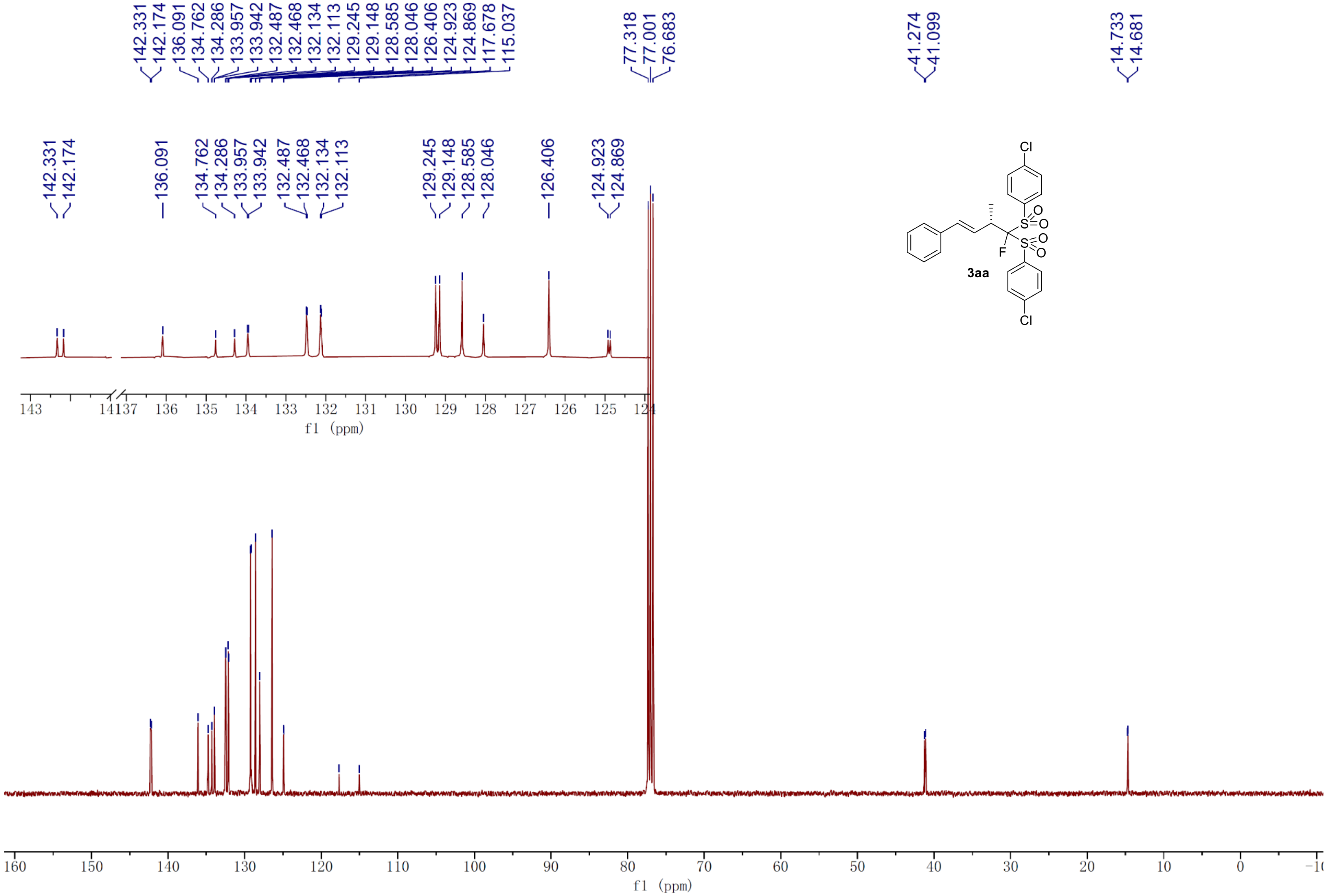

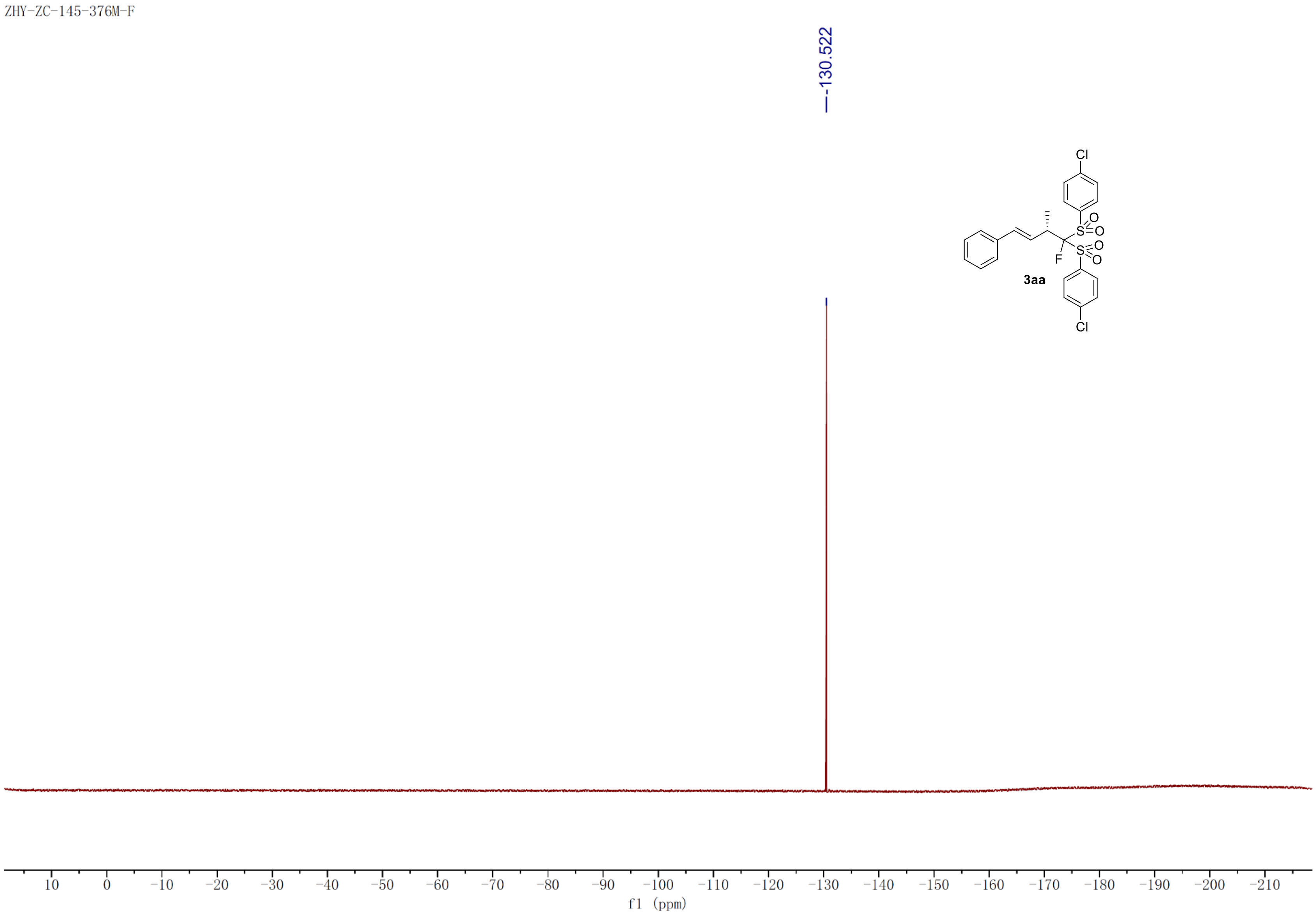
ZHY-ZC-145-376M-F
-130.522
Cl
S
O
O
S
O
O
F
3aa
Cl
10
0
-10
-20
-30
-40
-50
-60
-70
-80
-90
-100
-110
-120
-130
-140
-150
-160
-170
-180
-190
-200
-210
f1 (ppm)

ZHY-ZC-146-400M-H

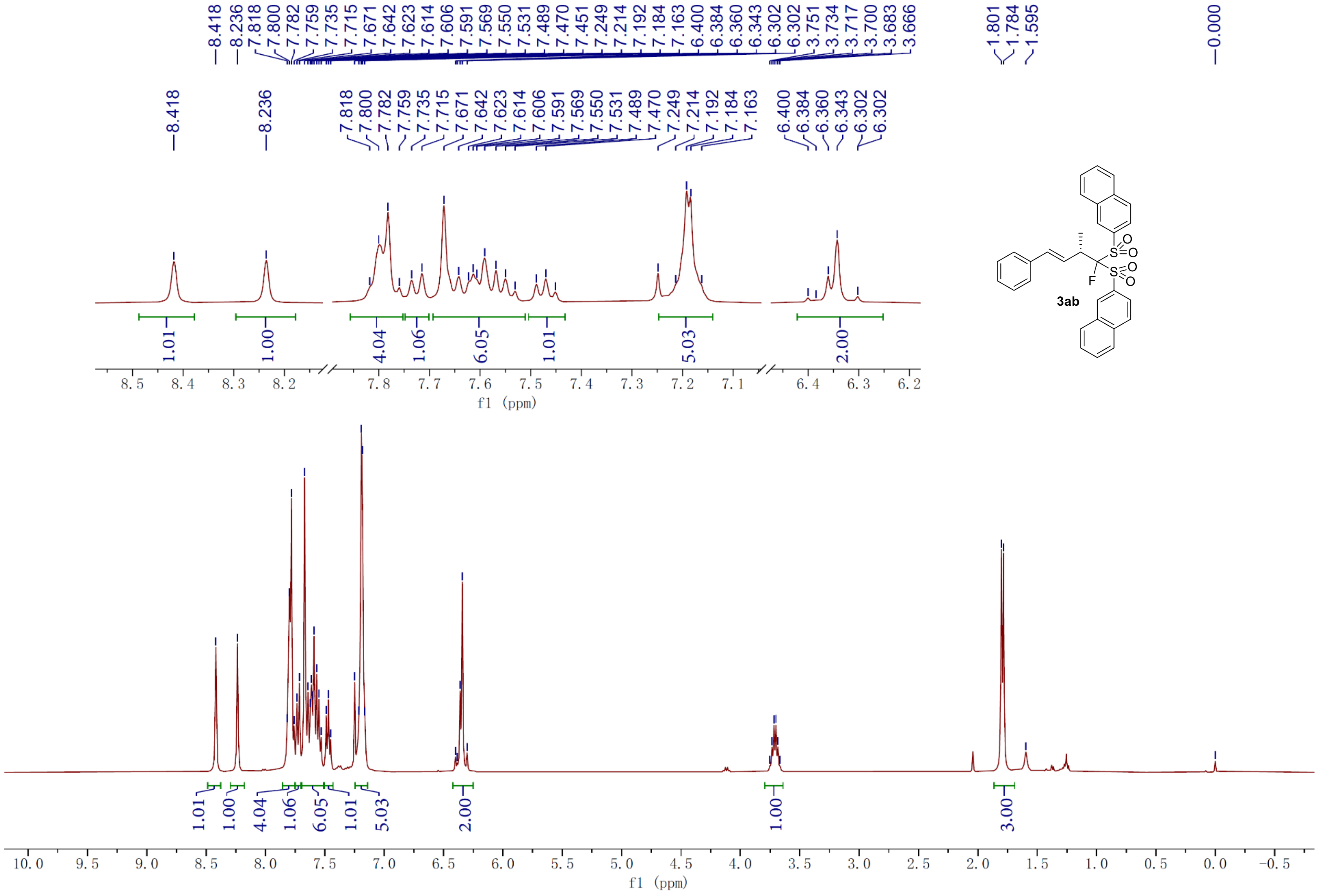

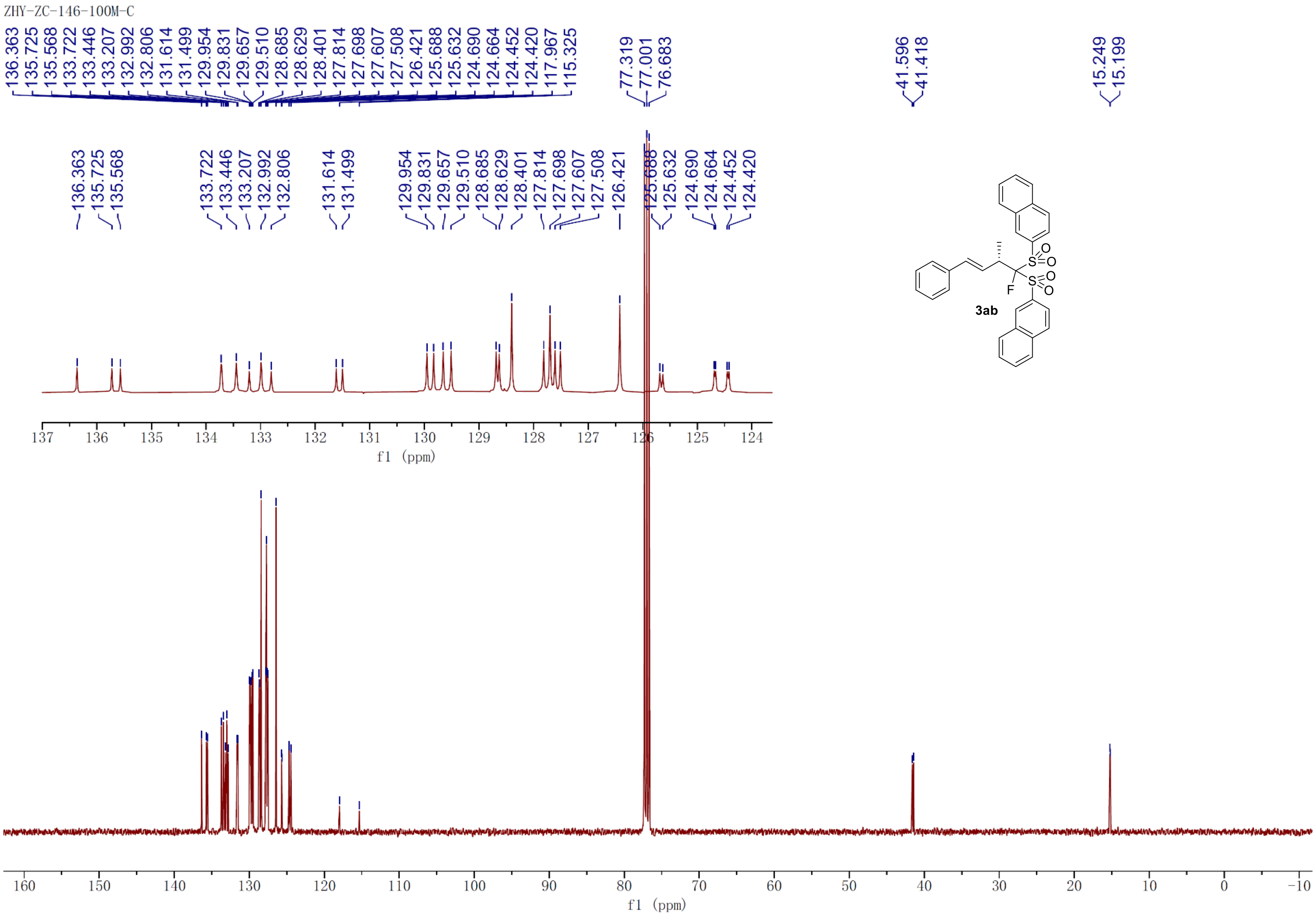
ZHY-ZC-146-100M-C
136.363
135.725
135.568
133.722
133.446
133.207
132.992
132.806
131.614
131.499
129.954
129.831
129.657
129.510
128.685
128.629
128.401
127.814
127.698
127.607
127.508
126.421
125.688
125.632
124.690
124.664
124.452
124.420
117.967
115.325
77.319
77.001
76.683
41.596
41.418
15.249
15.199
3ab
f1 (ppm)

ZHY-ZC-146-376M-F

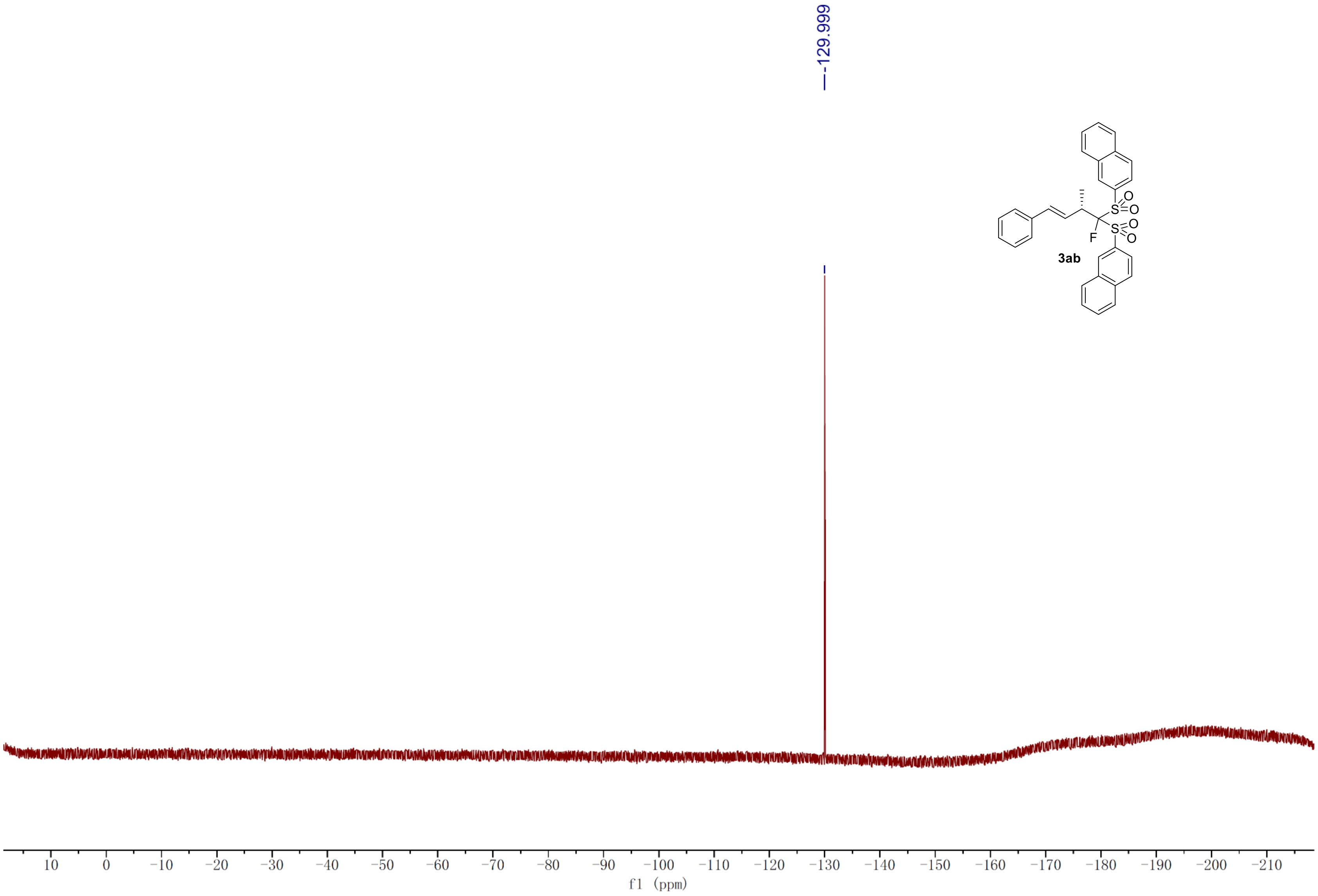

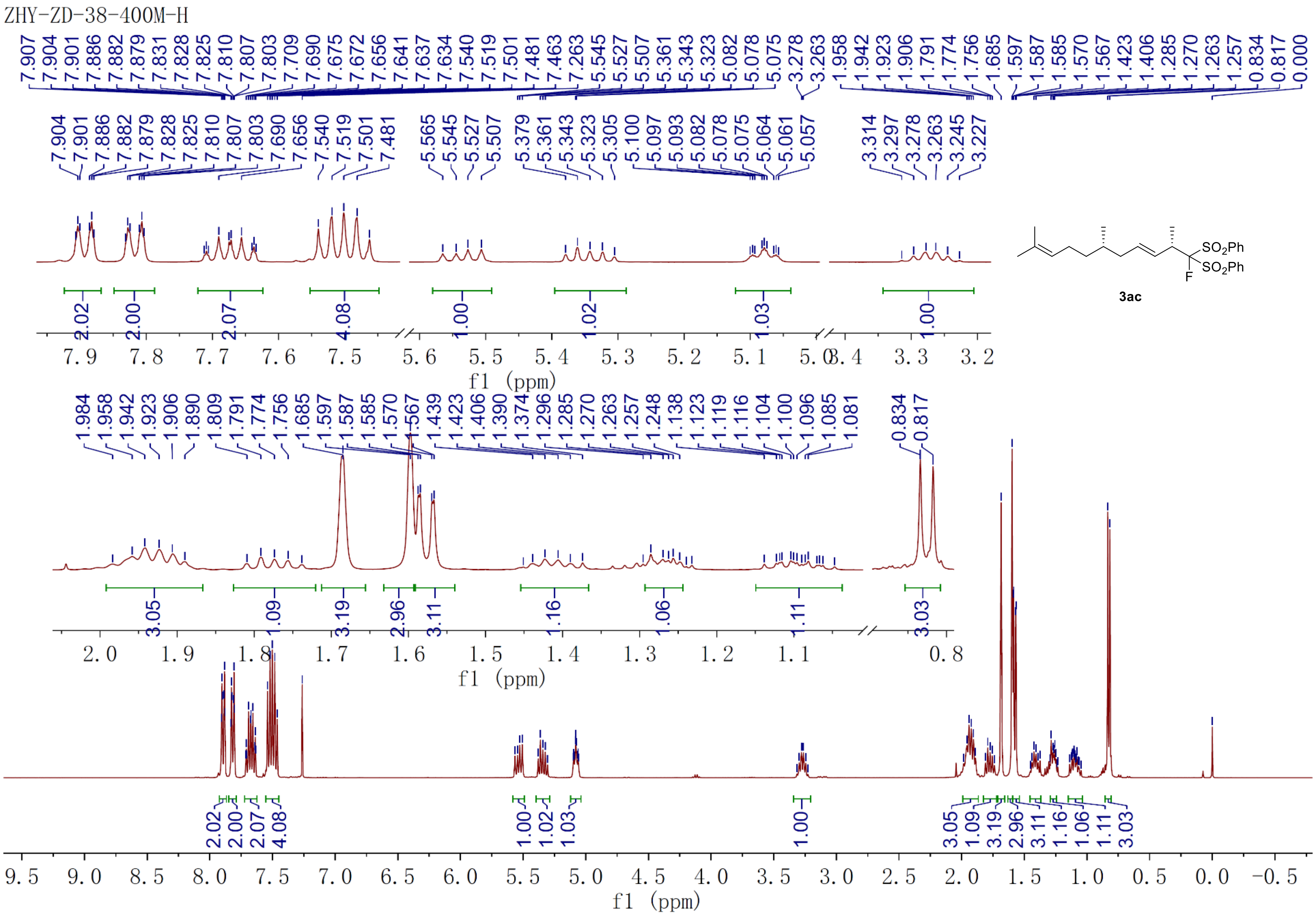
ZHY-ZD-38-400M-H
SO2Ph
SO2Ph
F
3ac
f1 (ppm)

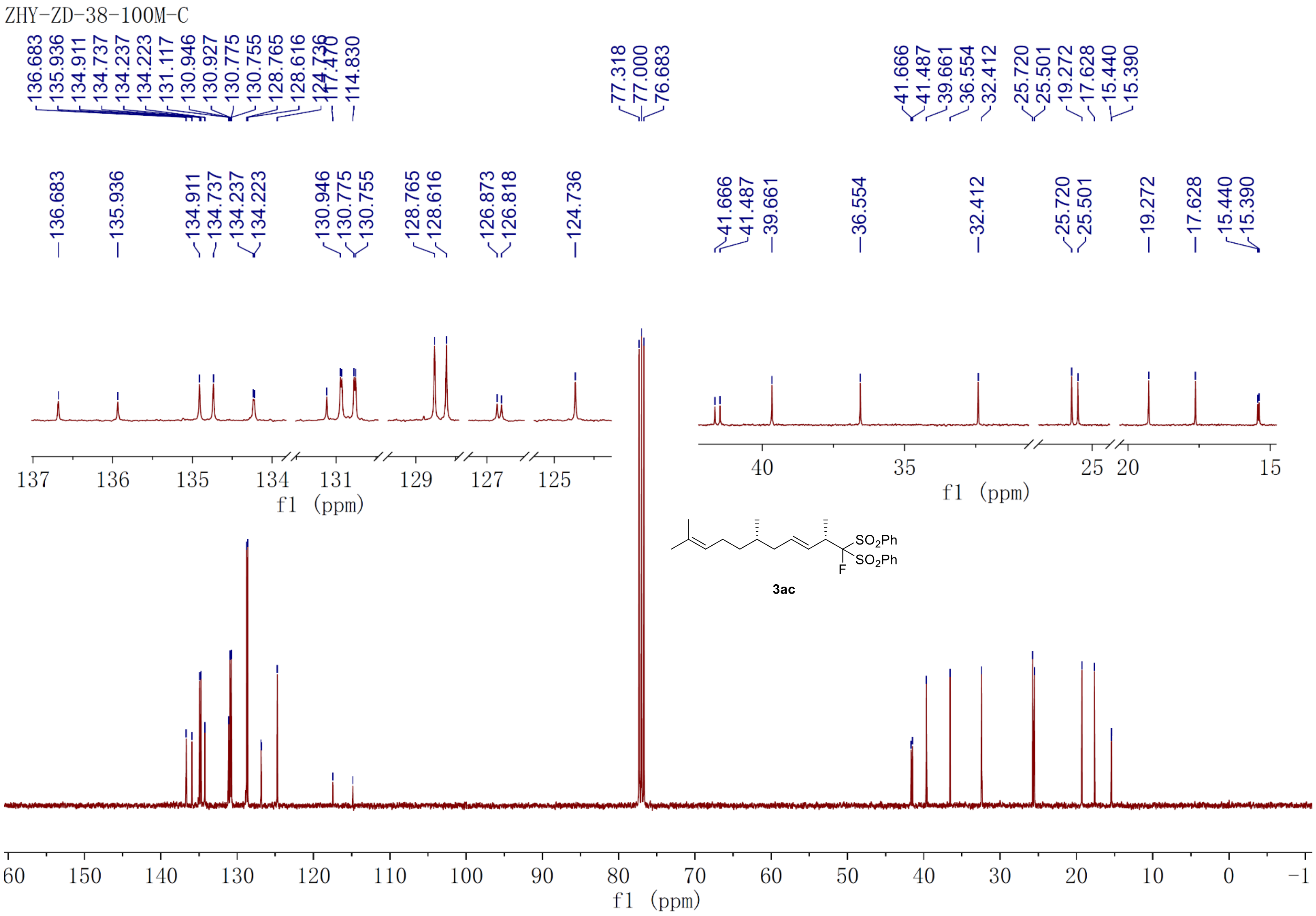
ZHY-ZD-38-100M-C
136.683
135.936
134.911
134.737
134.237
134.223
131.117
130.946
130.927
130.775
130.755
128.765
128.616
124.736
117.470
114.830
77.318
77.000
76.683
41.666
41.487
39.661
36.554
32.412
25.720
25.501
19.272
17.628
15.440
15.390
126.873
126.818
SO2Ph
SO2Ph
F
3ac
f1 (ppm)

ZHY-ZD-38-376M-F

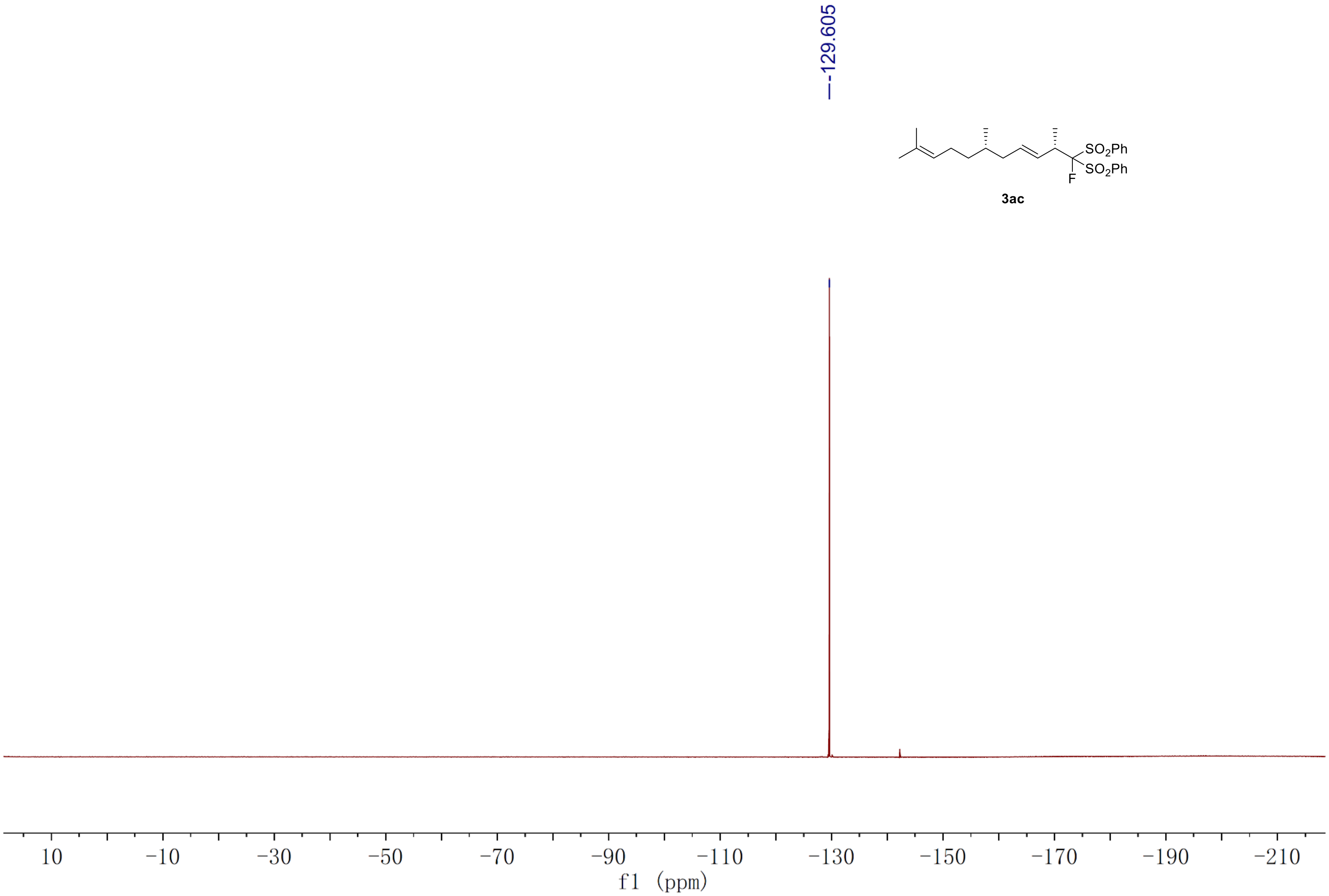

ZHY-ZC-23-400M-H

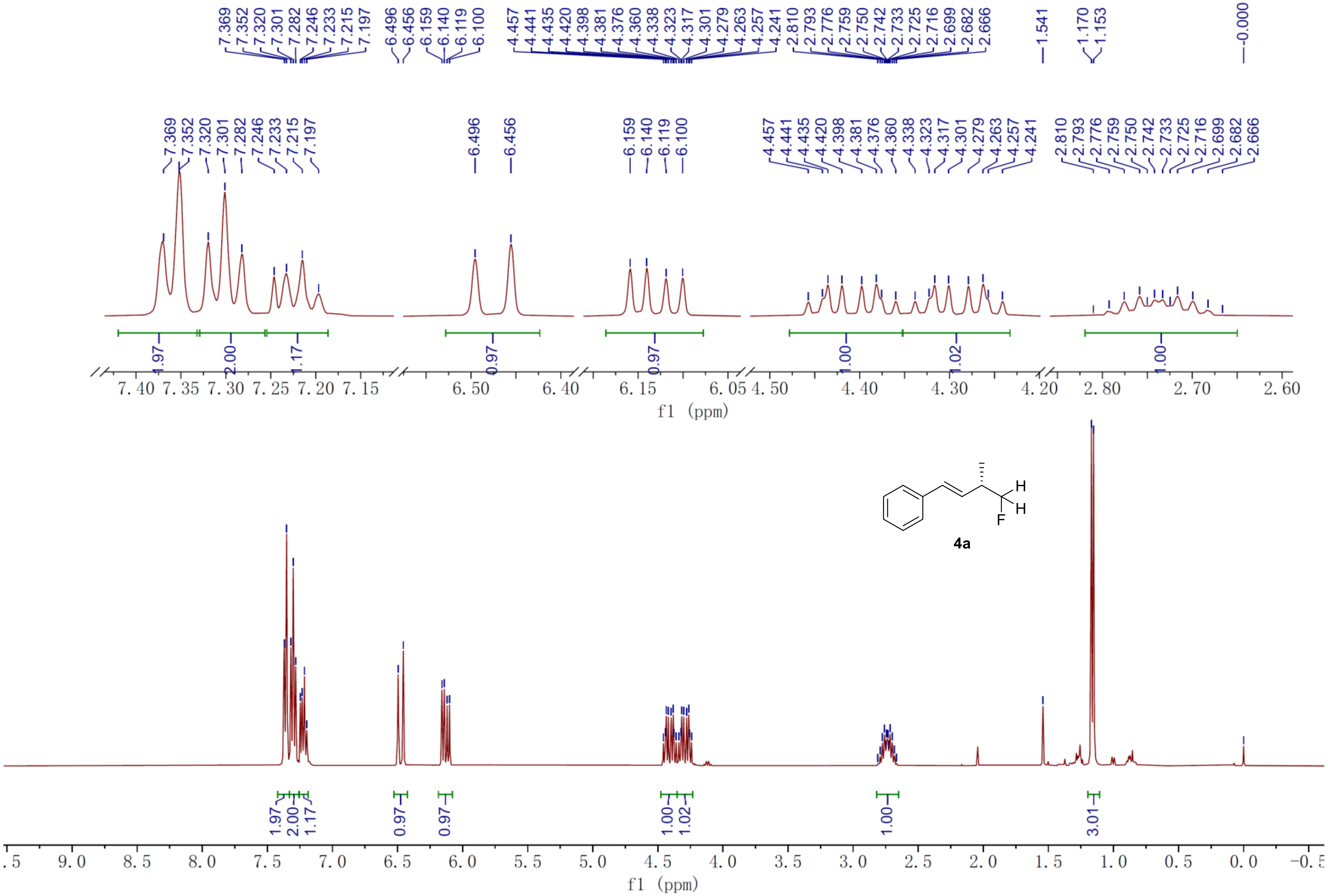

ZHY-ZC-23-100M-C

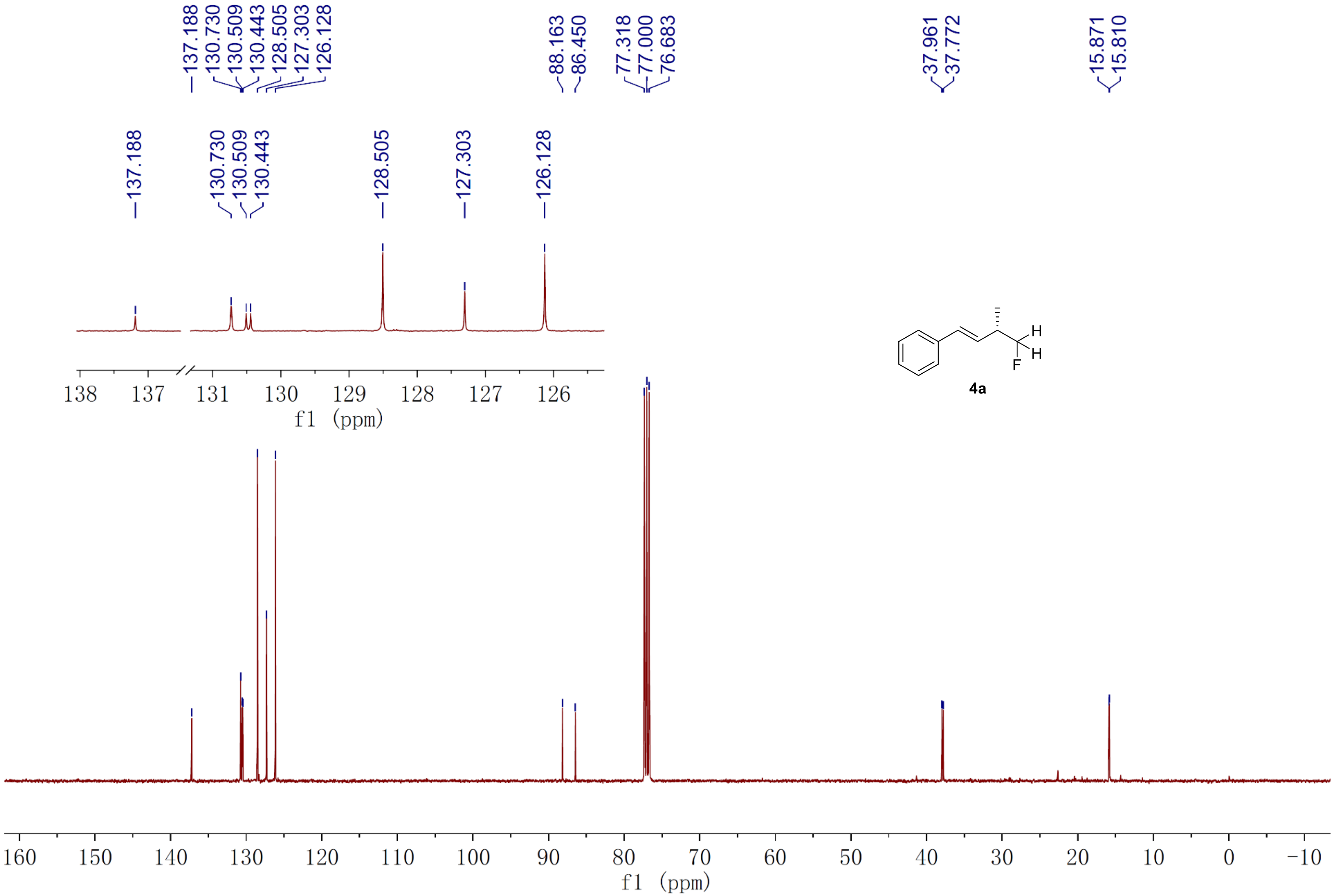

4a

ZHY-ZC-23-376M-F

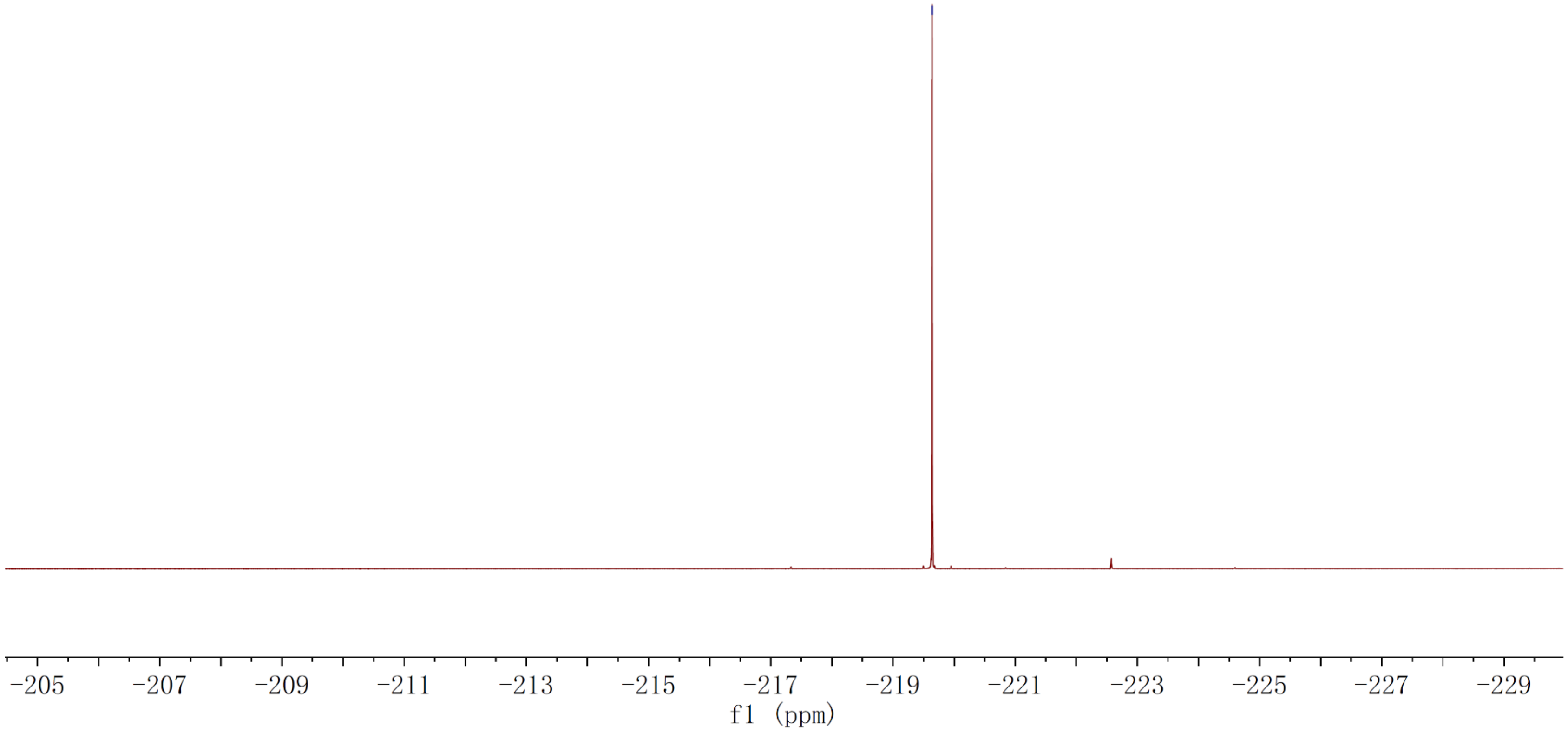

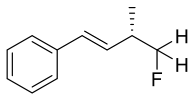

4a

ZHY-ZD-41-400M-H

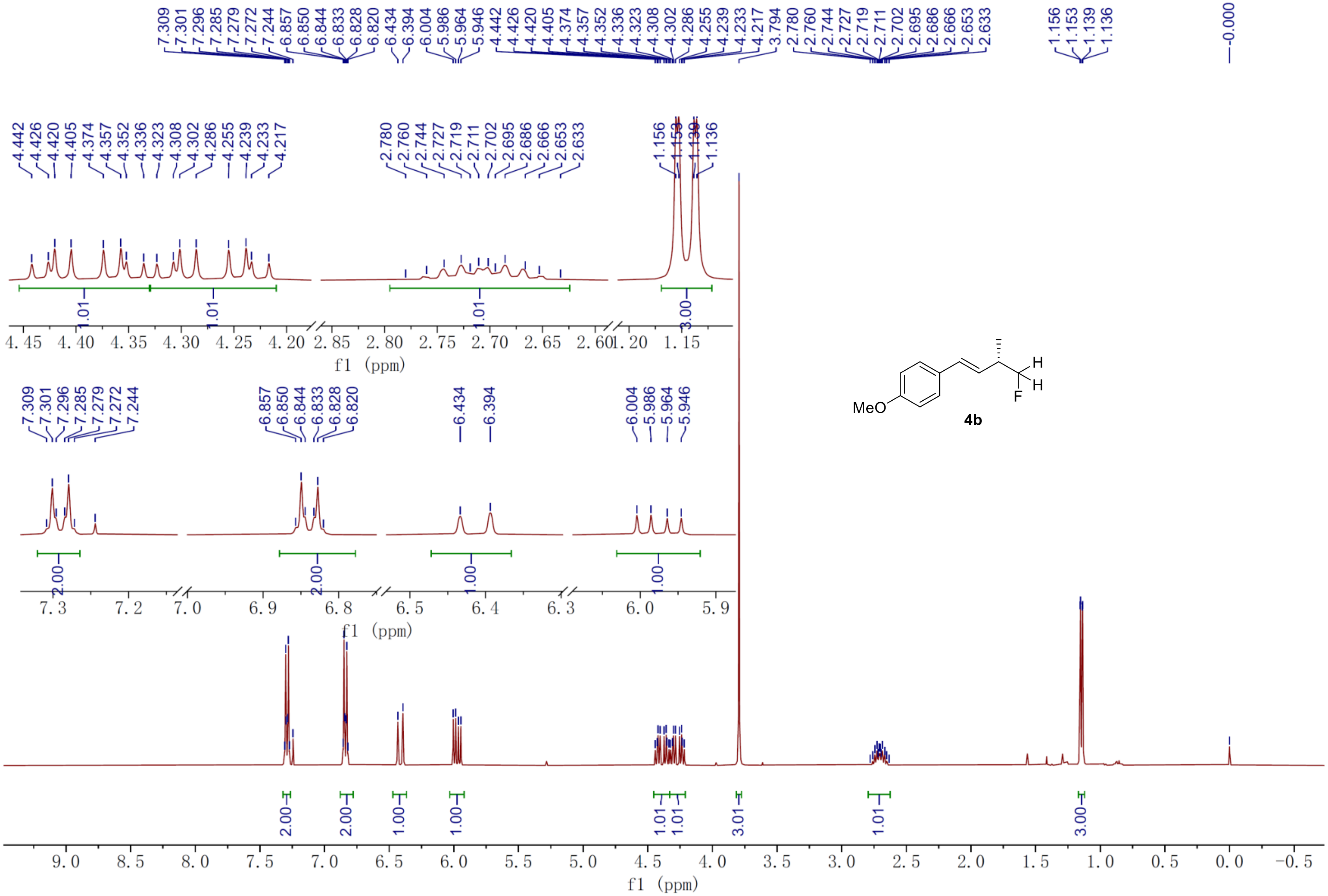

4b

ZHY-ZD-41-3.20.fid — 400M C

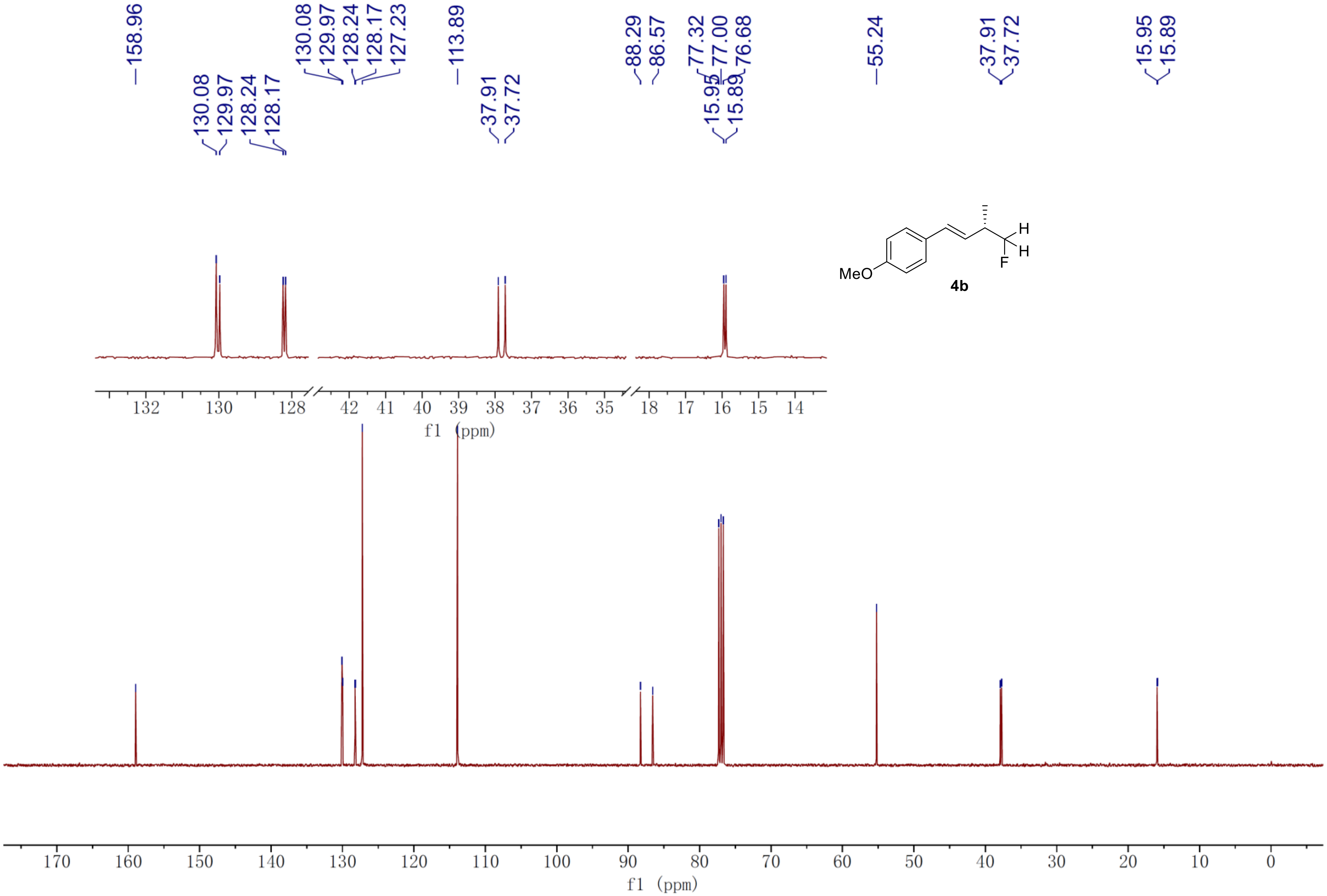

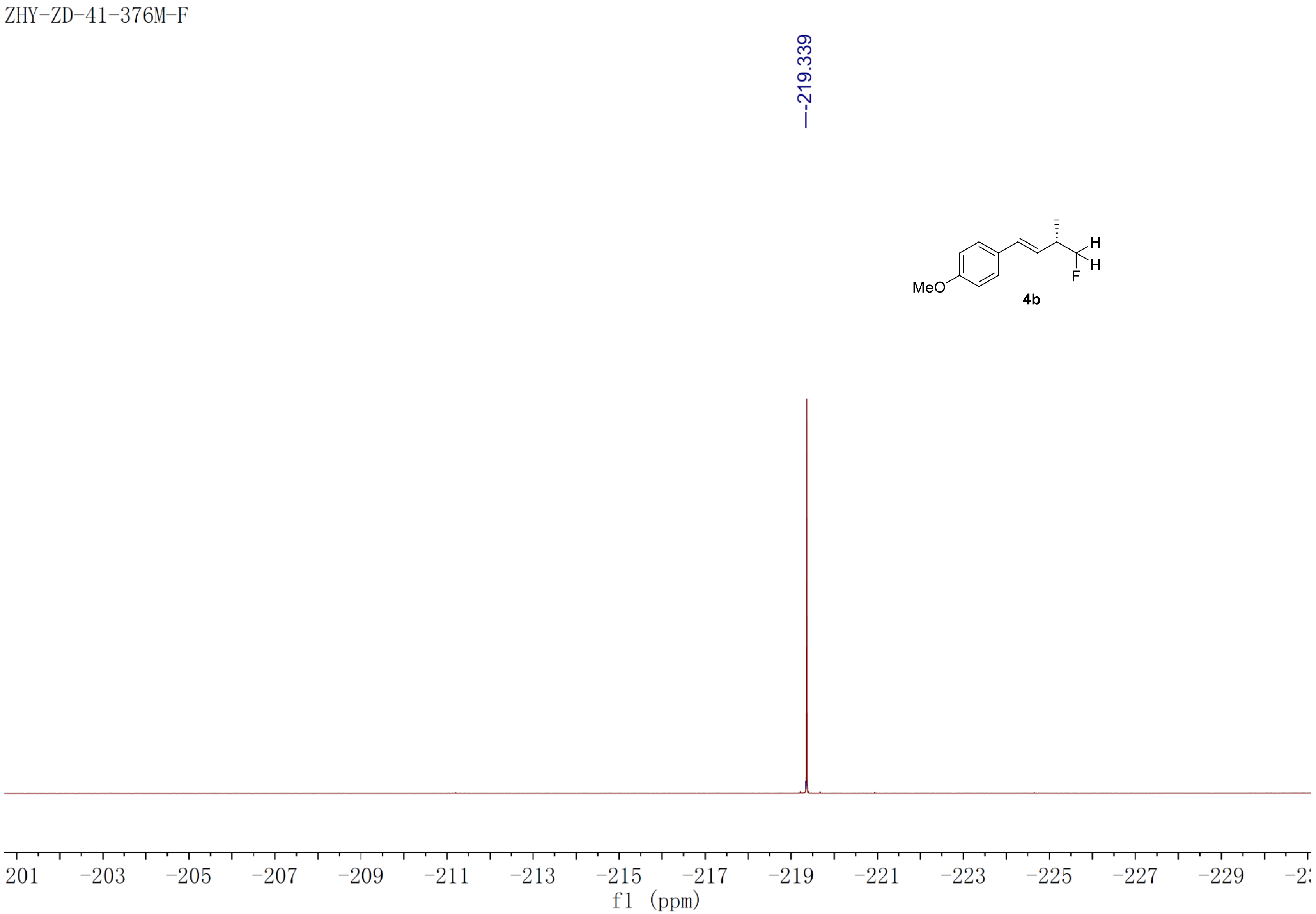

ZHY-ZD-41-376M-F
-219.339
MeO
H
H
F
4b
201
−203
−205
−207
−209
−211
−213
−215
−217
−219
−221
−223
−225
−227
−229
f1 (ppm)

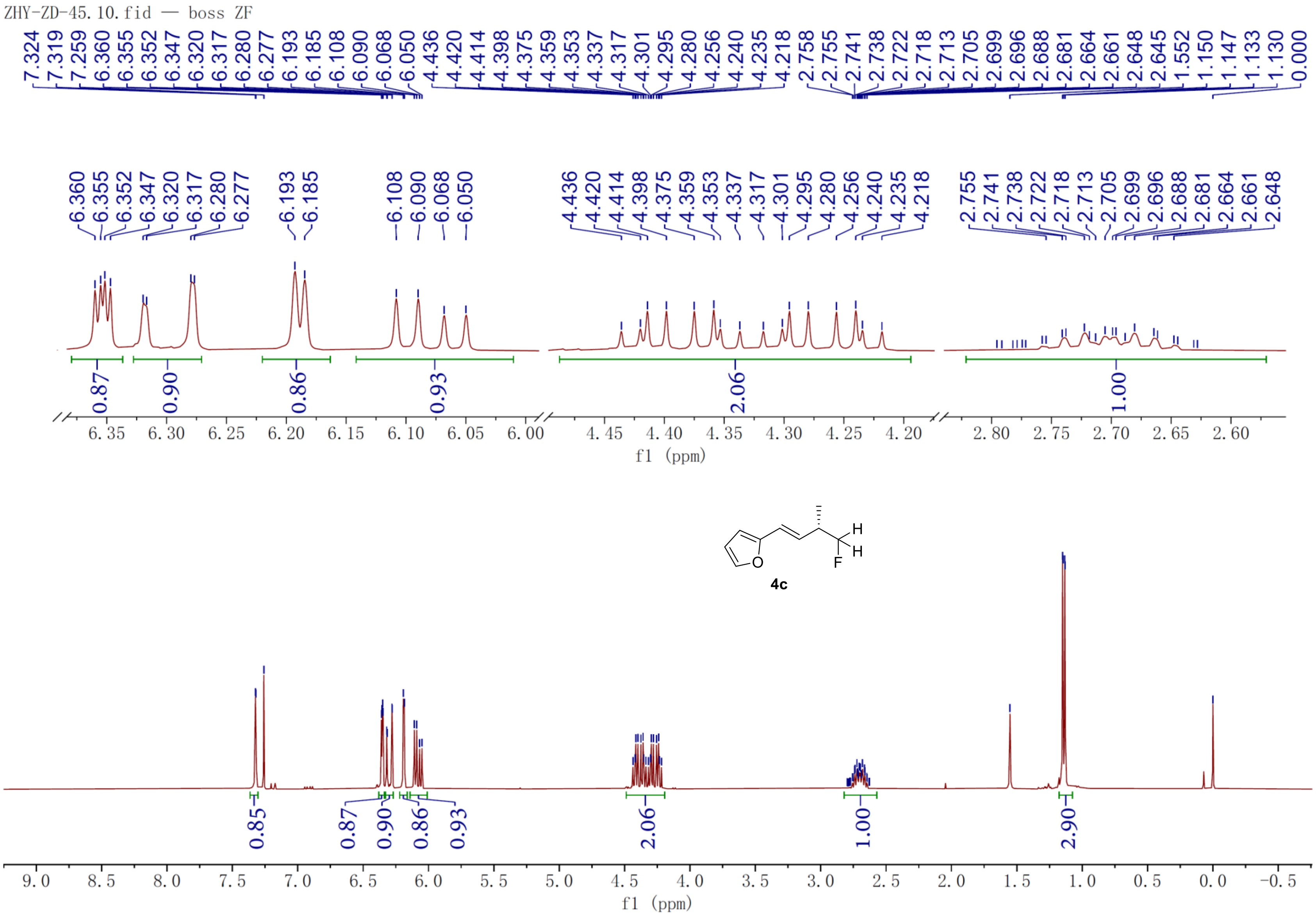
ZHY-ZD-45.10.fid — boss ZF
7.324 7.319 7.259 6.360 6.355 6.352 6.347 6.320 6.317 6.280 6.277 6.193 6.185 6.108 6.090 6.068 6.050 4.436 4.420 4.414 4.398 4.375 4.359 4.353 4.337 4.317 4.301 4.295 4.280 4.256 4.240 4.235 4.218 2.758 2.755 2.741 2.738 2.722 2.718 2.713 2.705 2.699 2.696 2.688 2.681 2.664 2.661 2.648 2.645 1.552 1.150 1.147 1.133 1.130 0.000
4c
f1 (ppm)

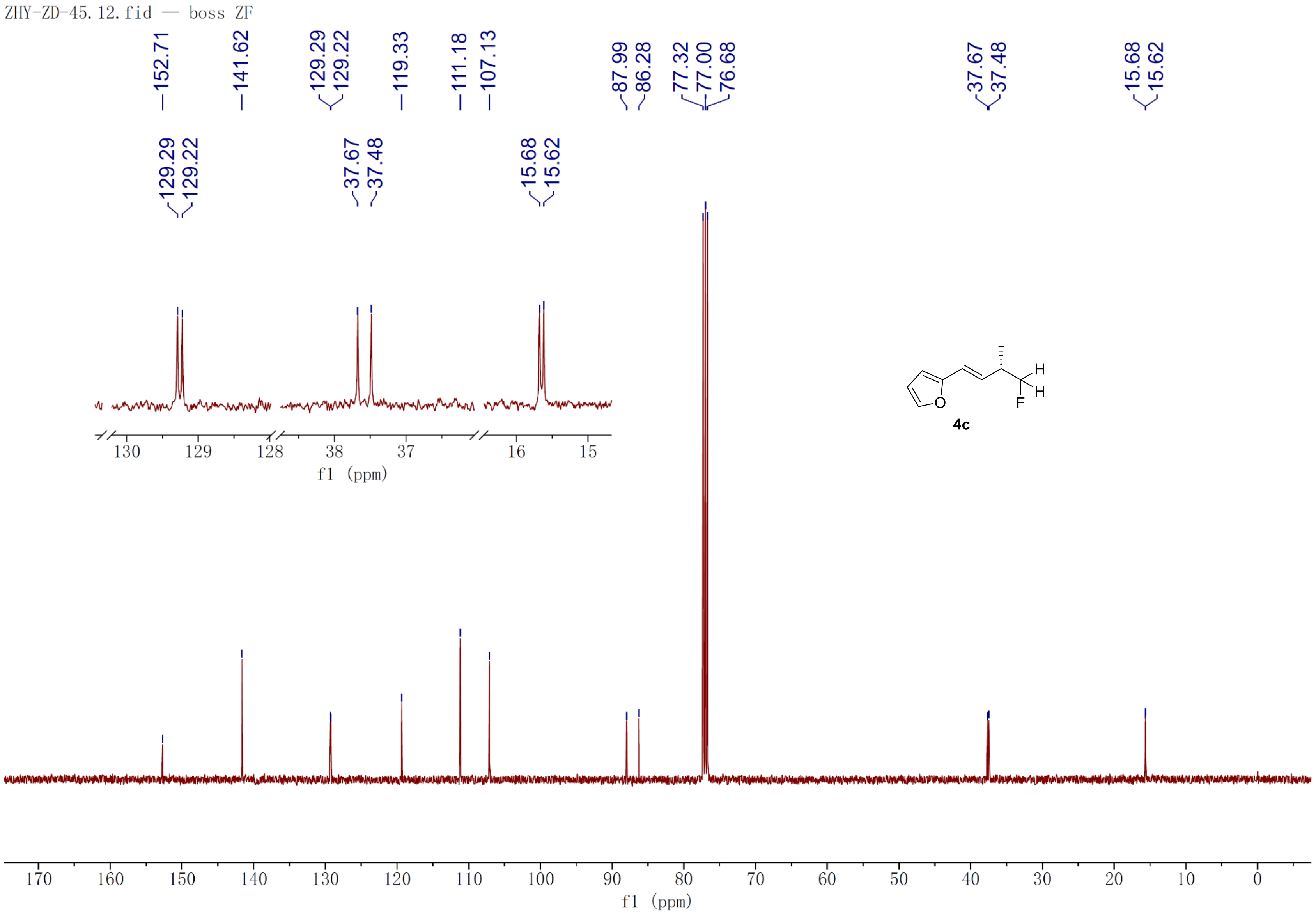
ZHY-ZD-45.12.fid — boss ZF
152.71
141.62
129.29
129.22
119.33
111.18
107.13
87.99
86.28
77.32
77.00
76.68
37.67
37.48
15.68
15.62
129.29
129.22
37.67
37.48
15.68
15.62
130
129
128
38
37
16
15
f1 (ppm)
O
H
H
F
4c
170
160
150
140
130
120
110
100
90
80
70
60
50
40
30
20
10
0
f1 (ppm)

ZHY-ZD-45.11.fid — 400M F

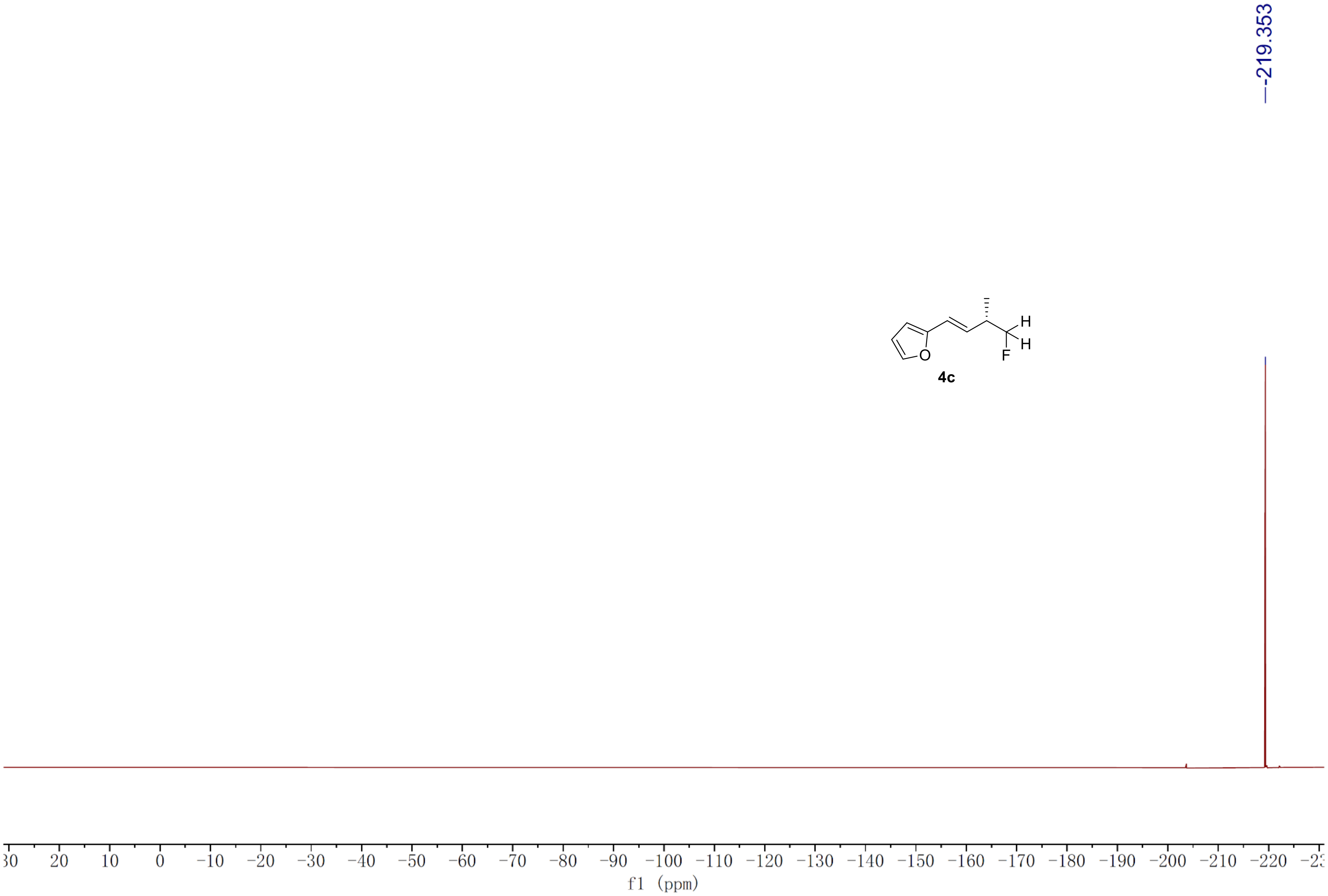

4c

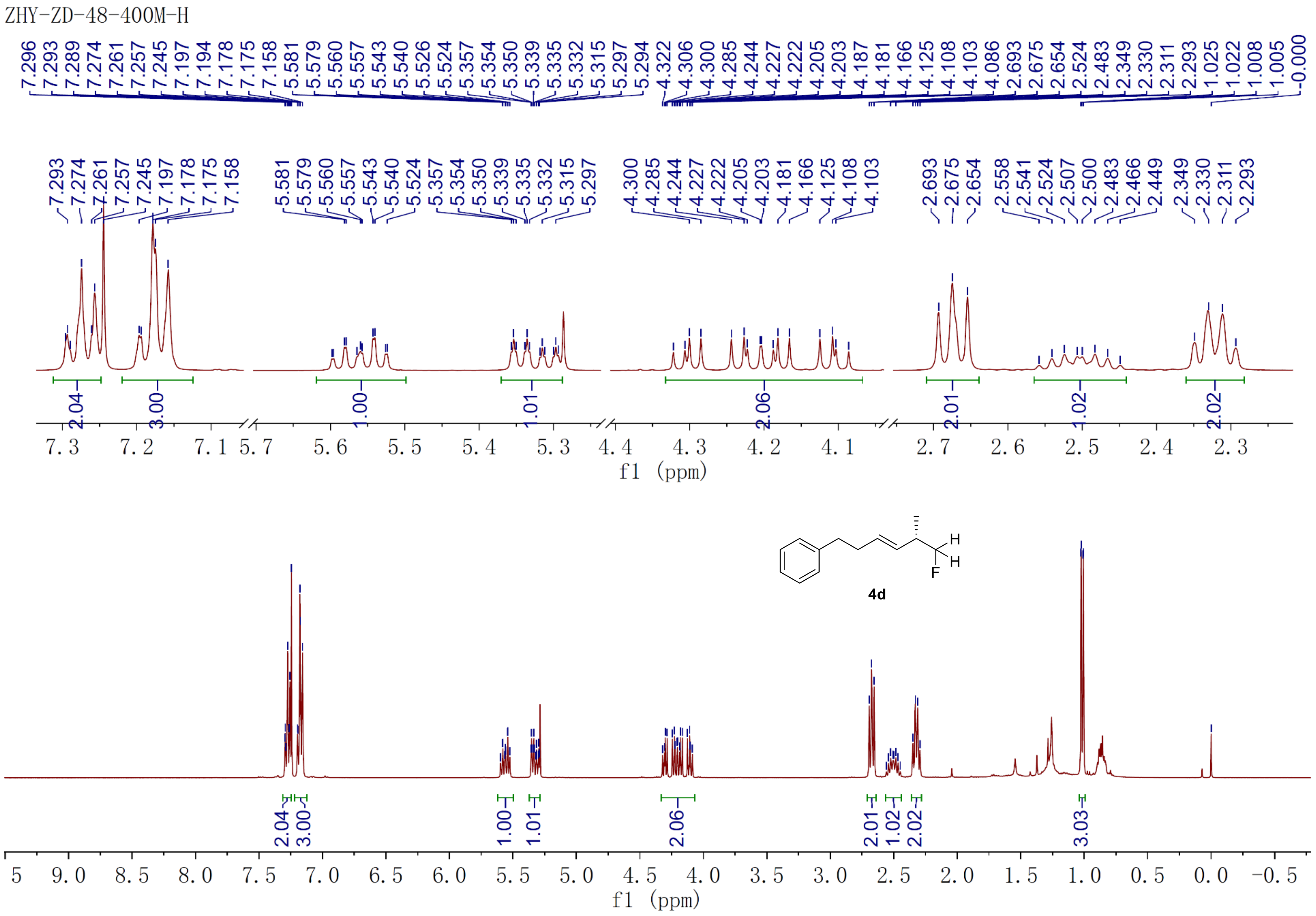
ZHY-ZD-48-400M-H
4d
F
H
H
f1 (ppm)
f1 (ppm)

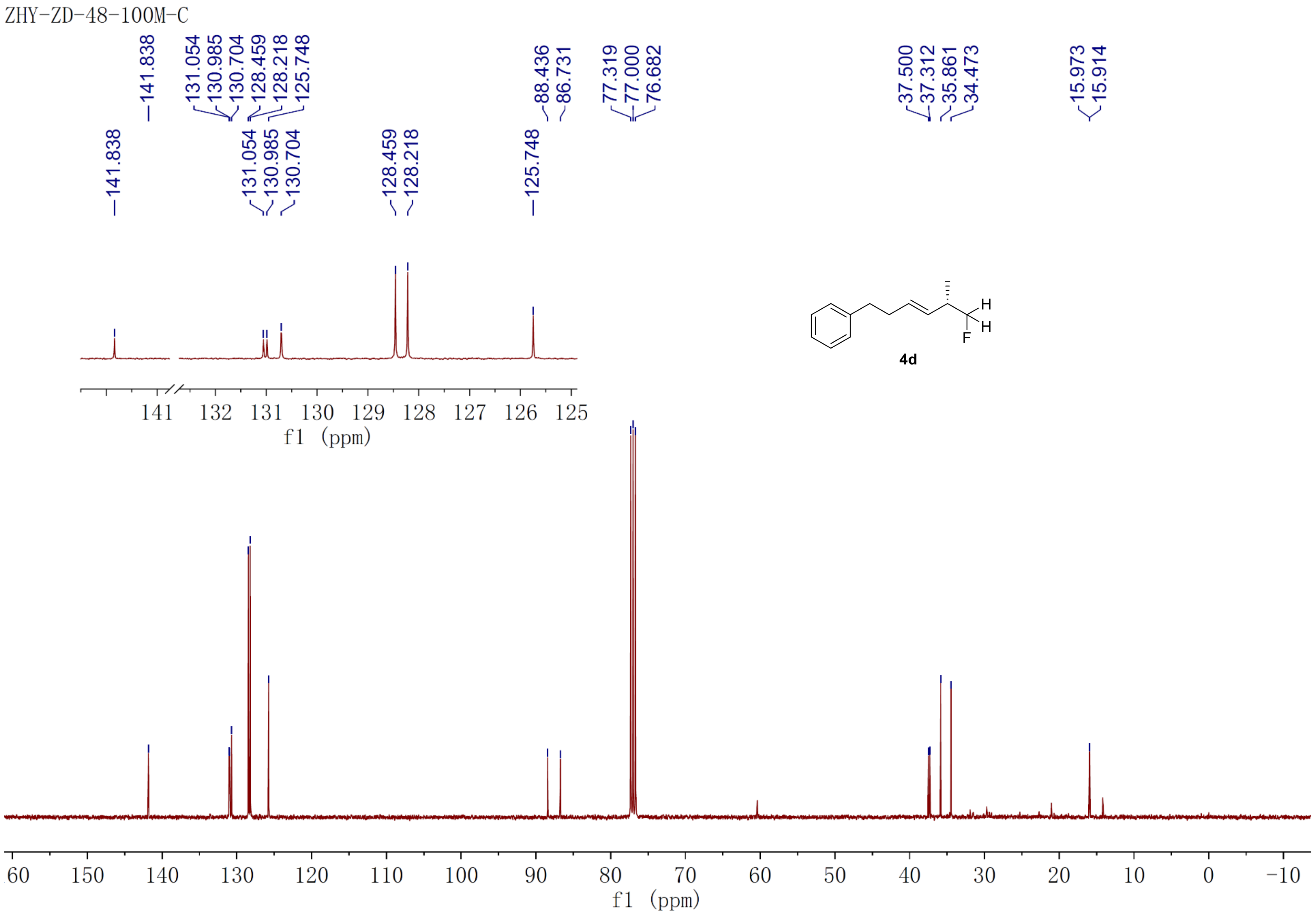
ZHY-ZD-48-100M-C
141.838
131.054
130.985
130.704
128.459
128.218
125.748
88.436
86.731
77.319
77.000
76.682
37.500
37.312
35.861
34.473
15.973
15.914
141.838
131.054
130.985
130.704
128.459
128.218
125.748
H
H
F
4d
f1 (ppm)
f1 (ppm)

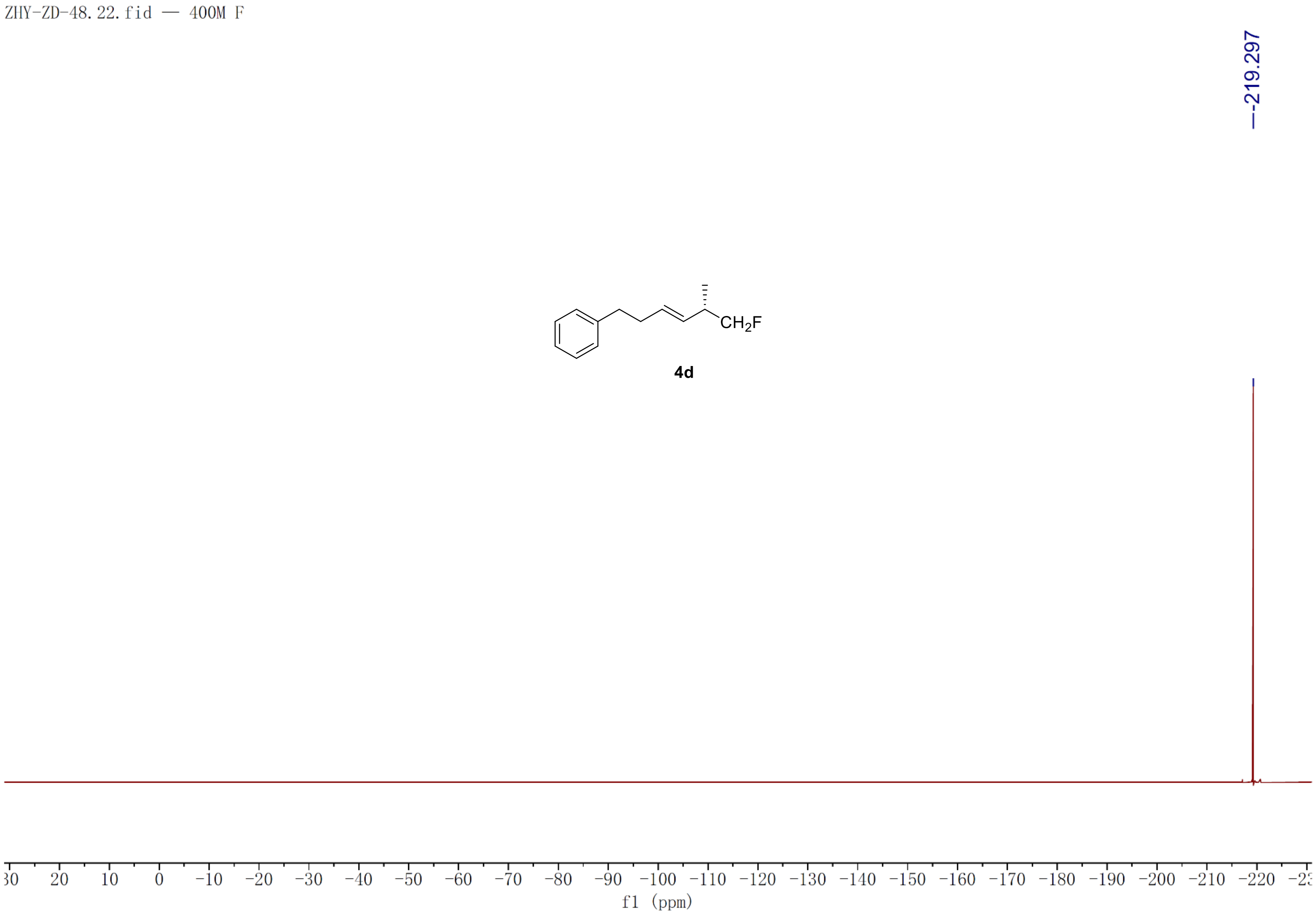
ZHY-ZD-48.22.fid — 400M F
-219.297
CH2F
4d
30 20 10 0 -10 -20 -30 -40 -50 -60 -70 -80 -90 -100 -110 -120 -130 -140 -150 -160 -170 -180 -190 -200 -210 -220
f1 (ppm)

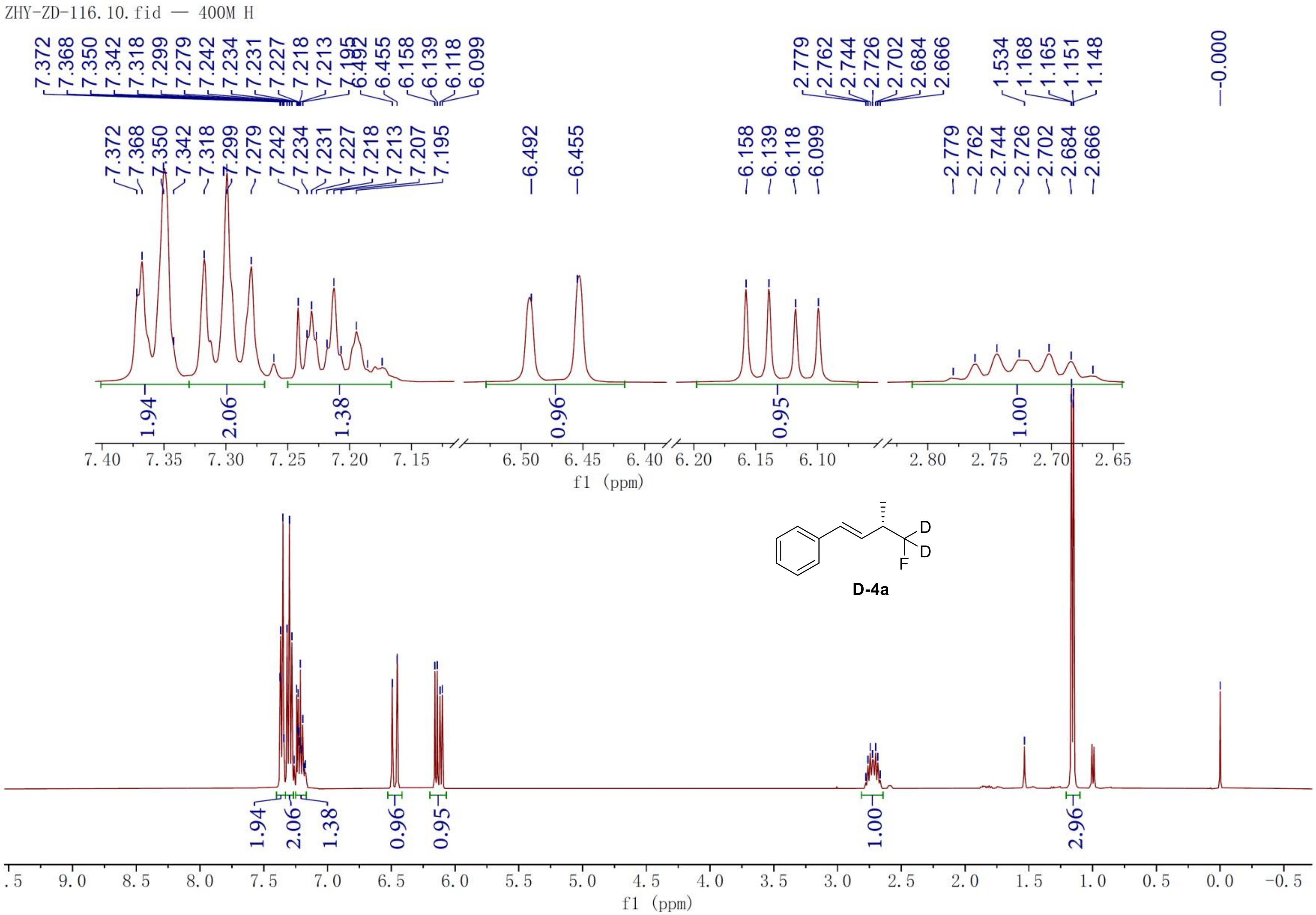
ZHY-ZD-116.10.fid — 400M H
7.372 7.368 7.350 7.342 7.318 7.299 7.279 7.242 7.234 7.231 7.227 7.218 7.213 7.207 7.195
6.492 6.455
6.158 6.139 6.118 6.099
2.779 2.762 2.744 2.726 2.702 2.684 2.666
1.534 1.168 1.165 1.151 1.148
0.000
1.94 2.06 1.38 0.96 0.95 1.00 2.96
f1 (ppm)
D
D
F
D-4a

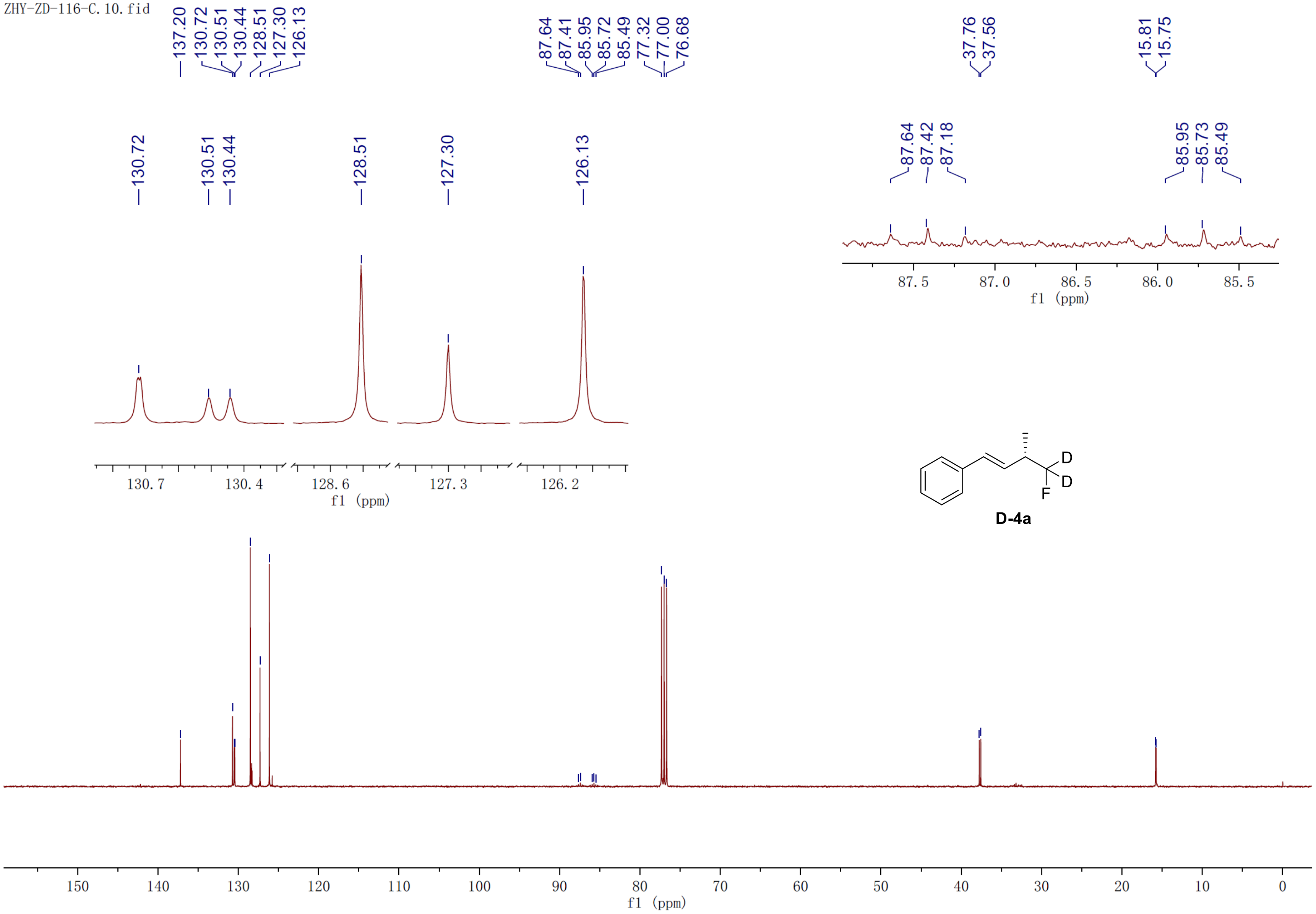
ZHY-ZD-116-C.10.fid
137.20
130.72
130.51
130.44
128.51
127.30
126.13
87.64
87.41
85.95
85.72
85.49
77.32
77.00
76.68
37.76
37.56
15.81
15.75
87.64
87.42
87.18
85.95
85.73
85.49
f1 (ppm)
D
D
F
D-4a

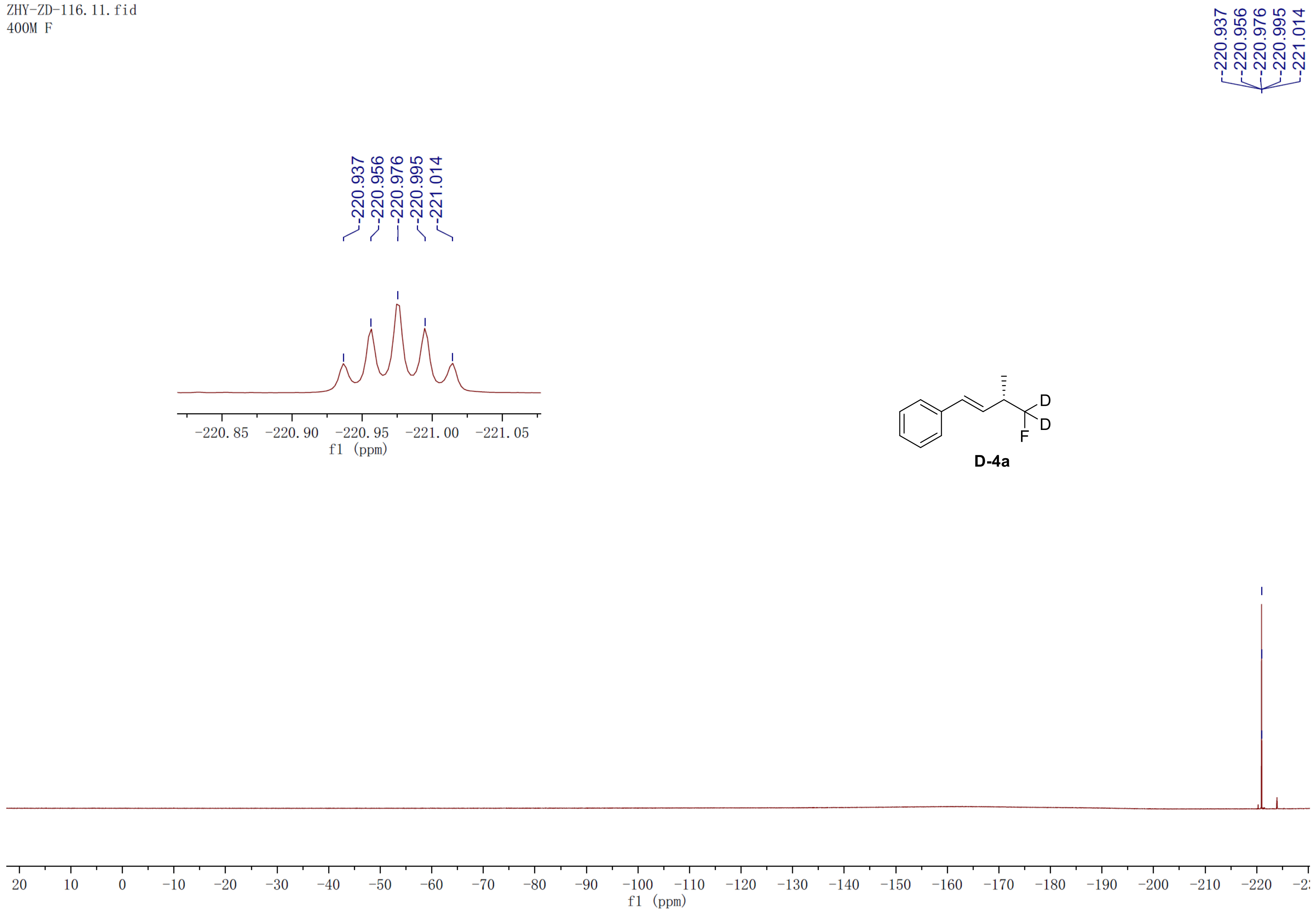
ZHY-ZD-116.11.fid
400M F
-220.937
-220.956
-220.976
-220.995
-221.014
-220.85 -220.90 -220.95 -221.00 -221.05
f1 (ppm)
D
D
F
D-4a
20 10 0 -10 -20 -30 -40 -50 -60 -70 -80 -90 -100 -110 -120 -130 -140 -150 -160 -170 -180 -190 -200 -210 -220
f1 (ppm)

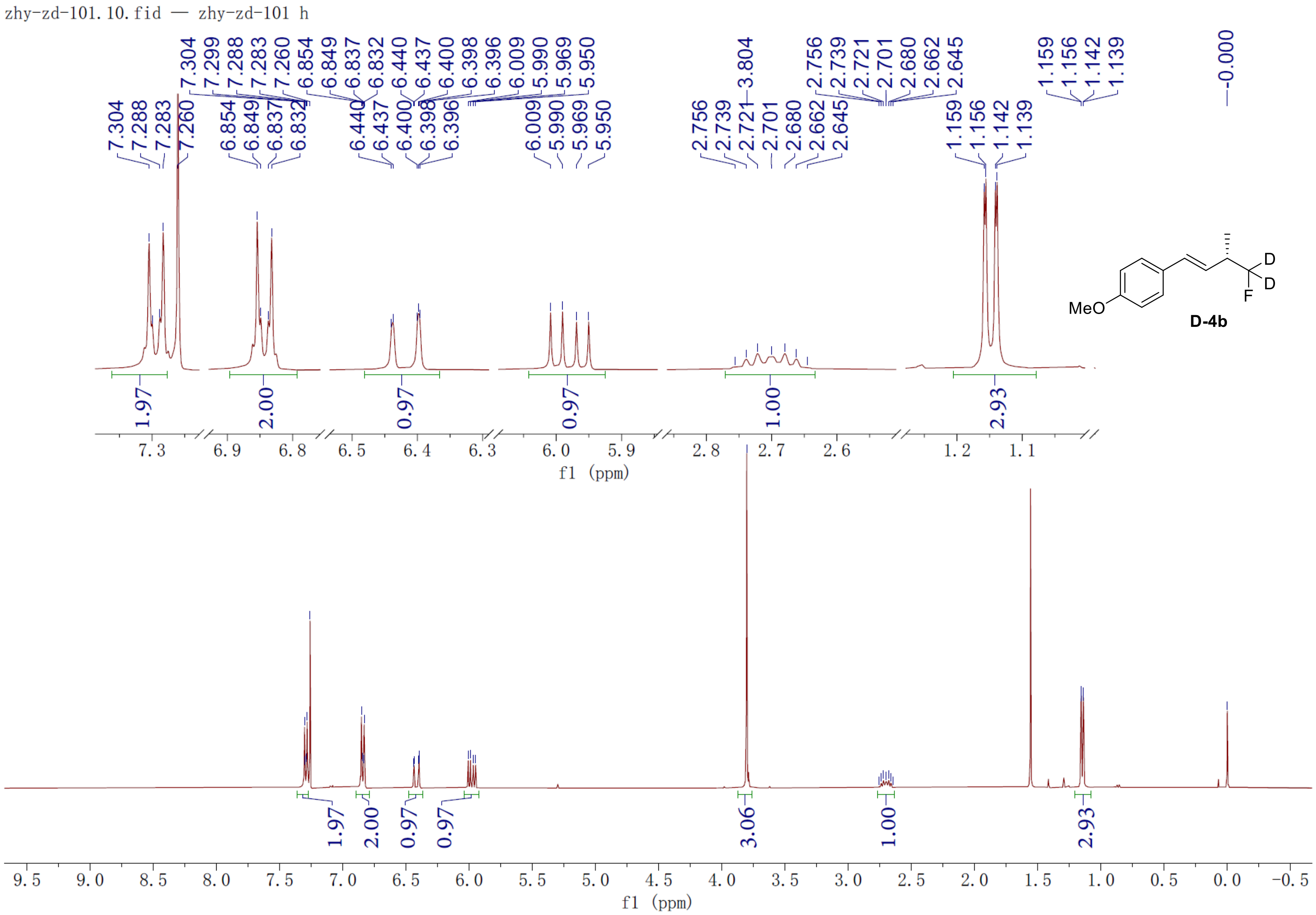
zhy-zd-101.10.fid — zhy-zd-101 h
MeO
D
D
F
D-4b
f1 (ppm)

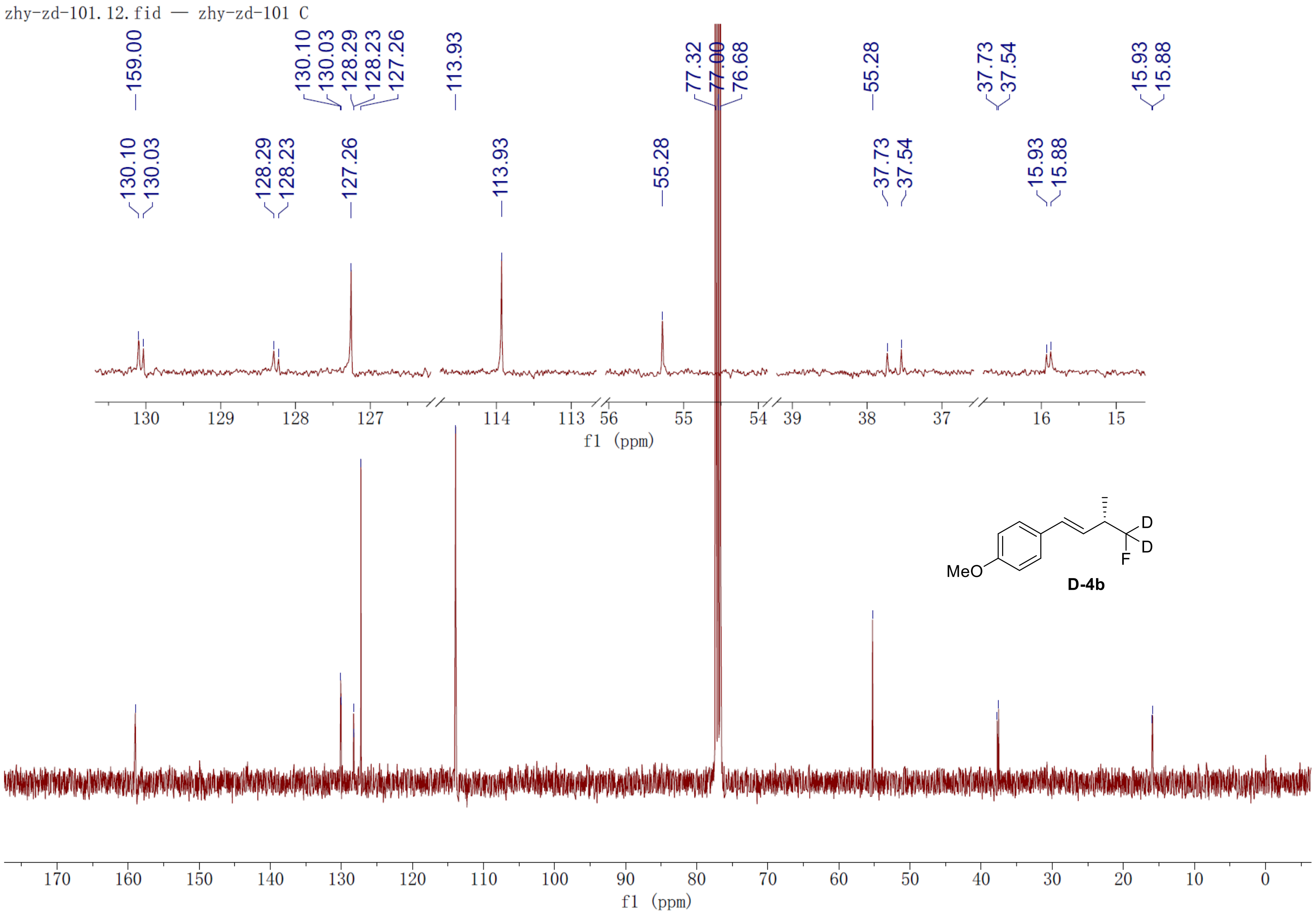
zhy-zd-101.12.fid — zhy-zd-101 C
159.00
130.10
130.03
128.29
128.23
127.26
113.93
77.32
77.00
76.68
55.28
37.73
37.54
15.93
15.88
f1 (ppm)
MeO
D
D
F
D-4b

zhy-zd-101.14.fid
zhy-zd-101 f

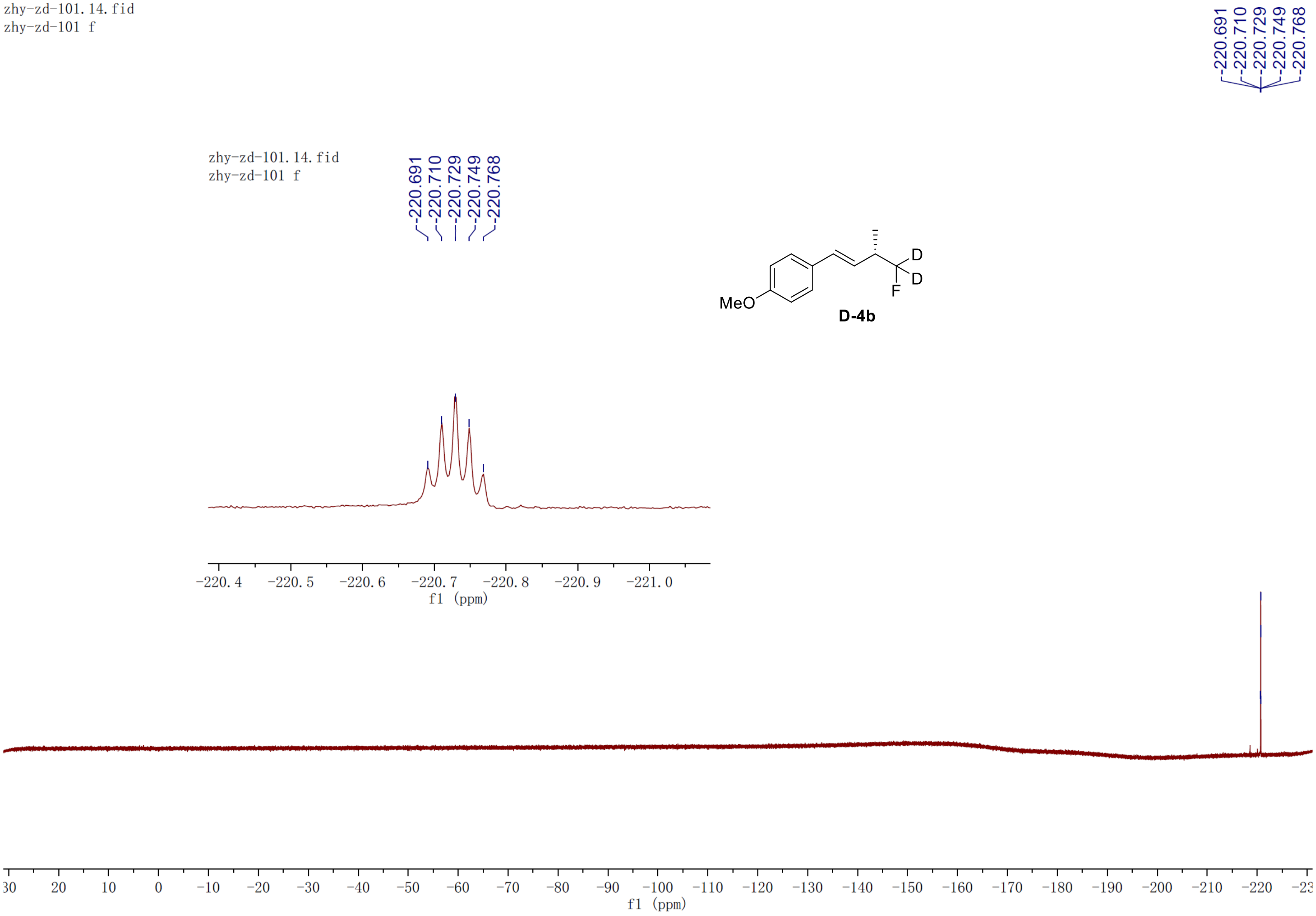

D-4b

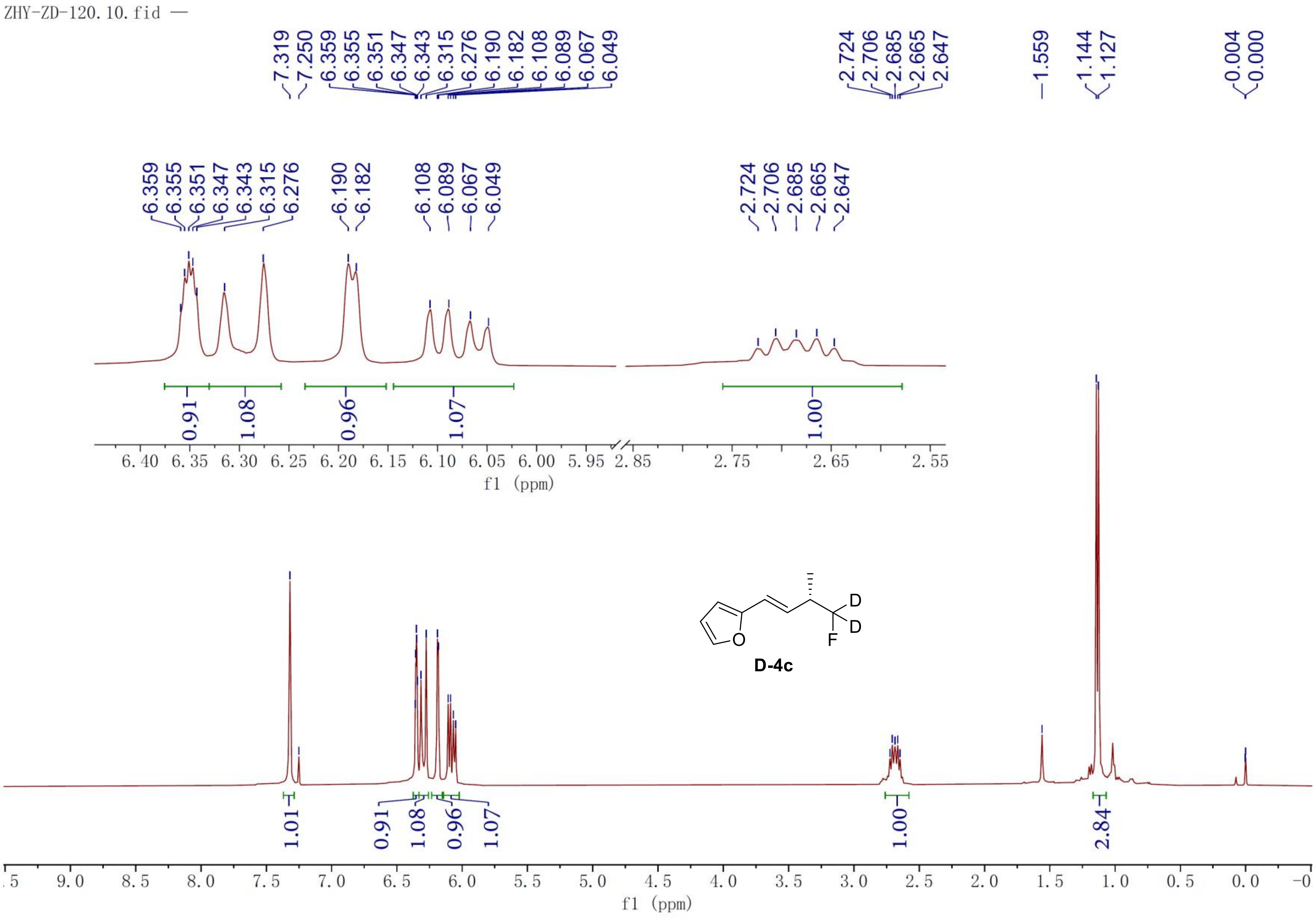
ZHY-ZD-120.10.fid
D-4c
f1 (ppm)

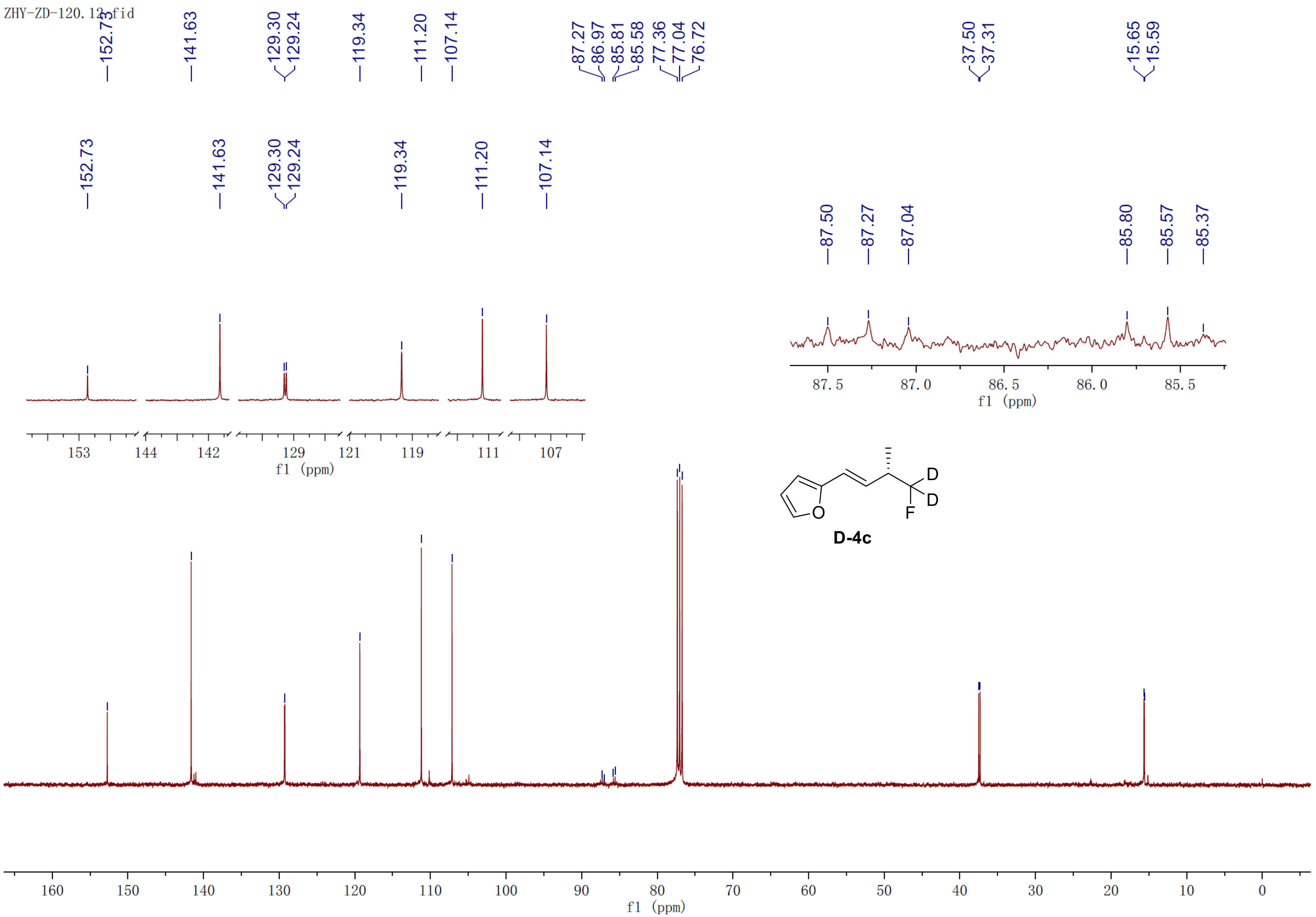
ZHY-ZD-120.12.fid
152.73
141.63
129.30
129.24
119.34
111.20
107.14
87.27
86.97
85.81
85.58
77.36
77.04
76.72
37.50
37.31
15.65
15.59
87.50
87.27
87.04
85.80
85.57
85.37
f1 (ppm)
D
D
F
O
D-4c

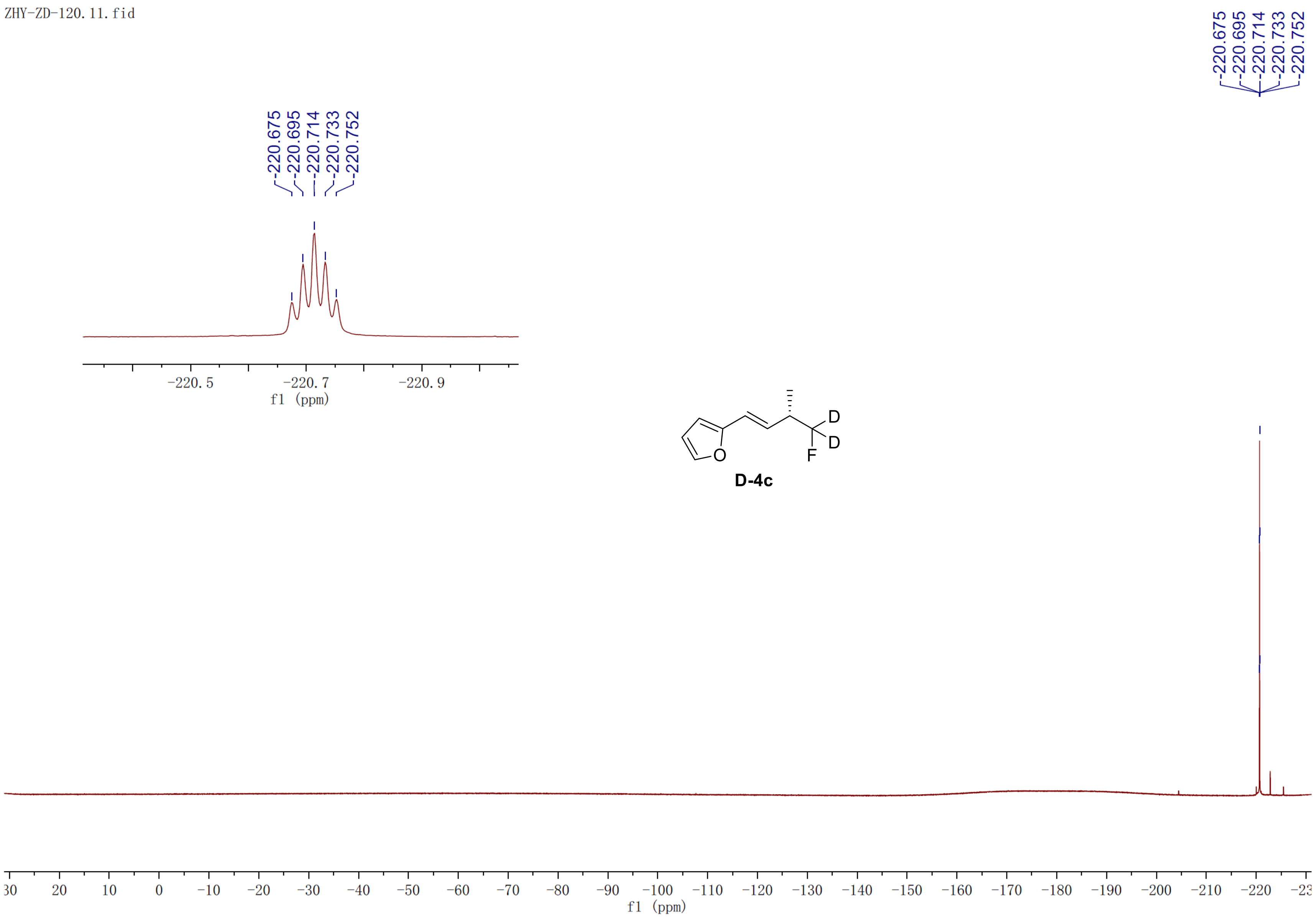
ZHY-ZD-120.11.fid
-220.675
-220.695
-220.714
-220.733
-220.752
-220.5
-220.7
-220.9
f1 (ppm)
D
D
F
O
D-4c
30 20 10 0 -10 -20 -30 -40 -50 -60 -70 -80 -90 -100 -110 -120 -130 -140 -150 -160 -170 -180 -190 -200 -210 -220
f1 (ppm)

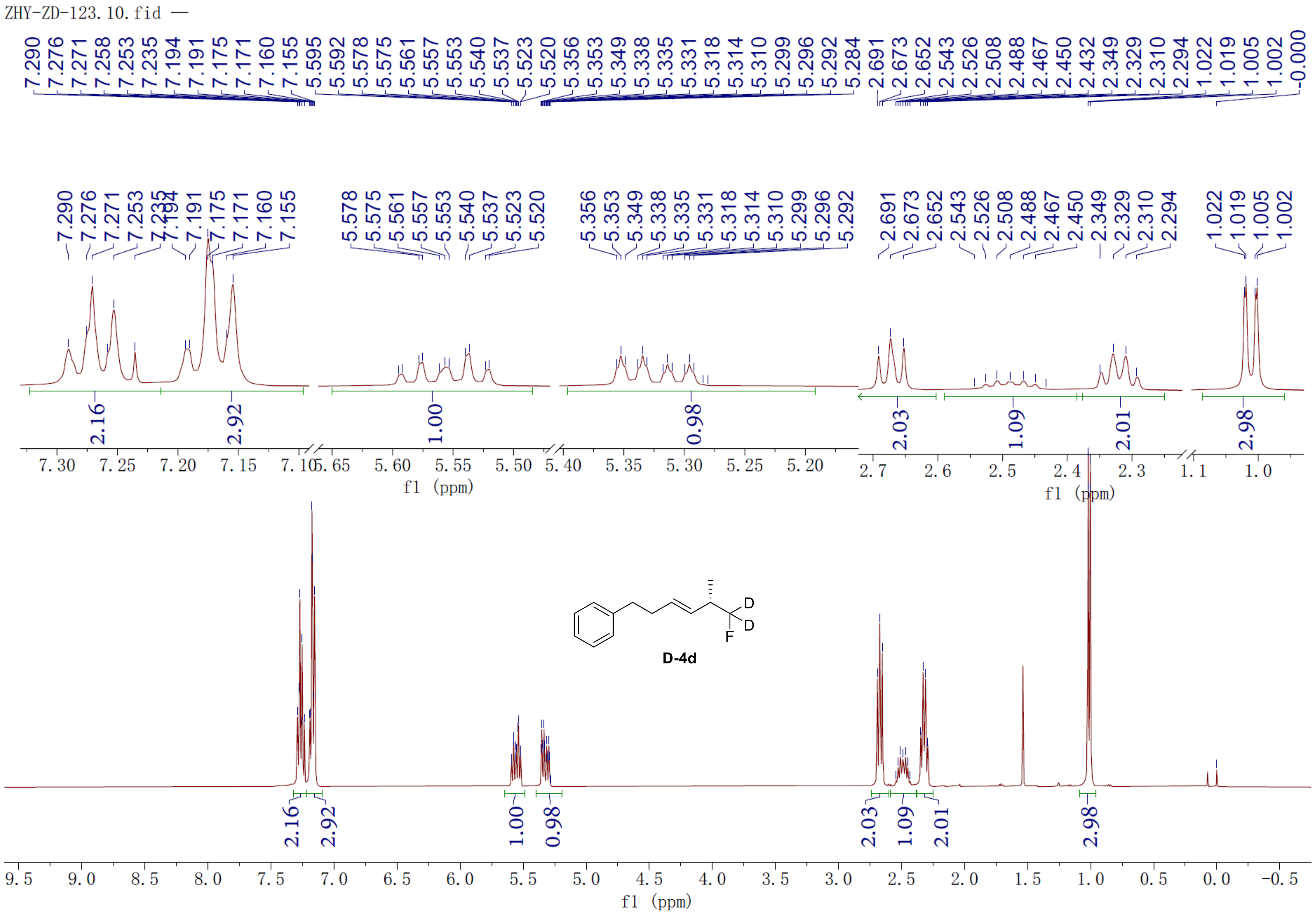
ZHY-ZD-123.10.fid —
7.290 7.276 7.271 7.258 7.253 7.235 7.194 7.191 7.175 7.171 7.160 7.155 5.595 5.592 5.578 5.575 5.561 5.557 5.553 5.540 5.537 5.523 5.520 5.356 5.353 5.349 5.338 5.335 5.331 5.318 5.314 5.310 5.299 5.296 5.292 5.284 2.691 2.673 2.652 2.543 2.526 2.508 2.488 2.467 2.450 2.432 2.349 2.329 2.310 2.294 1.022 1.019 1.005 1.002 -0.000
2.16
2.92
1.00
0.98
2.03
1.09
2.01
2.98
f1 (ppm)
D
D
F
D-4d

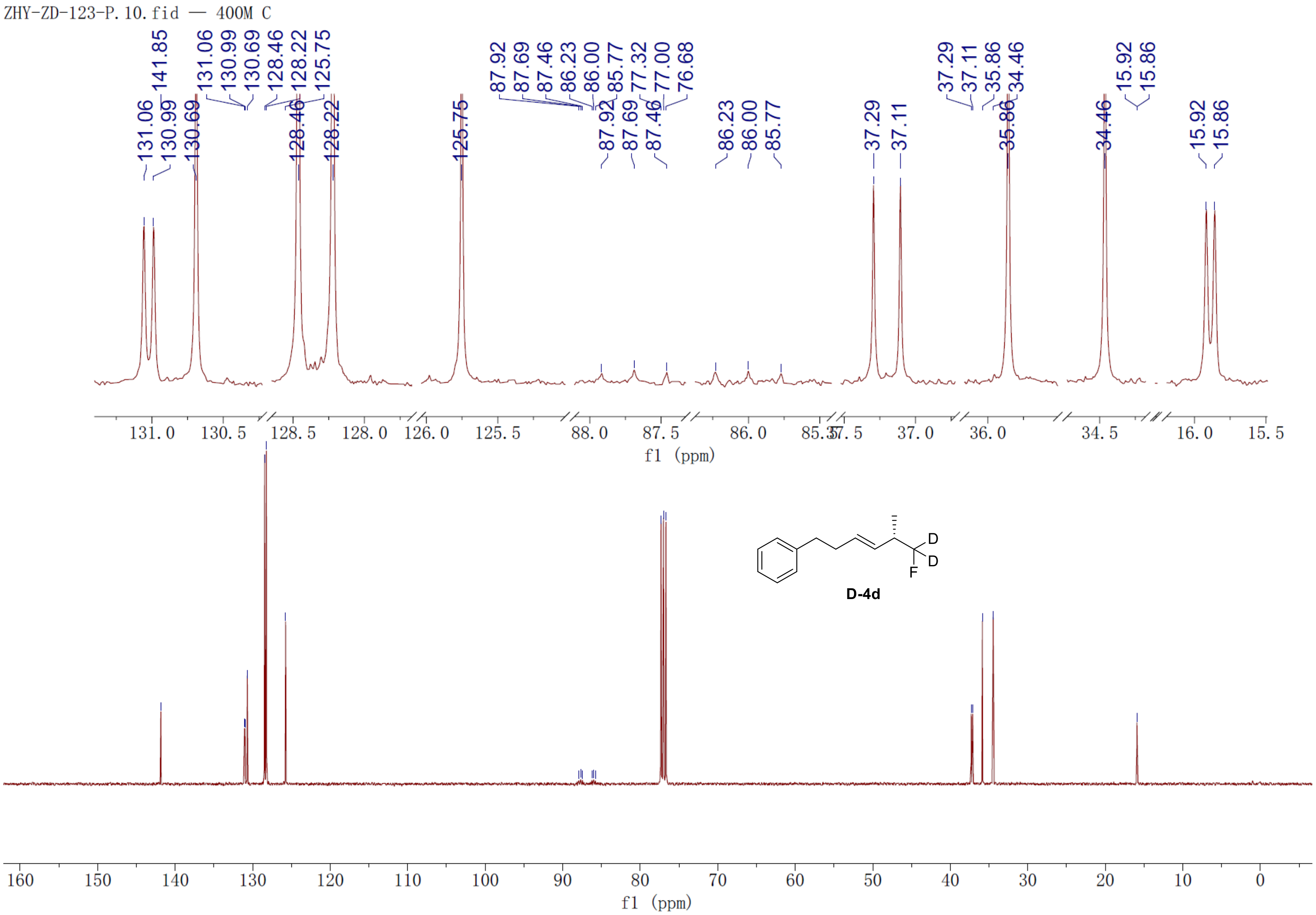
ZHY-ZD-123-P.10.fid — 400M C
141.85
131.06
130.99
130.69
128.46
128.22
125.75
87.92
87.69
87.46
86.23
86.00
85.77
77.32
77.00
76.68
37.29
37.11
35.86
34.46
15.92
15.86
f1 (ppm)
D
D
F
D-4d

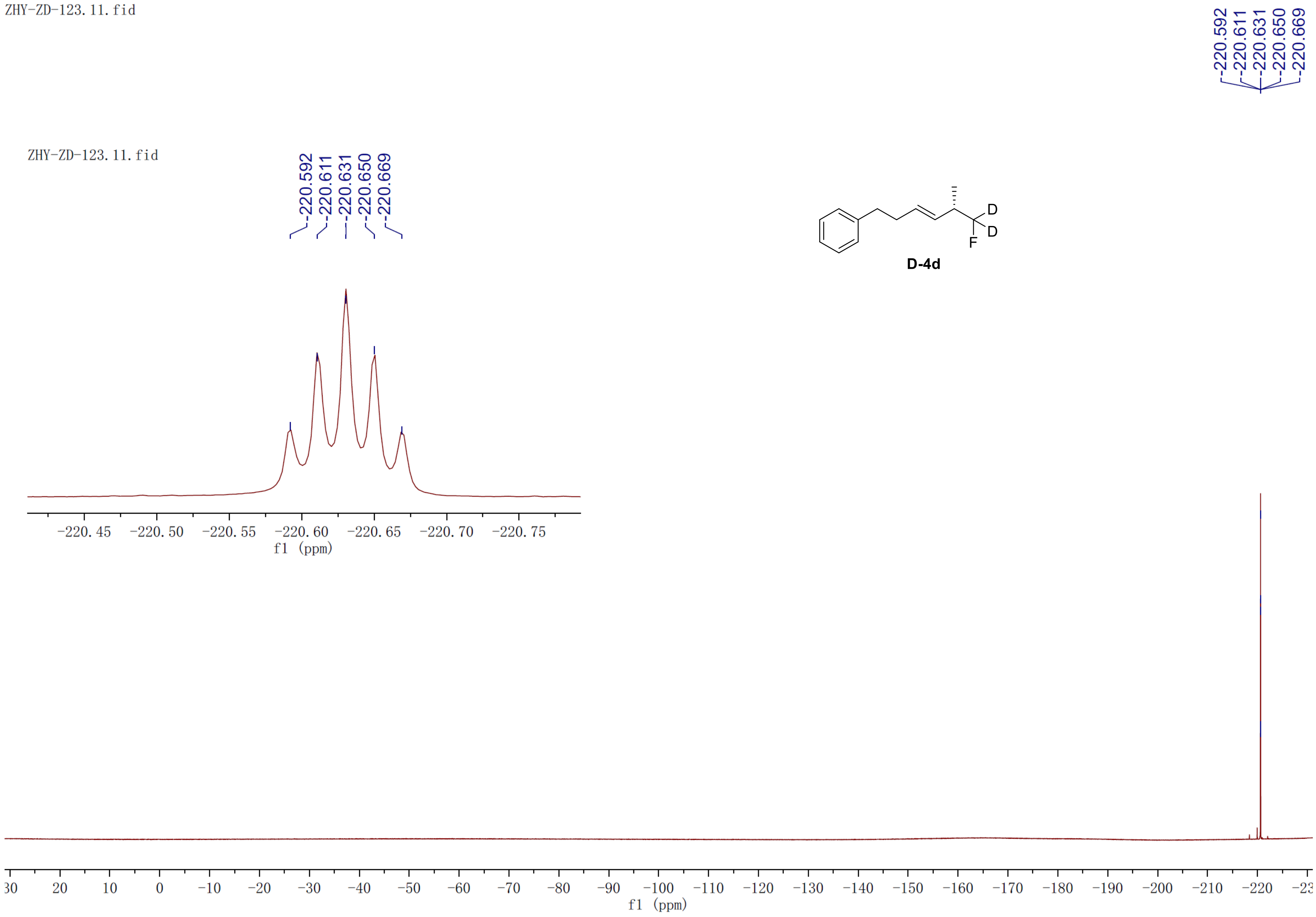
ZHY-ZD-123.11.fid
-220.592
-220.611
-220.631
-220.650
-220.669
ZHY-ZD-123.11.fid
-220.592
-220.611
-220.631
-220.650
-220.669
D
D
F
D-4d
-220.45 -220.50 -220.55 -220.60 -220.65 -220.70 -220.75
f1 (ppm)
20 10 0 -10 -20 -30 -40 -50 -60 -70 -80 -90 -100 -110 -120 -130 -140 -150 -160 -170 -180 -190 -200 -210 -220
f1 (ppm)

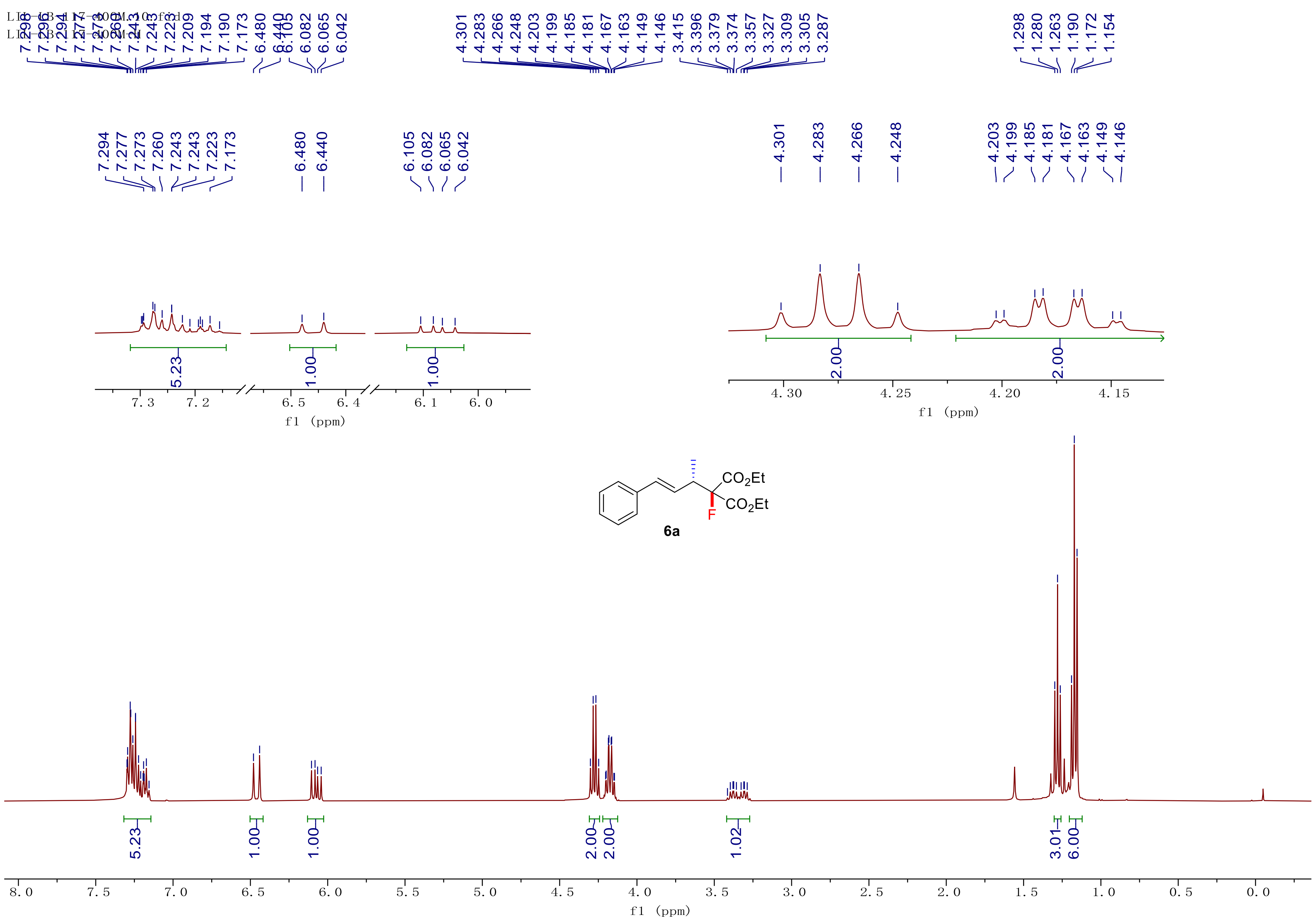
CO2Et
CO2Et
F
6a
f1 (ppm)

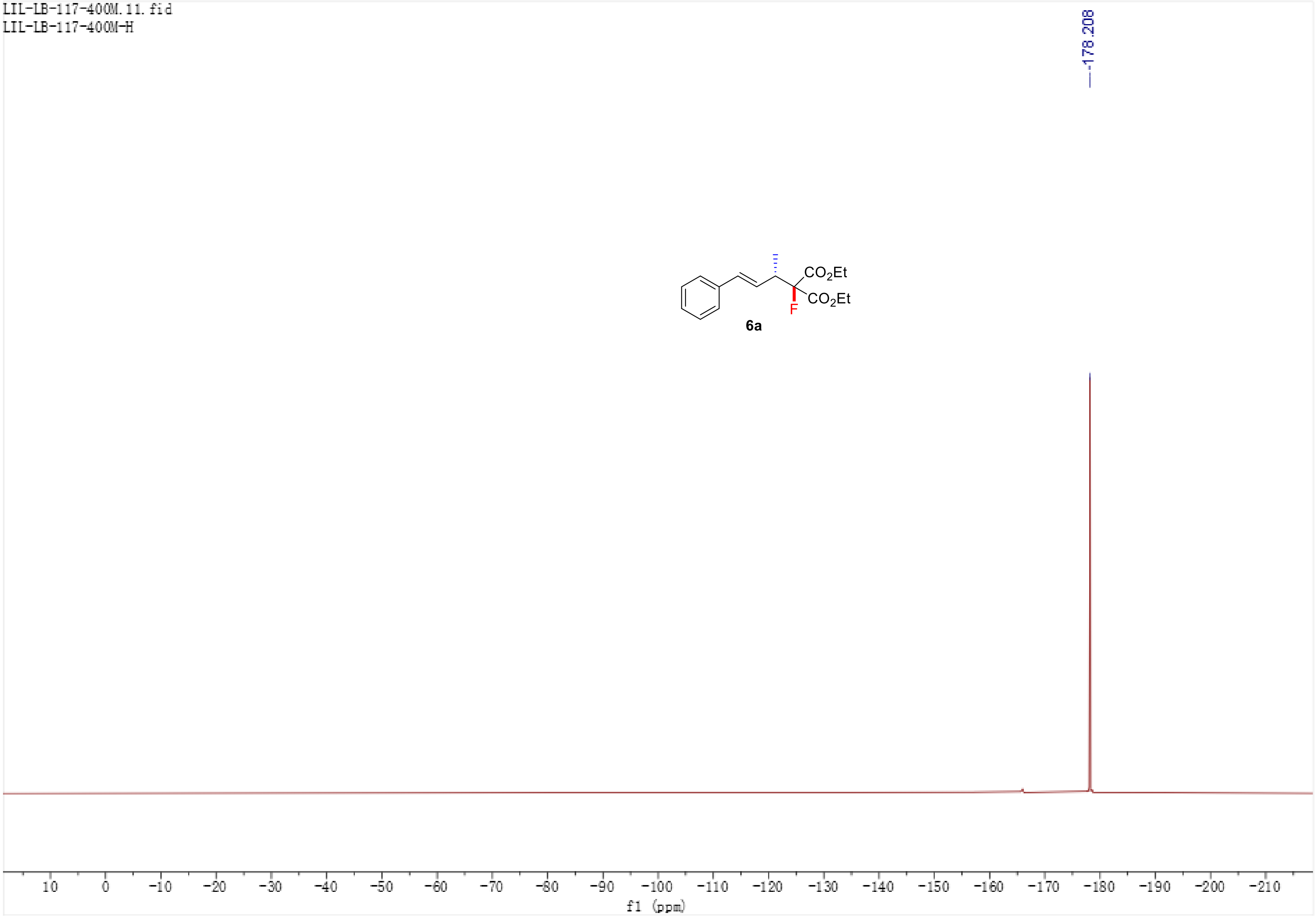
LIL-LB-117-400M.11.fid
LIL-LB-117-400M-H
-178.208
CO2Et
CO2Et
F
6a
10 0 −10 −20 −30 −40 −50 −60 −70 −80 −90 −100 −110 −120 −130 −140 −150 −160 −170 −180 −190 −200 −210
f1 (ppm)

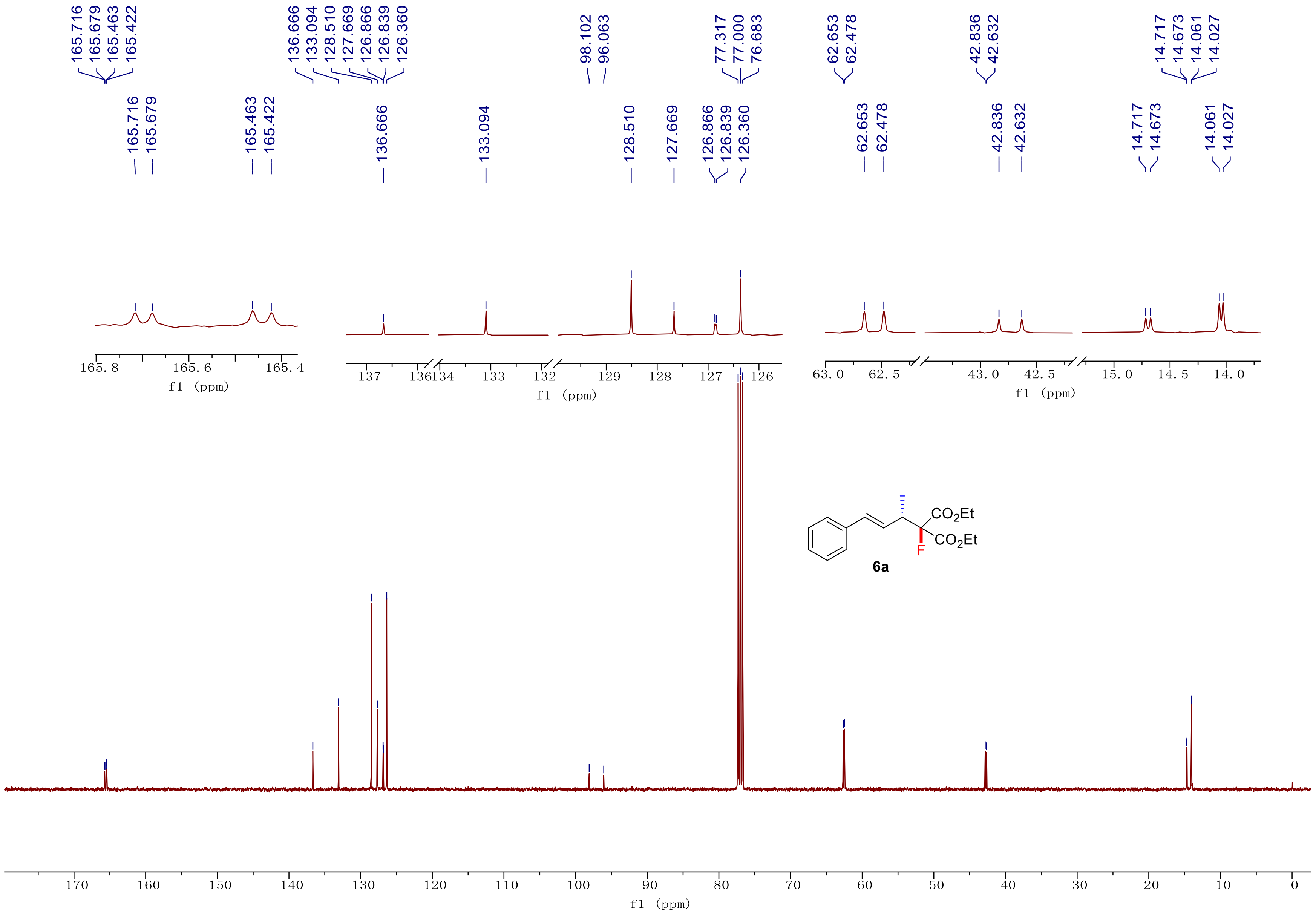

165.716
165.679
165.463
165.422
136.666
133.094
128.510
127.669
126.866
126.839
126.360
98.102
96.063
77.317
77.000
76.683
62.653
62.478
42.836
42.632
14.717
14.673
14.061
14.027
f1 (ppm)
CO_2Et
CO_2Et
F
6a

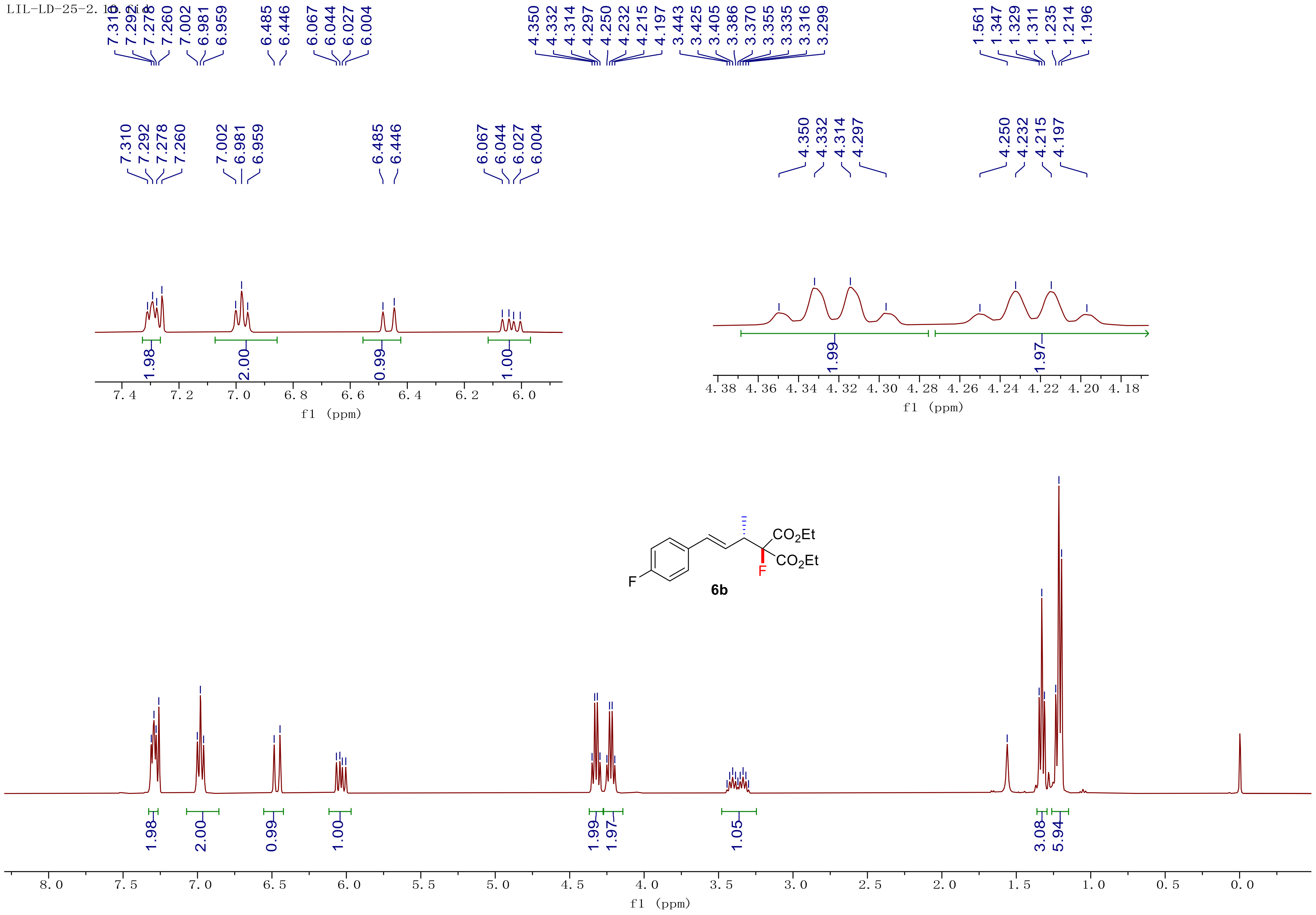
LIL-LD-25-2.1[illegible]
7.310 7.292 7.278 7.260 7.002 6.981 6.959 6.485 6.446 6.067 6.044 6.027 6.004
4.350 4.332 4.314 4.297 4.250 4.232 4.215 4.197 3.443 3.425 3.405 3.386 3.370 3.355 3.335 3.316 3.299
1.561 1.347 1.329 1.311 1.235 1.214 1.196
1.98 2.00 0.99 1.00
1.99 1.97
1.98 2.00 0.99 1.00 1.99 1.97 1.05 3.08 5.94
f1 (ppm)
CO_2Et
CO_2Et
F
F
6b

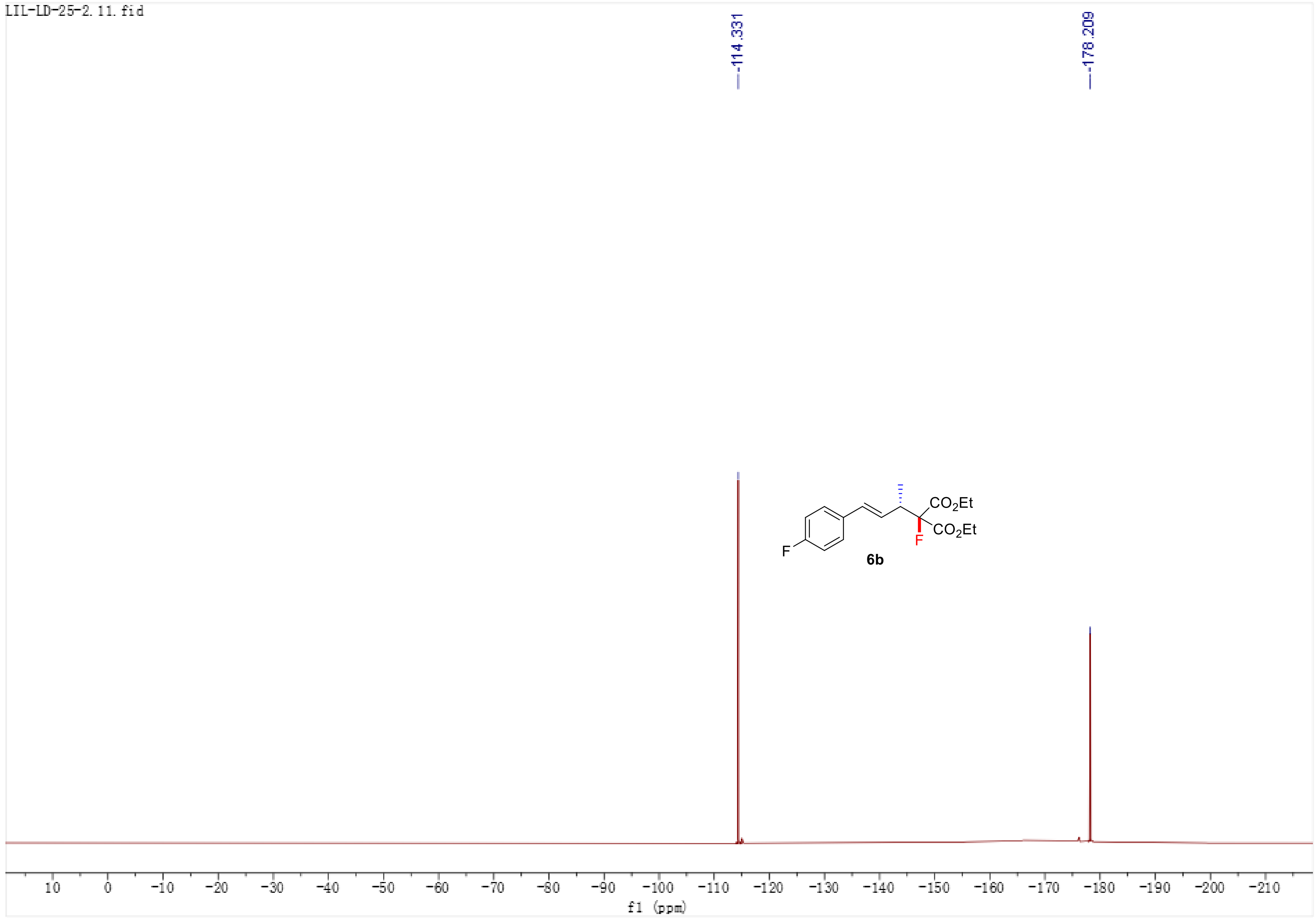
LIL-LD-25-2.11.fid
-114.331
-178.209
F
CO2Et
CO2Et
F
10
0
-10
-20
-30
-40
-50
-60
-70
-80
-90
-100
-110
-120
-130
-140
-150
-160
-170
-180
-190
-200
-210
f1 (ppm)

6b

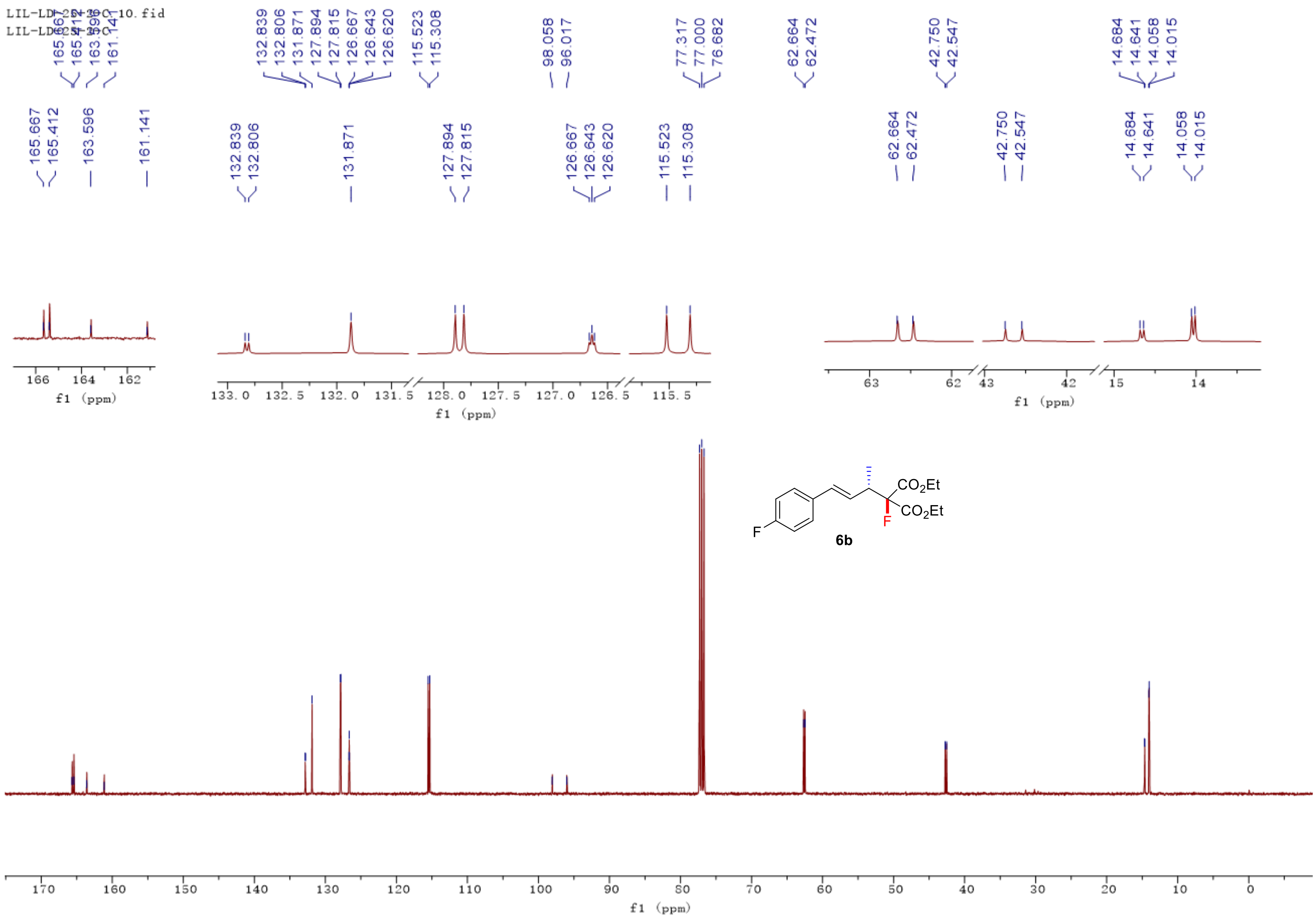

LIL-LD-25-3-C-10.fid
LIL-LD-25-3-C
165.667
165.412
163.596
161.141
132.839
132.806
131.871
127.894
127.815
126.667
126.643
126.620
115.523
115.308
98.058
96.017
77.317
77.000
76.682
62.664
62.472
42.750
42.547
14.684
14.641
14.058
14.015
f1 (ppm)
CO2Et
CO2Et
F
F
6b

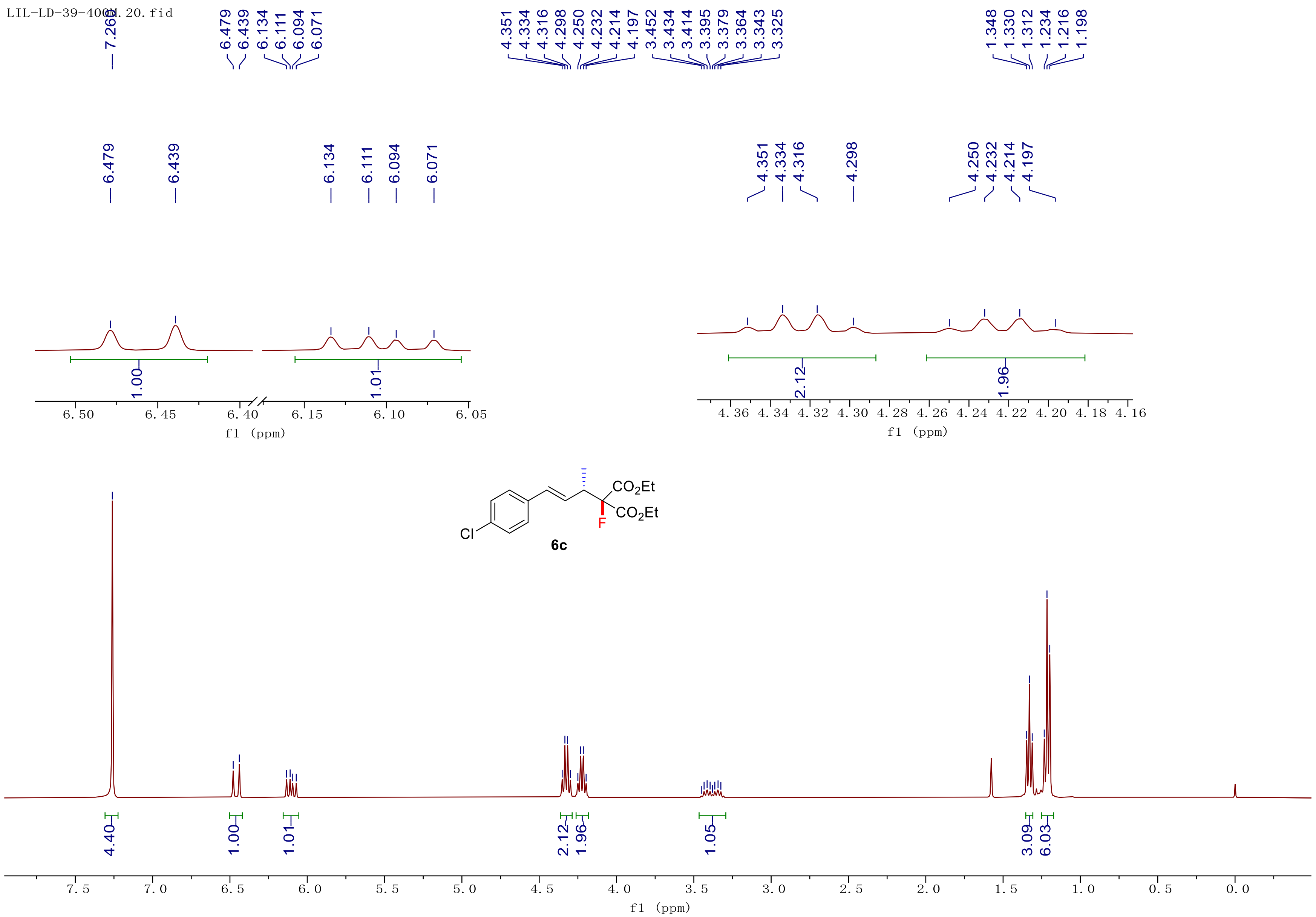
LIL-LD-39-400M.20.fid
7.260
6.479
6.439
6.134
6.111
6.094
6.071
4.351
4.334
4.316
4.298
4.250
4.232
4.214
4.197
3.452
3.434
3.414
3.395
3.379
3.364
3.343
3.325
1.348
1.330
1.312
1.234
1.216
1.198
1.00
1.01
2.12
1.96
f1 (ppm)
CO2Et
CO2Et
F
Cl
6c
4.40
1.00
1.01
2.12
1.96
1.05
3.09
6.03
f1 (ppm)

LIL-LE-39-1-400M.11.fid

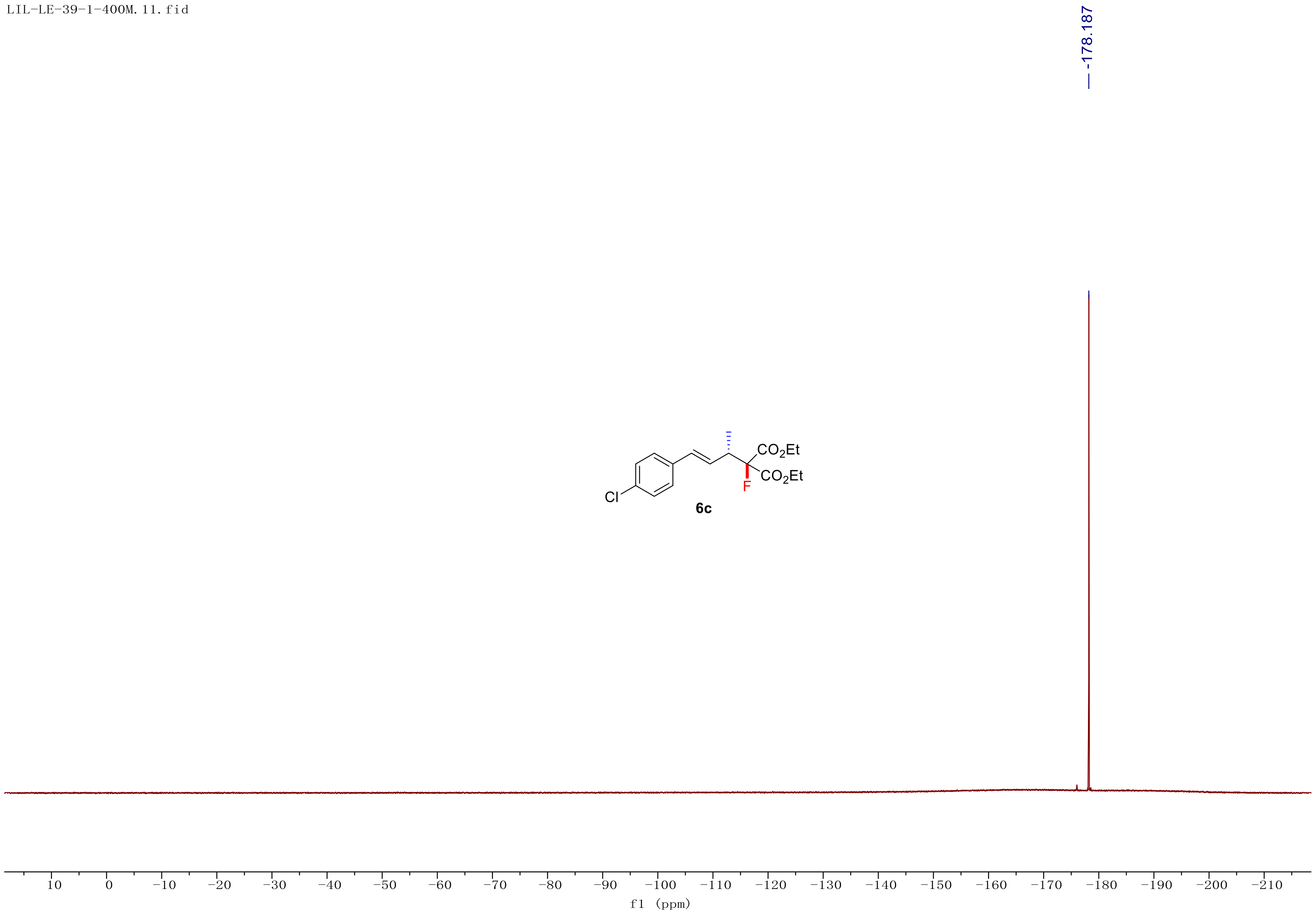

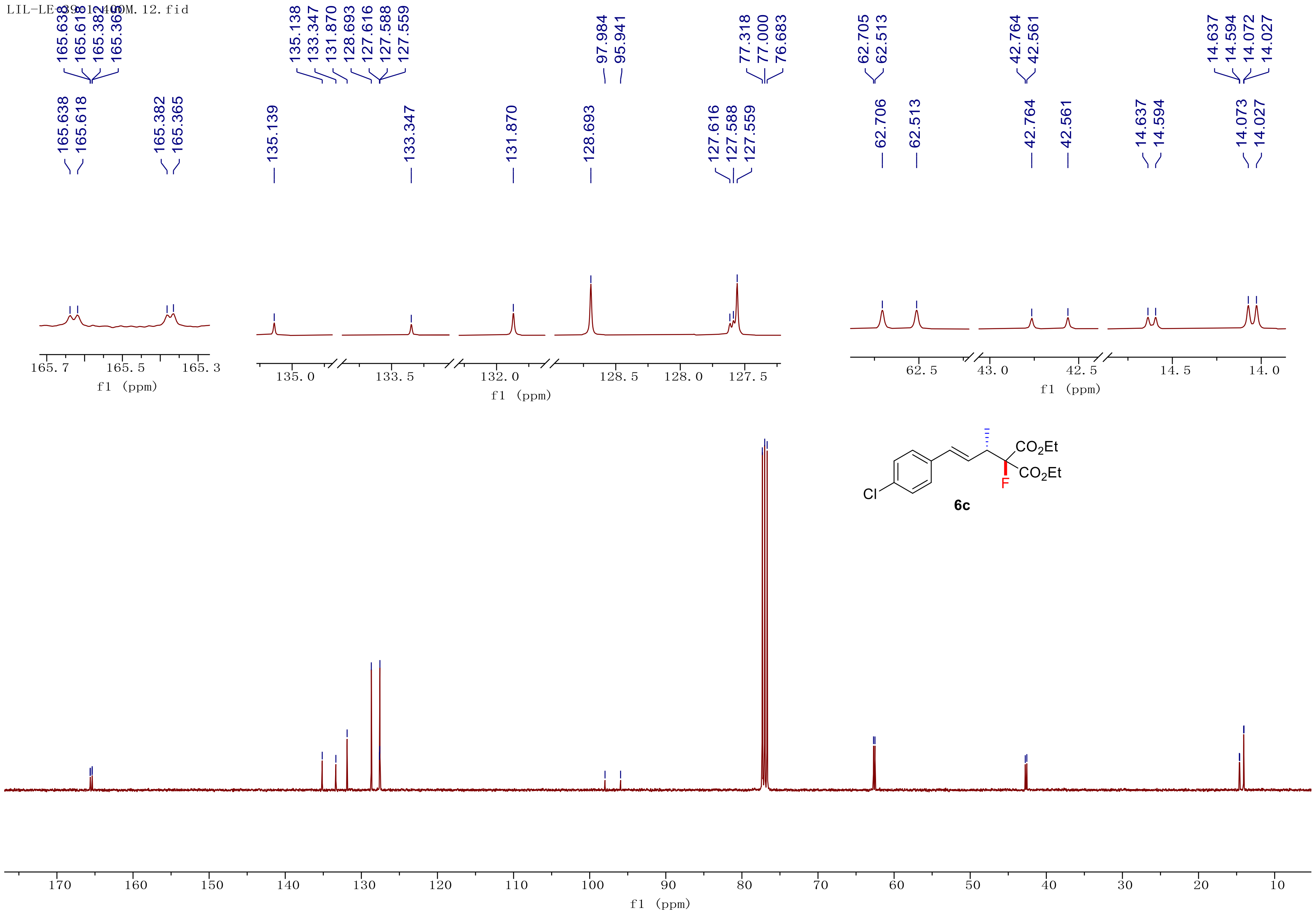
LIL-LE-3981-400M.12.fid
165.638
165.618
165.382
165.365
135.138
133.347
131.870
128.693
127.616
127.588
127.559
97.984
95.941
77.318
77.000
76.683
62.705
62.513
42.764
42.561
14.637
14.594
14.072
14.027
CO2Et
CO2Et
F
Cl
6c
f1 (ppm)

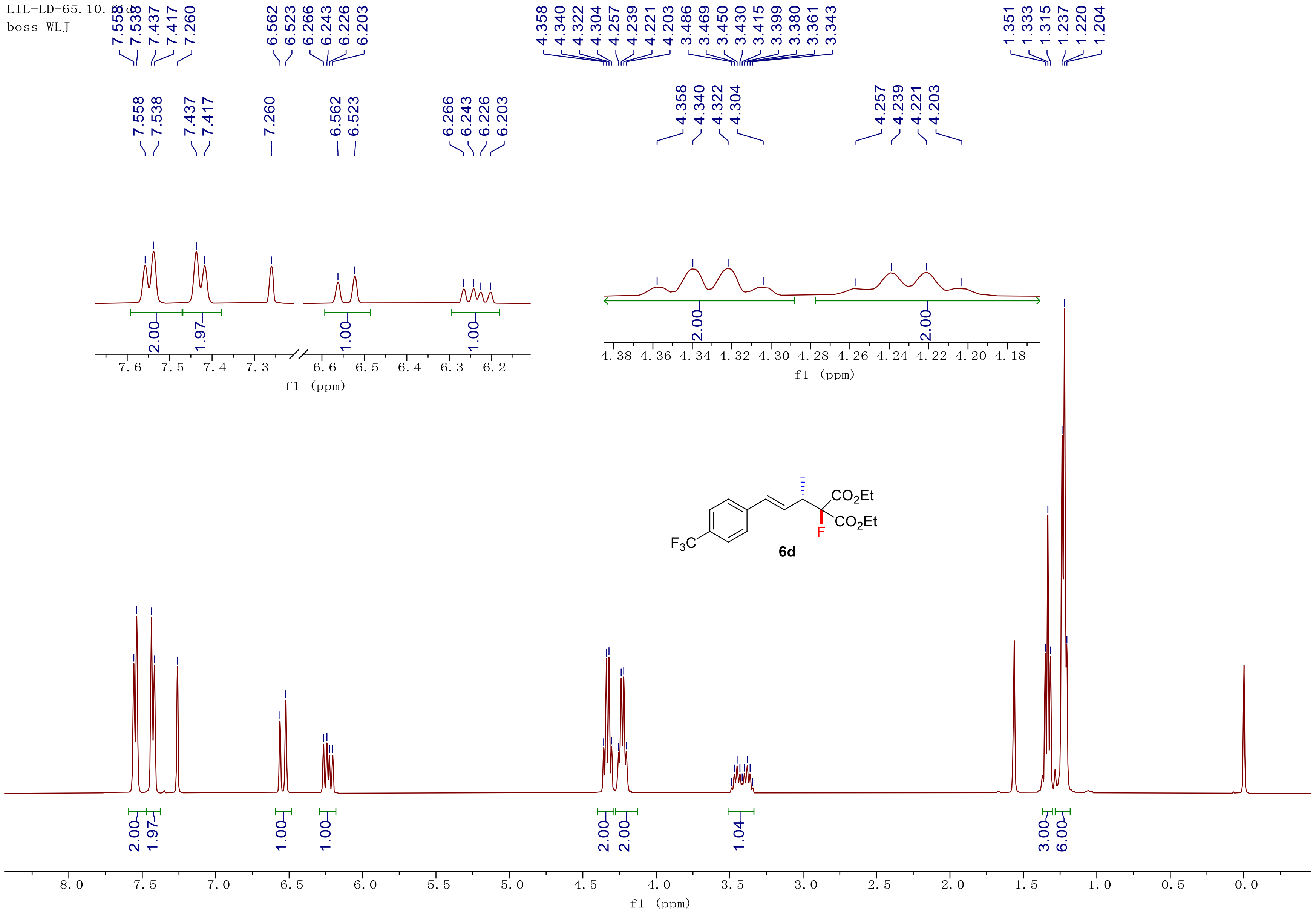
LIL-LD-65.10.fid
boss WLJ
7.558 7.538 7.437 7.417 7.260
6.562 6.523 6.266 6.243 6.226 6.203
4.358 4.340 4.322 4.304 4.257 4.239 4.221 4.203 3.486 3.469 3.450 3.430 3.415 3.399 3.380 3.361 3.343
1.351 1.333 1.315 1.237 1.220 1.204
CO2Et
CO2Et
F
F3C
6d
f1 (ppm)

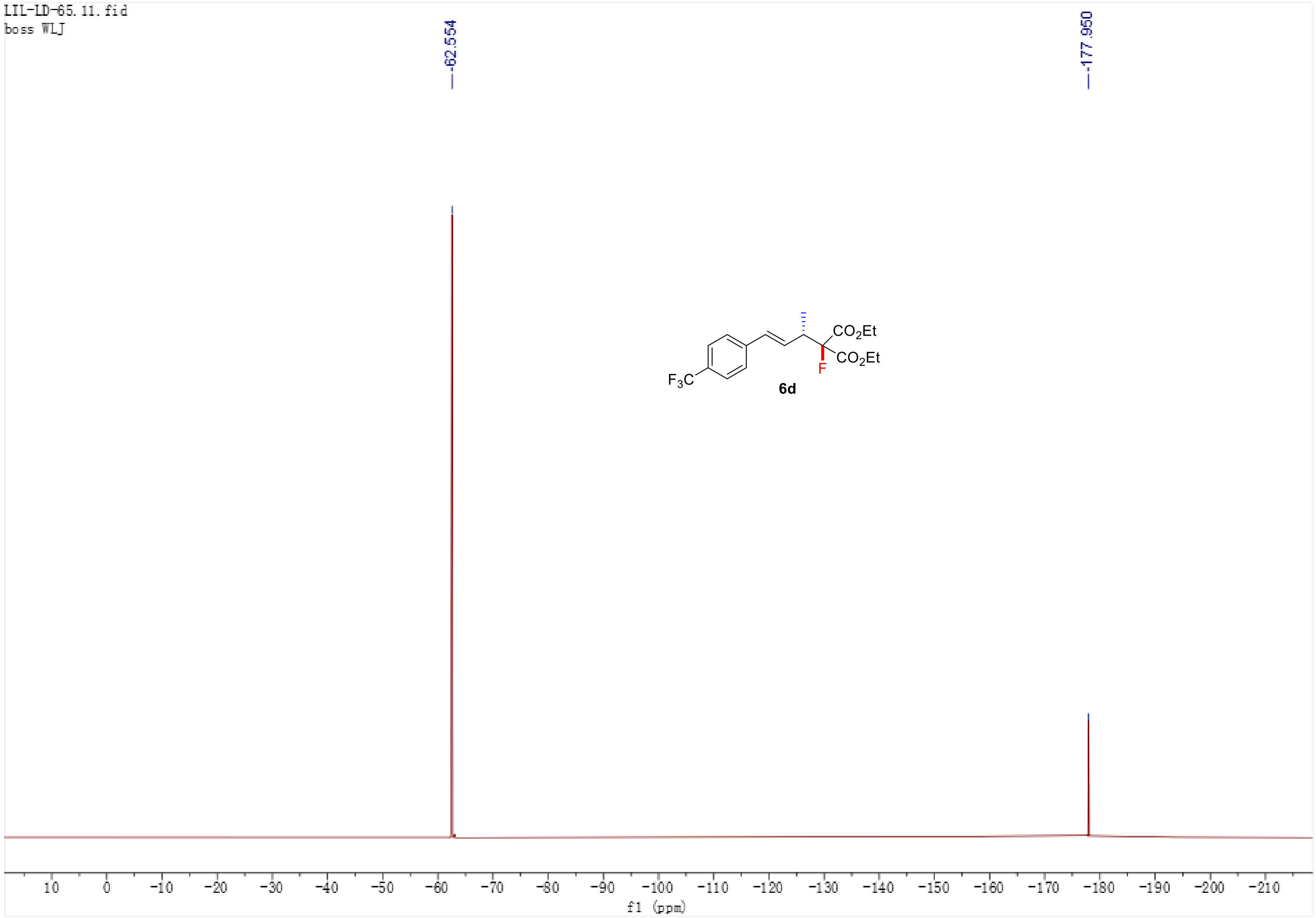

LIL-LD-65.11.fid
boss WLJ
-62.554
-177.950
F_3C
CO_2Et
CO_2Et
F
6d
f1 (ppm)

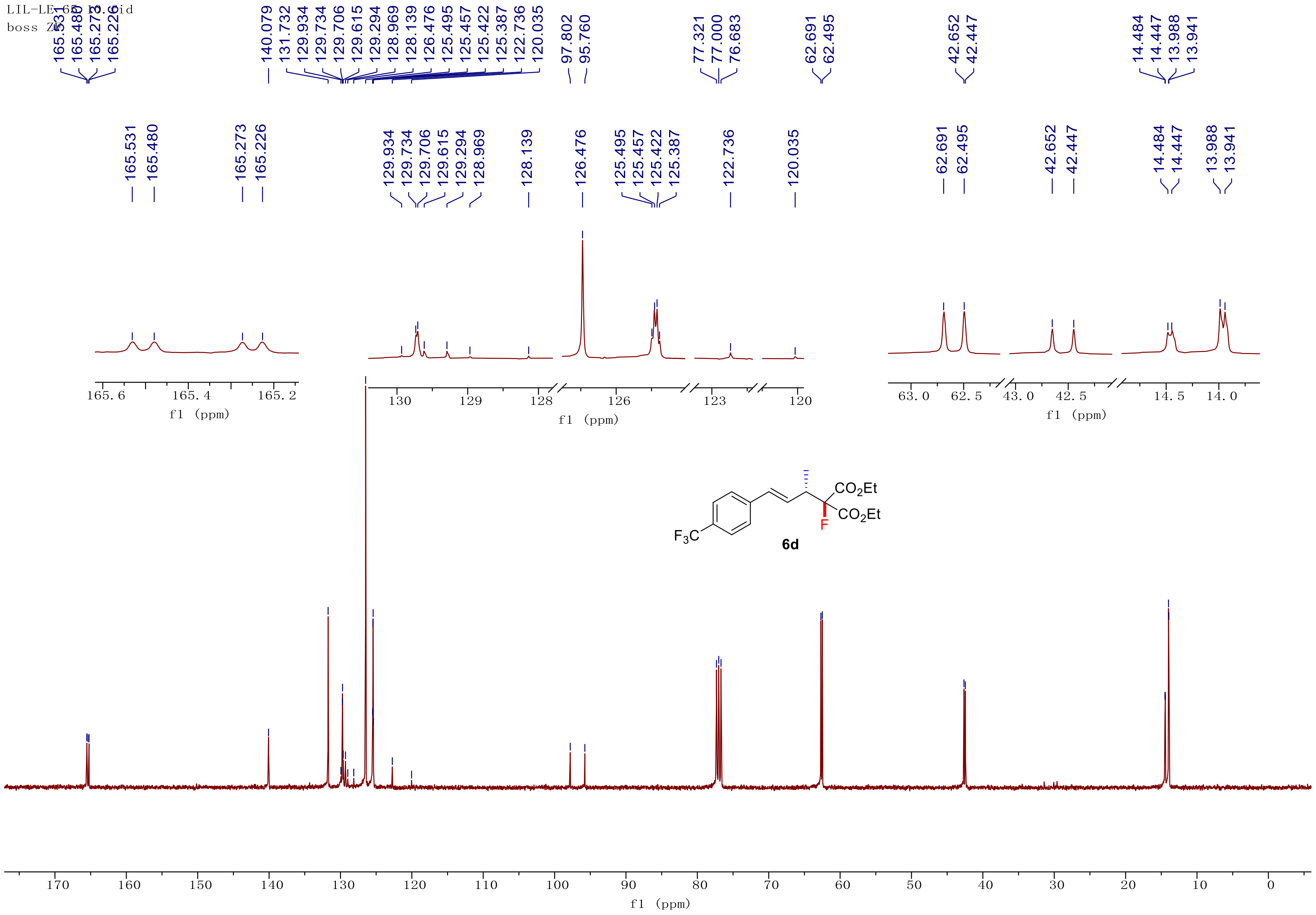
LIL-LE-65 10.fid
boss ZC
6d
f1 (ppm)

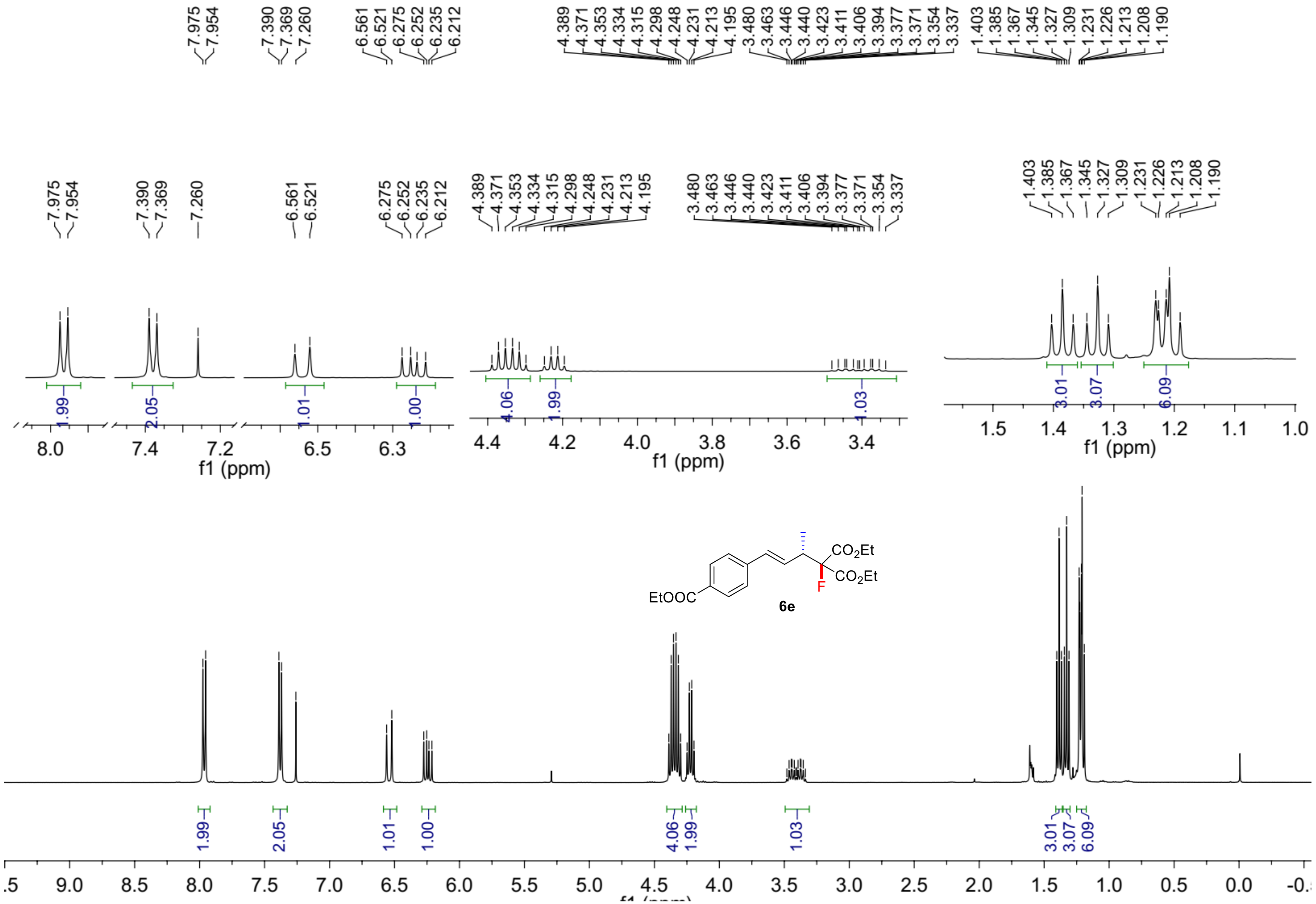
7.975
7.954
7.390
7.369
7.260
6.561
6.521
6.275
6.252
6.235
6.212
4.389
4.371
4.353
4.334
4.315
4.298
4.248
4.231
4.213
4.195
3.480
3.463
3.446
3.440
3.423
3.411
3.406
3.394
3.377
3.371
3.354
3.337
1.403
1.385
1.367
1.345
1.327
1.309
1.231
1.226
1.213
1.208
1.190
1.99
2.05
1.01
1.00
4.06
1.99
1.03
3.01
3.07
6.09
f1 (ppm)
CO_2Et
CO_2Et
F
EtOOC
6e

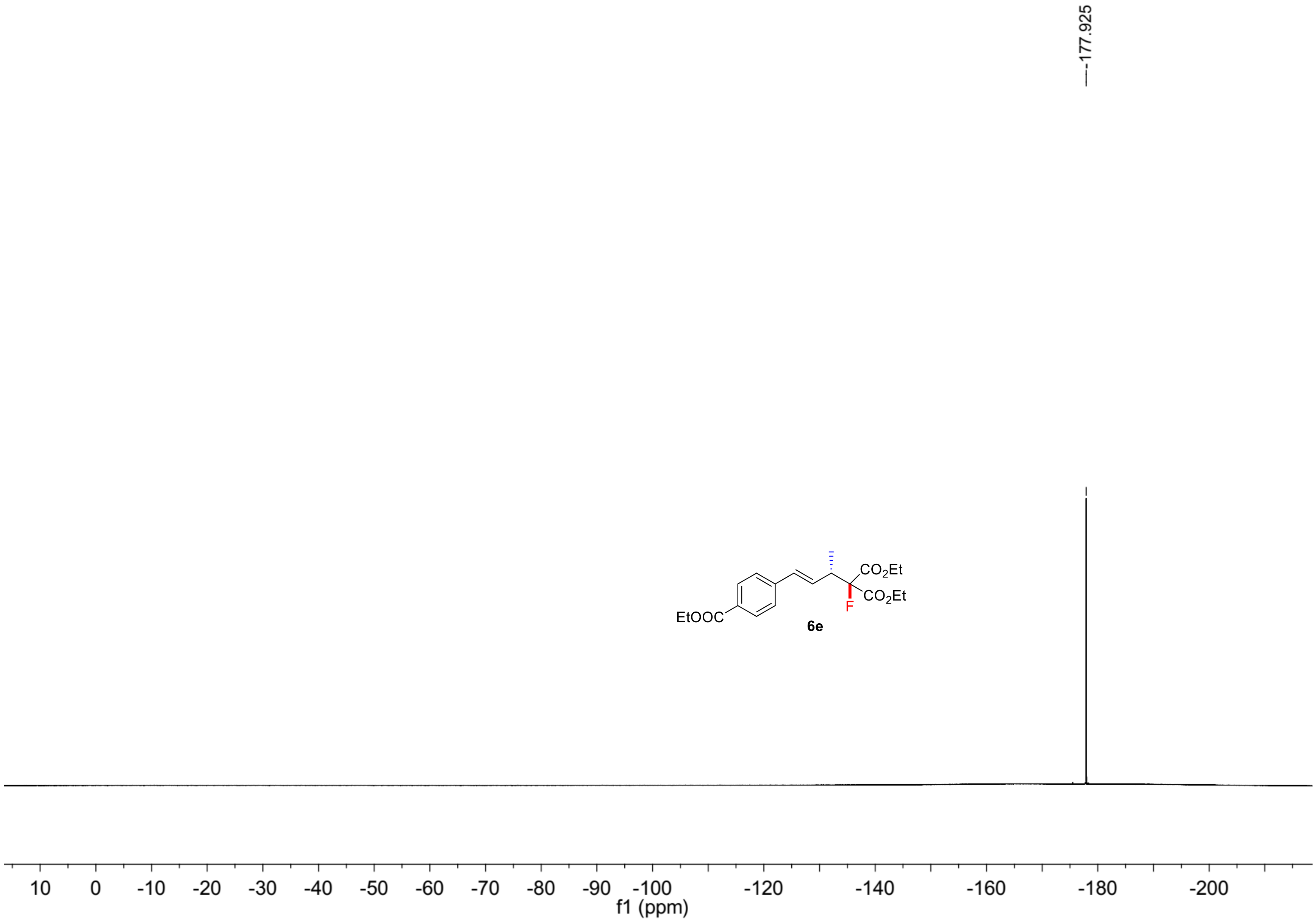
-177.925
EtOOC
CO_2Et
CO_2Et
F
6e
10 0 -10 -20 -30 -40 -50 -60 -70 -80 -90 -100 -120 -140 -160 -180 -200
f1 (ppm)

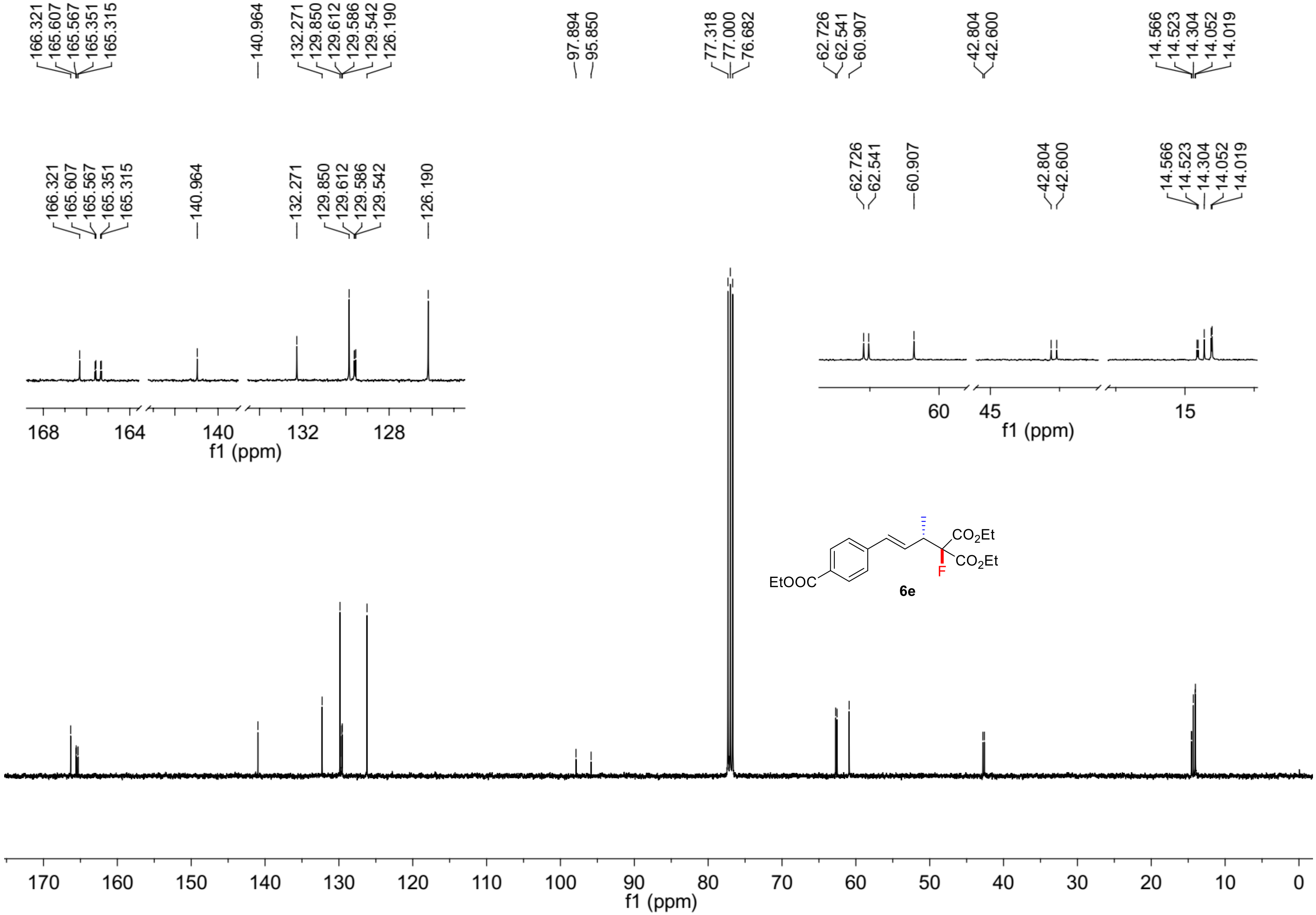

166.321
165.607
165.567
165.351
165.315
140.964
132.271
129.850
129.612
129.586
129.542
126.190
97.894
95.850
77.318
77.000
76.682
62.726
62.541
60.907
42.804
42.600
14.566
14.523
14.304
14.052
14.019
EtOOC
CO2Et
CO2Et
F
6e
f1 (ppm)

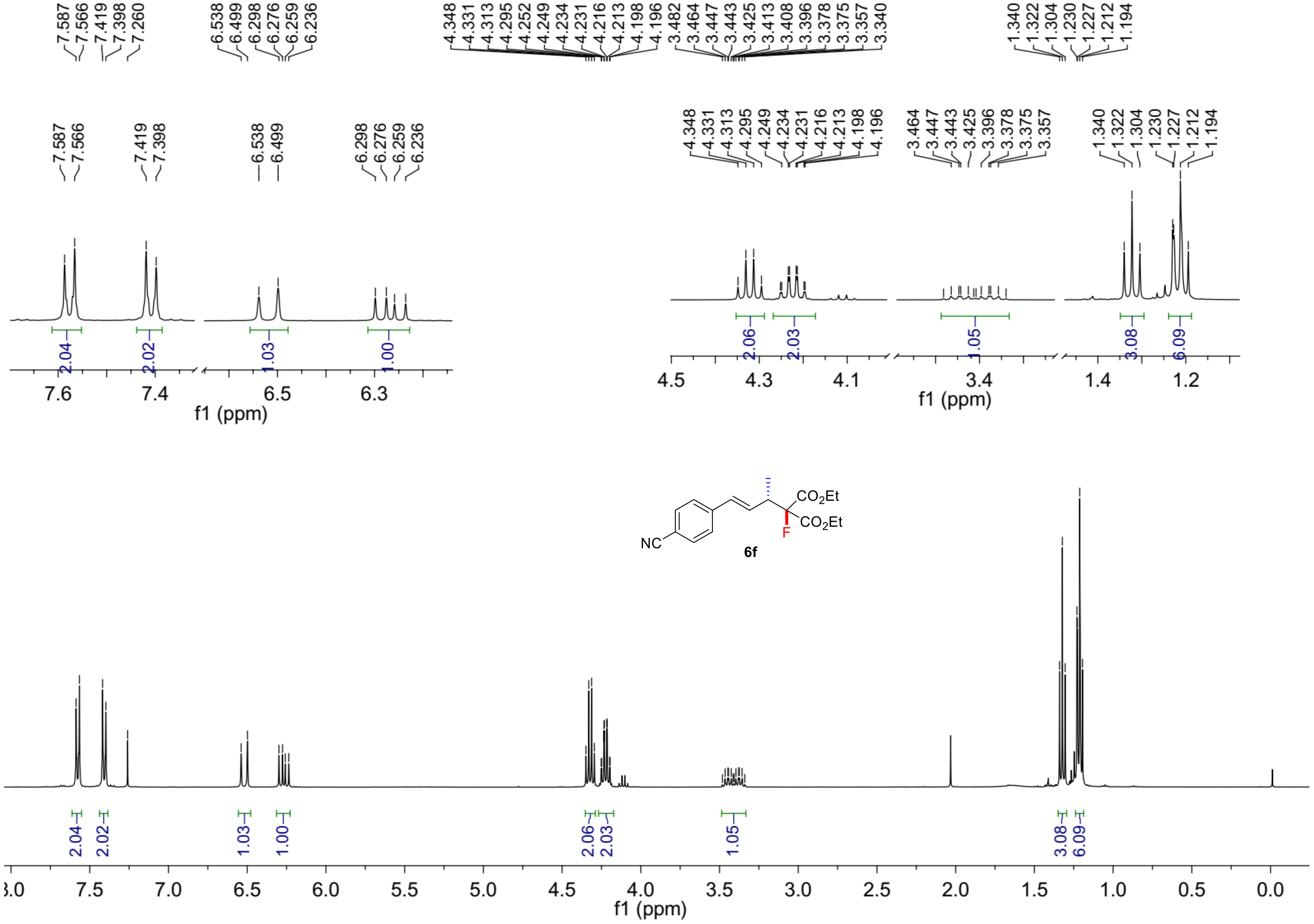

7.587
7.566
7.419
7.398
7.260
6.538
6.499
6.298
6.276
6.259
6.236
4.348
4.331
4.313
4.295
4.252
4.249
4.234
4.231
4.216
4.213
4.198
4.196
3.482
3.464
3.447
3.443
3.425
3.413
3.408
3.396
3.378
3.375
3.357
3.340
1.340
1.322
1.304
1.230
1.227
1.212
1.194
2.04
2.02
1.03
1.00
2.06
2.03
1.05
3.08
6.09
f1 (ppm)
NC
CO_2Et
CO_2Et
F
6f

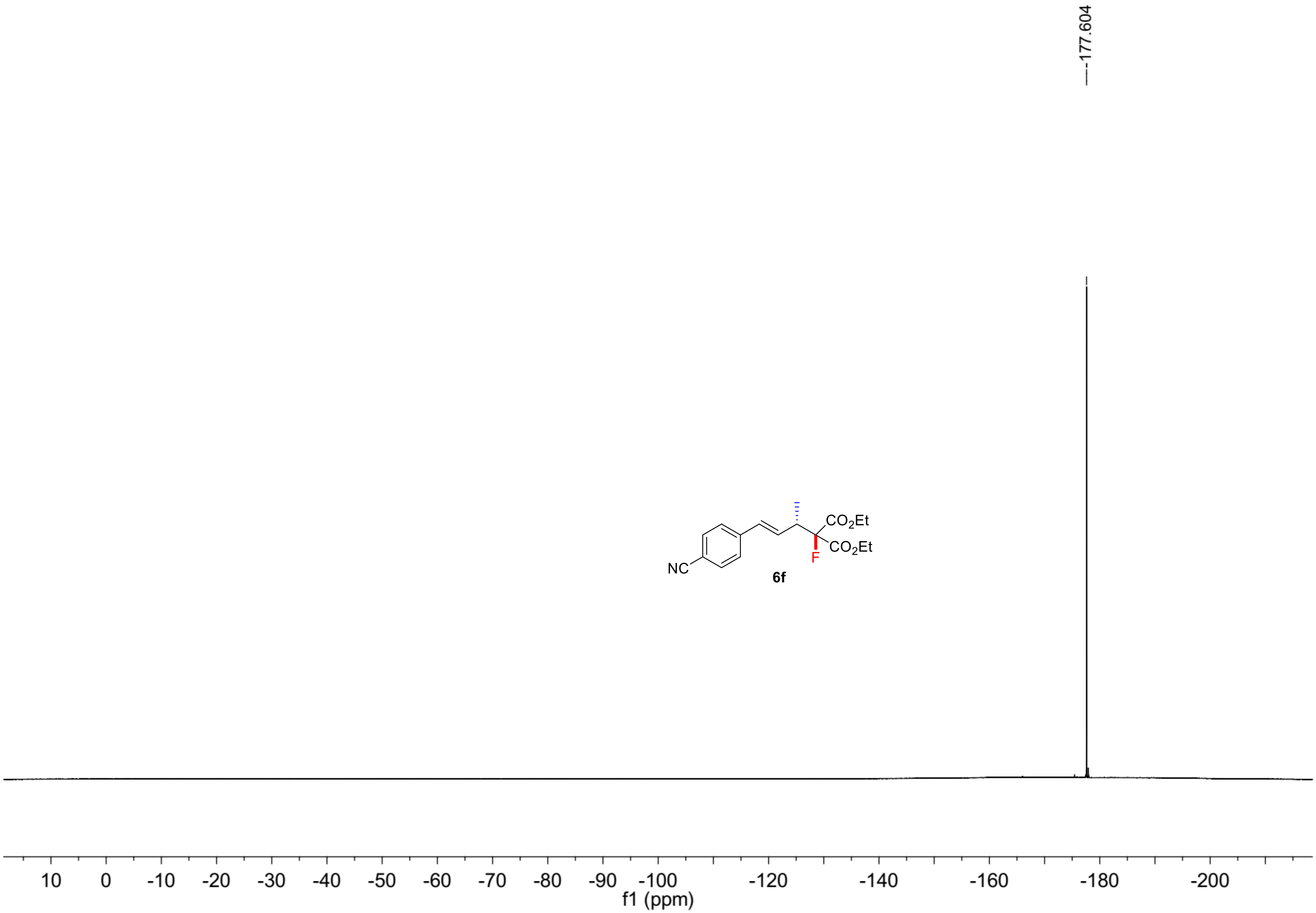

-177.604
CO2Et
CO2Et
F
NC
6f
10
0
-10
-20
-30
-40
-50
-60
-70
-80
-90
-100
-120
-140
-160
-180
-200
f1 (ppm)

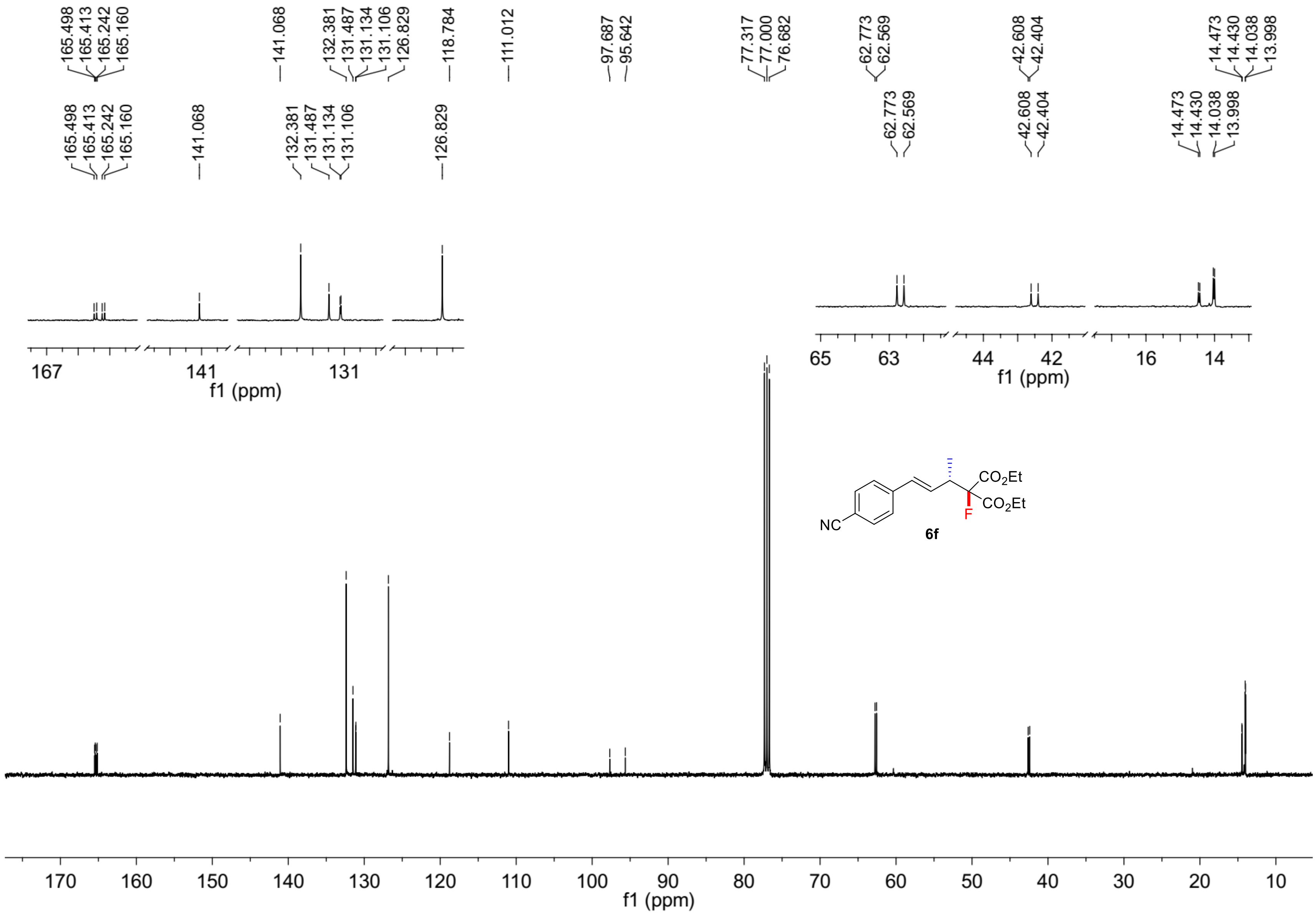

165.498
165.413
165.242
165.160
141.068
132.381
131.487
131.134
131.106
126.829
118.784
111.012
97.687
95.642
77.317
77.000
76.682
62.773
62.569
42.608
42.404
14.473
14.430
14.038
13.998
f1 (ppm)
CO2Et
CO2Et
F
NC
6f

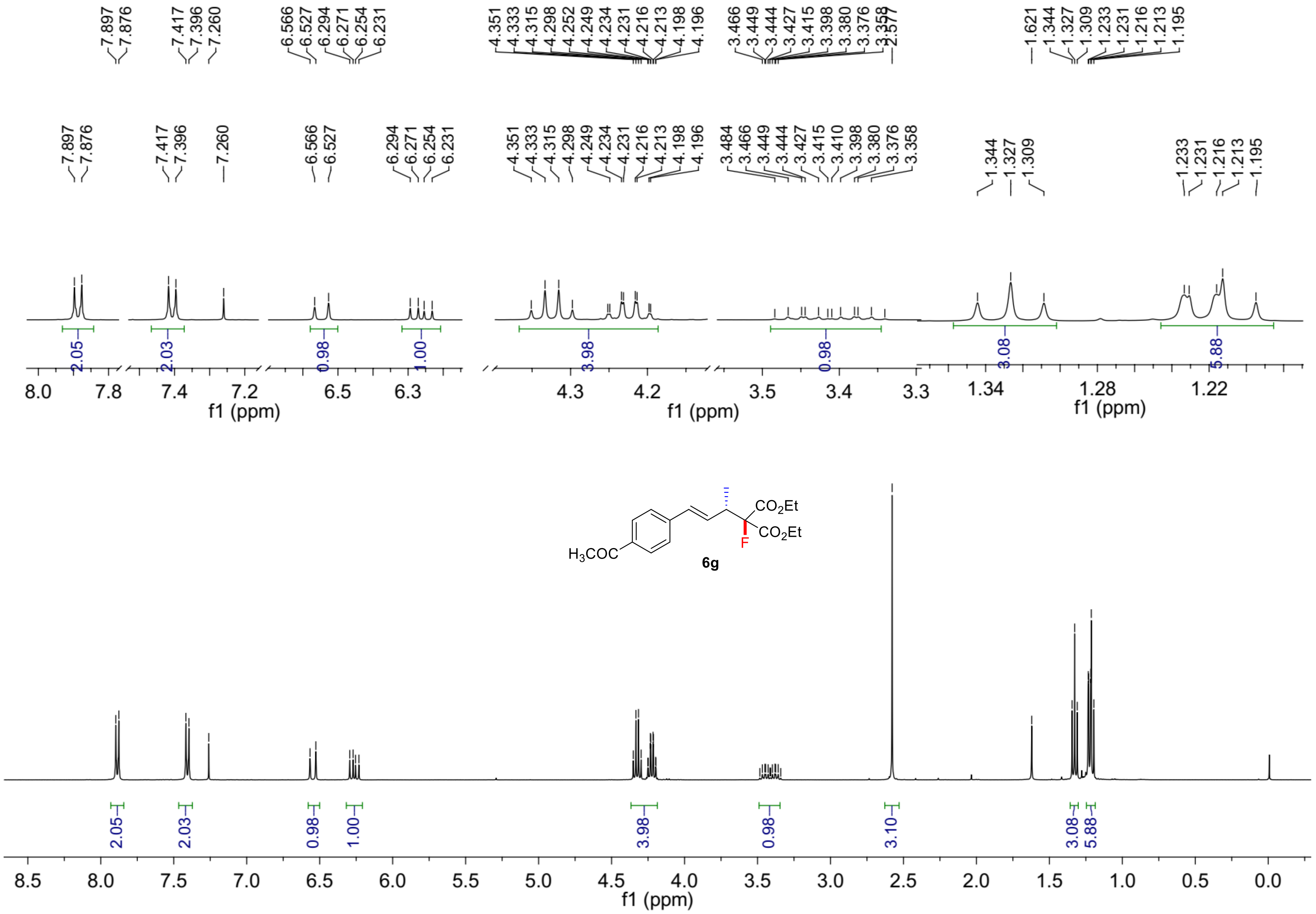
6g
f1 (ppm)

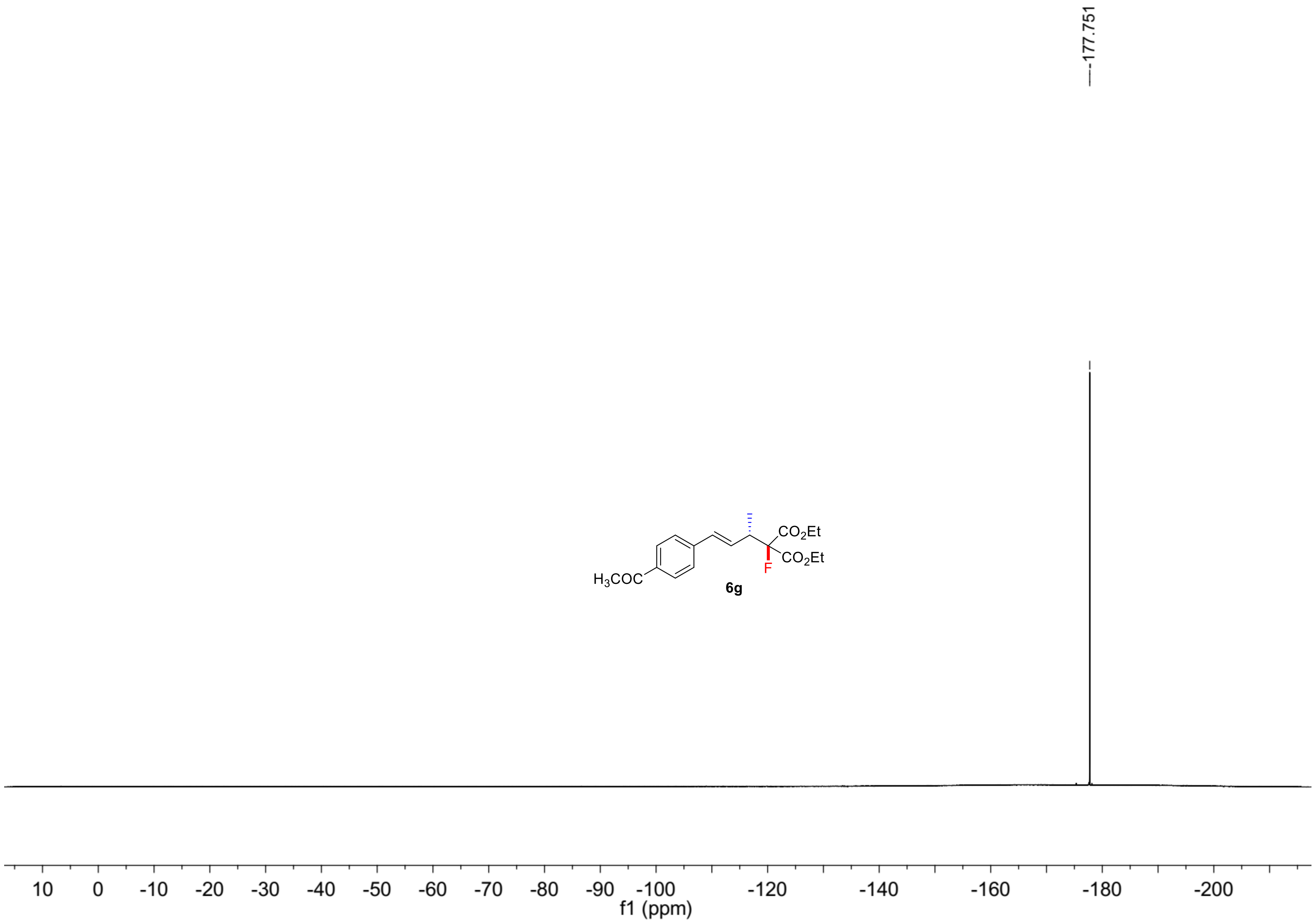

-177.751
H3COC
CO2Et
CO2Et
F
6g
10
0
-10
-20
-30
-40
-50
-60
-70
-80
-90
-100
-120
-140
-160
-180
-200
f1 (ppm)

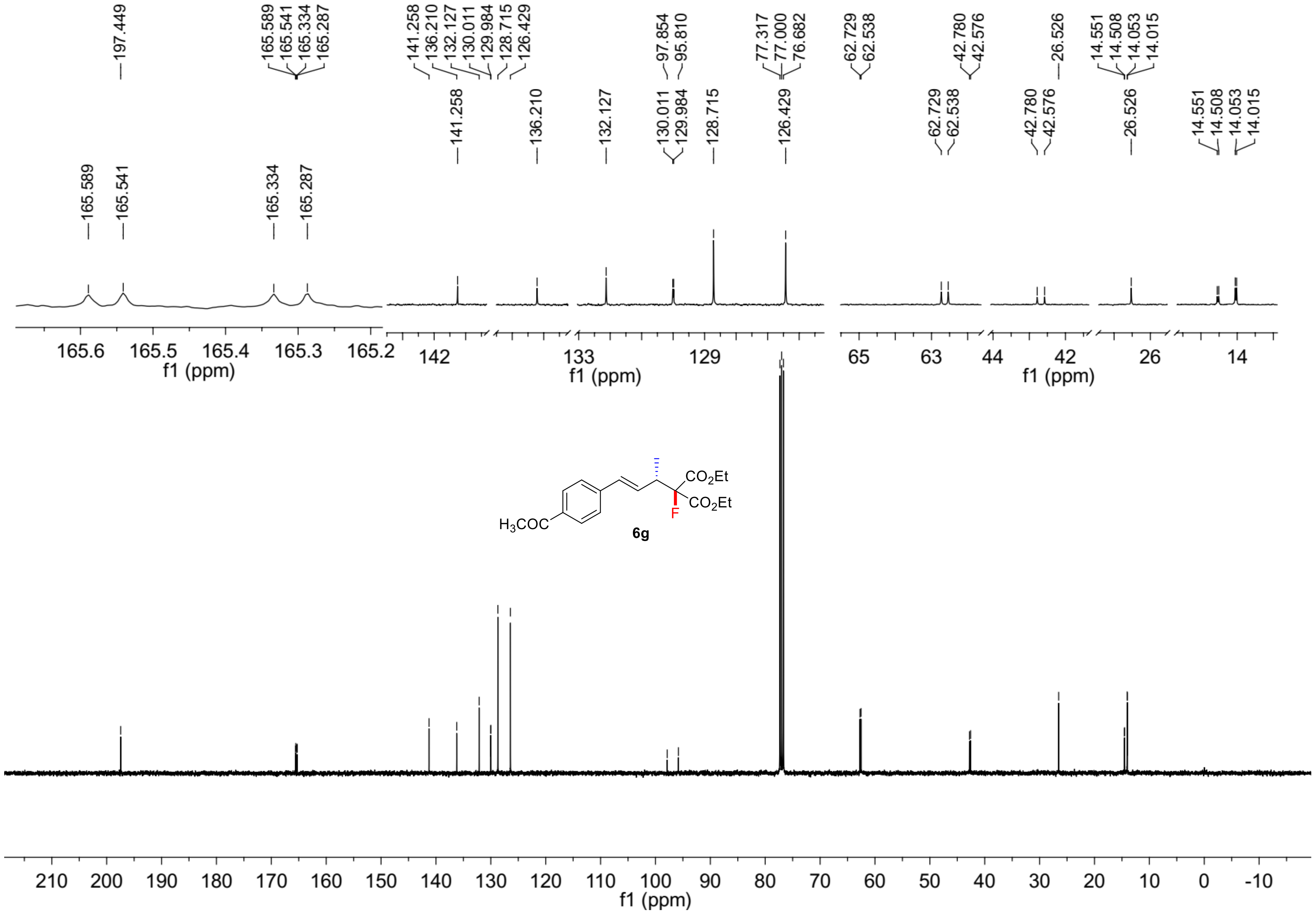
197.449
165.589
165.541
165.334
165.287
141.258
136.210
132.127
130.011
129.984
128.715
126.429
97.854
95.810
77.317
77.000
76.682
62.729
62.538
42.780
42.576
26.526
14.551
14.508
14.053
14.015
f1 (ppm)
CO2Et
CO2Et
F
H3COC
6g

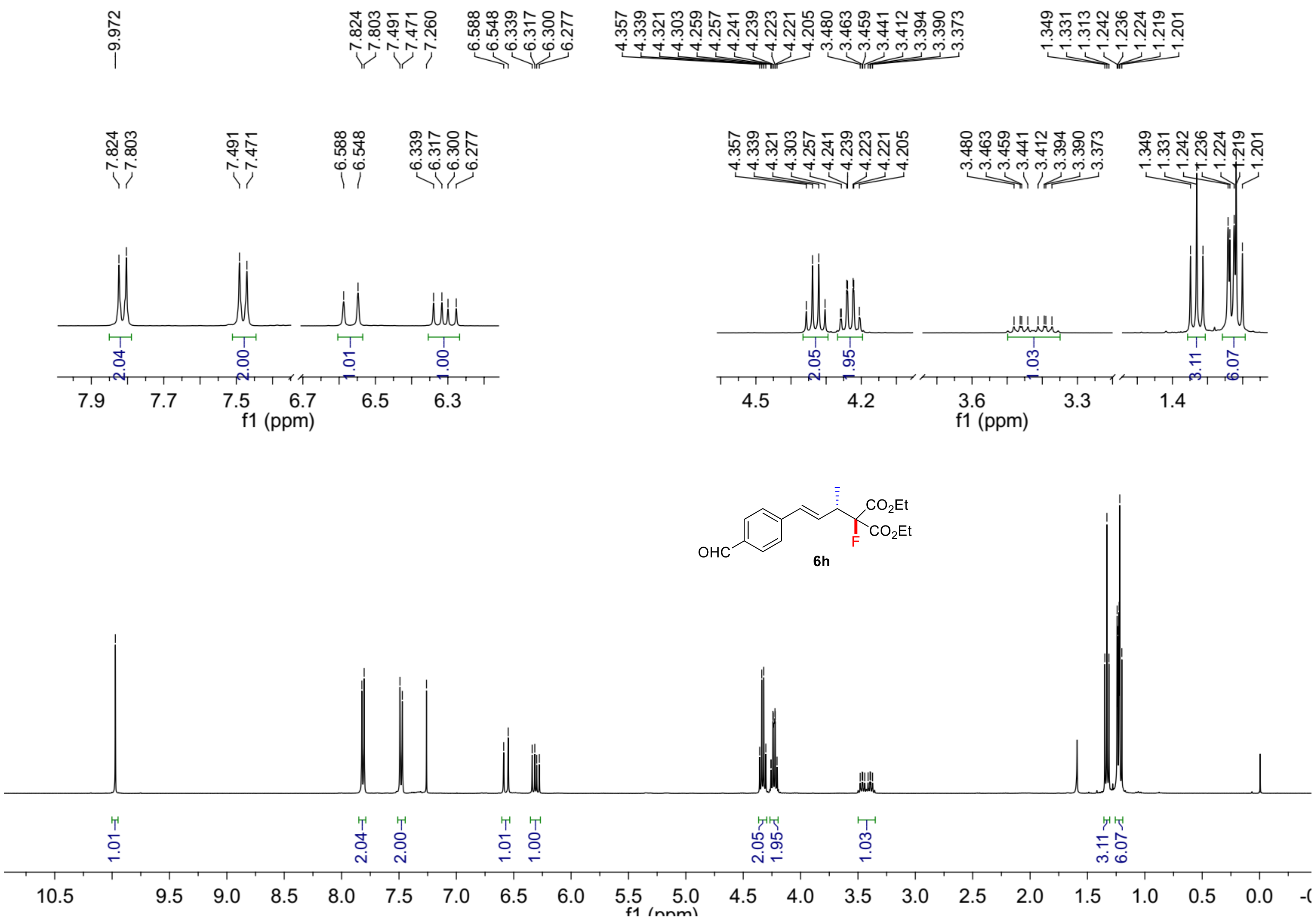
OHC
CO2Et
CO2Et
F
6h
f1 (ppm)

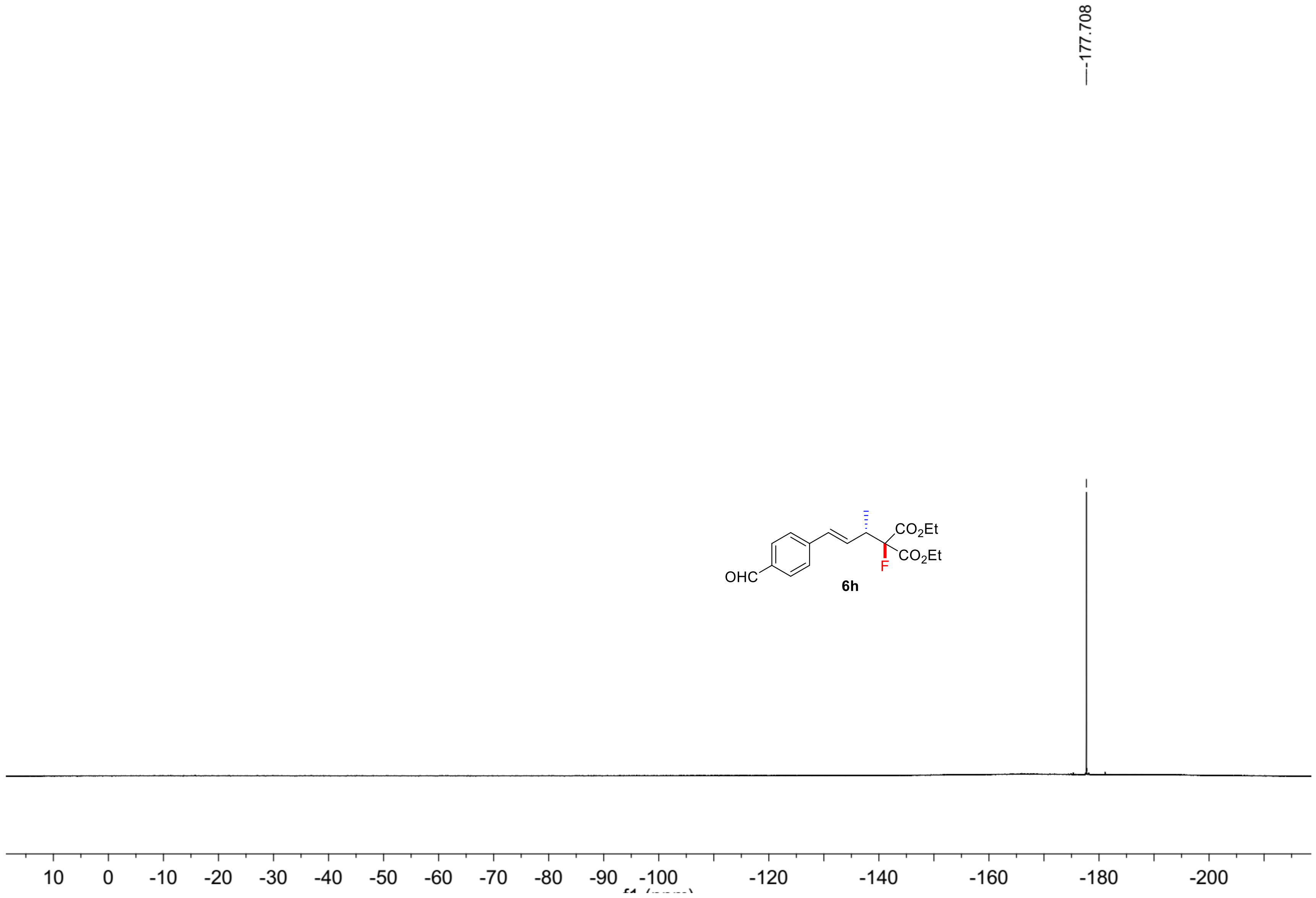
-177.708
OHC
CO2Et
CO2Et
F
6h
10 0 -10 -20 -30 -40 -50 -60 -70 -80 -90 -100 -120 -140 -160 -180 -200

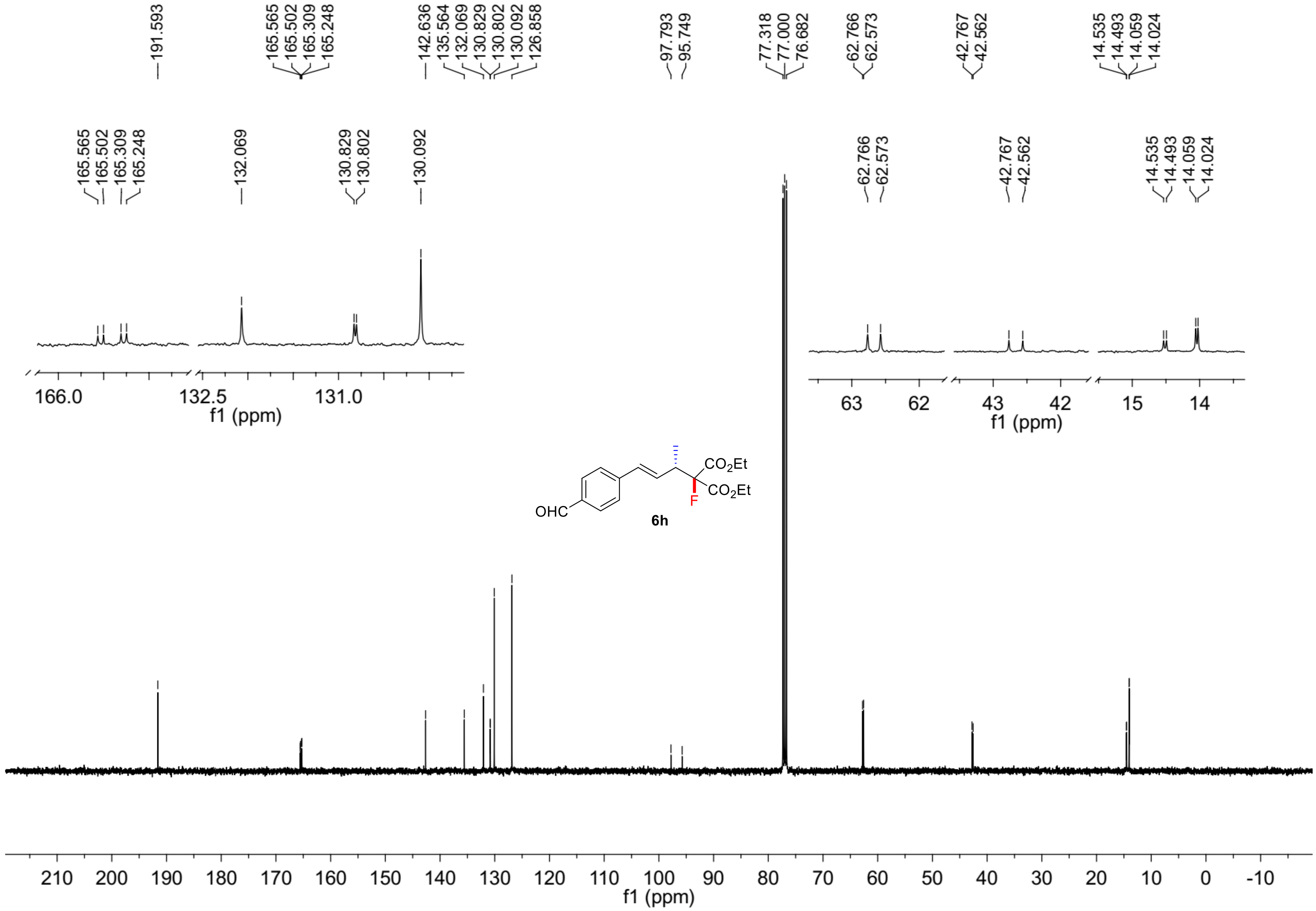
191.593
165.565
165.502
165.309
165.248
142.636
135.564
132.069
130.829
130.802
130.092
126.858
97.793
95.749
77.318
77.000
76.682
62.766
62.573
42.767
42.562
14.535
14.493
14.059
14.024
166.0
132.5
131.0
f1 (ppm)
63
62
43
42
15
14
f1 (ppm)
CO2Et
CO2Et
F
OHC
6h
210 200 190 180 170 160 150 140 130 120 110 100 90 80 70 60 50 40 30 20 10 0 -10
f1 (ppm)

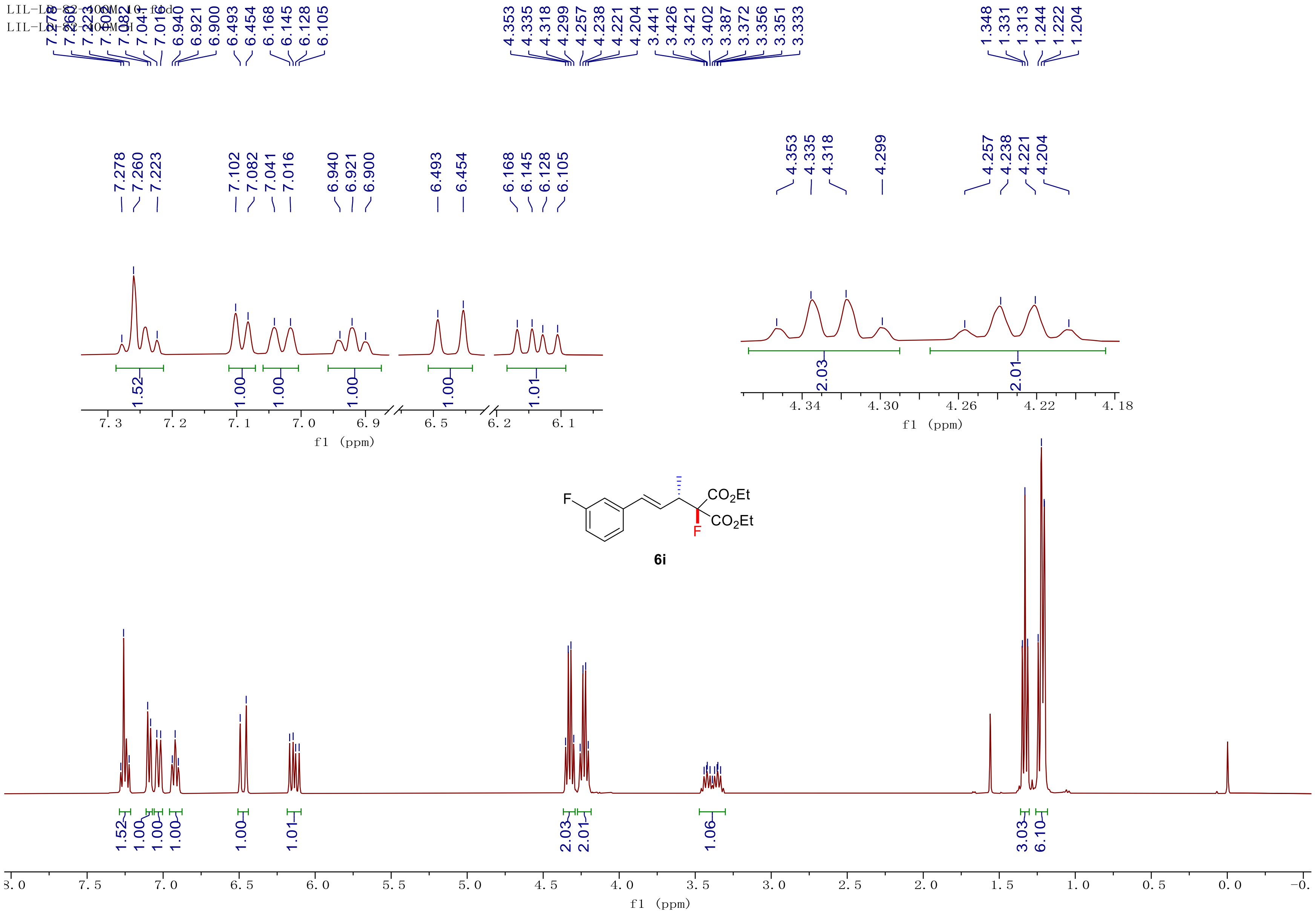

4.353
4.335
4.318
4.299
4.257
4.238
4.221
4.204
3.441
3.426
3.421
3.402
3.387
3.372
3.356
3.351
3.333
1.348
1.331
1.313
1.244
1.222
1.204
7.278
7.260
7.223
7.102
7.082
7.041
7.016
6.940
6.921
6.900
6.493
6.454
6.168
6.145
6.128
6.105
1.52
1.00
1.00
1.00
1.00
1.01
f1 (ppm)
2.03
2.01
f1 (ppm)
F
CO_2Et
CO_2Et
F
6i
1.52
1.00
1.00
1.00
1.00
1.01
2.03
2.01
1.06
3.03
6.10
f1 (ppm)

.

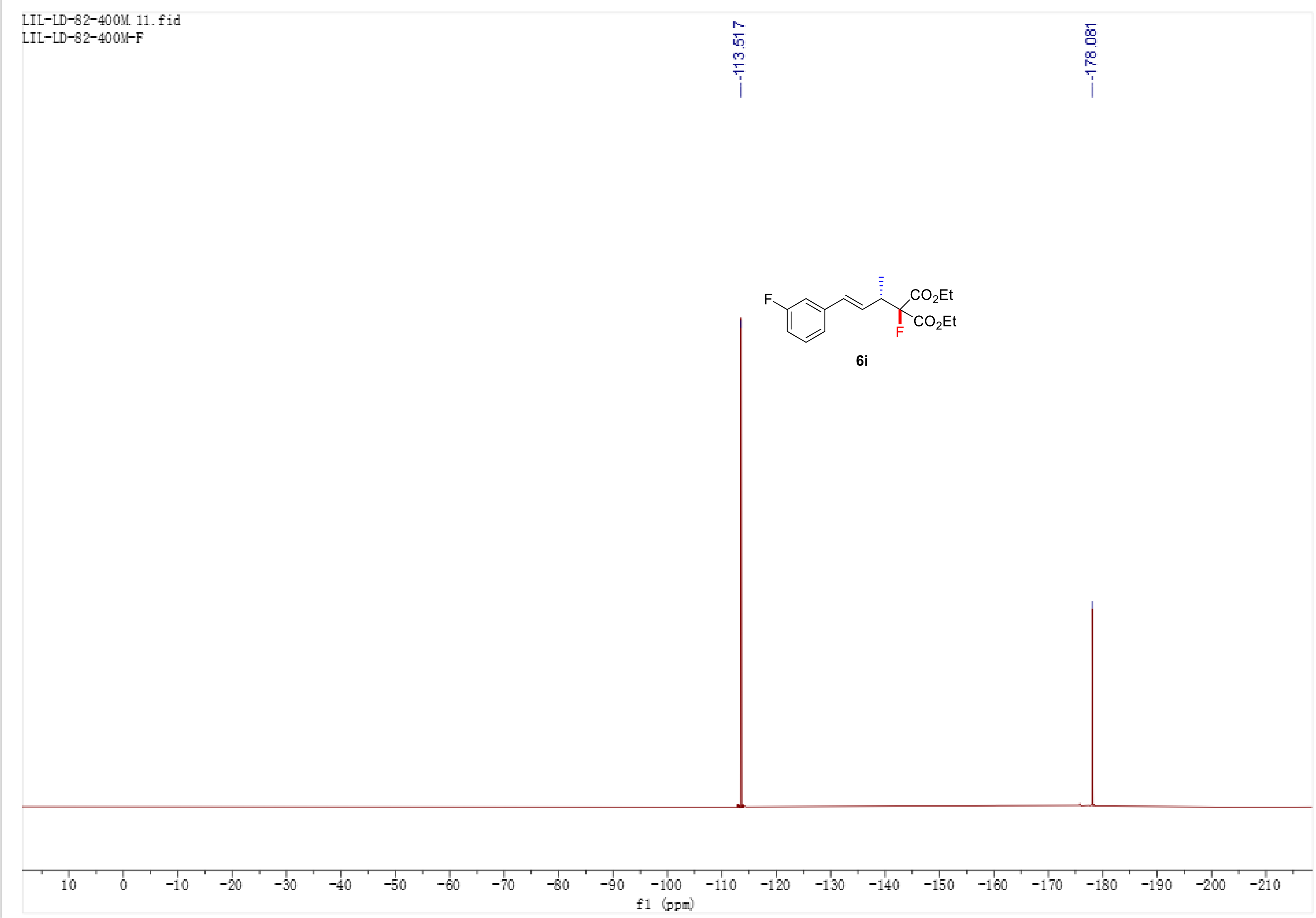

LIL-LD-82-400M.11.fid
LIL-LD-82-400M-F
-113.517
-178.081
F
CO2Et
CO2Et
F
6i
10
0
-10
-20
-30
-40
-50
-60
-70
-80
-90
-100
-110
-120
-130
-140
-150
-160
-170
-180
-190
-200
-210
f1 (ppm)

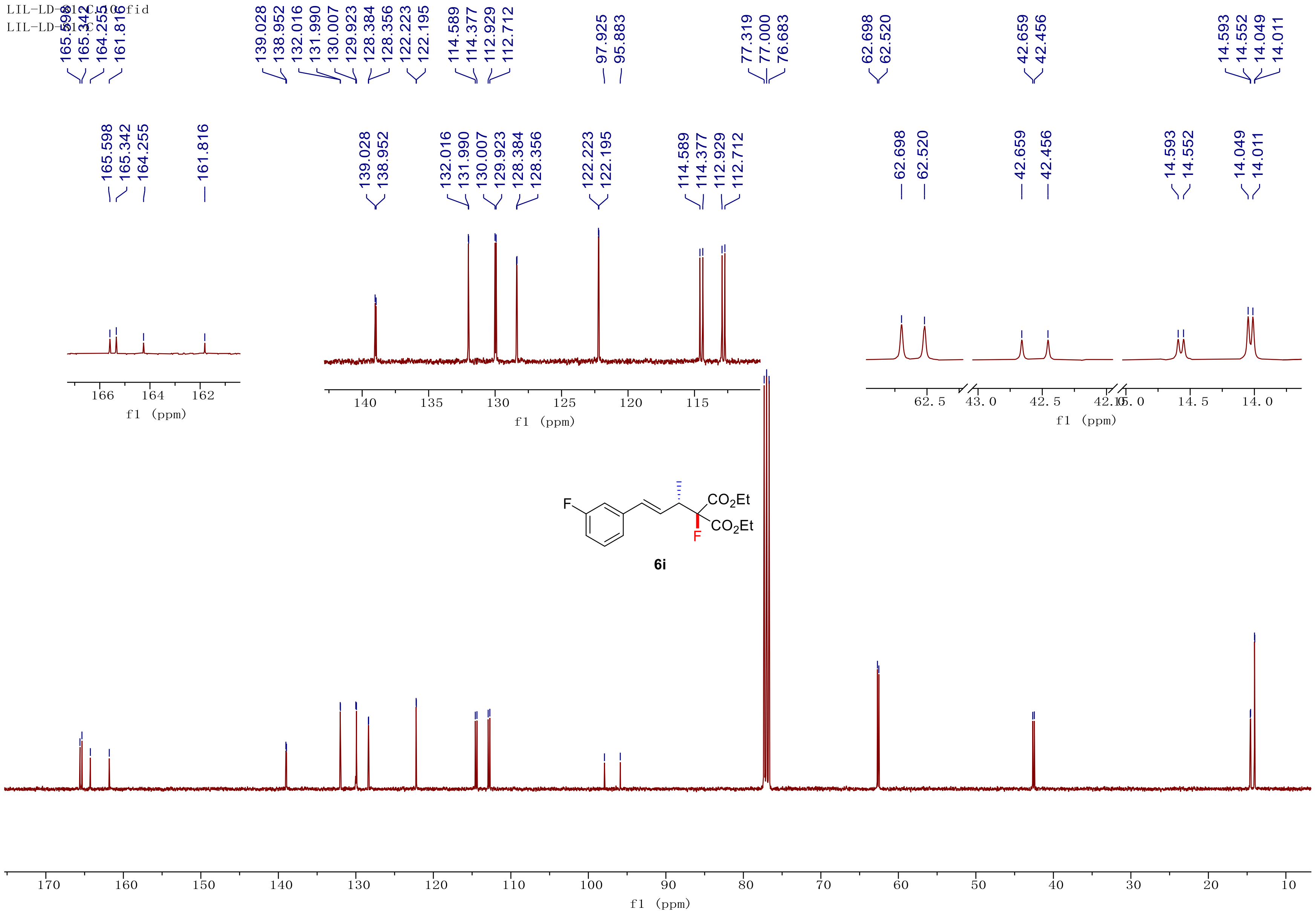
165.598
165.342
164.255
161.816
139.028
138.952
132.016
131.990
130.007
129.923
128.384
128.356
122.223
122.195
114.589
114.377
112.929
112.712
97.925
95.883
77.319
77.000
76.683
62.698
62.520
42.659
42.456
14.593
14.552
14.049
14.011
F
CO_2Et
CO_2Et
F
6i
f1 (ppm)

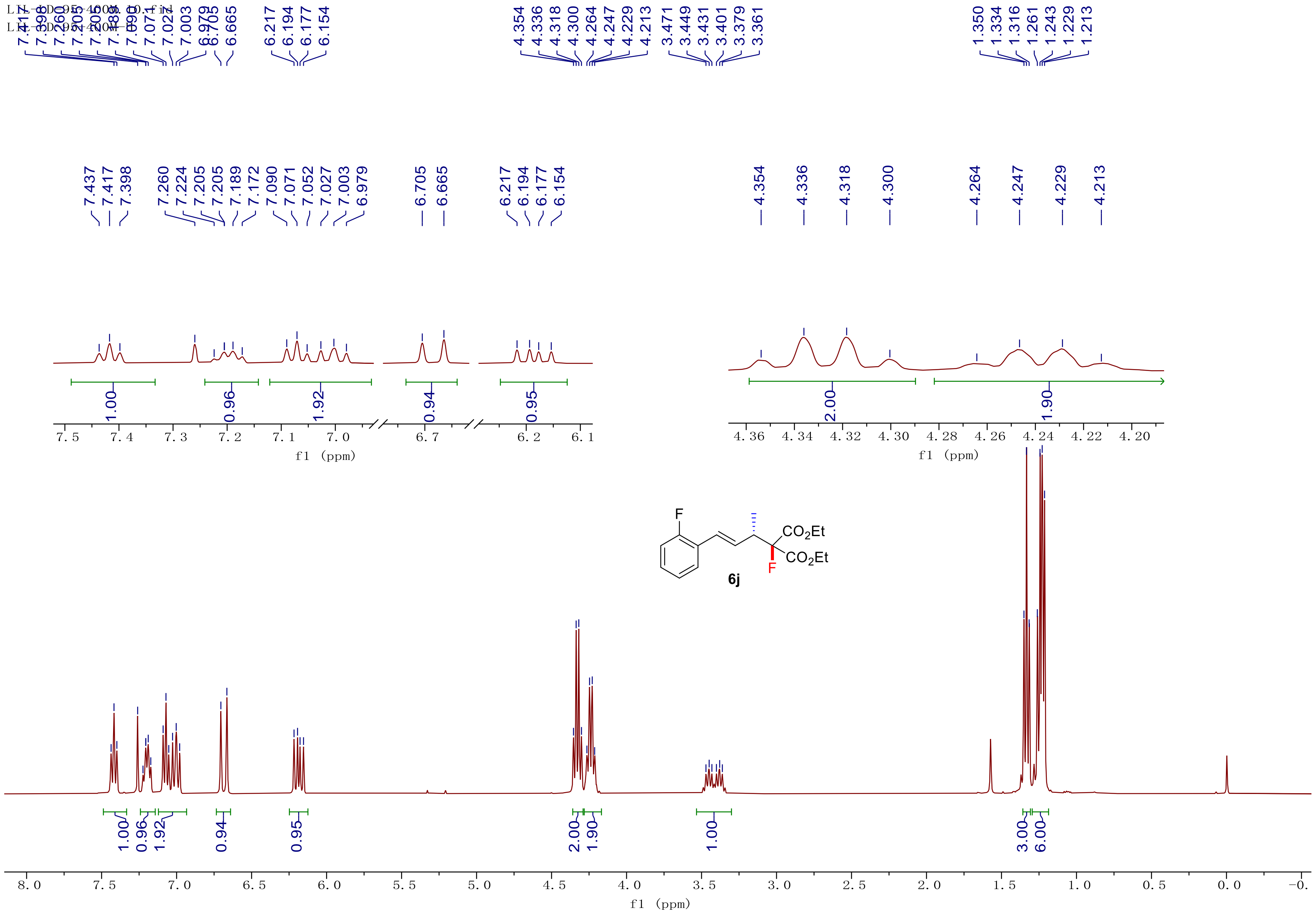
7.437 7.417 7.398 7.260 7.224 7.205 7.205 7.189 7.172 7.090 7.071 7.052 7.027 7.003 6.979 6.705 6.665 6.217 6.194 6.177 6.154
4.354 4.336 4.318 4.300 4.264 4.247 4.229 4.213 3.471 3.449 3.431 3.401 3.379 3.361
1.350 1.334 1.316 1.261 1.243 1.229 1.213
1.00 0.96 1.92 0.94 0.95
2.00 1.90
1.00 0.96 1.92 0.94 0.95 2.00 1.90 1.00 3.00 6.00
f1 (ppm)
F
CO2Et
CO2Et
F
6j

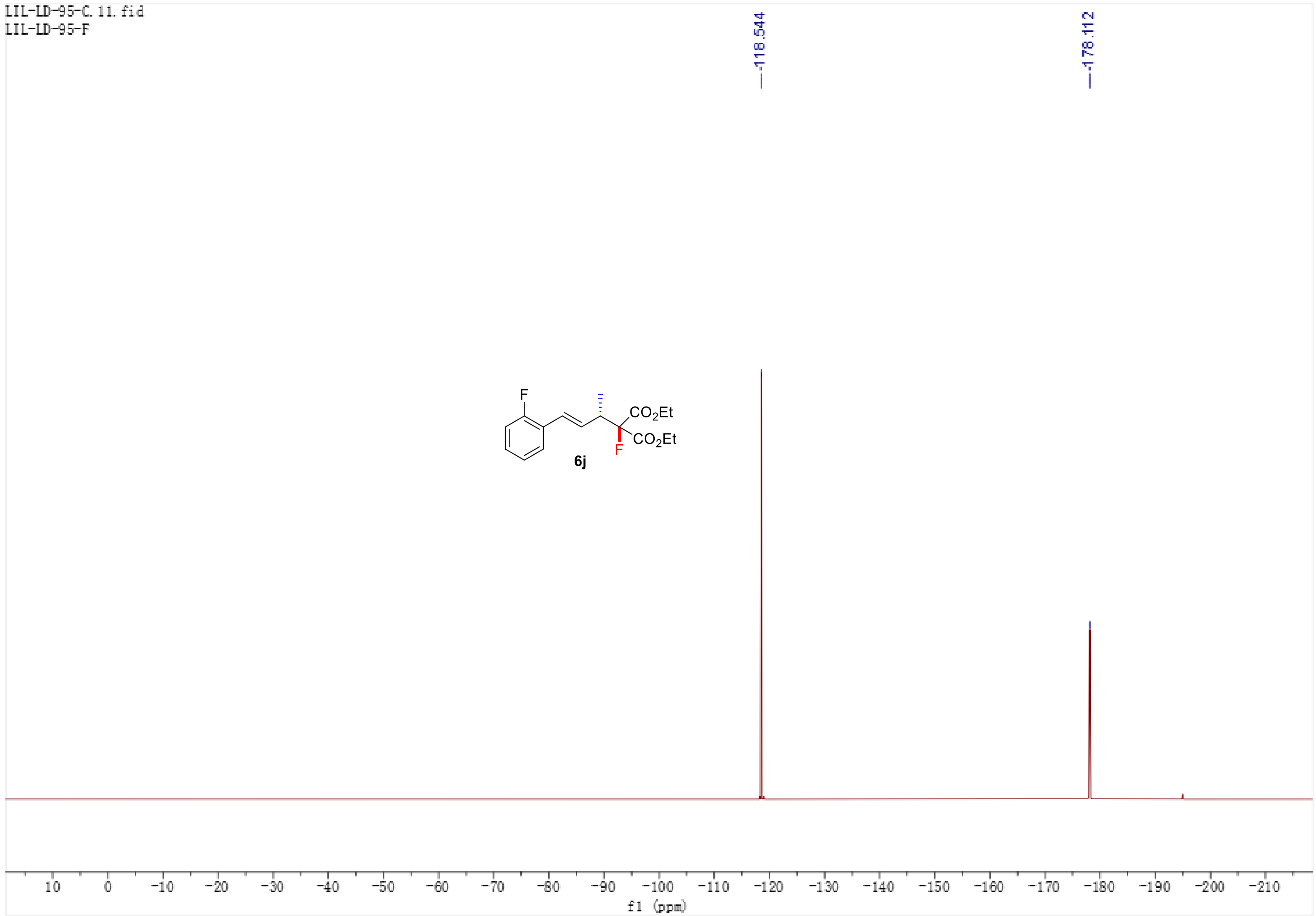

LIL-LD-95-C.11.fid
LIL-LD-95-F
-118.544
-178.112
F
CO2Et
CO2Et
F
6j
10 0 -10 -20 -30 -40 -50 -60 -70 -80 -90 -100 -110 -120 -130 -140 -150 -160 -170 -180 -190 -200 -210
f1 (ppm)

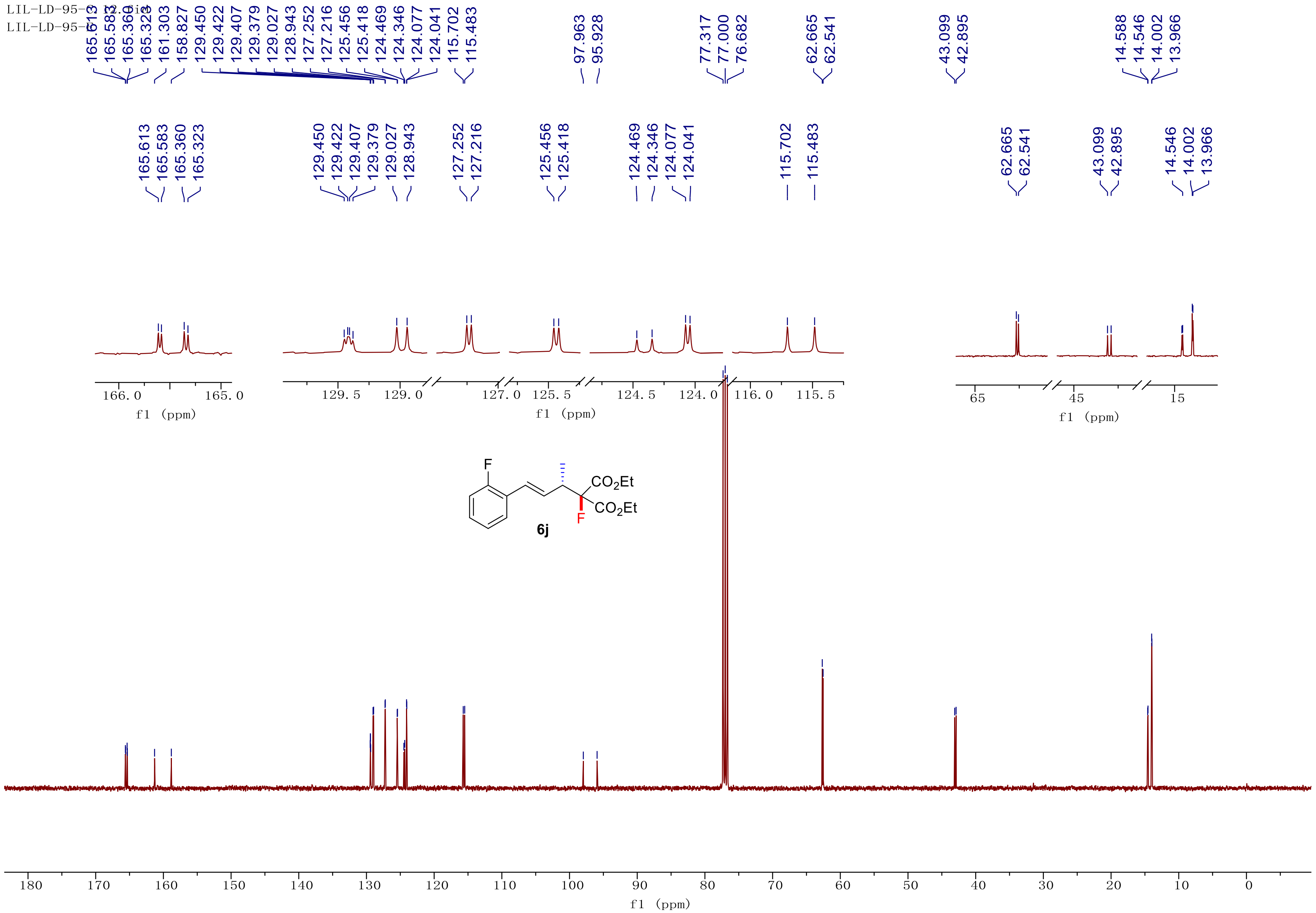
LIL-LD-95-[illegible]
LIL-LD-95-[illegible]
165.613
165.583
165.360
165.323
161.303
158.827
129.450
129.422
129.407
129.379
129.027
128.943
127.252
127.216
125.456
125.418
124.469
124.346
124.077
124.041
115.702
115.483
97.963
95.928
77.317
77.000
76.682
62.665
62.541
43.099
42.895
14.588
14.546
14.002
13.966
F
CO_2Et
CO_2Et
F
6j
f1 (ppm)

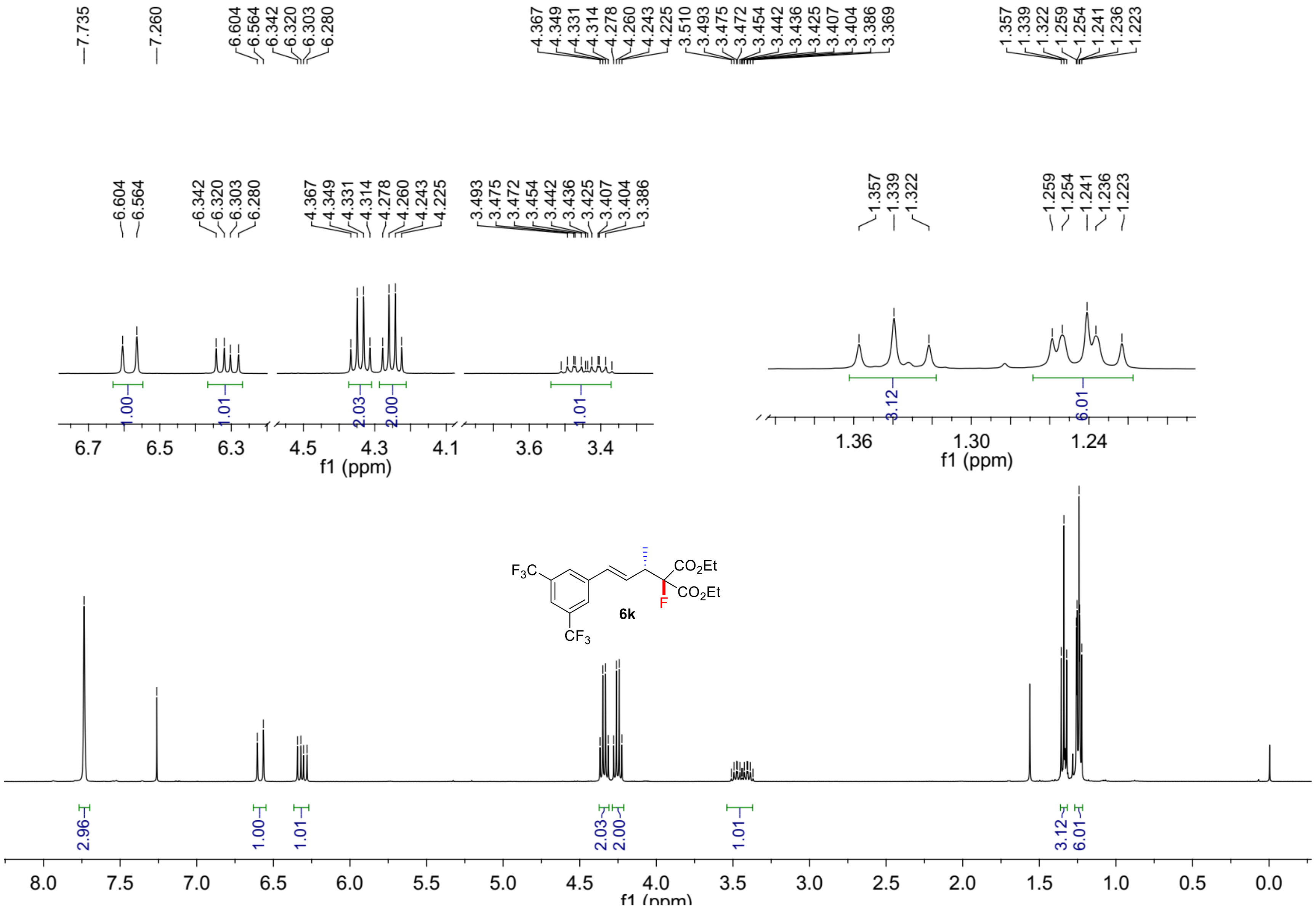

F3C
CF3
CO2Et
CO2Et
F
6k
f1 (ppm)

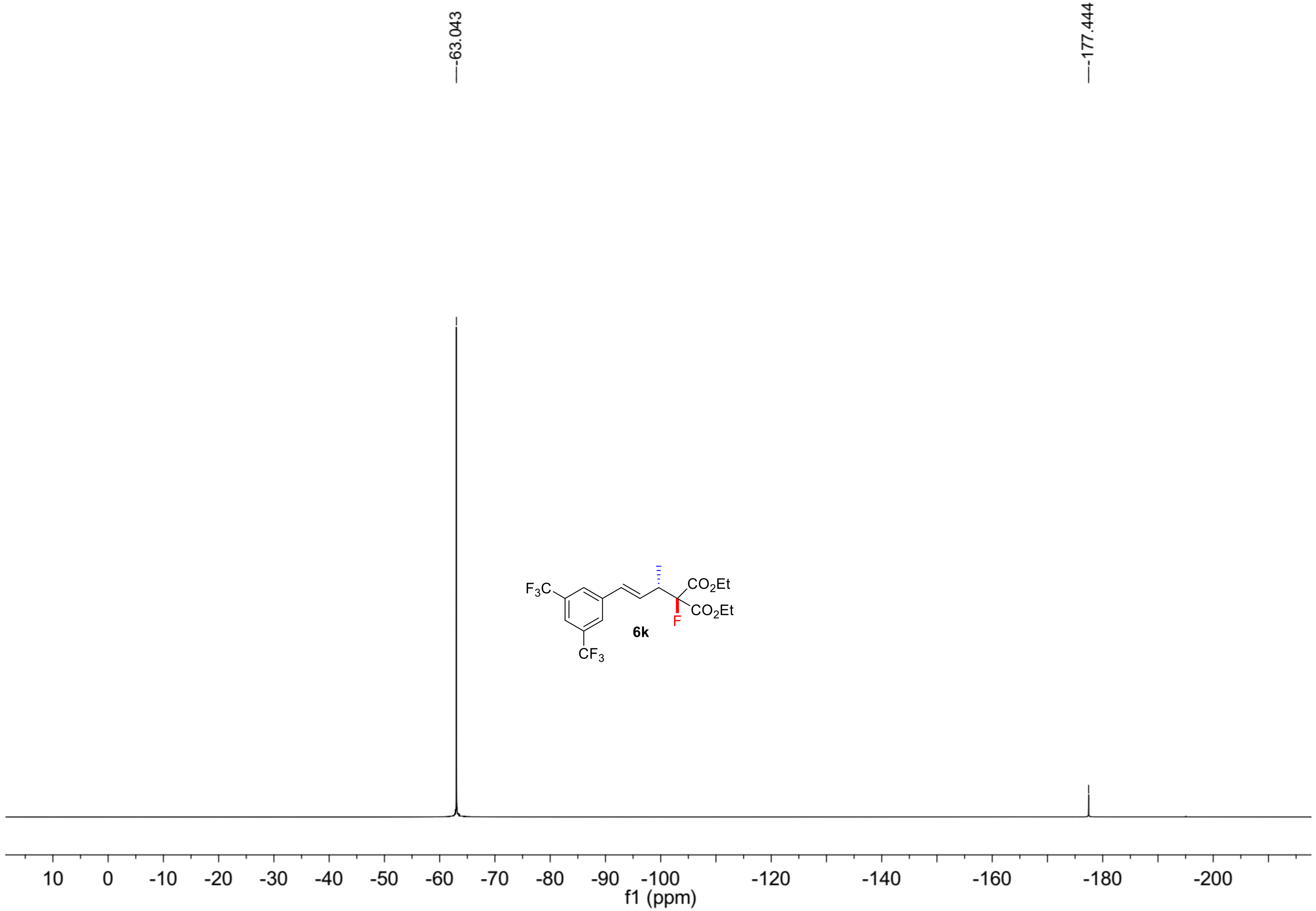
-63.043
-177.444
F3C
CO2Et
CO2Et
F
6k
CF3
10
0
-10
-20
-30
-40
-50
-60
-70
-80
-90
-100
-120
-140
-160
-180
-200
f1 (ppm)

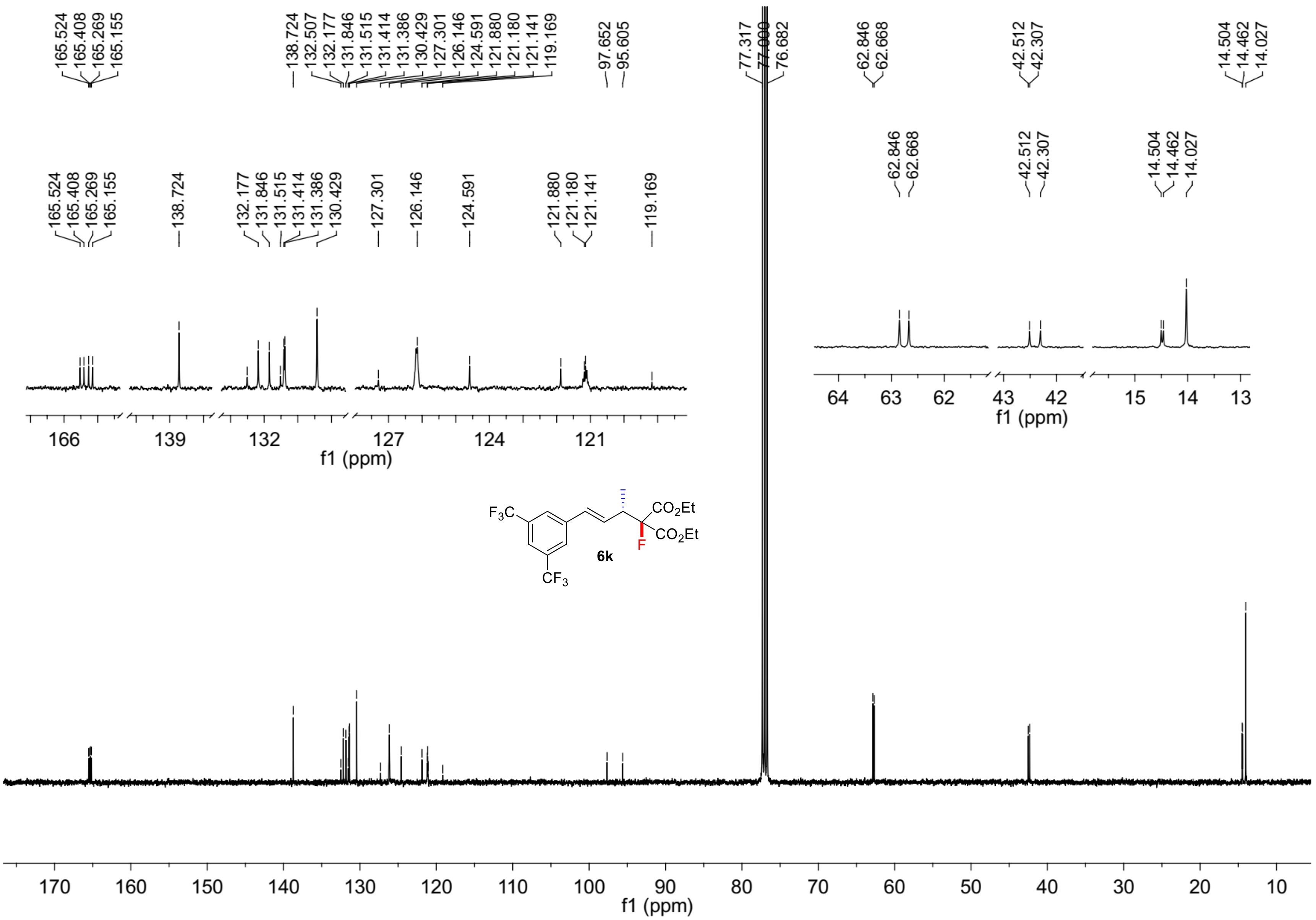

165.524
165.408
165.269
165.155
138.724
132.507
132.177
131.846
131.515
131.414
131.386
130.429
127.301
126.146
124.591
121.880
121.180
121.141
119.169
97.652
95.605
77.317
77.000
76.682
62.846
62.668
42.512
42.307
14.504
14.462
14.027
F3C
CF3
CO2Et
CO2Et
F
6k
f1 (ppm)

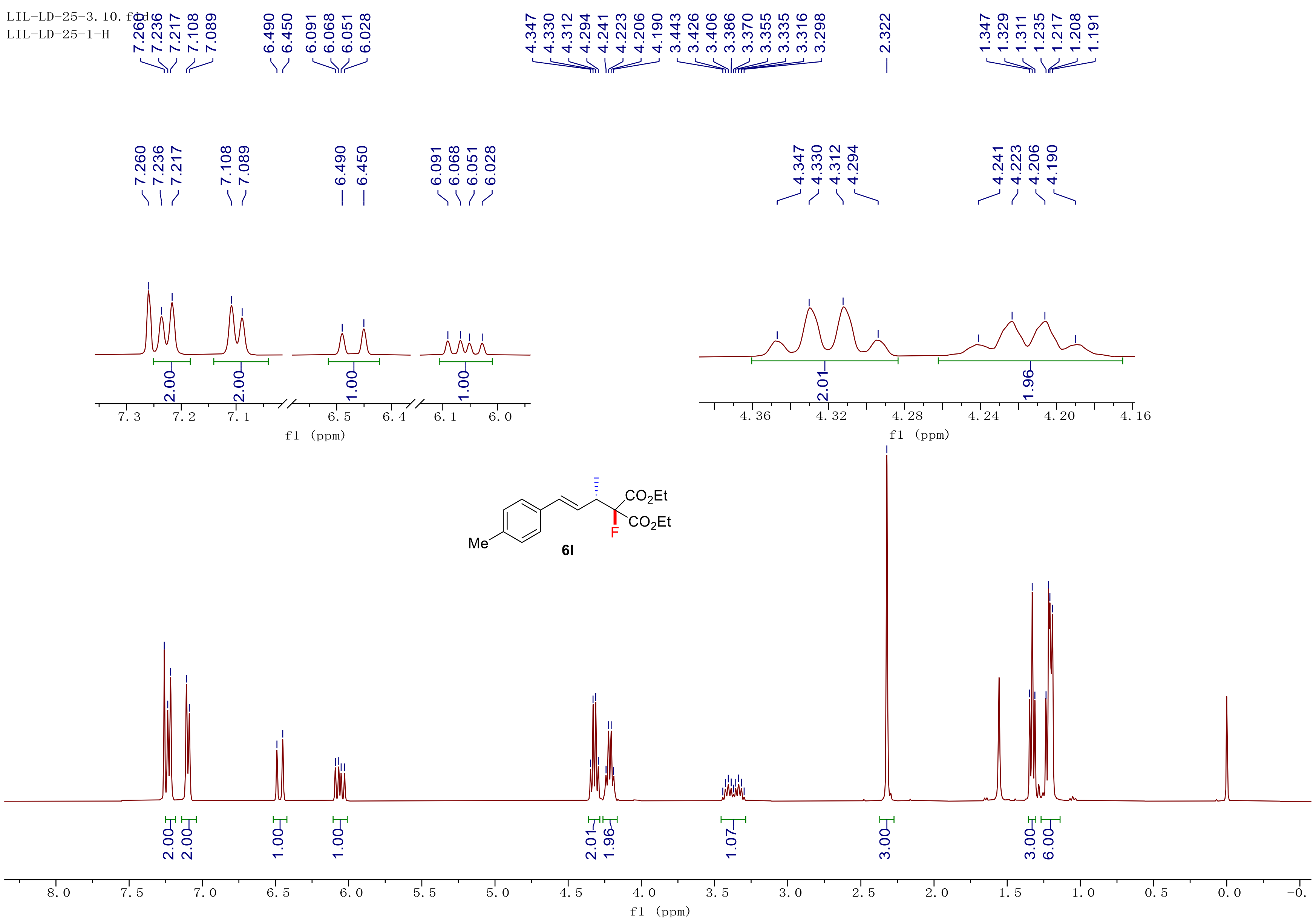
LIL-LD-25-3.10.fid
LIL-LD-25-1-H
7.260 7.236 7.217 7.108 7.089 6.490 6.450 6.091 6.068 6.051 6.028 4.347 4.330 4.312 4.294 4.241 4.223 4.206 4.190 3.443 3.426 3.406 3.386 3.370 3.355 3.335 3.316 3.298 2.322 1.347 1.329 1.311 1.235 1.217 1.208 1.191
2.00 2.00 1.00 1.00 2.01 1.96
f1 (ppm)
CO2Et
CO2Et
F
Me
6l
2.00 2.00 1.00 1.00 2.01 1.96 1.07 3.00 3.00 6.00
f1 (ppm)

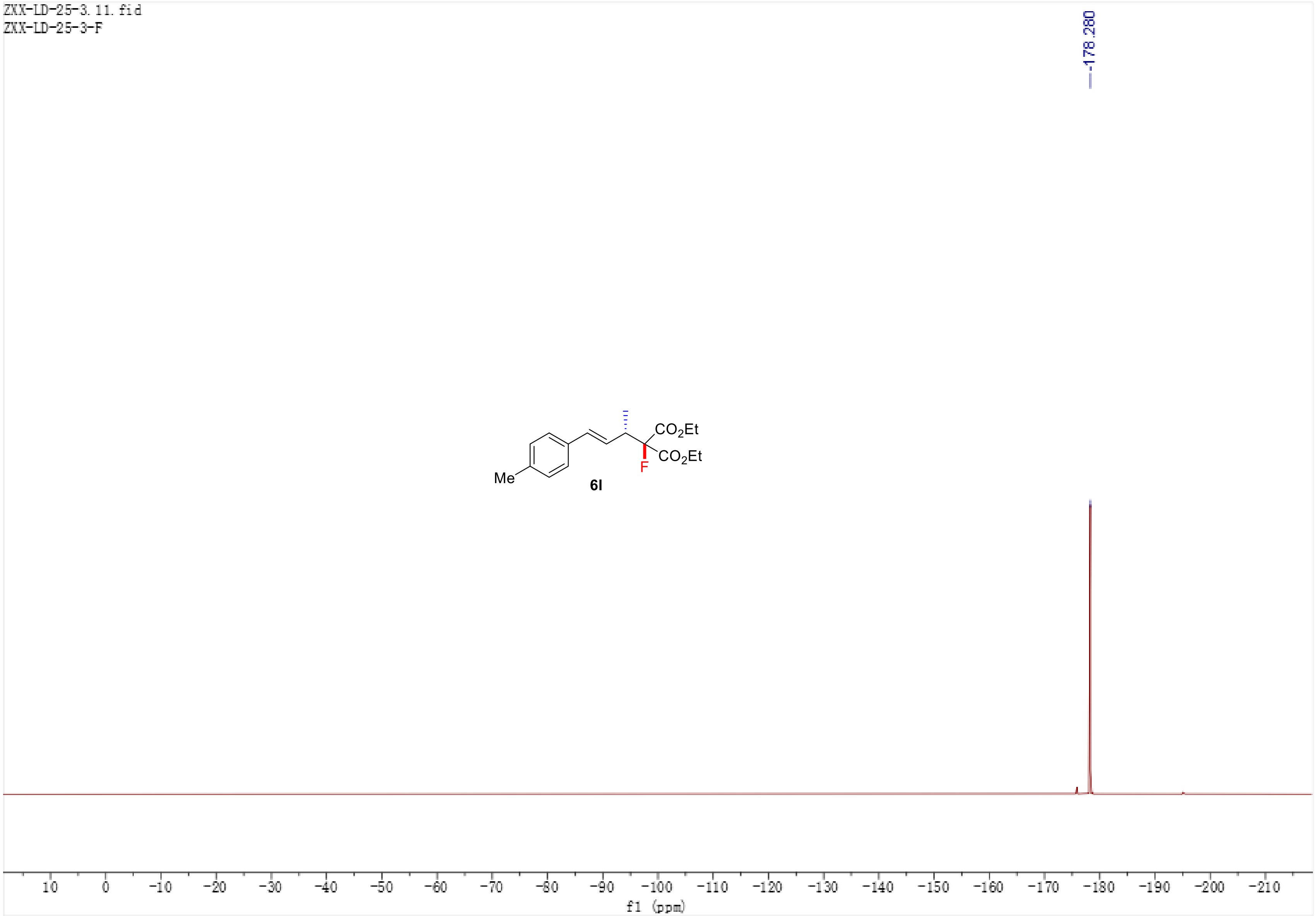
ZXX-LD-25-3.11.fid
ZXX-LD-25-3-F
-178.280
CO2Et
CO2Et
F
Me
6l
10
0
-10
-20
-30
-40
-50
-60
-70
-80
-90
-100
-110
-120
-130
-140
-150
-160
-170
-180
-190
-200
-210
f1 (ppm)

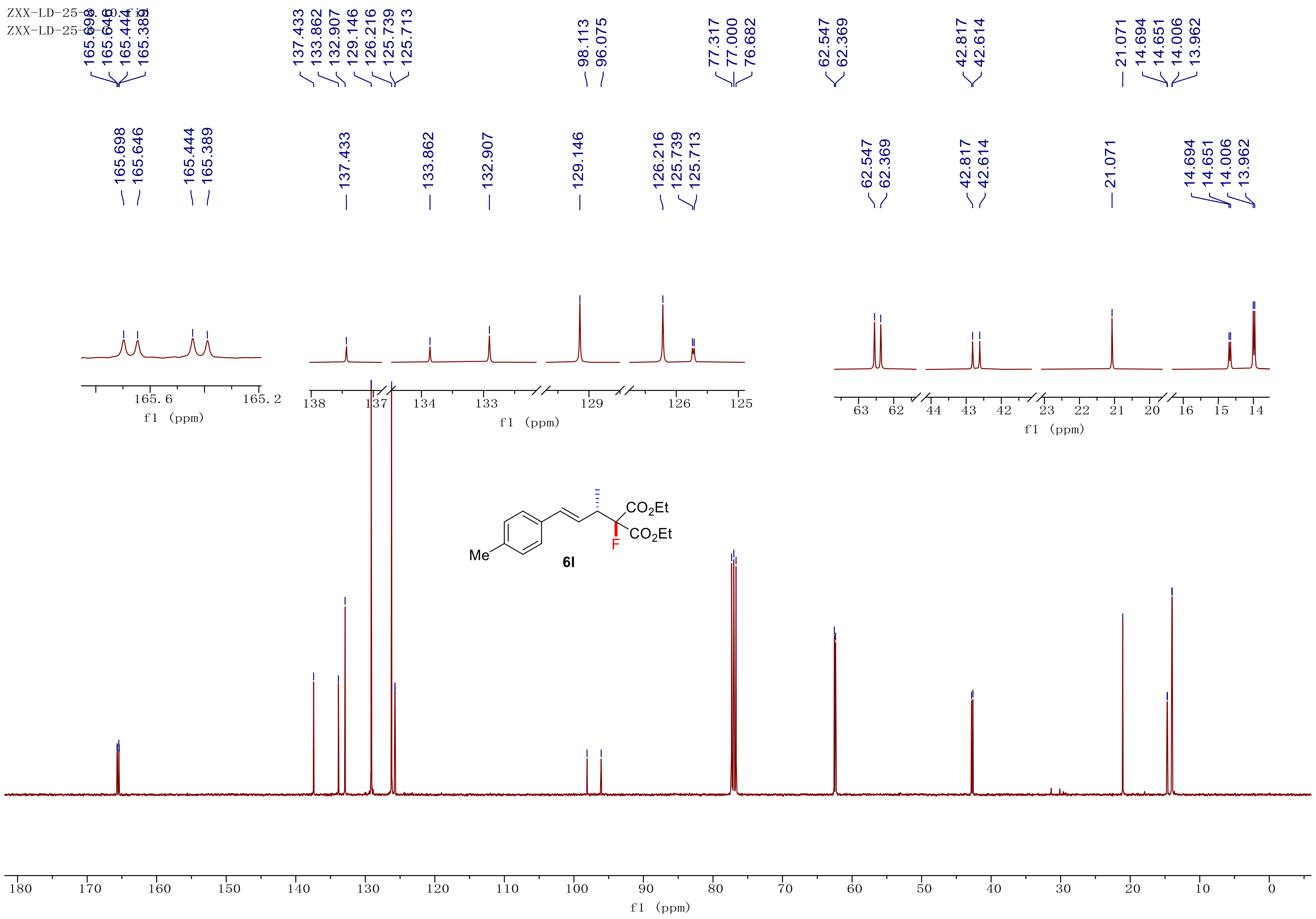
ZXX-LD-25-6-C.10.fid
165.698
165.646
165.444
165.389
137.433
133.862
132.907
129.146
126.216
125.739
125.713
98.113
96.075
77.317
77.000
76.682
62.547
62.369
42.817
42.614
21.071
14.694
14.651
14.006
13.962
CO2Et
CO2Et
F
Me
6l
f1 (ppm)

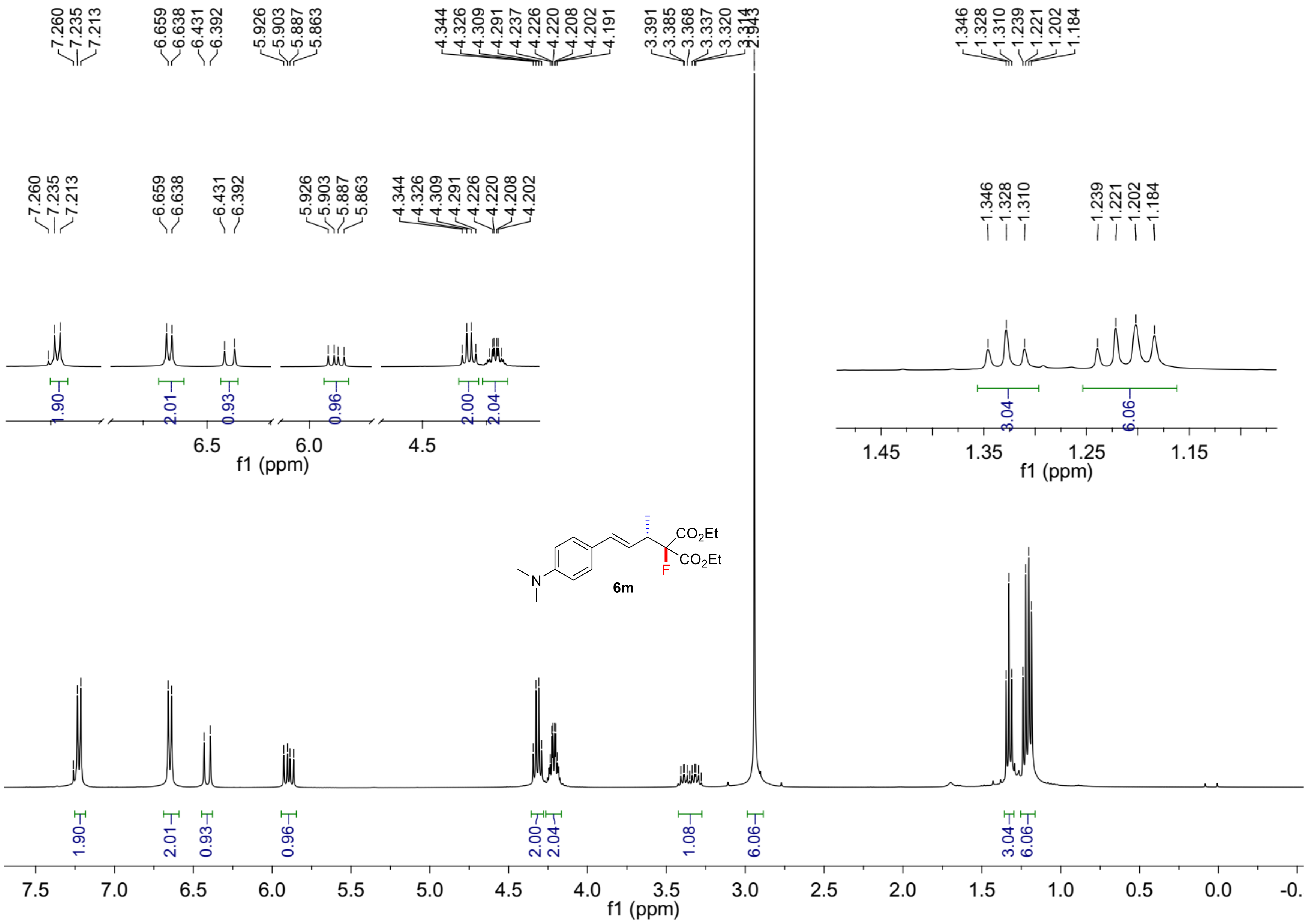
6m
CO2Et
CO2Et
F
N
f1 (ppm)

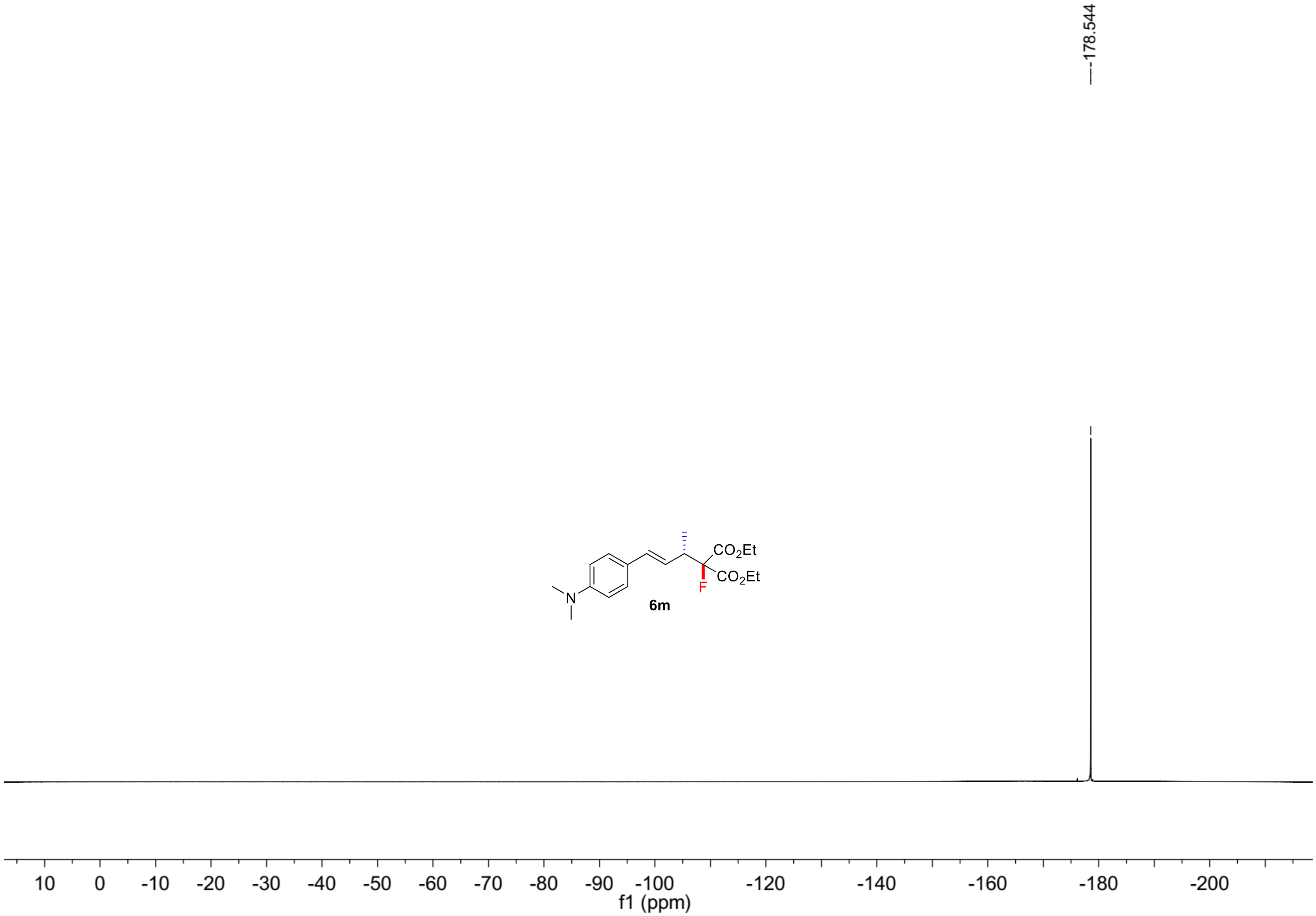

-178.544
CO2Et
CO2Et
F
N
6m
10 0 -10 -20 -30 -40 -50 -60 -70 -80 -90 -100 -120 -140 -160 -180 -200
f1 (ppm)

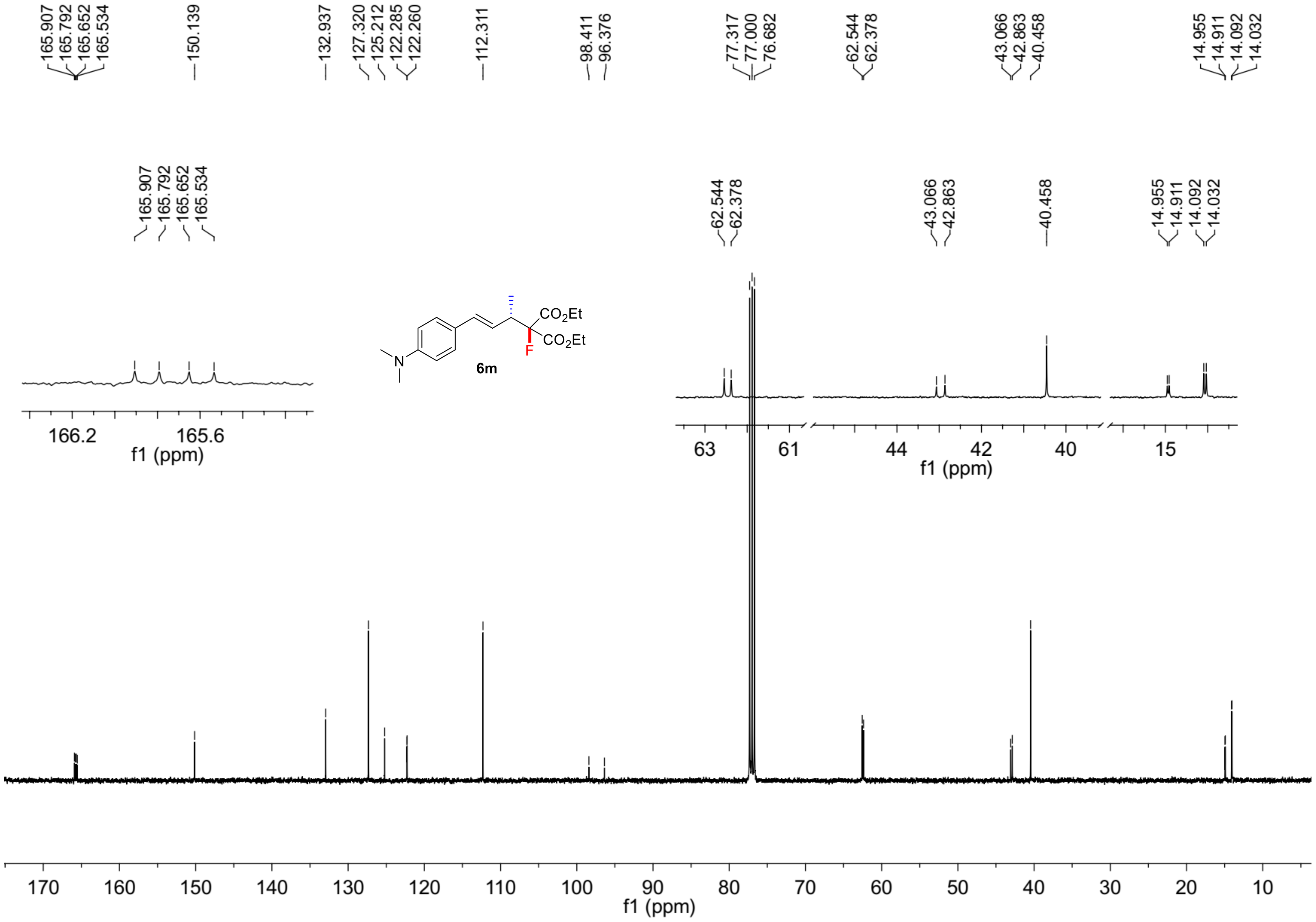
165.907
165.792
165.652
165.534
150.139
132.937
127.320
125.212
122.285
122.260
112.311
98.411
96.376
77.317
77.000
76.682
62.544
62.378
43.066
42.863
40.458
14.955
14.911
14.092
14.032
CO_2Et
CO_2Et
F
N
6m
166.2
165.6
f1 (ppm)
63
61
44
42
40
15
f1 (ppm)
170
160
150
140
130
120
110
100
90
80
70
60
50
40
30
20
10
f1 (ppm)

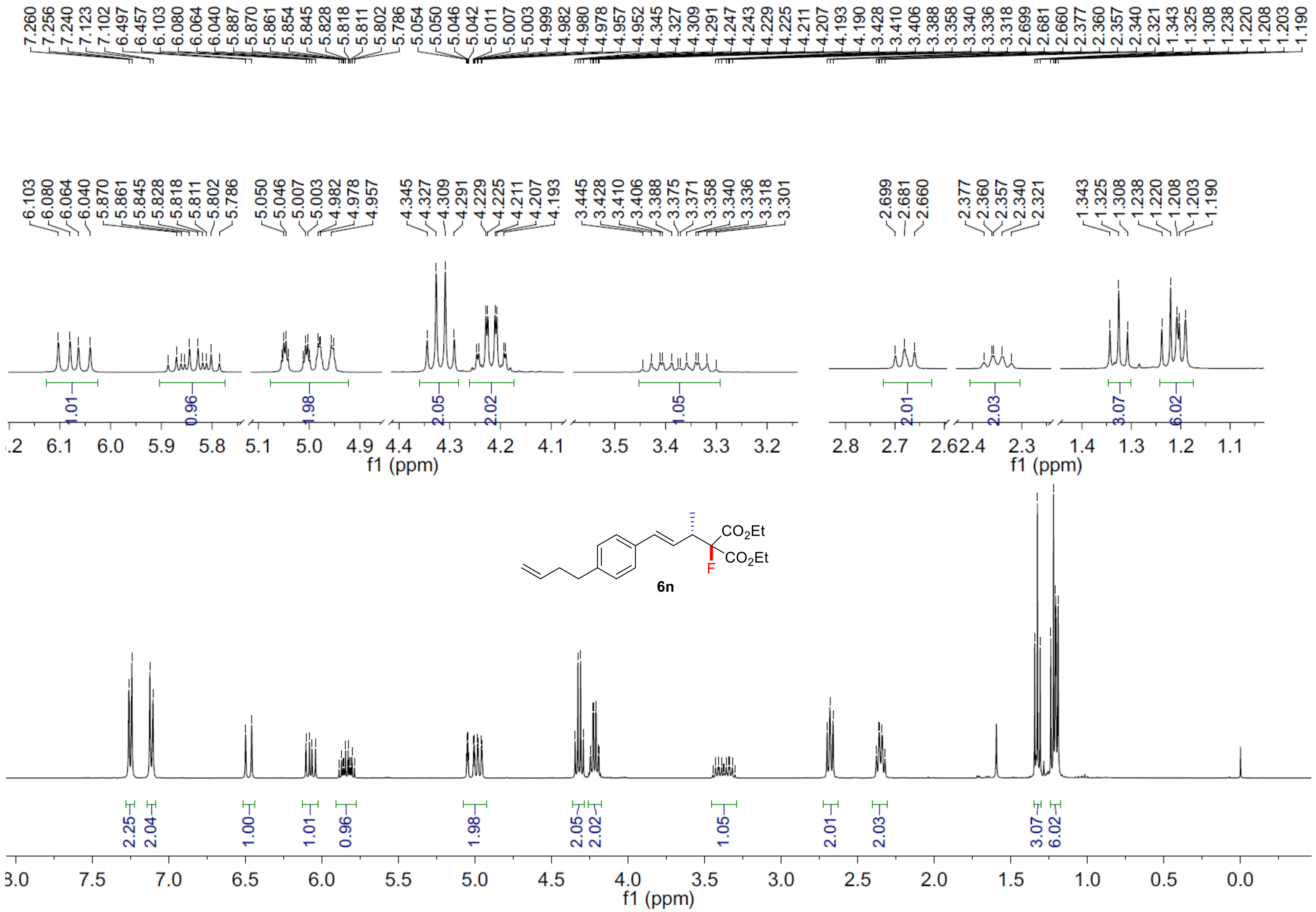
CO2Et
CO2Et
F
6n
f1 (ppm)

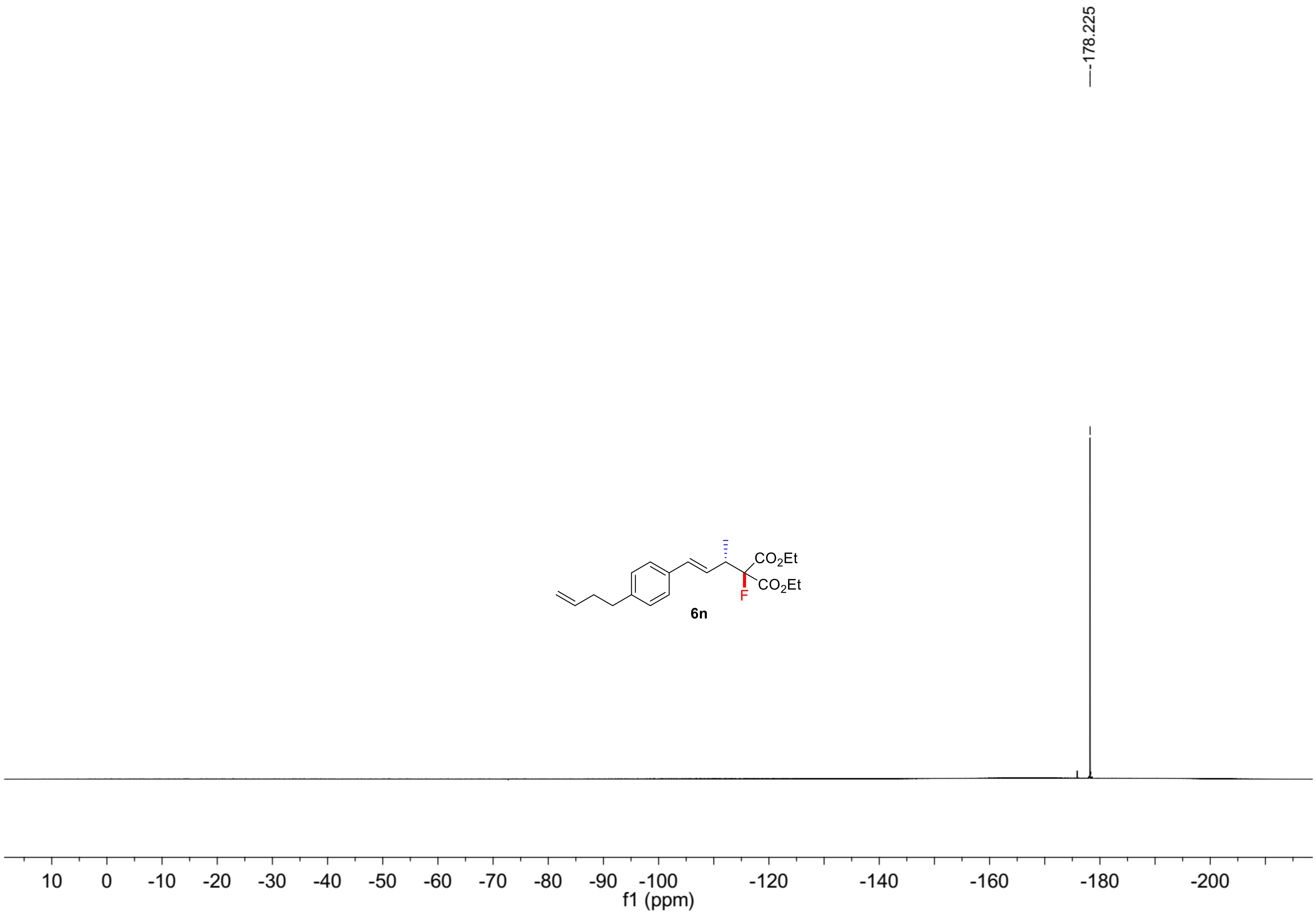

-178.225
CO2Et
CO2Et
F
6n
10 0 -10 -20 -30 -40 -50 -60 -70 -80 -90 -100 -120 -140 -160 -180 -200
f1 (ppm)

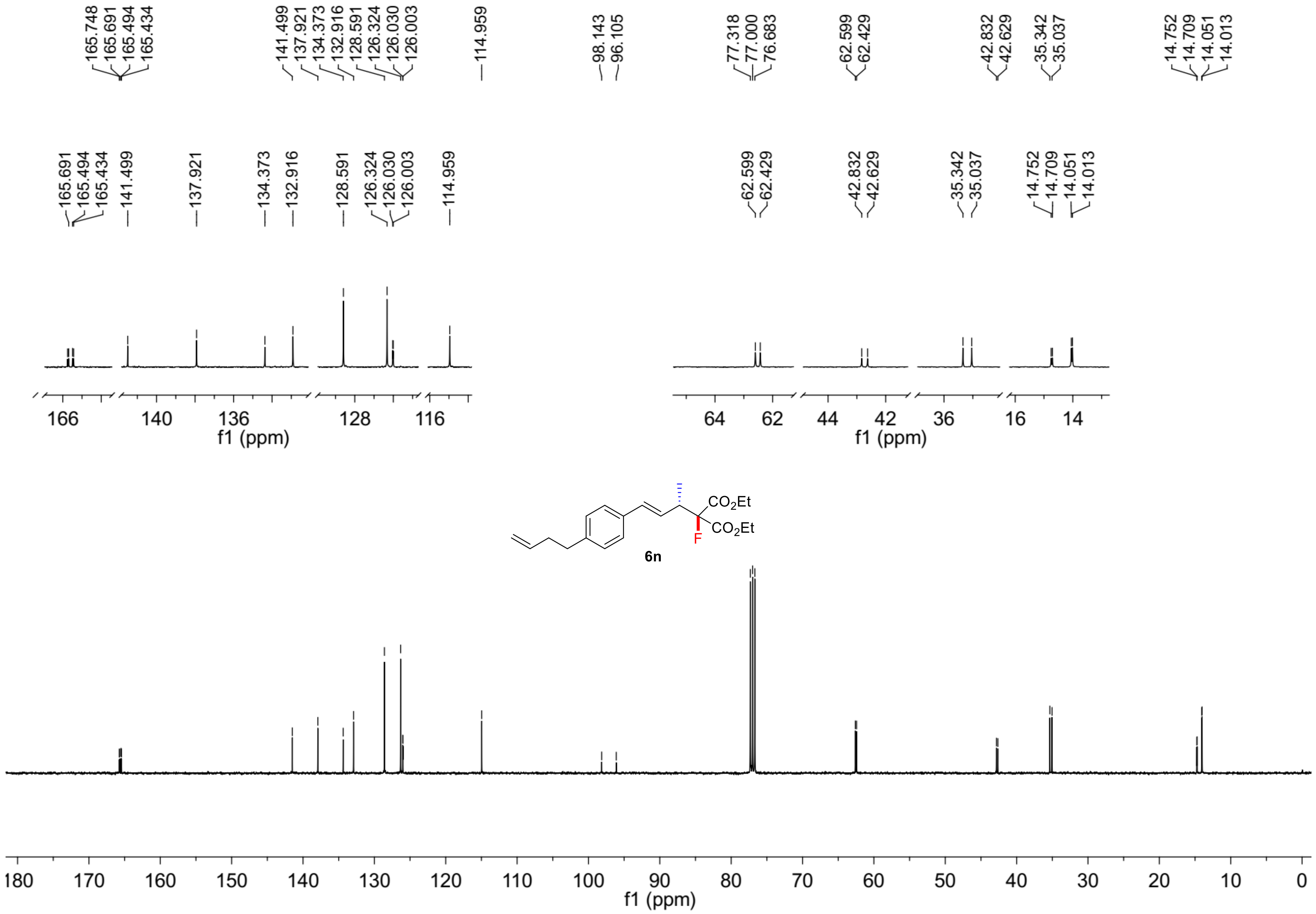
165.748
165.691
165.494
165.434
141.499
137.921
134.373
132.916
128.591
126.324
126.030
126.003
114.959
98.143
96.105
77.318
77.000
76.683
62.599
62.429
42.832
42.629
35.342
35.037
14.752
14.709
14.051
14.013
f1 (ppm)
CO_2Et
CO_2Et
F
6n

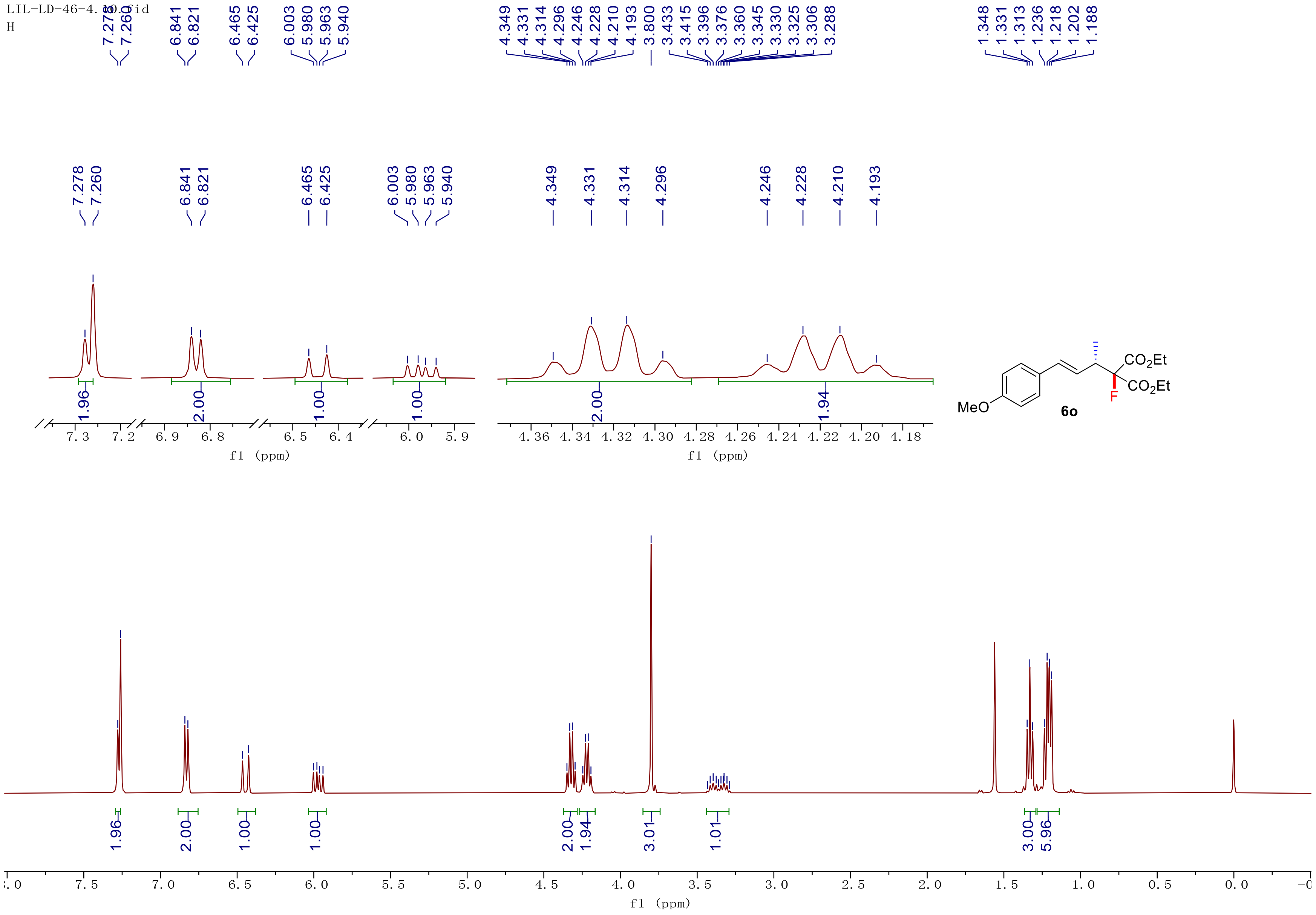
LIL-LD-46-4.10.fid
H
7.278 7.260 6.841 6.821 6.465 6.425 6.003 5.980 5.963 5.940
4.349 4.331 4.314 4.296 4.246 4.228 4.210 4.193 3.800 3.433 3.415 3.396 3.376 3.360 3.345 3.330 3.325 3.306 3.288
1.348 1.331 1.313 1.236 1.218 1.202 1.188
MeO
CO2Et
CO2Et
F
6o
1.96 2.00 1.00 1.00 2.00 1.94 3.01 1.01 3.00 5.96
f1 (ppm)

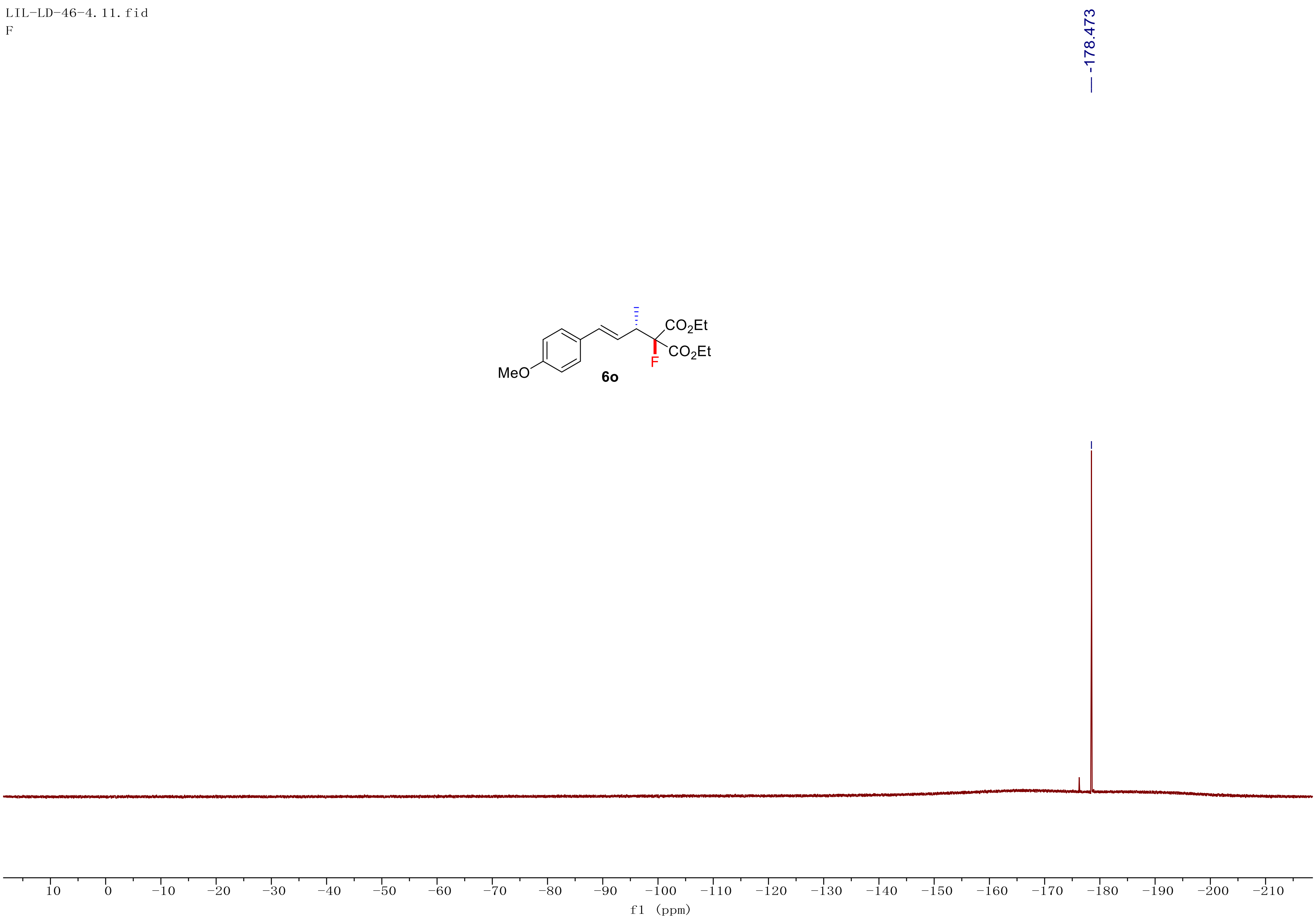
LIL-LD-46-4.11.fid
F
-178.473
MeO
CO2Et
CO2Et
F
6o
10 0 −10 −20 −30 −40 −50 −60 −70 −80 −90 −100 −110 −120 −130 −140 −150 −160 −170 −180 −190 −200 −210
f1 (ppm)

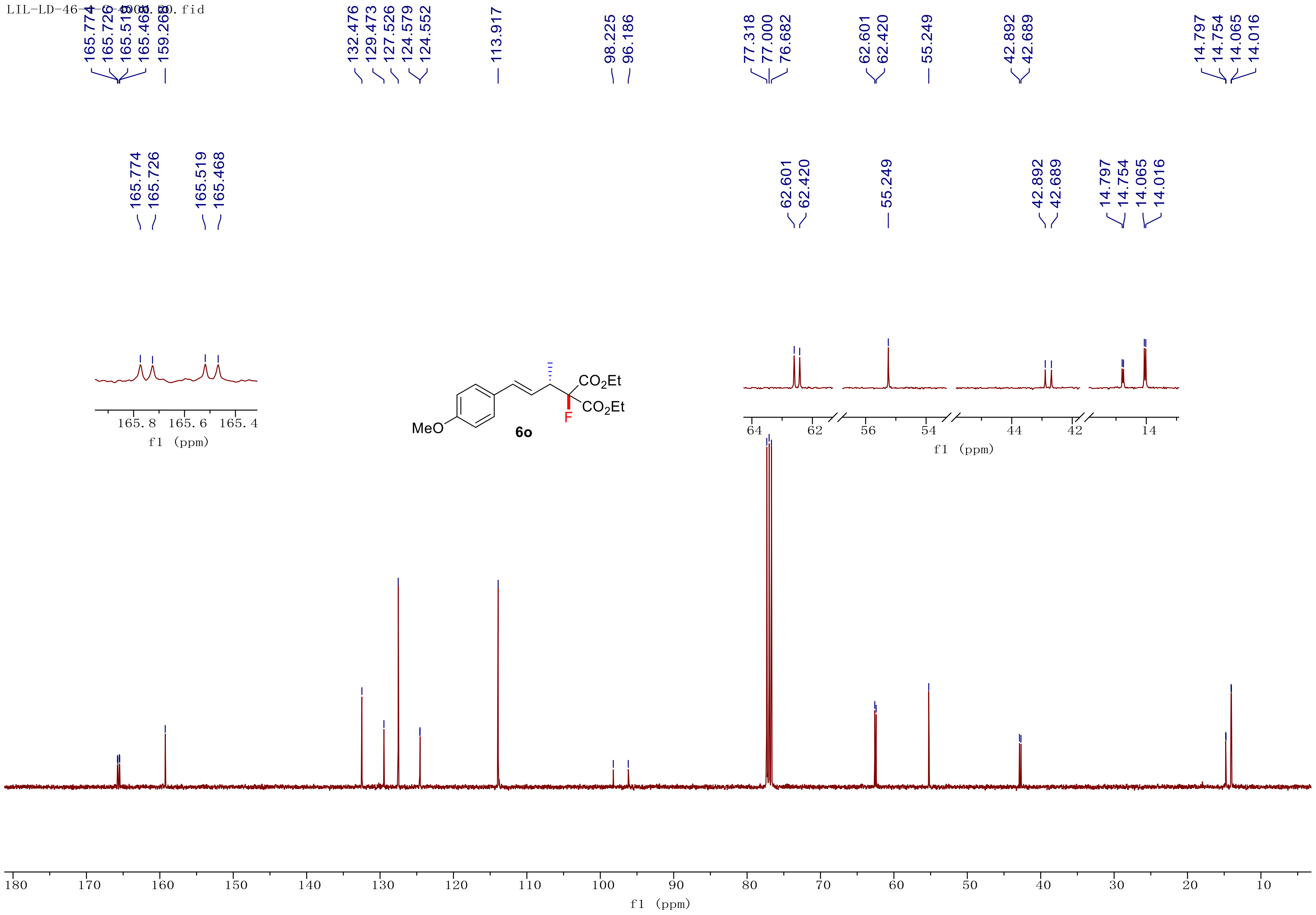
LIL-LD-46-4-C-400M.10.fid
165.774
165.726
165.519
165.468
159.268
132.476
129.473
127.526
124.579
124.552
113.917
98.225
96.186
77.318
77.000
76.682
62.601
62.420
55.249
42.892
42.689
14.797
14.754
14.065
14.016
MeO
CO2Et
CO2Et
F
6o
f1 (ppm)

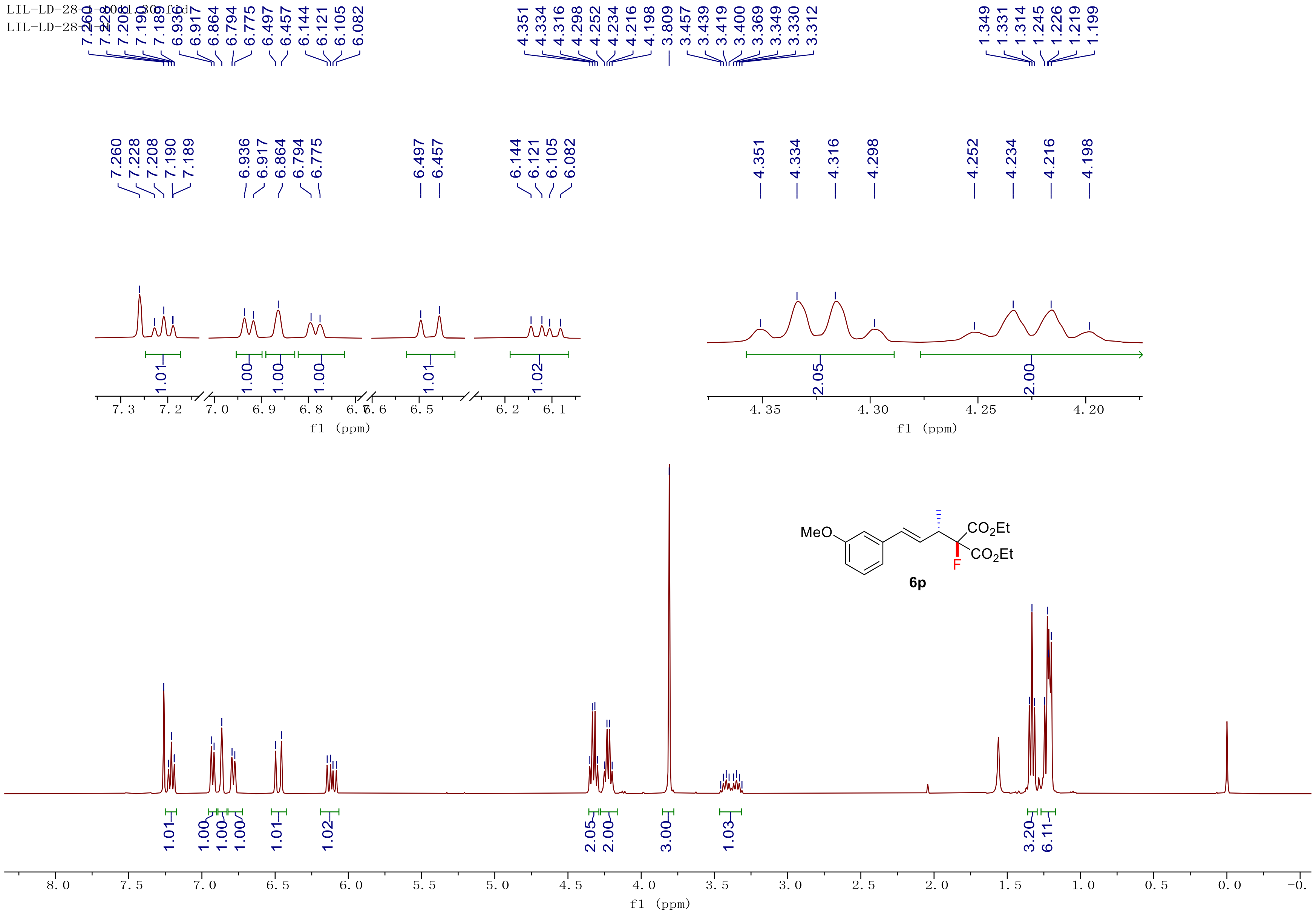
LIL-LD-28-1-1011.30.fid
LIL-LD-28-1-1
MeO
CO2Et
CO2Et
F
6p
f1 (ppm)

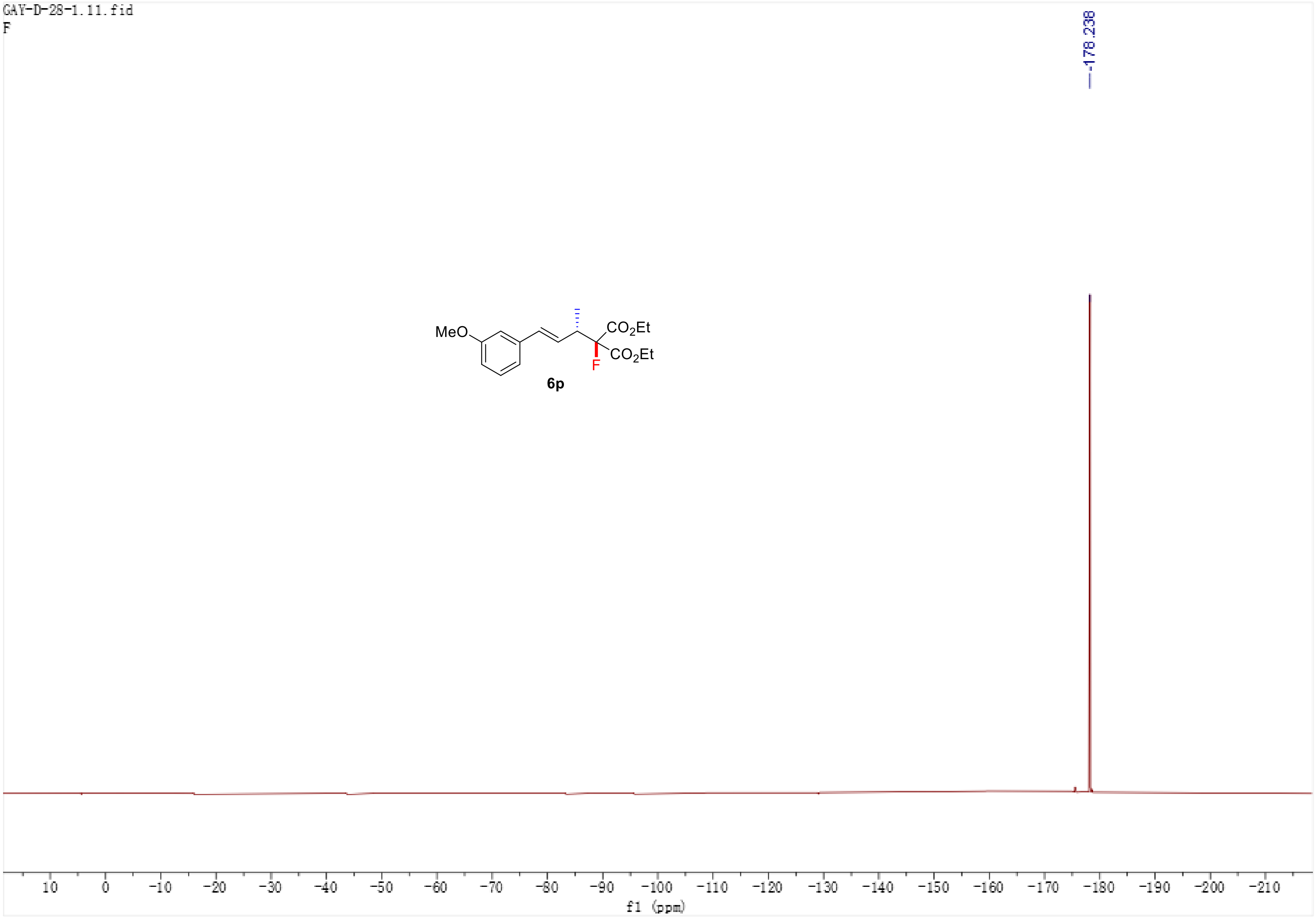
GAY-D-28-1.11.fid
F
-178.238
MeO
CO2Et
CO2Et
F
6p
10 0 -10 -20 -30 -40 -50 -60 -70 -80 -90 -100 -110 -120 -130 -140 -150 -160 -170 -180 -190 -200 -210
f1 (ppm)

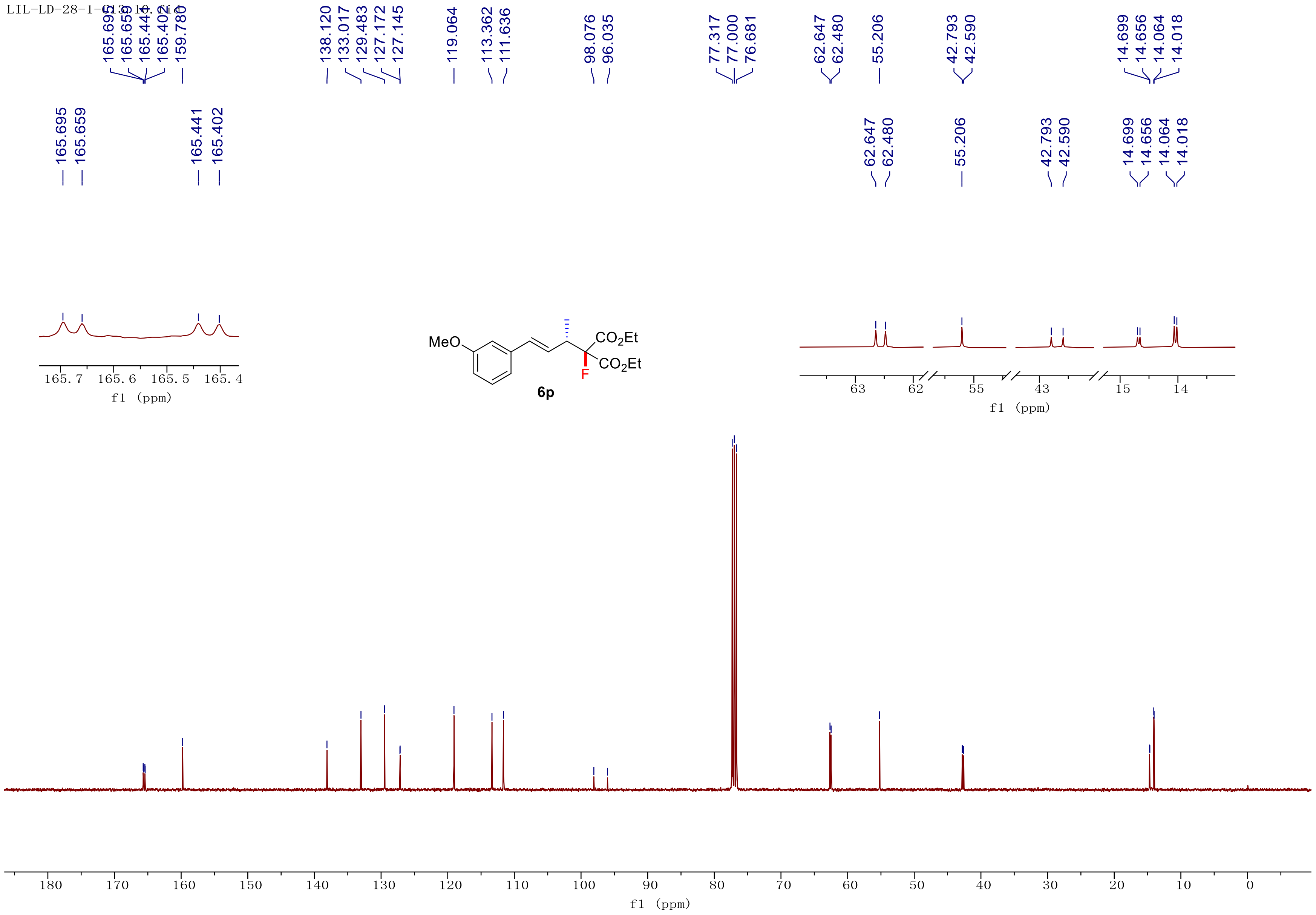
LIL-LD-28-1-C13-14.fid
165.695
165.659
165.441
165.402
159.780
138.120
133.017
129.483
127.172
127.145
119.064
113.362
111.636
98.076
96.035
77.317
77.000
76.681
62.647
62.480
55.206
42.793
42.590
14.699
14.656
14.064
14.018
MeO
CO2Et
CO2Et
F
6p
f1 (ppm)

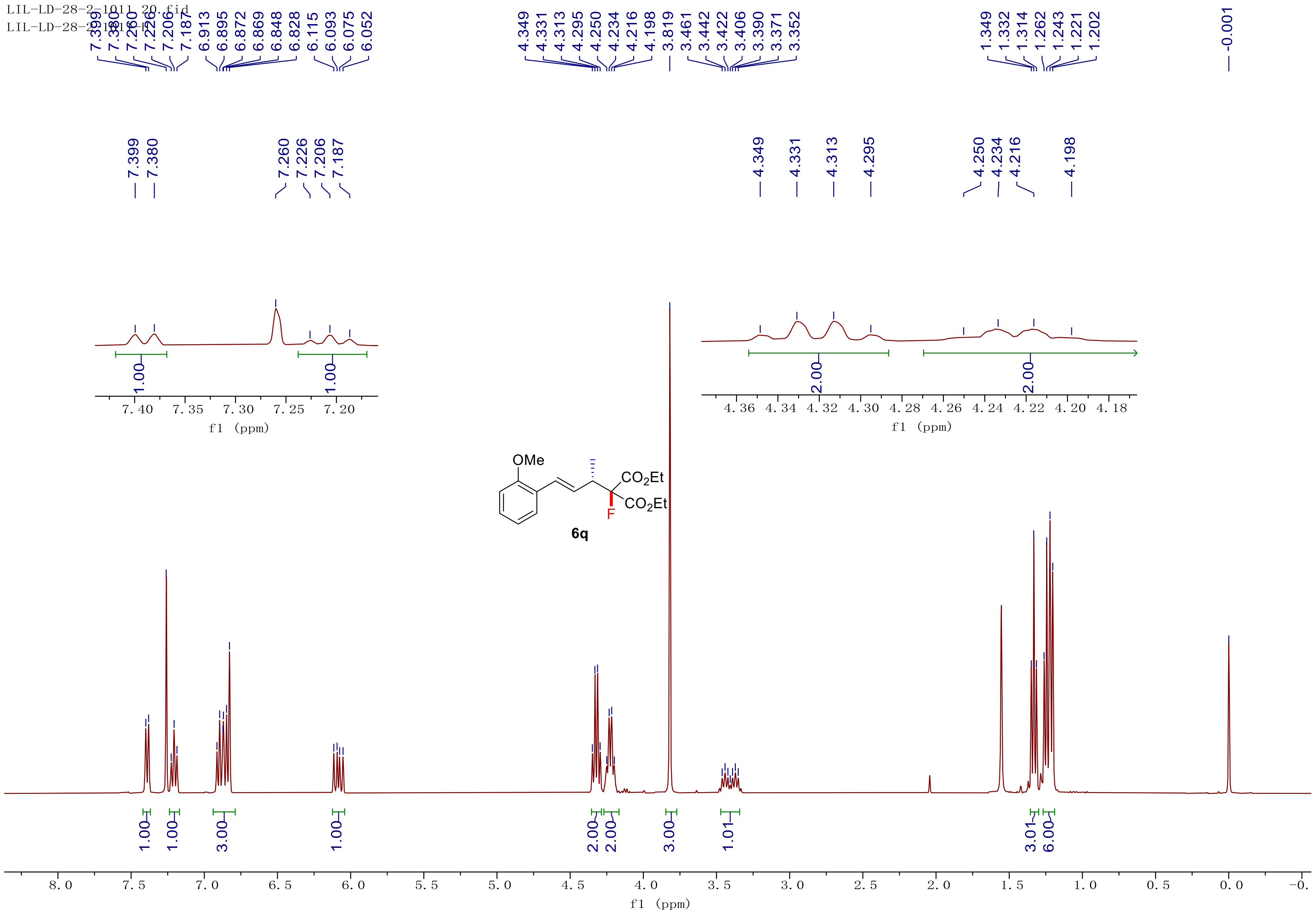
LIL-LD-28-2-1011_20.fid
OMe
CO2Et
CO2Et
F
6q
f1 (ppm)

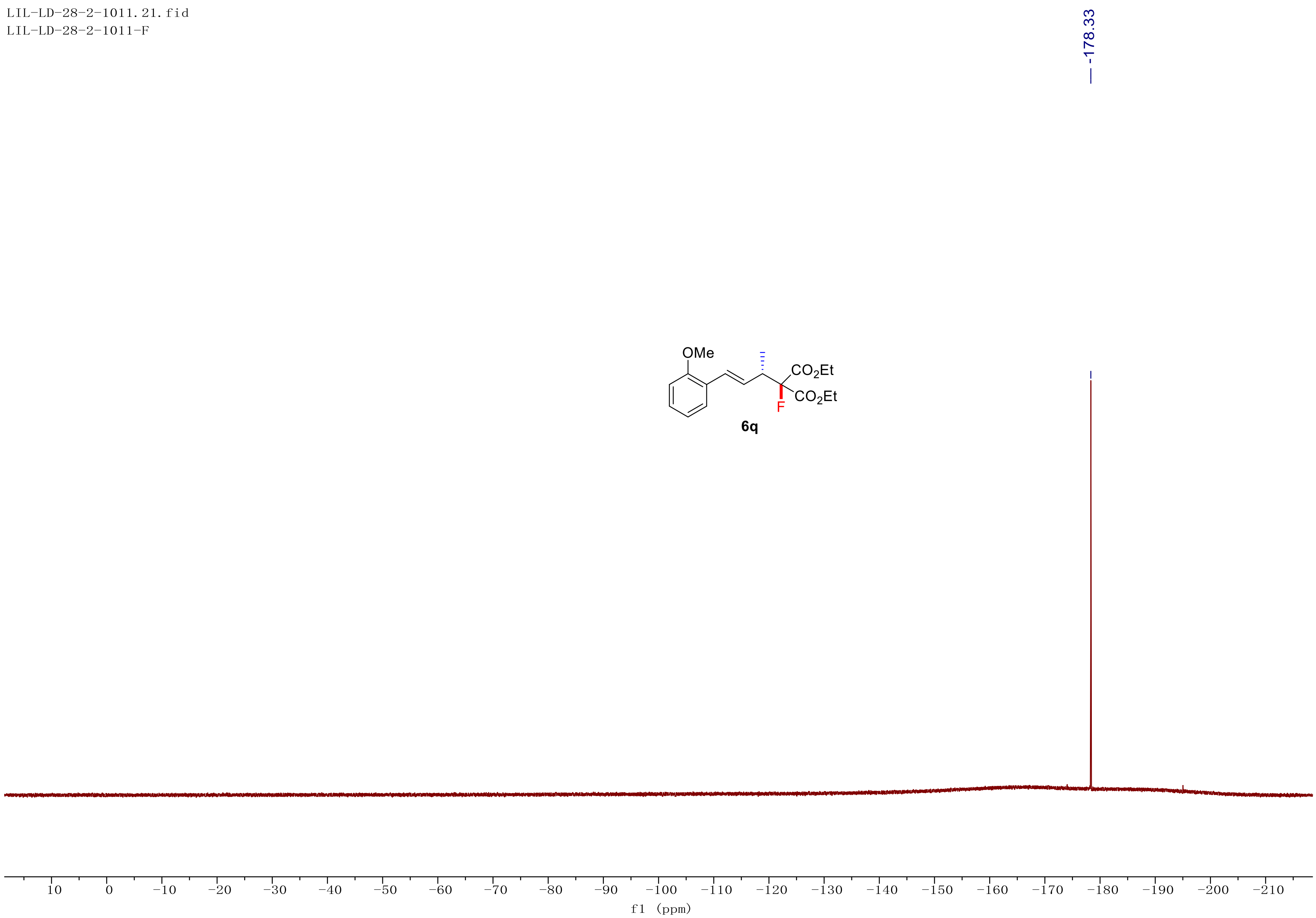
LIL-LD-28-2-1011.21.fid
LIL-LD-28-2-1011-F
-178.33
OMe
CO_2Et
CO_2Et
F
6q
10 0 −10 −20 −30 −40 −50 −60 −70 −80 −90 −100 −110 −120 −130 −140 −150 −160 −170 −180 −190 −200 −210
f1 (ppm)

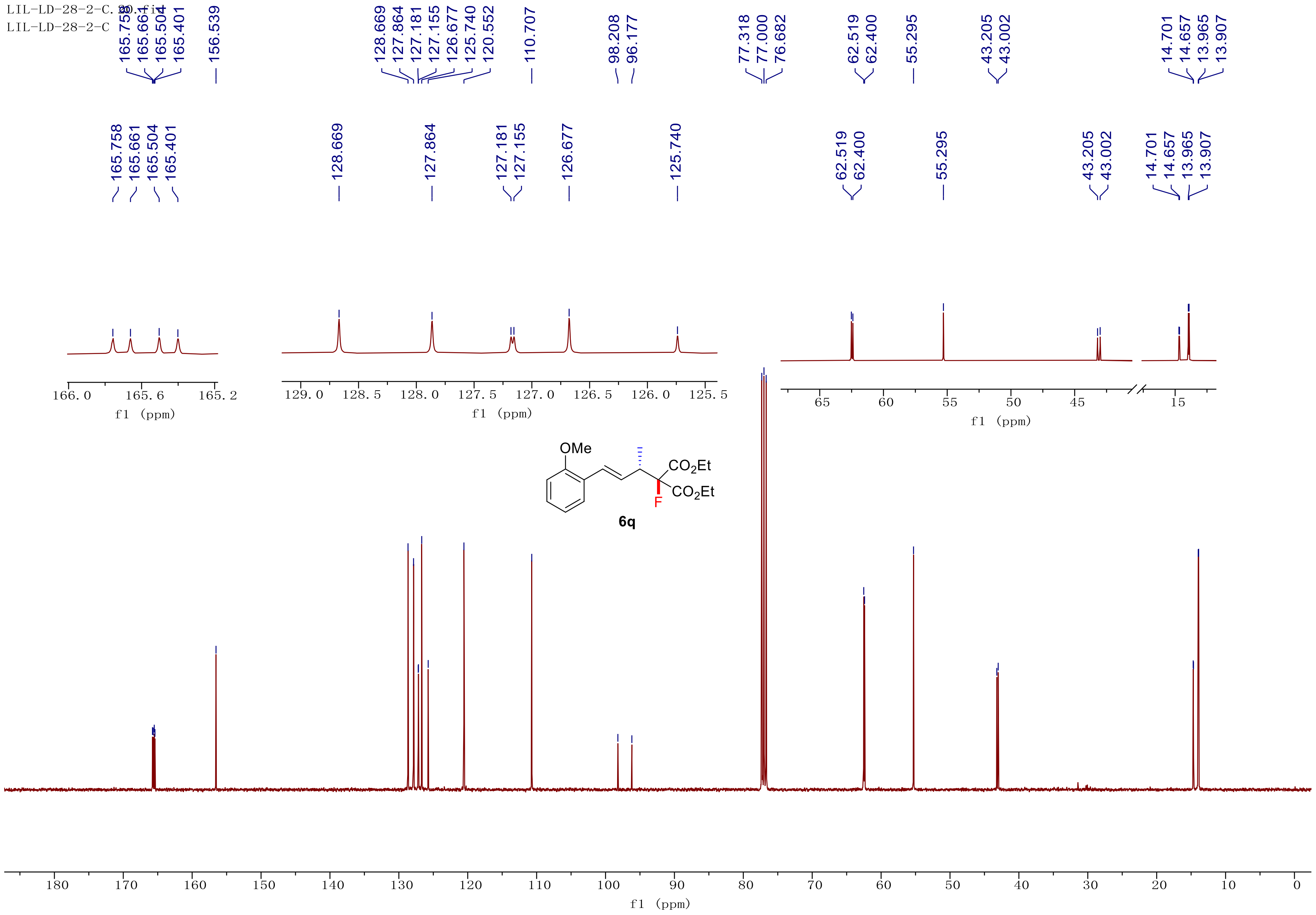
LIL-LD-28-2-C.10.fid
LIL-LD-28-2-C
165.758
165.661
165.504
165.401
156.539
128.669
127.864
127.181
127.155
126.677
125.740
120.552
110.707
98.208
96.177
77.318
77.000
76.682
62.519
62.400
55.295
43.205
43.002
14.701
14.657
13.965
13.907
OMe
CO2Et
CO2Et
F
6q
f1 (ppm)

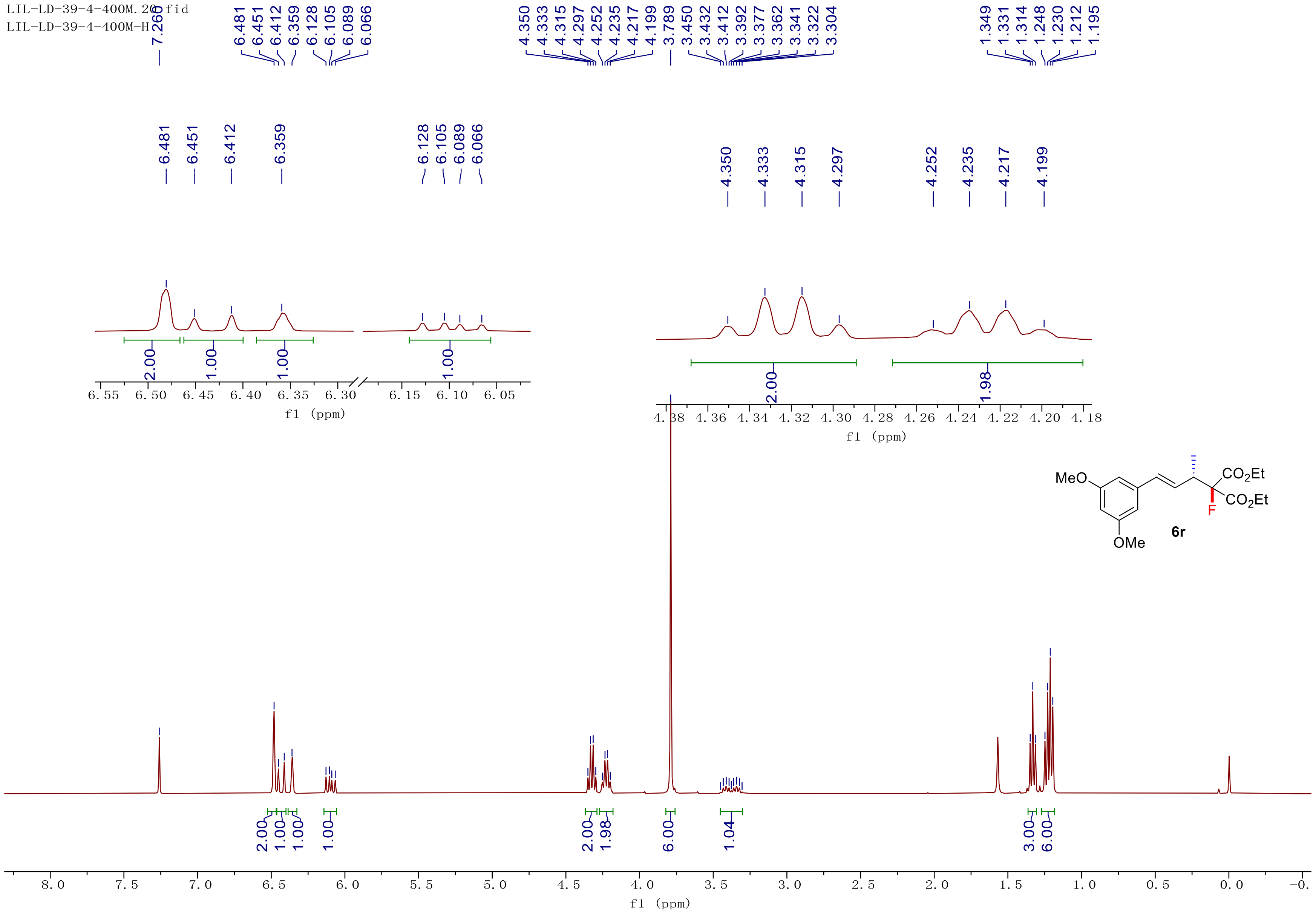
LIL-LD-39-4-400M.20.fid
LIL-LD-39-4-400M-H
7.260
6.481 6.451 6.412 6.359 6.128 6.105 6.089 6.066
4.350 4.333 4.315 4.297 4.252 4.235 4.217 4.199 3.789 3.450 3.432 3.412 3.392 3.377 3.362 3.341 3.322 3.304
1.349 1.331 1.314 1.248 1.230 1.212 1.195
2.00 1.00 1.00 1.00
2.00 1.98
2.00 1.00 1.00 1.00 2.00 1.98 6.00 1.04 3.00 6.00
f1 (ppm)
MeO
OMe
CO2Et
CO2Et
F
6r

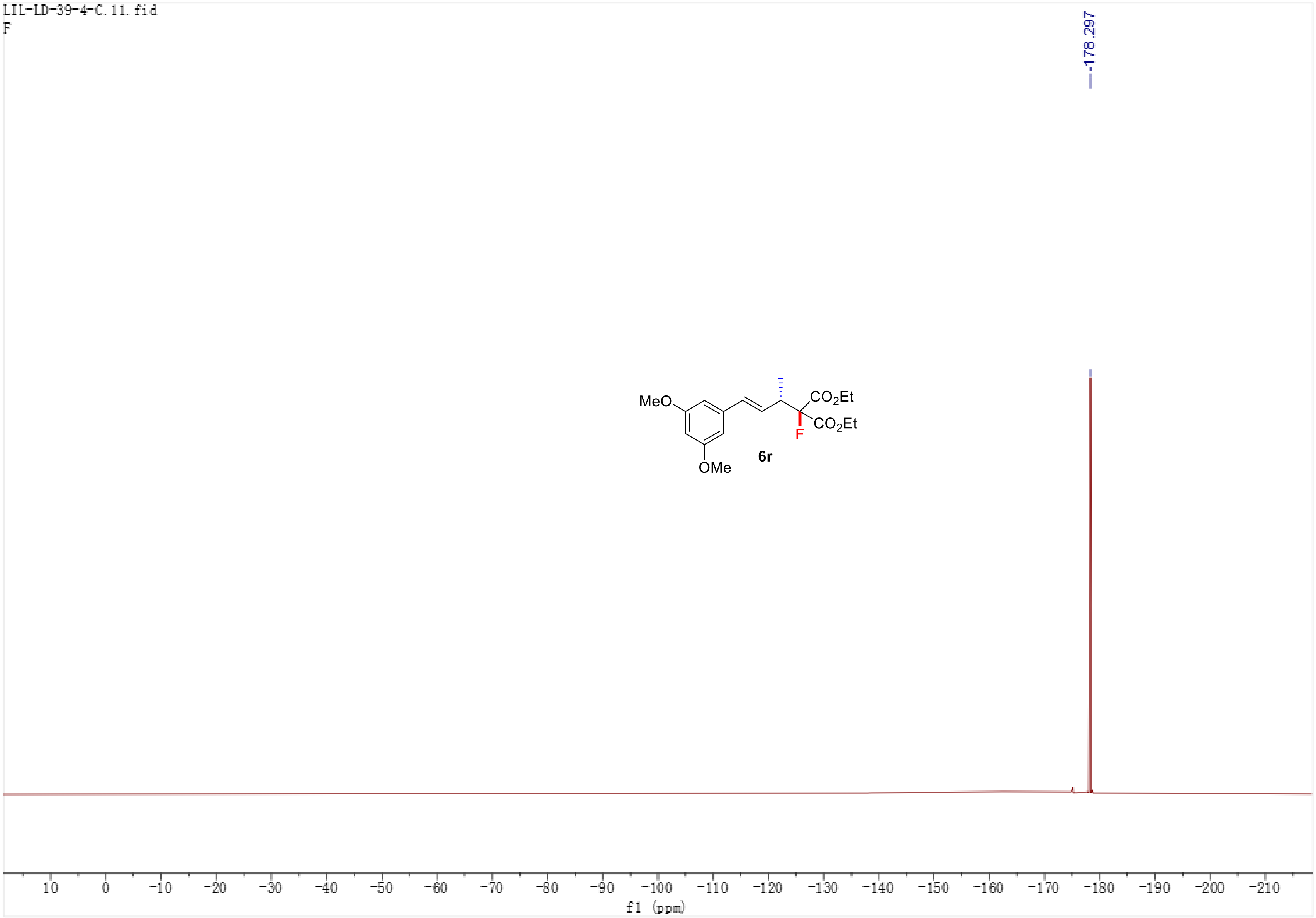
LIL-LD-39-4-C.11.fid
F
-178.297
MeO
OMe
CO_2Et
CO_2Et
F
6r
10
0
-10
-20
-30
-40
-50
-60
-70
-80
-90
-100
-110
-120
-130
-140
-150
-160
-170
-180
-190
-200
-210
f1 (ppm)

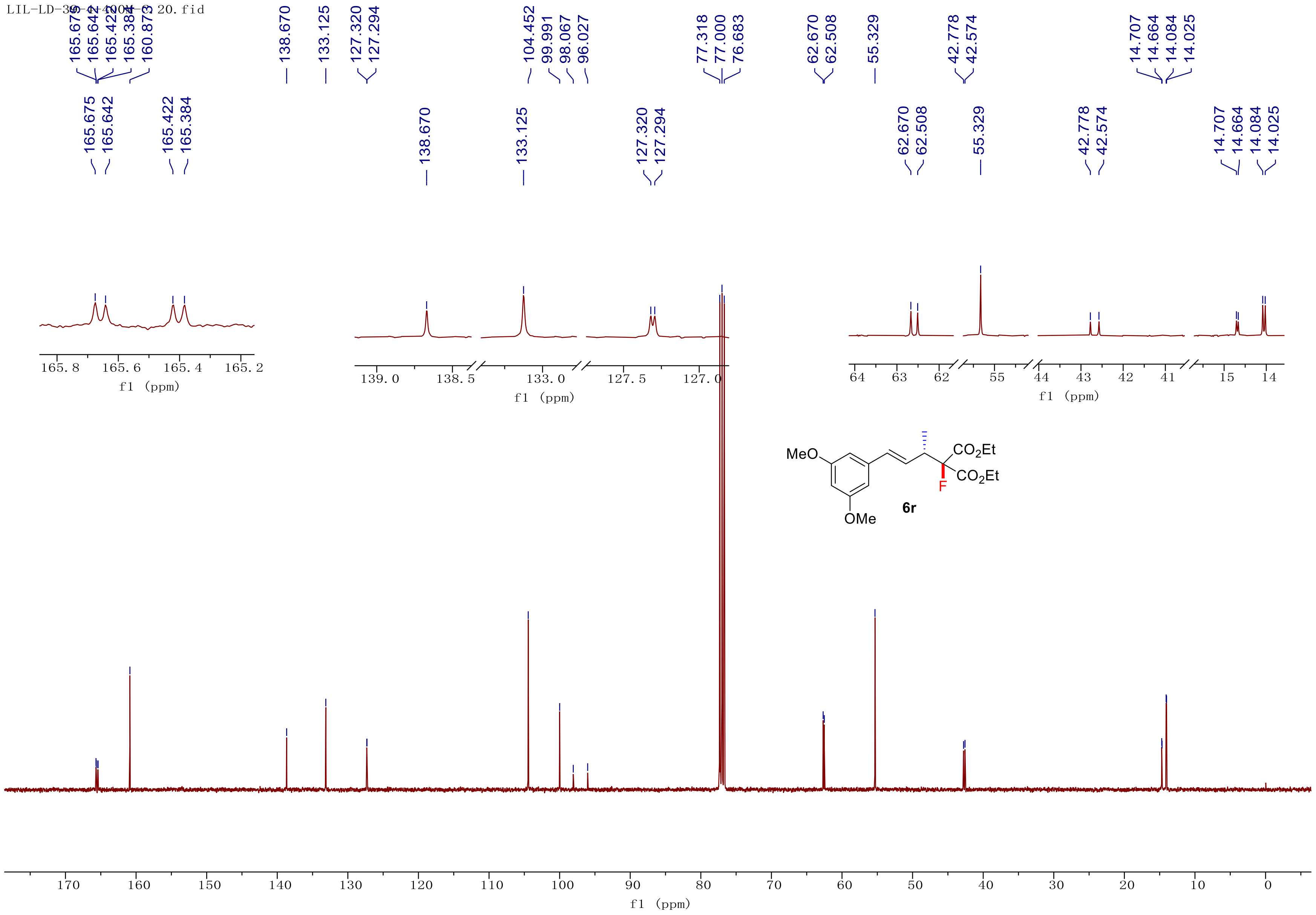

LIL-LD-39-4-400M-C 20.fid
165.675
165.642
165.422
165.384
160.873
138.670
133.125
127.320
127.294
104.452
99.991
98.067
96.027
77.318
77.000
76.683
62.670
62.508
55.329
42.778
42.574
14.707
14.664
14.084
14.025
f1 (ppm)
MeO
OMe
CO2Et
CO2Et
F
6r

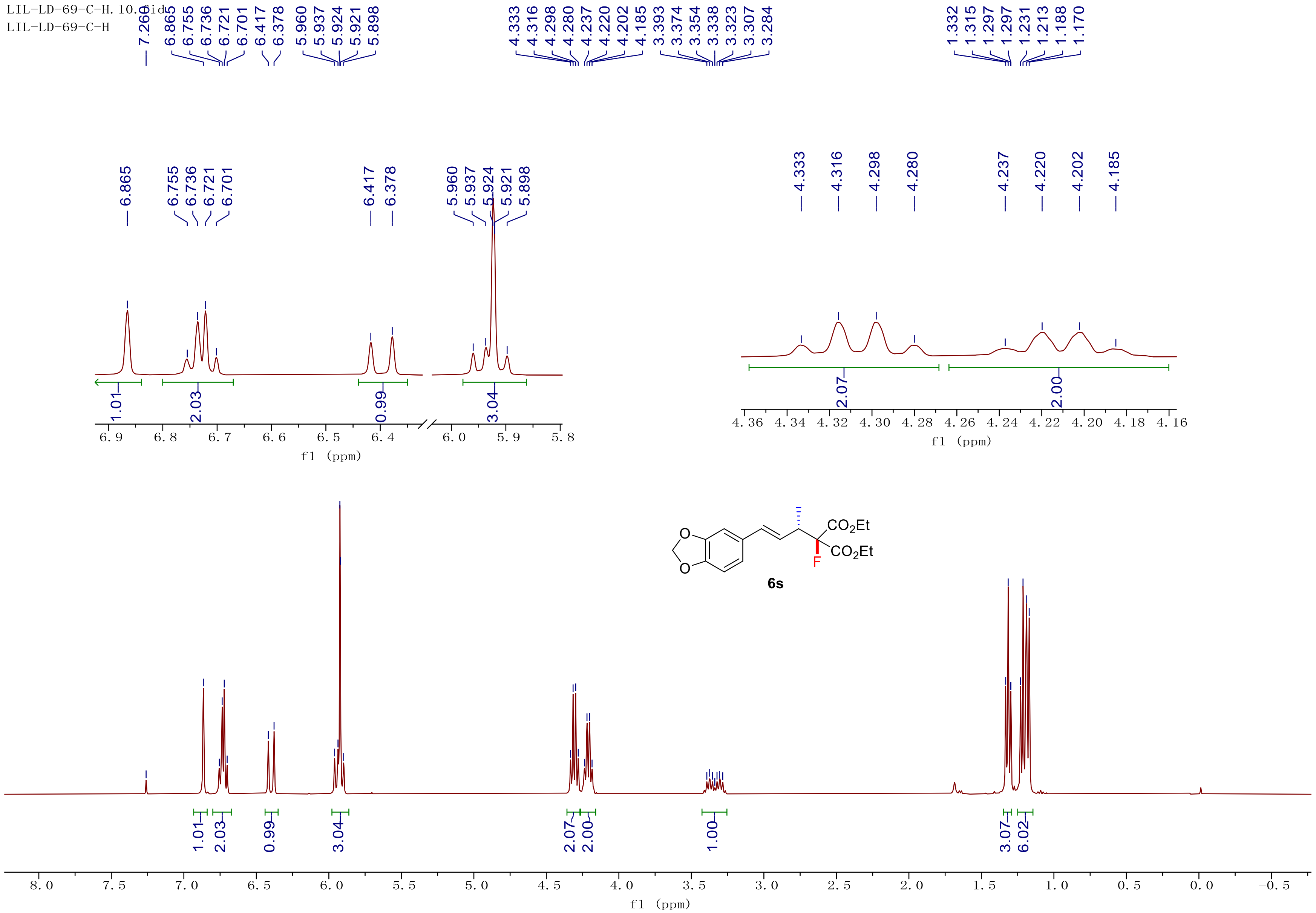
LIL-LD-69-C-H.10.fid
LIL-LD-69-C-H
7.260
6.865 6.755 6.736 6.721 6.701 6.417 6.378 5.960 5.937 5.924 5.921 5.898
4.333 4.316 4.298 4.280 4.237 4.220 4.202 4.185 3.393 3.374 3.354 3.338 3.323 3.307 3.284
1.332 1.315 1.297 1.297 1.231 1.213 1.188 1.170
1.01 2.03 0.99 3.04
2.07 2.00
1.00
3.07 6.02
CO2Et
CO2Et
F
O
O
6s
f1 (ppm)

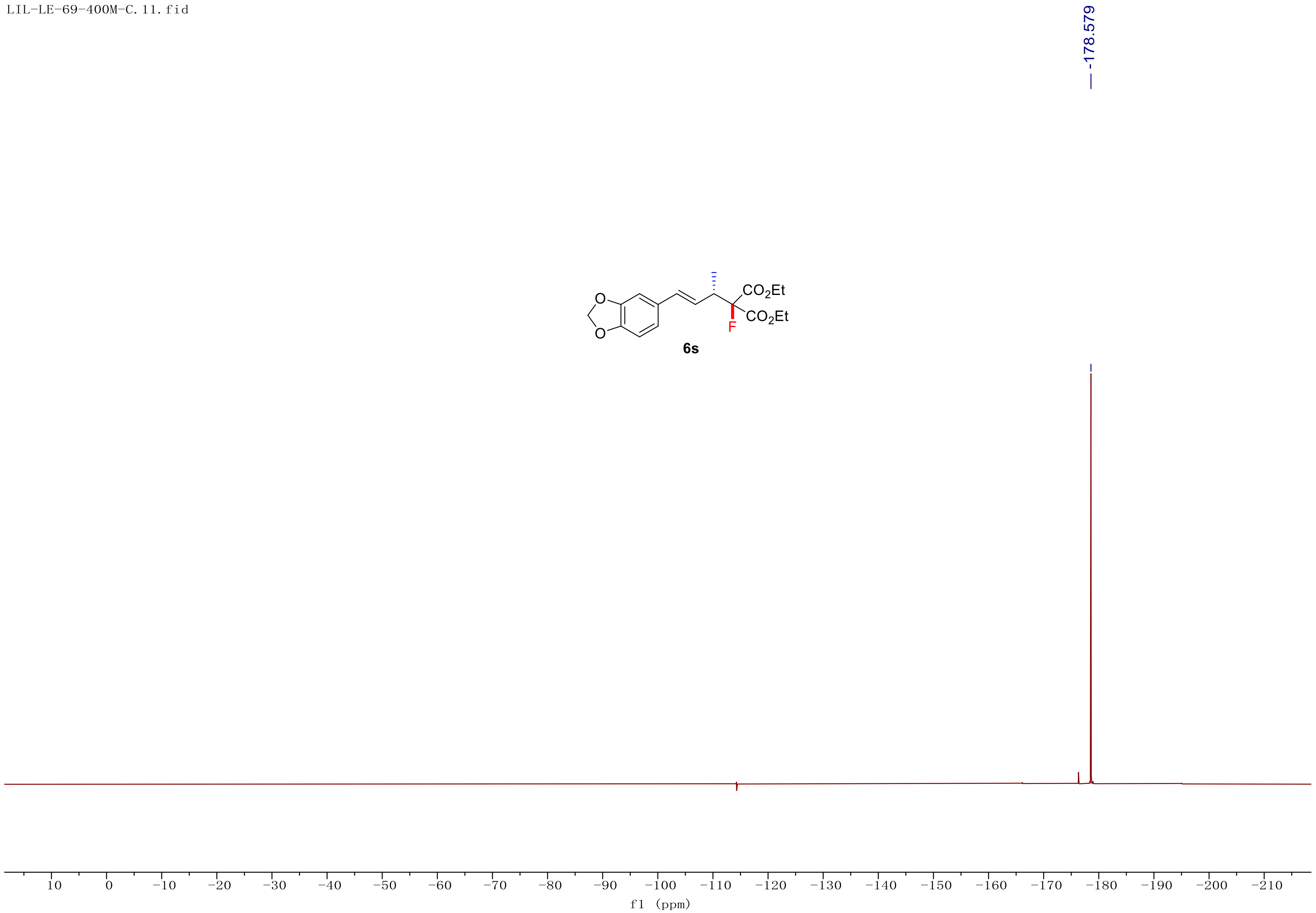
LIL-LE-69-400M-C.11.fid
-178.579
CO2Et
CO2Et
F
6s
10 0 −10 −20 −30 −40 −50 −60 −70 −80 −90 −100 −110 −120 −130 −140 −150 −160 −170 −180 −190 −200 −210
f1 (ppm)

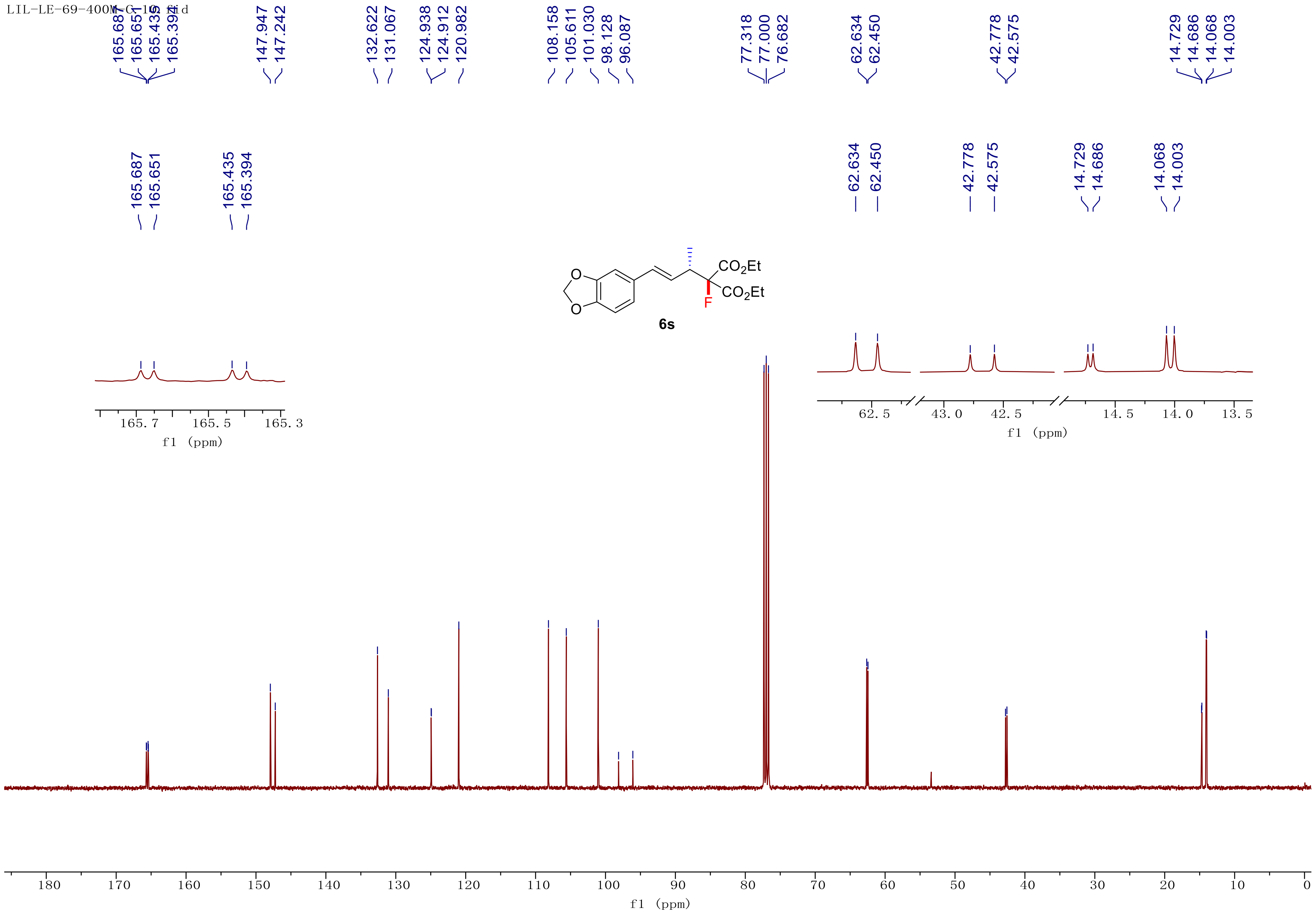
LIL-LE-69-400M-C-10.fid
165.687
165.651
165.435
165.394
147.947
147.242
132.622
131.067
124.938
124.912
120.982
108.158
105.611
101.030
98.128
96.087
77.318
77.000
76.682
62.634
62.450
42.778
42.575
14.729
14.686
14.068
14.003
CO2Et
CO2Et
F
O
O
6s
f1 (ppm)

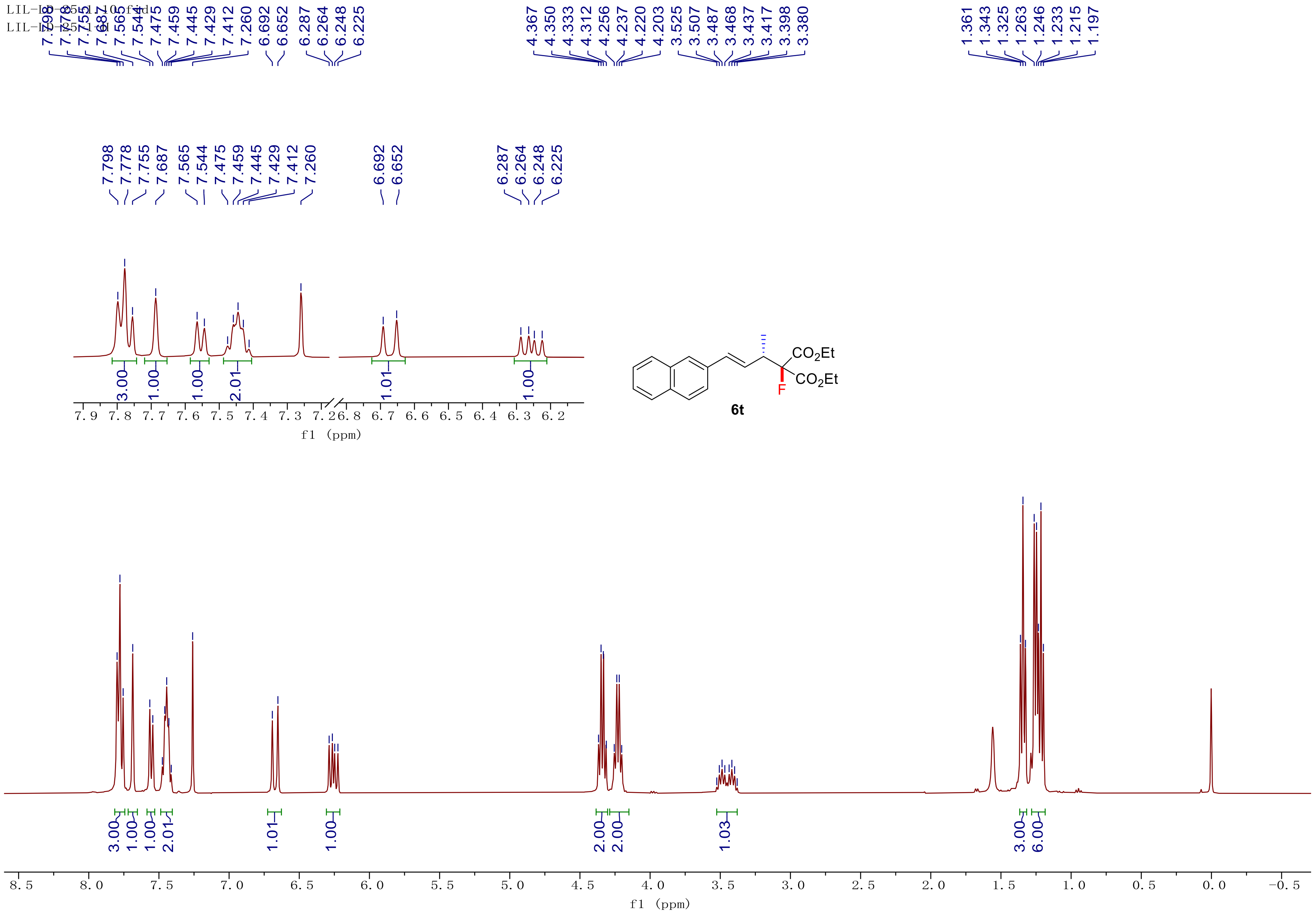

CO2Et
CO2Et
F
6t
f1 (ppm)

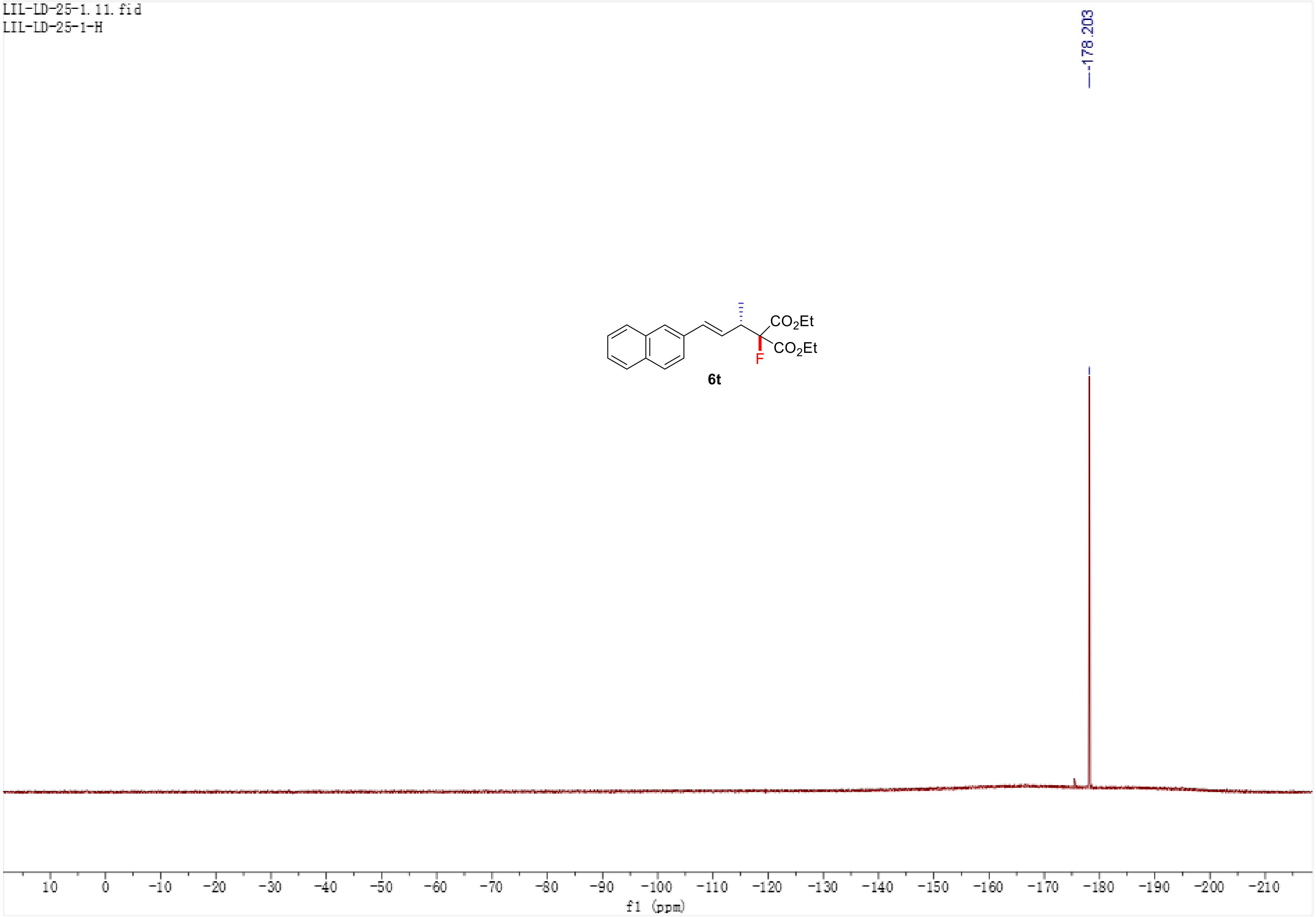

LIL-LD-25-1.11.fid
LIL-LD-25-1-H
-178.203
CO2Et
CO2Et
F
6t
10
0
-10
-20
-30
-40
-50
-60
-70
-80
-90
-100
-110
-120
-130
-140
-150
-160
-170
-180
-190
-200
-210
f1 (ppm)

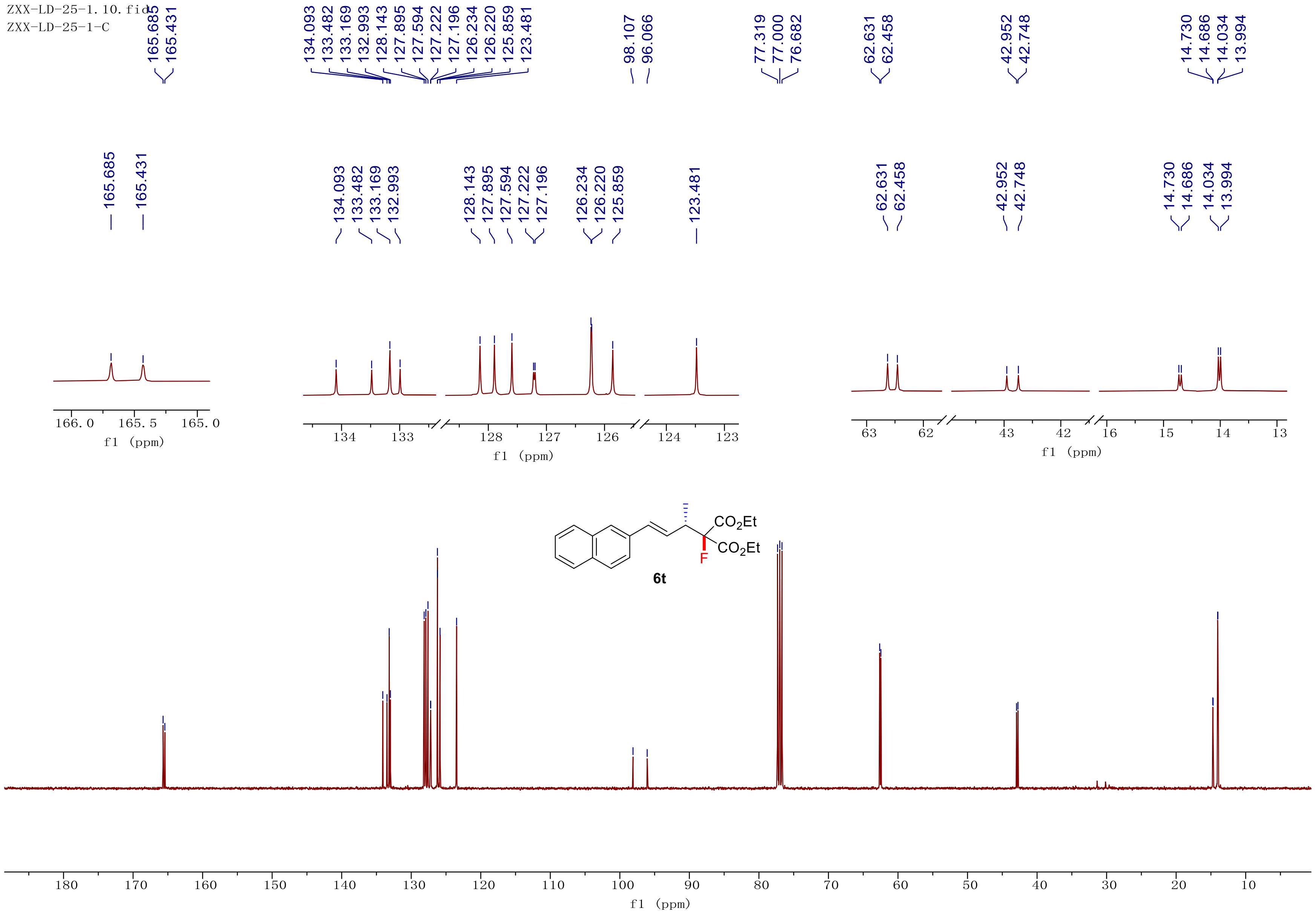
ZXX-LD-25-1.10.fid
ZXX-LD-25-1-C
165.685
165.431
134.093
133.482
133.169
132.993
128.143
127.895
127.594
127.222
127.196
126.234
126.220
125.859
123.481
98.107
96.066
77.319
77.000
76.682
62.631
62.458
42.952
42.748
14.730
14.686
14.034
13.994
CO2Et
CO2Et
F
6t
f1 (ppm)

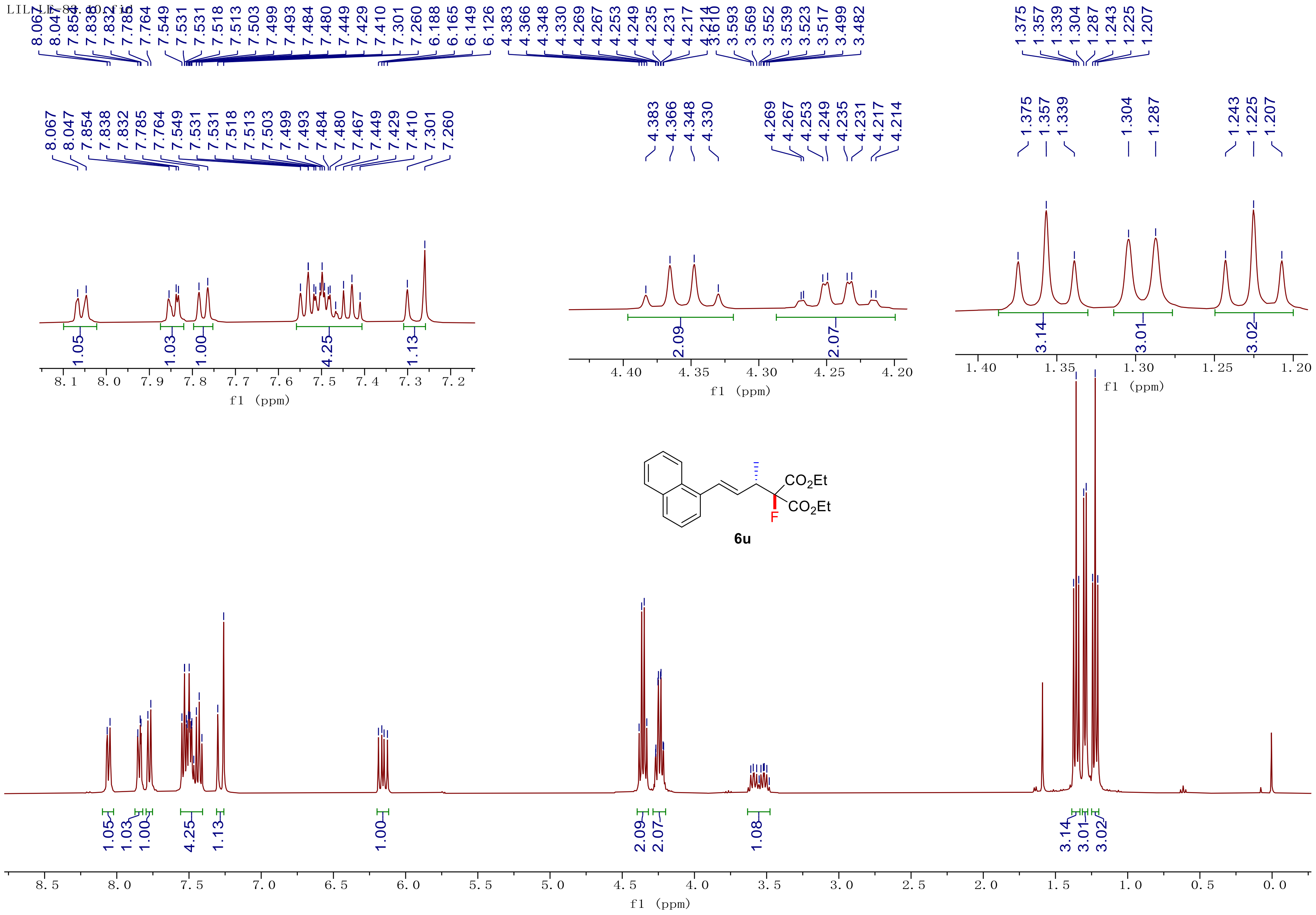
LILILF-84.10.fid
CO2Et
CO2Et
F
6u
f1 (ppm)

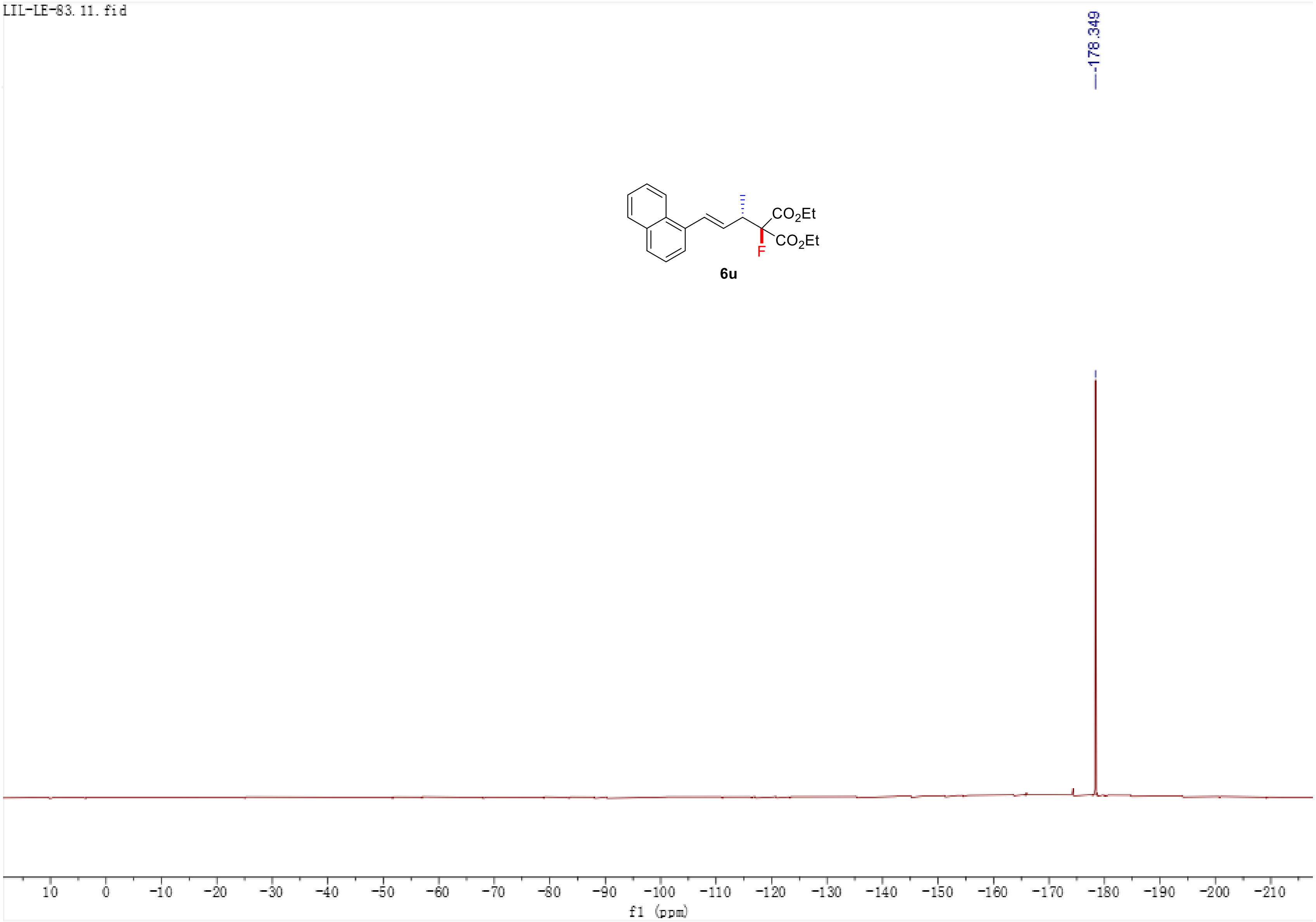
LIL-LE-83.11.fid
-178.349
CO2Et
CO2Et
F
6u
10 0 −10 −20 −30 −40 −50 −60 −70 −80 −90 −100 −110 −120 −130 −140 −150 −160 −170 −180 −190 −200 −210
f1 (ppm)

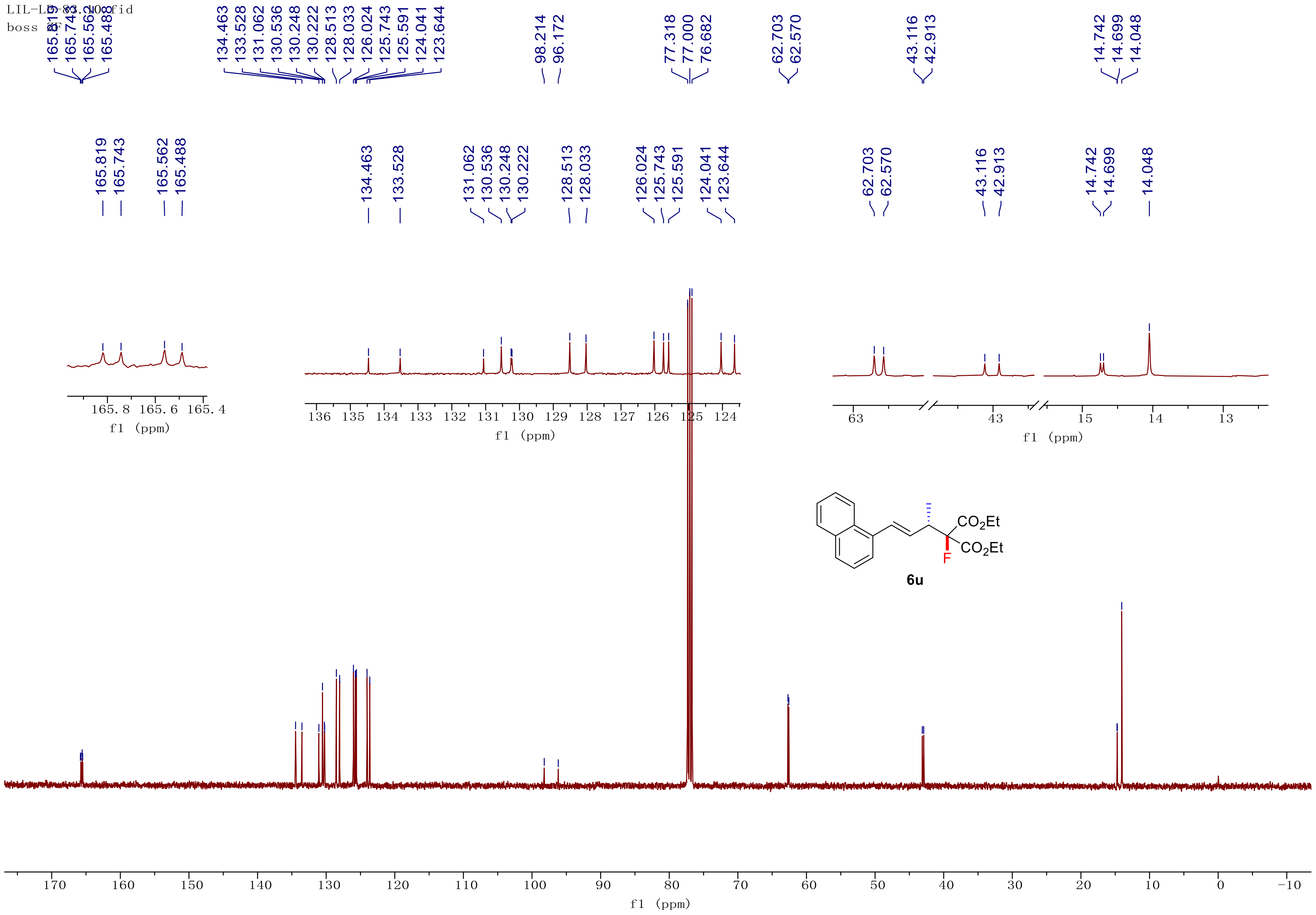

165.819
165.743
165.562
165.488
134.463
133.528
131.062
130.536
130.248
130.222
128.513
128.033
126.024
125.743
125.591
124.041
123.644
98.214
96.172
77.318
77.000
76.682
62.703
62.570
43.116
42.913
14.742
14.699
14.048
f1 (ppm)
CO2Et
CO2Et
F
6u

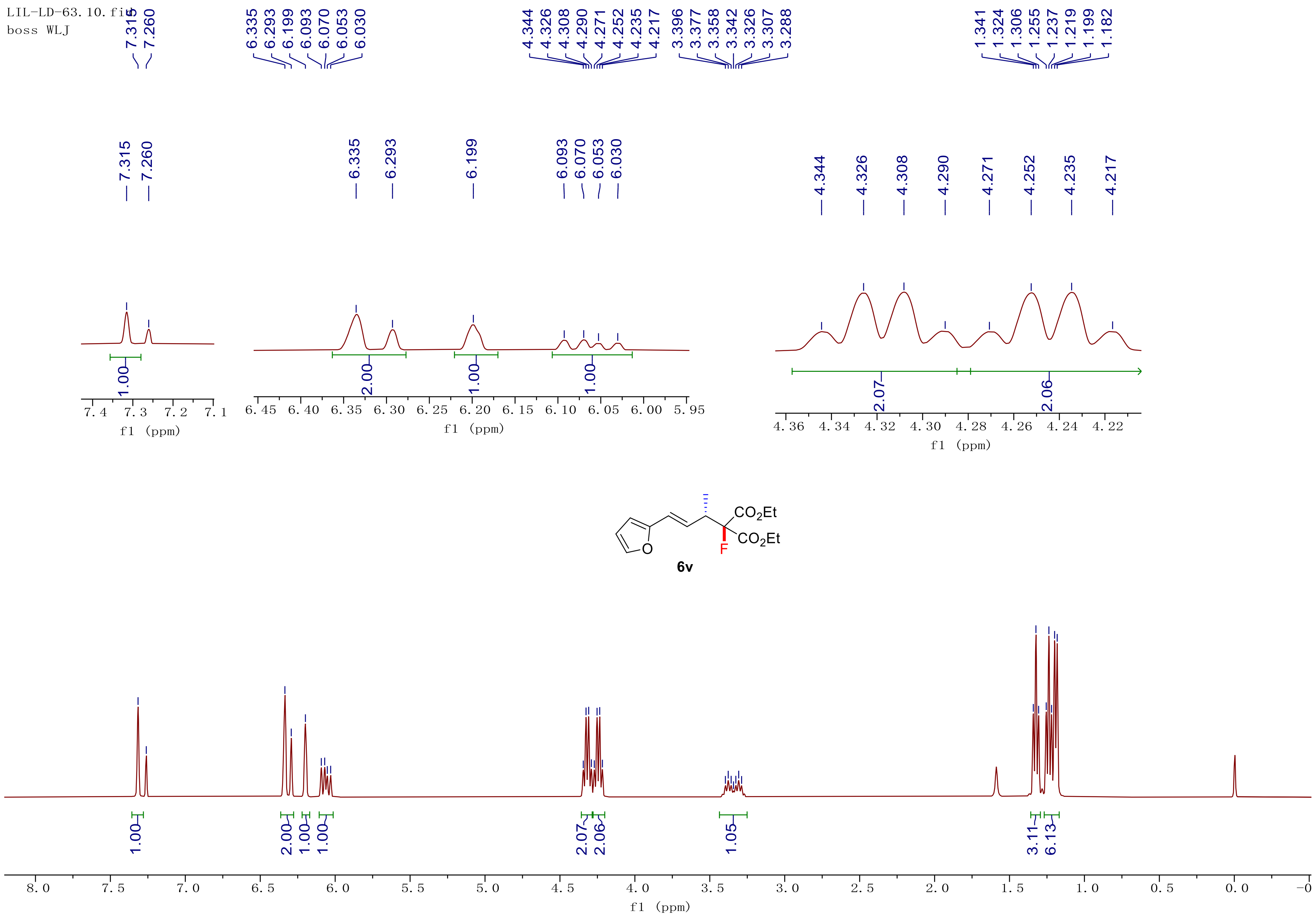
LIL-LD-63.10.fid
boss WLJ
7.315
7.260
6.335
6.293
6.199
6.093
6.070
6.053
6.030
4.344
4.326
4.308
4.290
4.271
4.252
4.235
4.217
3.396
3.377
3.358
3.342
3.326
3.307
3.288
1.341
1.324
1.306
1.255
1.237
1.219
1.199
1.182
1.00
2.00
1.00
1.00
2.07
2.06
1.05
3.11
6.13
f1 (ppm)
CO2Et
CO2Et
F
O
6v

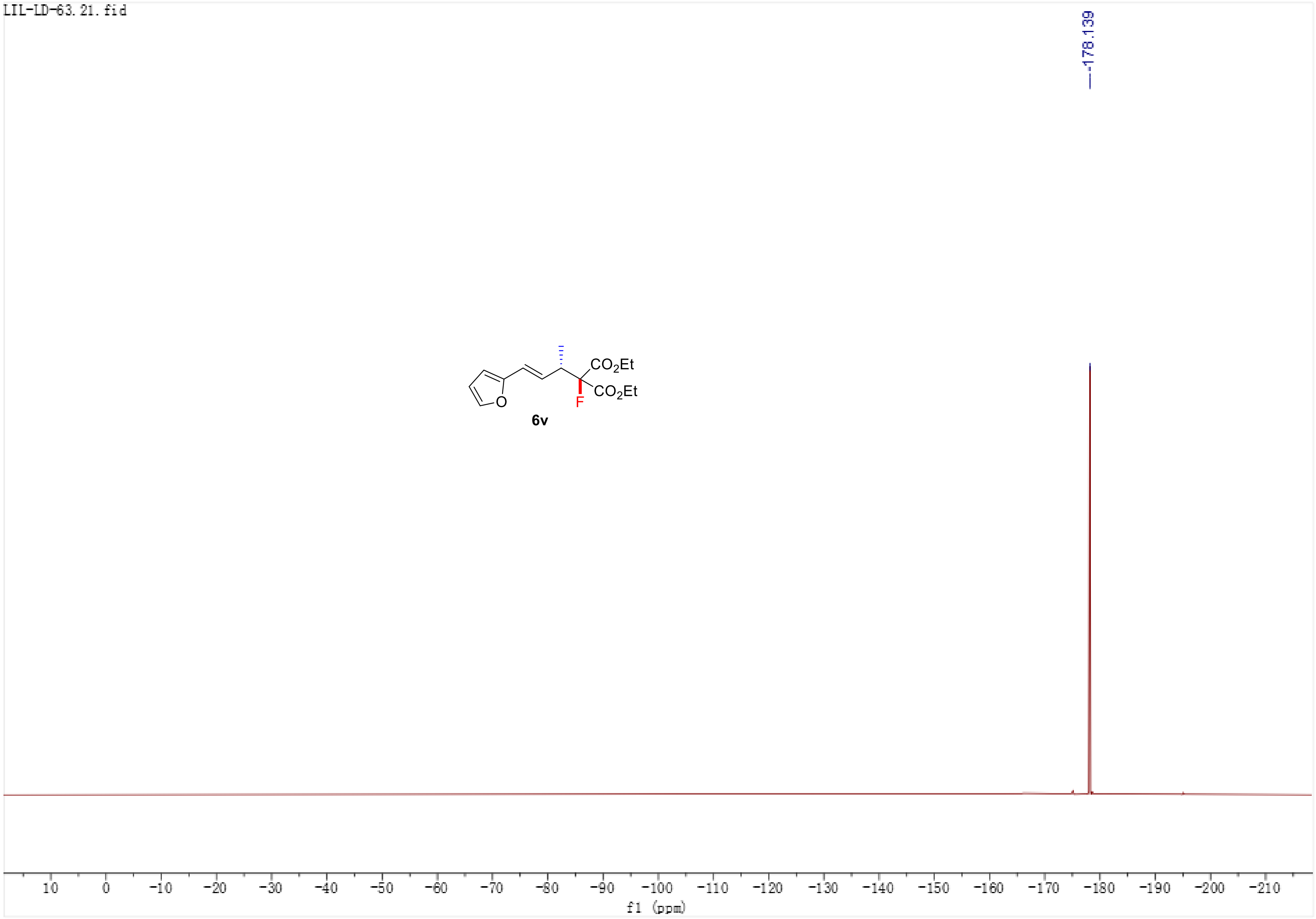
LIL-LD-63.21.fid
-178.139
CO2Et
CO2Et
F
O
6v
10
0
-10
-20
-30
-40
-50
-60
-70
-80
-90
-100
-110
-120
-130
-140
-150
-160
-170
-180
-190
-200
-210
f1 (ppm)

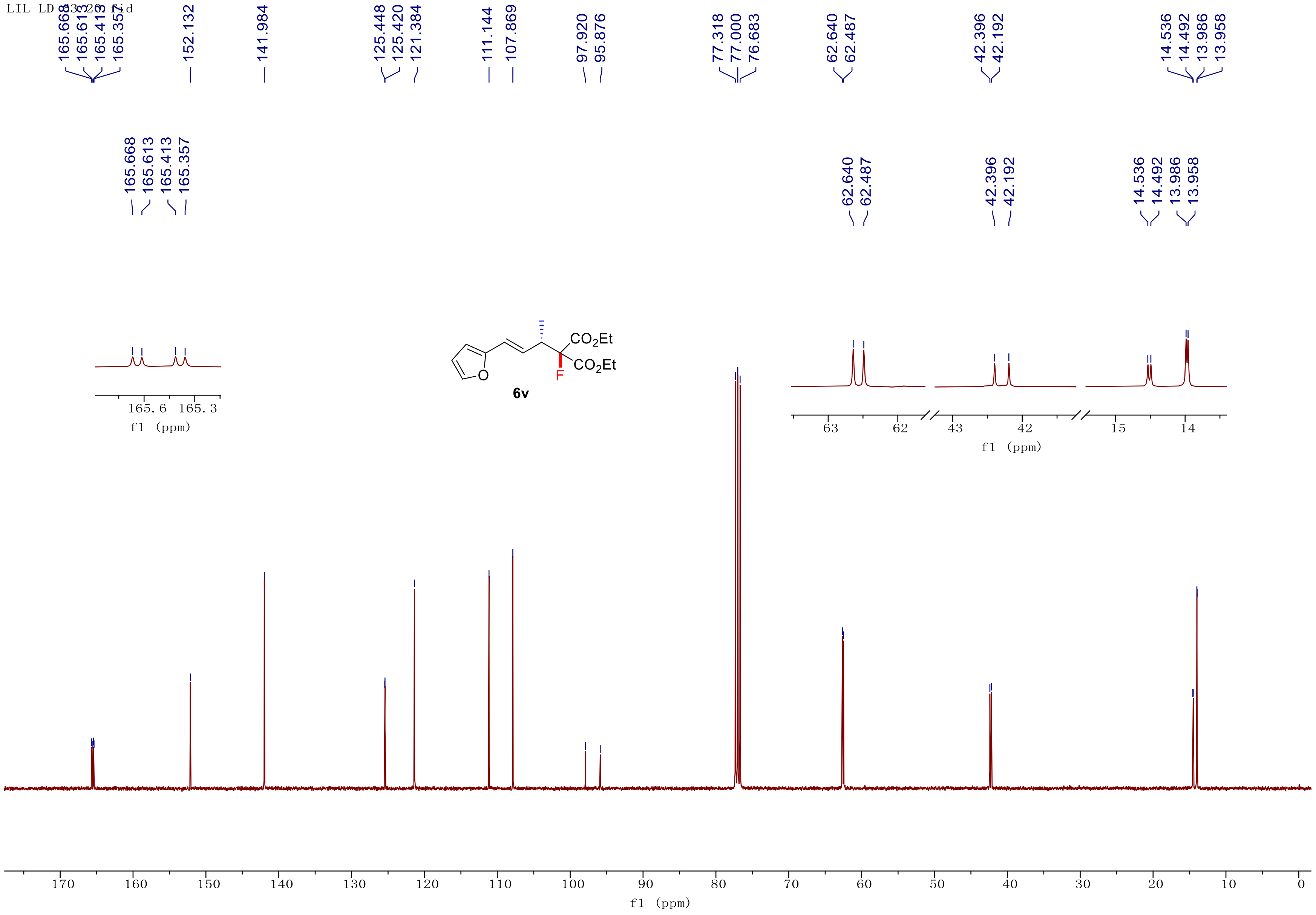
LIL-LD-03-20.fid
165.668
165.613
165.413
165.357
152.132
141.984
125.448
125.420
121.384
111.144
107.869
97.920
95.876
77.318
77.000
76.683
62.640
62.487
42.396
42.192
14.536
14.492
13.986
13.958
CO2Et
CO2Et
F
O
6v
f1 (ppm)

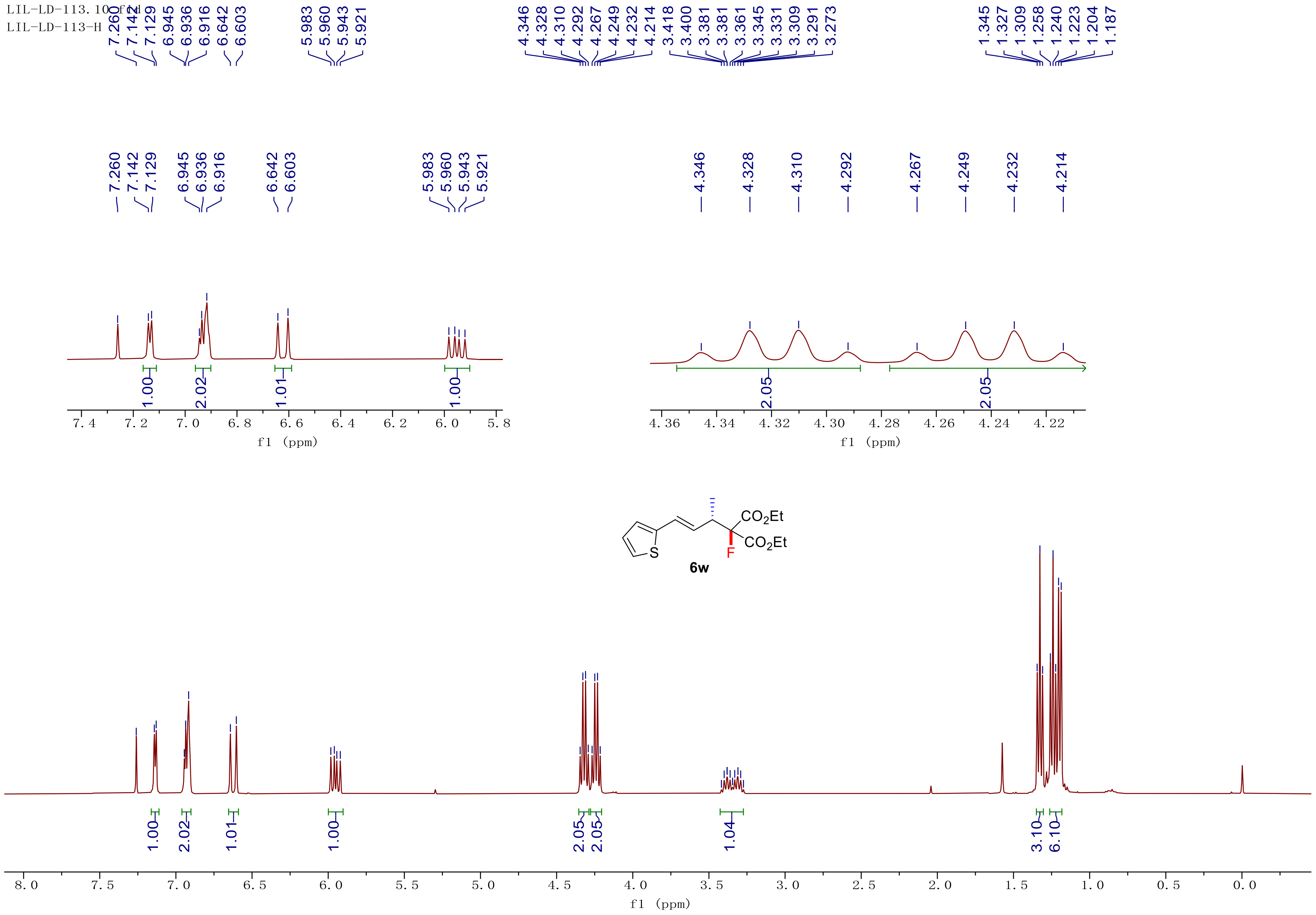
LIL-LD-113.10.fid
LIL-LD-113-H
6w
f1 (ppm)

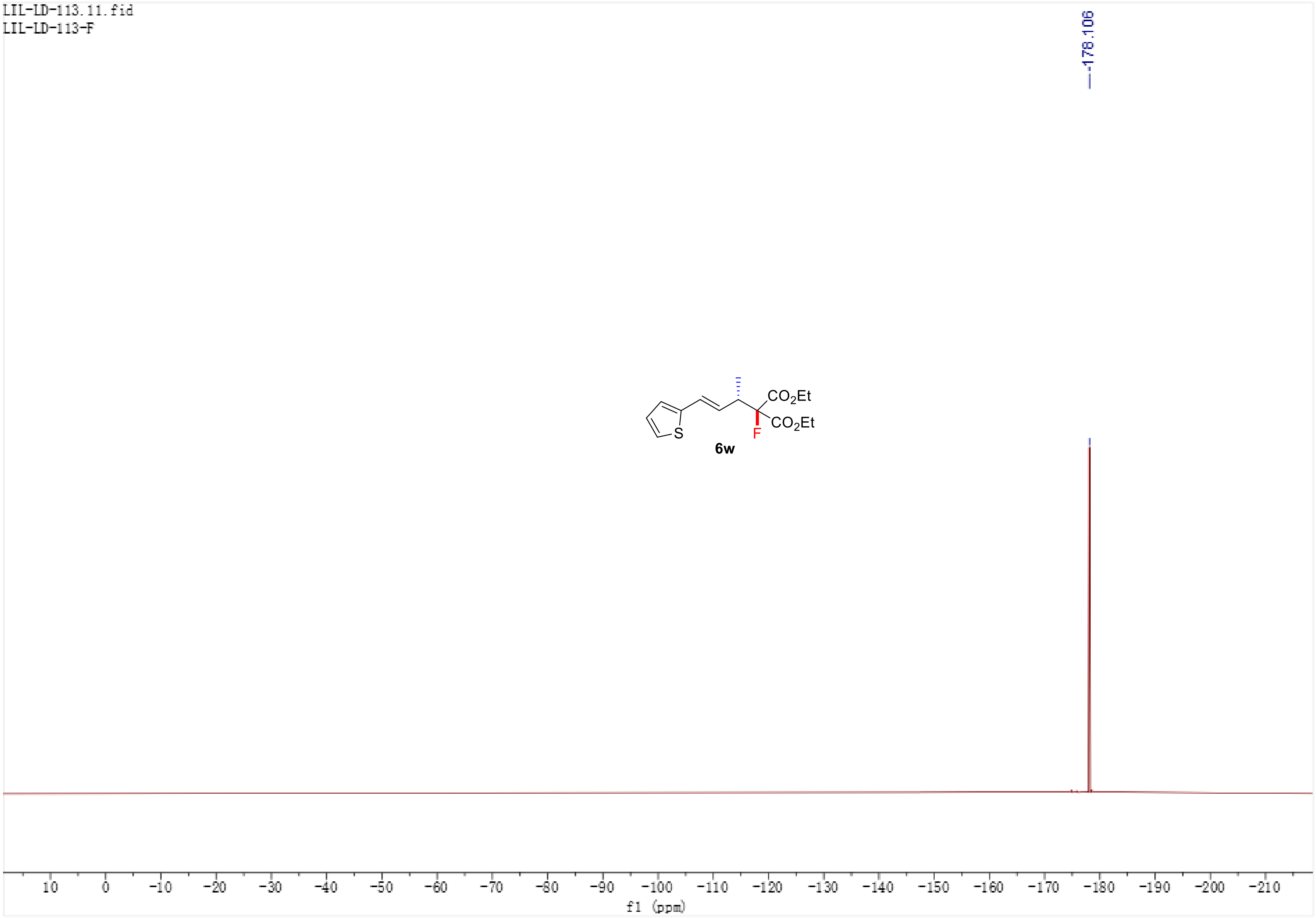
LIL-LD-113.11.fid
LIL-LD-113-F
-178.106
CO2Et
CO2Et
S
F
6w
10 0 −10 −20 −30 −40 −50 −60 −70 −80 −90 −100 −110 −120 −130 −140 −150 −160 −170 −180 −190 −200 −210
f1 (ppm)

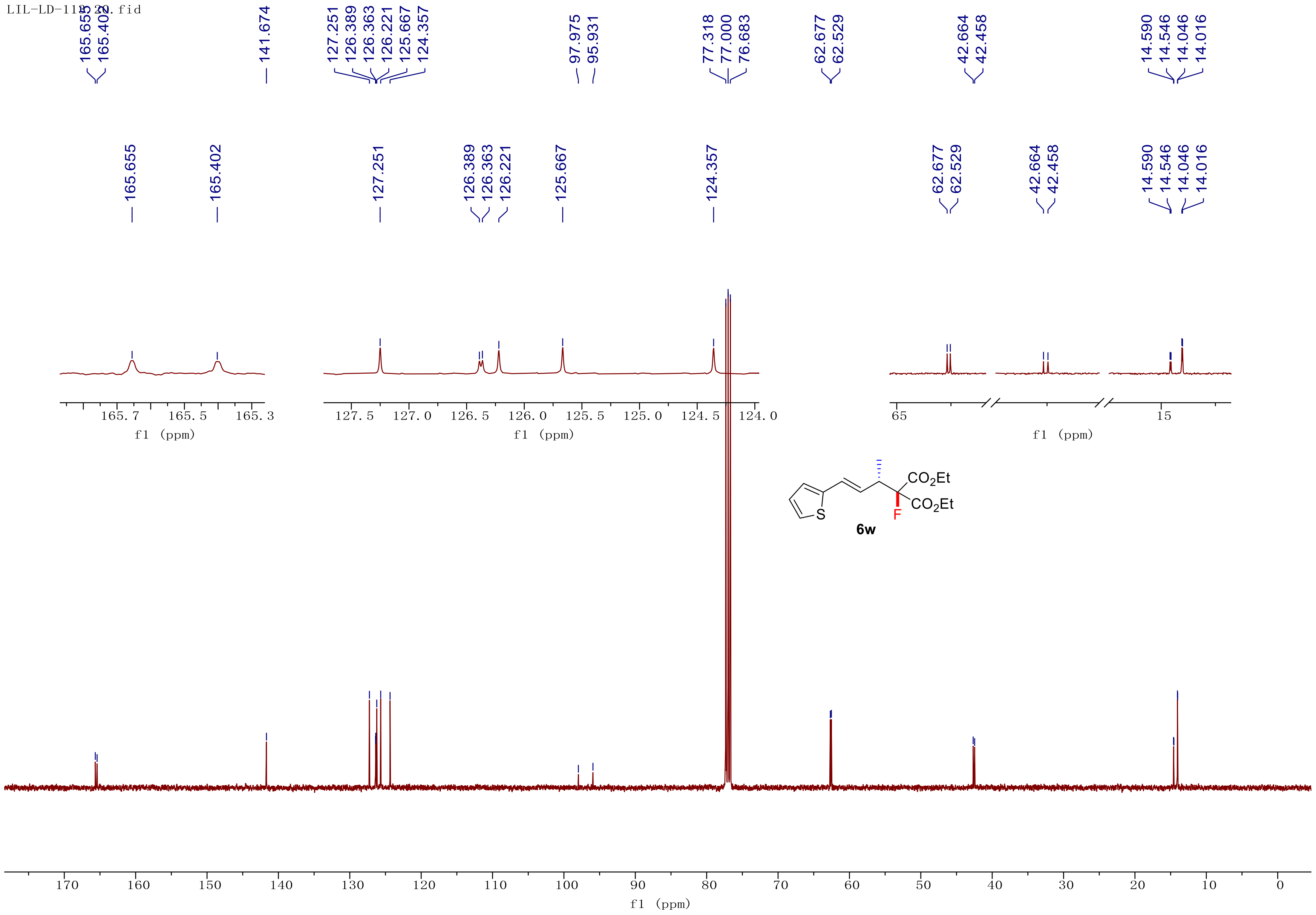

LIL-LD-118 20.fid
165.655
165.402
141.674
127.251
126.389
126.363
126.221
125.667
124.357
97.975
95.931
77.318
77.000
76.683
62.677
62.529
42.664
42.458
14.590
14.546
14.046
14.016
f1 (ppm)
CO2Et
CO2Et
S
F

6w

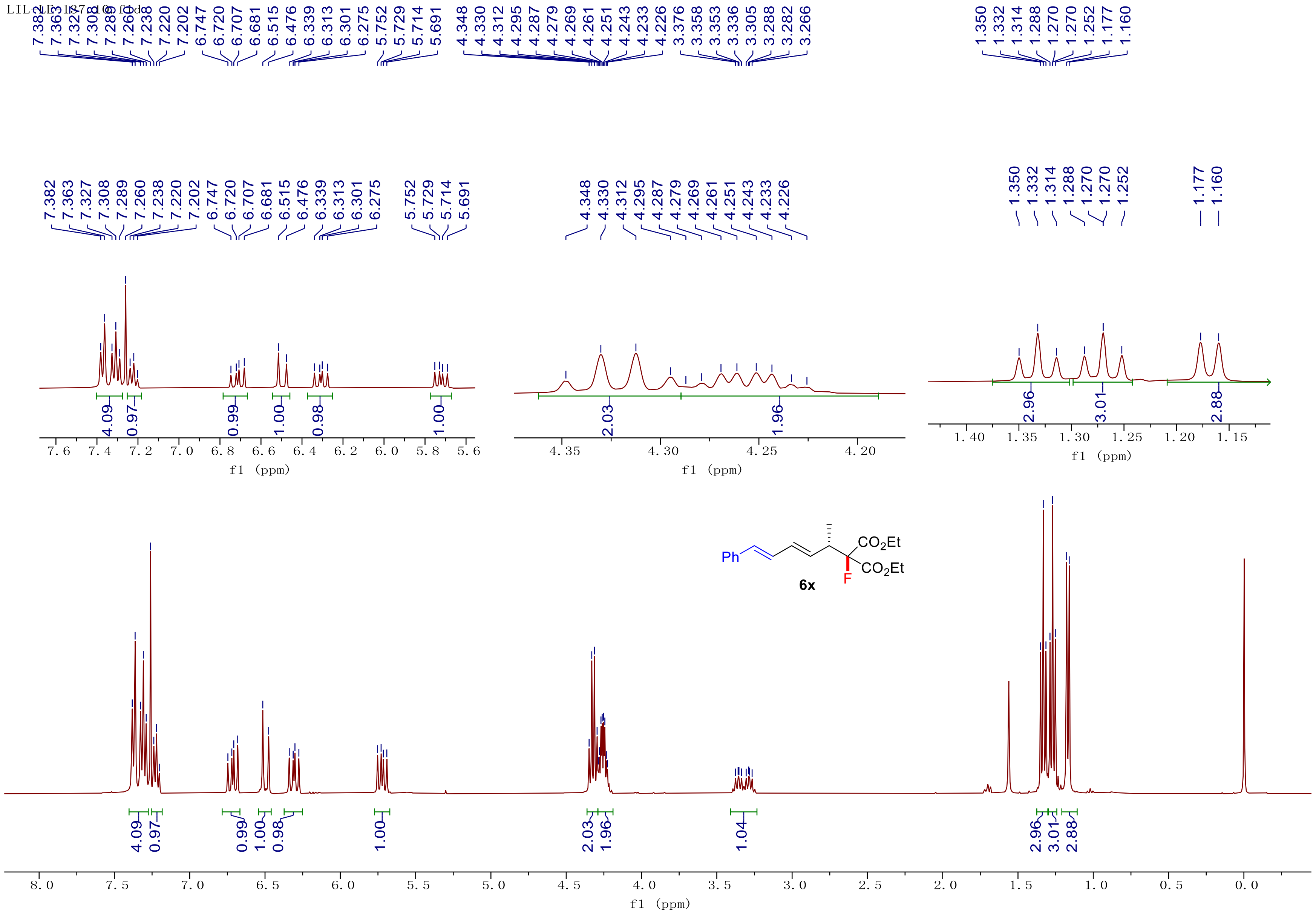
LIL-LR-187-10.fid
Ph
CO_2Et
CO_2Et
F
6x
f1 (ppm)

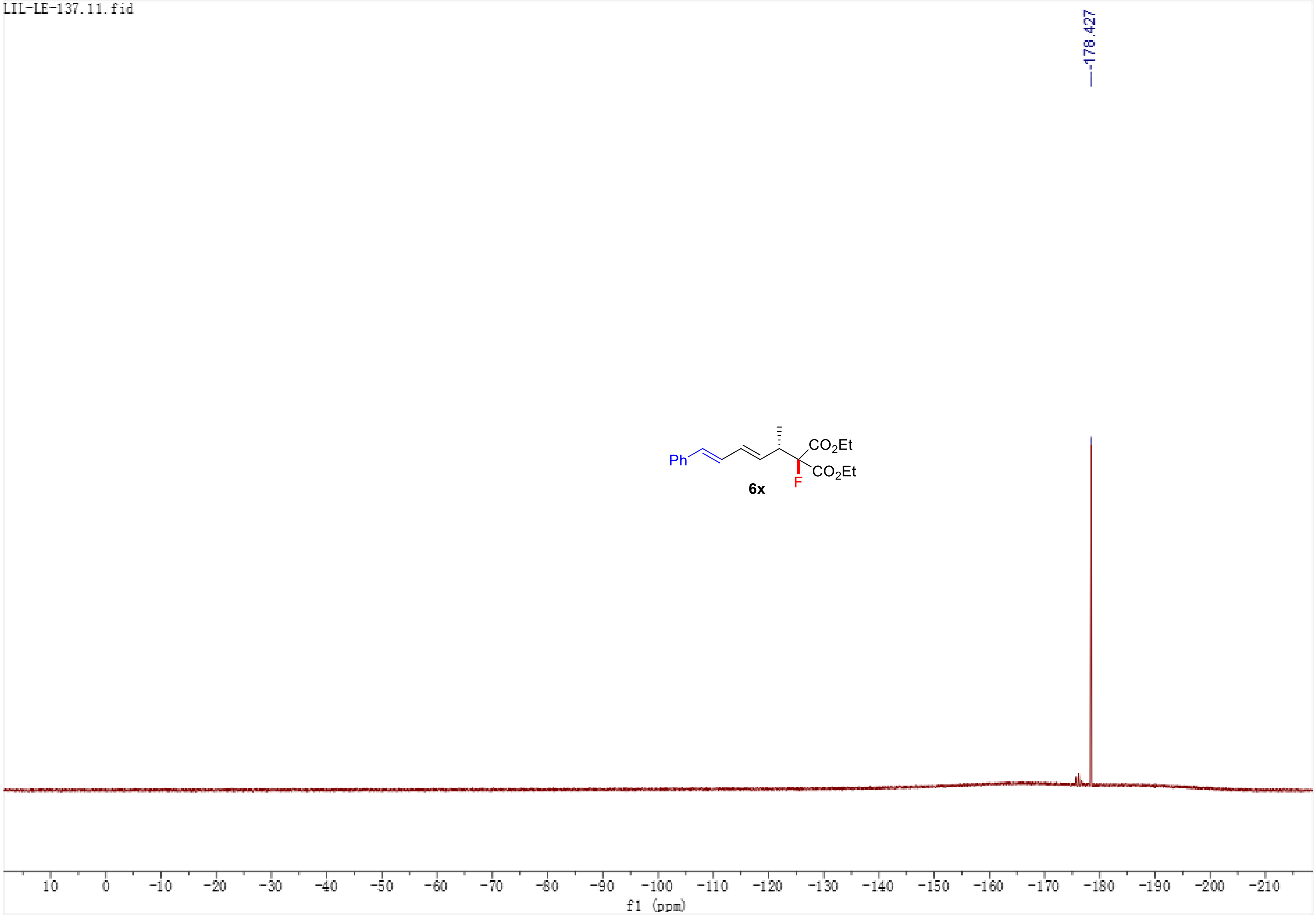
LIL-LE-137.11.fid
-178.427
Ph
CO_2Et
CO_2Et
F
6x
f1 (ppm)

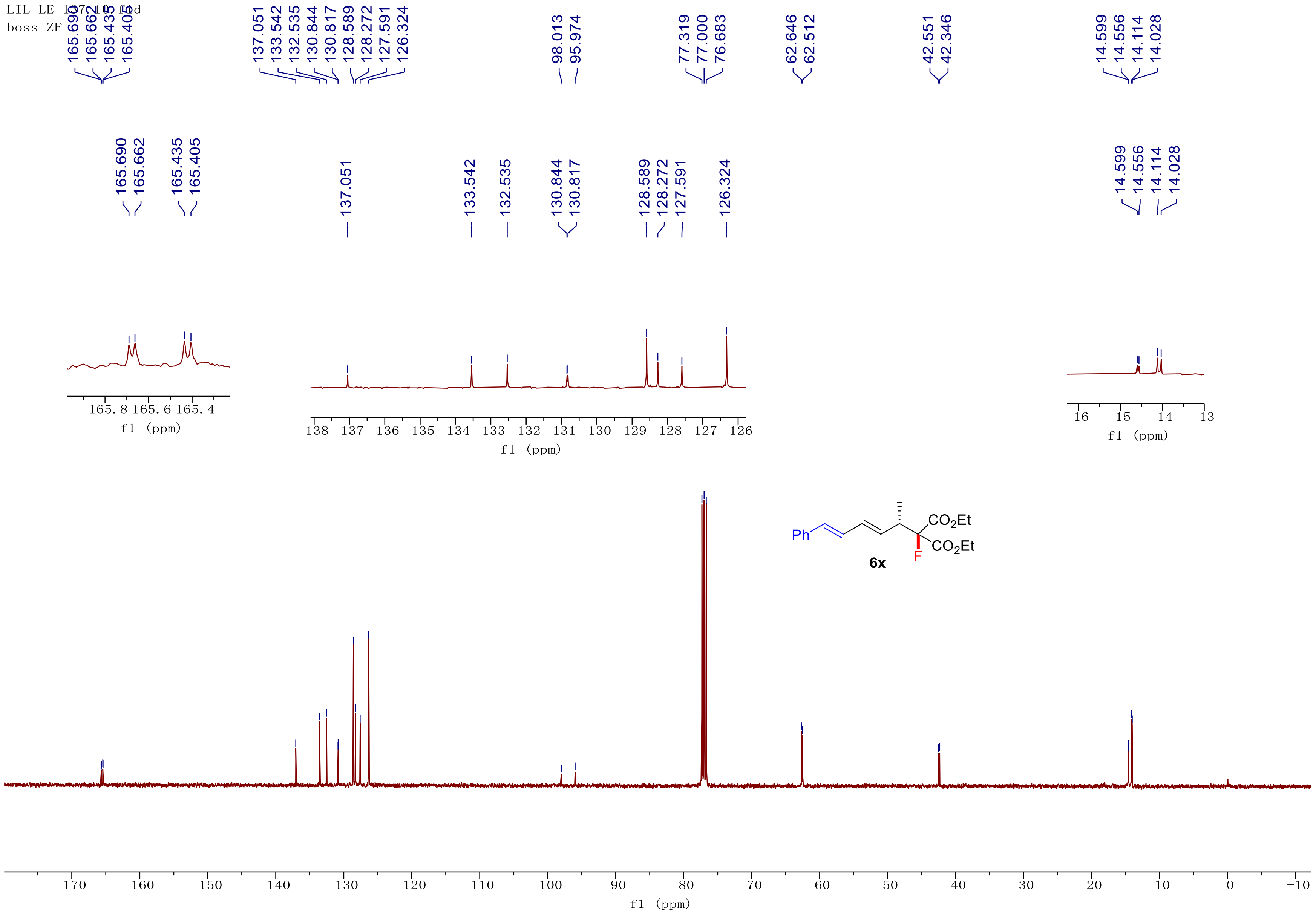
LIL-LE-137.10.fid
boss ZF
6x
f1 (ppm)

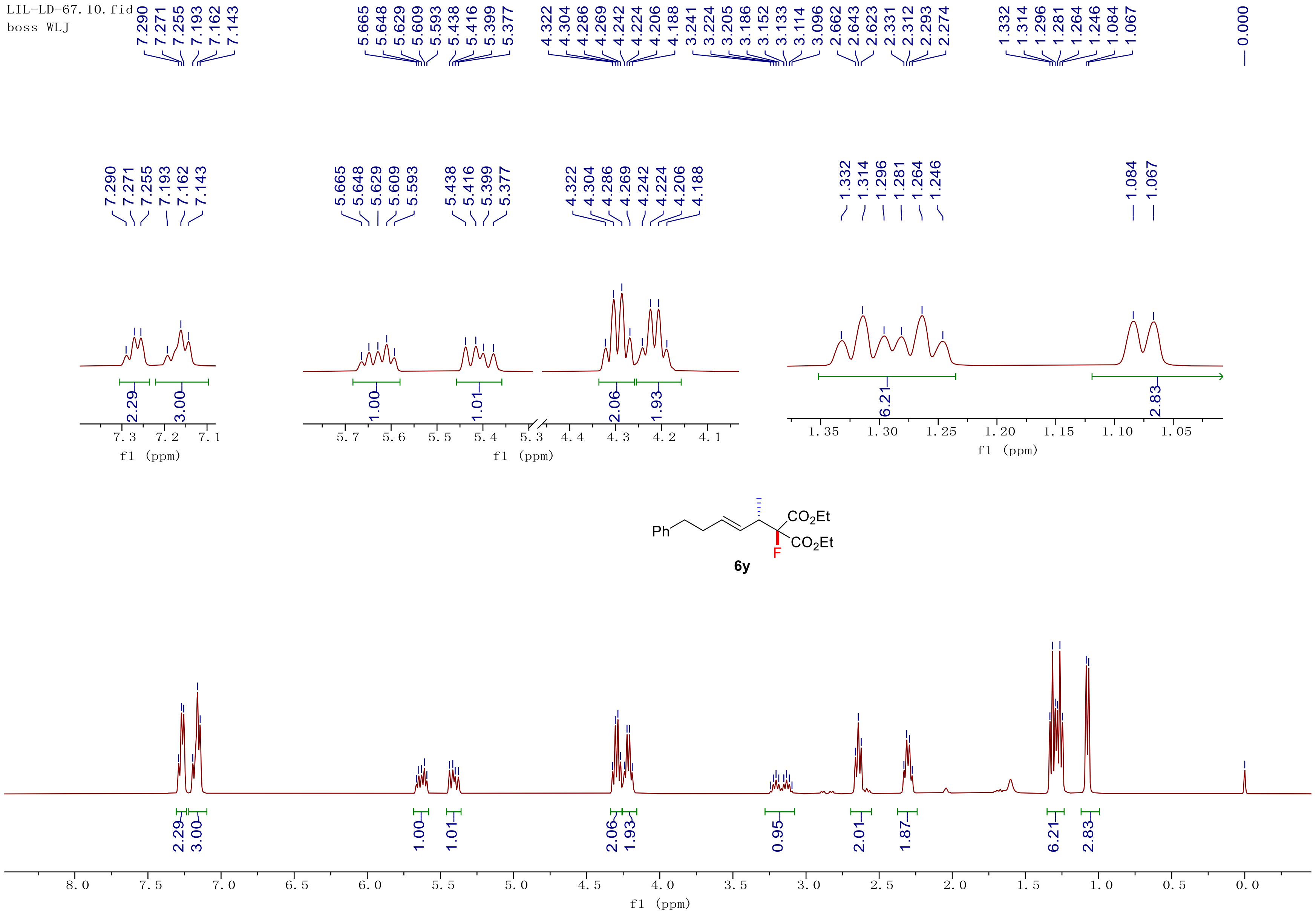
LIL-LD-67.10.fid
boss WLJ
7.290 7.271 7.255 7.193 7.162 7.143
5.665 5.648 5.629 5.609 5.593 5.438 5.416 5.399 5.377
4.322 4.304 4.286 4.269 4.242 4.224 4.206 4.188
3.241 3.224 3.205 3.186 3.152 3.133 3.114 3.096
2.662 2.643 2.623 2.331 2.312 2.293 2.274
1.332 1.314 1.296 1.281 1.264 1.246 1.084 1.067
0.000
2.29 3.00 1.00 1.01 2.06 1.93 0.95 2.01 1.87 6.21 2.83
Ph
CO_2Et
CO_2Et
F
6y
f1 (ppm)

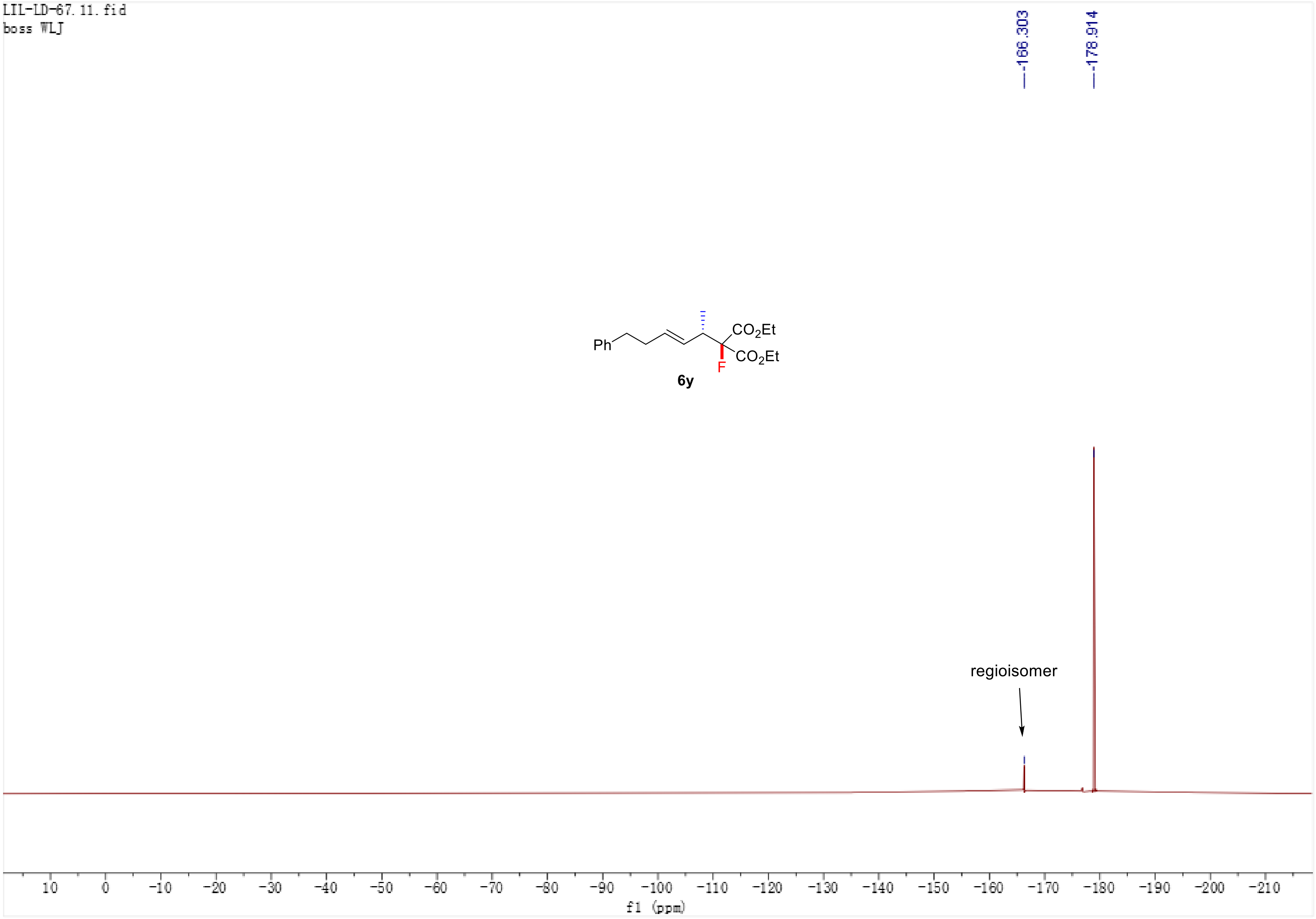

LIL-LD-67.11.fid
boss WLJ
-166.303
-178.914
Ph
CO2Et
CO2Et
F
6y
regioisomer
f1 (ppm)

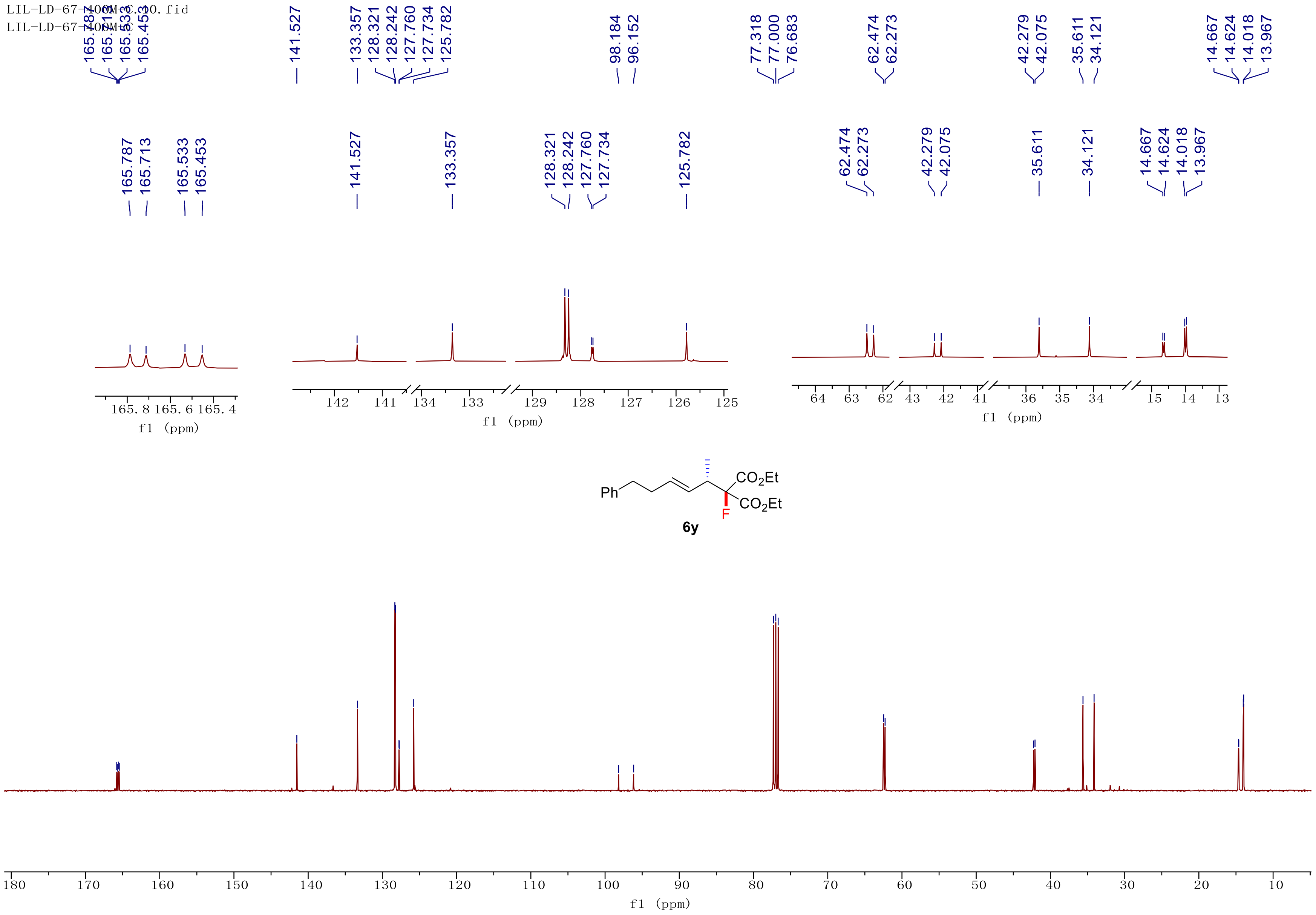
LIL-LD-67-400M-C.10.fid
LIL-LD-67-400M-C
165.787
165.713
165.533
165.453
141.527
133.357
128.321
128.242
127.760
127.734
125.782
98.184
96.152
77.318
77.000
76.683
62.474
62.273
42.279
42.075
35.611
34.121
14.667
14.624
14.018
13.967
f1 (ppm)
Ph
CO_2Et
CO_2Et
F
6y

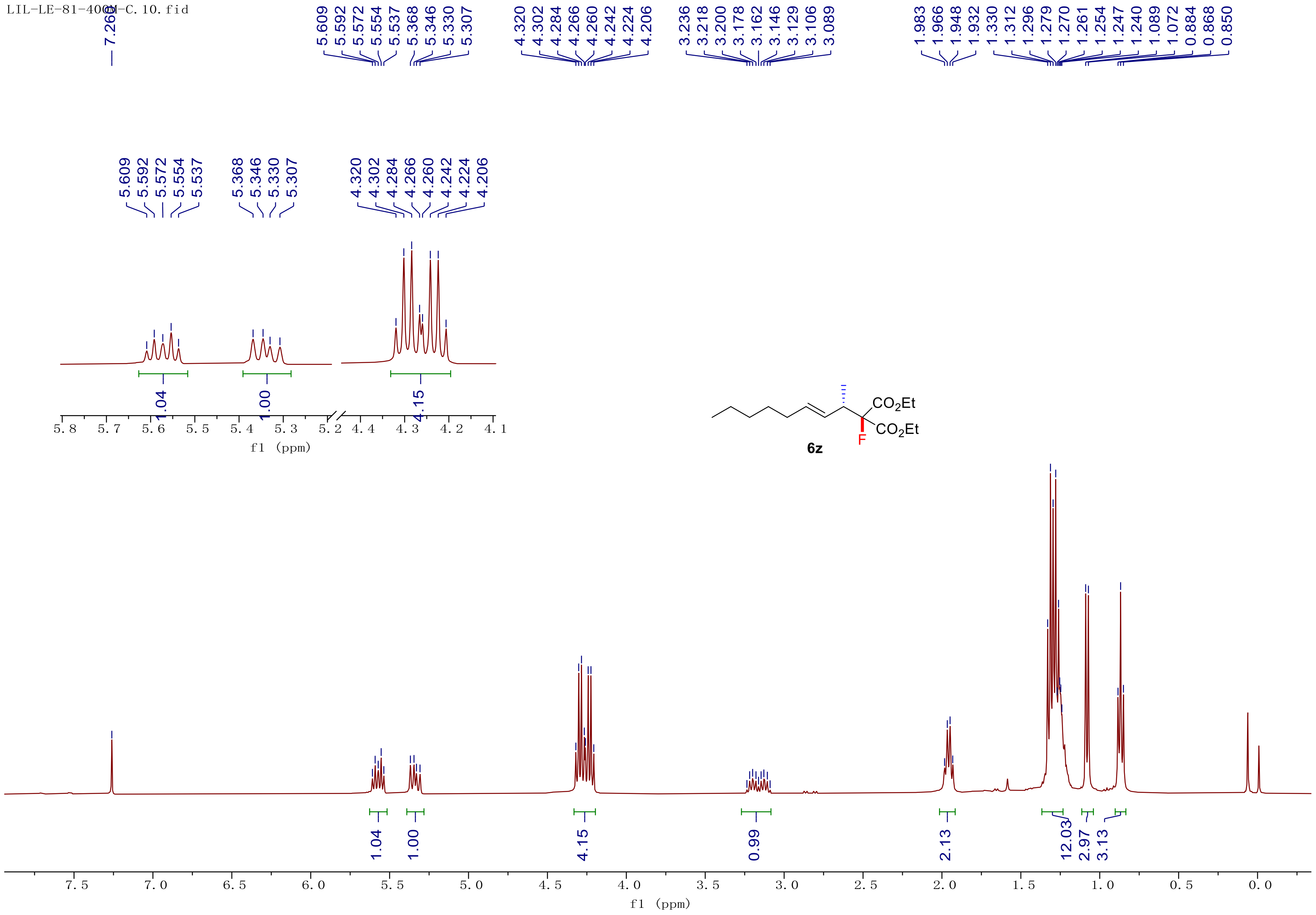
LIL-LE-81-400M-C.10.fid
7.260
5.609
5.592
5.572
5.554
5.537
5.368
5.346
5.330
5.307
4.320
4.302
4.284
4.266
4.260
4.242
4.224
4.206
3.236
3.218
3.200
3.178
3.162
3.146
3.129
3.106
3.089
1.983
1.966
1.948
1.932
1.330
1.312
1.296
1.279
1.270
1.261
1.254
1.247
1.240
1.089
1.072
0.884
0.868
0.850
1.04
1.00
4.15
0.99
2.13
12.03
2.97
3.13
f1 (ppm)
CO2Et
CO2Et
F
6z

LIL-LE-81-400M-C.12.fid

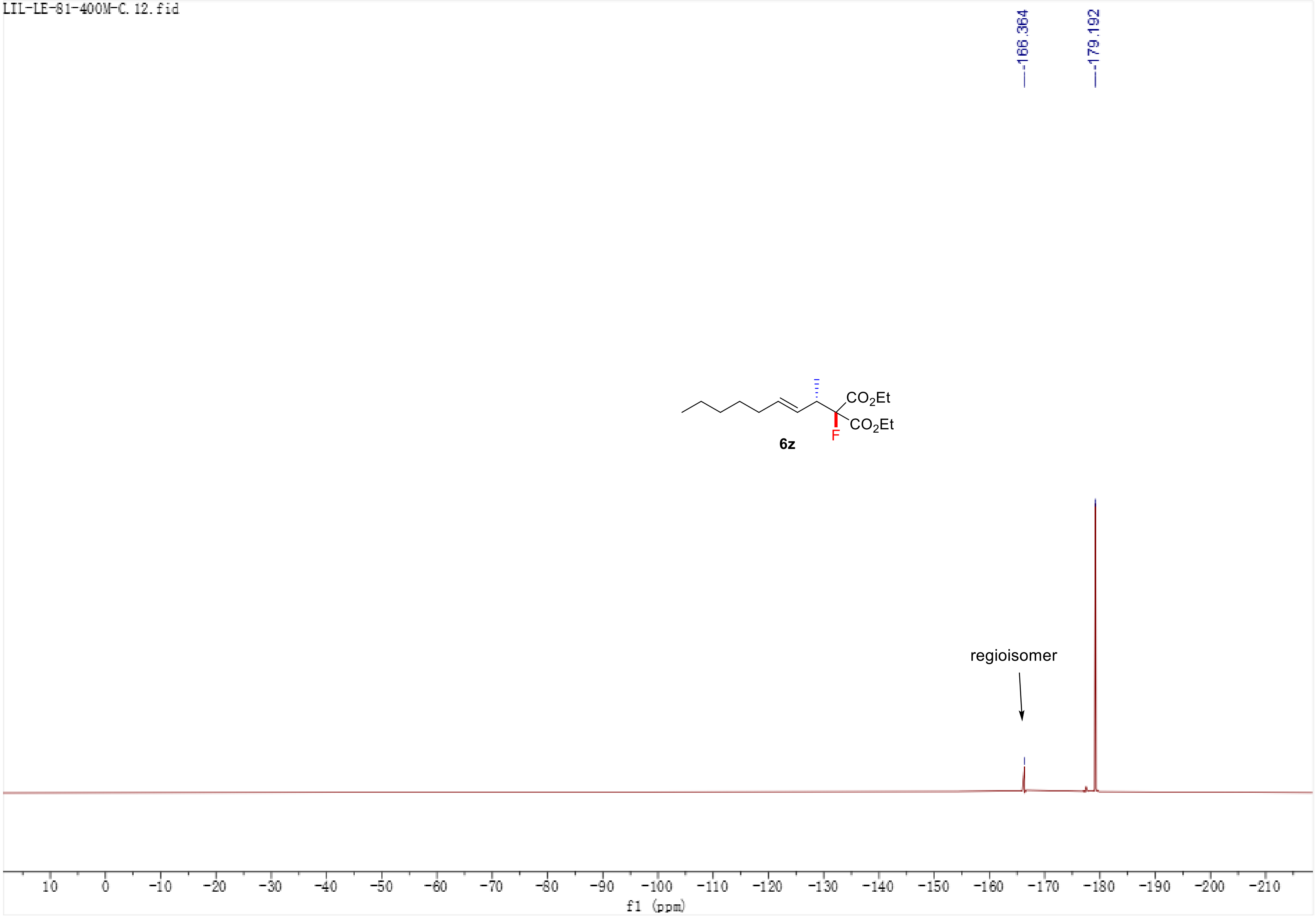

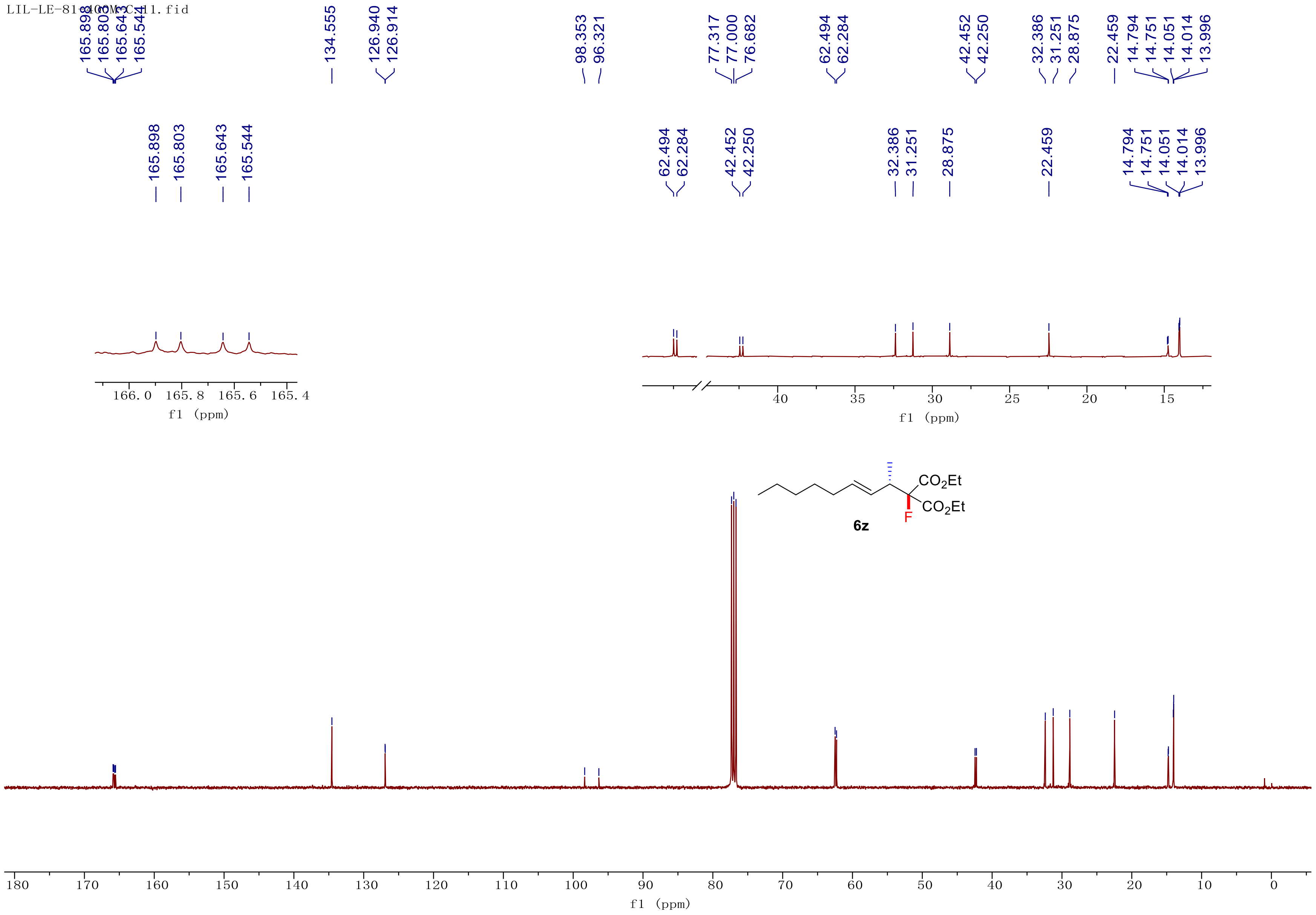
LIL-LE-81 400M-C.11.fid
165.898
165.803
165.643
165.544
134.555
126.940
126.914
98.353
96.321
77.317
77.000
76.682
62.494
62.284
42.452
42.250
32.386
31.251
28.875
22.459
14.794
14.751
14.051
14.014
13.996
CO2Et
CO2Et
F
6z
f1 (ppm)

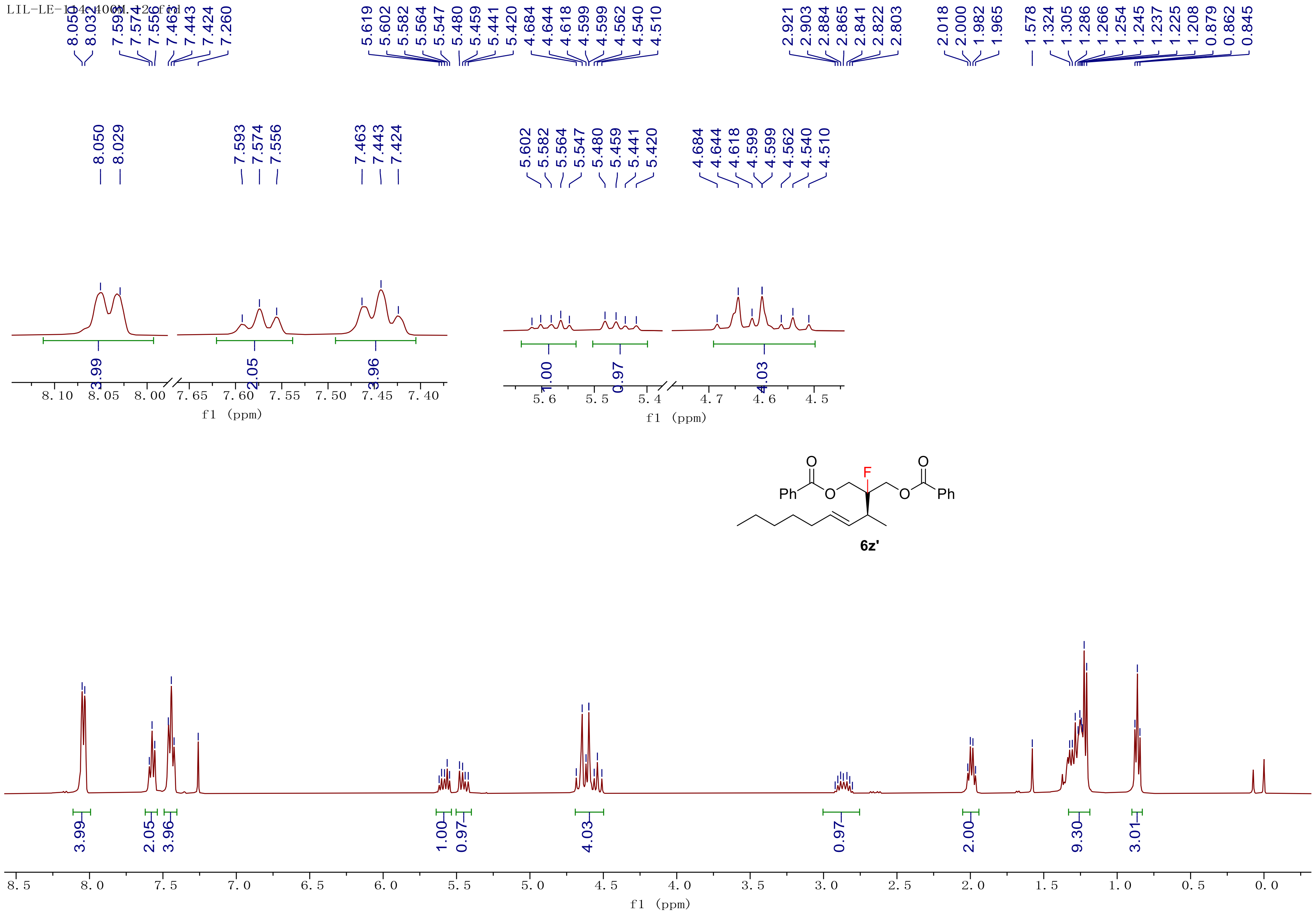
8.050 8.032 7.593 7.574 7.556 7.463 7.443 7.424 7.260
5.619 5.602 5.582 5.564 5.547 5.480 5.459 5.441 5.420 4.684 4.644 4.618 4.599 4.599 4.562 4.540 4.510
2.921 2.903 2.884 2.865 2.841 2.822 2.803
2.018 2.000 1.982 1.965
1.578 1.324 1.305 1.286 1.266 1.254 1.245 1.237 1.225 1.208 0.879 0.862 0.845
8.050 8.029
7.593 7.574 7.556
7.463 7.443 7.424
5.602 5.582 5.564 5.547 5.480 5.459 5.441 5.420
4.684 4.644 4.618 4.599 4.599 4.562 4.540 4.510
3.99 2.05 3.96
1.00 0.97 4.03
f1 (ppm)
O
F
O
Ph
O
O
Ph
6z'
3.99 2.05 3.96 1.00 0.97 4.03 0.97 2.00 9.30 3.01
f1 (ppm)

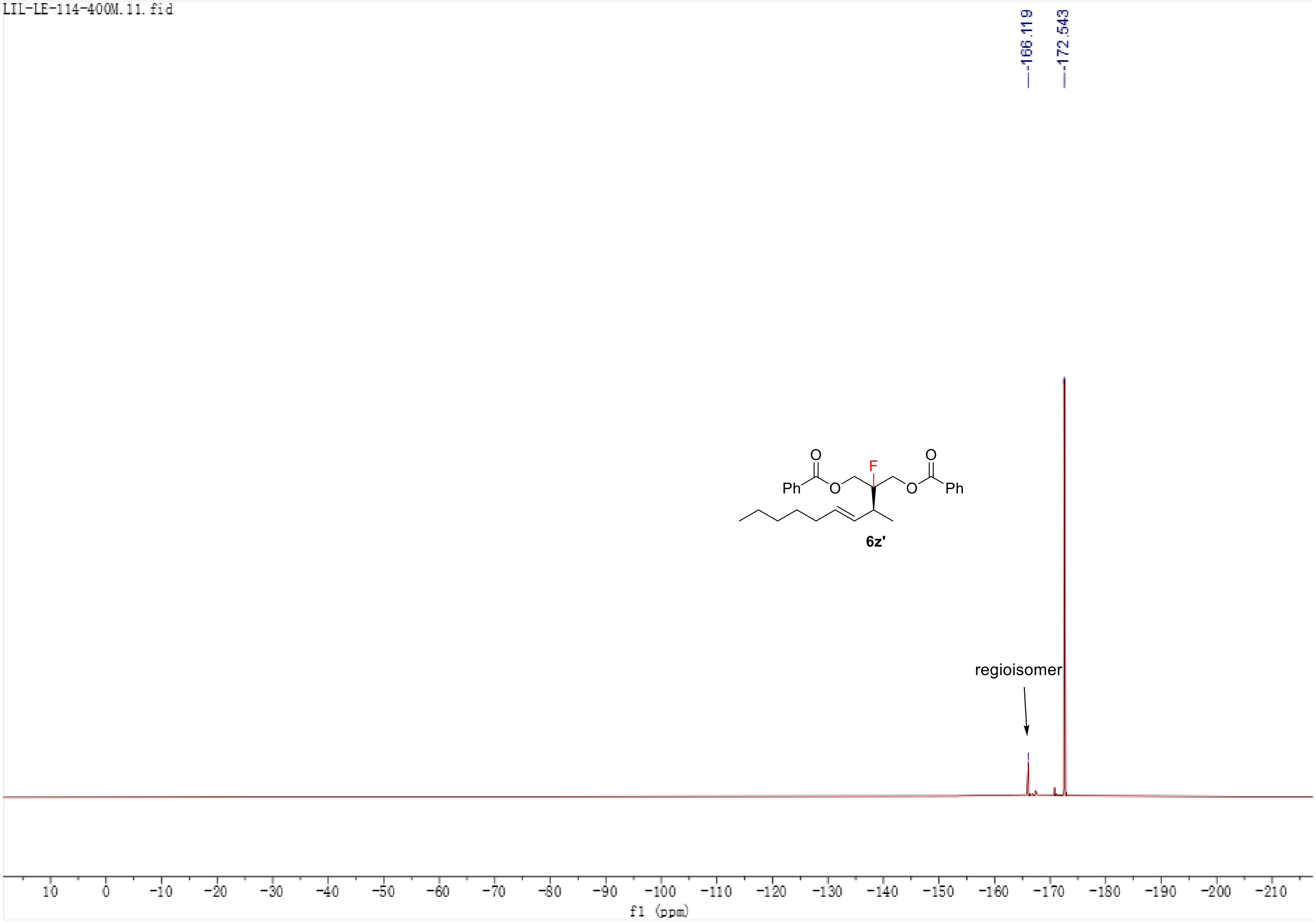
LIL-LE-114-400M.11.fid
-166.119
-172.543
O
F
O
Ph
O
O
Ph
6z'
regioisomer
10 0 -10 -20 -30 -40 -50 -60 -70 -80 -90 -100 -110 -120 -130 -140 -150 -160 -170 -180 -190 -200 -210
f1 (ppm)

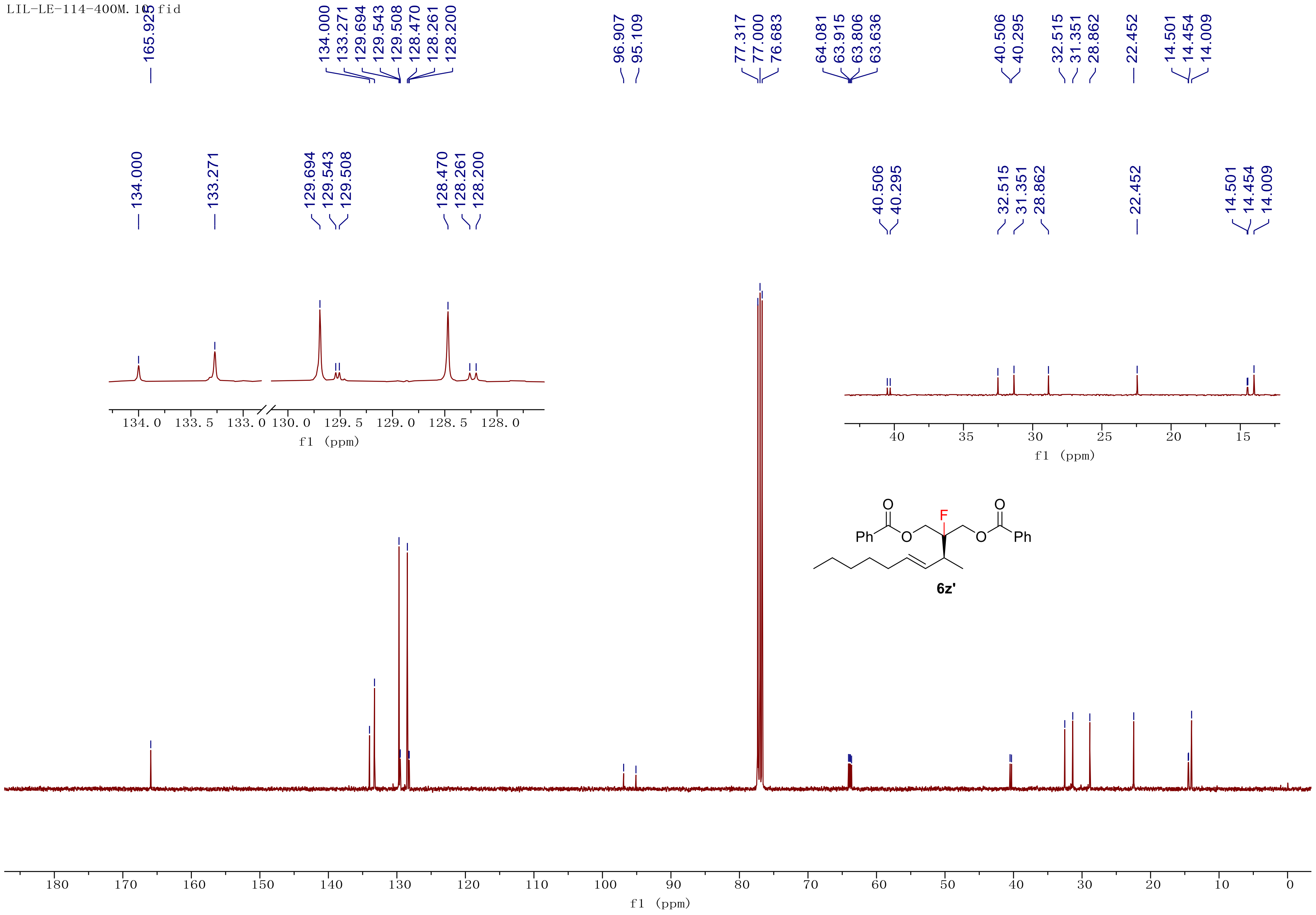
LIL-LE-114-400M.10.fid
165.925
134.000
133.271
129.694
129.543
129.508
128.470
128.261
128.200
96.907
95.109
77.317
77.000
76.683
64.081
63.915
63.806
63.636
40.506
40.295
32.515
31.351
28.862
22.452
14.501
14.454
14.009
f1 (ppm)
F
O
Ph
6z'

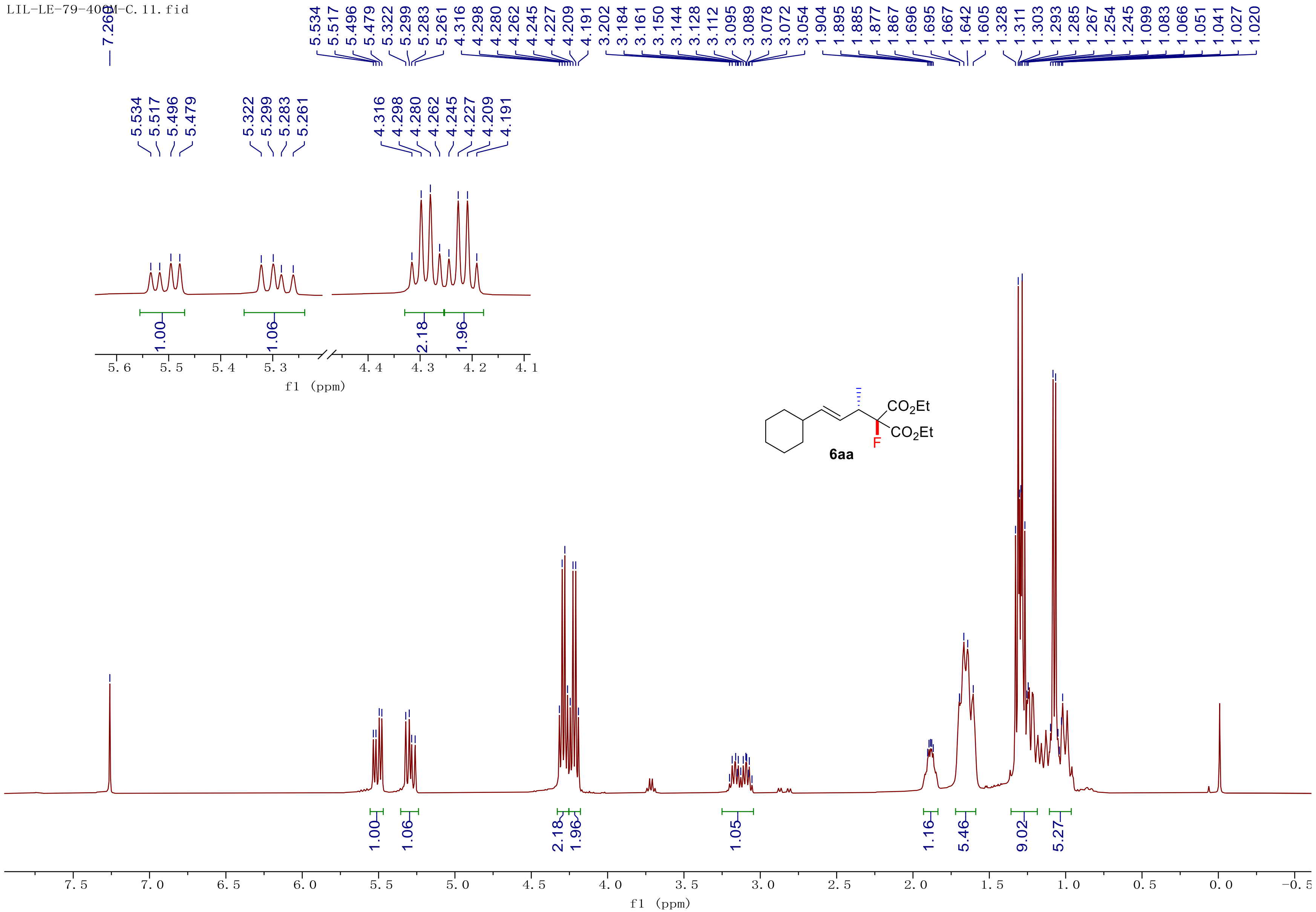
LIL-LE-79-400M-C.11.fid
7.260
5.534
5.517
5.496
5.479
5.322
5.299
5.283
5.261
4.316
4.298
4.280
4.262
4.245
4.227
4.209
4.191
3.202
3.184
3.161
3.150
3.144
3.128
3.112
3.095
3.089
3.078
3.072
3.054
1.904
1.895
1.885
1.877
1.867
1.696
1.695
1.667
1.642
1.605
1.328
1.311
1.303
1.293
1.285
1.267
1.254
1.245
1.099
1.083
1.066
1.051
1.041
1.027
1.020
CO_2Et
CO_2Et
F
6aa
1.00
1.06
2.18
1.96
1.05
1.16
5.46
9.02
5.27
f1 (ppm)

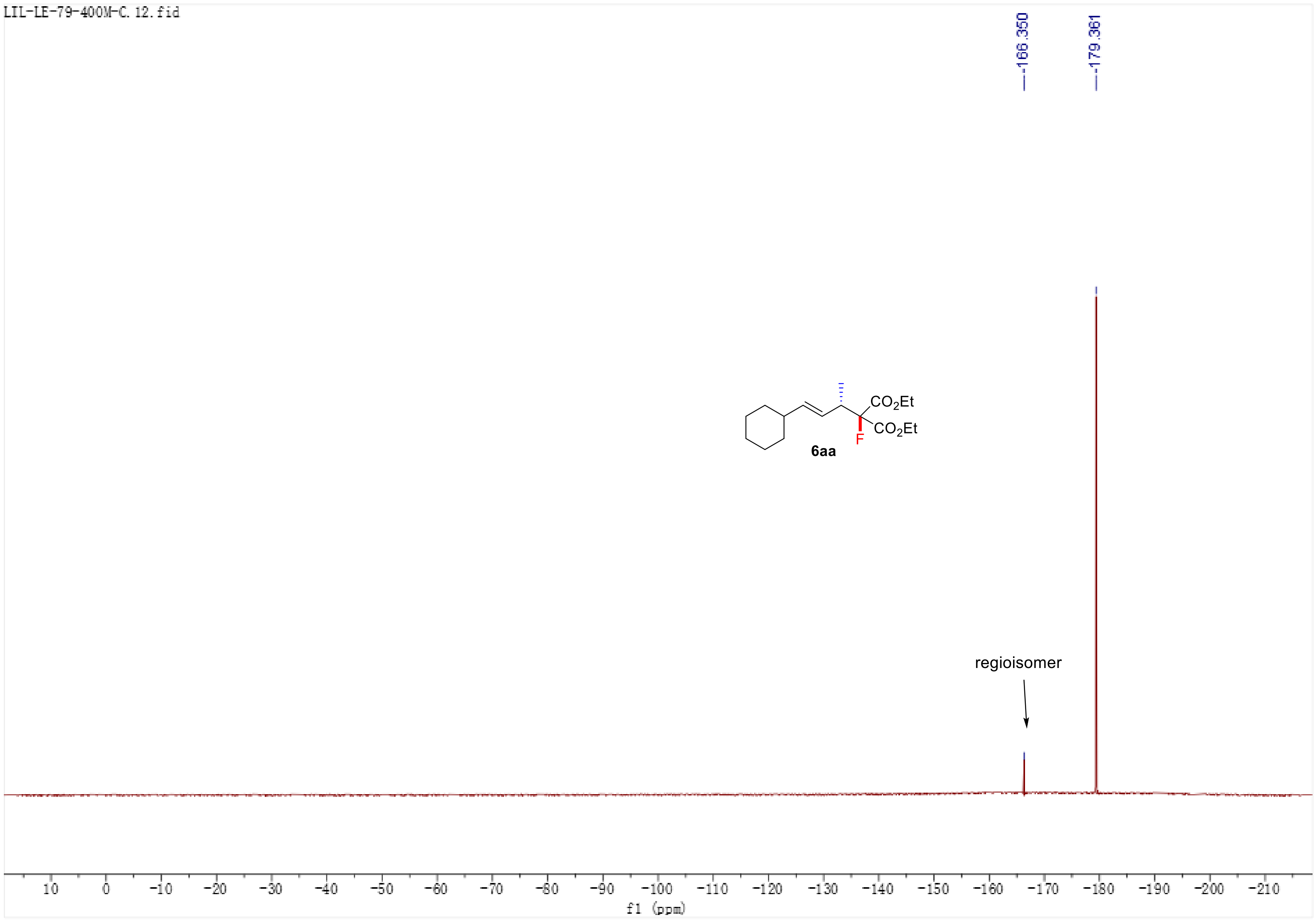
LIL-LE-79-400M-C.12.fid
-166.350
-179.361
CO2Et
CO2Et
F
6aa
regioisomer
10
0
-10
-20
-30
-40
-50
-60
-70
-80
-90
-100
-110
-120
-130
-140
-150
-160
-170
-180
-190
-200
-210
f1 (ppm)

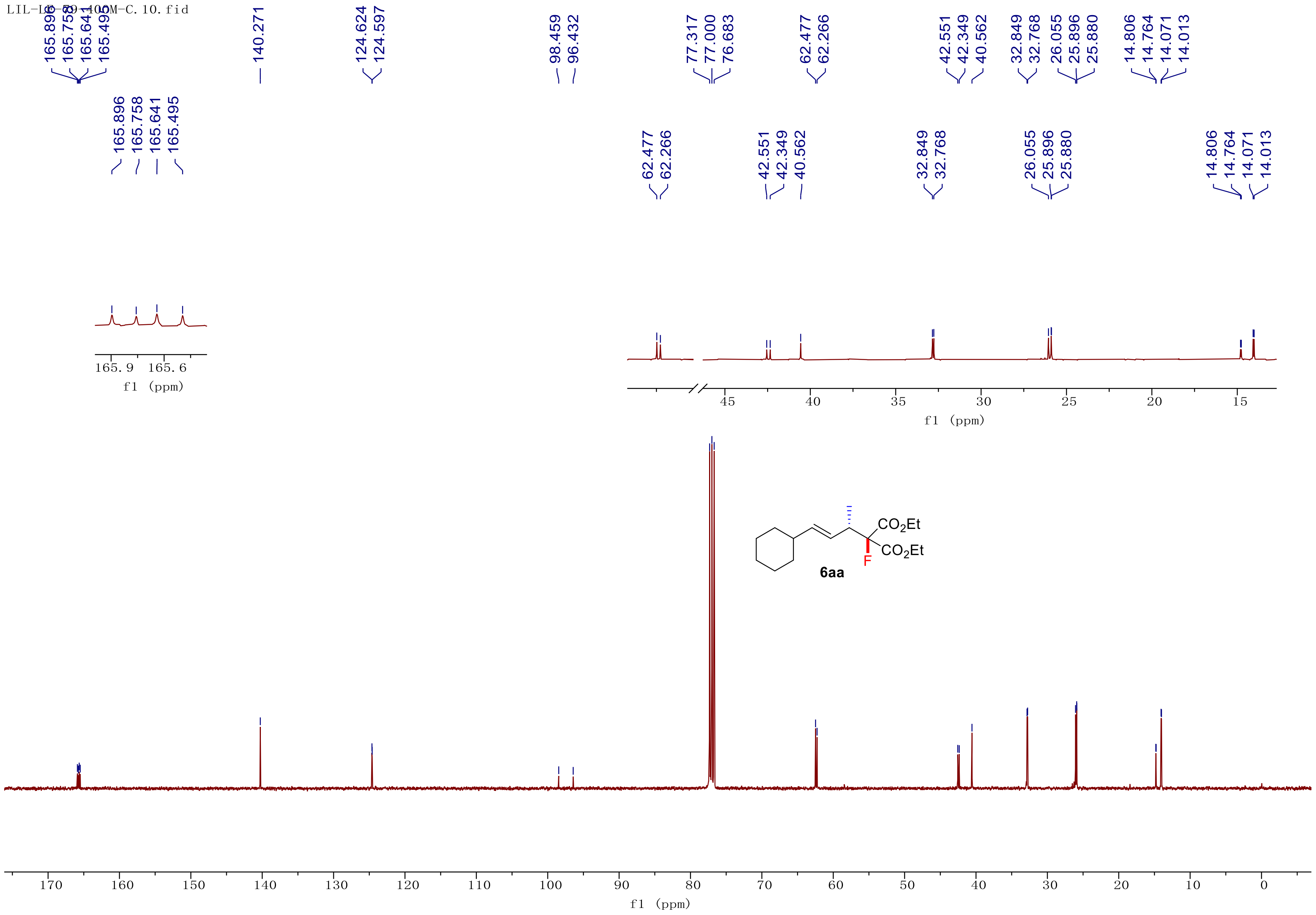
LIL-LL-59-400M-C.10.fid
165.896
165.758
165.641
165.495
140.271
124.624
124.597
98.459
96.432
77.317
77.000
76.683
62.477
62.266
42.551
42.349
40.562
32.849
32.768
26.055
25.896
25.880
14.806
14.764
14.071
14.013
165.9 165.6
f1 (ppm)
45 40 35 30 25 20 15
f1 (ppm)
CO2Et
CO2Et
F
6aa
170 160 150 140 130 120 110 100 90 80 70 60 50 40 30 20 10 0
f1 (ppm)

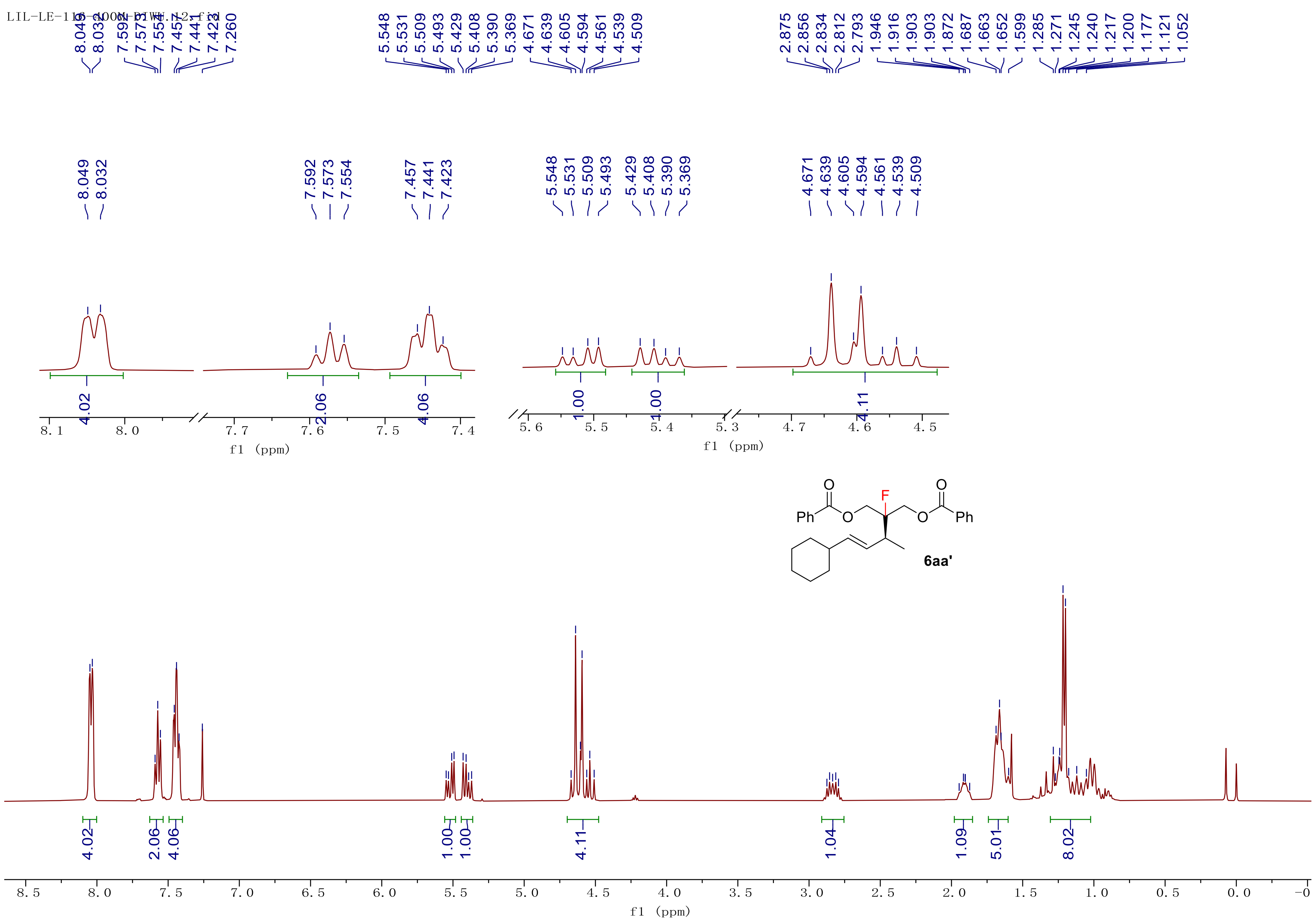

;

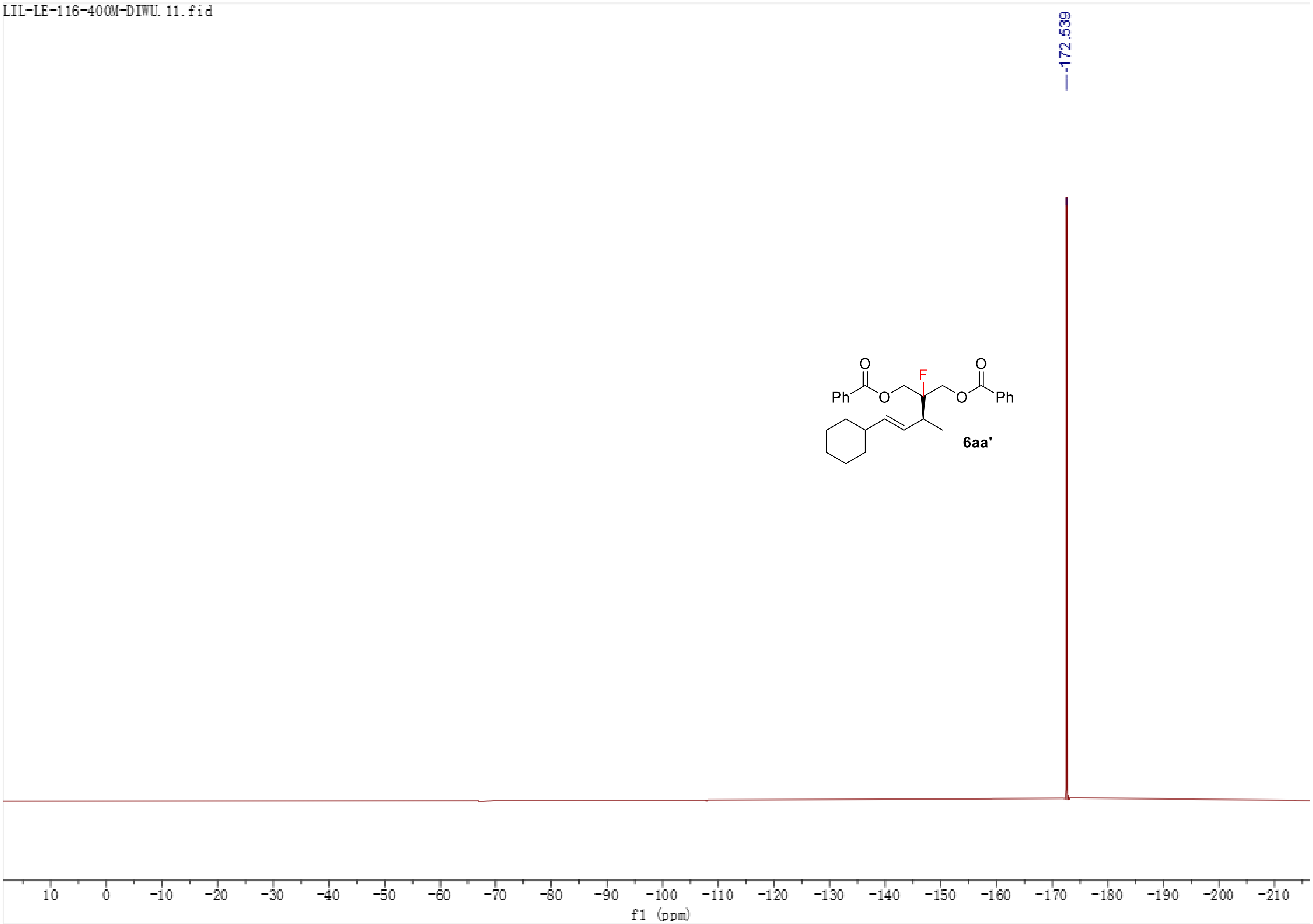

LIL-LE-116-400M-DIWU.11.fid
-172.539
O
F
O
Ph
O
O
Ph
6aa'
10 0 -10 -20 -30 -40 -50 -60 -70 -80 -90 -100 -110 -120 -130 -140 -150 -160 -170 -180 -190 -200 -210
f1 (ppm)

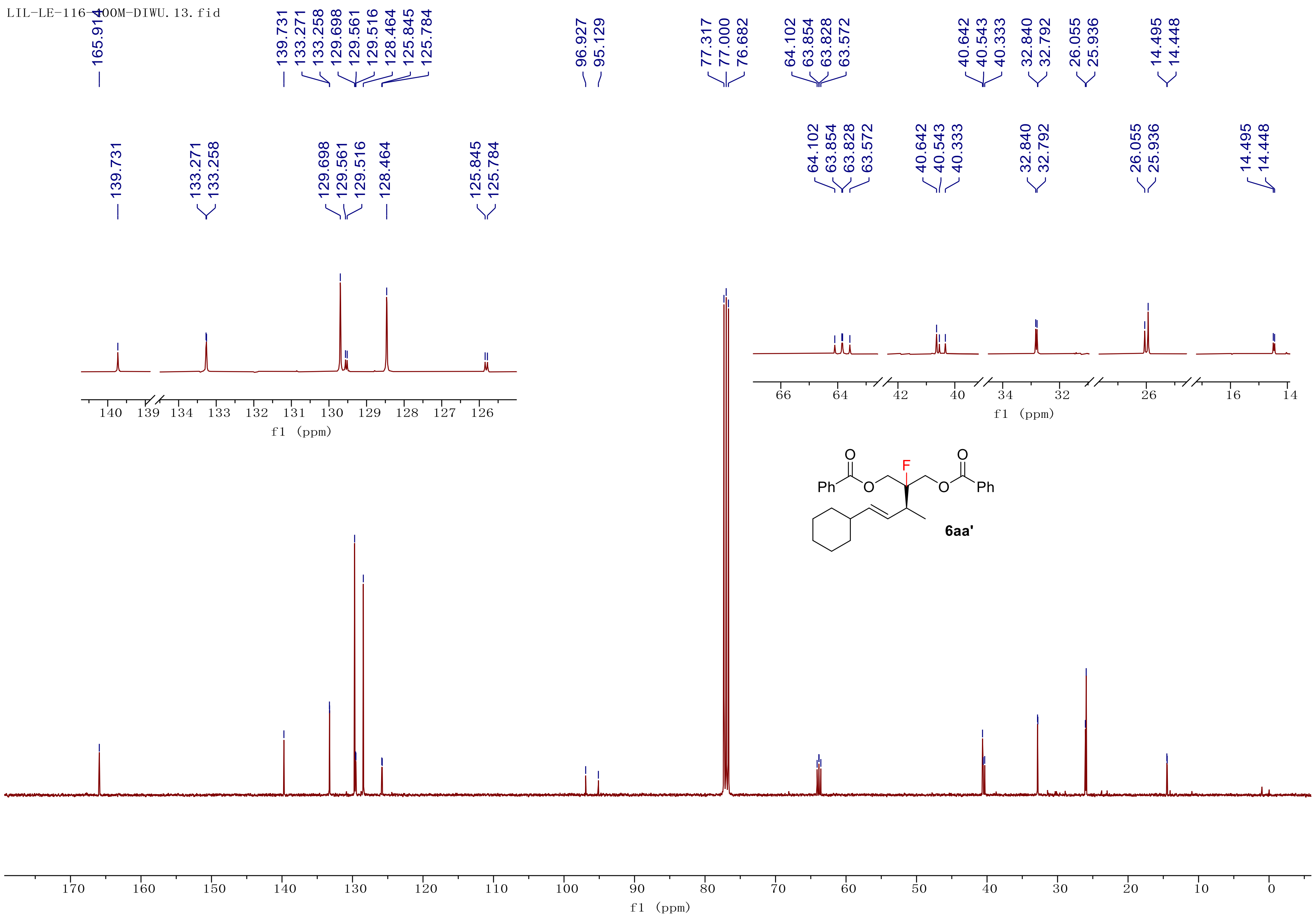
LIL-LE-116-400M-DIWU.13.fid
165.914
139.731
133.271
133.258
129.698
129.561
129.516
128.464
125.845
125.784
96.927
95.129
77.317
77.000
76.682
64.102
63.854
63.828
63.572
40.642
40.543
40.333
32.840
32.792
26.055
25.936
14.495
14.448
f1 (ppm)
6aa'

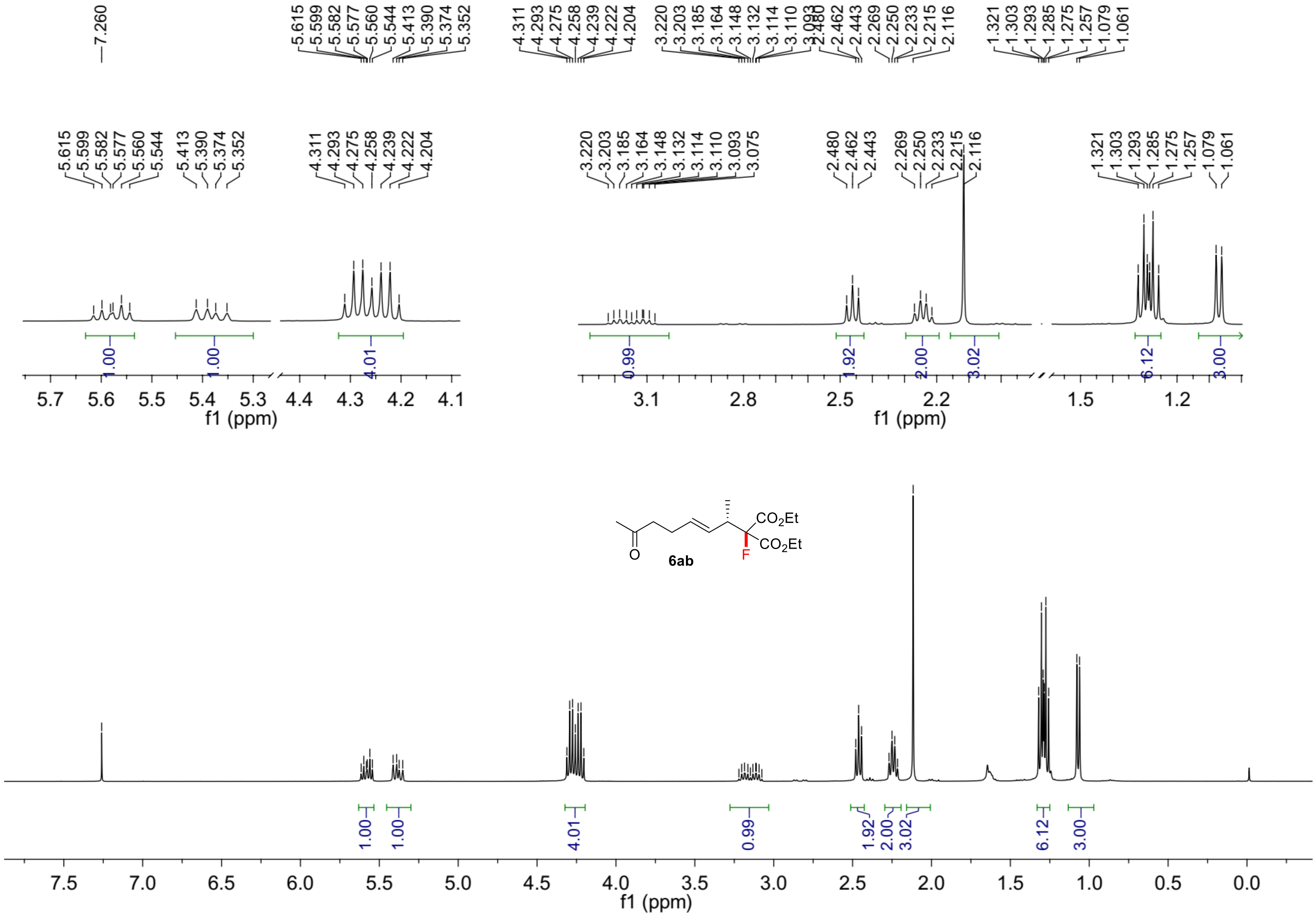
6ab
f1 (ppm)

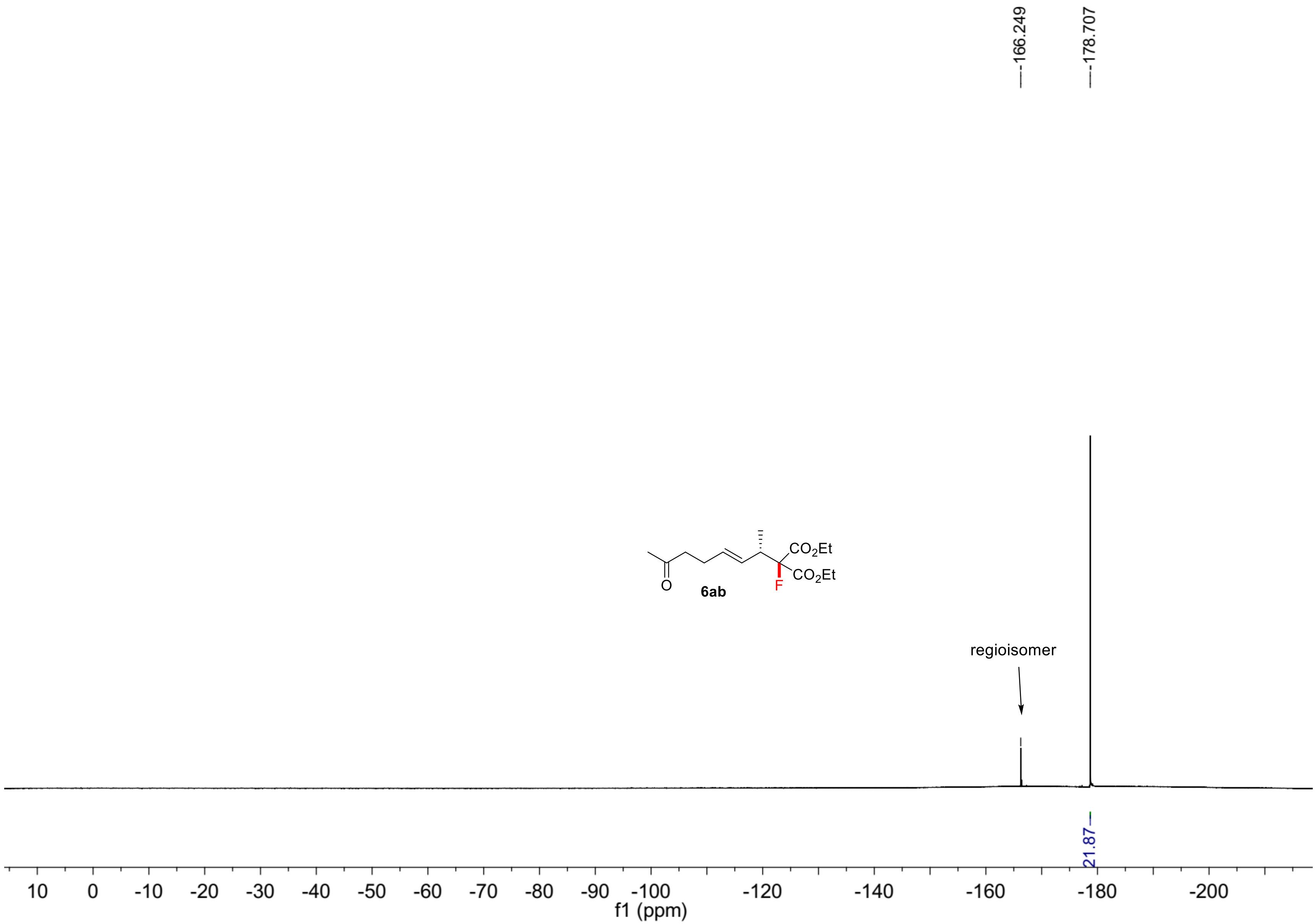

-166.249
-178.707
CO2Et
CO2Et
F
O
6ab
regioisomer
21.87
10 0 -10 -20 -30 -40 -50 -60 -70 -80 -90 -100 -120 -140 -160 -180 -200
f1 (ppm)

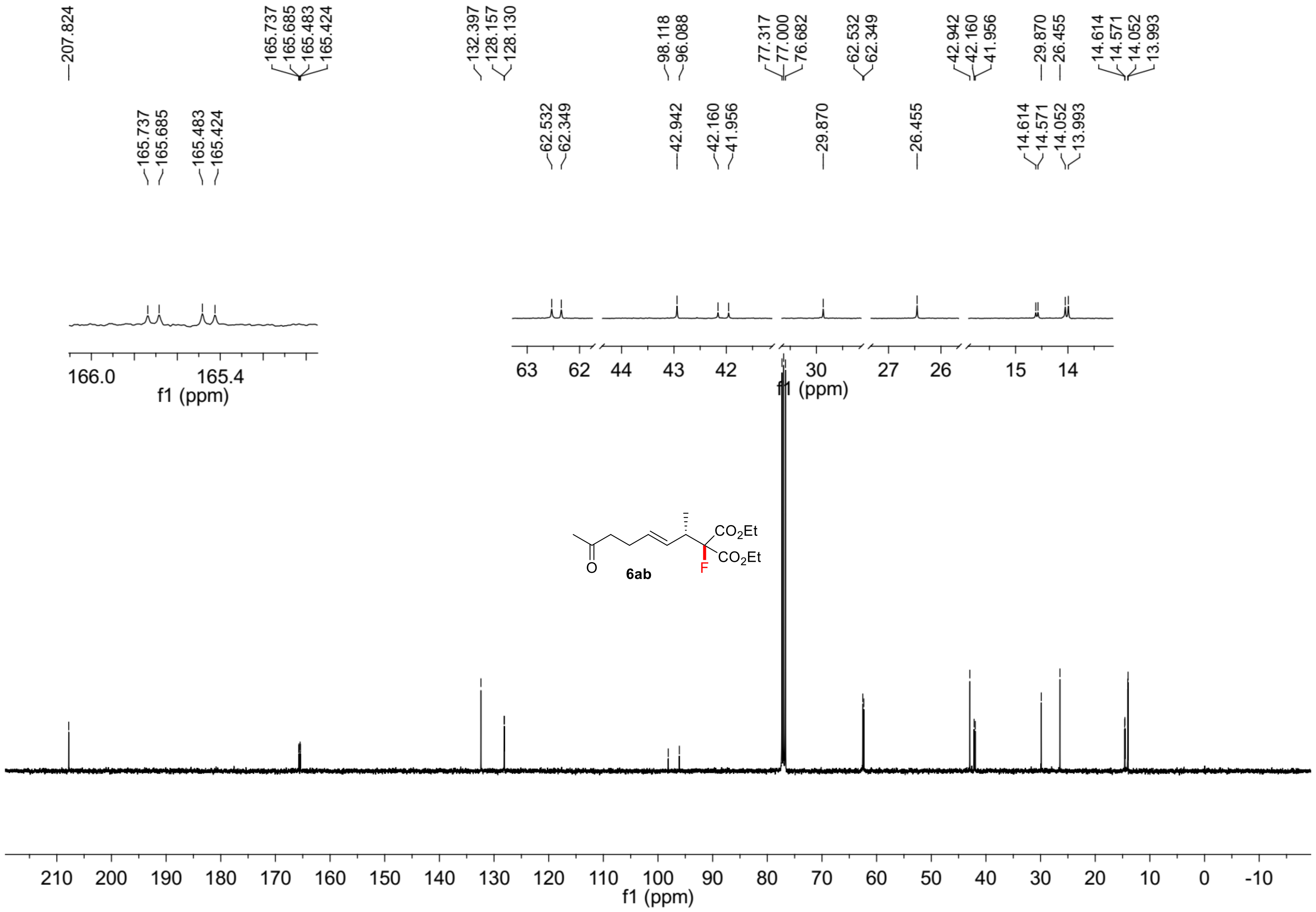

207.824
165.737
165.685
165.483
165.424
132.397
128.157
128.130
98.118
96.088
77.317
77.000
76.682
62.532
62.349
42.942
42.160
41.956
29.870
26.455
14.614
14.571
14.052
13.993
f1 (ppm)
6ab

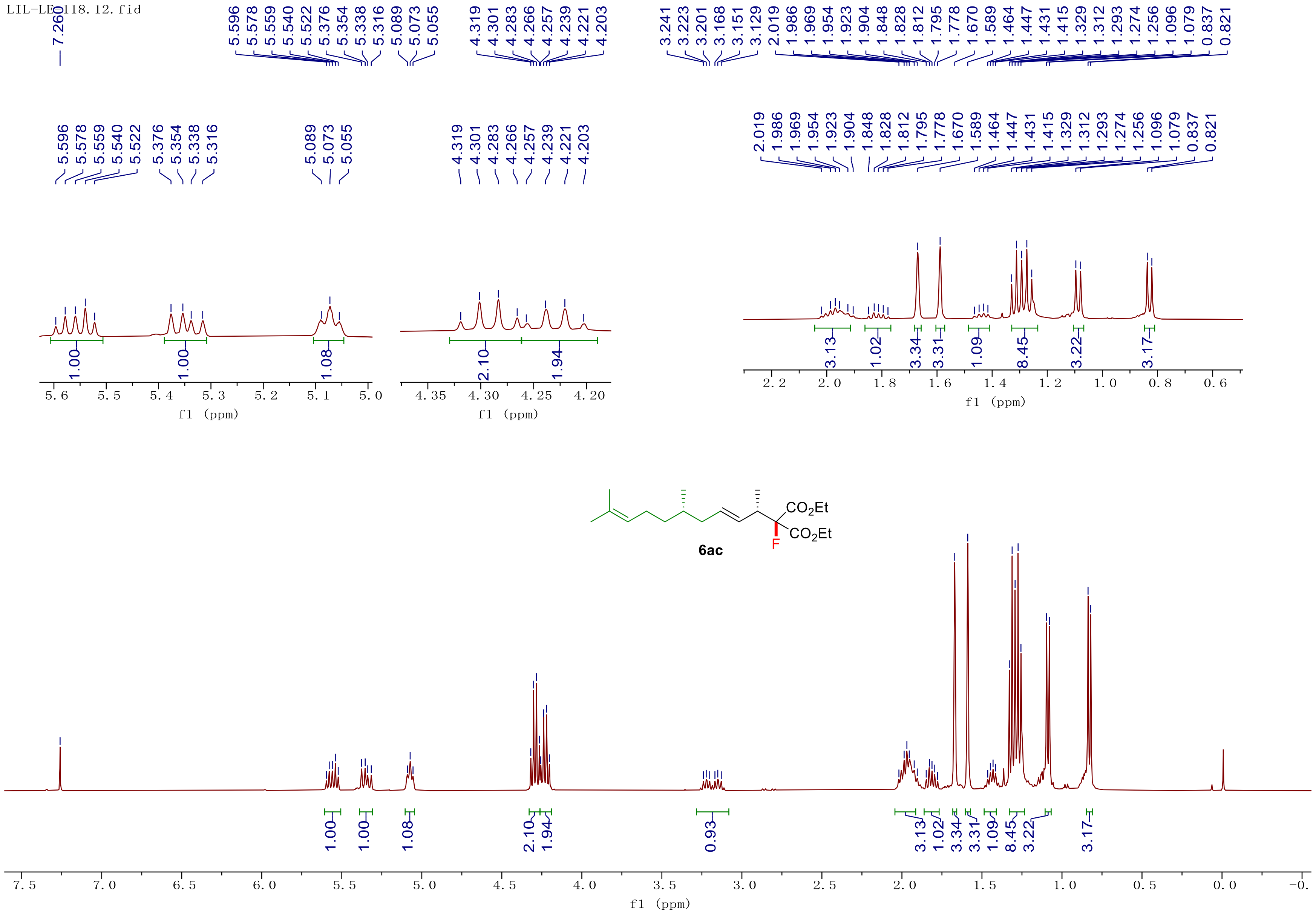

LIL-LE-118.12.fid
CO2Et
CO2Et
F
6ac
f1 (ppm)

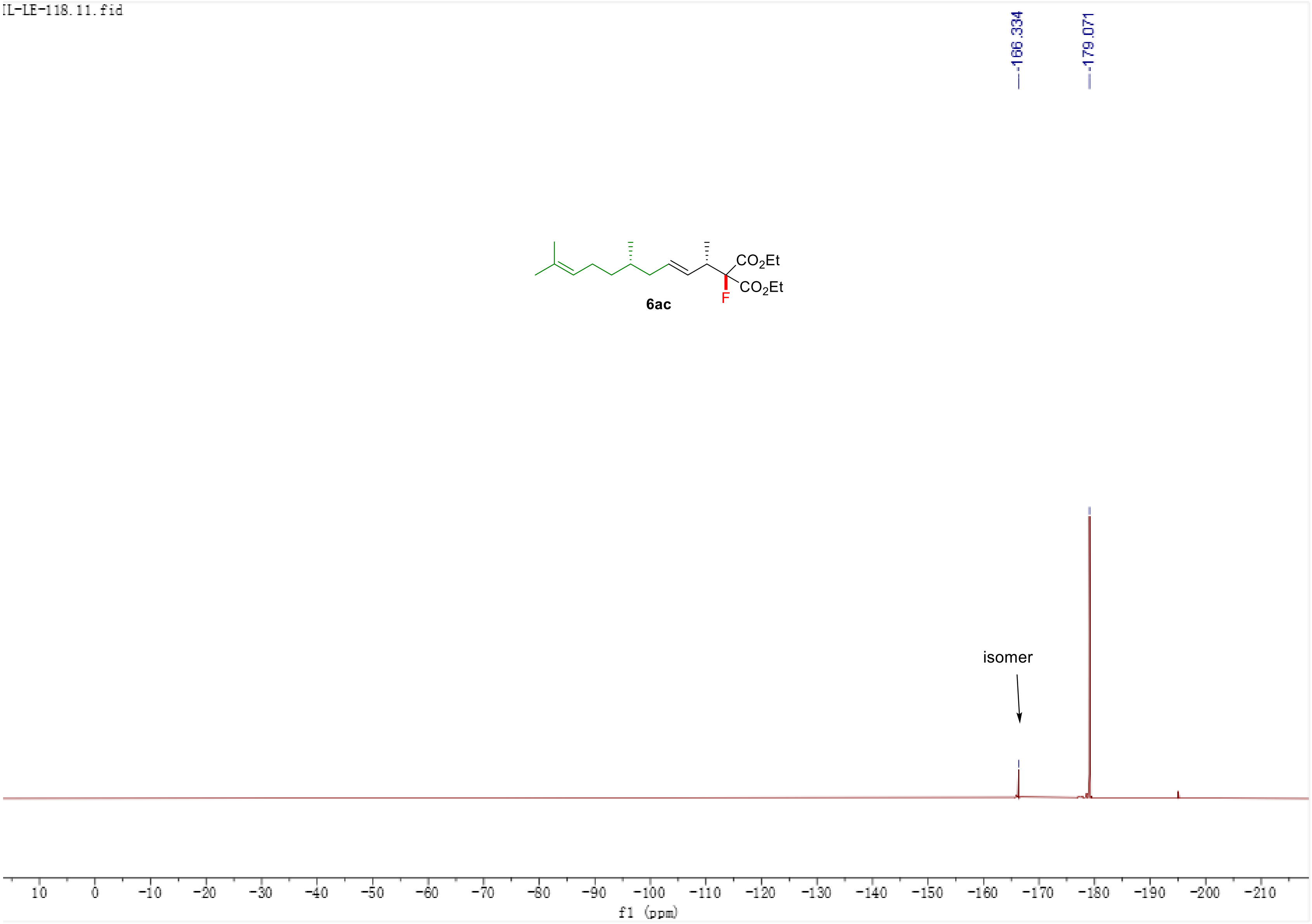

IL-LE-118.11.fid
-166.334
-179.071
CO2Et
CO2Et
F
6ac
isomer
10
0
-10
-20
-30
-40
-50
-60
-70
-80
-90
-100
-110
-120
-130
-140
-150
-160
-170
-180
-190
-200
-210
f1 (ppm)

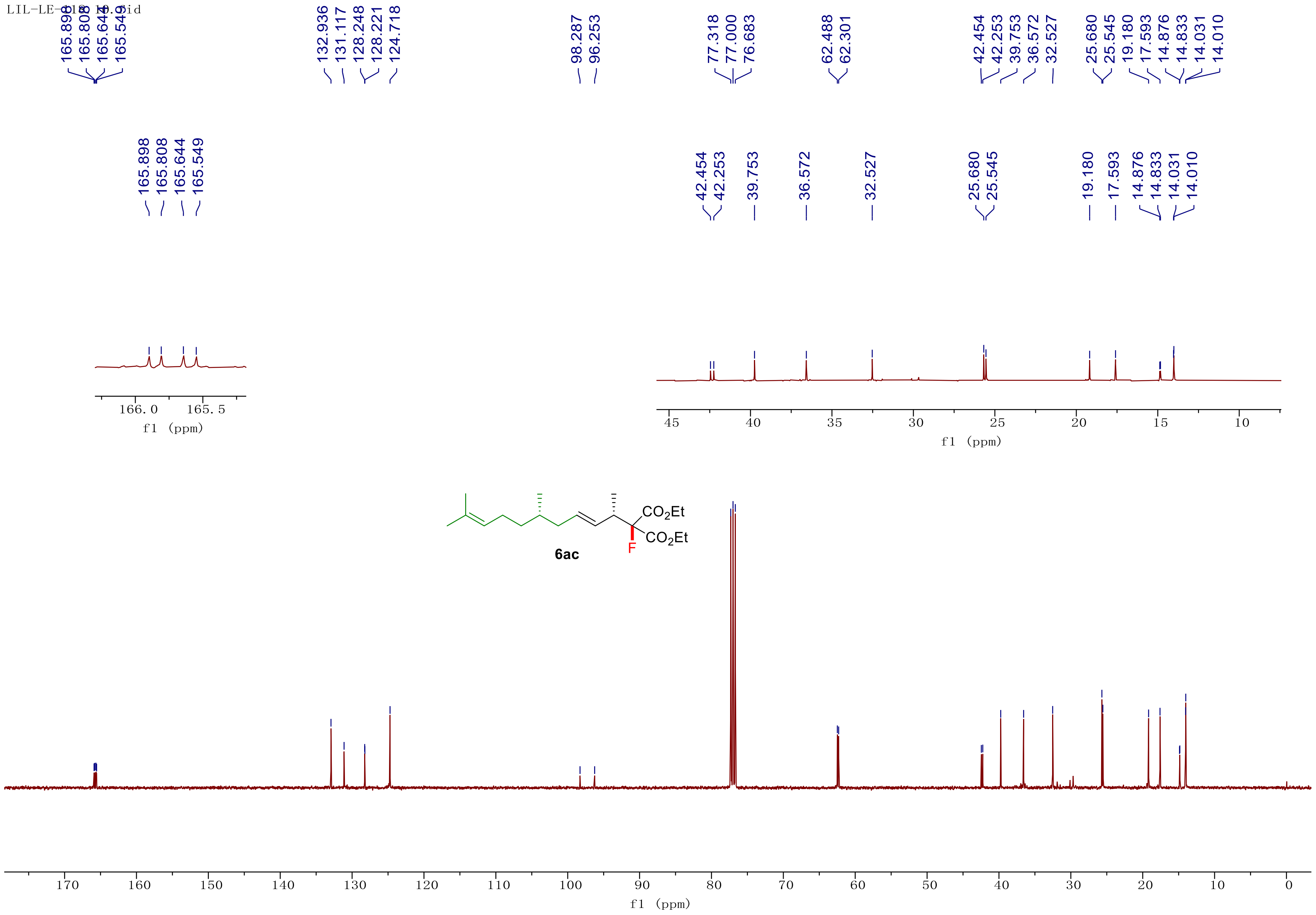
165.898
165.808
165.644
165.549
132.936
131.117
128.248
128.221
124.718
98.287
96.253
77.318
77.000
76.683
62.488
62.301
42.454
42.253
39.753
36.572
32.527
25.680
25.545
19.180
17.593
14.876
14.833
14.031
14.010
166.0
165.5
f1 (ppm)
45
40
35
30
25
20
15
10
f1 (ppm)
CO_2Et
CO_2Et
F
6ac
170
160
150
140
130
120
110
100
90
80
70
60
50
40
30
20
10
0
f1 (ppm)

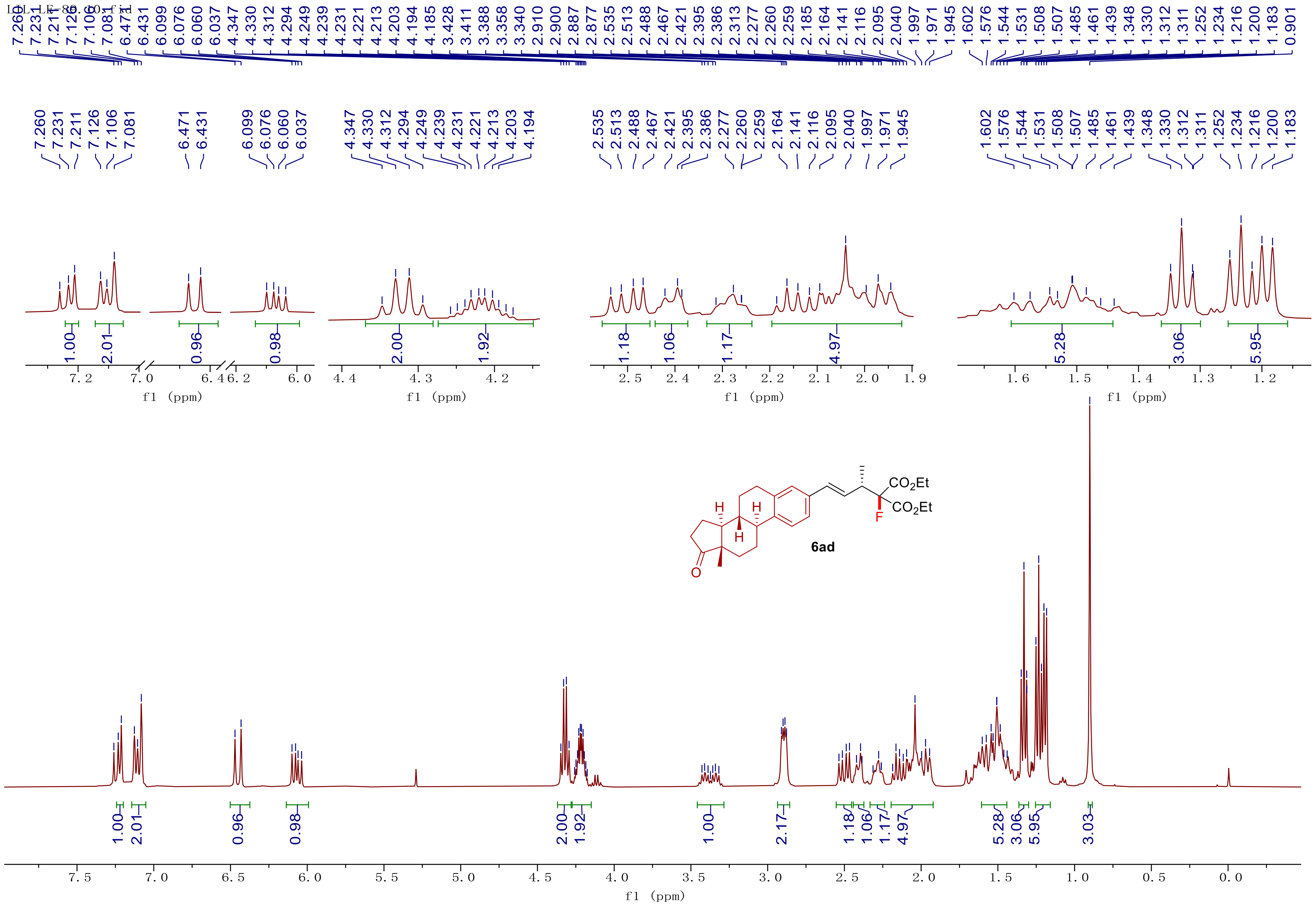
6ad
f1 (ppm)

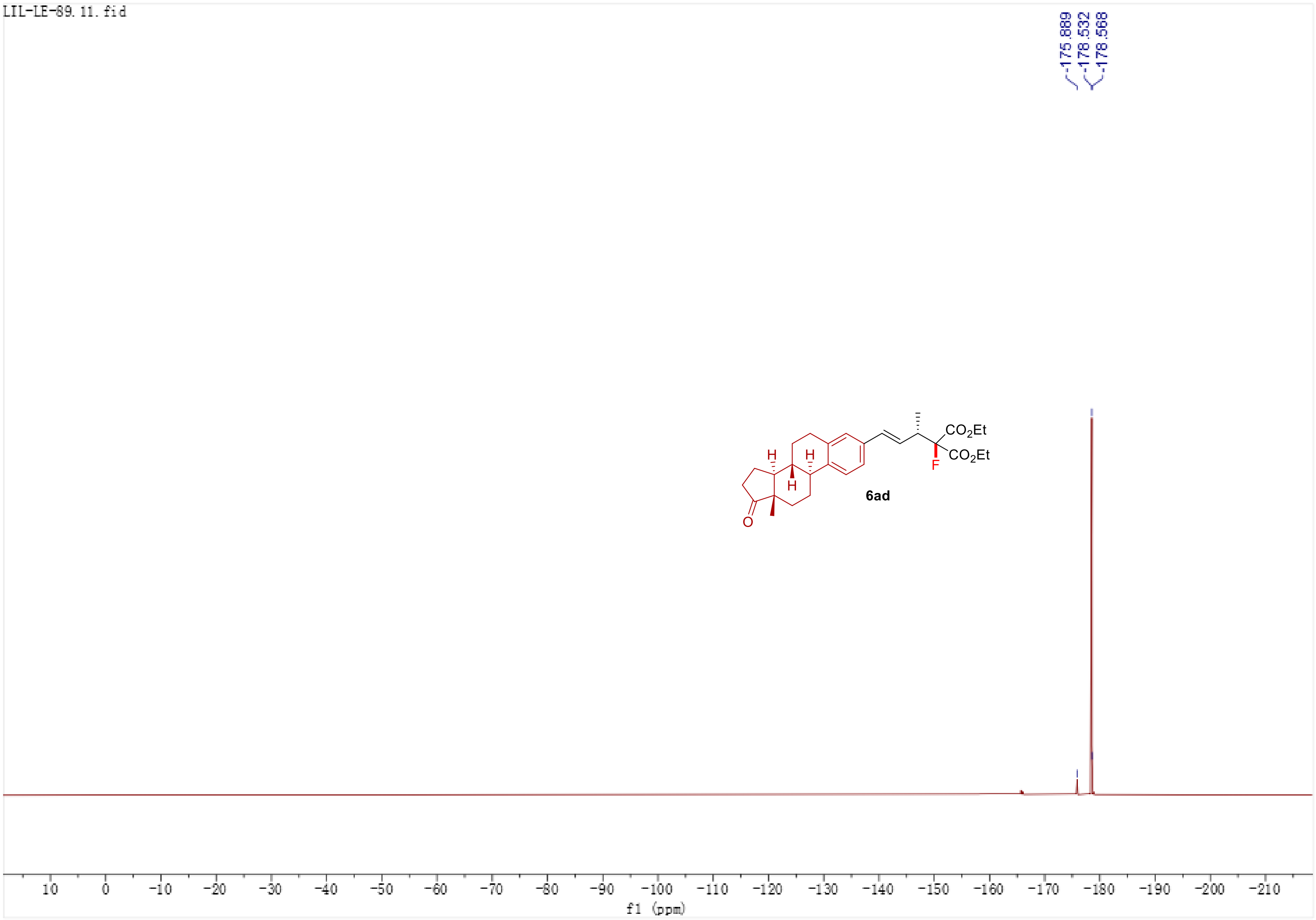
LIL-LE-89.11.fid
-175.889
-178.532
-178.568
CO2Et
CO2Et
F
H
H
H
O
6ad
10 0 −10 −20 −30 −40 −50 −60 −70 −80 −90 −100 −110 −120 −130 −140 −150 −160 −170 −180 −190 −200 −210
f1 (ppm)

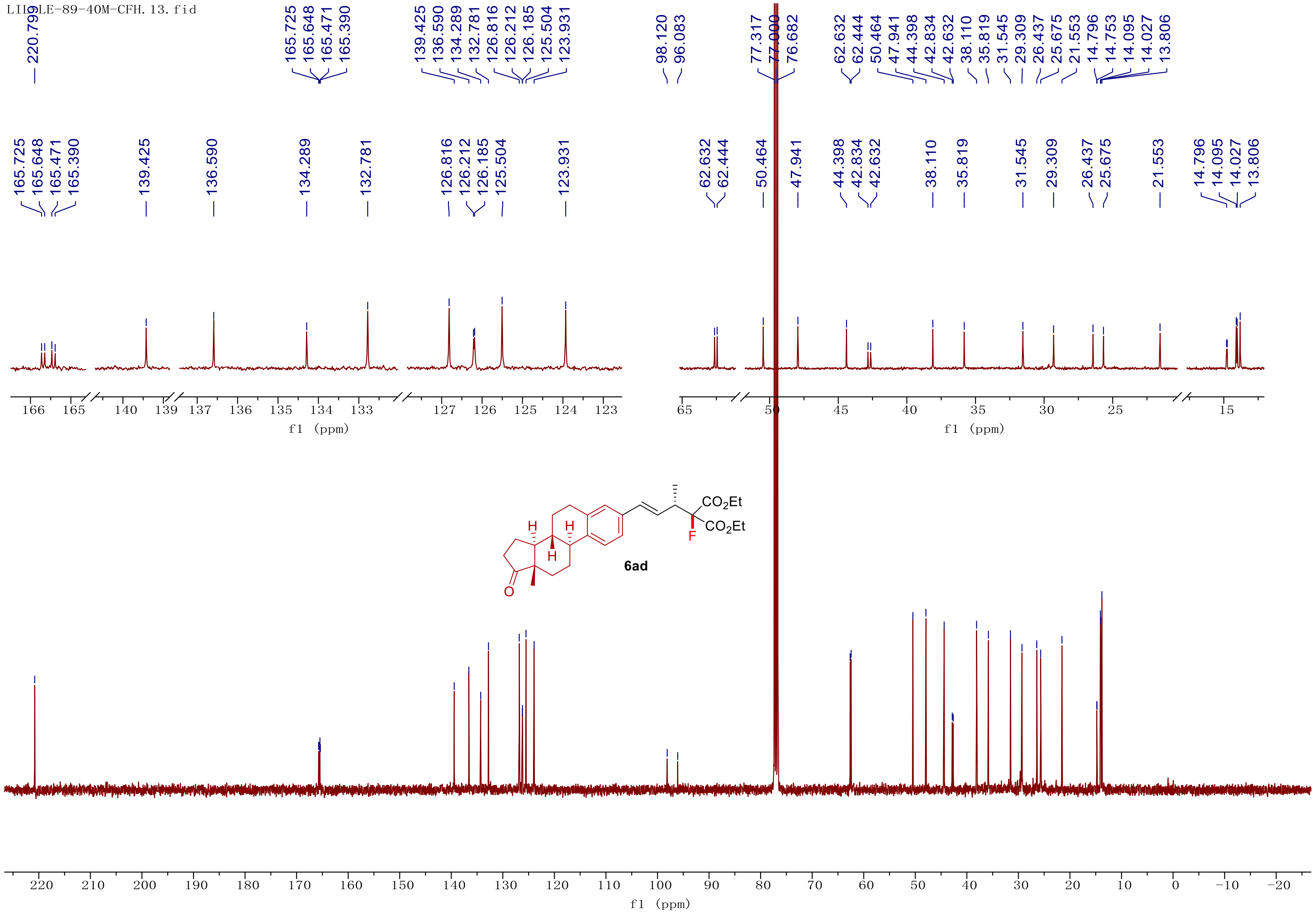
LIDLE-89-40M-CFH.13.fid
220.799
165.725
165.648
165.471
165.390
139.425
136.590
134.289
132.781
126.816
126.212
126.185
125.504
123.931
98.120
96.083
77.317
77.000
76.682
62.632
62.444
50.464
47.941
44.398
42.834
42.632
38.110
35.819
31.545
29.309
26.437
25.675
21.553
14.796
14.753
14.095
14.027
13.806
f1 (ppm)
CO2Et
CO2Et
F
H
H
H
O
6ad

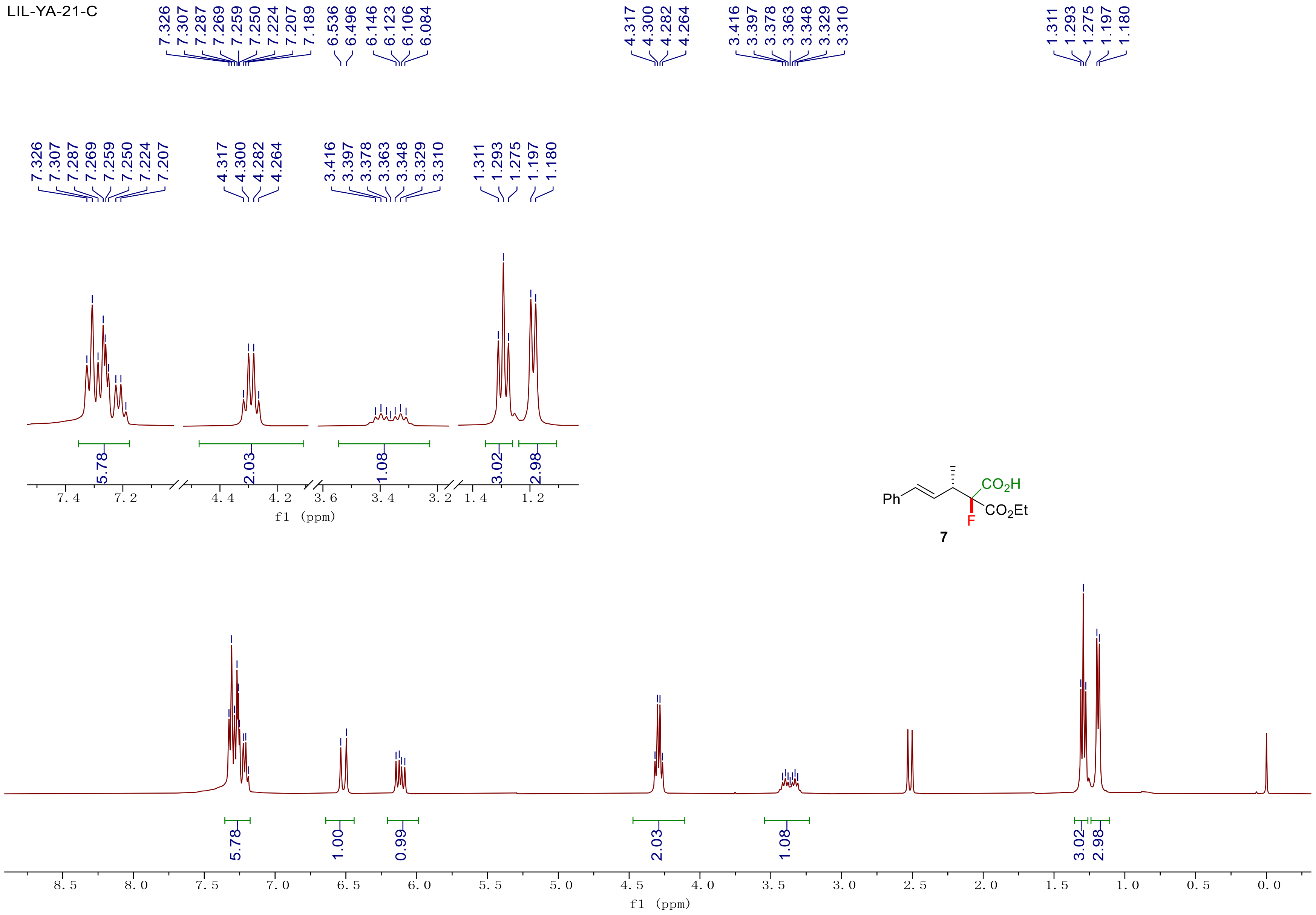
LIL-YA-21-C
7.326
7.307
7.287
7.269
7.259
7.250
7.224
7.207
7.189
6.536
6.496
6.146
6.123
6.106
6.084
4.317
4.300
4.282
4.264
3.416
3.397
3.378
3.363
3.348
3.329
3.310
1.311
1.293
1.275
1.197
1.180
5.78
2.03
1.08
3.02
2.98
1.00
0.99
f1 (ppm)
Ph
CO2H
CO2Et
F
7

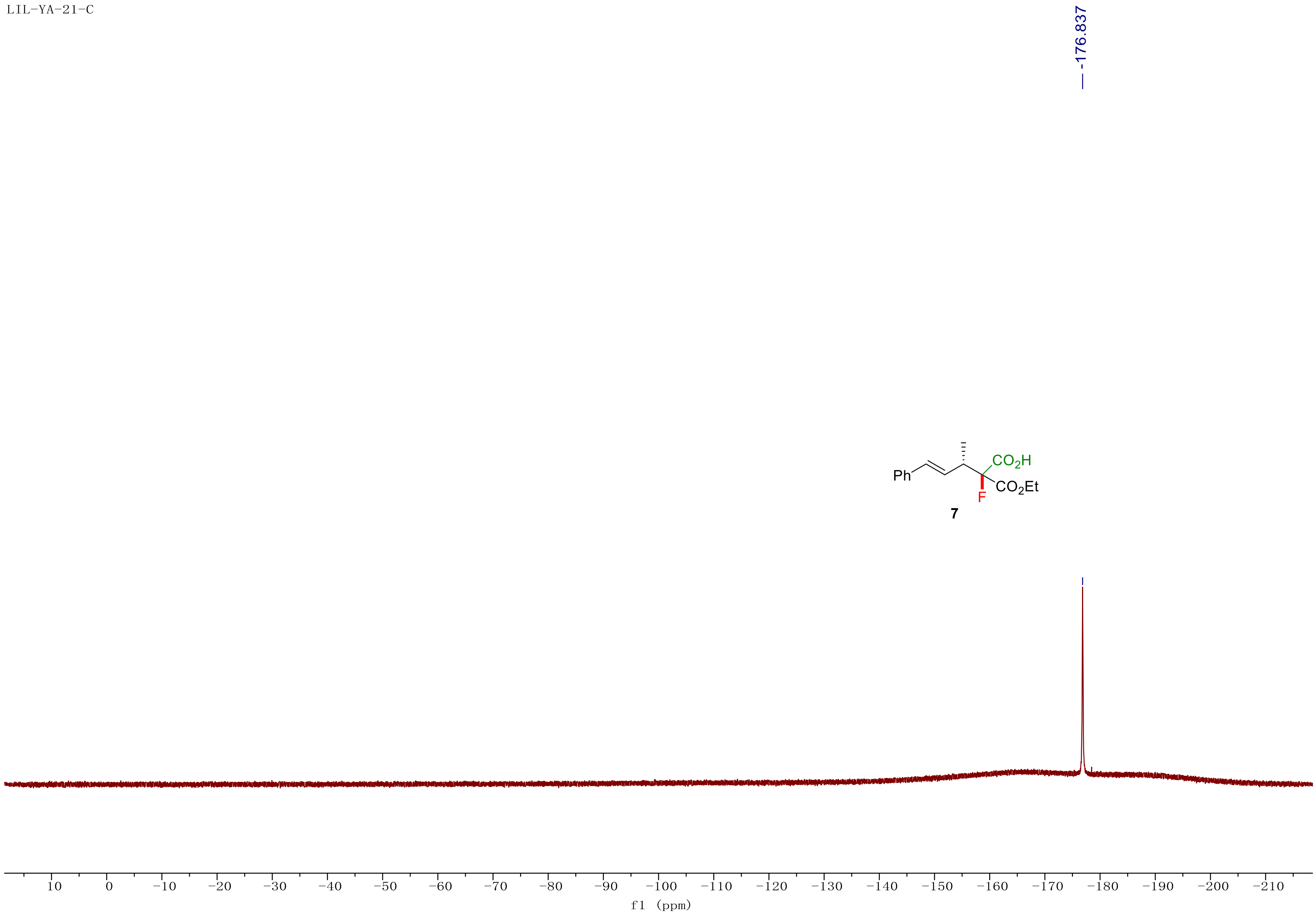
LIL-YA-21-C
-176.837
Ph
CO2H
CO2Et
F
7
10 0 −10 −20 −30 −40 −50 −60 −70 −80 −90 −100 −110 −120 −130 −140 −150 −160 −170 −180 −190 −200 −210
f1 (ppm)

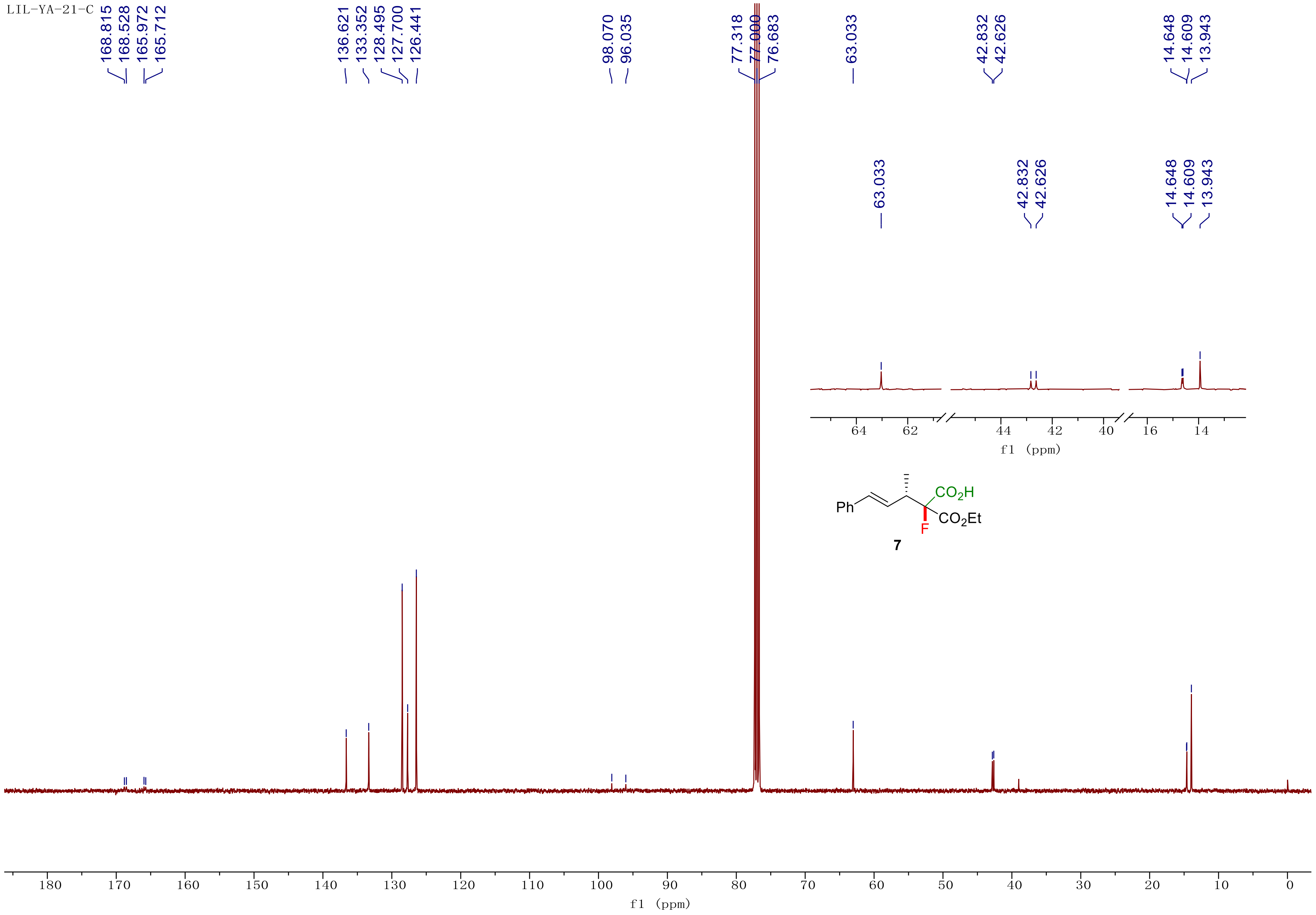
LIL-YA-21-C
168.815
168.528
165.972
165.712
136.621
133.352
128.495
127.700
126.441
98.070
96.035
77.318
77.000
76.683
63.033
42.832
42.626
14.648
14.609
13.943
64
62
44
42
40
16
14
f1 (ppm)
Ph
CO2H
CO2Et
F
7
180
170
160
150
140
130
120
110
100
90
80
70
60
50
40
30
20
10
0
f1 (ppm)

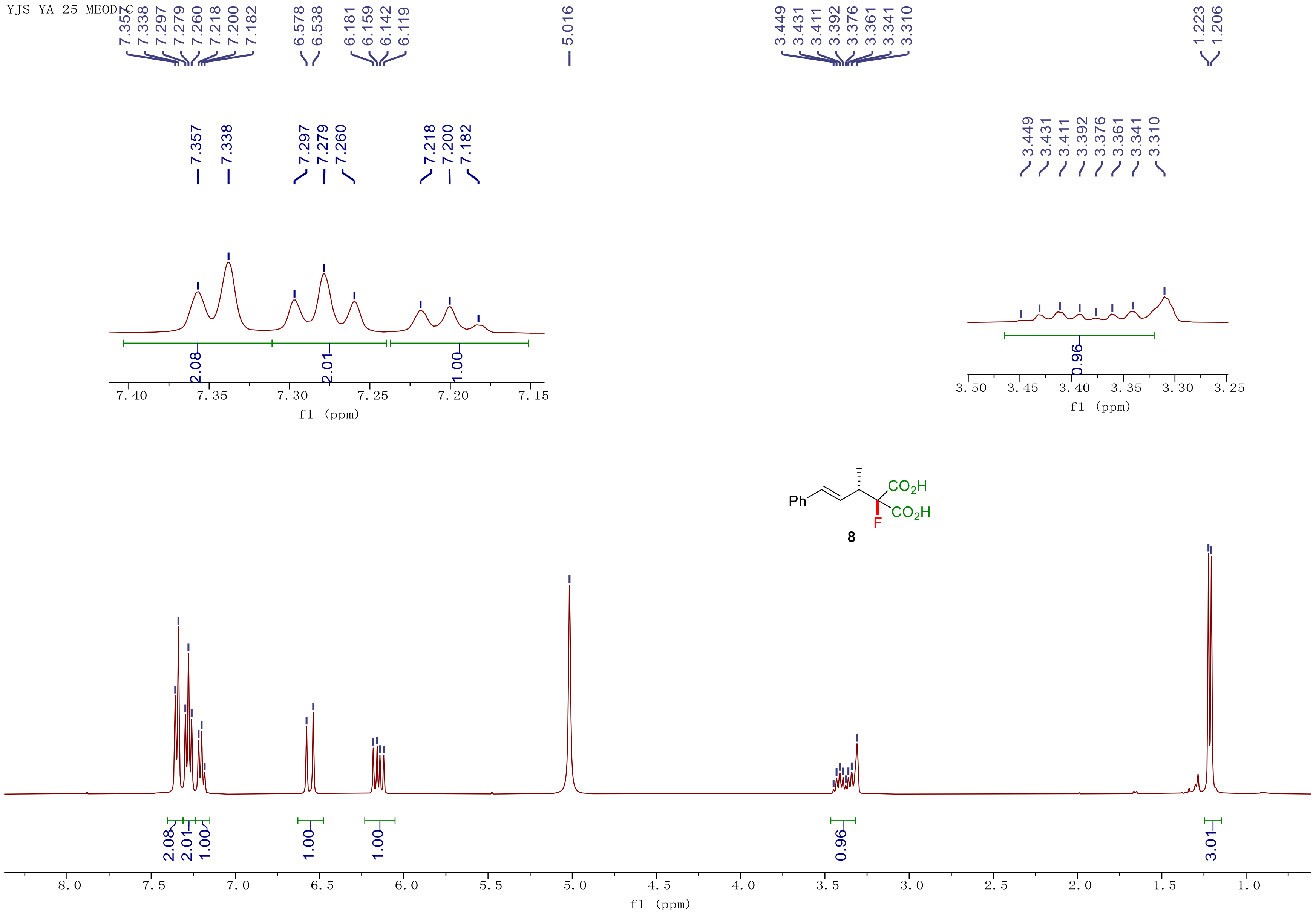
YJS-YA-25-MEOD-C
7.357 7.338 7.297 7.279 7.260 7.218 7.200 7.182
6.578 6.538
6.181 6.159 6.142 6.119
5.016
3.449 3.431 3.411 3.392 3.376 3.361 3.341 3.310
1.223 1.206
2.08 2.01 1.00
0.96
f1 (ppm)
Ph
CO2H
CO2H
F
8
2.08 2.01 1.00 1.00 1.00 0.96 3.01
f1 (ppm)

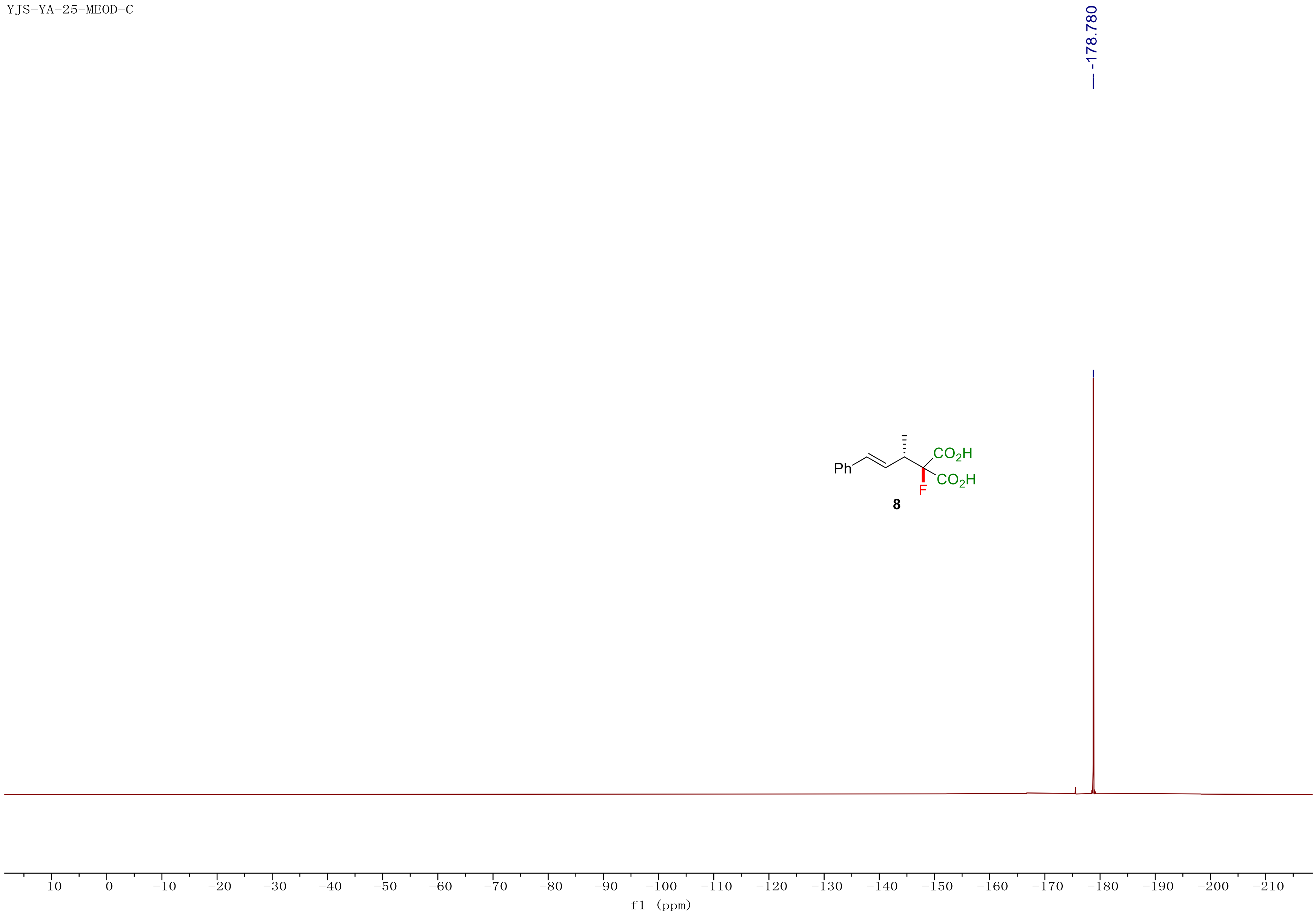
YJS-YA-25-MEOD-C
-178.780
Ph
CO_2H
CO_2H
F
8
10
0
−10
−20
−30
−40
−50
−60
−70
−80
−90
−100
−110
−120
−130
−140
−150
−160
−170
−180
−190
−200
−210
f1 (ppm)

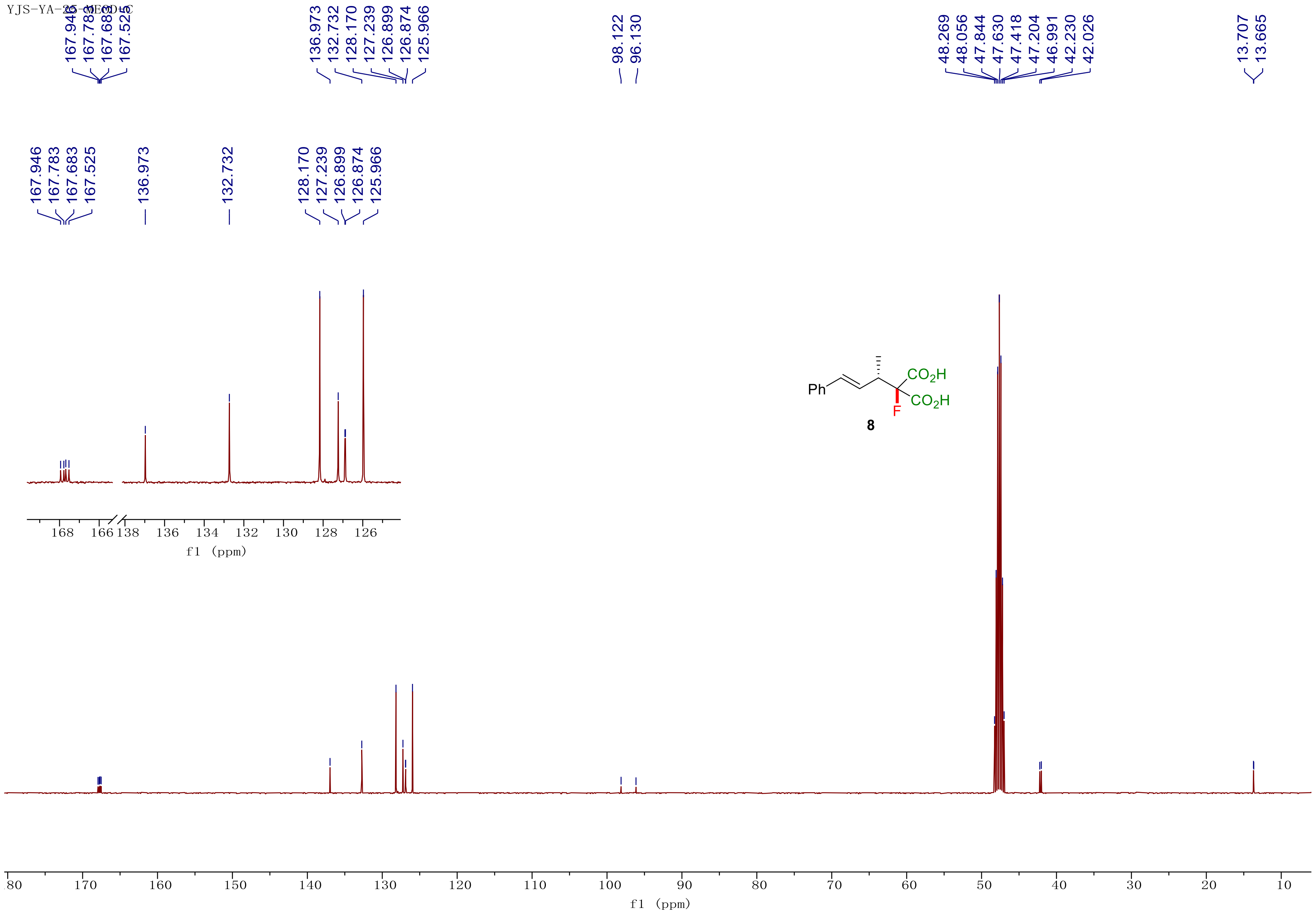
YJS-YA-25-MEOD-C
167.946
167.783
167.683
167.525
136.973
132.732
128.170
127.239
126.899
126.874
125.966
98.122
96.130
48.269
48.056
47.844
47.630
47.418
47.204
46.991
42.230
42.026
13.707
13.665
Ph
CO_2H
CO_2H
F
8
f1 (ppm)

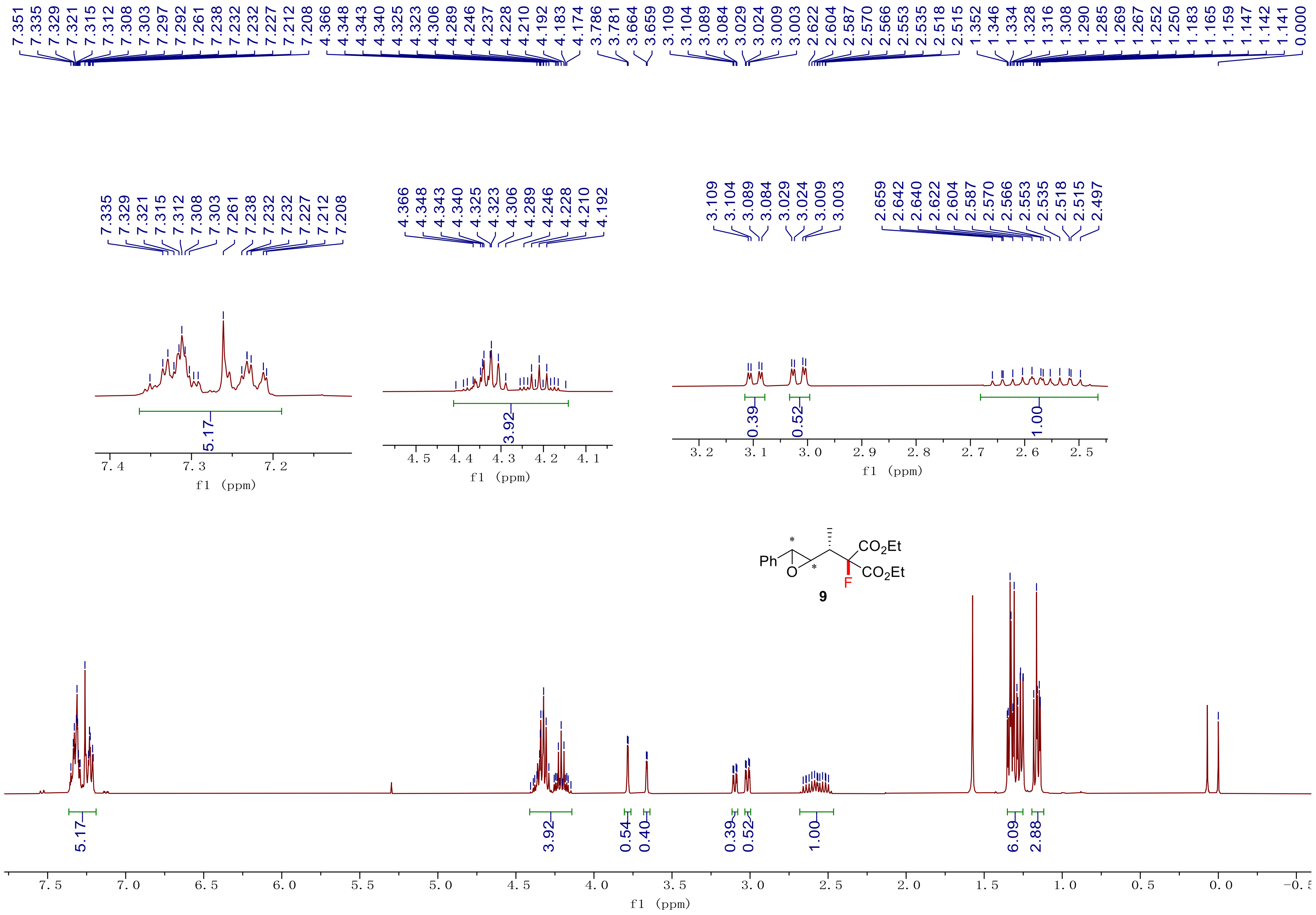

LIL-LE-101-400M-C.21.fid

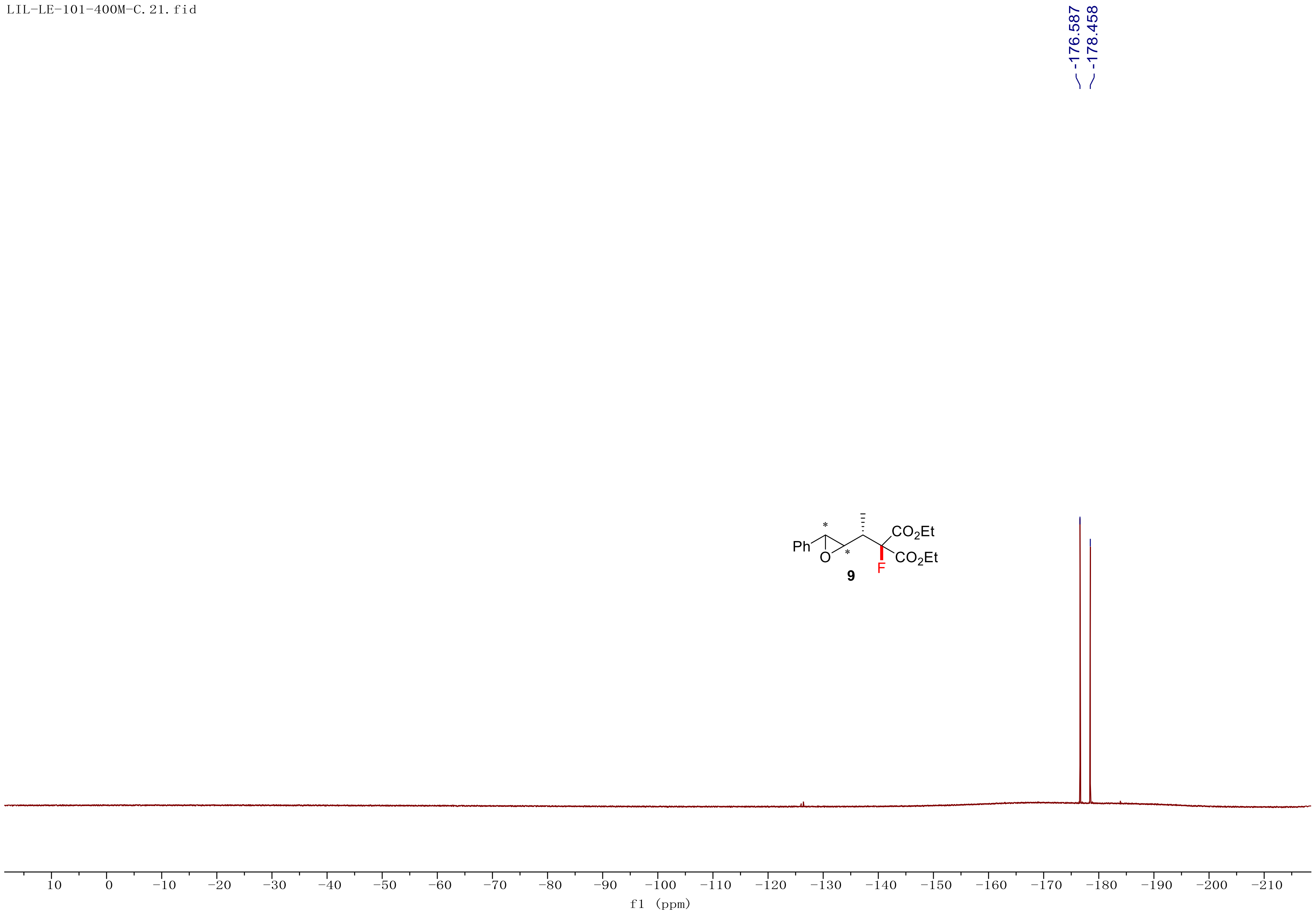

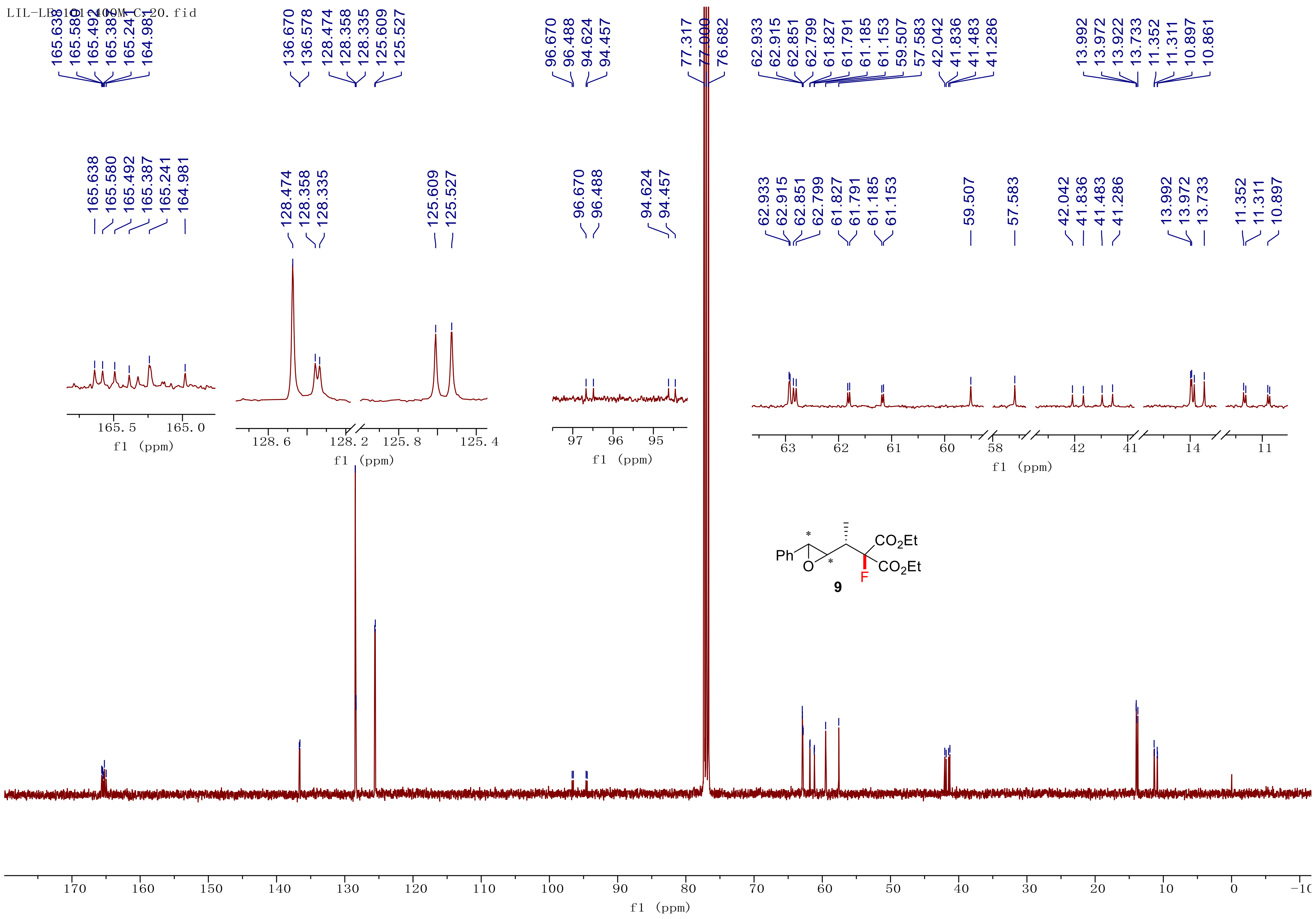
165.638
165.580
165.492
165.387
165.241
164.981
136.670
136.578
128.474
128.358
128.335
125.609
125.527
96.670
96.488
94.624
94.457
77.317
77.000
76.682
62.933
62.915
62.851
62.799
61.827
61.791
61.185
61.153
59.507
57.583
42.042
41.836
41.483
41.286
13.992
13.972
13.922
13.733
11.352
11.311
10.897
10.861
Ph
O
CO2Et
CO2Et
F
9
f1 (ppm)

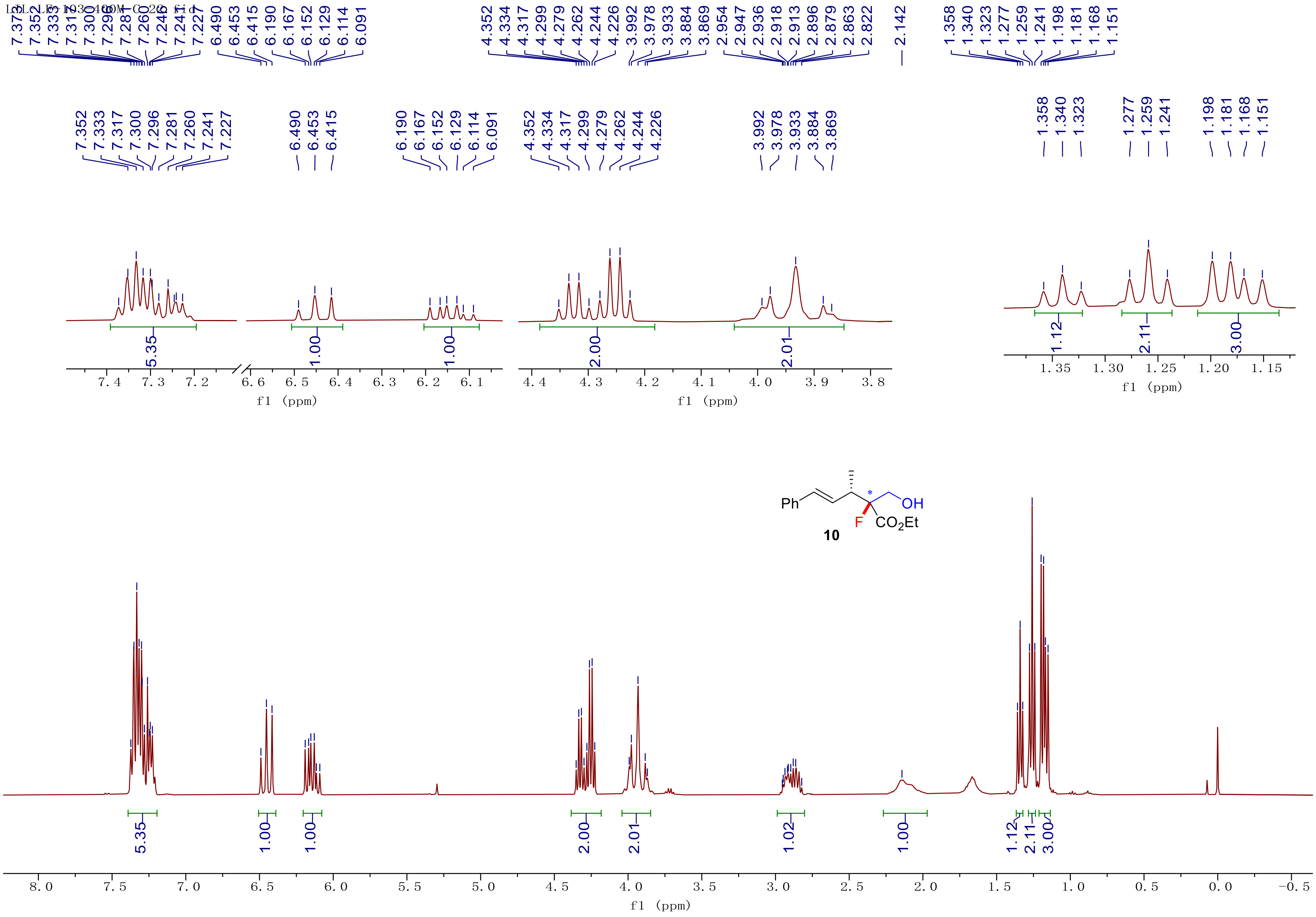
Ph
OH
F
CO2Et
10
f1 (ppm)

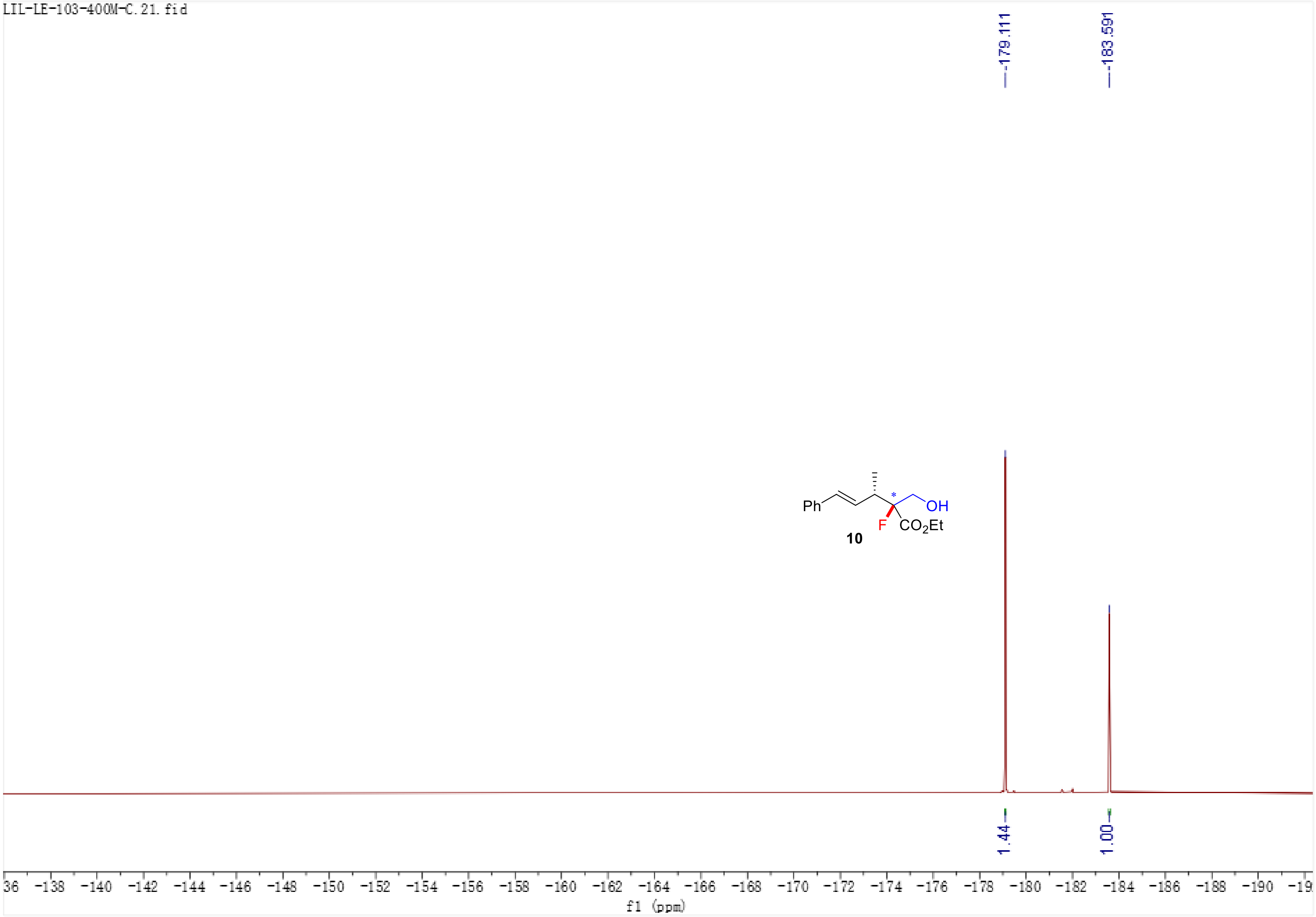
LIL-LE-103-400M-C.21.fid
-179.111
-183.591
Ph
OH
F
CO2Et
10
1.44
1.00
36 -138 -140 -142 -144 -146 -148 -150 -152 -154 -156 -158 -160 -162 -164 -166 -168 -170 -172 -174 -176 -178 -180 -182 -184 -186 -188 -190 -19
f1 (ppm)

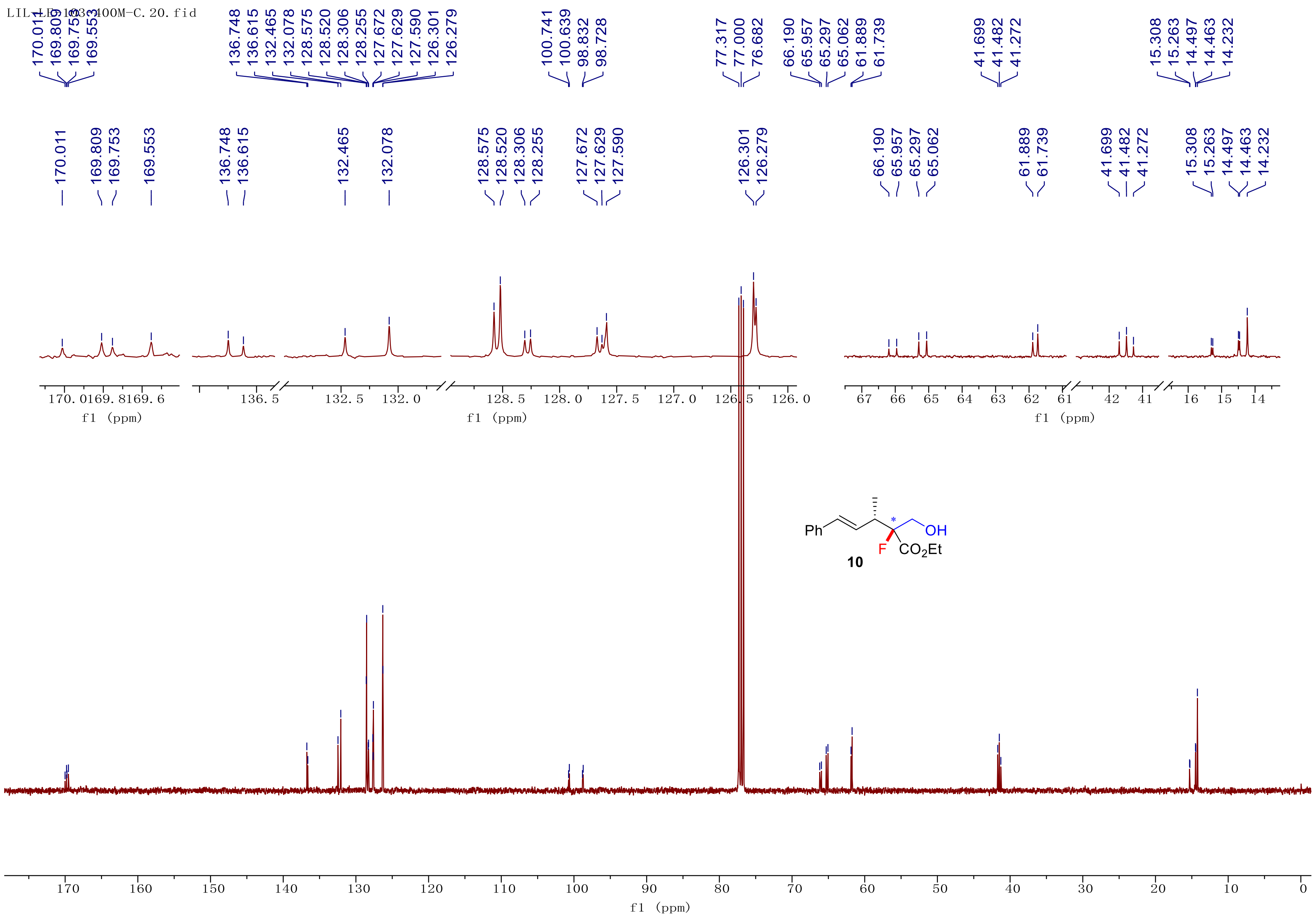
LIL-LB-103-400M-C.20.fid
170.011
169.809
169.753
169.553
136.748
136.615
132.465
132.078
128.575
128.520
128.306
128.255
127.672
127.629
127.590
126.301
126.279
100.741
100.639
98.832
98.728
77.317
77.000
76.682
66.190
65.957
65.297
65.062
61.889
61.739
41.699
41.482
41.272
15.308
15.263
14.497
14.463
14.232
Ph
OH
F
CO_2Et
10
f1 (ppm)

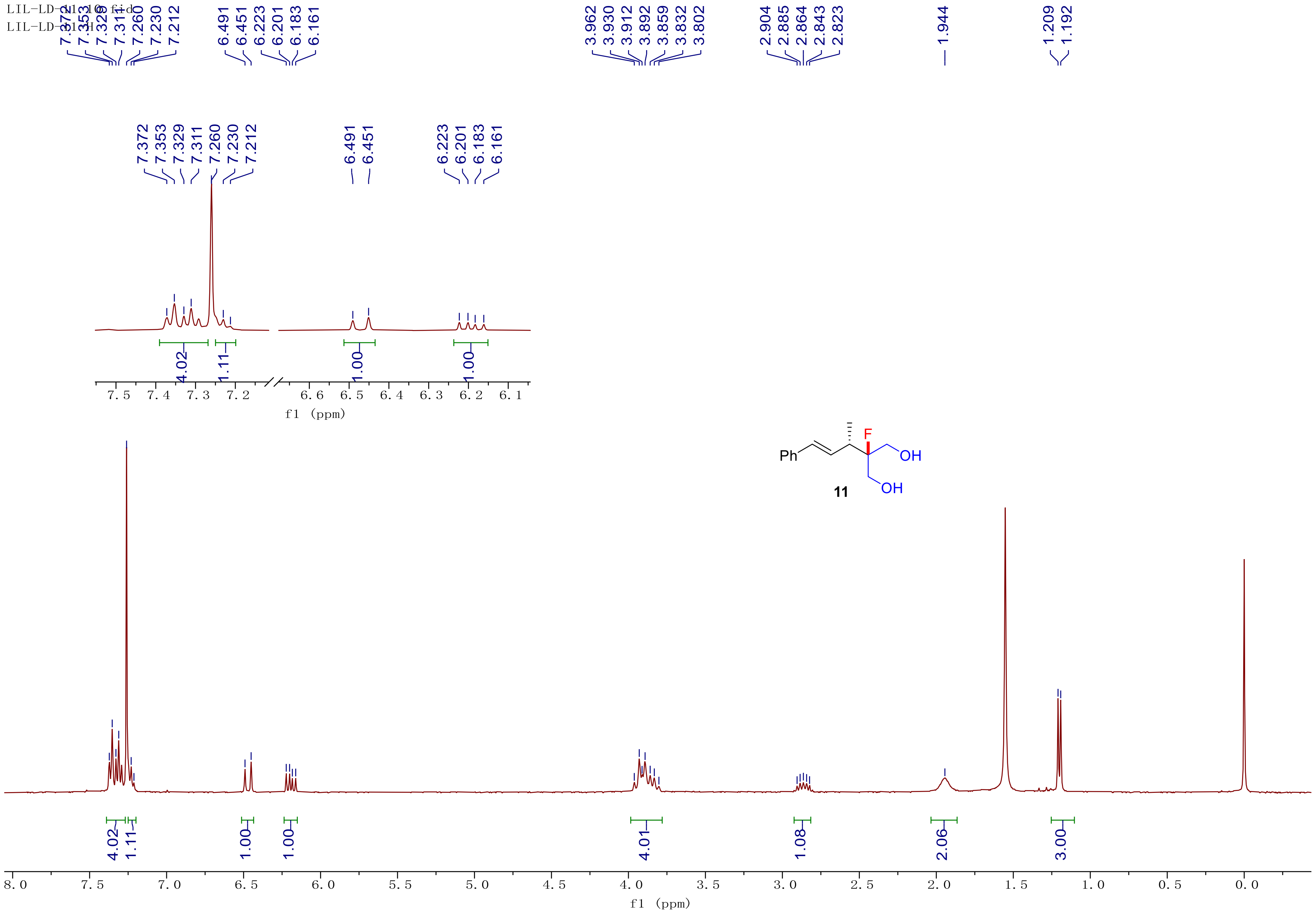
7.372
7.353
7.329
7.311
7.260
7.230
7.212
6.491
6.451
6.223
6.201
6.183
6.161
3.962
3.930
3.912
3.892
3.859
3.832
3.802
2.904
2.885
2.864
2.843
2.823
1.944
1.209
1.192
4.02
1.11
1.00
1.00
4.01
1.08
2.06
3.00
f1 (ppm)
Ph
F
OH
OH
11

YJS-YA-32

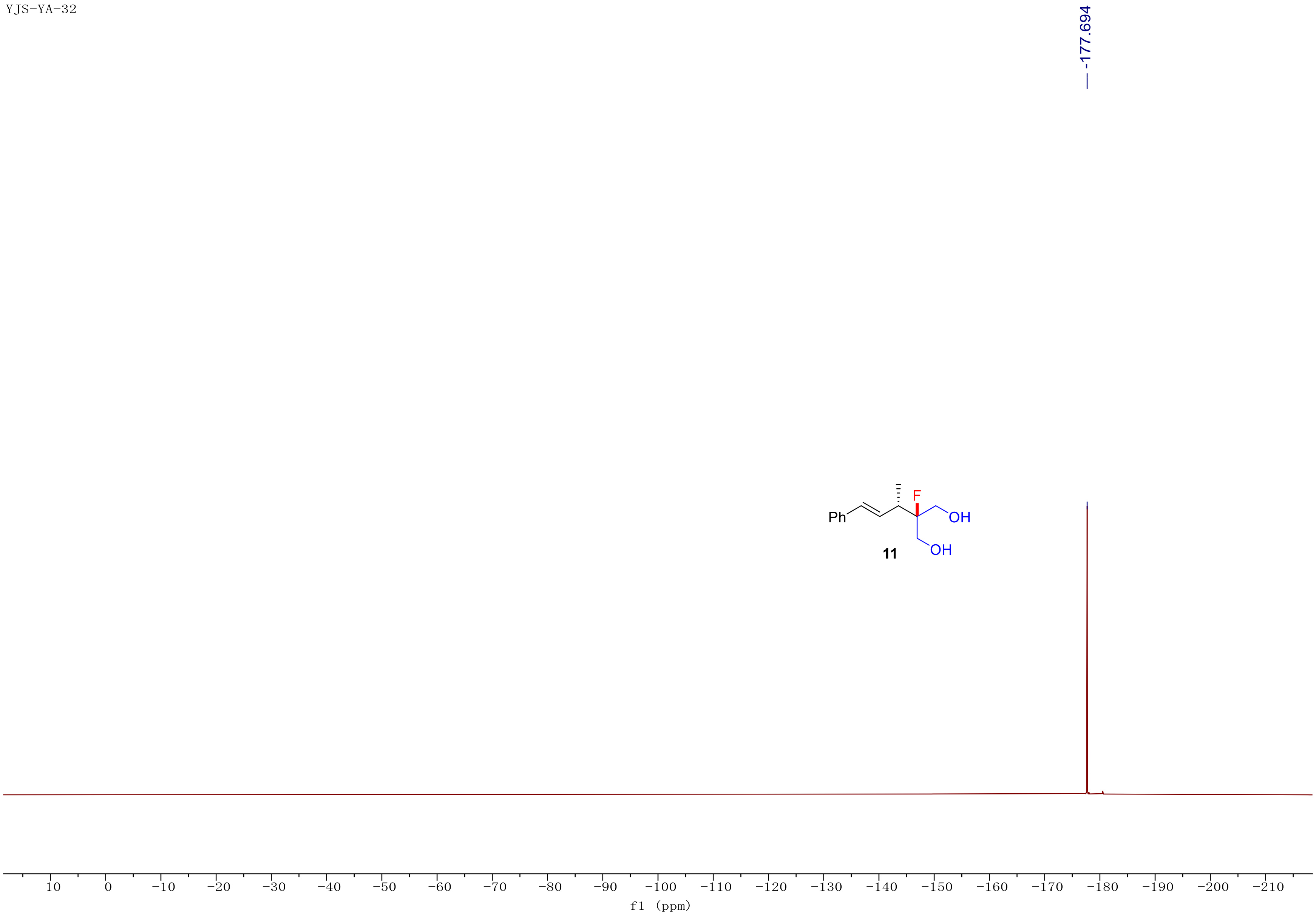
-177.694
Ph
F
OH
OH
10
0
−10
−20
−30
−40
−50
−60
−70
−80
−90
−100
−110
−120
−130
−140
−150
−160
−170
−180
−190
−200
−210
f1 (ppm)

11

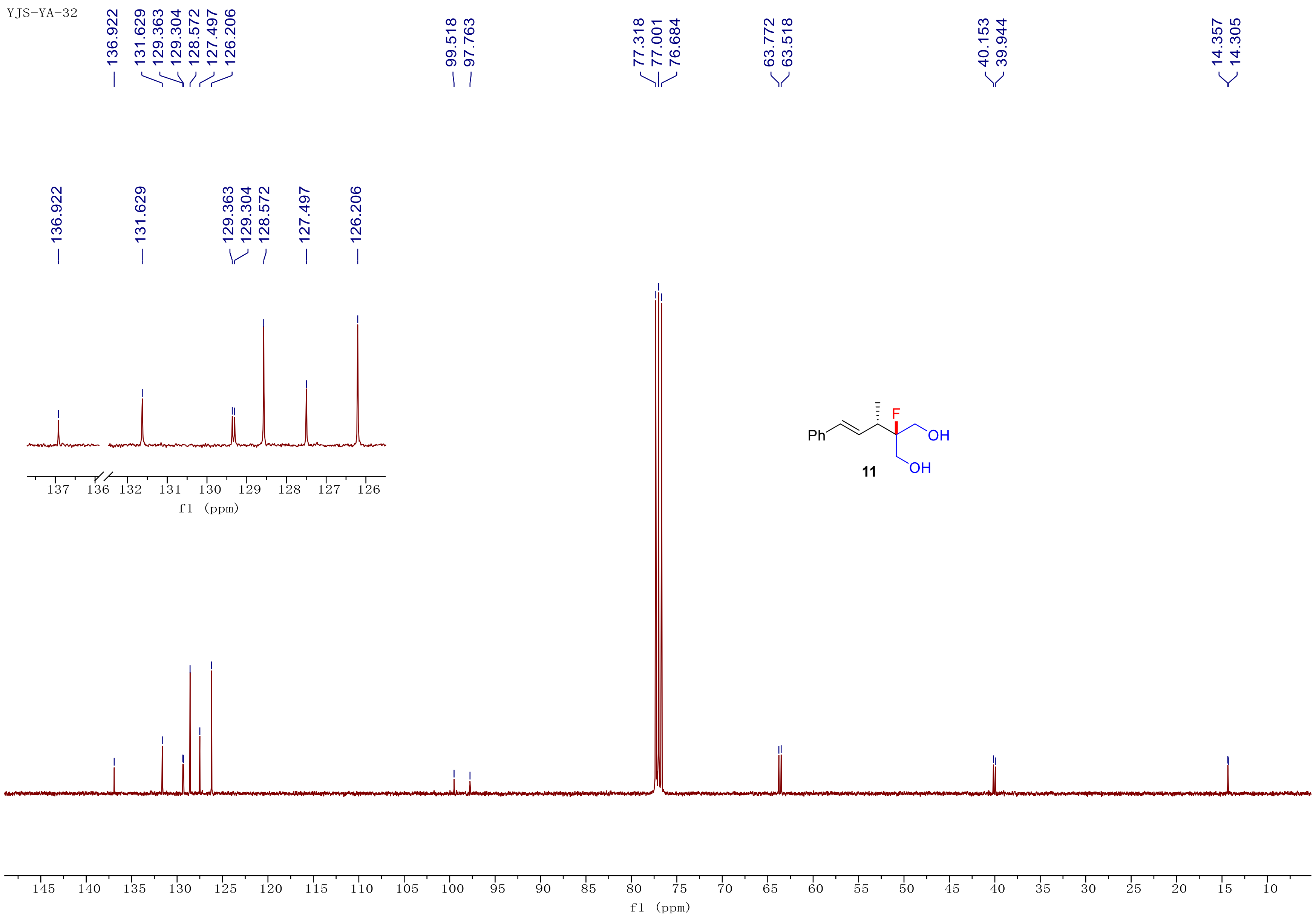
YJS-YA-32
136.922
131.629
129.363
129.304
128.572
127.497
126.206
99.518
97.763
77.318
77.001
76.684
63.772
63.518
40.153
39.944
14.357
14.305
Ph
F
OH
OH
11
f1 (ppm)

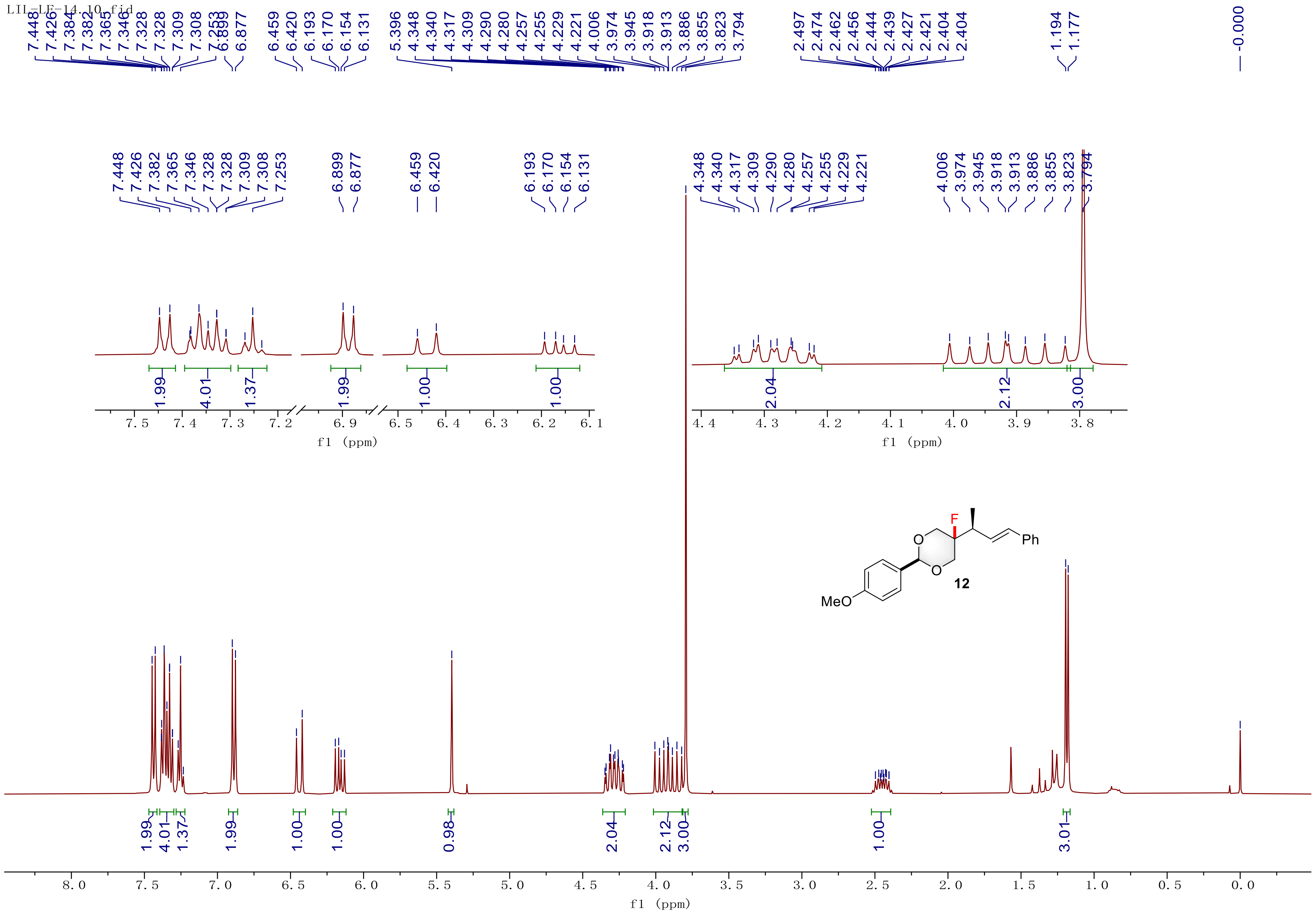
LJL-LF-14.10.fid
12
MeO
F
Ph
f1 (ppm)

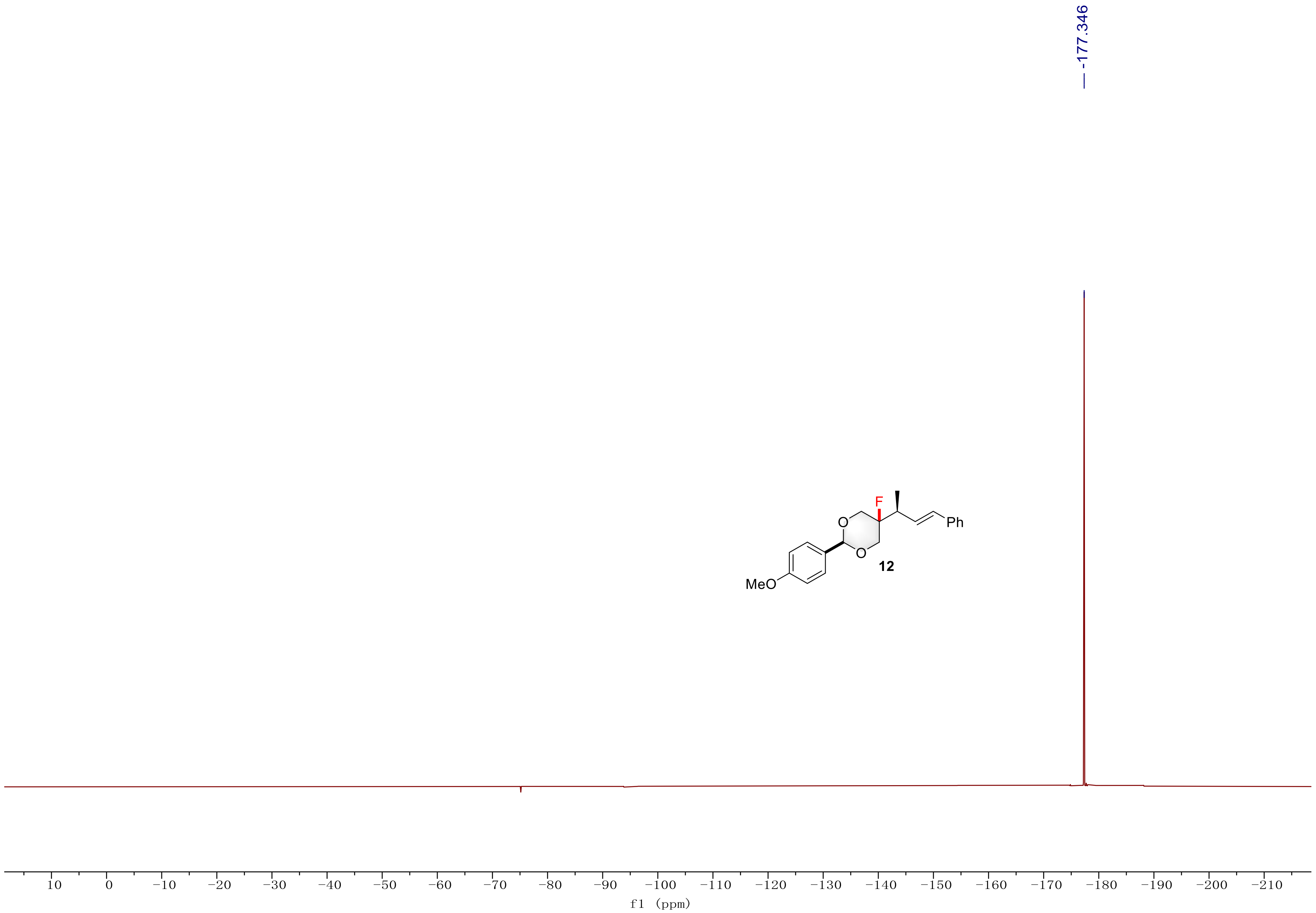
-177.346
F
Ph
MeO
12
10 0 −10 −20 −30 −40 −50 −60 −70 −80 −90 −100 −110 −120 −130 −140 −150 −160 −170 −180 −190 −200 −210
f1 (ppm)

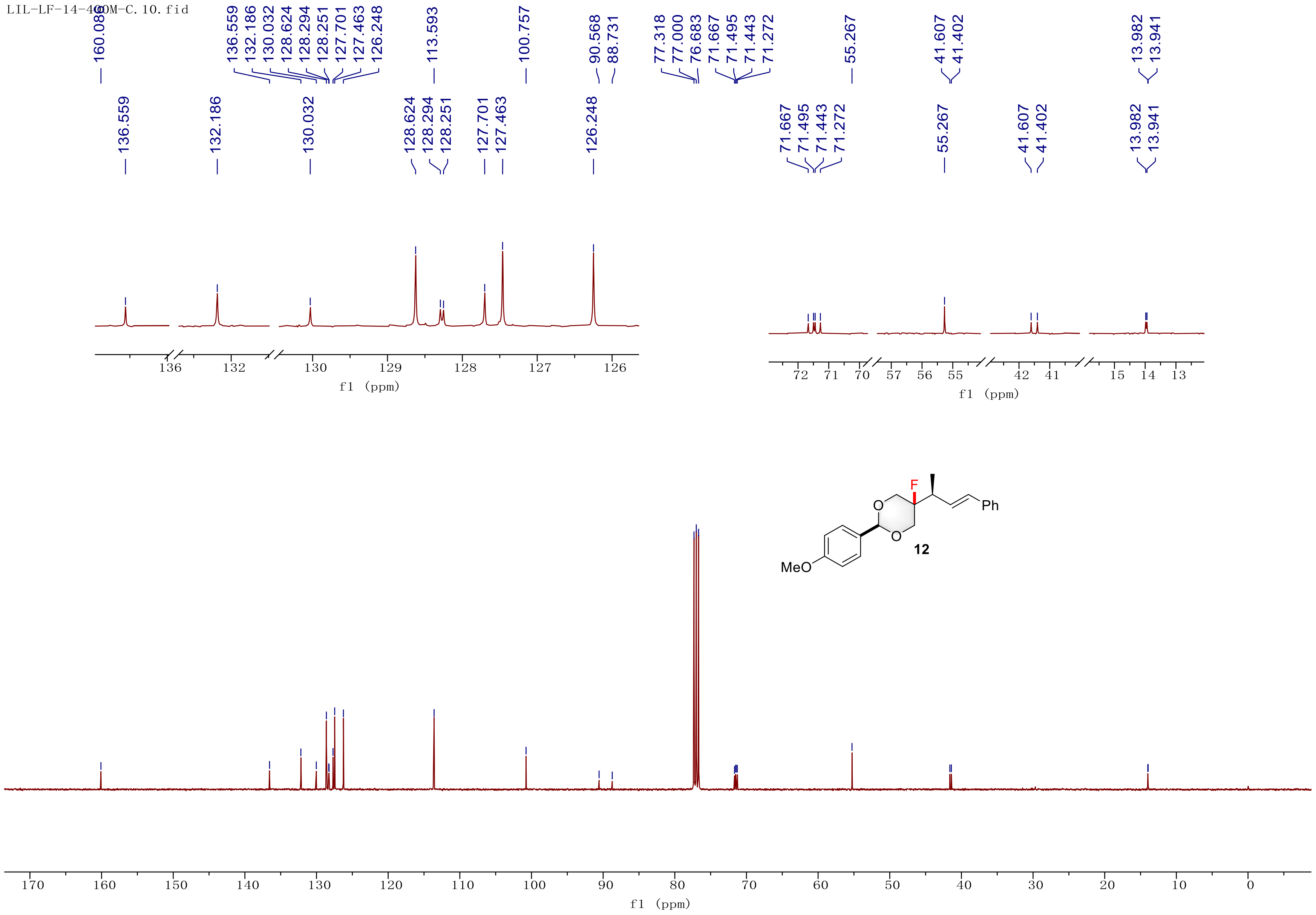
LIL-LF-14-400M-C.10.fid
160.080
136.559
132.186
130.032
128.624
128.294
128.251
127.701
127.463
126.248
113.593
100.757
90.568
88.731
77.318
77.000
76.683
71.667
71.495
71.443
71.272
55.267
41.607
41.402
13.982
13.941
F
O
O
Ph
MeO
12
f1 (ppm)

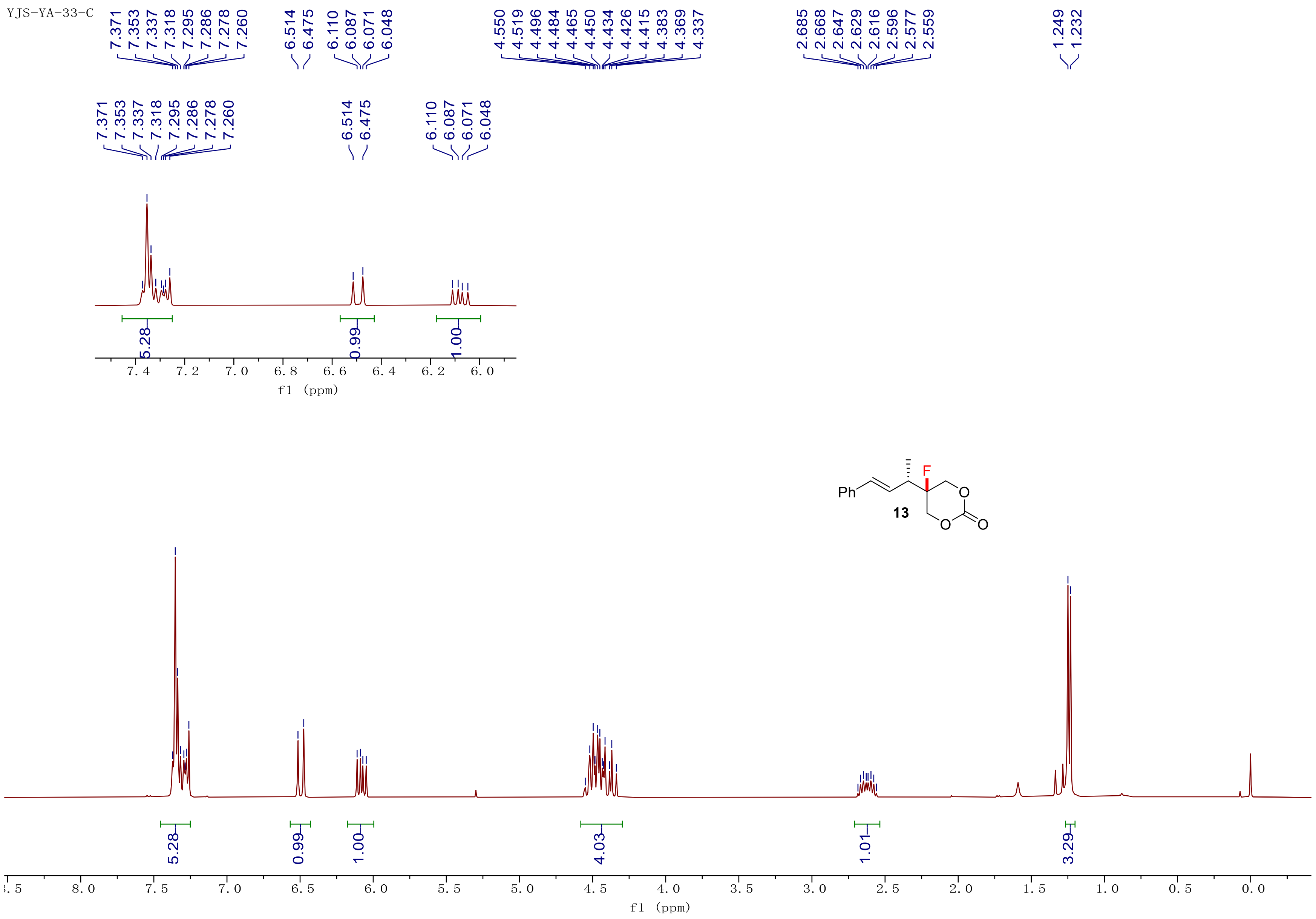
YJS-YA-33-C
7.371
7.353
7.337
7.318
7.295
7.286
7.278
7.260
6.514
6.475
6.110
6.087
6.071
6.048
4.550
4.519
4.496
4.484
4.465
4.450
4.434
4.426
4.415
4.383
4.369
4.337
2.685
2.668
2.647
2.629
2.616
2.596
2.577
2.559
1.249
1.232
5.28
0.99
1.00
4.03
1.01
3.29
f1 (ppm)
Ph
F
O
13

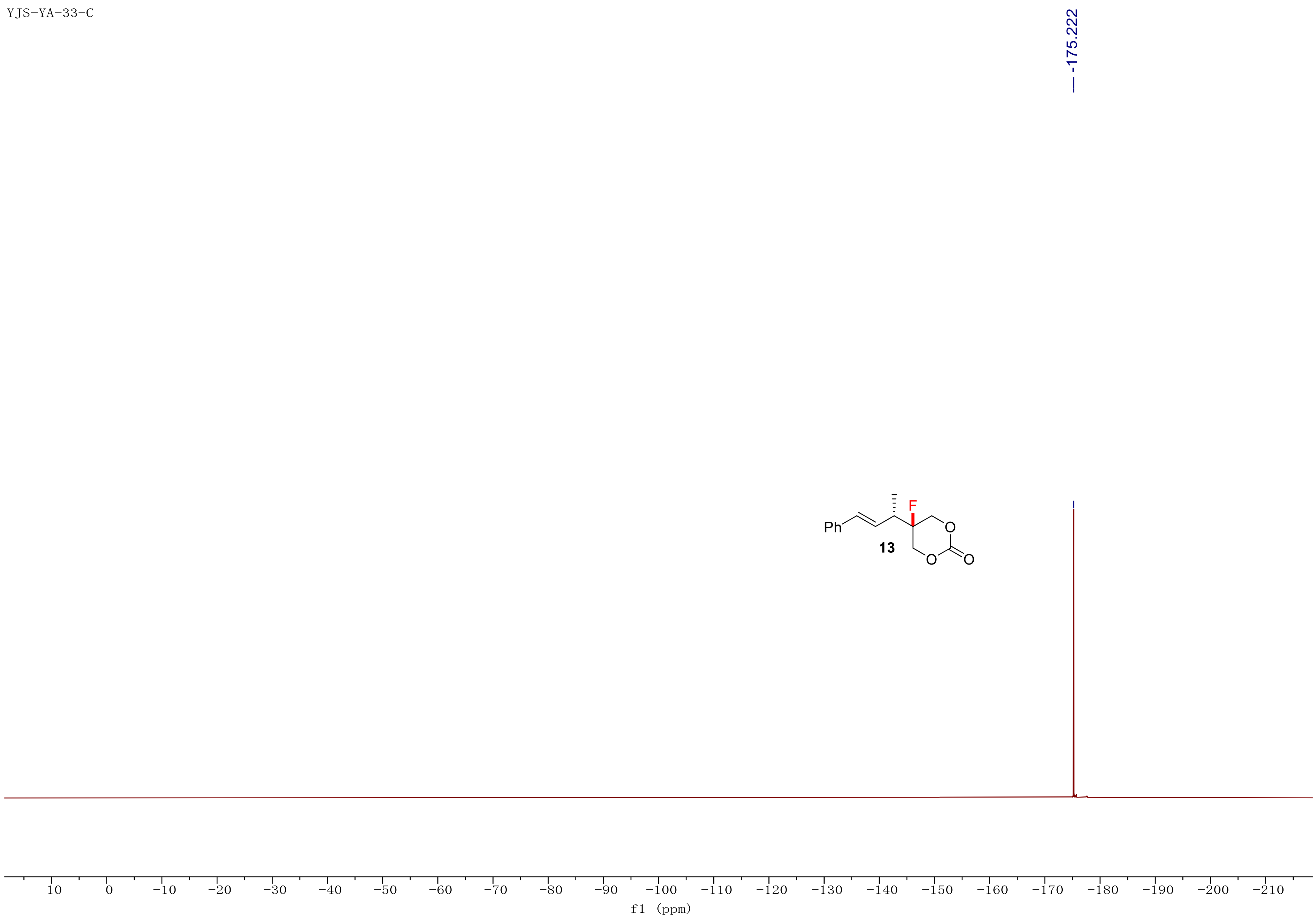
YJS-YA-33-C
-175.222
Ph
F
O
O
O
13
10 0 −10 −20 −30 −40 −50 −60 −70 −80 −90 −100 −110 −120 −130 −140 −150 −160 −170 −180 −190 −200 −210
f1 (ppm)

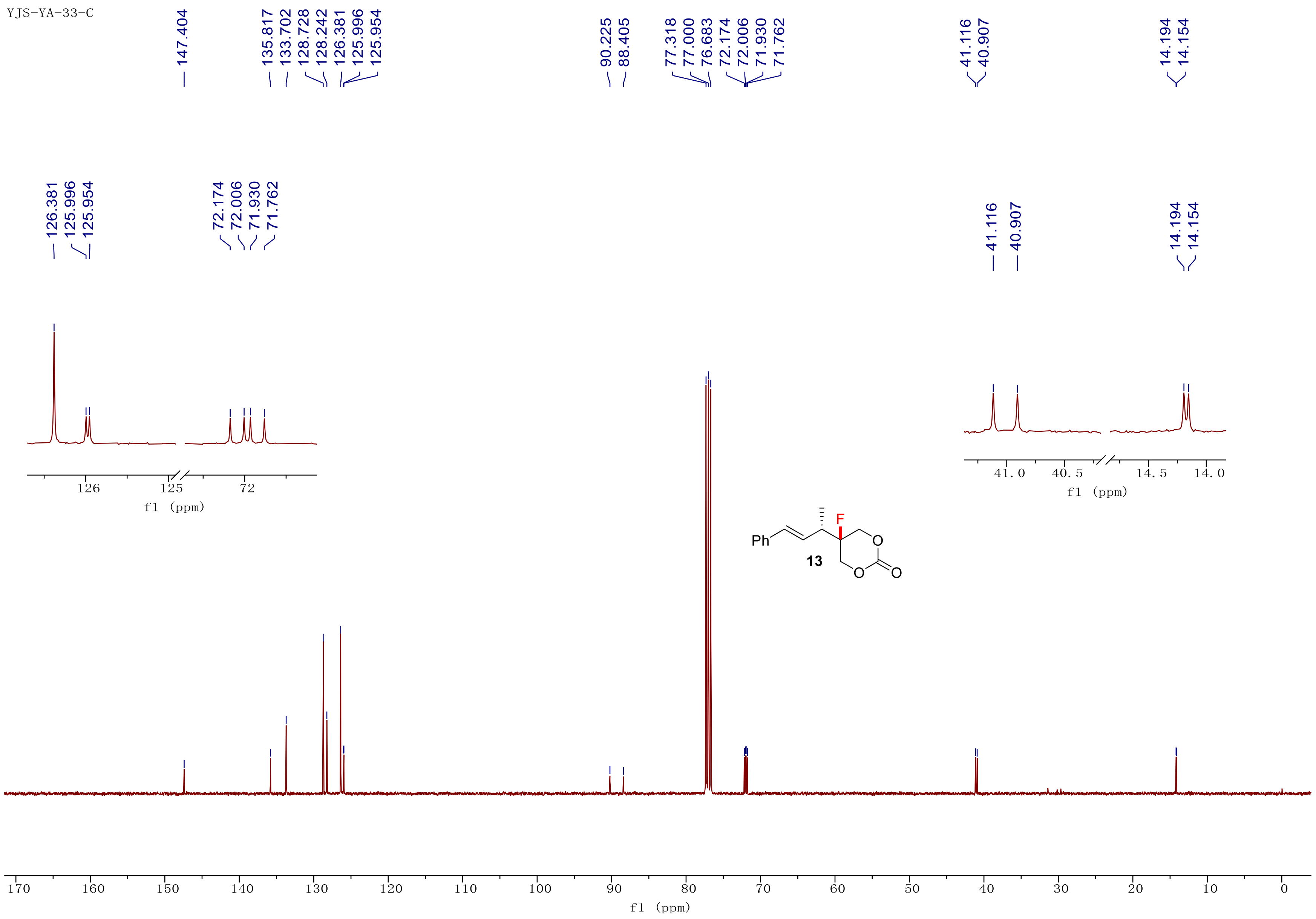
YJS-YA-33-C
147.404
135.817
133.702
128.728
128.242
126.381
125.996
125.954
90.225
88.405
77.318
77.000
76.683
72.174
72.006
71.930
71.762
41.116
40.907
14.194
14.154
Ph
F
O
O
O
13
f1 (ppm)

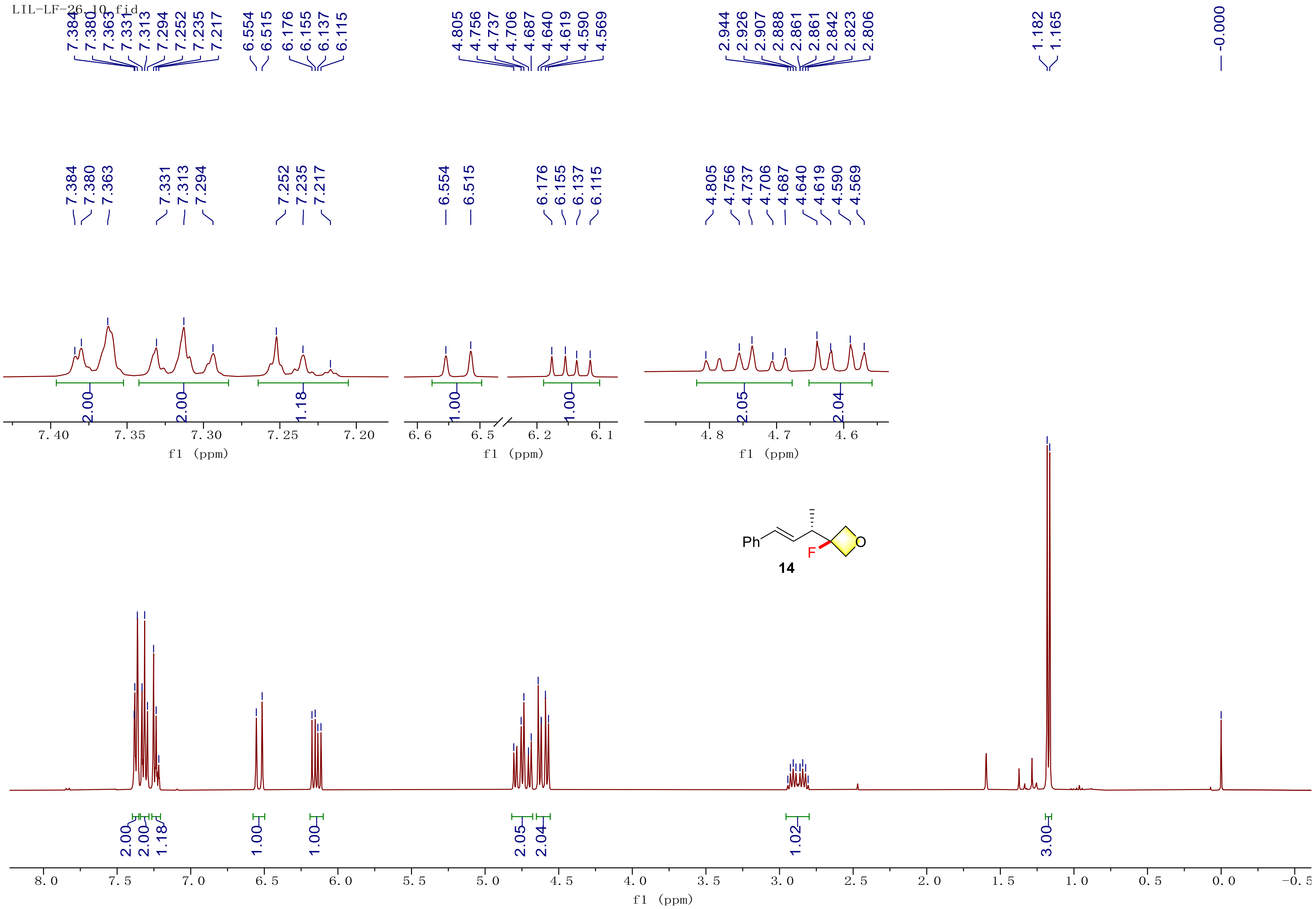
LIL-LF-26.10.fid
7.384 7.380 7.363 7.331 7.313 7.294 7.252 7.235 7.217
6.554 6.515 6.176 6.155 6.137 6.115
4.805 4.756 4.737 4.706 4.687 4.640 4.619 4.590 4.569
2.944 2.926 2.907 2.888 2.861 2.861 2.842 2.823 2.806
1.182 1.165
-0.000
2.00 2.00 1.18 1.00 1.00 2.05 2.04 1.02 3.00
f1 (ppm)
Ph
F
O
14

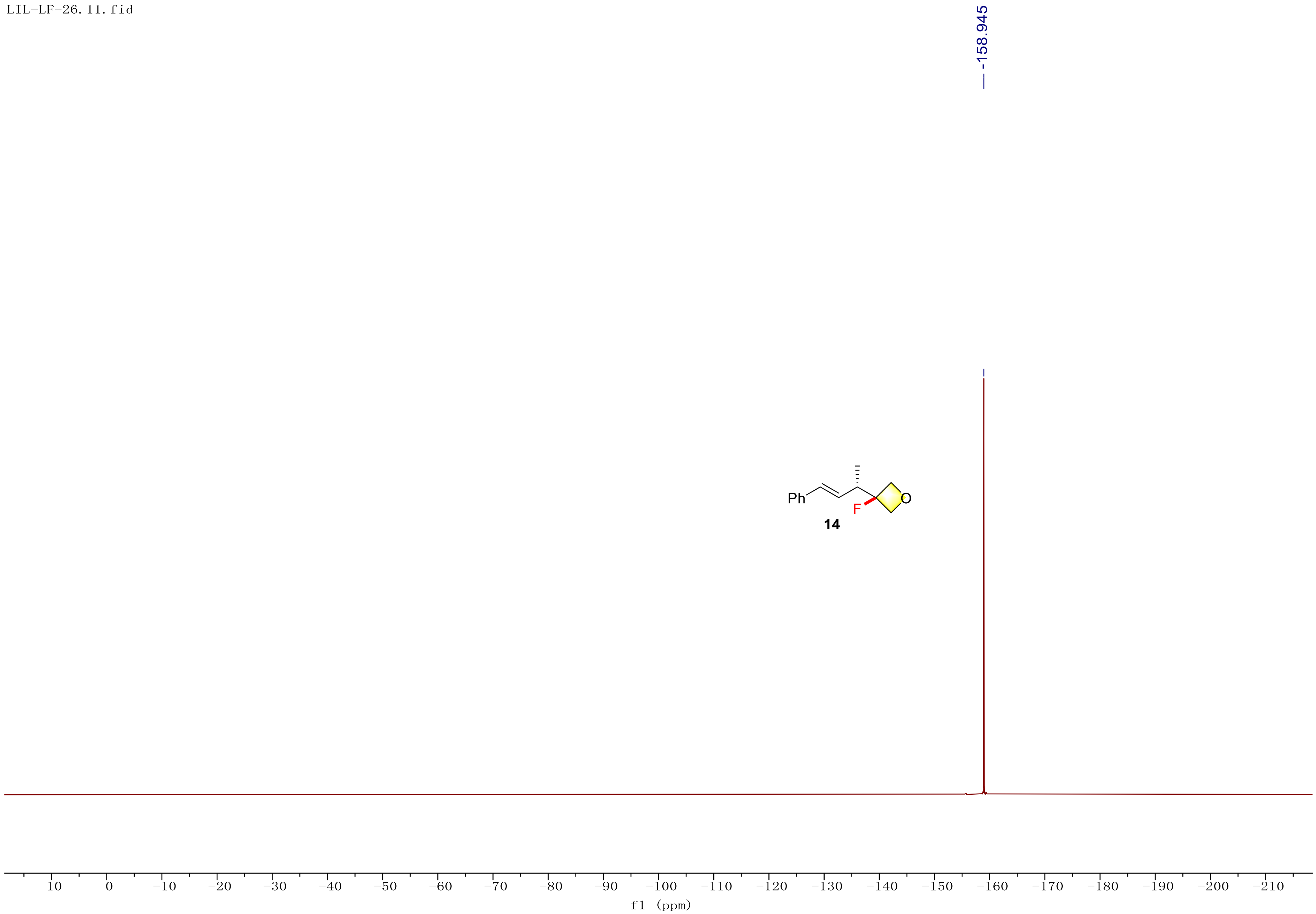
LIL-LF-26.11.fid
-158.945
Ph
F
O
14
10 0 −10 −20 −30 −40 −50 −60 −70 −80 −90 −100 −110 −120 −130 −140 −150 −160 −170 −180 −190 −200 −210
f1 (ppm)

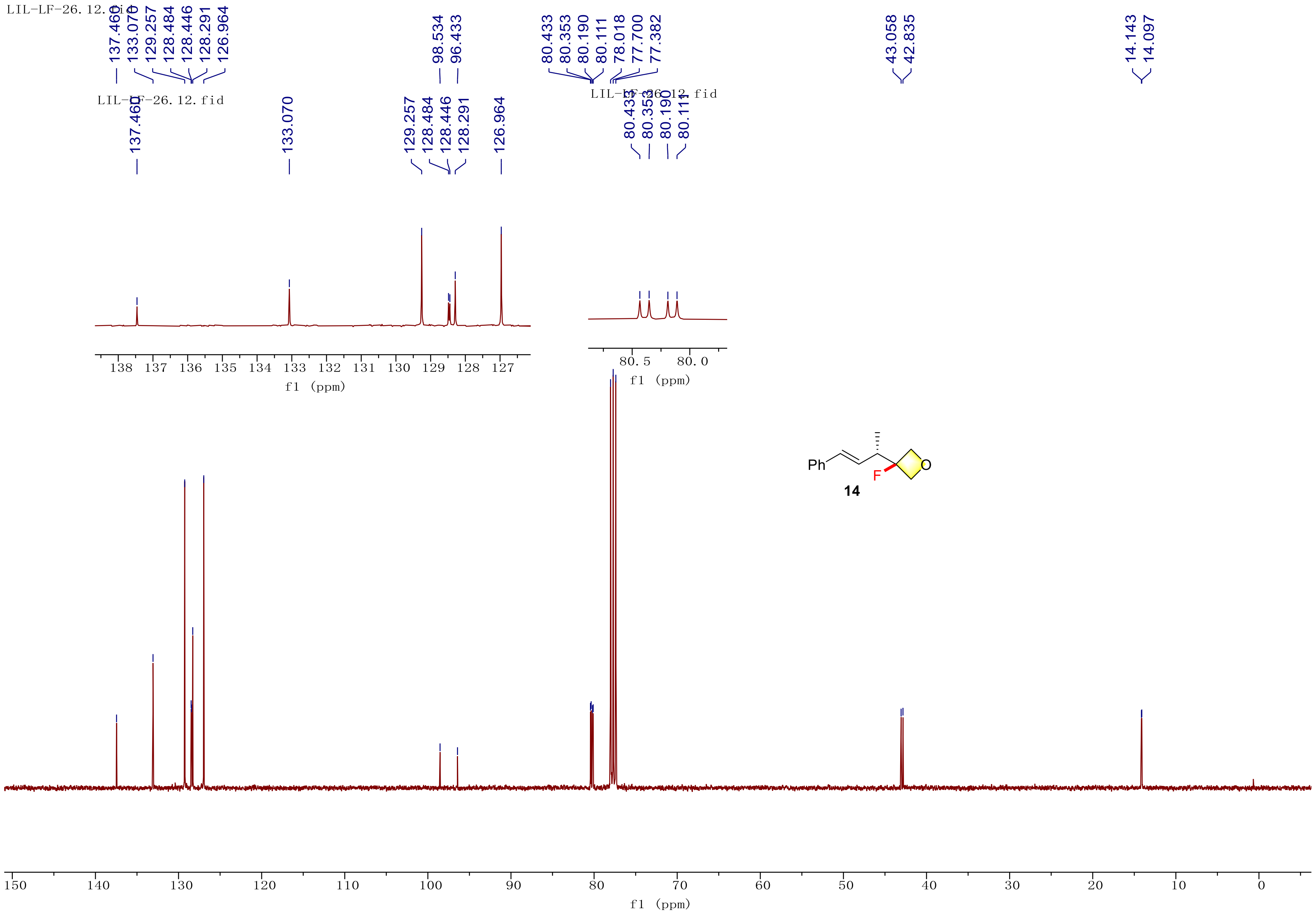
LIL-LF-26.12.fid
137.460
133.070
129.257
128.484
128.446
128.291
126.964
98.534
96.433
80.433
80.353
80.190
80.111
78.018
77.700
77.382
43.058
42.835
14.143
14.097
LIL-LF-26.12.fid
137.460
133.070
129.257
128.484
128.446
128.291
126.964
138 137 136 135 134 133 132 131 130 129 128 127
f1 (ppm)
LIL-LF-26.12.fid
80.433
80.353
80.190
80.111
80.5 80.0
f1 (ppm)
Ph
F
O
14
150 140 130 120 110 100 90 80 70 60 50 40 30 20 10 0
f1 (ppm)

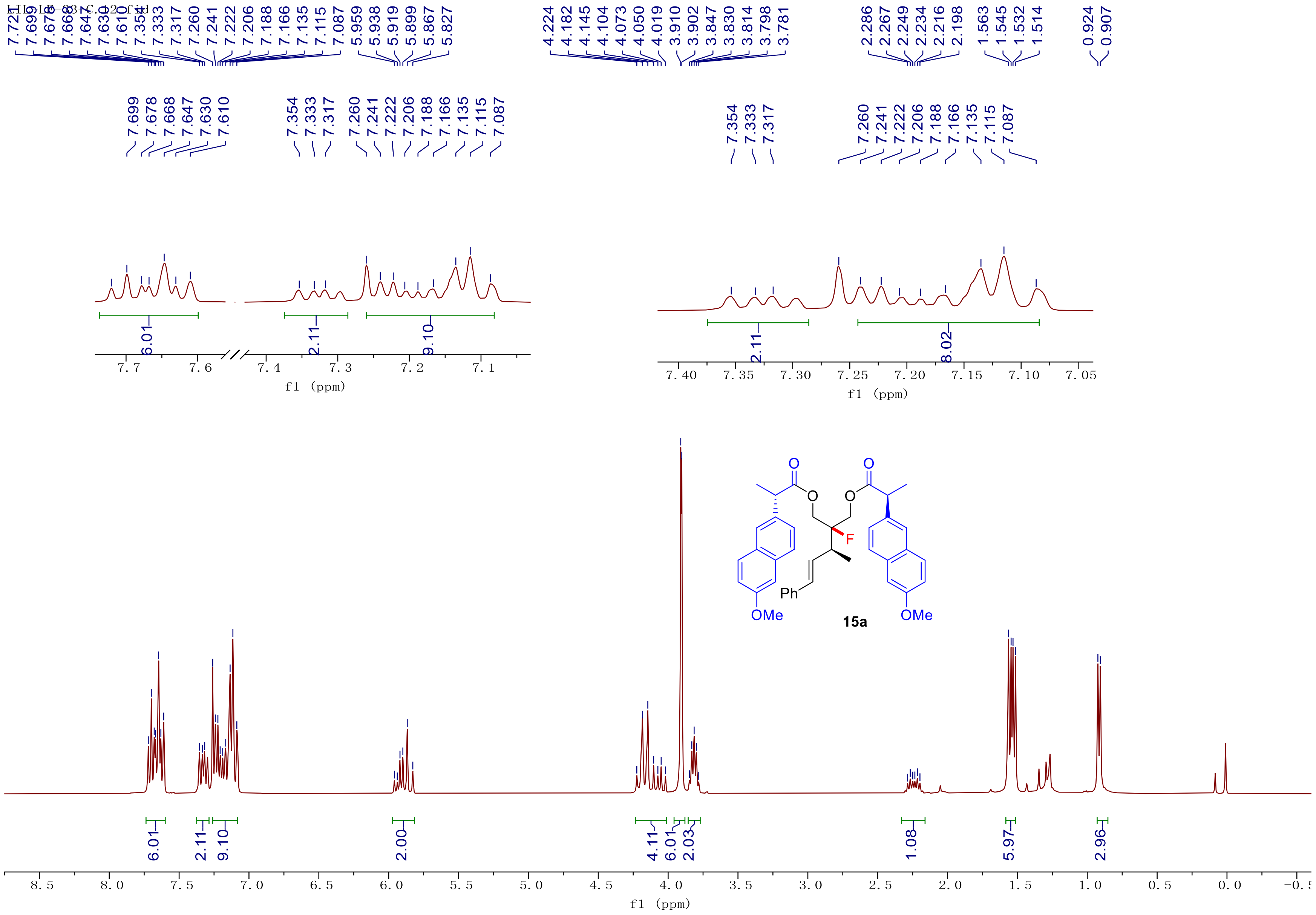
HHpld-83-C.12.fid
7.721 7.699 7.678 7.668 7.647 7.630 7.610 7.354 7.333 7.317 7.260 7.241 7.222 7.206 7.188 7.166 7.135 7.115 7.087 5.959 5.938 5.919 5.899 5.867 5.827
4.224 4.182 4.145 4.104 4.073 4.050 4.019 3.910 3.902 3.847 3.830 3.814 3.798 3.781
2.286 2.267 2.249 2.234 2.216 2.198 1.563 1.545 1.532 1.514 0.924 0.907
6.01
2.11
9.10
8.02
2.00
4.11
6.01
2.03
1.08
5.97
2.96
f1 (ppm)
O
O
F
Ph
OMe
15a

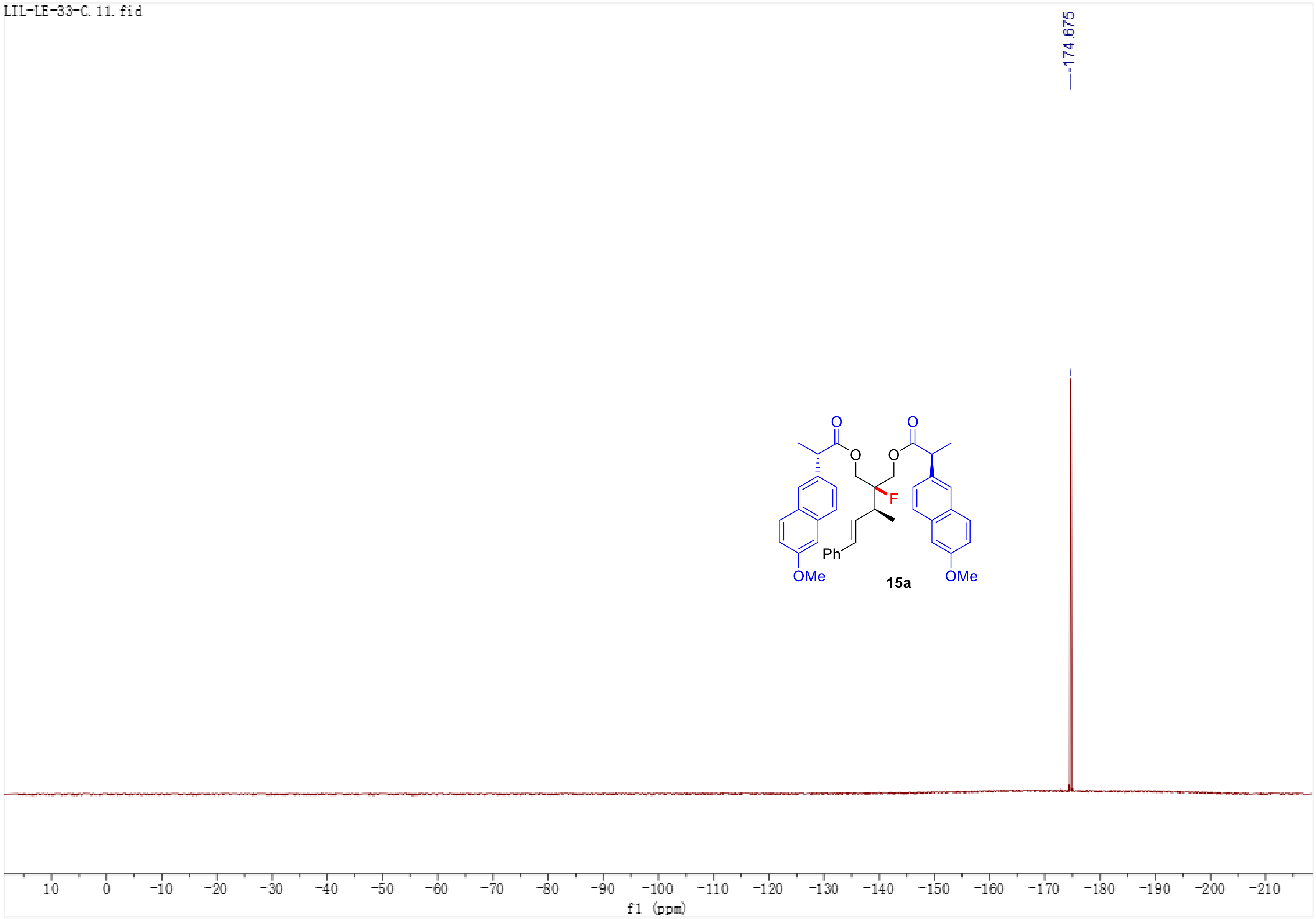
LIL-LE-33-C.11.fid
-174.675
O
O
O
O
F
Ph
OMe
OMe
15a
10 0 -10 -20 -30 -40 -50 -60 -70 -80 -90 -100 -110 -120 -130 -140 -150 -160 -170 -180 -190 -200 -210
f1 (ppm)

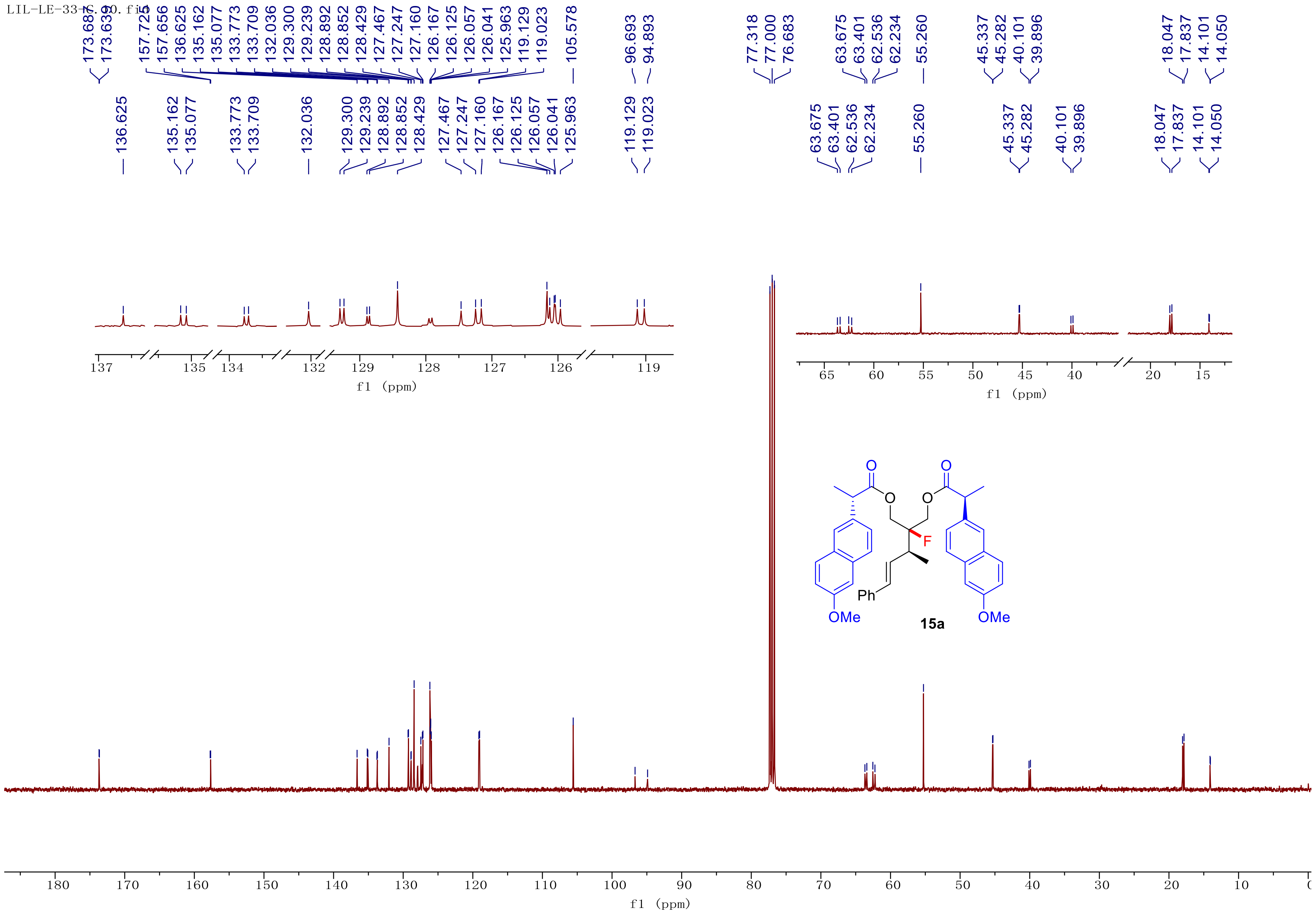
LIL-LE-33-C.10.fid
OMe
OMe
Ph
F
15a
f1 (ppm)

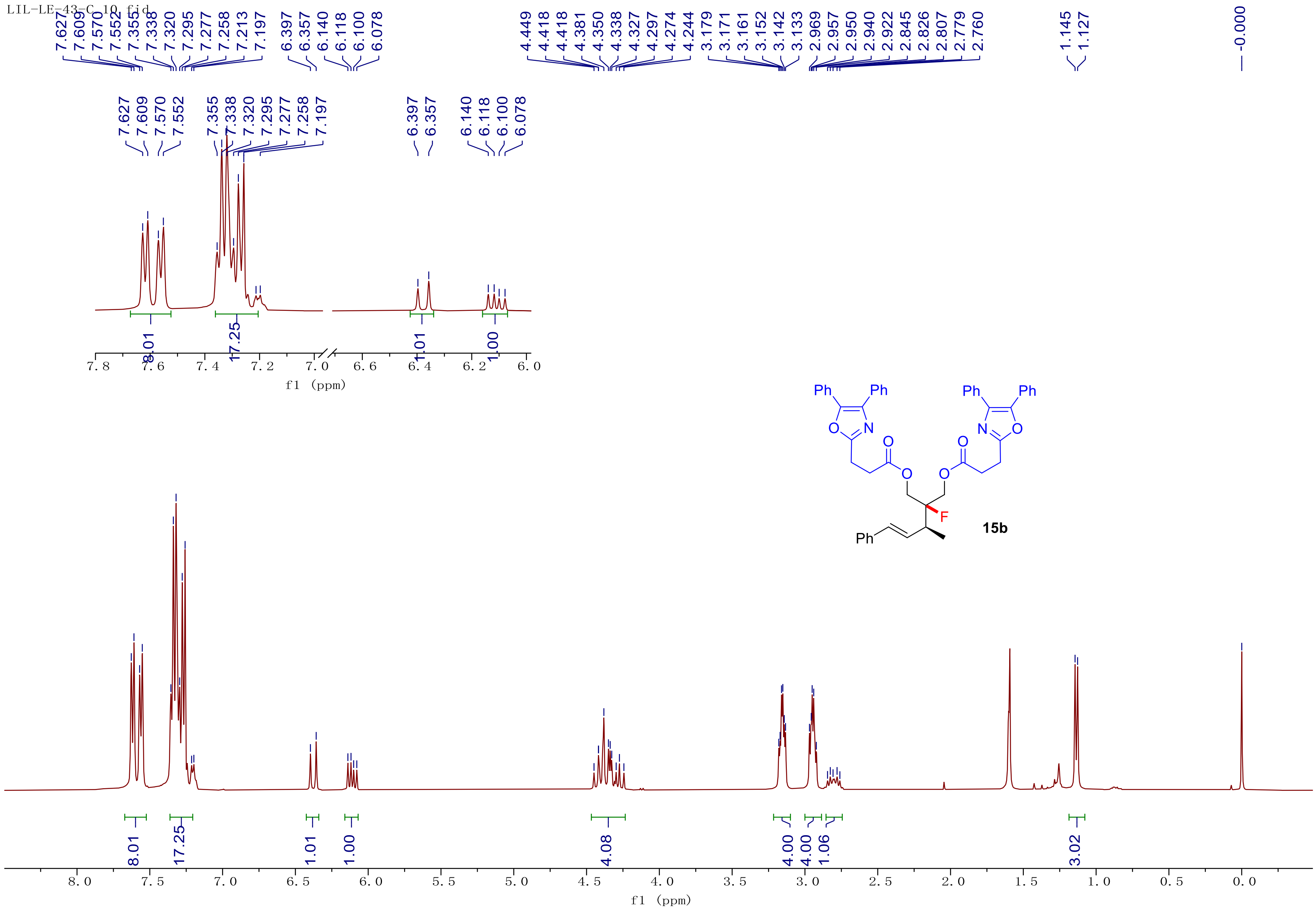
LIL-LE-43-C_10.fid
7.627 7.609 7.570 7.552 7.355 7.338 7.320 7.295 7.277 7.258 7.213 7.197 6.397 6.357 6.140 6.118 6.100 6.078
4.449 4.418 4.418 4.381 4.350 4.338 4.327 4.297 4.274 4.244 3.179 3.171 3.161 3.152 3.142 3.133 2.969 2.957 2.950 2.940 2.922 2.845 2.826 2.807 2.779 2.760
1.145 1.127
-0.000
8.01 17.25 1.01 1.00
f1 (ppm)
8.01 17.25 1.01 1.00 4.08 4.00 4.00 1.06 3.02
f1 (ppm)
Ph Ph Ph Ph
O N N O
O O
O O
F
Ph
15b

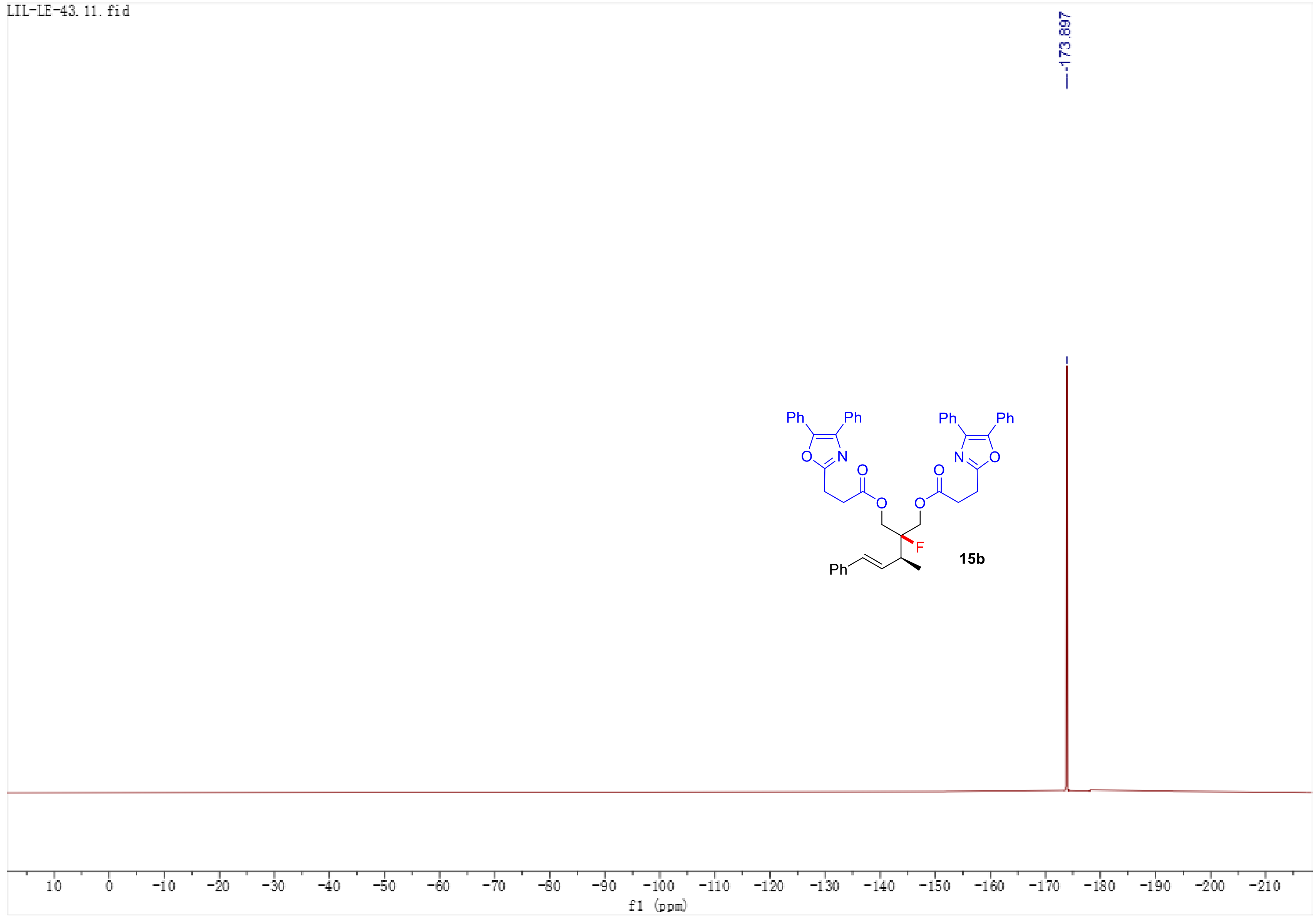
LIL-LE-43.11.fid
-173.897
Ph
Ph
O
N
O
O
Ph
Ph
N
O
O
O
F
Ph
15b
10 0 -10 -20 -30 -40 -50 -60 -70 -80 -90 -100 -110 -120 -130 -140 -150 -160 -170 -180 -190 -200 -210
f1 (ppm)

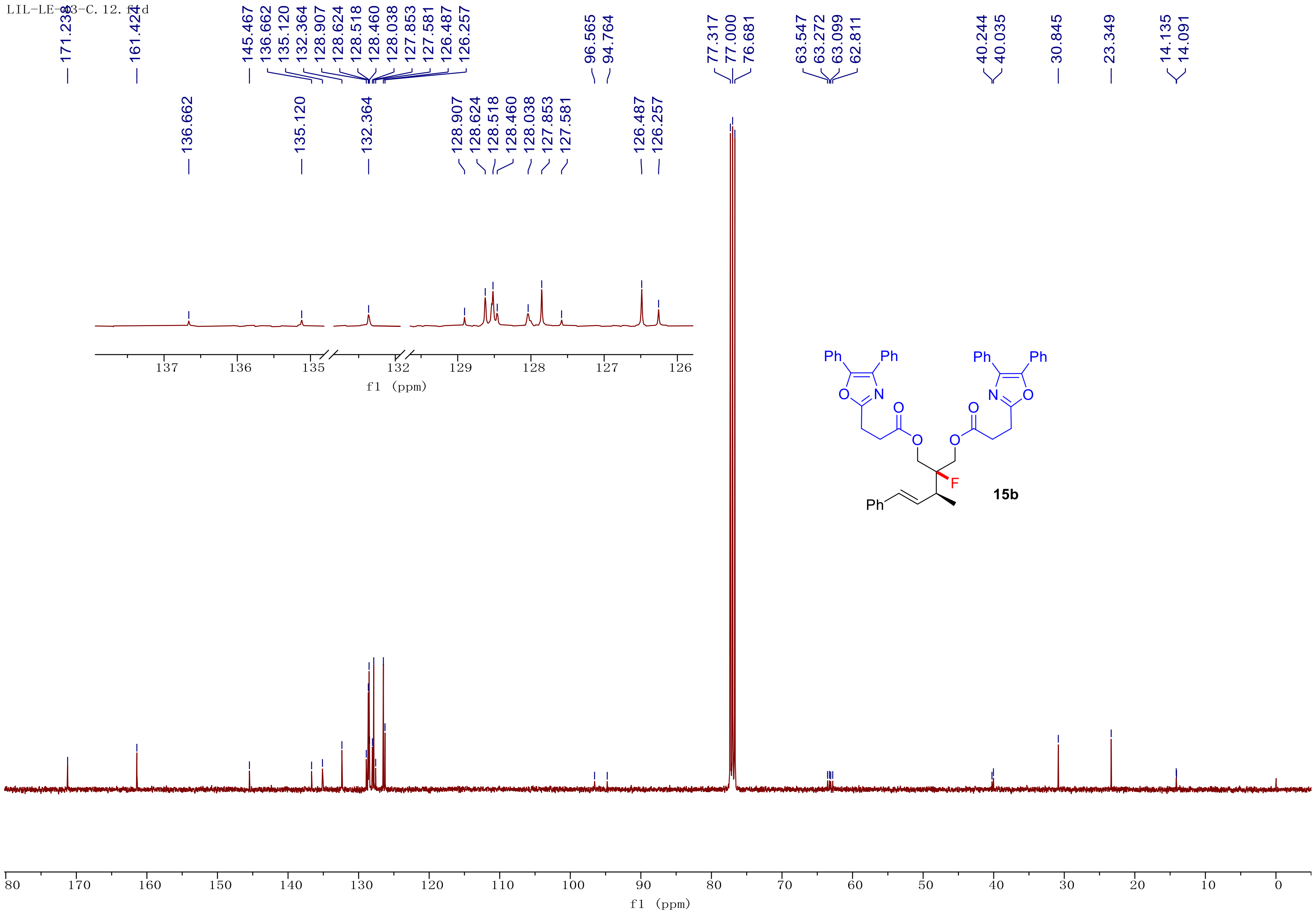
LIL-LE-03-C.12.fid
171.238
161.424
145.467
136.662
135.120
132.364
128.907
128.624
128.518
128.460
128.038
127.853
127.581
126.487
126.257
96.565
94.764
77.317
77.000
76.681
63.547
63.272
63.099
62.811
40.244
40.035
30.845
23.349
14.135
14.091
f1 (ppm)
Ph
O
N
F
15b

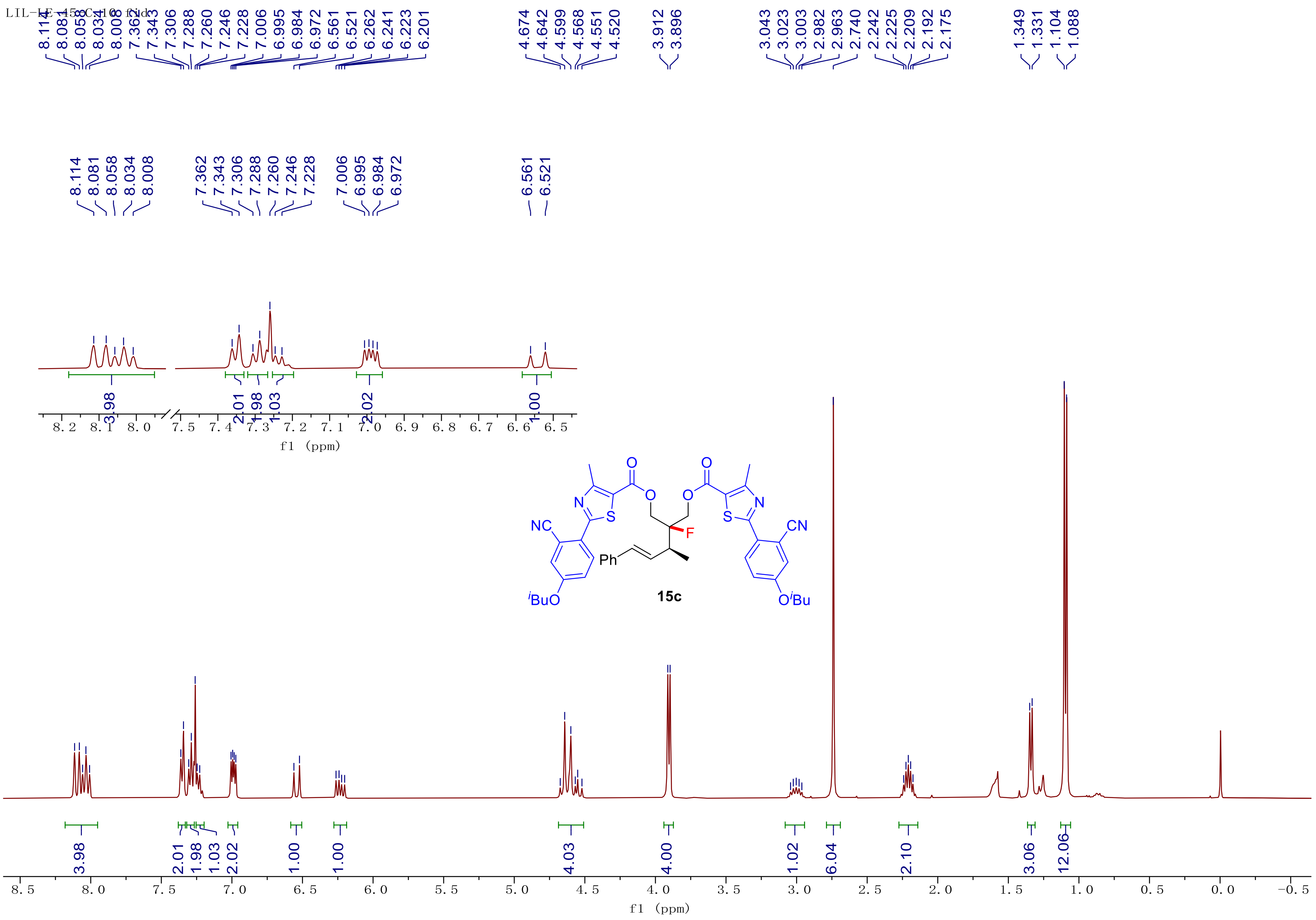
15c
f1 (ppm)

LIL-LE-45-C.11.fid

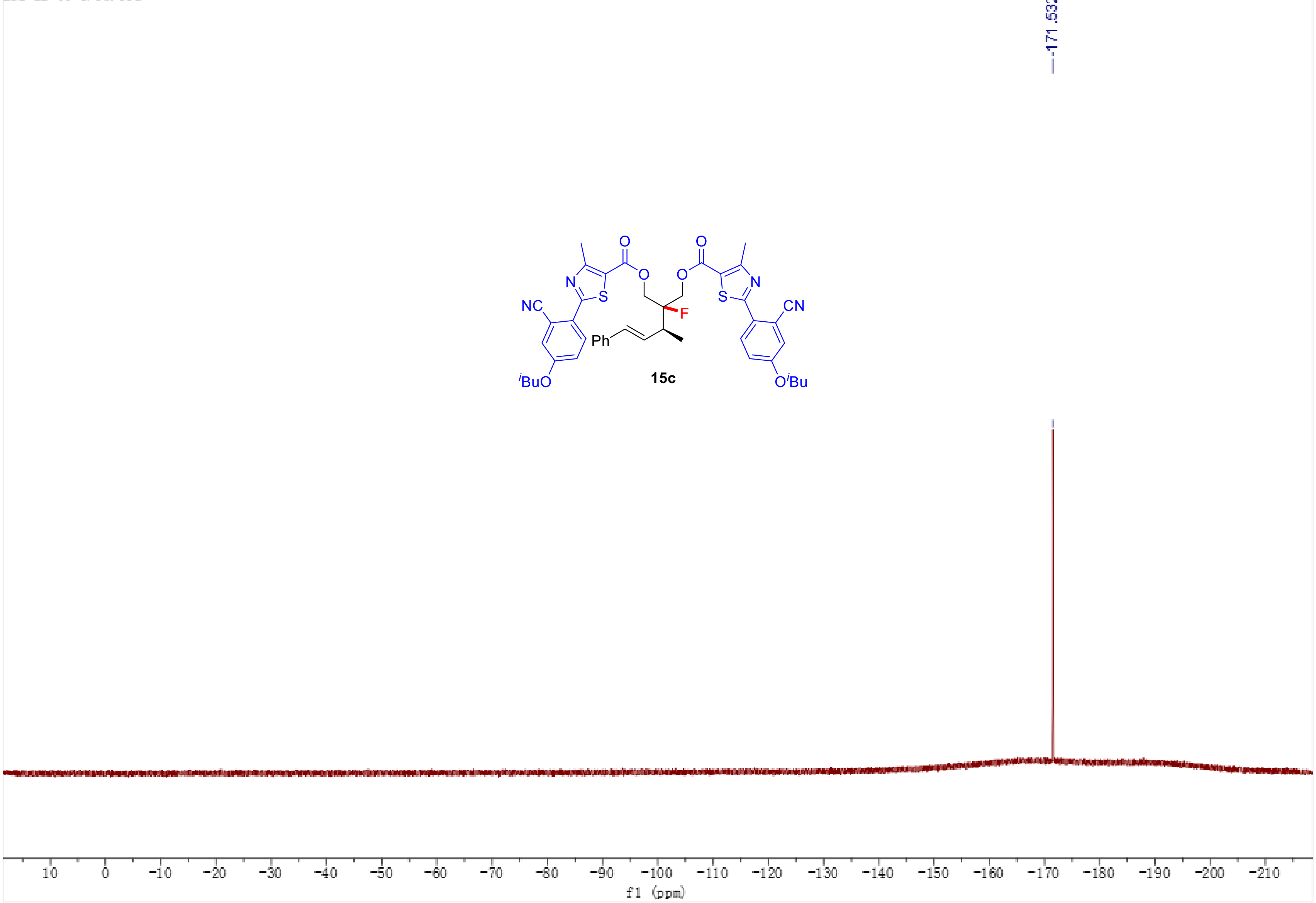

15c

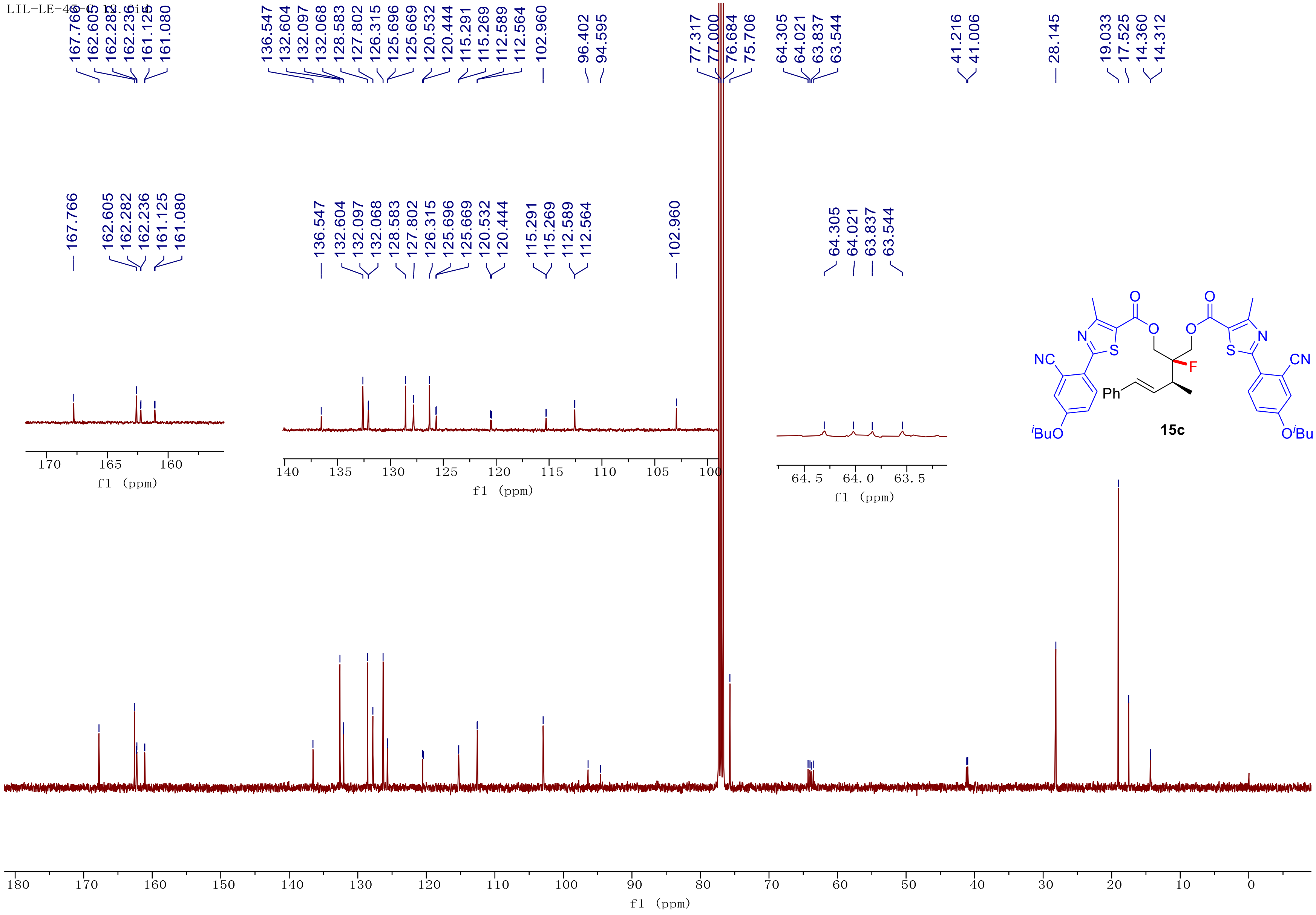
167.766
162.605
162.282
162.236
161.125
161.080
136.547
132.604
132.097
132.068
128.583
127.802
126.315
125.696
125.669
120.532
120.444
115.291
115.269
112.589
112.564
102.960
96.402
94.595
77.317
77.000
76.684
75.706
64.305
64.021
63.837
63.544
41.216
41.006
28.145
19.033
17.525
14.360
14.312
NC
N
S
O
O
F
Ph
^{i}BuO
15c
CN
O^{i}Bu
f1 (ppm)

10. HPLC traces

2021/7/13 16:42:55 Page 1 / 1

Analysis Report

<Sample Information>

Sample Name : zhy-zb-91-rac-oxh-80-20-1.0-254-2
Sample ID :
Data Filename : zhy-zb-91-rac-oxh-80-20-1.0-254-2.lcd
Method Filename : 1.0.lcm
Batch Filename :
Vial # : 1-1 Sample Type : Unknown
Injection Volume : 20 uL
Date Acquired : 2021/7/9 16:23:53 Acquired by : System Administrator
Date Processed : 2021/7/9 16:46:01 Processed by : System Administrator

<Chromatogram>

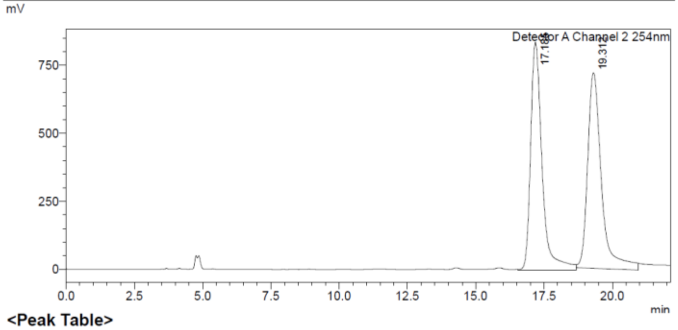

<Peak Table>

Detector A Channel 2 254nm

Peak#	Ret. Time	Area	Height	Conc.
1	17.185	24388331	837306	49.110
2	19.312	25271850	719407	50.890
Total		49660181	1556713	

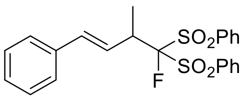

***rac*-3a**

D:\Data\ZHY\ZB\zhy-zb-91-rac-oxh-80-20-1.0-254-2.lcd

2021/7/30 21:52:14 Page 1 / 1

Analysis Report

<Sample Information>

Sample Name : zhy-zb-110-1-asy-oxh-80-20-254-2
Sample ID :
Data Filename : zhy-zb-110-1-asy-oxh-80-20-254-2.lcd
Method Filename : 1.0.lcm
Batch Filename :
Vial # : 1-1 Sample Type : Unknown
Injection Volume : 20 uL
Date Acquired : 2021/7/29 22:10:22 Acquired by : System Administrator
Date Processed : 2021/7/29 22:32:47 Processed by : System Administrator

<Chromatogram>

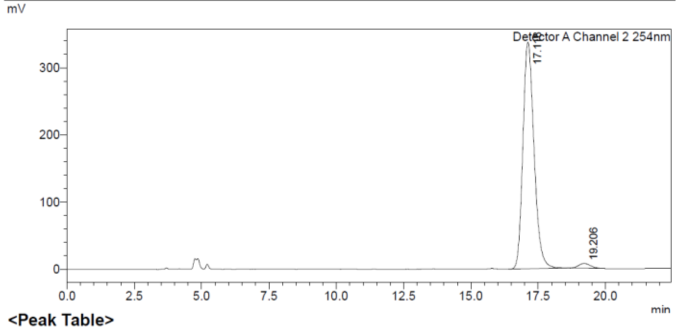

<Peak Table>

Detector A Channel 1 205nm

Peak#	Ret. Time	Area	Height	Conc.
1	17.116	17109271	594579	98.063
2	19.177	337896	11882	1.937
Total		17447166	606460	

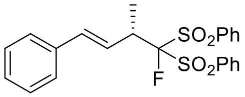

3a

D:\Data\ZHY\ZB\zhy-zb-110-1-asy-oxh-80-20-254-2.lcd

Analysis Report

<Sample Information>

Sample Name : zhy-zc-98-rac-oxh-80-20-1.0-254-205-1
Sample ID :
Data Filename : zhy-zc-98-rac-oxh-80-20-1.0-254-205-1.lcd
Method Filename : 1.0.lcm
Batch Filename :
Vial # : 1-1 Sample Type : Unknown
Injection Volume : 20 uL
Date Acquired : 2021/10/29 15:08:10 Acquired by : System Administrator
Date Processed : 2021/10/29 15:32:09 Processed by : System Administrator

<Chromatogram>

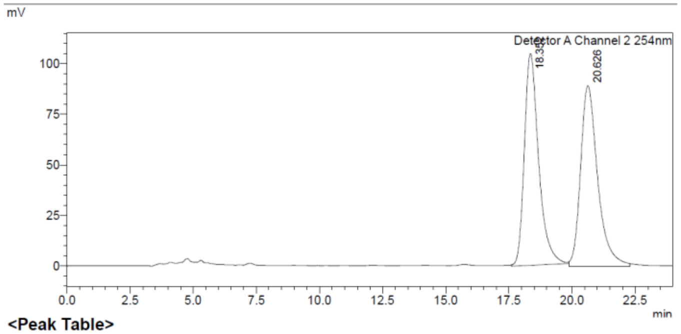

<Peak Table>

Detector A Channel 2 254nm

Peak#	Ret. Time	Area	Height	Conc.
1	18.352	4121317	104706	49.632
2	20.626	4182485	89224	50.368
Total		8303802	193930	

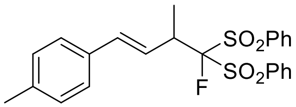

***rac*-3b**

Analysis Report

<Sample Information>

Sample Name : zhy-zc-98-asy-oxh-80-20-1.0-254-205-1
Sample ID :
Data Filename : zhy-zc-98-asy-oxh-80-20-1.0-254-205-1.lcd
Method Filename : 1.0.lcm
Batch Filename :
Vial # : 1-1 Sample Type : Unknown
Injection Volume : 20 uL
Date Acquired : 2021/10/29 14:27:28 Acquired by : System Administrator
Date Processed : 2021/10/29 14:54:36 Processed by : System Administrator

<Chromatogram>

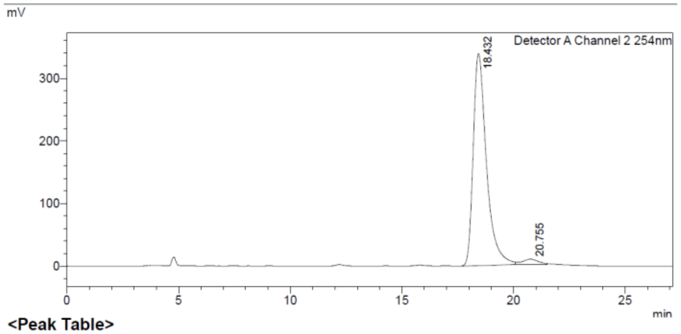

<Peak Table>

Detector A Channel 2 254nm

Peak#	Ret. Time	Area	Height	Conc.
1	18.432	13934638	338741	96.834
2	20.755	455580	8672	3.166
Total		14390218	347414	

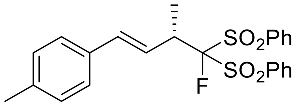

3b

Analysis Report

<Sample Information>

Sample Name	: zhy-zc-21-1-rac-oxh-1.0-254nm-01		
Sample ID	:		
Data Filename	: zhy-zc-21-1-rac-oxh-1.0-254nm-01.lcd		
Method Filename	: 1.0.lcm		
Batch Filename	:		
Vial #	: 1-1	Sample Type	: Unknown
Injection Volume	: 20 uL		
Date Acquired	: 2021/9/16 22:00:26	Acquired by	: System Administrator
Date Processed	: 2021/9/16 22:40:22	Processed by	: System Administrator

<Chromatogram>

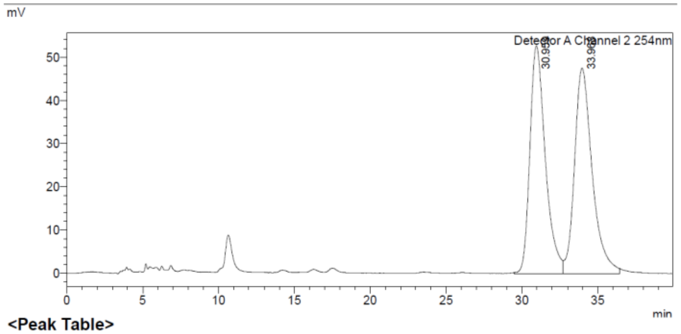

<Peak Table>

Detector A Channel 2 254nm

Peak#	Ret. Time	Area	Height	Conc.
1	30.959	3726525	52666	49.153
2	33.968	3854982	47529	50.847
Total		7581507	100195	

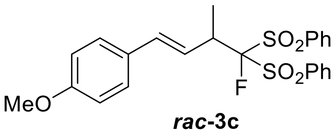

***rac*-3c**

Analysis Report

<Sample Information>

Sample Name	: zhy-zc-21-1-asy-oxh-1.0-254nm-01		
Sample ID	:		
Data Filename	: zhy-zc-21-1-asy-oxh-1.0-254nm-01.lcd		
Method Filename	: 1.0.lcm		
Batch Filename	:		
Vial #	: 1-1	Sample Type	: Unknown
Injection Volume	: 20 uL		
Date Acquired	: 2021/9/16 20:51:31	Acquired by	: System Administrator
Date Processed	: 2021/9/16 21:28:38	Processed by	: System Administrator

<Chromatogram>

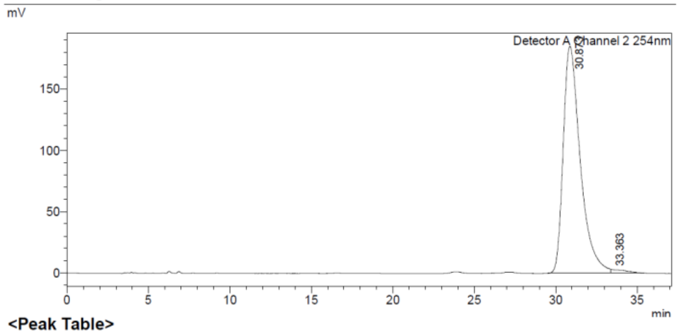

<Peak Table>

Detector A Channel 2 254nm

Peak#	Ret. Time	Area	Height	Conc.
1	30.873	13480901	185167	98.566
2	33.363	196098	3294	1.434
Total		13676999	188461	

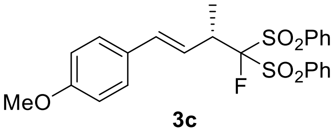

3c

Operator:dell Timebase:U-3000 Sequence:Zhang Xuexin

Page 1-1
2008-8-20 12:13 下午

254 ZHY-ZC-117-RAC-OXH-80-20-1.0-254-1

Sample Name:	**ZHY-ZC-117-RAC-OXH-80-20-1.0-254-1**	*Injection Volume:*	**20.0**
Vial Number:	**352**	*Channel:*	**UV_VIS_1**
Sample Type:	**standard**	*Wavelength:*	**254**
Control Program:	**Zhang Xuexin**	*Bandwidth:*	**n.a.**
Quantif. Method:	**Zhang Xuexin**	*Dilution Factor:*	**1.0000**
Run Time (min):	**34.65**	*Sample Amount:*	**1.0000**

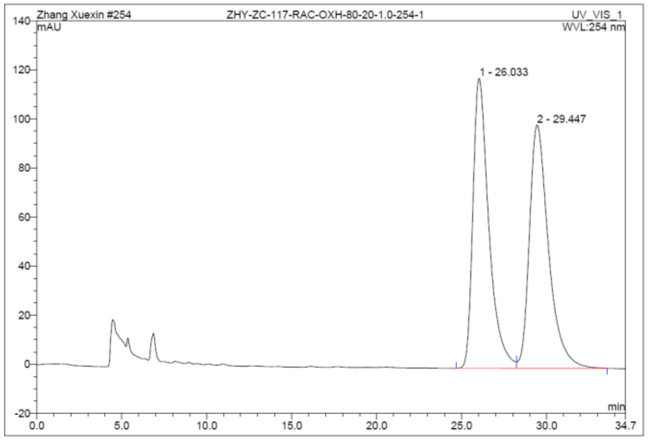

No.	Ret.Time min	Peak Name	Height mAU	Area mAU*min	Rel.Area %	Amount	Type
1	26.03	n.a.	118.203	128.222	49.76	n.a.	BM
2	29.45	n.a.	99.210	129.444	50.24	n.a.	MB
Total:			217.413	257.666	100.00	0.000	

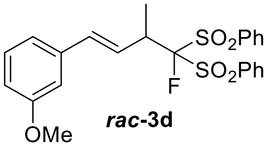

***rac*-3d**

Mu/Integration

Chromeleon (c) Dionex 1996-2006
Version 6.80 SR8a Build 2643 (158225)

Operator:dell Timebase:U-3000 Sequence:Zhang Xuexin

Page 1-1
2008-8-20 8:34 上午

256 ZHY-ZC-117-ASY-OXH-80-20-1.0-254-2

Sample Name:	**ZHY-ZC-117-ASY-OXH-80-20-1.0-254-2**	*Injection Volume:*	**20.0**
Vial Number:	**354**	*Channel:*	**UV_VIS_1**
Sample Type:	**standard**	*Wavelength:*	**254**
Control Program:	**Zhang Xuexin**	*Bandwidth:*	**n.a.**
Quantif. Method:	**Zhang Xuexin**	*Dilution Factor:*	**1.0000**
Run Time (min):	**35.17**	*Sample Amount:*	**1.0000**

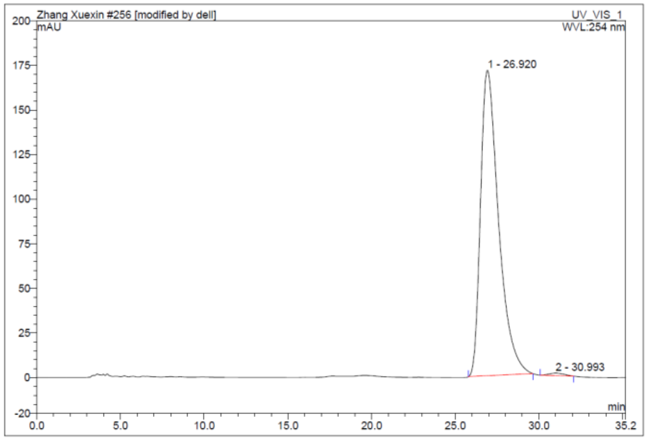

No.	Ret.Time min	Peak Name	Height mAU	Area mAU*min	Rel.Area %	Amount	Type
1	26.92	n.a.	171.178	213.866	99.33	n.a.	BMB*
2	30.99	n.a.	1.384	1.445	0.67	n.a.	BMB*
Total:			172.562	215.311	100.00	0.000	

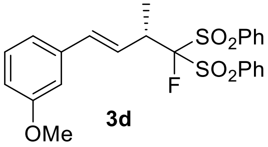

3d

Mu/Integration

Chromeleon (c) Dionex 1996-2006
Version 6.80 SR8a Build 2643 (158225)

Operator:dell Timebase:U-3000 Sequence:Zhang Xuexin

Page 1-1
2022-1-13 10:25 上午

280 zhy-zc-78-rac-oxh-80-20-1.0-254-1

Sample Name:	**zhy-zc-78-rac-oxh-80-20-1.0-254-1**	*Injection Volume:*	**20.0**
Vial Number:	**378**	*Channel:*	**UV_VIS_1**
Sample Type:	**standard**	*Wavelength:*	**254**
Control Program:	**Zhang Xuexin**	*Bandwidth:*	**n.a.**
Quantif. Method:	**Zhang Xuexin**	*Dilution Factor:*	**1.0000**
Run Time (min):	**36.91**	*Sample Amount:*	**1.0000**

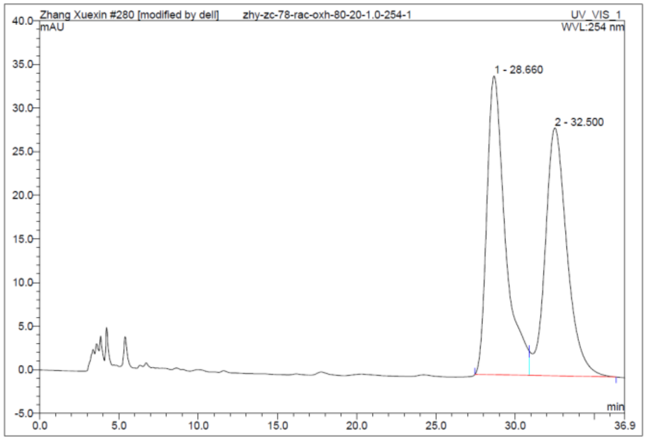

No.	Ret.Time min	Peak Name	Height mAU	Area mAU*min	Rel.Area %	Amount	Type
1	28.66	n.a.	34.234	46.206	50.45	n.a.	BM *
2	32.50	n.a.	28.418	45.376	49.55	n.a.	MB*
Total:			62.651	91.583	100.00	0.000	

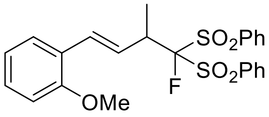

***rac*-3e**

Mu/Integration

Chromeleon (c) Dionex 1996-2006
Version 6.80 SR8a Build 2643 (158225)

Operator:dell Timebase:U-3000 Sequence:Zhang Xuexin

Page 1-1
2022-1-13 11:05 上午

281 zhy-zc-78-asy-oxh-80-20-1.0-254-1

Sample Name:	**zhy-zc-78-asy-oxh-80-20-1.0-254-1**	*Injection Volume:*	**20.0**
Vial Number:	**379**	*Channel:*	**UV_VIS_1**
Sample Type:	**standard**	*Wavelength:*	**254**
Control Program:	**Zhang Xuexin**	*Bandwidth:*	**n.a.**
Quantif. Method:	**Zhang Xuexin**	*Dilution Factor:*	**1.0000**
Run Time (min):	**37.29**	*Sample Amount:*	**1.0000**

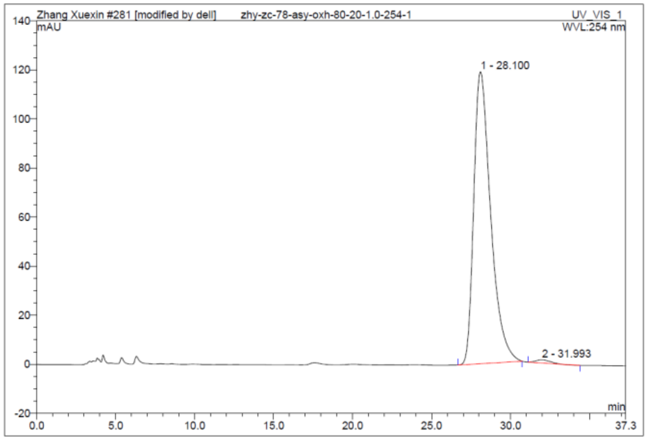

No.	Ret.Time min	Peak Name	Height mAU	Area mAU*min	Rel.Area %	Amount	Type
1	28.10	n.a.	118.993	147.530	99.12	n.a.	BMB*
2	31.99	n.a.	1.258	1.311	0.88	n.a.	BMB*
Total:			120.251	148.840	100.00	0.000	

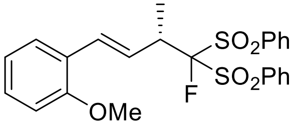

3e

Mu/Integration

Chromeleon (c) Dionex 1996-2006
Version 6.80 SR8a Build 2643 (158225)

Operator:dell Timebase:U-3000 Sequence:Zhang Xuexin
Page 1-1
2008-8-20 12:14 下午

257 ZHY-ZC-106-RAC-OXH-80-20-1.0-254-1

Sample Name:	**ZHY-ZC-106-RAC-OXH-80-20-1.0-254-1**	*Injection Volume:*	**20.0**
Vial Number:	**355**	*Channel:*	**UV_VIS_1**
Sample Type:	**standard**	*Wavelength:*	**254**
Control Program:	**Zhang Xuexin**	*Bandwidth:*	**n.a.**
Quantif. Method:	**Zhang Xuexin**	*Dilution Factor:*	**1.0000**
Run Time (min):	**49.07**	*Sample Amount:*	**1.0000**

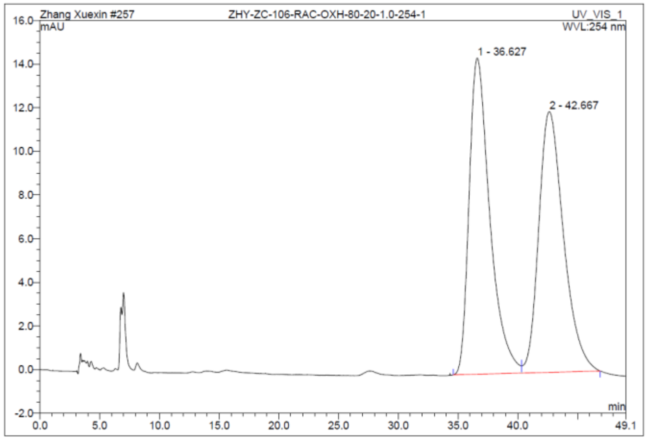

No.	Ret.Time min	Peak Name	Height mAU	Area mAU*min	Rel.Area %	Amount	Type
1	36.63	n.a.	14.490	29.183	50.28	n.a.	BM
2	42.67	n.a.	11.955	28.860	49.72	n.a.	MB
Total:			26.444	58.043	100.00	0.000	

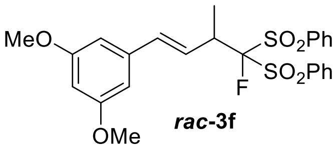

***rac*-3f**

Mu/Integration
Chromeleon (c) Dionex 1996-2006
Version 6.80 SR8a Build 2643 (158225)

Operator:dell Timebase:U-3000 Sequence:Zhang Xuexin
Page 1-1
2008-8-20 12:17 下午

258 ZHY-ZC-106-ASY-OXH-80-20-1.0-254-1

Sample Name:	**ZHY-ZC-106-ASY-OXH-80-20-1.0-254-1**	*Injection Volume:*	**20.0**
Vial Number:	**356**	*Channel:*	**UV_VIS_1**
Sample Type:	**standard**	*Wavelength:*	**254**
Control Program:	**Zhang Xuexin**	*Bandwidth:*	**n.a.**
Quantif. Method:	**Zhang Xuexin**	*Dilution Factor:*	**1.0000**
Run Time (min):	**54.31**	*Sample Amount:*	**1.0000**

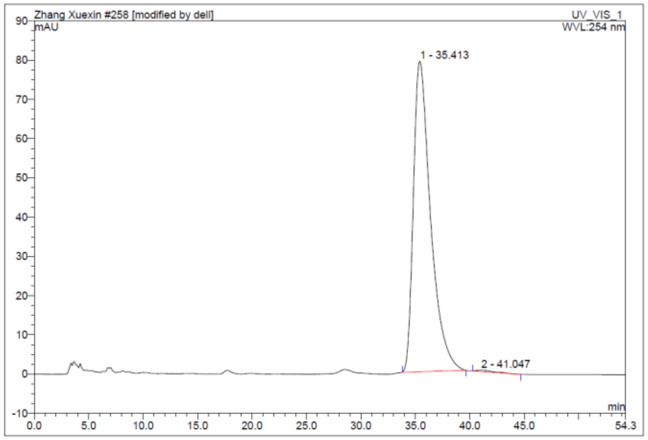

No.	Ret.Time min	Peak Name	Height mAU	Area mAU*min	Rel.Area %	Amount	Type
1	35.41	n.a.	79.060	144.461	99.57	n.a.	BMB*
2	41.05	n.a.	0.353	0.618	0.43	n.a.	BMB*
Total:			79.414	145.079	100.00	0.000	

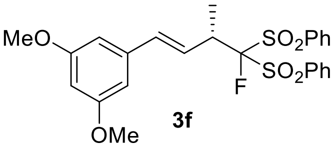

3f

Mu/Integration
Chromeleon (c) Dionex 1996-2006
Version 6.80 SR8a Build 2643 (158225)

Analysis Report

<Sample Information>

Sample Name : zhy-zf-65-1-rac-oxh-80-20-1.0-254-205-5
Sample ID :
Data Filename : zhy-zf-65-1-rac-oxh-80-20-1.0-254-205-5.lcd
Method Filename : 1.0.lcm
Batch Filename :
Vial # : 1-1
Injection Volume : 20 uL
Date Acquired : 2022/8/29 10:50:21
Date Processed : 2022/8/29 14:21:43
Sample Type : Unknown
Acquired by : System Administrator
Processed by : System Administrator

<Chromatogram>

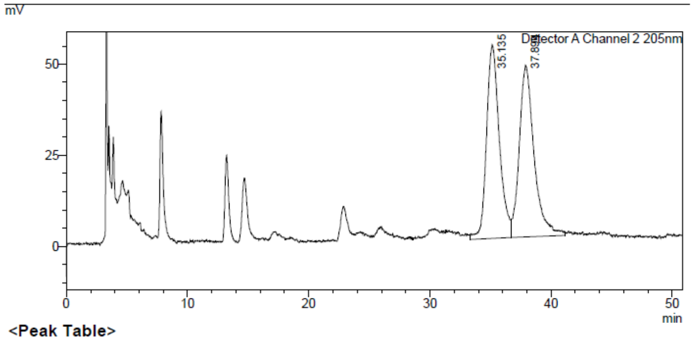

<Peak Table>

Detector A Channel 2 205nm

Peak#	Ret. Time	Area	Height	Conc.
1	35.135	3914565	53210	49.501
2	37.894	3993556	47111	50.499
Total		7908121	100320	

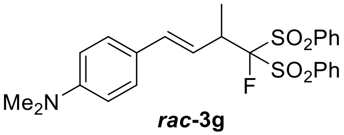

***rac*-3g**

Analysis Report

<Sample Information>

Sample Name : zhy-zf-67-1-asy-oxh-80-20-1.0-254-205-5
Sample ID :
Data Filename : zhy-zf-67-1-asy-oxh-80-20-1.0-254-205-5.lcd
Method Filename : 1.0.lcm
Batch Filename :
Vial # : 1-1
Injection Volume : 20 uL
Date Acquired : 2022/8/29 11:42:50
Date Processed : 2022/8/29 13:22:51
Sample Type : Unknown
Acquired by : System Administrator
Processed by : System Administrator

<Chromatogram>

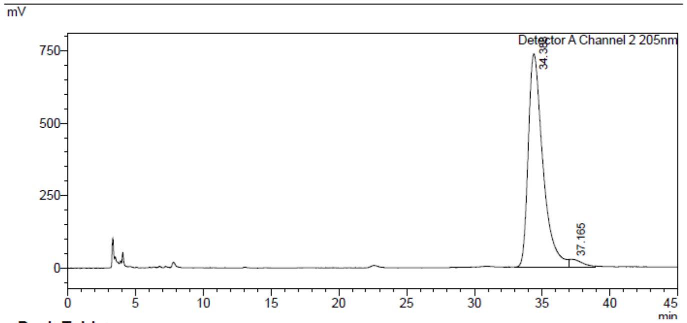

<Peak Table>

Detector A Channel 2 205nm

Peak#	Ret. Time	Area	Height	Conc.
1	34.388	55432822	735811	96.709
2	37.165	1886518	27511	3.291
Total		57319340	763322	

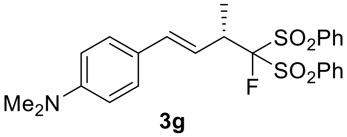

3g

2022/8/22 15:49:29 Page 1 / 1

Analysis Report

<Sample Information>

Sample Name	: zhy-zf-65-3-rac-oxh-80-20-1.0-254-230-2		
Sample ID	:		
Data Filename	: zhy-zf-65-3-rac-oxh-80-20-1.0-254-230-2.lcd		
Method Filename	: 1.0.lcm		
Batch Filename	:		
Vial #	: 1-1	Sample Type	: Unknown
Injection Volume	: 20 uL		
Date Acquired	: 2022/8/19 18:38:40	Acquired by	: System Administrator
Date Processed	: 2022/8/19 19:24:29	Processed by	: System Administrator

<Chromatogram>

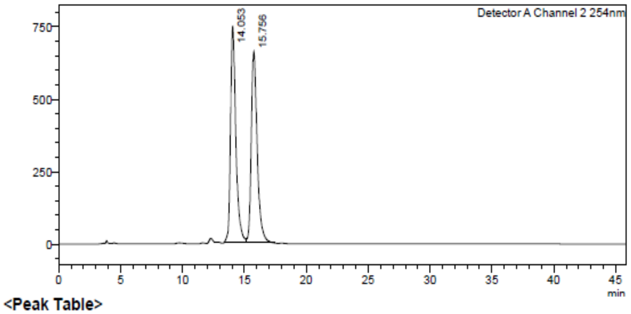

<Peak Table>

Detector A Channel 2 254nm

Peak#	Ret. Time	Area	Height	Conc.
1	14.053	22476758	746773	50.378
2	15.756	22139355	659977	49.622
Total		44616113	1406751	

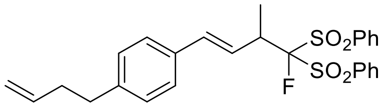

***rac*-3h**

D:\Data\ZHY\ZF\zhy-zf-65-3-rac-oxh-80-20-1.0-254-230-2.lcd

2022/8/22 15:49:46 Page 1 / 1

Analysis Report

<Sample Information>

Sample Name	: zhy-zf-67-4-asy-oxh-80-20--1.0-254-2		
Sample ID	:		
Data Filename	: zhy-zf-67-4-asy-oxh-80-20--1.0-254-2.lcd		
Method Filename	: 1.0.lcm		
Batch Filename	:		
Vial #	: 1-1	Sample Type	: Unknown
Injection Volume	: 20 uL		
Date Acquired	: 2022/8/22 15:27:14	Acquired by	: System Administrator
Date Processed	: 2022/8/22 15:45:19	Processed by	: System Administrator

<Chromatogram>

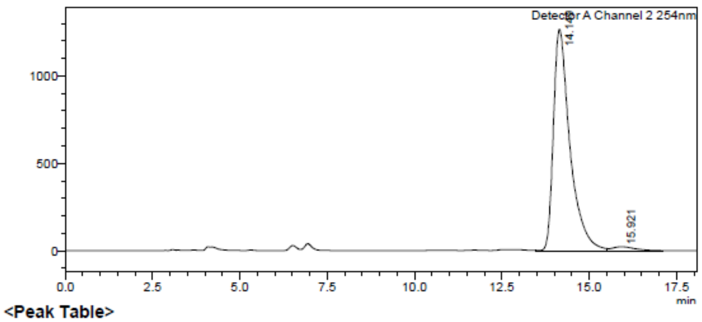

<Peak Table>

Detector A Channel 2 254nm

Peak#	Ret. Time	Area	Height	Conc.
1	14.149	41695651	1265016	97.623
2	15.921	1015371	22037	2.377
Total		42711022	1287053	

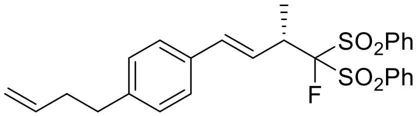

3h

D:\Data\ZHY\ZF\zhy-zf-67-4-asy-oxh-80-20--1.0-254-2.lcd

Analysis Report

<Sample Information>

Sample Name	: zhy-zc-96-rac-oxh-85-15-1.0-254-205-1		
Sample ID	:		
Data Filename	: zhy-zc-96-rac-oxh-85-15-1.0-254-205-1.lcd		
Method Filename	: 1.0.lcm		
Batch Filename	:		
Vial #	: 1-1	Sample Type	: Unknown
Injection Volume	: 20 uL		
Date Acquired	: 2021/10/29 11:46:10	Acquired by	: System Administrator
Date Processed	: 2021/10/29 12:06:55	Processed by	: System Administrator

<Chromatogram>

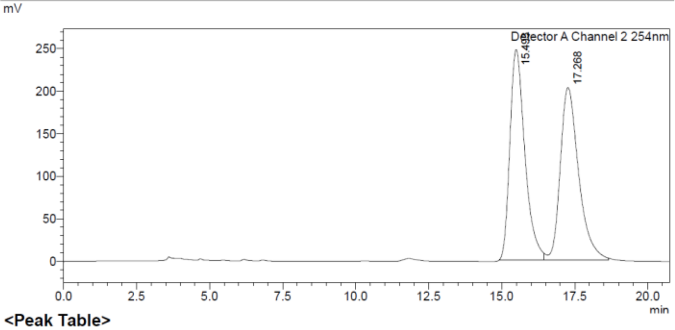

<Peak Table>

Detector A Channel 2 254nm

Peak#	Ret. Time	Area	Height	Conc.
1	15.493	8811811	247486	49.746
2	17.268	8901829	202826	50.254
Total		17713640	450312	

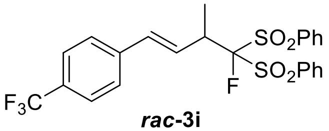

***rac*-3i**

Analysis Report

<Sample Information>

Sample Name	: zhy-zc-96-asy-oxh-85-15-1.0-254-205-1		
Sample ID	:		
Data Filename	: zhy-zc-96-asy-oxh-85-15-1.0-254-205-1.lcd		
Method Filename	: 1.0.lcm		
Batch Filename	:		
Vial #	: 1-1	Sample Type	: Unknown
Injection Volume	: 20 uL		
Date Acquired	: 2021/10/29 12:07:34	Acquired by	: System Administrator
Date Processed	: 2021/10/29 12:43:27	Processed by	: System Administrator

<Chromatogram>

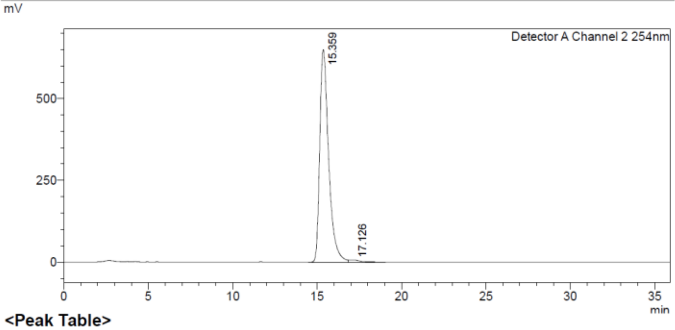

<Peak Table>

Detector A Channel 2 254nm

Peak#	Ret. Time	Area	Height	Conc.
1	15.359	23761739	650345	98.643
2	17.126	326780	6458	1.357
Total		24088519	656804	

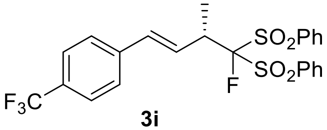

3i

2022/8/25 10:45:32 Page 1 / 1

Analysis Report

<Sample Information>

Sample Name	: zhy-zf-65-5-rac-adh-80-20-1.0-254-1		
Sample ID	:		
Data Filename	: zhy-zf-65-5-rac-adh-80-20-1.0-254-1.lcd		
Method Filename	: 1.0.lcm		
Batch Filename	:		
Vial #	: 1-1	Sample Type	: Unknown
Injection Volume	: 20 uL		
Date Acquired	: 2022/8/25 9:27:28	Acquired by	: System Administrator
Date Processed	: 2022/8/25 10:14:21	Processed by	: System Administrator

<Chromatogram>

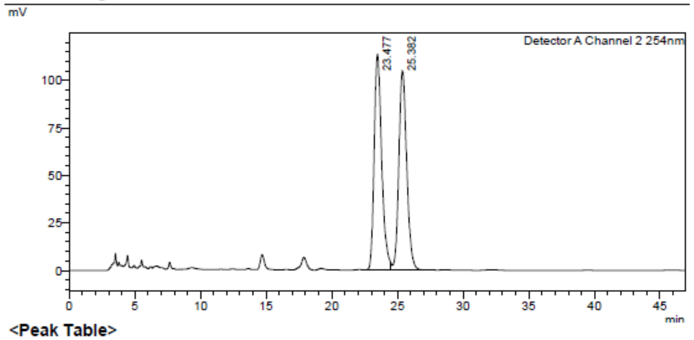

<Peak Table>

Detector A Channel 2 254nm

Peak#	Ret. Time	Area	Height	Conc.
1	23.477	4500363	113307	50.747
2	25.382	4367892	104606	49.253
Total		8868255	217913	

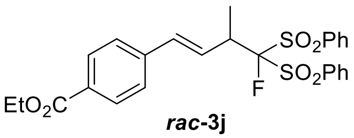

***rac*-3j**

D:\Data\ZHY\ZF\zhy-zf-65-5-rac-adh-80-20-1.0-254-1.lcd

2022/8/25 10:44:40 Page 1 / 1

Analysis Report

<Sample Information>

Sample Name	: zhy-zf-69-2-asy-adh-80-20-1.0-254-1		
Sample ID	:		
Data Filename	: zhy-zf-69-2-asy-adh-80-20-1.0-254-1.lcd		
Method Filename	: 1.0.lcm		
Batch Filename	:		
Vial #	: 1-1	Sample Type	: Unknown
Injection Volume	: 20 uL		
Date Acquired	: 2022/8/25 8:54:41	Acquired by	: System Administrator
Date Processed	: 2022/8/25 9:26:06	Processed by	: System Administrator

<Chromatogram>

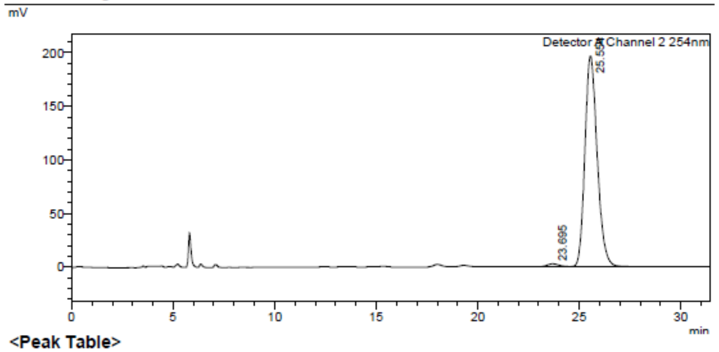

<Peak Table>

Detector A Channel 2 254nm

Peak#	Ret. Time	Area	Height	Conc.
1	23.695	110795	2873	1.320
2	25.554	8280913	196352	98.680
Total		8391708	199225	

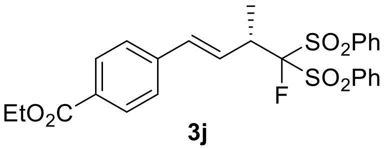

3j

D:\Data\ZHY\ZF\zhy-zf-69-2-asy-adh-80-20-1.0-254-1.lcd

2022/8/26 15:29:33 Page 1 / 1

Analysis Report

<Sample Information>

Sample Name : ZHY-ZF-67-5-RAC-ODH-80-20-1.0-254-230-7
Sample ID :
Data Filename : ZHY-ZF-67-5-RAC-ODH-80-20-1.0-254-230-7.lcd
Method Filename : 1.0.lcm
Batch Filename :
Vial # : 1-1 Sample Type : Unknown
Injection Volume : 20 uL
Date Acquired : 2022/8/26 11:19:07 Acquired by : System Administrator
Date Processed : 2022/8/26 12:02:13 Processed by : System Administrator

<Chromatogram>

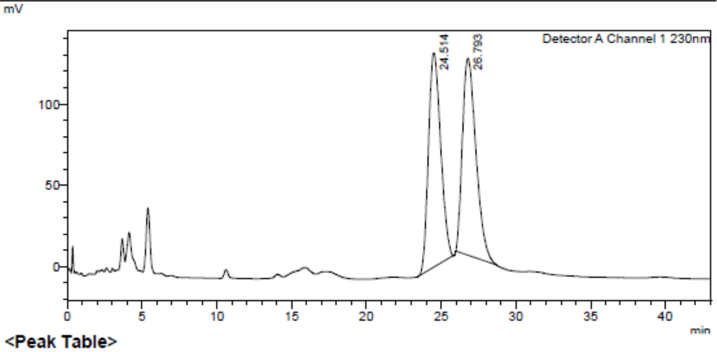

<Peak Table>

Detector A Channel 2 254nm

Peak#	Ret. Time	Area	Height	Conc.
1	24.512	1997093	33900	50.295
2	26.794	1973629	31048	49.705
Total		3970722	64948	

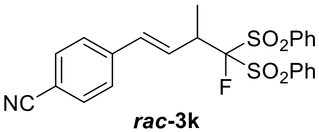

***rac*-3k**

D:\Data\ZHY\ZF\ZHY-ZF-67-5-RAC-ODH-80-20-1.0-254-230-7.lcd

2022/8/27 17:59:49 Page 1 / 1

Analysis Report

<Sample Information>

Sample Name : ZHY-ZF-67-5-ASY-ODH-80-20-1.0-254-230-1
Sample ID :
Data Filename : ZHY-ZF-67-5-ASY-ODH-80-20-1.0-254-230-1.lcd
Method Filename : 1.0.lcm
Batch Filename :
Vial # : 1-1 Sample Type : Unknown
Injection Volume : 20 uL
Date Acquired : 2022/8/25 20:21:32 Acquired by : System Administrator
Date Processed : 2022/8/25 20:51:43 Processed by : System Administrator

<Chromatogram>

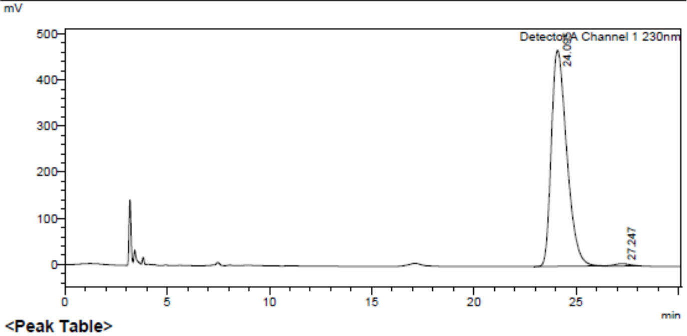

<Peak Table>

Detector A Channel 1 230nm

Peak#	Ret. Time	Area	Height	Conc.
1	24.095	24589820	468056	98.832
2	27.247	290526	5120	1.168
Total		24880346	473176	

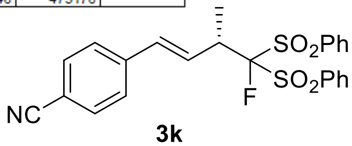

3k

D:\Data\ZHY\ZF\ZHY-ZF-67-5-ASY-ODH-80-20-1.0-254-230-1.lcd

2022/8/24 21:40:40 Page 1 / 1

Analysis Report

<Sample Information>

Sample Name	: zhy-zf-65-4-rac-oxh-60-40-1.0-254-230-2		
Sample ID	:		
Data Filename	: zhy-zf-65-4-rac-oxh-60-40-1.0-254-230-2.lcd		
Method Filename	: 1.0.lcm		
Batch Filename	:		
Vial #	: 1-1	Sample Type	: Unknown
Injection Volume	: 20 uL		
Date Acquired	: 2022/8/19 22:02:17	Acquired by	: System Administrator
Date Processed	: 2022/8/19 22:38:53	Processed by	: System Administrator

<Chromatogram>

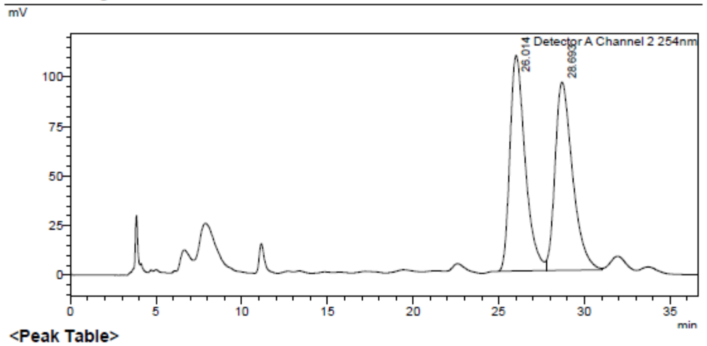

<Peak Table>

Detector A Channel 2 254nm

Peak#	Ret. Time	Area	Height	Conc.
1	26.014	6757957	108828	49.989
2	28.693	6760871	95061	50.011
Total		13518827	203889	

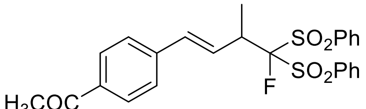

***rac*-3l**

D:\Data\ZHY\ZF\zhy-zf-65-4-rac-oxh-60-40-1.0-254-230-2.lcd

2022/8/26 15:34:06 Page 1 / 1

Analysis Report

<Sample Information>

Sample Name	: zhy-zf-67-3-asy-oxh-60-40--1.0-254-2		
Sample ID	:		
Data Filename	: zhy-zf-67-3-asy-oxh-60-40--1.0-254-2.lcd		
Method Filename	: 1.0.lcm		
Batch Filename	:		
Vial #	: 1-1	Sample Type	: Unknown
Injection Volume	: 20 uL		
Date Acquired	: 2022/8/22 17:59:23	Acquired by	: System Administrator
Date Processed	: 2022/8/24 21:41:11	Processed by	: System Administrator

<Chromatogram>

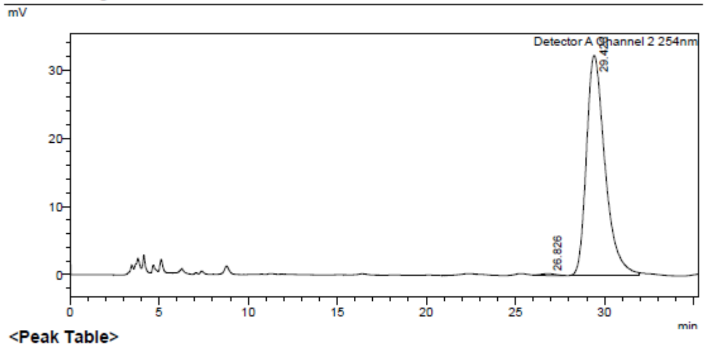

<Peak Table>

Detector A Channel 2 254nm

Peak#	Ret. Time	Area	Height	Conc.
1	26.826	17390	295	0.734
2	29.426	2351375	32314	99.266
Total		2368765	32609	

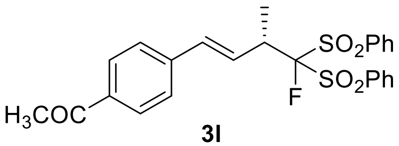

3l

D:\Data\ZHY\ZF\zhy-zf-67-3-asy-oxh-60-40--1.0-254-2.lcd

2022/8/25 19:57:13 Page 1 / 1

Analysis Report

<Sample Information>

Sample Name : ZHY-ZF-65-2-ASY-ODH-80-20-1.0-254-230-1
Sample ID :
Data Filename : ZHY-ZF-65-2-ASY-ODH-80-20-1.0-254-230-1.lcd
Method Filename : 1.0.lcm
Batch Filename :
Vial # : 1-1 Sample Type : Unknown
Injection Volume : 20 uL
Date Acquired : 2022/8/25 17:57:38 Acquired by : System Administrator
Date Processed : 2022/8/25 19:53:02 Processed by : System Administrator

<Chromatogram>

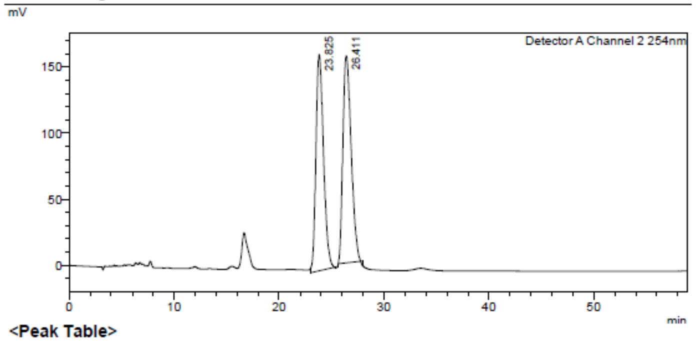

<Peak Table>

Detector A Channel 2 254nm

Peak#	Ret. Time	Area	Height	Conc.
1	23.825	8217558	163776	48.923
2	26.411	8579308	156993	51.077
Total		16796866	320769	

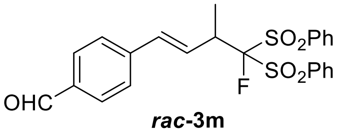

***rac*-3m**

D:\Data\ZHY\ZF\ZHY-ZF-65-2-ASY-ODH-80-20-1.0-254-230-1.lcd

2022/8/25 19:52:06 Page 1 / 1

Analysis Report

<Sample Information>

Sample Name : ZHY-ZF-69-1-ASY-ODH-80-20-1.0-254-230-1
Sample ID :
Data Filename : ZHY-ZF-69-1-ASY-ODH-80-20-1.0-254-230-1.lcd
Method Filename : 1.0.lcm
Batch Filename :
Vial # : 1-1 Sample Type : Unknown
Injection Volume : 20 uL
Date Acquired : 2022/8/25 18:57:11 Acquired by : System Administrator
Date Processed : 2022/8/25 19:47:07 Processed by : System Administrator

<Chromatogram>

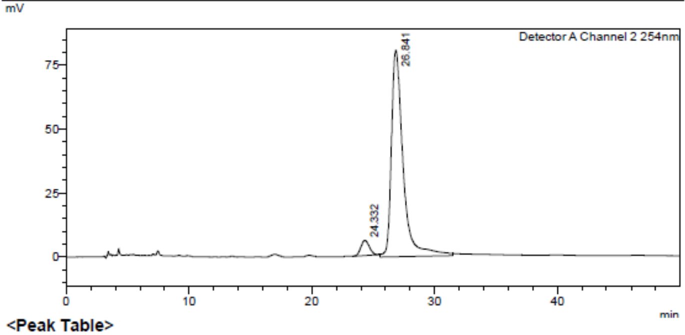

<Peak Table>

Detector A Channel 2 254nm

Peak#	Ret. Time	Area	Height	Conc.
1	24.332	286764	5929	5.063
2	26.841	5376784	80813	94.937
Total		5663548	86742	

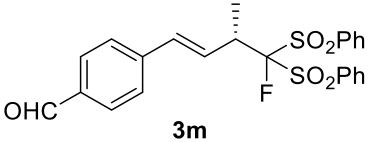

3m

D:\Data\ZHY\ZF\ZHY-ZF-69-1-ASY-ODH-80-20-1.0-254-230-1.lcd

Analysis Report

<Sample Information>

Sample Name	: zhy-zc-105-rac-oxh-80-20-1.0-254-205-2
Sample ID	:
Data Filename	: zhy-zc-105-rac-oxh-80-20-1.0-254-205-2.lcd
Method Filename	: 1.0.lcm
Batch Filename	:
Vial #	: 1-1
Injection Volume	: 20 uL
Date Acquired	: 2021/10/29 17:02:49
Date Processed	: 2021/10/29 17:32:24
Sample Type	: Unknown
Acquired by	: System Administrator
Processed by	: System Administrator

<Chromatogram>

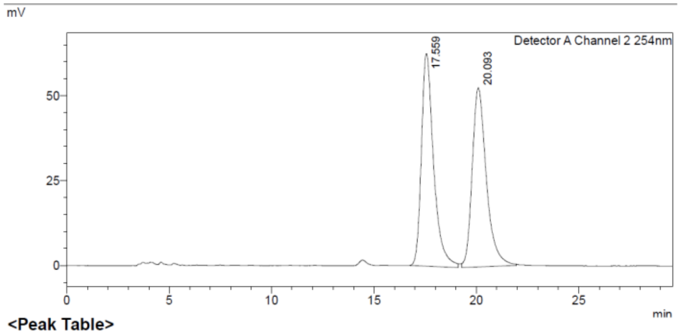

<Peak Table>

Detector A Channel 2 254nm

Peak#	Ret. Time	Area	Height	Conc.
1	17.559	2593355	62587	50.522
2	20.093	2539787	52683	49.478
Total		5133142	115270	

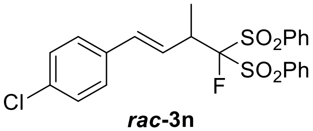

***rac*-3n**

D:\Data\ZHY\ZC\zhy-zc-105-rac-oxh-80-20-1.0-254-205-2.lcd

Analysis Report

<Sample Information>

Sample Name	: zhy-zc-105-asy-oxh-80-20-1.0-254-205-2
Sample ID	:
Data Filename	: zhy-zc-105-asy-oxh-80-20-1.0-254-205-2.lcd
Method Filename	: 1.0.lcm
Batch Filename	:
Vial #	: 1-1
Injection Volume	: 20 uL
Date Acquired	: 2021/10/29 16:34:18
Date Processed	: 2021/10/29 17:01:41
Sample Type	: Unknown
Acquired by	: System Administrator
Processed by	: System Administrator

<Chromatogram>

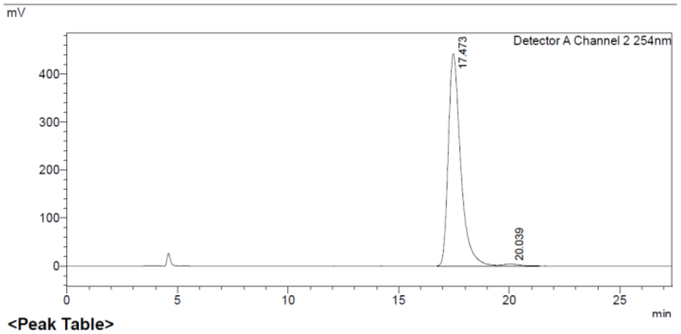

<Peak Table>

Detector A Channel 2 254nm

Peak#	Ret. Time	Area	Height	Conc.
1	17.473	17147412	442070	98.619
2	20.039	240159	4498	1.381
Total		17387571	446569	

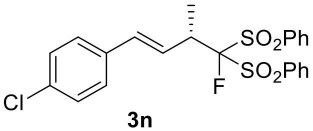

3n

D:\Data\ZHY\ZC\zhy-zc-105-asy-oxh-80-20-1.0-254-205-2.lcd

Operator:dell Timebase:U-3000 Sequence:Zhang Xuexin

Page 1-1
2008-8-21 1:51 上午

267 ZHY-Zd-21-2-RAC-OXH-80-20-1.0-205-1

Sample Name:	**ZHY-Zd-21-2-RAC-OXH-80-20-1.0-205-1**	*Injection Volume:*	**20.0**
Vial Number:	**365**	*Channel:*	**UV_VIS_1**
Sample Type:	**standard**	*Wavelength:*	**205**
Control Program:	**Zhang Xuexin**	*Bandwidth:*	**n.a.**
Quantif. Method:	**Zhang Xuexin**	*Dilution Factor:*	**1.0000**
Run Time (min):	**26.37**	*Sample Amount:*	**1.0000**

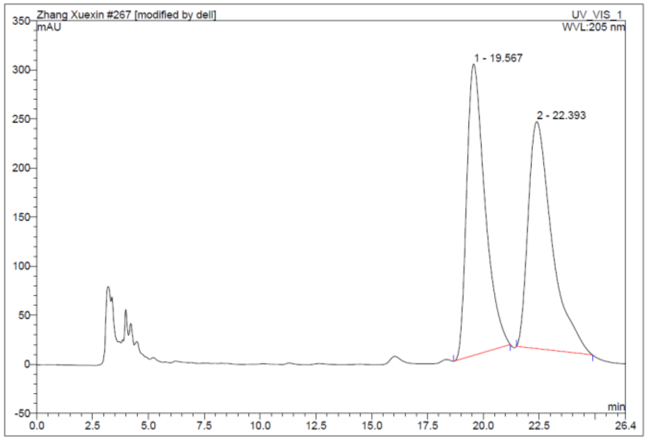

No.	Ret.Time min	Peak Name	Height mAU	Area mAU*min	Rel.Area %	Amount	Type
1	19.57	n.a.	296.777	281.422	49.56	n.a.	BMB*
2	22.39	n.a.	231.145	286.457	50.44	n.a.	BMB*
**Total:**			527.923	567.879	100.00	0.000	

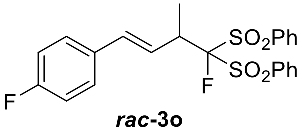

***rac*-3o**

Mu/Integration

Chromeleon (c) Dionex 1996-2006
Version 6.80 SR8a Build 2643 (158225)

Operator:dell Timebase:U-3000 Sequence:Zhang Xuexin

Page 1-1
2008-8-21 1:52 上午

264 ZHY-Zd-21-2-ASY-OXH-80-20-1.0-254-1

Sample Name:	**ZHY-Zd-21-2-ASY-OXH-80-20-1.0-254-1**	*Injection Volume:*	**20.0**
Vial Number:	**362**	*Channel:*	**UV_VIS_1**
Sample Type:	**standard**	*Wavelength:*	**205**
Control Program:	**Zhang Xuexin**	*Bandwidth:*	**n.a.**
Quantif. Method:	**Zhang Xuexin**	*Dilution Factor:*	**1.0000**
Run Time (min):	**24.48**	*Sample Amount:*	**1.0000**

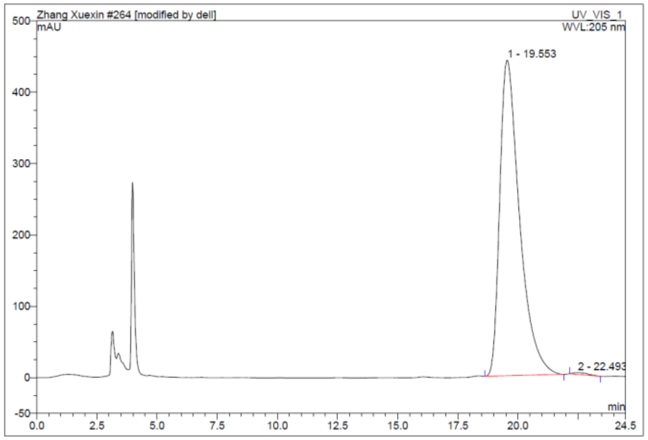

No.	Ret.Time min	Peak Name	Height mAU	Area mAU*min	Rel.Area %	Amount	Type
1	19.55	n.a.	442.077	421.641	99.54	n.a.	BMB
2	22.49	n.a.	2.613	1.938	0.46	n.a.	BMB*
**Total:**			444.690	423.579	100.00	0.000	

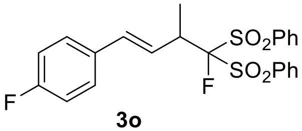

3o

Mu/Integration

Chromeleon (c) Dionex 1996-2006
Version 6.80 SR8a Build 2643 (158225)

Analysis Report

<Sample Information>

Sample Name	: zhy-zc-23-1-rac-oxh-0.5-254nm-01		
Sample ID	:		
Data Filename	: zhy-zc-23-1-rac-oxh-0.5-254nm-01.lcd		
Method Filename	: 1.0.lcm		
Batch Filename	:		
Vial #	: 1-1	Sample Type	: Unknown
Injection Volume	: 20 uL		
Date Acquired	: 2021/9/16 16:47:58	Acquired by	: System Administrator
Date Processed	: 2021/9/16 17:16:13	Processed by	: System Administrator

<Chromatogram>

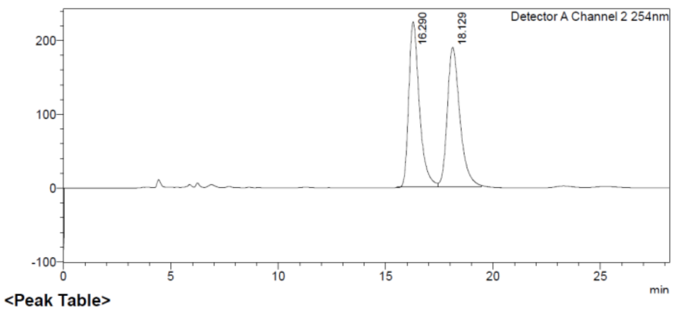

<Peak Table>

Detector A Channel 2 254nm

Peak#	Ret. Time	Area	Height	Conc.
1	16.290	7718407	223972	49.767
2	18.129	7790641	189374	50.233
Total		15509048	413346	

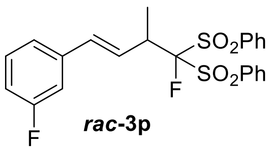

***rac*-3p**

Analysis Report

<Sample Information>

Sample Name	: zhy-zd-21-asy-oxh-80-20-1.0-254-230-2		
Sample ID	:		
Data Filename	: zhy-zd-21-asy-oxh-80-20-1.0-254-230-2.lcd		
Method Filename	: 1.0.lcm		
Batch Filename	:		
Vial #	: 1-1	Sample Type	: Unknown
Injection Volume	: 20 uL		
Date Acquired	: 2021/12/10 17:28:34	Acquired by	: System Administrator
Date Processed	: 2021/12/10 20:02:44	Processed by	: System Administrator

<Chromatogram>

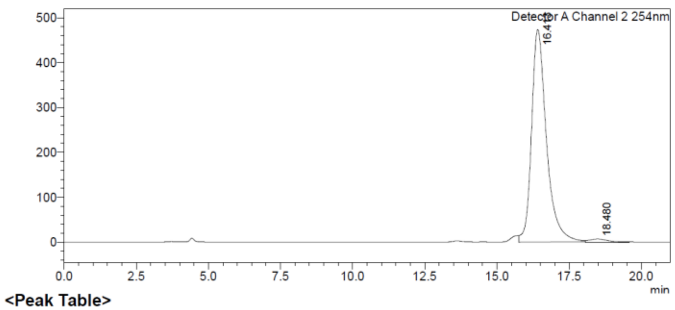

<Peak Table>

Detector A Channel 2 254nm

Peak#	Ret. Time	Area	Height	Conc.
1	16.413	17010065	473420	98.242
2	18.480	304355	6973	1.758
Total		17314420	480392	

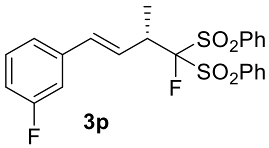

3p

Operator:dell Timebase:U-3000 Sequence:Zhang Xuexin

Page 1-1
2008-8-21 7:45 上午

275 ZHY-Zd-17-rac-OXH-95-5-1.0-205-3

Sample Name:	**ZHY-Zd-17-rac-OXH-95-5-1.0-205-3**	*Injection Volume:*	**20.0**
Vial Number:	**373**	*Channel:*	**UV_VIS_1**
Sample Type:	**standard**	*Wavelength:*	**205**
Control Program:	**Zhang Xuexin**	*Bandwidth:*	**n.a.**
Quantif. Method:	**Zhang Xuexin**	*Dilution Factor:*	**1.0000**
Run Time (min):	**24.93**	*Sample Amount:*	**1.0000**

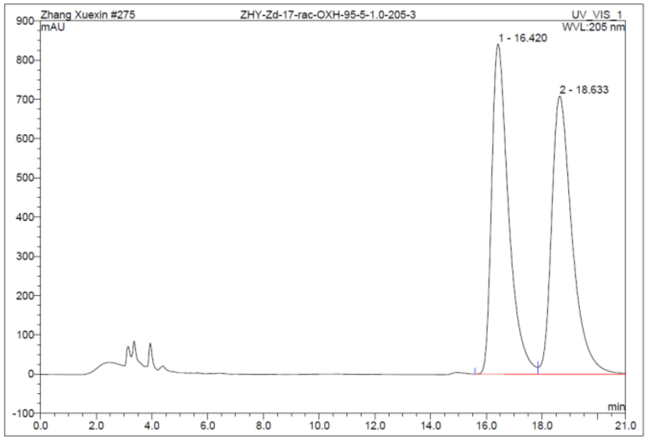

No.	Ret.Time min	Peak Name	Height mAU	Area mAU*min	Rel.Area %	Amount	Type
1	16.42	n.a.	840.926	590.824	47.64	n.a.	BM
2	18.63	n.a.	708.380	604.343	48.73	n.a.	M
3	22.14	n.a.	51.278	45.081	3.63	n.a.	MB
Total:			1600.584	1240.247	100.00	0.000	

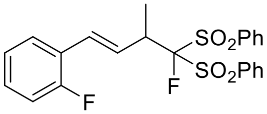

***rac*-3q**

Mu/Integration

Chromeleon (c) Dionex 1996-2006
Version 6.80 SR8a Build 2643 (158225)

Operator:dell Timebase:U-3000 Sequence:Zhang Xuexin

Page 1-1
2008-8-21 7:44 上午

276 ZHY-Zd-17-asy-OXH-95-5-1.0-205-3

Sample Name:	**ZHY-Zd-17-asy-OXH-95-5-1.0-205-3**	*Injection Volume:*	**20.0**
Vial Number:	**374**	*Channel:*	**UV_VIS_1**
Sample Type:	**standard**	*Wavelength:*	**205**
Control Program:	**Zhang Xuexin**	*Bandwidth:*	**n.a.**
Quantif. Method:	**Zhang Xuexin**	*Dilution Factor:*	**1.0000**
Run Time (min):	**60.00**	*Sample Amount:*	**1.0000**

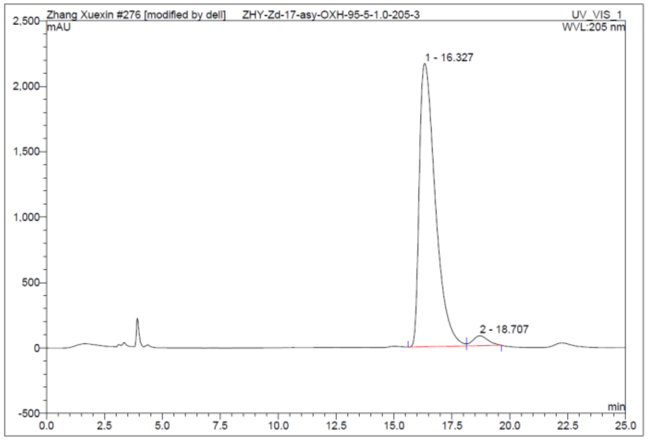

No.	Ret.Time min	Peak Name	Height mAU	Area mAU*min	Rel.Area %	Amount	Type
1	16.33	n.a.	2165.153	1735.145	96.83	n.a.	BM *
2	18.71	n.a.	75.066	56.743	3.17	n.a.	MB*
Total:			2240.220	1791.888	100.00	0.000	

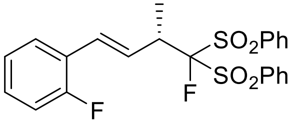

3q

Mu/Integration

Chromeleon (c) Dionex 1996-2006
Version 6.80 SR8a Build 2643 (158225)

Analysis Report

<Sample Information>

Sample Name : zhy-zf-53-2-rac-oxh-80-20--1.0-254-2
Sample ID :
Data Filename : zhy-zf-53-2-rac-oxh-80-20--1.0-254-2.lcd
Method Filename : 1.0.lcm
Batch Filename :
Vial # : 1-1 Sample Type : Unknown
Injection Volume : 20 uL
Date Acquired : 2022/8/22 14:46:17 Acquired by : System Administrator
Date Processed : 2022/8/23 12:02:43 Processed by : System Administrator

<Chromatogram>

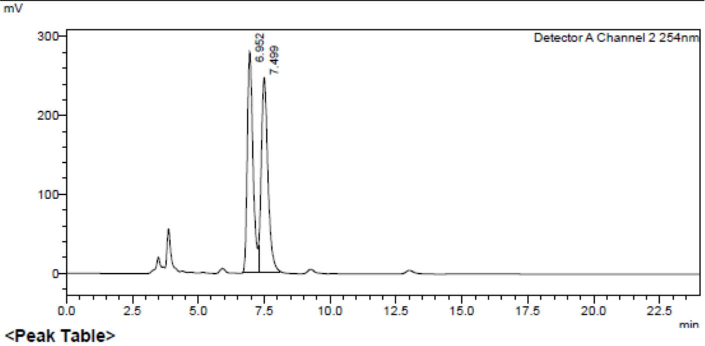

<Peak Table>

Detector A Channel 2 254nm

Peak#	Ret. Time	Area	Height	Conc.
1	6.952	4191234	280479	49.122
2	7.499	4340988	247604	50.878
Total		8532222	528083	

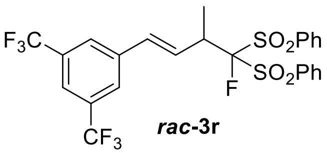

***rac*-3r**

Analysis Report

<Sample Information>

Sample Name : zhy-zf-67-2-asy-oxh-80-20-1.0-254-230-3
Sample ID :
Data Filename : zhy-zf-67-2-asy-oxh-80-20-1.0-254-230-3.lcd
Method Filename : 1.0.lcm
Batch Filename :
Vial # : 1-1 Sample Type : Unknown
Injection Volume : 20 uL
Date Acquired : 2022/8/23 11:51:26 Acquired by : System Administrator
Date Processed : 2022/8/23 12:01:28 Processed by : System Administrator

<Chromatogram>

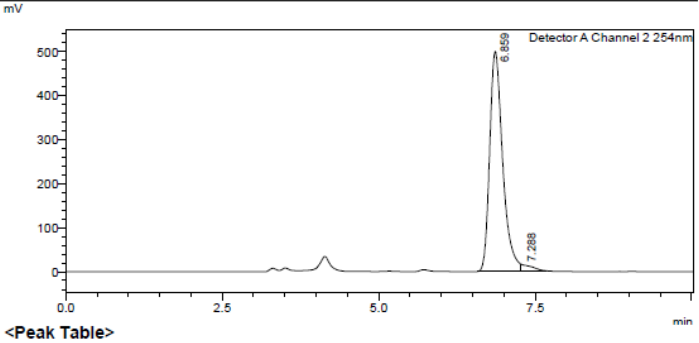

<Peak Table>

Detector A Channel 2 254nm

Peak#	Ret. Time	Area	Height	Conc.
1	6.859	7187831	498658	97.664
2	7.288	171939	14528	2.336
Total		7359771	513186	

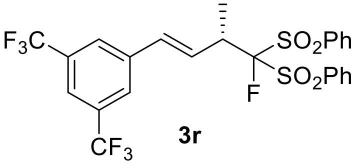

3r

2022/1/11 21:48:52 Page 1 / 1

Analysis Report

<Sample Information>

Sample Name	: zhy-zB-141-2-rac--oXH-80-20-1.0-254nm-1		
Sample ID	:		
Data Filename	: zhy-zB-141-2-rac--oXH-80-20-1.0-254nm-1.lcd		
Method Filename	: 1.0.lcm		
Batch Filename	:		
Vial #	: 1-1	Sample Type	: Unknown
Injection Volume	: 20 uL		
Date Acquired	: 2021/9/7 10:53:58	Acquired by	: System Administrator
Date Processed	: 2022/1/10 21:47:58	Processed by	: System Administrator

<Chromatogram>

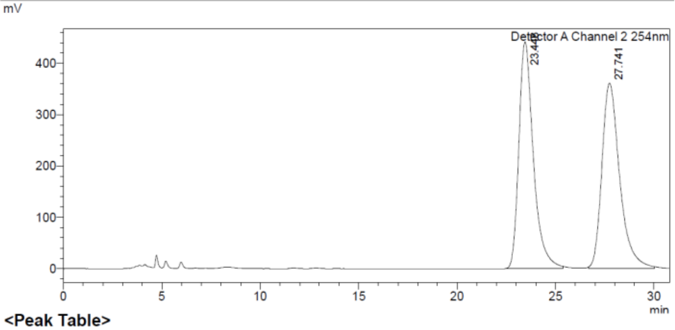

<Peak Table>

Detector A Channel 2 254nm

Peak#	Ret. Time	Area	Height	Conc.
1	23.448	22601474	441137	49.934
2	27.741	22660974	361293	50.066
Total		45262449	802430	

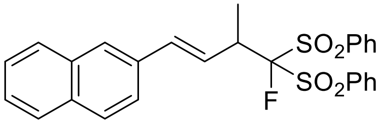

***rac*-3s**

D:\Data\ZHY\ZB\zhy-zB-141-2-rac--oXH-80-20-1.0-254nm-1.lcd

2022/1/10 21:47:25 Page 1 / 1

Analysis Report

<Sample Information>

Sample Name	: zhy-zc-26-1-asy-ox-h-80-20-1-254nm-1		
Sample ID	:		
Data Filename	: zhy-zc-26-1-asy-ox-h-80-20-1-254nm-1.lcd		
Method Filename	: 1.0.lcm		
Batch Filename	:		
Vial #	: 1-1	Sample Type	: Unknown
Injection Volume	: 20 uL		
Date Acquired	: 2021/10/1 15:05:39	Acquired by	: System Administrator
Date Processed	: 2021/10/1 15:40:00	Processed by	: System Administrator

<Chromatogram>

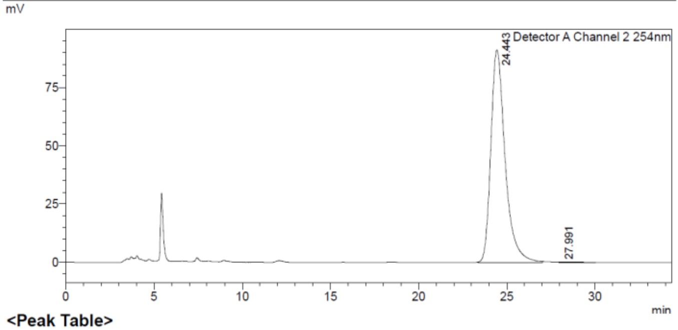

<Peak Table>

Detector A Channel 2 254nm

Peak#	Ret. Time	Area	Height	Conc.
1	24.443	5124014	91223	99.859
2	27.991	7219	89	0.141
Total		5131233	91312	

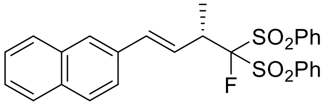

3s

D:\Data\ZHY\ZC\zhy-zc-26-1-asy-ox-h-80-20-1-254nm-1.lcd

Analysis Report

<Sample Information>

Sample Name	: zhy-zd-13-rac-oxh-80-20-254-205-1.0-1		
Sample ID	:		
Data Filename	: zhy-zd-13-rac-oxh-80-20-254-205-1.0-1.lcd		
Method Filename	: 1.0.lcm		
Batch Filename	:		
Vial #	: 1-1	Sample Type	: Unknown
Injection Volume	: 20 uL		
Date Acquired	: 2021/12/3 16:06:10	Acquired by	: System Administrator
Date Processed	: 2021/12/3 16:57:37	Processed by	: System Administrator

<Chromatogram>

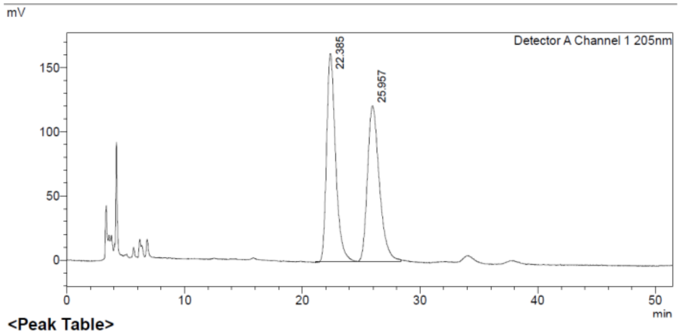

<Peak Table>

Detector A Channel 1 205nm

Peak#	Ret. Time	Area	Height	Conc.
1	22.385	8541836	161885	50.112
2	25.957	8503647	121100	49.888
Total		17045483	282986	

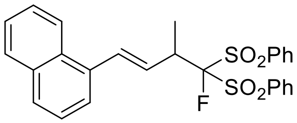

***rac*-3t**

Analysis Report

<Sample Information>

Sample Name	: ZHY-ZD-26-ASY-OXH-80-20-1.0-2544-205-1		
Sample ID	:		
Data Filename	: ZHY-ZD-26-ASY-OXH-80-20-1.0-2544-205-1.lcd		
Method Filename	: 1.0.lcm		
Batch Filename	:		
Vial #	: 1-1	Sample Type	: Unknown
Injection Volume	: 20 uL		
Date Acquired	: 2021/12/21 14:49:01	Acquired by	: System Administrator
Date Processed	: 2021/12/21 15:18:05	Processed by	: System Administrator

<Chromatogram>

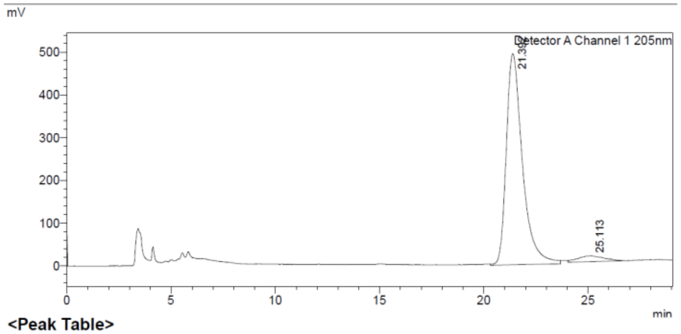

<Peak Table>

Detector A Channel 1 205nm

Peak#	Ret. Time	Area	Height	Conc.
1	21.392	26601734	493882	95.604
2	25.113	1223324	13698	4.396
Total		27825058	507580	

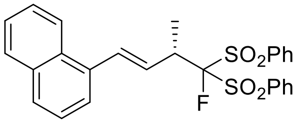

3t

Analysis Report

<Sample Information>

Sample Name : zhy-zc-101-rac-oxh-80-20-1.0-254-230-1
Sample ID :
Data Filename : zhy-zc-101-rac-oxh-80-20-1.0-254-230-1.lcd
Method Filename : 1.0.lcm
Batch Filename :
Vial # : 1-1 Sample Type : Unknown
Injection Volume : 20 uL
Date Acquired : 2021/10/27 15:32:06 Acquired by : System Administrator
Date Processed : 2021/10/27 15:59:08 Processed by : System Administrator

<Chromatogram>

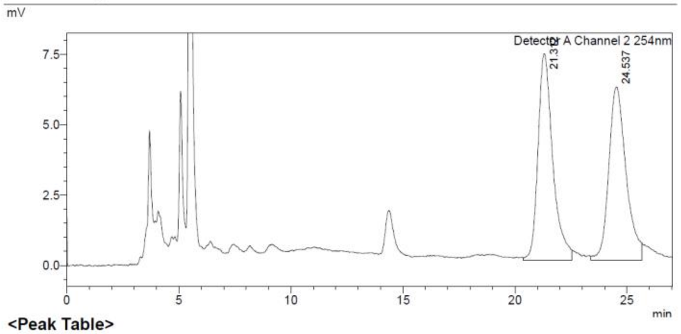

<Peak Table>

Detector A Channel 2 254nm

Peak#	Ret. Time	Area	Height	Conc.
1	21.312	334603	7330	50.453
2	24.537	328588	6150	49.547
Total		663191	13481	

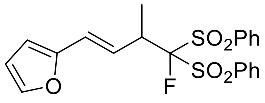

***rac*-3u**

Analysis Report

<Sample Information>

Sample Name : zhy-zc-101-asy-oxh-80-20-1.0-254-230-1
Sample ID :
Data Filename : zhy-zc-101-asy-oxh-80-20-1.0-254-230-1.lcd
Method Filename : 1.0.lcm
Batch Filename :
Vial # : 1-1 Sample Type : Unknown
Injection Volume : 20 uL
Date Acquired : 2021/10/27 15:59:58 Acquired by : System Administrator
Date Processed : 2021/10/27 16:26:35 Processed by : System Administrator

<Chromatogram>

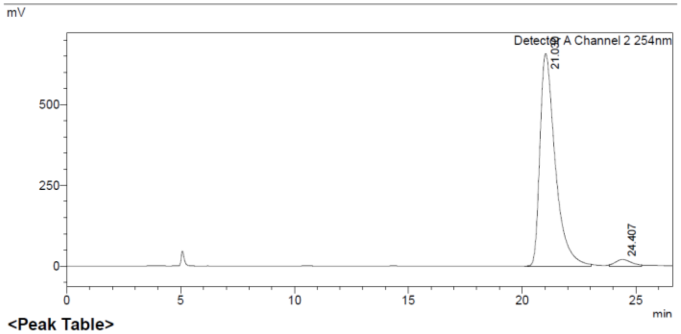

<Peak Table>

Detector A Channel 2 254nm

Peak#	Ret. Time	Area	Height	Conc.
1	21.030	31070718	659559	96.405
2	24.407	1158738	22273	3.595
Total		32229456	681833	

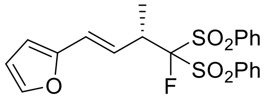

3u

Analysis Report

<Sample Information>

Sample Name	: zhy-zc-103-rac-oxh-80-20-1.0-254-230-1		
Sample ID	:		
Data Filename	: zhy-zc-103-rac-oxh-80-20-1.0-254-230-1.lcd		
Method Filename	: 1.0.lcm		
Batch Filename	:		
Vial #	: 1-1	Sample Type	: Unknown
Injection Volume	: 20 uL		
Date Acquired	: 2021/10/27 16:27:45	Acquired by	: System Administrator
Date Processed	: 2022/1/10 21:23:04	Processed by	: System Administrator

<Chromatogram>

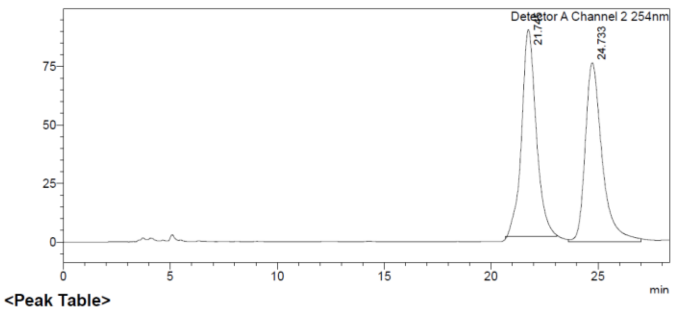

<Peak Table>

Detector A Channel 2 254nm

Peak#	Ret. Time	Area	Height	Conc.
1	21.745	4189858	88243	49.684
2	24.733	4243141	76550	50.316
Total		8433000	164793	

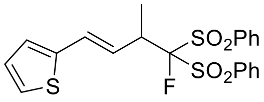

***rac*-3v**

Analysis Report

<Sample Information>

Sample Name	: zhy-zc-114-asy-oxh-80-20-1.0-254-205-1		
Sample ID	:		
Data Filename	: zhy-zc-114-asy-oxh-80-20-1.0-254-205-1.lcd		
Method Filename	: 1.0.lcm		
Batch Filename	:		
Vial #	: 1-1	Sample Type	: Unknown
Injection Volume	: 20 uL		
Date Acquired	: 2021/11/2 20:32:19	Acquired by	: System Administrator
Date Processed	: 2021/11/2 21:05:41	Processed by	: System Administrator

<Chromatogram>

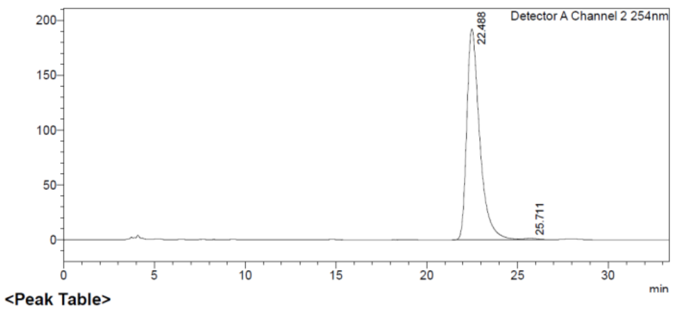

<Peak Table>

Detector A Channel 2 254nm

Peak#	Ret. Time	Area	Height	Conc.
1	22.488	9485031	192140	99.068
2	25.711	89236	1446	0.932
Total		9574267	193586	

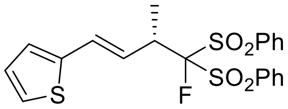

3v

Analysis Report

<Sample Information>

Sample Name : zhy-zc-100-rac-adh-80-20-1.0-254-205-2
Sample ID :
Data Filename : zhy-zc-100-rac-adh-80-20-1.0-254-205-2.lcd
Method Filename : 1.0.lcm
Batch Filename :
Vial # : 1-1 Sample Type : Unknown
Injection Volume : 20 uL
Date Acquired : 2021/10/29 21:33:01 Acquired by : System Administrator
Date Processed : 2021/10/29 22:10:01 Processed by : System Administrator

<Chromatogram>

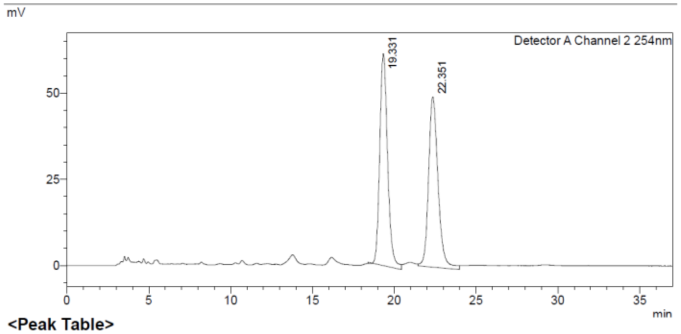

<Peak Table>

Detector A Channel 2 254nm

Peak#	Ret. Time	Area	Height	Conc.
1	19.331	2037939	61537	50.583
2	22.351	1990988	49432	49.417
Total		4028928	110970	

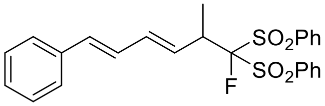

***rac*-3w**

Analysis Report

<Sample Information>

Sample Name : zhy-zc-100-asy-adh-80-20-1.0-254-205-2
Sample ID :
Data Filename : zhy-zc-100-asy-adh-80-20-1.0-254-205-2.lcd
Method Filename : 1.0.lcm
Batch Filename :
Vial # : 1-1 Sample Type : Unknown
Injection Volume : 20 uL
Date Acquired : 2021/10/29 20:56:08 Acquired by : System Administrator
Date Processed : 2021/10/29 21:23:01 Processed by : System Administrator

<Chromatogram>

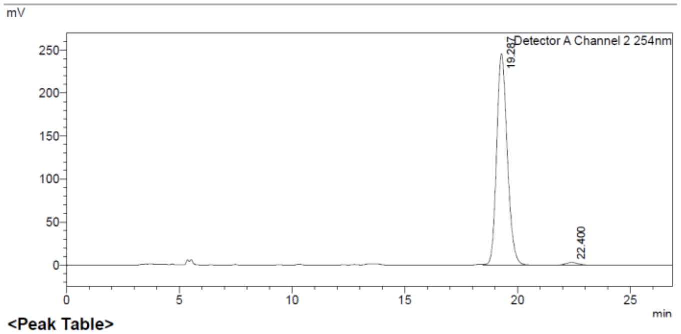

<Peak Table>

Detector A Channel 2 254nm

Peak#	Ret. Time	Area	Height	Conc.
1	19.287	8036969	245599	98.688
2	22.400	106854	2983	1.312
Total		8143823	248582	

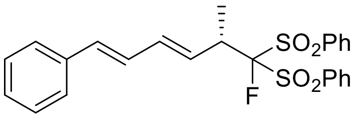

3w

Analysis Report

<Sample Information>

Sample Name	: zhy-zc-91-rac-odh-90-10-1.0-254-230-1		
Sample ID	:		
Data Filename	: zhy-zc-91-rac-odh-90-10-1.0-254-230-1.lcd		
Method Filename	: 1.0.lcm		
Batch Filename	:		
Vial #	: 1-1	Sample Type	: Unknown
Injection Volume	: 20 uL		
Date Acquired	: 2021/10/26 11:43:49	Acquired by	: System Administrator
Date Processed	: 2021/10/26 11:56:53	Processed by	: System Administrator

<Chromatogram>

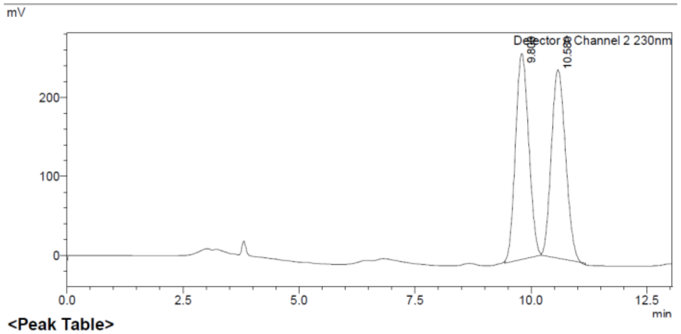

<Peak Table>

Detector A Channel 2 230nm

Peak#	Ret. Time	Area	Height	Conc.
1	9.800	5167359	260392	50.426
2	10.580	5080112	238379	49.574
Total		10247471	498771	

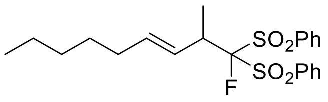

***rac*-3x**

Analysis Report

<Sample Information>

Sample Name	: zhy-zc-126-asy-odh-90-10-1.0-254-205-1		
Sample ID	:		
Data Filename	: zhy-zc-126-asy-odh-90-10-1.0-254-205-1.lcd		
Method Filename	: gay-1.0-254-1.lcm		
Batch Filename	:		
Vial #	: 1-1	Sample Type	: Unknown
Injection Volume	: 20 uL		
Date Acquired	: 2021/11/25 14:33:50	Acquired by	: System Administrator
Date Processed	: 2021/11/25 14:54:07	Processed by	: System Administrator

<Chromatogram>

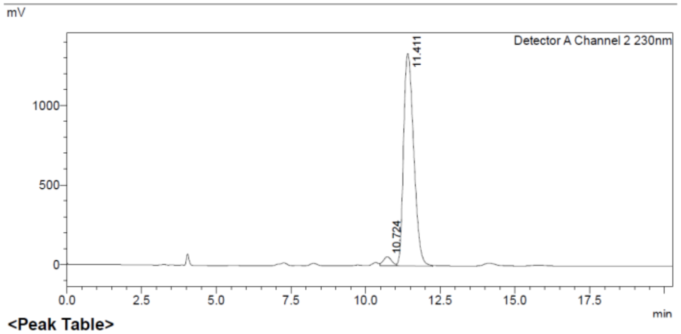

<Peak Table>

Detector A Channel 2 230nm

Peak#	Ret. Time	Area	Height	Conc.
1	10.724	1106956	56041	3.385
2	11.411	31592570	1334818	96.615
Total		32699526	1390859	

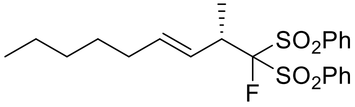

3x

Operator:dell Timebase:U-3000 Sequence:Zhang Xuexin

Page 1-1
2008-8-21 10:14 上午

279 zhy-zc-135-rac-odh-80-20-1.0-205-1

Sample Name:	**zhy-zc-135-rac-odh-80-20-1.0-205-1**	*Injection Volume:*	**20.0**
Vial Number:	**377**	*Channel:*	**UV_VIS_1**
Sample Type:	**standard**	*Wavelength:*	**205**
Control Program:	**Zhang Xuexin**	*Bandwidth:*	**n.a.**
Quantif. Method:	**Zhang Xuexin**	*Dilution Factor:*	**1.0000**
Run Time (min):	**23.95**	*Sample Amount:*	**1.0000**

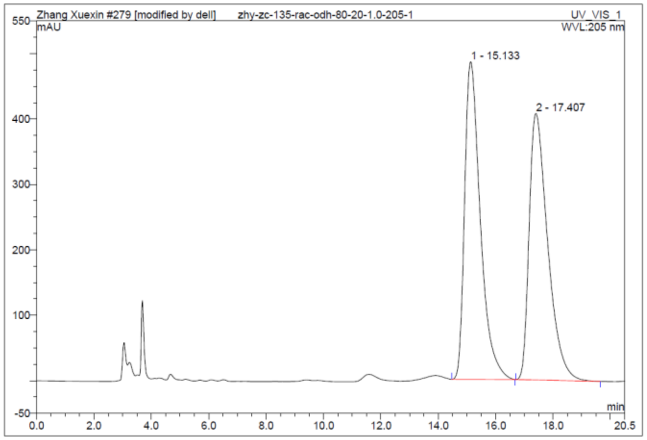

No.	Ret.Time min	Peak Name	Height mAU	Area mAU*min	Rel.Area %	Amount	Type
1	15.13	n.a.	485.429	307.382	50.22	n.a.	BMB
2	17.41	n.a.	407.402	304.685	49.78	n.a.	BMB
**Total:**			892.831	612.068	100.00	0.000	

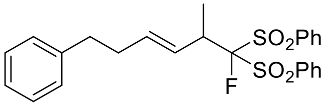

***rac*-3y**

Mu/Integration

Chromeleon (c) Dionex 1996-2006
Version 6.80 SR8a Build 2643 (158225)

Operator:dell Timebase:U-3000 Sequence:Zhang Xuexin

Page 1-1
2008-8-21 10:16 上午

278 zhy-zc-135-asy-odh-80-20-1.0-205-1

Sample Name:	**zhy-zc-135-asy-odh-80-20-1.0-205-1**	*Injection Volume:*	**20.0**
Vial Number:	**376**	*Channel:*	**UV_VIS_1**
Sample Type:	**standard**	*Wavelength:*	**205**
Control Program:	**Zhang Xuexin**	*Bandwidth:*	**n.a.**
Quantif. Method:	**Zhang Xuexin**	*Dilution Factor:*	**1.0000**
Run Time (min):	**33.78**	*Sample Amount:*	**1.0000**

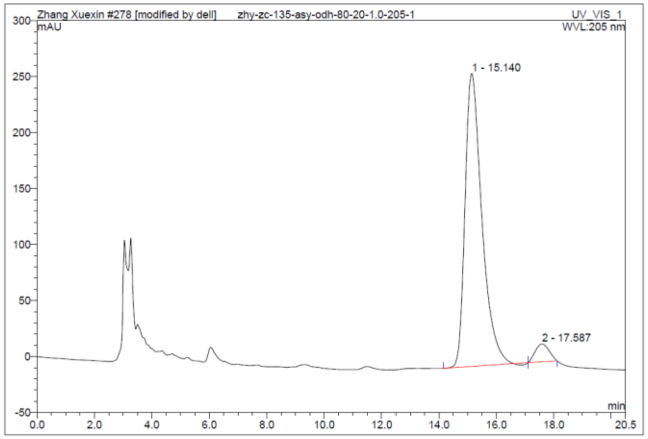

No.	Ret.Time min	Peak Name	Height mAU	Area mAU*min	Rel.Area %	Amount	Type
1	15.14	n.a.	261.597	177.165	95.24	n.a.	BMb*
2	17.59	n.a.	15.933	8.852	4.76	n.a.	bMB*
**Total:**			277.530	186.017	100.00	0.000	

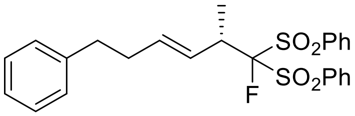

3y

Mu/Integration

Chromeleon (c) Dionex 1996-2006
Version 6.80 SR8a Build 2643 (158225)

Analysis Report

<Sample Information>

Sample Name	: ZHY-ZF-69-3-RAC-OXH-60-40-1.0-254-205-1		
Sample ID	:		
Data Filename	: ZHY-ZF-69-3-RAC-OXH-60-40-1.0-254-205-1.lcd		
Method Filename	: 1.0.lcm		
Batch Filename	:		
Vial #	: 1-1	Sample Type	: Unknown
Injection Volume	: 20 uL		
Date Acquired	: 2022/8/27 15:34:44	Acquired by	: System Administrator
Date Processed	: 2022/8/27 16:04:48	Processed by	: System Administrator

<Chromatogram>

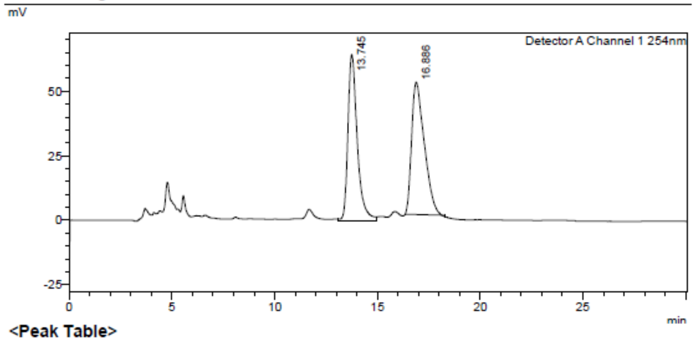

<Peak Table>

Detector A Channel 1 254nm

Peak#	Ret. Time	Area	Height	Conc.
1	13.745	2039662	64454	48.199
2	16.886	2192077	51369	51.801
Total		4231739	115823	

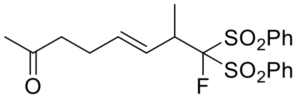

***rac*-3z**

Analysis Report

<Sample Information>

Sample Name	: ZHY-ZF-69-3-ASY-OXH-60-40-1.0-254-205-1		
Sample ID	:		
Data Filename	: ZHY-ZF-69-3-ASY-OXH-60-40-1.0-254-205-1.lcd		
Method Filename	: 1.0.lcm		
Batch Filename	:		
Vial #	: 1-1	Sample Type	: Unknown
Injection Volume	: 20 uL		
Date Acquired	: 2022/8/27 16:06:26	Acquired by	: System Administrator
Date Processed	: 2022/8/27 16:27:55	Processed by	: System Administrator

<Chromatogram>

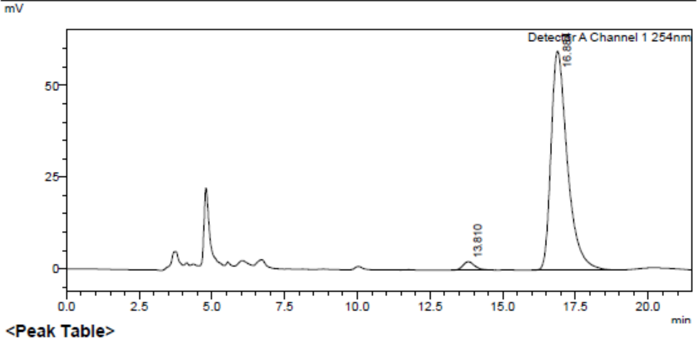

<Peak Table>

Detector A Channel 1 254nm

Peak#	Ret. Time	Area	Height	Conc.
1	13.810	67092	2327	2.775
2	16.884	2350301	59683	97.225
Total		2417393	62010	

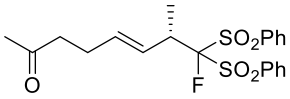

3z

Analysis Report

<Sample Information>

Sample Name : zhy-zc-88-rac-adh-85-15-1.0-254-230-1
Sample ID :
Data Filename : zhy-zc-88-rac-adh-85-15-1.0-254-230-1.lcd
Method Filename : 1.0.lcm
Batch Filename :
Vial # : 1-1 Sample Type : Unknown
Injection Volume : 20 uL
Date Acquired : 2021/10/20 15:30:49 Acquired by : System Administrator
Date Processed : 2021/11/4 21:07:01 Processed by : System Administrator

<Chromatogram>

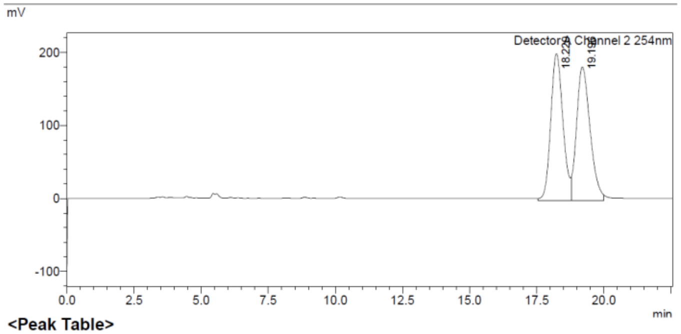

<Peak Table>

Detector A Channel 2 254nm

Peak#	Ret. Time	Area	Height	Conc.
1	18.229	6396490	201534	49.787
2	19.196	6451105	183310	50.213
Total		12847596	384844	

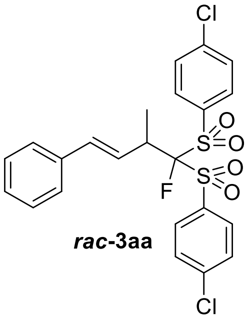

***rac*-3aa**

Analysis Report

<Sample Information>

Sample Name : zhy-zc-82-asy-adh-85-15-1.0-254-230-2
Sample ID :
Data Filename : zhy-zc-82-asy-adh-85-15-1.0-254-230-2.lcd
Method Filename : 1.0.lcm
Batch Filename :
Vial # : 1-1 Sample Type : Unknown
Injection Volume : 20 uL
Date Acquired : 2021/10/20 21:08:07 Acquired by : System Administrator
Date Processed : 2021/10/20 21:30:32 Processed by : System Administrator

<Chromatogram>

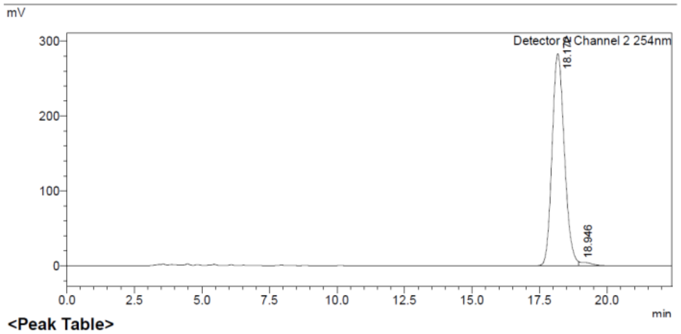

<Peak Table>

Detector A Channel 2 254nm

Peak#	Ret. Time	Area	Height	Conc.
1	18.172	8945681	283037	98.357
2	18.946	149398	5579	1.643
Total		9095079	288615	

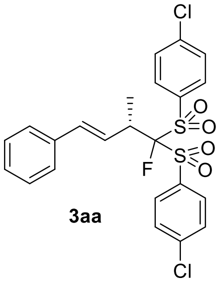

3aa

Analysis Report

<Sample Information>

Sample Name	: zhy-zc-81-rac-adh-60-40-1.0-254-230-1		
Sample ID	:		
Data Filename	: zhy-zc-81-rac-adh-60-40-1.0-254-230-1.lcd		
Method Filename	: xsl-230-254-1.0.lcm		
Batch Filename	:		
Vial #	: 1-1	Sample Type	: Unknown
Injection Volume	: 20 uL		
Date Acquired	: 2021/10/19 16:19:48	Acquired by	: System Administrator
Date Processed	: 2022/1/12 21:37:41	Processed by	: System Administrator

<Chromatogram>

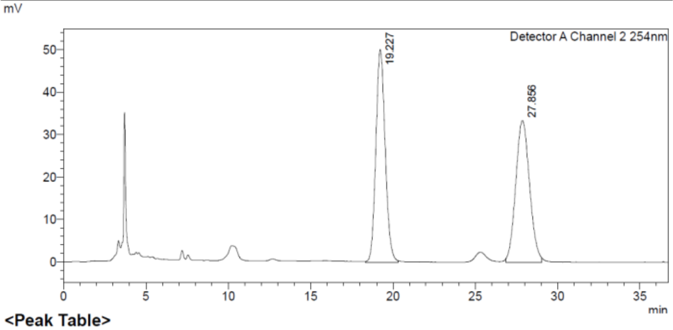

<Peak Table>

Detector A Channel 2 254nm

Peak#	Ret. Time	Area	Height	Conc.
1	19.227	1967862	50139	50.245
2	27.856	1948699	33400	49.755
Total		3916562	83539	

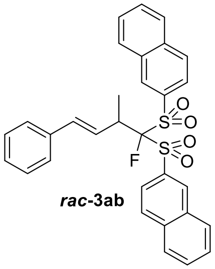

***rac*-3ab**

Analysis Report

<Sample Information>

Sample Name	: zhy-zc-146-asy-adh-60-40-1.--254-230-1		
Sample ID	:		
Data Filename	: zhy-zc-146-asy-adh-60-40-1.--254-230-1.lcd		
Method Filename	: 1.0.lcm		
Batch Filename	:		
Vial #	: 1-1	Sample Type	: Unknown
Injection Volume	: 20 uL		
Date Acquired	: 2021/11/26 16:43:38	Acquired by	: System Administrator
Date Processed	: 2022/1/10 21:00:17	Processed by	: System Administrator

<Chromatogram>

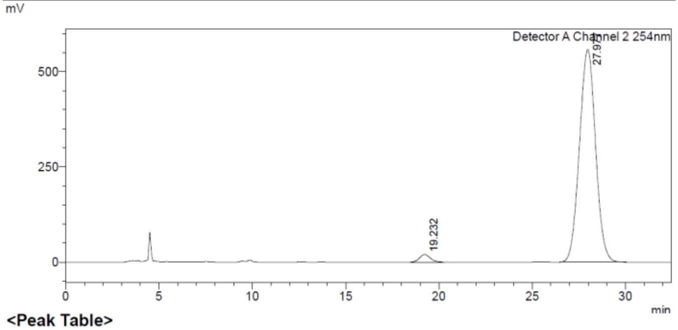

<Peak Table>

Detector A Channel 2 254nm

Peak#	Ret. Time	Area	Height	Conc.
1	19.232	859082	20406	2.504
2	27.977	33450234	557414	97.496
Total		34309315	577820	

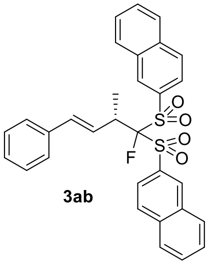

3ab

233 zhy-zd-38-rac-oxh-95-5-1.0-254-1

Sample Name:	**zhy-zd-38-rac-oxh-95-5-1.0-254-1**	*Injection Volume:*	**20.0**
Vial Number:	**331**	*Channel:*	**UV_VIS_1**
Sample Type:	**standard**	*Wavelength:*	**205**
Control Program:	**Zhang Xuexin**	*Bandwidth:*	**n.a.**
Quantif. Method:	**Zhang Xuexin**	*Dilution Factor:*	**1.0000**
Run Time (min):	**33.96**	*Sample Amount:*	**1.0000**

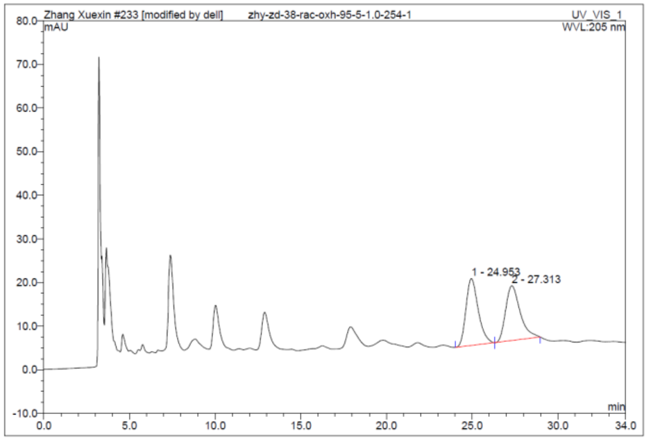

No.	Ret.Time min	Peak Name	Height mAU	Area mAU*min	Rel.Area %	Amount	Type
1	24.95	n.a.	15.330	12.683	50.83	n.a.	BM *
2	27.31	n.a.	12.564	12.268	49.17	n.a.	MB*
Total:			27.894	24.951	100.00	0.000	

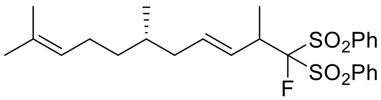

***rac*-3ac**

232 zhy-zd-38-asy-oxh-95-5-1.0-254-1

Sample Name:	**zhy-zd-38-asy-oxh-95-5-1.0-254-1**	*Injection Volume:*	**20.0**
Vial Number:	**330**	*Channel:*	**UV_VIS_1**
Sample Type:	**standard**	*Wavelength:*	**205**
Control Program:	**Zhang Xuexin**	*Bandwidth:*	**n.a.**
Quantif. Method:	**Zhang Xuexin**	*Dilution Factor:*	**1.0000**
Run Time (min):	**57.99**	*Sample Amount:*	**1.0000**

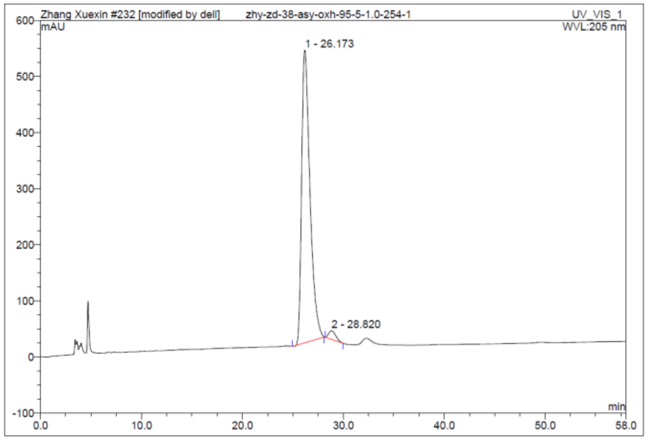

No.	Ret.Time min	Peak Name	Height mAU	Area mAU*min	Rel.Area %	Amount	Type
1	26.17	n.a.	521.506	498.400	97.51	n.a.	BMB*
2	28.82	n.a.	15.462	12.712	2.49	n.a.	BMB*
Total:			536.968	511.112	100.00	0.000	

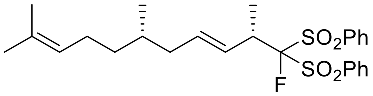

3ac

Analysis Report

<Sample Information>

Sample Name	: zhy-zD-23-RAC-odh-100-0-254-205-1.0-1		
Sample ID	:		
Data Filename	: zhy-zD-23-RAC-odh-100-0-254-205-1.0-1.lcd		
Method Filename	: 1.0.lcm		
Batch Filename	:		
Vial #	: 1-1	Sample Type	: Unknown
Injection Volume	: 20 uL		
Date Acquired	: 2021/12/4 10:36:57	Acquired by	: System Administrator
Date Processed	: 2022/1/12 16:43:56	Processed by	: System Administrator

<Chromatogram>

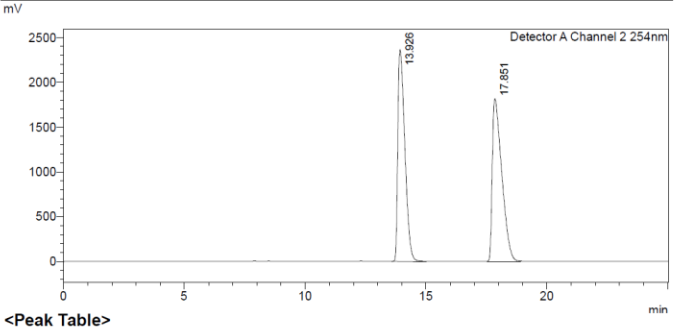

<Peak Table>

Detector A Channel 2 254nm

Peak#	Ret. Time	Area	Height	Conc.
1	13.926	48005818	2365030	49.251
2	17.851	49466173	1817779	50.749
Total		97471991	4182810	

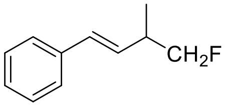

***rac*-4a**

Analysis Report

<Sample Information>

Sample Name	: zhy-zD-23-ASY-odh-100-0-254-205-1.0-1		
Sample ID	:		
Data Filename	: zhy-zD-23-ASY-odh-100-0-254-205-1.0-1.lcd		
Method Filename	: 1.0.lcm		
Batch Filename	:		
Vial #	: 1-1	Sample Type	: Unknown
Injection Volume	: 20 uL		
Date Acquired	: 2021/12/4 11:03:20	Acquired by	: System Administrator
Date Processed	: 2021/12/4 11:32:43	Processed by	: System Administrator

<Chromatogram>

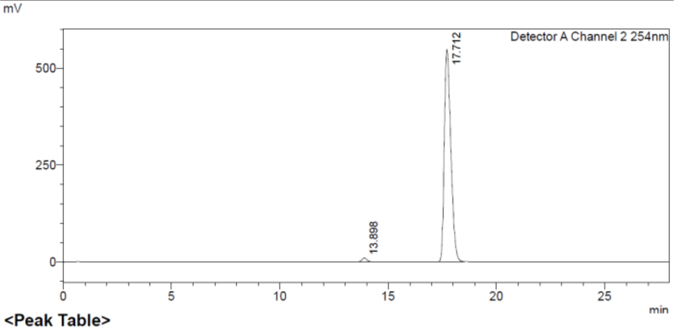

<Peak Table>

Detector A Channel 2 254nm

Peak#	Ret. Time	Area	Height	Conc.
1	13.898	146028	9834	1.261
2	17.712	11434501	547523	98.739
Total		11580529	557356	

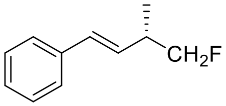

4a

2022/1/7 22:11:24 Page 1 / 1

Analysis Report

<Sample Information>

Sample Name	: ZHY-ZD-41-rac-ODH-99.9-0.1-1.0-205-254-2		
Sample ID	:		
Data Filename	: ZHY-ZD-41-rac-ODH-99.9-0.1-1.0-205-254-2.lcd		
Method Filename	: 1.0.lcm		
Batch Filename	:		
Vial #	: 1-1	Sample Type	: Unknown
Injection Volume	: 10 uL		
Date Acquired	: 2022/1/7 20:20:40	Acquired by	: System Administrator
Date Processed	: 2022/1/7 20:51:16	Processed by	: System Administrator

<Chromatogram>

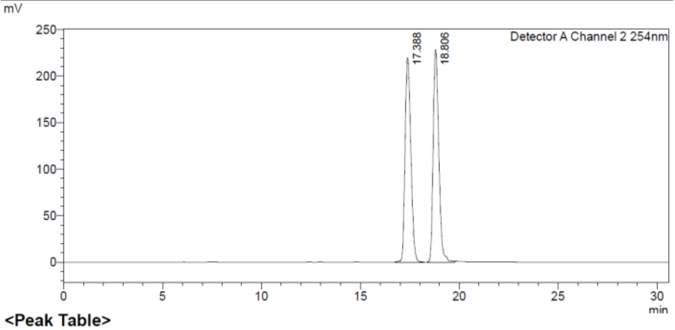

<Peak Table>

Detector A Channel 2 254nm

Peak#	Ret. Time	Area	Height	Conc.
1	17.388	4505776	220304	49.472
2	18.806	4601872	229073	50.528
Total		9107649	449377	

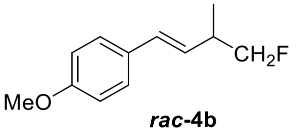

***rac*-4b**

D:\Data\ZHY\ZD\ZHY-ZD-41-rac-ODH-99.9-0.1-1.0-205-254-2.lcd

2022/1/7 22:10:33 Page 1 / 1

Analysis Report

<Sample Information>

Sample Name	: ZHY-ZD-41-asy-ODH-99.9-0.1-1.0-205-254-4		
Sample ID	:		
Data Filename	: ZHY-ZD-41-asy-ODH-99.9-0.1-1.0-205-254-4.lcd		
Method Filename	: 1.0.lcm		
Batch Filename	:		
Vial #	: 1-1	Sample Type	: Unknown
Injection Volume	: 10 uL		
Date Acquired	: 2022/1/7 21:39:57	Acquired by	: System Administrator
Date Processed	: 2022/1/7 22:06:58	Processed by	: System Administrator

<Chromatogram>

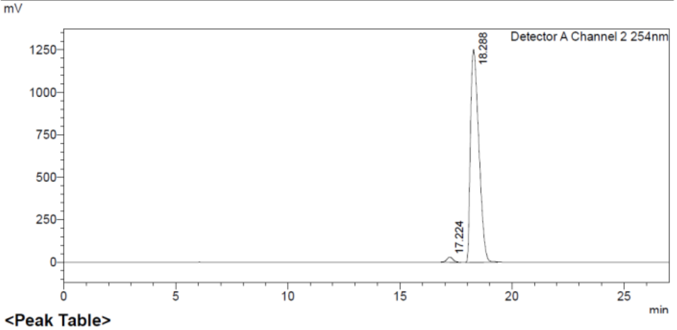

<Peak Table>

Detector A Channel 2 254nm

Peak#	Ret. Time	Area	Height	Conc.
1	17.224	622858	30688	1.878
2	18.288	32535022	1253488	98.122
Total		33157880	1284175	

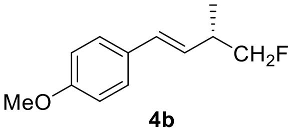

4b

D:\Data\ZHY\ZD\ZHY-ZD-41-asy-ODH-99.9-0.1-1.0-205-254-4.lcd

Analysis Report

<Sample Information>

Sample Name	: zhy-zd-45-rac-odh-100-0-1.0-205-254-1		
Sample ID	:		
Data Filename	: zhy-zd-45-rac-odh-100-0-1.0-205-254-1.lcd		
Method Filename	: 1.0.lcm		
Batch Filename	:		
Vial #	: 1-1	Sample Type	: Unknown
Injection Volume	: 10 uL		
Date Acquired	: 2022/1/11 15:20:57	Acquired by	: System Administrator
Date Processed	: 2022/1/11 15:33:05	Processed by	: System Administrator

<Chromatogram>

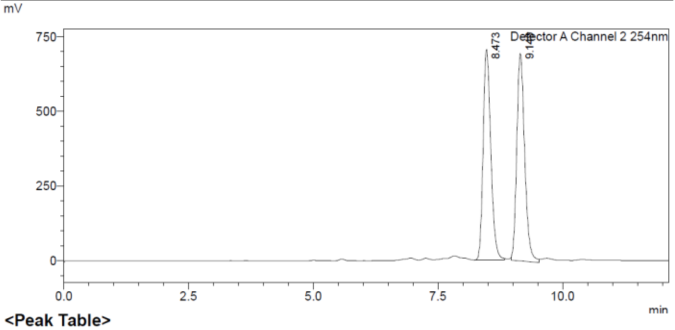

<Peak Table>

Detector A Channel 2 254nm

Peak#	Ret. Time	Area	Height	Conc.
1	8.473	7539346	705065	49.570
2	9.149	7670042	694637	50.430
Total		15209388	1399702	

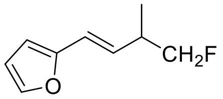

***rac*-4c**

Analysis Report

<Sample Information>

Sample Name	: zhy-zd-45-asy-odh-100-0-1.0-205-254-1		
Sample ID	:		
Data Filename	: zhy-zd-45-asy-odh-100-0-1.0-205-254-1.lcd		
Method Filename	: 1.0.lcm		
Batch Filename	:		
Vial #	: 1-1	Sample Type	: Unknown
Injection Volume	: 10 uL		
Date Acquired	: 2022/1/11 15:34:51	Acquired by	: System Administrator
Date Processed	: 2022/1/11 15:47:29	Processed by	: System Administrator

<Chromatogram>

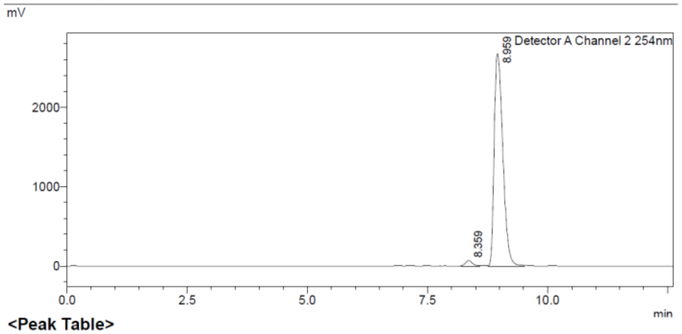

<Peak Table>

Detector A Channel 2 254nm

Peak#	Ret. Time	Area	Height	Conc.
1	8.359	753581	70667	2.199
2	8.959	33518956	2677099	97.801
Total		34272538	2747766	

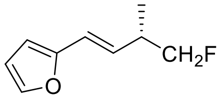

4c

Analysis Report

<Sample Information>

Sample Name : ZHY-ZD-48-rac-2odh-100-0-1.0-254-205-2
Sample ID :
Data Filename : ZHY-ZD-48-rac-2odh-100-0-1.0-254-205-2.lcd
Method Filename : 1.0.lcm
Batch Filename :
Vial # : 1-1 Sample Type : Unknown
Injection Volume : 10 uL
Date Acquired : 2022/1/10 21:40:54 Acquired by : System Administrator
Date Processed : 2022/1/10 22:03:13 Processed by : System Administrator

<Chromatogram>

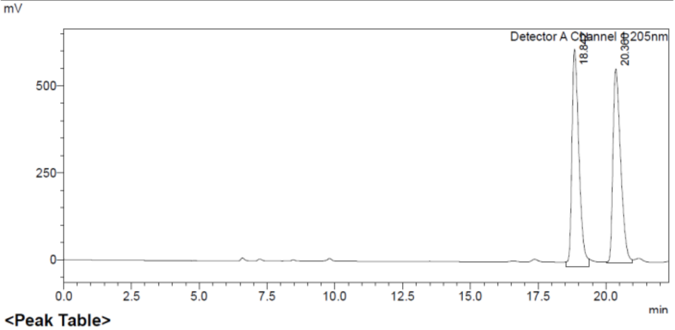

<Peak Table>

Detector A Channel 1 205nm

Peak#	Ret. Time	Area	Height	Conc.
1	18.842	11284893	623936	50.469
2	20.360	11075154	558414	49.531
Total		22360047	1182350	

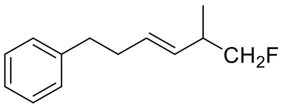

***rac*-4d**

Analysis Report

<Sample Information>

Sample Name : ZHY-ZD-48-asy-2odh-100-0-1.0-254-205-2
Sample ID :
Data Filename : ZHY-ZD-48-asy-2odh-100-0-1.0-254-205-2.lcd
Method Filename : 1.0.lcm
Batch Filename :
Vial # : 1-1 Sample Type : Unknown
Injection Volume : 10 uL
Date Acquired : 2022/1/10 21:12:55 Acquired by : System Administrator
Date Processed : 2022/1/10 21:38:51 Processed by : System Administrator

<Chromatogram>

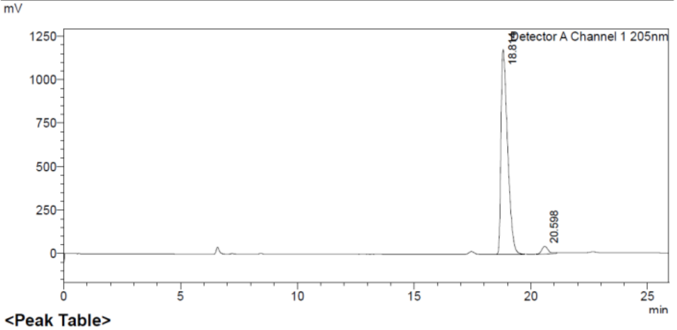

<Peak Table>

Detector A Channel 1 205nm

Peak#	Ret. Time	Area	Height	Conc.
1	18.814	23327612	1177878	96.306
2	20.598	894820	44587	3.694
Total		24222432	1222465	

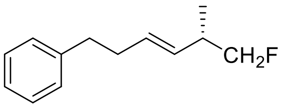

4d

Analysis Report

<Sample Information>

Sample Name	: ZHY-ZD-121-RAC-ODH-100-0-1.0-254-205-1		
Sample ID	:		
Data Filename	: ZHY-ZD-116-RAC-ODH-100-0-1.0-254-205-1.lcd		
Method Filename	: 1.0.lcm		
Batch Filename	:		
Vial #	: 1-1	Sample Type	: Unknown
Injection Volume	: 20 uL		
Date Acquired	: 2022/3/12 17:21:44	Acquired by	: System Administrator
Date Processed	: 2022/3/12 18:15:43	Processed by	: System Administrator

<Chromatogram>

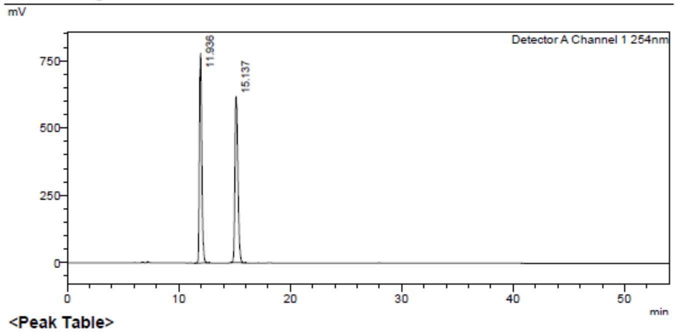

<Peak Table>

Detector A Channel 1 254nm

Peak#	Ret. Time	Area	Height	Conc.
1	11.936	11407645	779365	50.147
2	15.137	11340658	617626	49.853
Total		22748303	1396991	

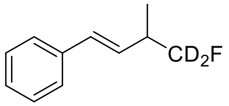

***rac*-D-4a**

Analysis Report

<Sample Information>

Sample Name	: ZHY-ZD-120-ASY-ODH-100-0-1.0-254-205-1		
Sample ID	:		
Data Filename	: ZHY-ZD-116-ASY-ODH-100-0-1.0-254-205-1.lcd		
Method Filename	: 1.0.lcm		
Batch Filename	:		
Vial #	: 1-1	Sample Type	: Unknown
Injection Volume	: 20 uL		
Date Acquired	: 2022/3/12 18:25:35	Acquired by	: System Administrator
Date Processed	: 2022/3/12 18:44:52	Processed by	: System Administrator

<Chromatogram>

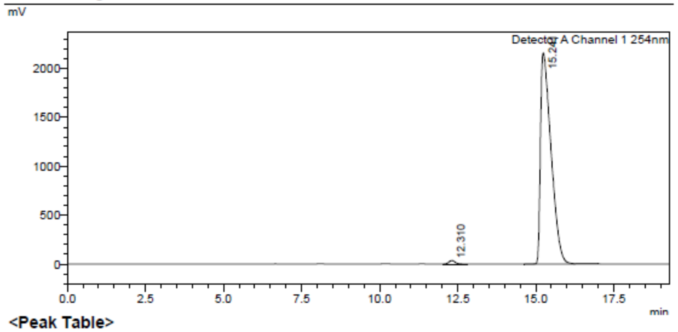

<Peak Table>

Detector A Channel 1 254nm

Peak#	Ret. Time	Area	Height	Conc.
1	12.310	477880	34766	0.933
2	15.241	50729706	2158138	99.067
Total		51207586	2192905	

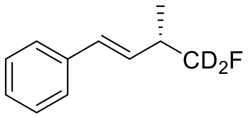

D-4a

2022/5/28 17:44:04 Page 1 / 1

Analysis Report

<Sample Information>

Sample Name	: ZHY-ZJ-101-RAC-IC-100-0-1.0-230-254-1		
Sample ID	:		
Data Filename	: ZHY-ZJ-101-RAC-IC-100-0-1.0-230-254-1.lcd		
Method Filename	: ZHY-101.lcm		
Batch Filename	:		
Vial #	: 1-1	Sample Type	: Unknown
Injection Volume	: 20 uL		
Date Acquired	: 2022/5/28 16:55:08	Acquired by	: System Administrator
Date Processed	: 2022/5/28 17:11:01	Processed by	: System Administrator

<Chromatogram>

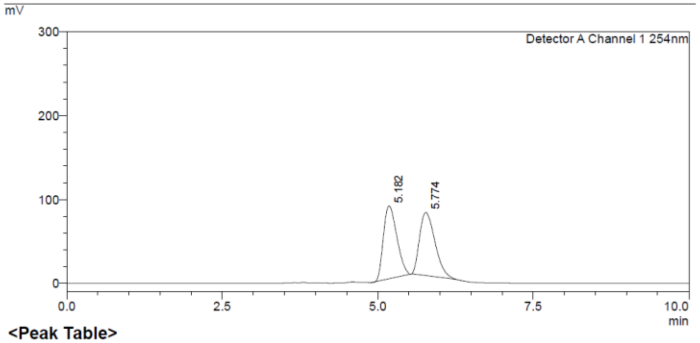

<Peak Table>

Detector A Channel 1 254nm

Peak#	Ret. Time	Area	Height	Conc.
1	5.182	1305700	86844	50.291
2	5.774	1290605	75106	49.709
Total		2596305	161951	

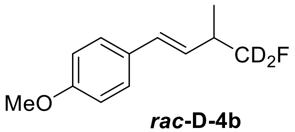

***rac*-D-4b**

C:\LabSolutions\Data\ZHY-ZJ-101-RAC-IC-100-0-1.0-230-254-1.lcd

2022/5/28 17:45:40 Page 1 / 1

Analysis Report

<Sample Information>

Sample Name	: ZHY-ZJ-101-ASY-IC-100-0-1.0-230-254-1		
Sample ID	:		
Data Filename	: ZHY-ZJ-101-ASY-IC-100-0-1.0-230-254-1.lcd		
Method Filename	: ZHY-101.lcm		
Batch Filename	:		
Vial #	: 1-1	Sample Type	: Unknown
Injection Volume	: 20 uL		
Date Acquired	: 2022/5/28 17:14:36	Acquired by	: System Administrator
Date Processed	: 2022/5/28 17:27:07	Processed by	: System Administrator

<Chromatogram>

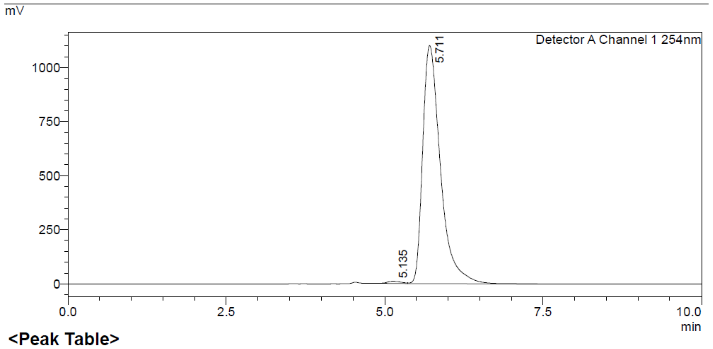

<Peak Table>

Detector A Channel 1 254nm

Peak#	Ret. Time	Area	Height	Conc.
1	5.135	152137	10010	0.709
2	5.711	21317143	1100025	99.291
Total		21469279	1110035	

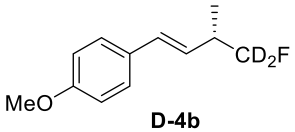

D-4b

C:\LabSolutions\Data\ZHY-ZJ-101-ASY-IC-100-0-1.0-230-254-1.lcd

Analysis Report

<Sample Information>

Sample Name	: ZHY-ZD-120-FUNAN -RAC-ODH-100-0-1.0-254-205-1		
Sample ID	:		
Data Filename	: ZHY-ZD-120-FUNAN -RAC-ODH-100-0-1.0-254-205-1.lcd		
Method Filename	: 1.0.lcm		
Batch Filename	:		
Vial #	: 1-1	Sample Type	: Unknown
Injection Volume	: 20 uL		
Date Acquired	: 2022/3/12 22:06:43	Acquired by	: System Administrator
Date Processed	: 2022/3/12 22:23:48	Processed by	: System Administrator

<Chromatogram>

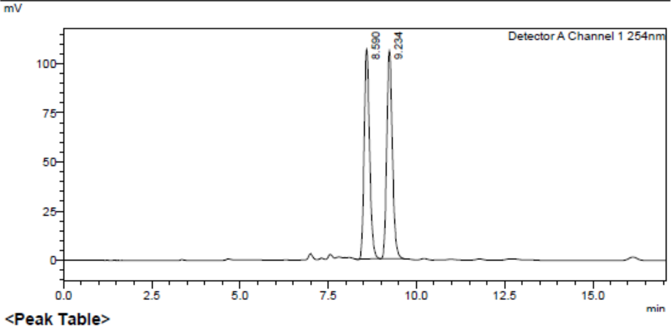

<Peak Table>

Detector A Channel 1 254nm

Peak#	Ret. Time	Area	Height	Conc.
1	8.590	1145583	106696	49.396
2	9.234	1173579	105831	50.604
Total		2319162	212527	

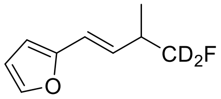

***rac*-D-4c**

Analysis Report

<Sample Information>

Sample Name	: ZHY-ZD-120-FUNAN -ASY-ODH-100-0-1.0-254-205-1		
Sample ID	:		
Data Filename	: ZHY-ZD-120-FUNAN -ASY-ODH-100-0-1.0-254-205-1.lcd		
Method Filename	: 1.0.lcm		
Batch Filename	:		
Vial #	: 1-1	Sample Type	: Unknown
Injection Volume	: 20 uL		
Date Acquired	: 2022/3/12 22:24:18	Acquired by	: System Administrator
Date Processed	: 2022/3/12 22:34:48	Processed by	: System Administrator

<Chromatogram>

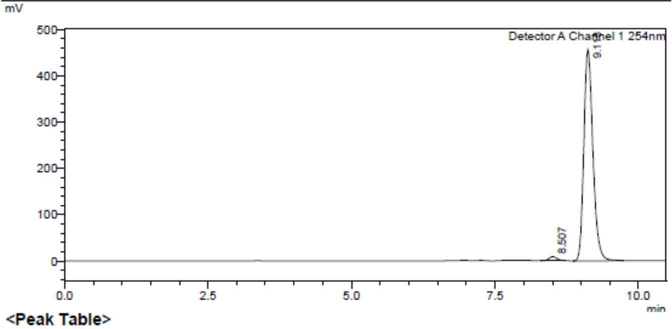

<Peak Table>

Detector A Channel 1 254nm

Peak#	Ret. Time	Area	Height	Conc.
1	8.507	86746	8757	1.699
2	9.118	5018713	456766	98.301
Total		5105459	465524	

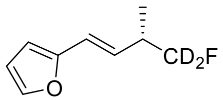

D-4c

Analysis Report

<Sample Information>

Sample Name	: ZHY-ZD-123-RAC-ODH-100-0-1-220-225-1		
Sample ID	:		
Data Filename	: ZHY-ZD-123-RAC-ODH-100-0-1-220-225-1.lcd		
Method Filename	: ZHY-ZD-101.lcm		
Batch Filename	:		
Vial #	: 1-1	Sample Type	: Unknown
Injection Volume	: 20 uL		
Date Acquired	: 2022/5/24 10:45:20	Acquired by	: System Administrator
Date Processed	: 2022/5/24 11:08:00	Processed by	: System Administrator

<Chromatogram>

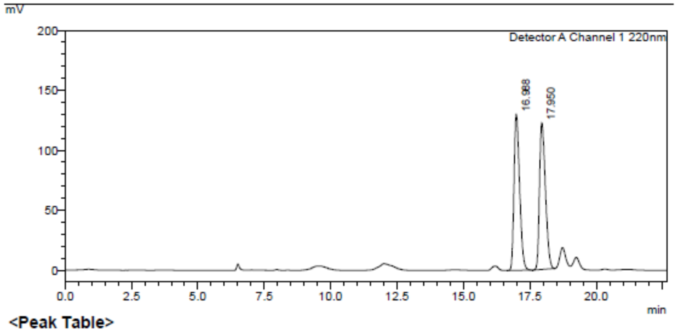

<Peak Table>

Detector A Channel 1 220nm

Peak#	Ret. Time	Area	Height	Conc.
1	16.988	1942318	130288	50.565
2	17.950	1898918	122382	49.435
Total		3841235	252670	

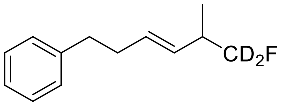

***rac*-D-4d**

Analysis Report

<Sample Information>

Sample Name	: ZHY-ZD-123-ASY-ODH-100-0-1-220-225-1		
Sample ID	:		
Data Filename	: ZHY-ZD-123-ASY-ODH-100-0-1-220-225-1.lcd		
Method Filename	: ZHY-ZD-101.lcm		
Batch Filename	:		
Vial #	: 1-1	Sample Type	: Unknown
Injection Volume	: 20 uL		
Date Acquired	: 2022/5/24 11:10:12	Acquired by	: System Administrator
Date Processed	: 2022/5/24 11:32:54	Processed by	: System Administrator

<Chromatogram>

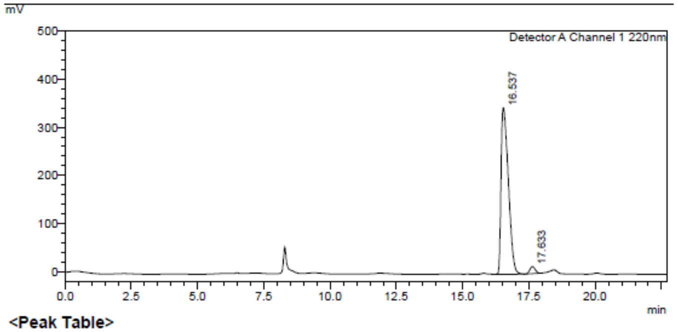

<Peak Table>

Detector A Channel 1 220nm

Peak#	Ret. Time	Area	Height	Conc.
1	16.537	6563571	344105	96.928
2	17.633	208021	14703	3.072
Total		6771592	358808	

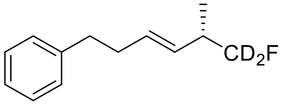

D-4d

Analysis Report

<Sample Information>

Sample Name : lil-lb-122-dppf-rac-adh-98-2-1.0-230nm-01
Sample ID :
Data Filename : lil-lb-122-dppf-rac-adh-98-2-1.0-230nm-01.lcd
Method Filename : xsl-230-254-1.0.lcm
Batch Filename :
Vial # : 1-1 Sample Type : Unknown
Injection Volume : 20 uL
Date Acquired : 2021/7/20 15:06:50 Acquired by : System Administrator
Date Processed : 2021/7/20 15:21:09 Processed by : System Administrator

<Chromatogram>

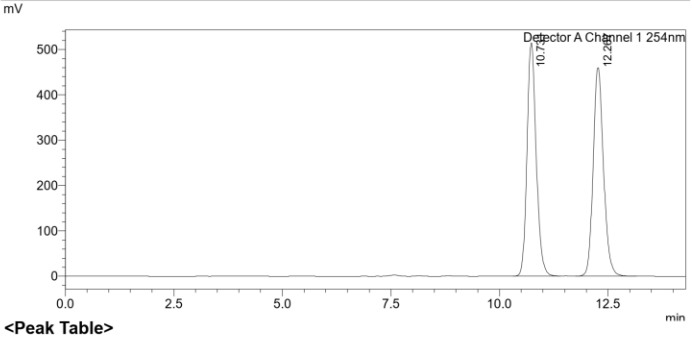

<Peak Table>

Detector A Channel 1 254nm

Peak#	Ret. Time	Area	Height	Conc.
1	10.731	7582676	514058	49.900
2	12.267	7612970	460166	50.100
Total		15195646	974224	

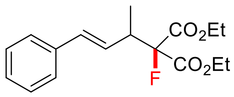

rac-**6a**

Analysis Report

<Sample Information>

Sample Name : lil-ld-13-5mol-50-asy-adh-98-2-254-1.0
Sample ID :
Data Filename : lil-ld-13-5mol-50-asy-adh-98-2-254-1.0.lcd
Method Filename : xsl-230-254-1.0.lcm
Batch Filename :
Vial # : 1-1 Sample Type : Unknown
Injection Volume : 20 uL
Date Acquired : 2021/9/30 12:21:47 Acquired by : System Administrator
Date Processed : 2021/9/30 12:40:42 Processed by : System Administrator

<Chromatogram>

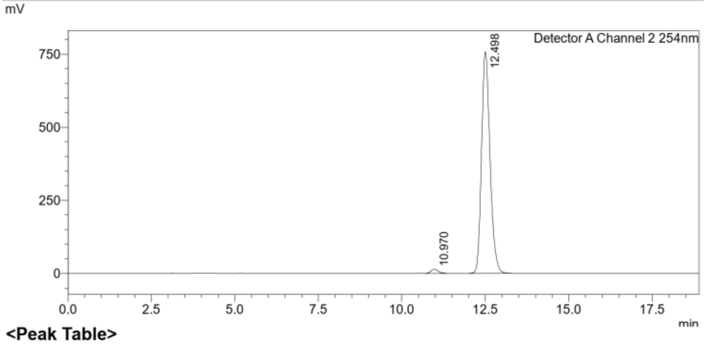

<Peak Table>

Detector A Channel 2 254nm

Peak#	Ret. Time	Area	Height	Conc.
1	10.970	222606	14401	1.636
2	12.498	13383782	757756	98.364
Total		13606388	772158	

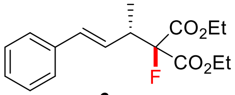

6a

Analysis Report

<Sample Information>

Sample Name : lil-ld-25-2-rac-adh-98-2-254-230-2
Sample ID :
Data Filename : lil-ld-25-2-rac-adh-98-2-254-230-2.lcd
Method Filename : xsl-230-254-1.0.lcm
Batch Filename :
Vial # : 1-1 Sample Type : Unknown
Injection Volume : 20 uL
Date Acquired : 2021/12/23 15:24:18 Acquired by : System Administrator
Date Processed : 2021/12/23 15:45:00 Processed by : System Administrator

<Chromatogram>

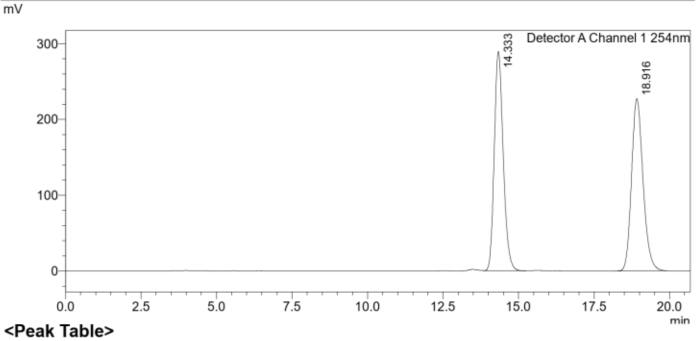

<Peak Table>

Detector A Channel 1 254nm

Peak#	Ret. Time	Area	Height	Conc.
1	14.333	5953905	289364	49.588
2	18.916	6052960	227312	50.412
Total		12006865	516677	

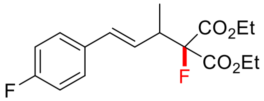

rac-**6b**

Analysis Report

<Sample Information>

Sample Name : lil-ld-25-2-asy-adh-98-2-254-230-2
Sample ID :
Data Filename : lil-ld-25-2-asy-adh-98-2-254-230-2.lcd
Method Filename : xsl-230-254-1.0.lcm
Batch Filename :
Vial # : 1-1 Sample Type : Unknown
Injection Volume : 20 uL
Date Acquired : 2021/12/23 15:01:59 Acquired by : System Administrator
Date Processed : 2021/12/23 15:23:08 Processed by : System Administrator

<Chromatogram>

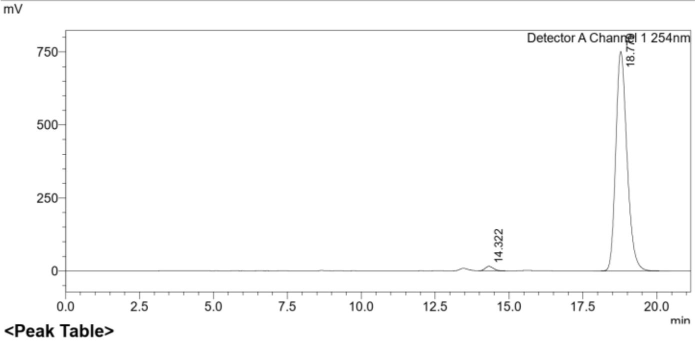

<Peak Table>

Detector A Channel 1 254nm

Peak#	Ret. Time	Area	Height	Conc.
1	14.322	306697	15510	1.517
2	18.779	19904988	751646	98.483
Total		20211685	767155	

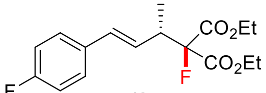

6b

Analysis Report

<Sample Information>

Sample Name	: lil-ld-p-cl--rac-adh-98-2-1.0-254-230-1		
Sample ID	:		
Data Filename	: lil-ld-p-cl--rac-adh-98-2-1.0-254-230-1.lcd		
Method Filename	: xsl-230-254-1.0.lcm		
Batch Filename	:		
Vial #	: 1-1	Sample Type	: Unknown
Injection Volume	: 20 uL		
Date Acquired	: 2021/10/17 16:13:57	Acquired by	: System Administrator
Date Processed	: 2021/10/17 16:31:52	Processed by	: System Administrator

<Chromatogram>

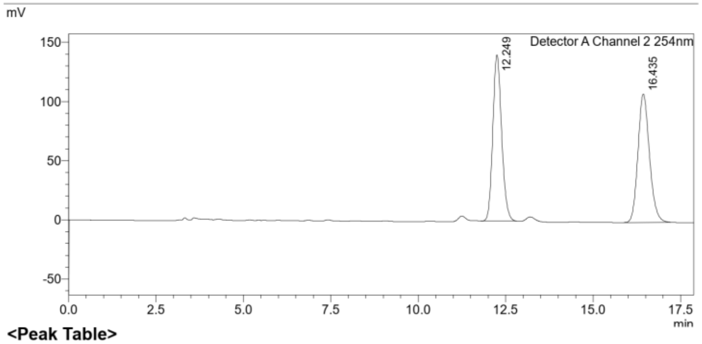

<Peak Table>

Detector A Channel 2 254nm

Peak#	Ret. Time	Area	Height	Conc.
1	12.249	2471188	140356	49.818
2	16.435	2489239	108405	50.182
Total		4960427	248761	

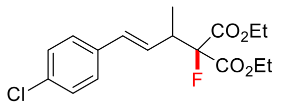

rac-**6c**

Analysis Report

<Sample Information>

Sample Name	: lil-ld-Cl--asy-adh-98-2-1.0-254-230-1		
Sample ID	:		
Data Filename	: lil-ld-Cl--asy-adh-98-2-1.0-254-230-1.lcd		
Method Filename	: xsl-230-254-1.0.lcm		
Batch Filename	:		
Vial #	: 1-1	Sample Type	: Unknown
Injection Volume	: 20 uL		
Date Acquired	: 2021/10/17 17:33:21	Acquired by	: System Administrator
Date Processed	: 2021/10/17 17:51:20	Processed by	: System Administrator

<Chromatogram>

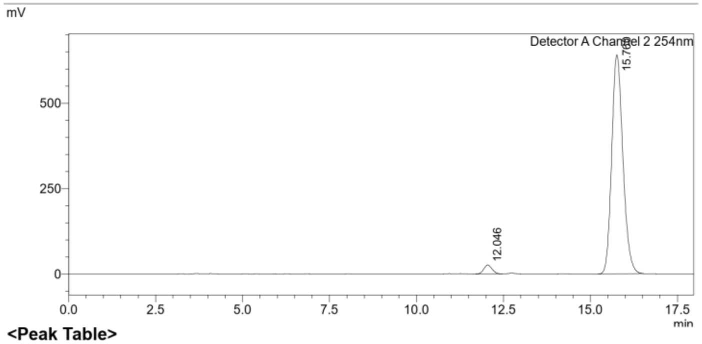

<Peak Table>

Detector A Channel 2 254nm

Peak#	Ret. Time	Area	Height	Conc.
1	12.046	444599	26397	3.003
2	15.760	14362138	640549	96.997
Total		14806736	666946	

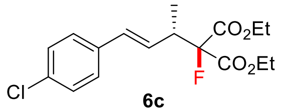

6c

Analysis Report

<Sample Information>

Sample Name : lil-ld-65-rac-adh-98-2-1.0-254-230-1
Sample ID :
Data Filename : lil-ld-65-rac-adh-98-2-1.0-254-230-1.lcd
Method Filename : xsl-230-254-1.0.lcm
Batch Filename :
Vial # : 1-1 Sample Type : Unknown
Injection Volume : 20 uL
Date Acquired : 2021/10/19 19:08:59 Acquired by : System Administrator
Date Processed : 2021/10/19 19:25:27 Processed by : System Administrator

<Chromatogram>

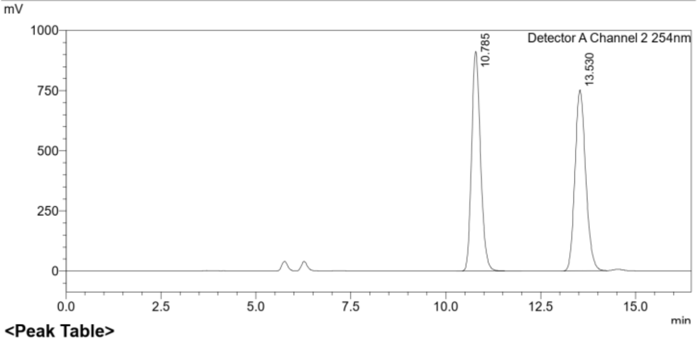

<Peak Table>

Detector A Channel 2 254nm

Peak#	Ret. Time	Area	Height	Conc.
1	10.785	14883679	912931	50.019
2	13.530	14872243	752408	49.981
Total		29755922	1665339	

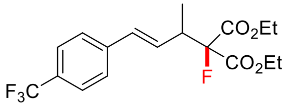

rac-**6d**

Analysis Report

<Sample Information>

Sample Name : lil-ld-65-asy-adh-98-2-1.0-254-230-1
Sample ID :
Data Filename : lil-ld-65-asy-adh-98-2-1.0-254-230-1.lcd
Method Filename : xsl-230-254-1.0.lcm
Batch Filename :
Vial # : 1-1 Sample Type : Unknown
Injection Volume : 20 uL
Date Acquired : 2021/10/19 18:24:08 Acquired by : System Administrator
Date Processed : 2021/10/19 18:40:47 Processed by : System Administrator

<Chromatogram>

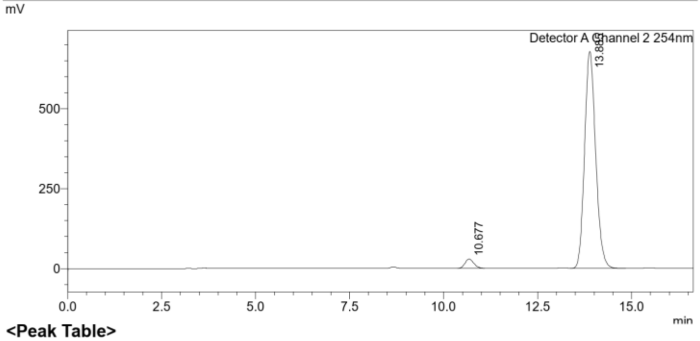

<Peak Table>

Detector A Channel 2 254nm

Peak#	Ret. Time	Area	Height	Conc.
1	10.677	474700	29069	3.296
2	13.885	13928903	678658	96.704
Total		14403603	707726	

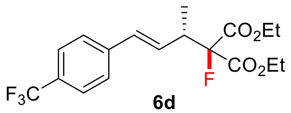

6d

90 lil-II-cooet-rac-ojh-85-15-230-0.5-2

Sample Name:	**lil-II-cooet-rac-ojh-85-15-230-0.5-2**	*Injection Volume:*	**20.0**
Vial Number:	**452**	*Channel:*	**UV_VIS_1**
Sample Type:	**standard**	*Wavelength:*	**230**
Control Program:	**XSL**	*Bandwidth:*	**n.a.**
Quantif. Method:	**XSL**	*Dilution Factor:*	**1.0000**
Run Time (min):	**29.18**	*Sample Amount:*	**1.0000**

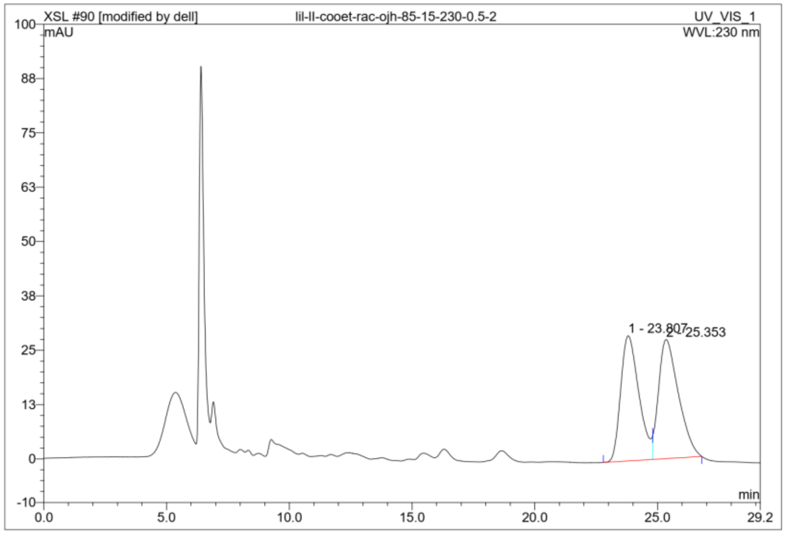

No.	Ret.Time min	Peak Name	Height mAU	Area mAU*min	Rel.Area %	Amount	Type
1	23.81	n.a.	28.785	24.800	49.11	n.a.	BM *
2	25.35	n.a.	27.387	25.701	50.89	n.a.	MB*
Total:			56.171	50.501	100.00	0.000	

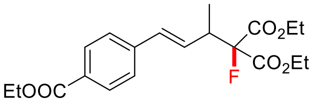

rac-**6e**

104 lil-II-COOET-ASI-OJH-230-1.0-3

Sample Name:	**lil-II-COOET-ASI-OJH-230-1.0-3**	*Injection Volume:*	**20.0**
Vial Number:	**466**	*Channel:*	**UV_VIS_1**
Sample Type:	**standard**	*Wavelength:*	**230**
Control Program:	**XSL**	*Bandwidth:*	**n.a.**
Quantif. Method:	**XSL**	*Dilution Factor:*	**1.0000**
Run Time (min):	**31.49**	*Sample Amount:*	**1.0000**

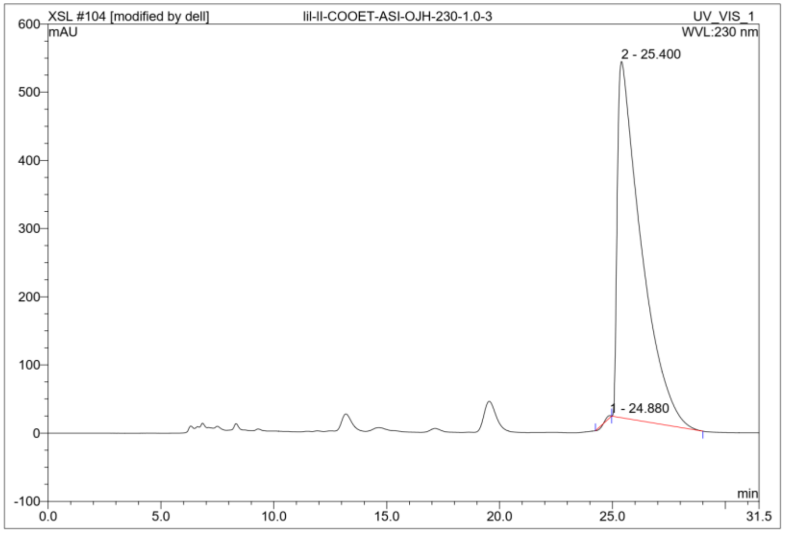

No.	Ret.Time min	Peak Name	Height mAU	Area mAU*min	Rel.Area %	Amount	Type
1	24.88	n.a.	3.133	0.372	0.06	n.a.	BMB*
2	25.40	n.a.	522.265	635.282	99.94	n.a.	BMB
Total:			525.398	635.653	100.00	0.000	

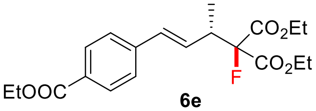

6e

Operator:dell Timebase:U-3000 Sequence:XSL

Page 1-1
2008-10-19 12:51 上午

44 lil-lh--cn-rac-ojh-95-5-254-1.0-1

Sample Name:	**lil-lh--cn-rac-ojh-95-5-254-1.0-1**	*Injection Volume:*	**20.0**
Vial Number:	**404**	*Channel:*	**UV_VIS_1**
Sample Type:	**standard**	*Wavelength:*	**254**
Control Program:	**XSL**	*Bandwidth:*	**n.a.**
Quantif. Method:	**XSL**	*Dilution Factor:*	**1.0000**
Run Time (min):	**44.43**	*Sample Amount:*	**1.0000**

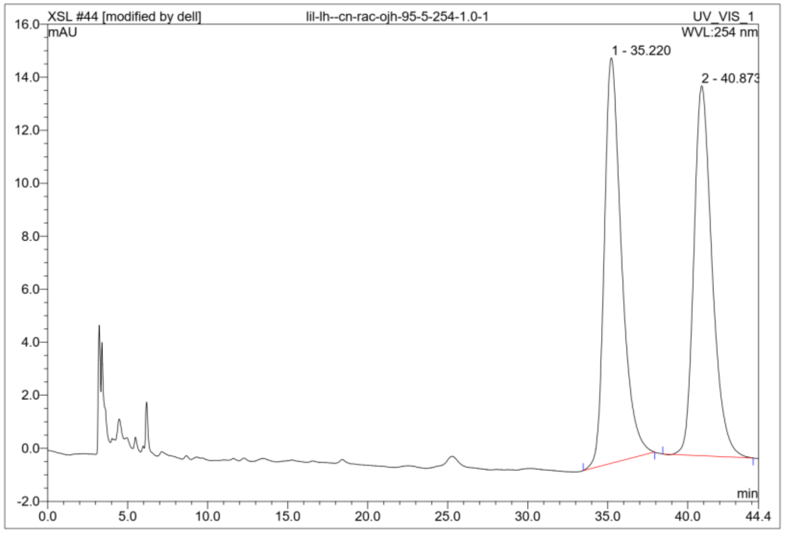

No.	Ret.Time min	Peak Name	Height mAU	Area mAU*min	Rel.Area %	Amount	Type
1	35.22	n.a.	15.294	19.169	50.93	n.a.	BMB*
2	40.87	n.a.	13.954	18.467	49.07	n.a.	BMB*
**Total:**			29.248	37.635	100.00	0.000	

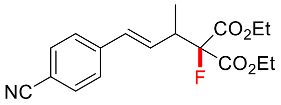

rac-**6f**

Mu/Integration

Chromeleon (c) Dionex 1996-2006
Version 6.80 SR8a Build 2643 (158225)

Operator:dell Timebase:U-3000 Sequence:XSL

Page 1-1
2008-10-19 12:50 上午

67 lil-ll-31-cn-ojh-asy-95-5--230-1.0-3

Sample Name:	**lil-ll-31-cn-ojh-asy-95-5--230-1.0-3**	*Injection Volume:*	**20.0**
Vial Number:	**428**	*Channel:*	**UV_VIS_1**
Sample Type:	**standard**	*Wavelength:*	**230**
Control Program:	**XSL**	*Bandwidth:*	**n.a.**
Quantif. Method:	**XSL**	*Dilution Factor:*	**1.0000**
Run Time (min):	**62.89**	*Sample Amount:*	**1.0000**

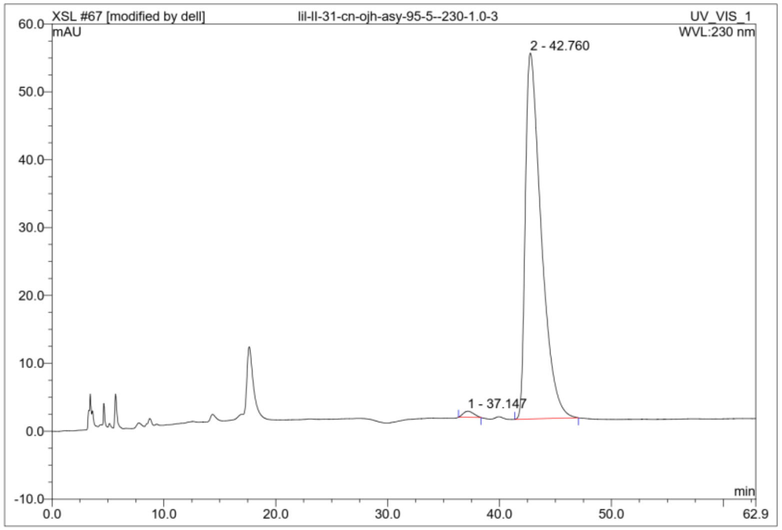

No.	Ret.Time min	Peak Name	Height mAU	Area mAU*min	Rel.Area %	Amount	Type
1	37.15	n.a.	0.901	0.985	1.10	n.a.	BMB*
2	42.76	n.a.	53.911	88.515	98.90	n.a.	BMB
**Total:**			54.812	89.500	100.00	0.000	

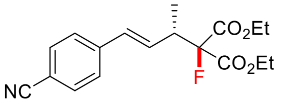

6f

Mu/Integration

Chromeleon (c) Dionex 1996-2006
Version 6.80 SR8a Build 2643 (158225)

64 lil-II-19-coch3-adh-rac-95-5--230-1.0-1

Sample Name:	**lil-II-19-coch3-adh-rac-95-5--230-1.0-1**	*Injection Volume:*	**20.0**
Vial Number:	**425**	*Channel:*	**UV_VIS_1**
Sample Type:	**standard**	*Wavelength:*	**230**
Control Program:	**XSL**	*Bandwidth:*	**n.a.**
Quantif. Method:	**XSL**	*Dilution Factor:*	**1.0000**
Run Time (min):	**22.89**	*Sample Amount:*	**1.0000**

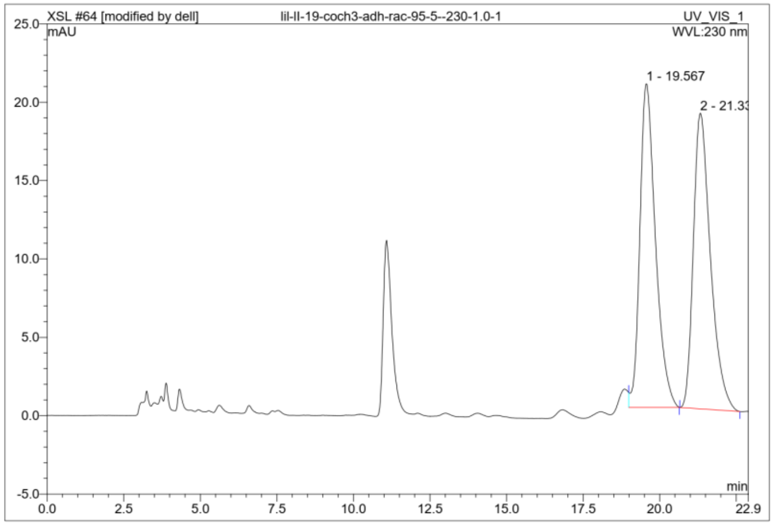

No.	Ret.Time min	Peak Name	Height mAU	Area mAU*min	Rel.Area %	Amount	Type
1	19.57	n.a.	20.655	12.204	49.98	n.a.	MB*
2	21.33	n.a.	18.877	12.214	50.02	n.a.	BMB
**Total:**			39.532	24.419	100.00	0.000	

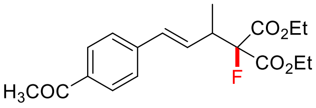

rac-**6g**

66 lil-II-19-coch3-adh-asy-95-5--230-1.0-3

Sample Name:	**lil-II-19-coch3-adh-asy-95-5--230-1.0-3**	*Injection Volume:*	**20.0**
Vial Number:	**427**	*Channel:*	**UV_VIS_1**
Sample Type:	**standard**	*Wavelength:*	**230**
Control Program:	**XSL**	*Bandwidth:*	**n.a.**
Quantif. Method:	**XSL**	*Dilution Factor:*	**1.0000**
Run Time (min):	**25.29**	*Sample Amount:*	**1.0000**

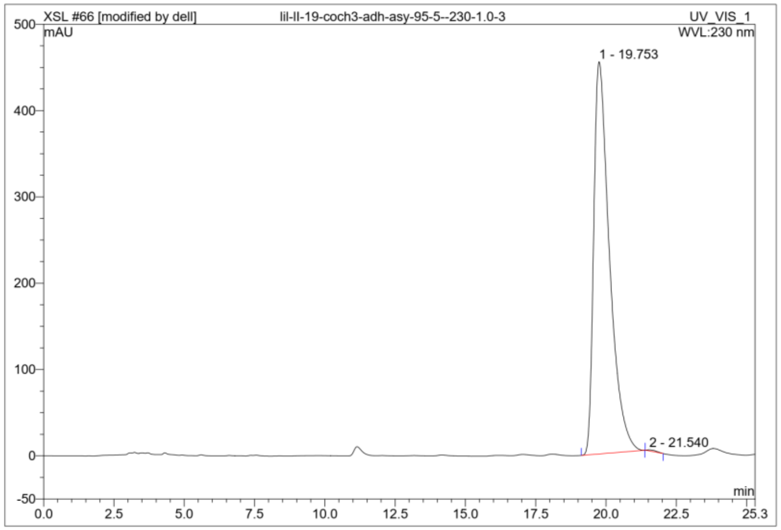

No.	Ret.Time min	Peak Name	Height mAU	Area mAU*min	Rel.Area %	Amount	Type
1	19.75	n.a.	454.452	294.346	99.80	n.a.	BMB
2	21.54	n.a.	1.306	0.580	0.20	n.a.	BMB*
**Total:**			455.758	294.926	100.00	0.000	

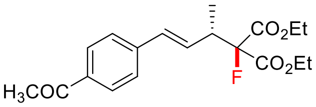

6g

83 lil-II-cho-rac-ojh-75-25--230-1.0-2

Sample Name:	**lil-II-cho-rac-ojh-75-25--230-1.0-2**	*Injection Volume:*	**20.0**
Vial Number:	**444**	*Channel:*	**UV_VIS_1**
Sample Type:	**standard**	*Wavelength:*	**230**
Control Program:	**XSL**	*Bandwidth:*	**n.a.**
Quantif. Method:	**XSL**	*Dilution Factor:*	**1.0000**
Run Time (min):	**31.95**	*Sample Amount:*	**1.0000**

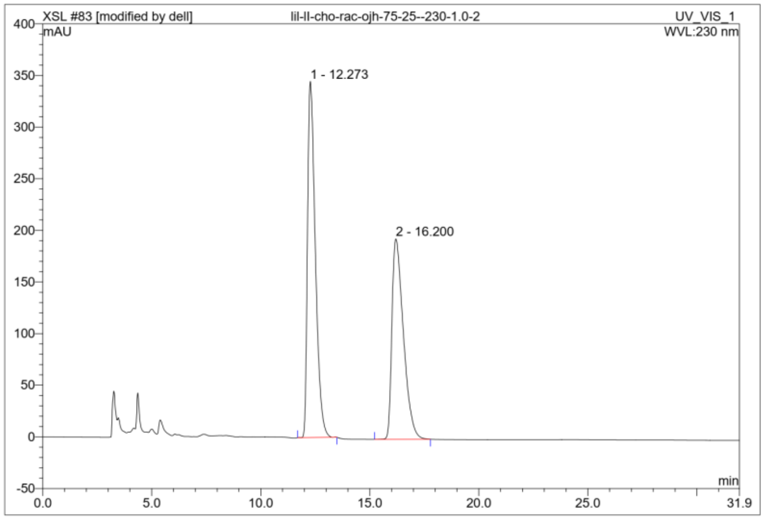

No.	Ret.Time min	Peak Name	Height mAU	Area mAU*min	Rel.Area %	Amount	Type
1	12.27	n.a.	344.902	143.496	54.77	n.a.	BMB*
2	16.20	n.a.	194.040	118.479	45.23	n.a.	BMB*
Total:			538.942	261.976	100.00	0.000	

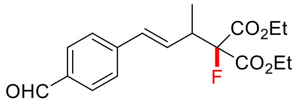

rac-**6h**

84 lil-II-cho-asy-ojh-95-5--230-1.0-2

Sample Name:	**lil-II-cho-asy-ojh-95-5--230-1.0-2**	*Injection Volume:*	**20.0**
Vial Number:	**445**	*Channel:*	**UV_VIS_1**
Sample Type:	**standard**	*Wavelength:*	**230**
Control Program:	**XSL**	*Bandwidth:*	**n.a.**
Quantif. Method:	**XSL**	*Dilution Factor:*	**1.0000**
Run Time (min):	**17.50**	*Sample Amount:*	**1.0000**

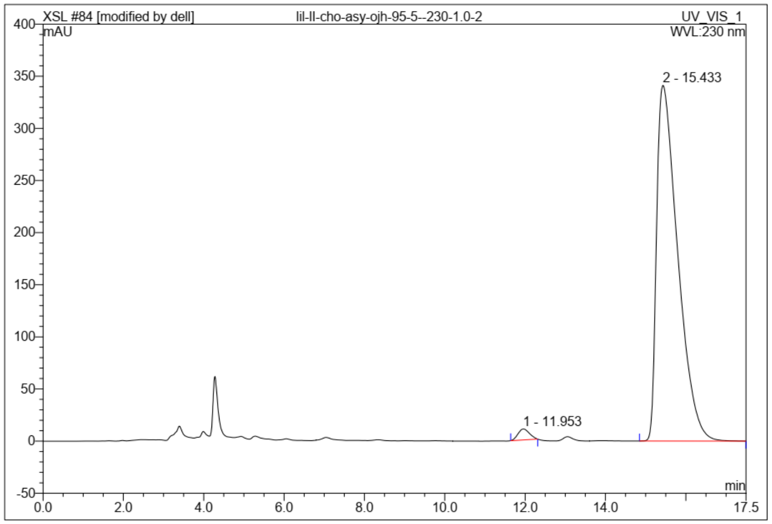

No.	Ret.Time min	Peak Name	Height mAU	Area mAU*min	Rel.Area %	Amount	Type
1	11.95	n.a.	10.524	3.450	1.64	n.a.	BMB*
2	15.43	n.a.	341.140	207.471	98.36	n.a.	BMB
Total:			351.664	210.921	100.00	0.000	

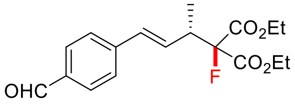

6h

Analysis Report

<Sample Information>

Sample Name	: lil-ldm-f-rac-adh-98-2-1.0-254-230-1		
Sample ID	:		
Data Filename	: lil-ldm-f-rac-adh-98-2-1.0-254-230-1.lcd		
Method Filename	: xsl-230-254-1.0.lcm		
Batch Filename	:		
Vial #	: 1-1	Sample Type	: Unknown
Injection Volume	: 20 uL		
Date Acquired	: 2021/10/17 21:37:10	Acquired by	: System Administrator
Date Processed	: 2021/10/17 21:54:48	Processed by	: System Administrator

<Chromatogram>

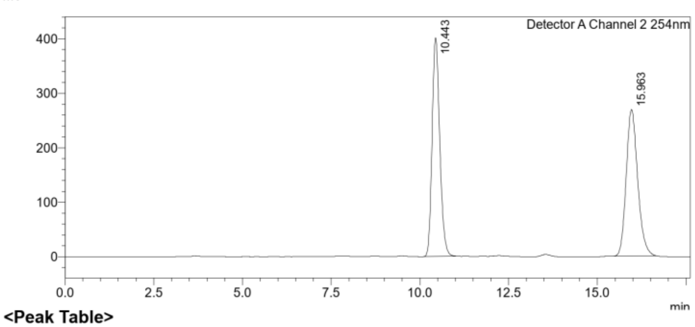

<Peak Table>

Detector A Channel 2 254nm

Peak#	Ret. Time	Area	Height	Conc.
1	10.443	5993496	401111	49.712
2	15.963	6062935	268945	50.288
Total		12056432	670057	

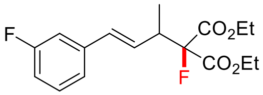

rac-**6i**

Analysis Report

<Sample Information>

Sample Name	: lil-ld-m-f--asy-adh-98-2-1.0-254-230-1		
Sample ID	:		
Data Filename	: lil-ld-m-f--asy-adh-98-2-1.0-254-230-1.lcd		
Method Filename	: xsl-230-254-1.0.lcm		
Batch Filename	:		
Vial #	: 1-1	Sample Type	: Unknown
Injection Volume	: 20 uL		
Date Acquired	: 2021/10/17 16:58:01	Acquired by	: System Administrator
Date Processed	: 2021/10/17 17:29:25	Processed by	: System Administrator

<Chromatogram>

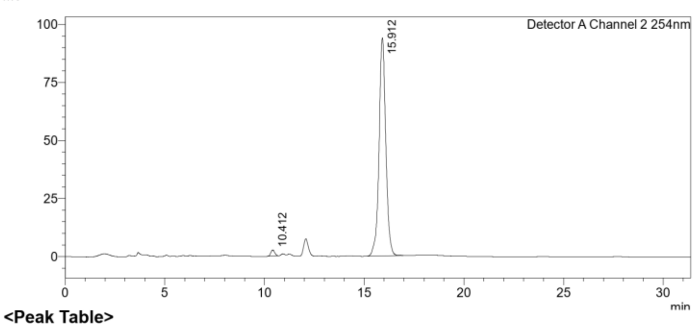

<Peak Table>

Detector A Channel 2 254nm

Peak#	Ret. Time	Area	Height	Conc.
1	10.412	35342	2587	1.577
2	15.912	2205091	93929	98.423
Total		2240433	96517	

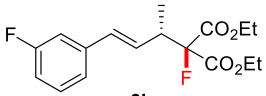

6i

Analysis Report

<Sample Information>

Sample Name	: lil-ld-95-rac-adh-98-2-1.0-254-230-1		
Sample ID	:		
Data Filename	: lil-ld-95-rac-adh-98-2-1.0-254-230-1.lcd		
Method Filename	: gay-1.0-254-1.lcm		
Batch Filename	:		
Vial #	: 1-1	Sample Type	: Unknown
Injection Volume	: 20 uL		
Date Acquired	: 2021/11/5 23:18:59	Acquired by	: System Administrator
Date Processed	: 2021/11/5 23:33:47	Processed by	: System Administrator

<Chromatogram>

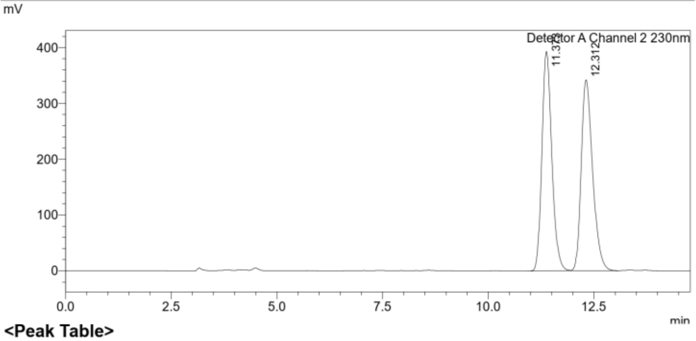

<Peak Table>

Detector A Channel 2 230nm

Peak#	Ret. Time	Area	Height	Conc.
1	11.373	6465052	393188	49.995
2	12.312	6466442	341893	50.005
Total		12931493	735081	

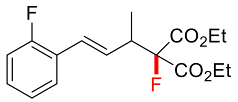

rac-**6j**

Analysis Report

<Sample Information>

Sample Name	: lil-ld-95-asy-adh-98-2-1.0-254-230-1		
Sample ID	:		
Data Filename	: lil-ld-95-asy-adh-98-2-1.0-254-230-1.lcd		
Method Filename	: gay-1.0-254-1.lcm		
Batch Filename	:		
Vial #	: 1-1	Sample Type	: Unknown
Injection Volume	: 20 uL		
Date Acquired	: 2021/11/5 22:25:21	Acquired by	: System Administrator
Date Processed	: 2021/11/5 23:10:05	Processed by	: System Administrator

<Chromatogram>

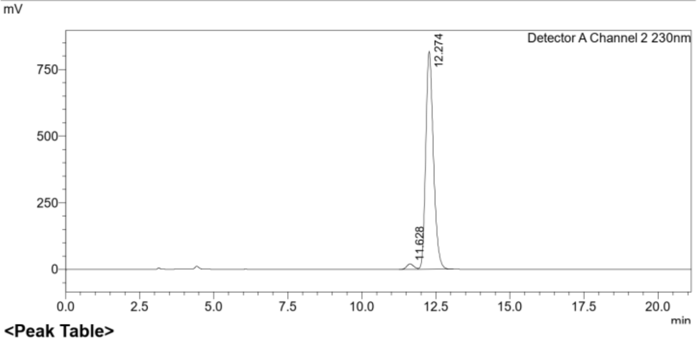

<Peak Table>

Detector A Channel 2 230nm

Peak#	Ret. Time	Area	Height	Conc.
1	11.628	343685	20362	2.276
2	12.274	14755555	816637	97.724
Total		15099240	836999	

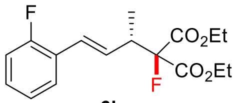

6j

Operator:dell Timebase:U-3000 Sequence:XSL

Page 1-1
2008-10-15 12:49 上午

184 lil-II-2CF3-ADH-RAC-95-5--230-1.0-1

Sample Name:	**lil-II-2CF3-ADH-RAC-95-5--230-1.0-1**	*Injection Volume:*	**20.0**
Vial Number:	**410**	*Channel:*	**UV_VIS_1**
Sample Type:	**standard**	*Wavelength:*	**230**
Control Program:	**XSL**	*Bandwidth:*	**n.a.**
Quantif. Method:	**XSL**	*Dilution Factor:*	**1.0000**
Run Time (min):	**8.88**	*Sample Amount:*	**1.0000**

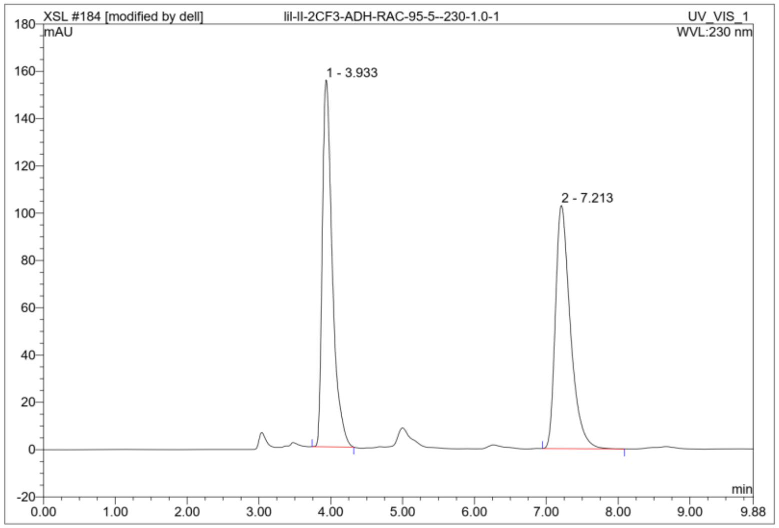

No.	Ret.Time min	Peak Name	Height mAU	Area mAU*min	Rel.Area %	Amount	Type
1	3.93	n.a.	155.159	25.076	50.29	n.a.	BMB*
2	7.21	n.a.	102.827	24.791	49.71	n.a.	BMB
Total:			257.986	49.867	100.00	0.000	

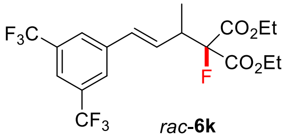

rac-**6k**

Mu/Integration

Chromeleon (c) Dionex 1996-2006
Version 6.80 SR8a Build 2643 (158225)

Operator:dell Timebase:U-3000 Sequence:XSL

Page 1-1
2008-10-15 12:37 上午

183 lil-II-2CF3-ADH-ASY-95-5--230-1.0-1

Sample Name:	**lil-II-2CF3-ADH-ASY-95-5--230-1.0-1**	*Injection Volume:*	**20.0**
Vial Number:	**409**	*Channel:*	**UV_VIS_1**
Sample Type:	**standard**	*Wavelength:*	**230**
Control Program:	**XSL**	*Bandwidth:*	**n.a.**
Quantif. Method:	**XSL**	*Dilution Factor:*	**1.0000**
Run Time (min):	**10.57**	*Sample Amount:*	**1.0000**

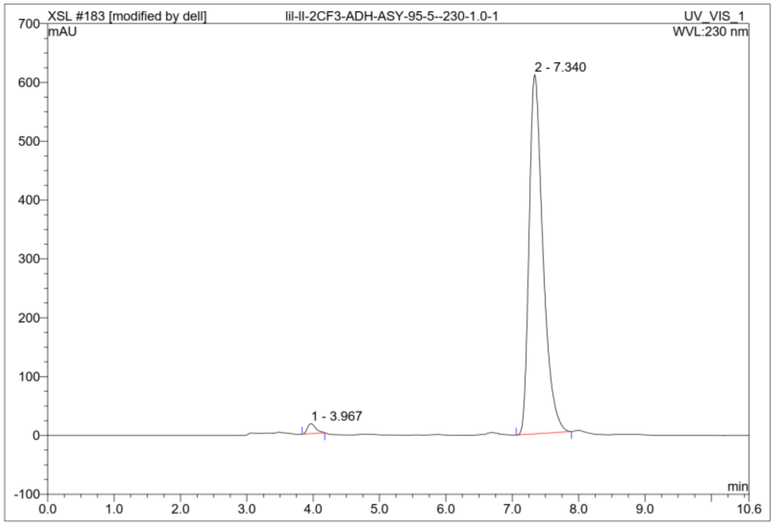

No.	Ret.Time min	Peak Name	Height mAU	Area mAU*min	Rel.Area %	Amount	Type
1	3.97	n.a.	17.017	2.472	1.64	n.a.	BMB*
2	7.34	n.a.	610.775	147.975	98.36	n.a.	BMB
Total:			627.792	150.447	100.00	0.000	

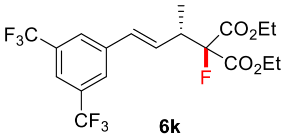

6k

Mu/Integration

Chromeleon (c) Dionex 1996-2006
Version 6.80 SR8a Build 2643 (158225)

Analysis Report

<Sample Information>

Sample Name : lil-ld-rac--ch3--adh-98-2-1.0-230-254nm-2
Sample ID :
Data Filename : lil-ld-rac--ch3--adh-98-2-1.0-230-254nm-2.lcd
Method Filename : ZXX-erchun.lcm
Batch Filename :
Vial # : 1-1
Sample Type : Unknown
Injection Volume : 20 uL
Date Acquired : 2021/10/9 20:34:01
Acquired by : System Administrator
Date Processed : 2021/10/9 20:47:26
Processed by : System Administrator

<Chromatogram>

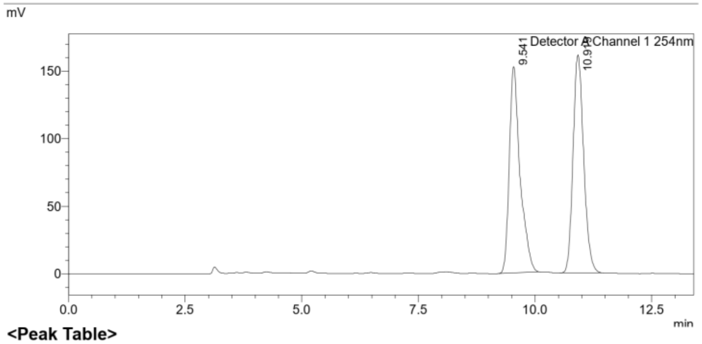

<Peak Table>

Detector A Channel 1 254nm

Peak#	Ret. Time	Area	Height	Conc.
1	9.541	2459151	152683	49.081
2	10.919	2551222	161456	50.919
Total		5010373	314139	

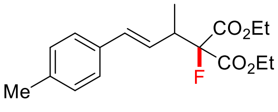

rac-**6l**

Analysis Report

<Sample Information>

Sample Name : lil-ld-25-3-ch3-asy-adh-98-2-1.0-230-254nm-2
Sample ID :
Data Filename : lil-ld-25-3-ch3-asy-adh-98-2-1.0-230-254nm-2.lcd
Method Filename : ZXX-erchun.lcm
Batch Filename :
Vial # : 1-1
Sample Type : Unknown
Injection Volume : 20 uL
Date Acquired : 2021/10/9 19:49:01
Acquired by : System Administrator
Date Processed : 2022/1/10 9:16:44
Processed by : System Administrator

<Chromatogram>

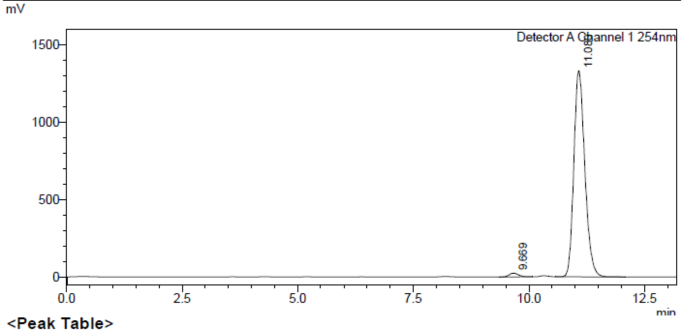

<Peak Table>

Detector A Channel 1 254nm

Peak#	Ret. Time	Area	Height	Conc.
1	9.669	342424	24012	1.531
2	11.080	22027480	1333743	98.469
Total		22369905	1357755	

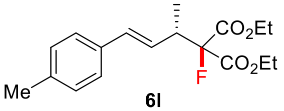

6l

Operator:dell Timebase:U-3000 Sequence:XSL

Page 1-1
2008-10-23 5:57 上午

59 lil-II-21-ojh-rac-ojh-95-5--230-1.0-1

Sample Name:	**lil-II-21-ojh-rac-ojh-95-5--230-1.0-1**	*Injection Volume:*	**20.0**
Vial Number:	**420**	*Channel:*	**UV_VIS_1**
Sample Type:	**standard**	*Wavelength:*	**230**
Control Program:	**XSL**	*Bandwidth:*	**n.a.**
Quantif. Method:	**XSL**	*Dilution Factor:*	**1.0000**
Run Time (min):	**68.66**	*Sample Amount:*	**1.0000**

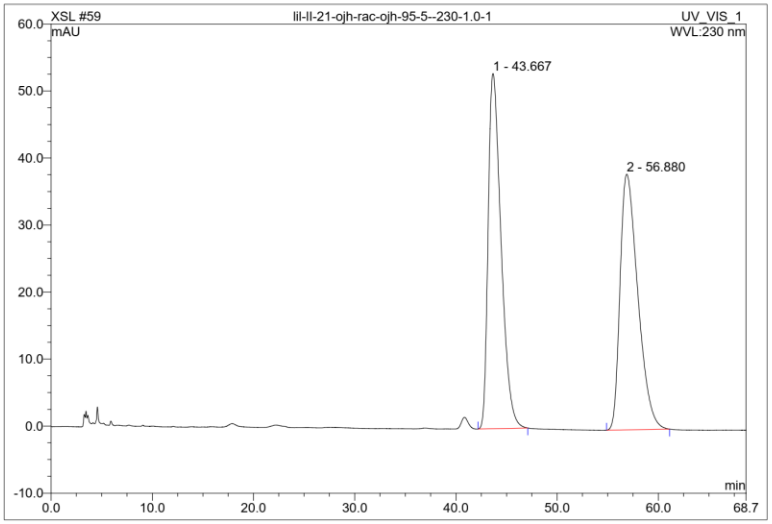

No.	Ret.Time min	Peak Name	Height mAU	Area mAU*min	Rel.Area %	Amount	Type
1	43.67	n.a.	52.988	77.887	50.11	n.a.	BMB
2	56.88	n.a.	38.142	77.539	49.89	n.a.	BMB
**Total:**			91.129	155.427	100.00	0.000	

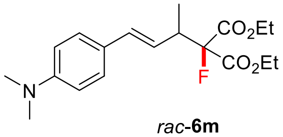

rac-**6m**

Mu/Integration

Chromeleon (c) Dionex 1996-2006
Version 6.80 SR8a Build 2643 (158225)

Operator:dell Timebase:U-3000 Sequence:XSL

Page 1-1
2008-10-23 5:58 上午

58 lil-II-21-ojh-asy-ojh-95-5--230-1.0-1

Sample Name:	**lil-II-21-ojh-asy-ojh-95-5--230-1.0-1**	*Injection Volume:*	**20.0**
Vial Number:	**419**	*Channel:*	**UV_VIS_1**
Sample Type:	**standard**	*Wavelength:*	**230**
Control Program:	**XSL**	*Bandwidth:*	**n.a.**
Quantif. Method:	**XSL**	*Dilution Factor:*	**1.0000**
Run Time (min):	**64.18**	*Sample Amount:*	**1.0000**

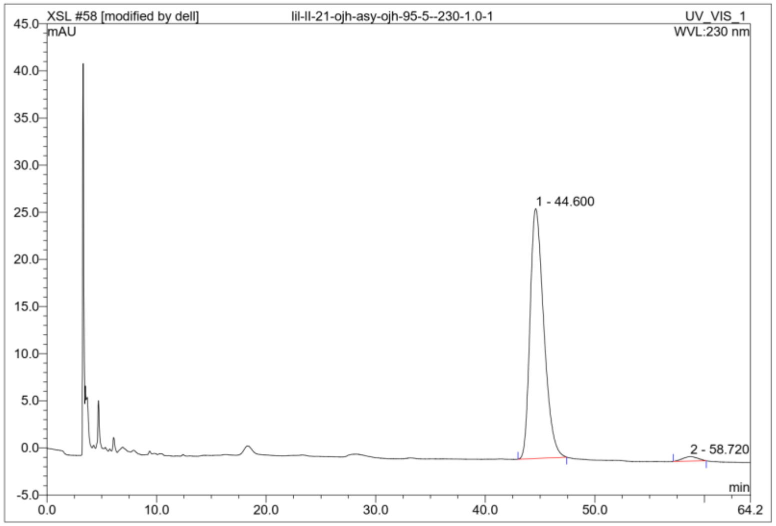

No.	Ret.Time min	Peak Name	Height mAU	Area mAU*min	Rel.Area %	Amount	Type
1	44.60	n.a.	26.499	38.499	98.12	n.a.	BMB
2	58.72	n.a.	0.459	0.736	1.88	n.a.	BMB*
**Total:**			26.958	39.235	100.00	0.000	

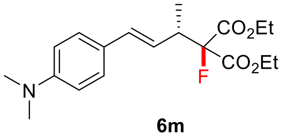

6m

Mu/Integration

Chromeleon (c) Dionex 1996-2006
Version 6.80 SR8a Build 2643 (158225)

Operator:dell Timebase:U-3000 Sequence:XSL Page 1-1
2008-10-15 6:24 上午

189 lil-II-ENE-IF-RAC-97-3--230-0.5-2

Sample Name:	**lil-II-ENE-IF-RAC-97-3--230-0.5-2**	*Injection Volume:*	**20.0**
Vial Number:	**416**	*Channel:*	**UV_VIS_1**
Sample Type:	**standard**	*Wavelength:*	**230**
Control Program:	**XSL**	*Bandwidth:*	**n.a.**
Quantif. Method:	**XSL**	*Dilution Factor:*	**1.0000**
Run Time (min):	**17.17**	*Sample Amount:*	**1.0000**

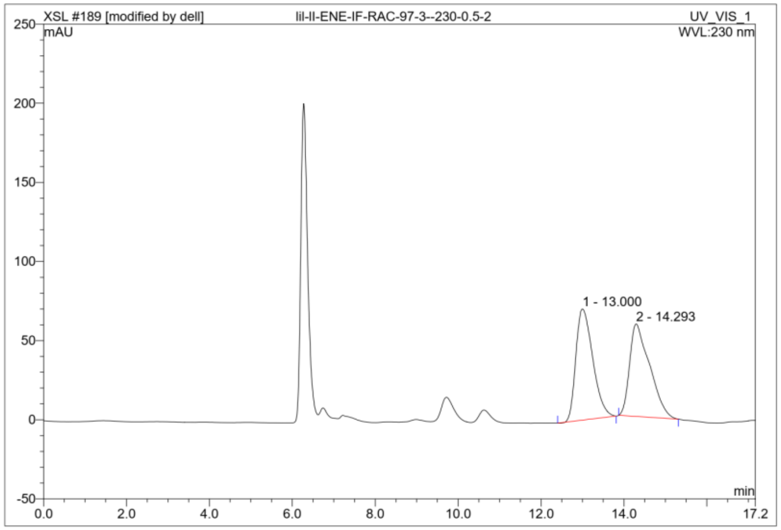

No.	Ret.Time min	Peak Name	Height mAU	Area mAU*min	Rel.Area %	Amount	Type
1	13.00	n.a.	70.224	33.157	50.25	n.a.	BMB*
2	14.29	n.a.	58.390	32.823	49.75	n.a.	BMB*
**Total:**			128.615	65.980	100.00	0.000	

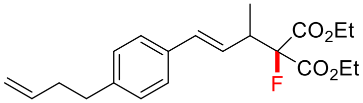

rac-**6n**

Mu/Integration

Chromeleon (c) Dionex 1996-2006
Version 6.80 SR8a Build 2643 (158225)

Operator:dell Timebase:U-3000 Sequence:XSL Page 1-1
2008-10-19 12:54 上午

62 lil-II-20-1-ene-if-asy-95-5--230-0.5-2

Sample Name:	**lil-II-20-1-ene-if-asy-95-5--230-0.5-2**	*Injection Volume:*	**20.0**
Vial Number:	**423**	*Channel:*	**UV_VIS_1**
Sample Type:	**standard**	*Wavelength:*	**230**
Control Program:	**XSL**	*Bandwidth:*	**n.a.**
Quantif. Method:	**XSL**	*Dilution Factor:*	**1.0000**
Run Time (min):	**15.81**	*Sample Amount:*	**1.0000**

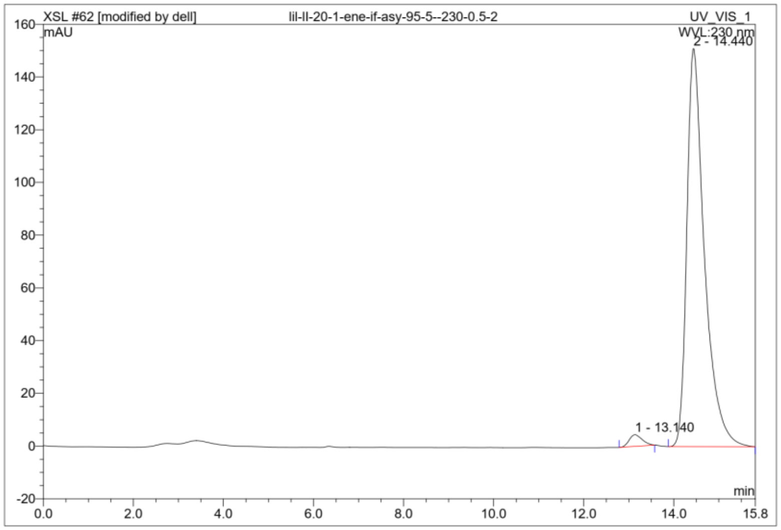

No.	Ret.Time min	Peak Name	Height mAU	Area mAU*min	Rel.Area %	Amount	Type
1	13.14	n.a.	4.415	1.479	2.12	n.a.	BMB*
2	14.44	n.a.	151.062	68.417	97.88	n.a.	BMB
**Total:**			155.477	69.896	100.00	0.000	

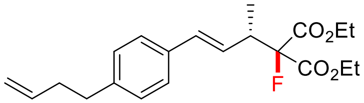

6n

Mu/Integration

Chromeleon (c) Dionex 1996-2006
Version 6.80 SR8a Build 2643 (158225)

Analysis Report

<Sample Information>

Sample Name : lil-lc-p-ome-rac--oxh-99-1-1.0-230nm-01-2
Sample ID :
Data Filename : lil-lc-p-ome-rac--oxh-99-1-1.0-230nm-01-2.lcd
Method Filename : xsl-230-254-1.0.lcm
Batch Filename :
Vial # : 1-1 Sample Type : Unknown
Injection Volume : 20 uL
Date Acquired : 2021/9/17 22:33:31 Acquired by : System Administrator
Date Processed : 2021/9/17 22:59:28 Processed by : System Administrator

<Chromatogram>

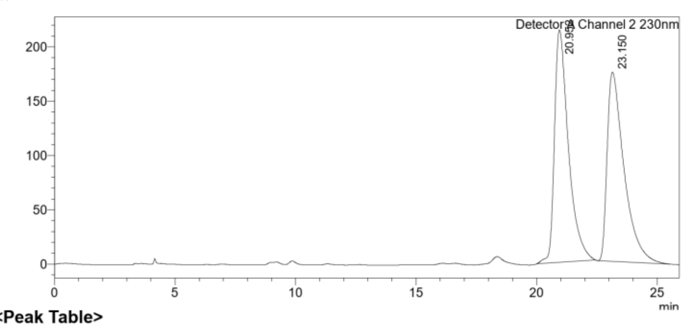

<Peak Table>

Detector A Channel 2 230nm

Peak#	Ret. Time	Area	Height	Conc.
1	20.952	8626320	213902	50.386
2	23.150	8494174	174235	49.614
Total		17120494	388136	

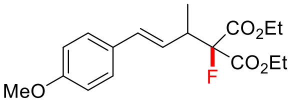

rac-**6o**

Analysis Report

<Sample Information>

Sample Name : lil-ld-46-4-p-ome-asy-oxh-99-1-1.0-254-230-1
Sample ID :
Data Filename : lil-ld-46-4-p-ome-asy-oxh-99-1-1.0-254-230-1.lcd
Method Filename : xsl-230-254-1.0.lcm
Batch Filename :
Vial # : 1-1 Sample Type : Unknown
Injection Volume : 20 uL
Date Acquired : 2021/10/17 22:40:37 Acquired by : System Administrator
Date Processed : 2021/10/17 23:05:14 Processed by : System Administrator

<Chromatogram>

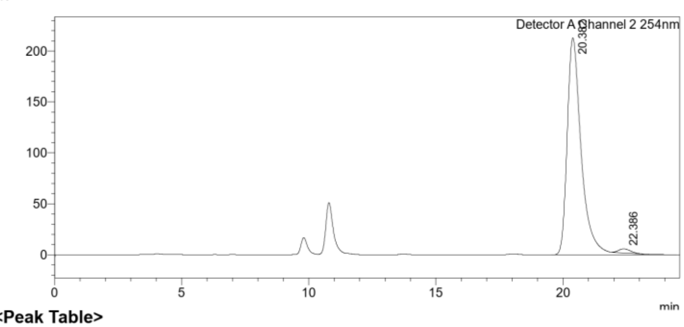

<Peak Table>

Detector A Channel 2 254nm

Peak#	Ret. Time	Area	Height	Conc.
1	20.382	8297643	213158	98.501
2	22.386	126288	3903	1.499
Total		8423931	217061	

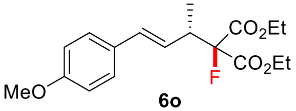

6o

Analysis Report

<Sample Information>

Sample Name	: lil-ld-m-ome-rac-oxh-99-1--1.0-254-230-1		
Sample ID	:		
Data Filename	: lil-ld-m-ome-rac-ojh-99-1--1.0-254-230-1.lcd		
Method Filename	: xsl-230-254-1.0.lcm		
Batch Filename	:		
Vial #	: 1-1	Sample Type	: Unknown
Injection Volume	: 20 uL		
Date Acquired	: 2021/12/23 16:16:40	Acquired by	: System Administrator
Date Processed	: 2021/12/23 17:17:47	Processed by	: System Administrator

<Chromatogram>

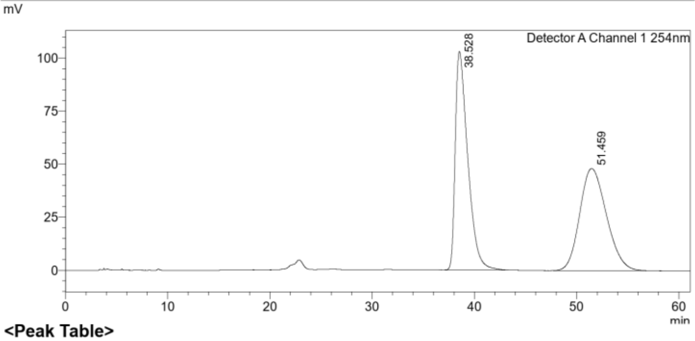

<Peak Table>

Detector A Channel 1 254nm

Peak#	Ret. Time	Area	Height	Conc.
1	38.528	8752090	102957	50.064
2	51.459	8729737	48129	49.936
Total		17481827	151086	

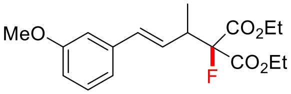

rac-**6p**

Analysis Report

<Sample Information>

Sample Name	: lil-ld-m-ome-asy-ojh-99-1--1.0-254-230-1		
Sample ID	:		
Data Filename	: lil-ld-m-ome-asy-ojh-99-1--1.0-254-230-1.lcd		
Method Filename	: xsl-230-254-1.0.lcm		
Batch Filename	:		
Vial #	: 1-1	Sample Type	: Unknown
Injection Volume	: 20 uL		
Date Acquired	: 2021/12/23 17:19:41	Acquired by	: System Administrator
Date Processed	: 2021/12/23 18:23:19	Processed by	: System Administrator

<Chromatogram>

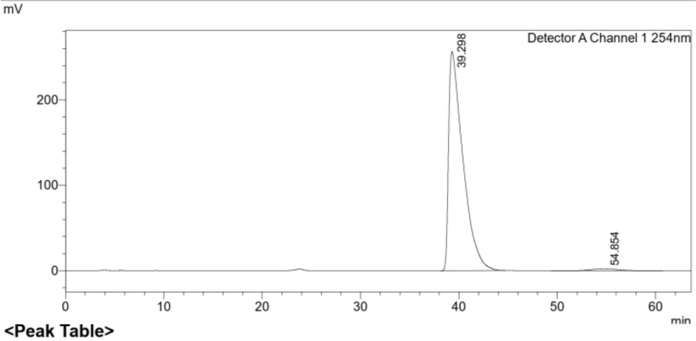

<Peak Table>

Detector A Channel 1 254nm

Peak#	Ret. Time	Area	Height	Conc.
1	39.298	26000860	256690	98.227
2	54.854	469368	2387	1.773
Total		26470228	259077	

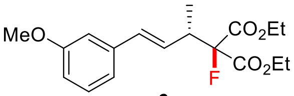

6p

Analysis Report

<Sample Information>

Sample Name : LIL-LD-O-OME-RAC-OXH-98-2-254-230-1
Sample ID :
Data Filename : LIL-LD-O-OME-RAC-OXH-98-2-254-230-1.lcd
Method Filename : gay-1.0-254-1.lcm
Batch Filename :
Vial # : 1-1
Injection Volume : 10 uL
Date Acquired : 2022/1/10 9:35:54
Date Processed : 2022/1/10 9:52:24

Sample Type : Unknown
Acquired by : System Administrator
Processed by : System Administrator

<Chromatogram>

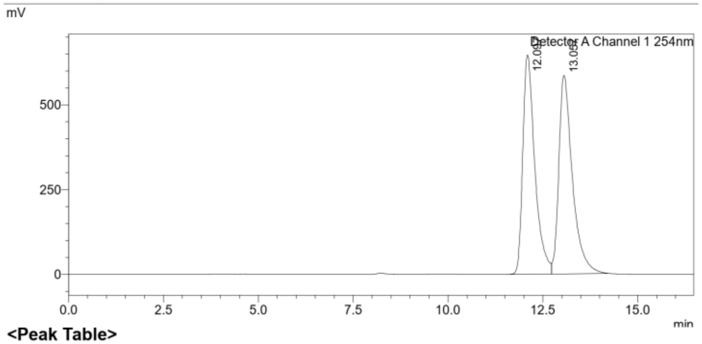

<Peak Table>

Detector A Channel 1 254nm

Peak#	Ret. Time	Area	Height	Conc.
1	12.097	14283545	646520	49.498
2	13.057	14573498	585382	50.502
Total		28857044	1231902	

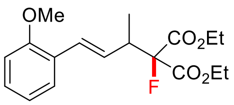

rac-**6q**

Analysis Report

<Sample Information>

Sample Name : LIL-LD-O-OME-ASY-ADH-98-2-254-230-1
Sample ID :
Data Filename : LIL-LD-O-OME-ASY-ADH-98-2-254-230-1.lcd
Method Filename : gay-1.0-254-1.lcm
Batch Filename :
Vial # : 1-1
Injection Volume : 10 uL
Date Acquired : 2022/1/10 9:09:44
Date Processed : 2022/1/10 9:27:59

Sample Type : Unknown
Acquired by : System Administrator
Processed by : System Administrator

<Chromatogram>

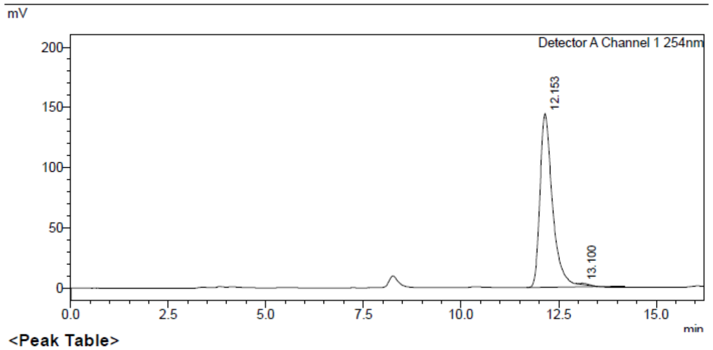

<Peak Table>

Detector A Channel 1 254nm

Peak#	Ret. Time	Area	Height	Conc.
1	12.153	3221014	144187	99.484
2	13.100	16691	1013	0.516
Total		3237705	145200	

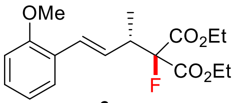

6q

Analysis Report

<Sample Information>

Sample Name : lil-ld-39-4-RAC-90-10-1.0-230-254nm-1
Sample ID :
Data Filename : lil-ld-39-4-RAC-90-10-1.0-230-254nm-1.lcd
Method Filename : xsl-230-254-1.0.lcm
Batch Filename :
Vial # : 1-1 Sample Type : Unknown
Injection Volume : 20 uL
Date Acquired : 2021/10/12 19:57:06 Acquired by : System Administrator
Date Processed : 2021/10/12 20:37:22 Processed by : System Administrator

<Chromatogram>

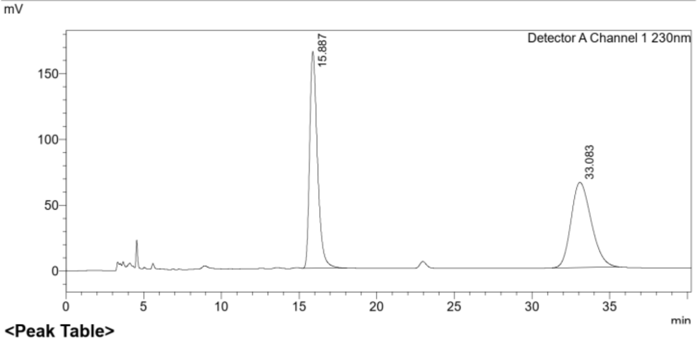

<Peak Table>

Detector A Channel 1 230nm

Peak#	Ret. Time	Area	Height	Conc.
1	15.887	5739438	164770	49.554
2	33.083	5842799	64688	50.446
Total		11582237	229458	

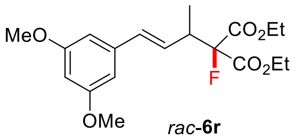

rac-**6r**

Analysis Report

<Sample Information>

Sample Name : lil-ld-39-4-asy-90-10-1.0-230-254nm-1
Sample ID :
Data Filename : lil-ld-39-4-asy-90-10-1.0-230-254nm-1.lcd
Method Filename : xsl-230-254-1.0.lcm
Batch Filename :
Vial # : 1-1 Sample Type : Unknown
Injection Volume : 20 uL
Date Acquired : 2021/10/12 18:42:17 Acquired by : System Administrator
Date Processed : 2021/10/12 19:27:35 Processed by : System Administrator

<Chromatogram>

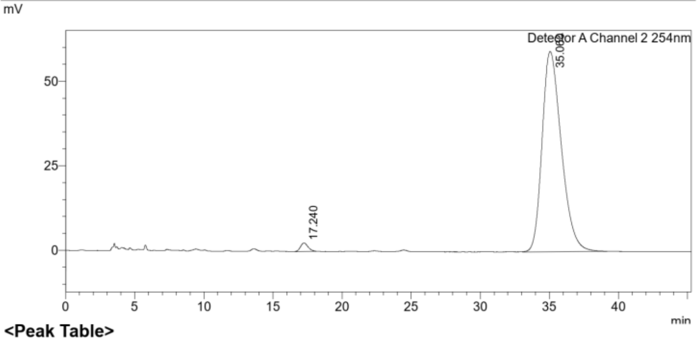

<Peak Table>

Detector A Channel 2 254nm

Peak#	Ret. Time	Area	Height	Conc.
1	17.240	95048	2502	1.627
2	35.060	5745789	59215	98.373
Total		5840837	61717	

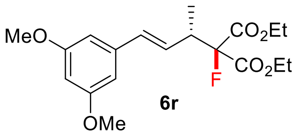

6r

Analysis Report

<Sample Information>

Sample Name	: lil-ld-69-rac-adh98-2--1.0-230-254nm-2		
Sample ID	:		
Data Filename	: lil-ld-69-rac-adh98-2--1.0-230-254nm-2.lcd		
Method Filename	: xsl-230-254-1.0.lcm		
Batch Filename	:		
Vial #	: 1-1	Sample Type	: Unknown
Injection Volume	: 20 uL		
Date Acquired	: 2021/10/21 23:11:27	Acquired by	: System Administrator
Date Processed	: 2021/10/22 0:02:43	Processed by	: System Administrator

<Chromatogram>

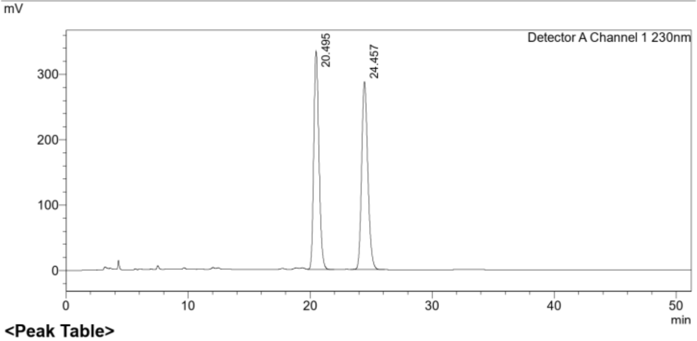

<Peak Table>

Detector A Channel 1 230nm

Peak#	Ret. Time	Area	Height	Conc.
1	20.495	9799820	333732	49.292
2	24.457	10081455	287060	50.708
Total		19881275	620793	

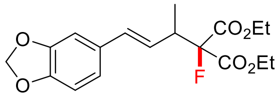

rac-**6s**

Analysis Report

<Sample Information>

Sample Name	: lil-ld-69-asy-adh98-2--1.0-230-254nm-1		
Sample ID	:		
Data Filename	: lil-ld-69-asy-adh98-2--1.0-230-254nm-1.lcd		
Method Filename	: xsl-230-254-1.0.lcm		
Batch Filename	:		
Vial #	: 1-1	Sample Type	: Unknown
Injection Volume	: 20 uL		
Date Acquired	: 2021/10/21 21:01:49	Acquired by	: System Administrator
Date Processed	: 2021/10/21 21:30:25	Processed by	: System Administrator

<Chromatogram>

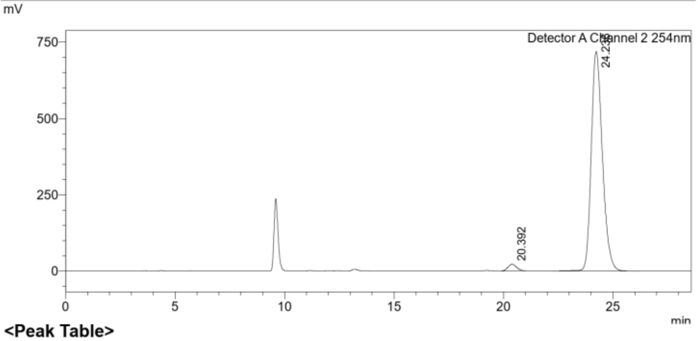

<Peak Table>

Detector A Channel 2 254nm

Peak#	Ret. Time	Area	Height	Conc.
1	20.392	597385	21683	2.287
2	24.236	25519065	719665	97.713
Total		26116450	741348	

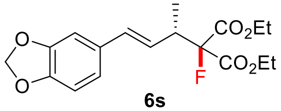

6s

Analysis Report

<Sample Information>

Sample Name	: lil-lc-78-3-rac--ADH-98-02-1.0-230nm-01
Sample ID	:
Data Filename	: lil-lc-78-3-rac--ADH-98-02-1.0-230nm-01.lcd
Method Filename	: xsl-230-254-1.0.lcm
Batch Filename	:
Vial #	: 1-1
Sample Type	: Unknown
Injection Volume	: 20 uL
Date Acquired	: 2021/9/2 17:26:51
Acquired by	: System Administrator
Date Processed	: 2021/9/2 18:05:19
Processed by	: System Administrator

<Chromatogram>

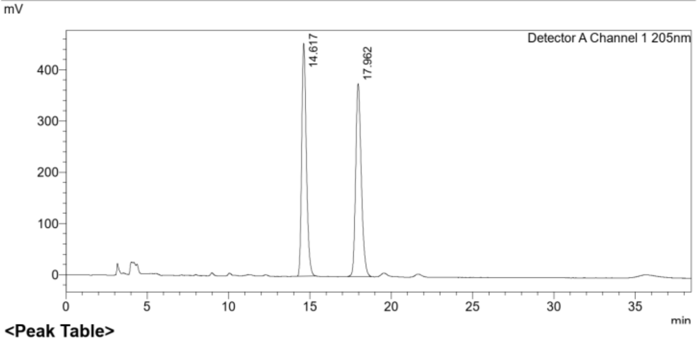

<Peak Table>

Detector A Channel 1 205nm

Peak#	Ret. Time	Area	Height	Conc.
1	14.617	9005622	453746	49.780
2	17.962	9085371	376444	50.220
Total		18090994	830190	

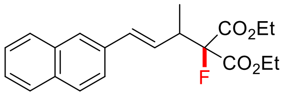

rac-**6t**

Analysis Report

<Sample Information>

Sample Name	: lil-ld-25-1-asy--adh-98-2-1.0-230-254nm-1
Sample ID	:
Data Filename	: lil-ld-25-1-asy--adh-98-2-1.0-230-254nm-1.lcd
Method Filename	: ZXX-erchun.lcm
Batch Filename	:
Vial #	: 1-1
Sample Type	: Unknown
Injection Volume	: 20 uL
Date Acquired	: 2021/10/9 20:50:16
Acquired by	: System Administrator
Date Processed	: 2021/10/9 21:29:15
Processed by	: System Administrator

<Chromatogram>

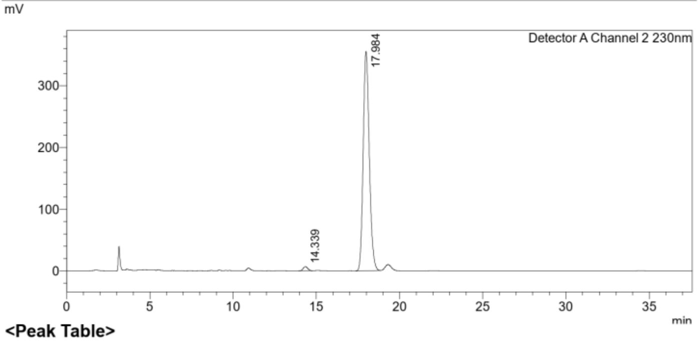

<Peak Table>

Detector A Channel 2 230nm

Peak#	Ret. Time	Area	Height	Conc.
1	14.339	137415	7063	1.490
2	17.984	9084778	355357	98.510
Total		9222193	362420	

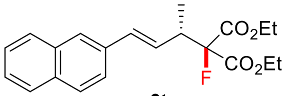

6t

Analysis Report

<Sample Information>

Sample Name	: lil-le-83-rac-adh-98-2-254-230-1.0-1		
Sample ID	:		
Data Filename	: lil-l3-83-rac-adh-98-2-254-230-1.0-1.lcd		
Method Filename	: xsl-230-254-1.0.lcm		
Batch Filename	:		
Vial #	: 1-1	Sample Type	: Unknown
Injection Volume	: 20 uL		
Date Acquired	: 2021/12/23 10:13:20	Acquired by	: System Administrator
Date Processed	: 2021/12/23 10:33:16	Processed by	: System Administrator

<Chromatogram>

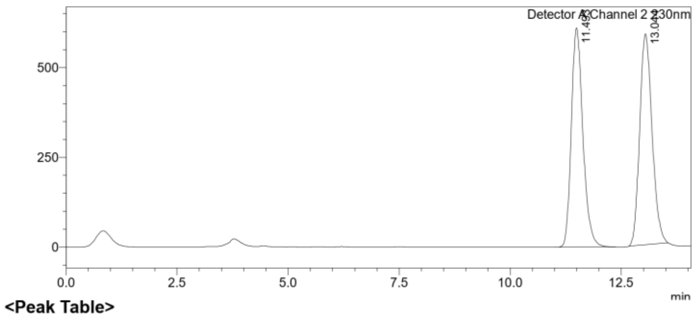

<Peak Table>

Detector A Channel 2 230nm

Peak#	Ret. Time	Area	Height	Conc.
1	11.493	11107232	610994	49.409
2	13.044	11373090	587631	50.591
Total		22480323	1198625	

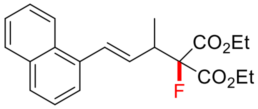

rac-**6u**

Analysis Report

<Sample Information>

Sample Name	: lil-ld-saifen--rac-adh-98-21.0-254-230-1		
Sample ID	:		
Data Filename	: lil-ld-saifen--rac-adh-98-21.0-254-230-1.lcd		
Method Filename	: xsl-230-254-1.0.lcm		
Batch Filename	:		
Vial #	: 1-1	Sample Type	: Unknown
Injection Volume	: 20 uL		
Date Acquired	: 2021/10/25 11:39:23	Acquired by	: System Administrator
Date Processed	: 2021/11/1 16:40:31	Processed by	: System Administrator

<Chromatogram>

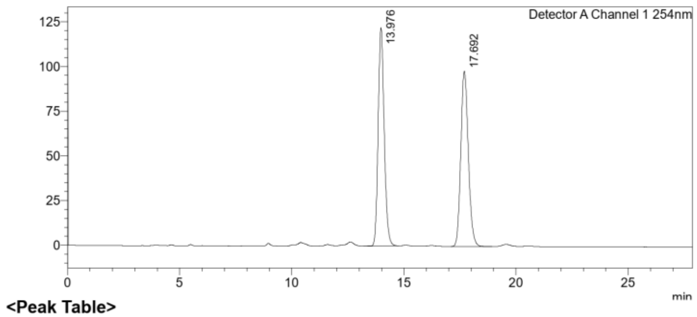

<Peak Table>

Detector A Channel 1 254nm

Peak#	Ret. Time	Area	Height	Conc.
1	13.976	2290704	122068	49.996
2	17.692	2291095	98030	50.004
Total		4581800	220098	

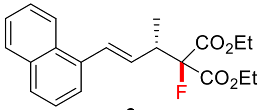

6u

Analysis Report

<Sample Information>

Sample Name : lil-ld-funan-rac-adh-98-2-254-230-1.0-2
Sample ID :
Data Filename : lil-ld-funan-rac-adh-98-2-254-230-1.0-2.lcd
Method Filename : xsl-230-254-1.0.lcm
Batch Filename :
Vial # : 1-1 Sample Type : Unknown
Injection Volume : 20 uL
Date Acquired : 2021/10/23 17:34:12 Acquired by : System Administrator
Date Processed : 2021/10/23 17:48:50 Processed by : System Administrator

<Chromatogram>

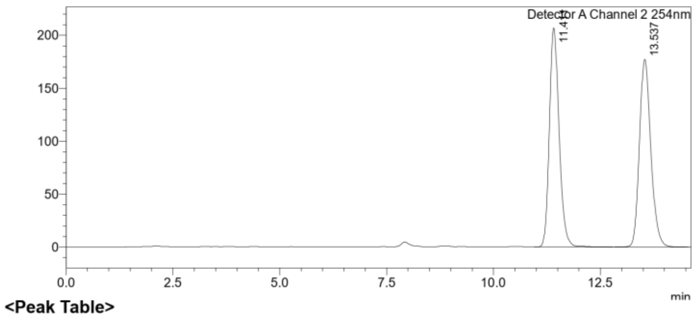

<Peak Table>

Detector A Channel 2 254nm

Peak#	Ret. Time	Area	Height	Conc.
1	11.411	3295908	206901	50.034
2	13.537	3291490	177294	49.966
Total		6587398	384195	

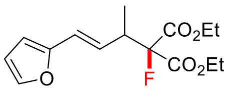

rac-**6v**

Analysis Report

<Sample Information>

Sample Name : lil-ld-63-asy-adh98-2--1.0-230-254nm-2
Sample ID :
Data Filename : lil-ld-63-asy-adh98-2--1.0-230-254nm-2.lcd
Method Filename : xsl-230-254-1.0.lcm
Batch Filename :
Vial # : 1-1 Sample Type : Unknown
Injection Volume : 20 uL
Date Acquired : 2021/10/21 20:37:01 Acquired by : System Administrator
Date Processed : 2021/10/21 21:19:43 Processed by : System Administrator

<Chromatogram>

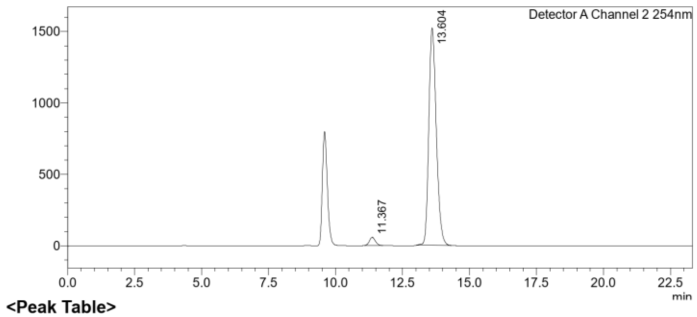

<Peak Table>

Detector A Channel 2 254nm

Peak#	Ret. Time	Area	Height	Conc.
1	11.367	895088	57395	2.929
2	13.604	29661879	1522716	97.071
Total		30556968	1580112	

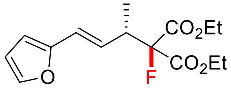

6v

2021/11/1 17:15:50 Page 1 / 1

Analysis Report

<Sample Information>

Sample Name	: lil-ld-saifen--rac-adh-98-21.0-254-230-1		
Sample ID	:		
Data Filename	: lil-ld-saifen--rac-adh-98-21.0-254-230-1.lcd		
Method Filename	: xsl-230-254-1.0.lcm		
Batch Filename	:		
Vial #	: 1-1	Sample Type	: Unknown
Injection Volume	: 20 uL		
Date Acquired	: 2021/10/25 11:39:23	Acquired by	: System Administrator
Date Processed	: 2021/11/1 16:40:31	Processed by	: System Administrator

<Chromatogram>

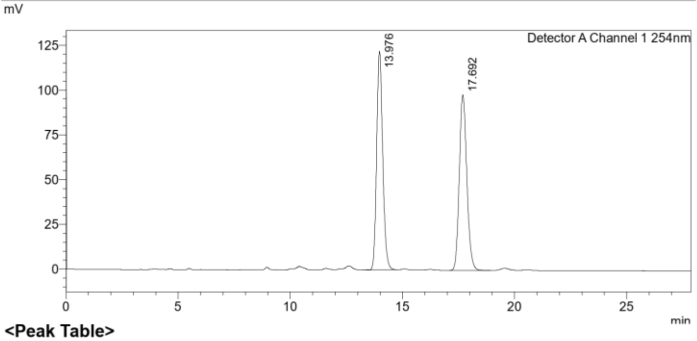

<Peak Table>

Detector A Channel 1 254nm

Peak#	Ret. Time	Area	Height	Conc.
1	13.976	2290704	122068	49.996
2	17.692	2291095	98030	50.004
Total		4581800	220098	

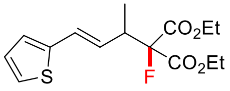

rac-**6w**

D:\Data\lil\LD\lil-ld-saifen--rac-adh-98-21.0-254-230-1.lcd

2021/11/1 16:52:18 Page 1 / 1

Analysis Report

<Sample Information>

Sample Name	: lil-ld-113-saifen-asy-adh-98-2-1.0-254-230-1		
Sample ID	:		
Data Filename	: lil-ld-113-saifen-asy-adh-98-2-1.0-254-230-1.lcd		
Method Filename	: xsl-230-254-1.0.lcm		
Batch Filename	:		
Vial #	: 1-1	Sample Type	: Unknown
Injection Volume	: 20 uL		
Date Acquired	: 2021/11/1 16:20:24	Acquired by	: System Administrator
Date Processed	: 2021/11/1 16:41:00	Processed by	: System Administrator

<Chromatogram>

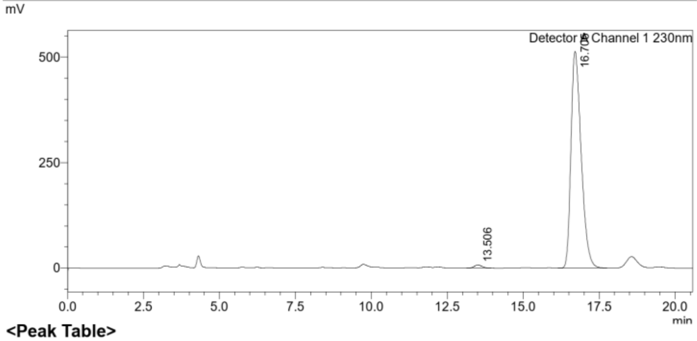

<Peak Table>

Detector A Channel 1 230nm

Peak#	Ret. Time	Area	Height	Conc.
1	13.506	130461	7309	1.078
2	16.705	11970898	513156	98.922
Total		12101360	520466	

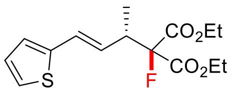

6w

D:\Data\lil\LD\lil-ld-113-saifen-asy-adh-98-2-1.0-254-230-1.lcd

Analysis Report

<Sample Information>

Sample Name : LIL-LE137-RAC-ADH-99-1-254-230-0.5-2
Sample ID :
Data Filename : LIL-LE137-RAC-ADH-99-1-254-230-0.5-2.lcd
Method Filename : xsl-230-254-1.0.lcm
Batch Filename :
Vial # : 1-1 Sample Type : Unknown
Injection Volume : 20 uL
Date Acquired : 2021/12/21 10:49:17 Acquired by : System Administrator
Date Processed : 2021/12/21 11:31:56 Processed by : System Administrator

<Chromatogram>

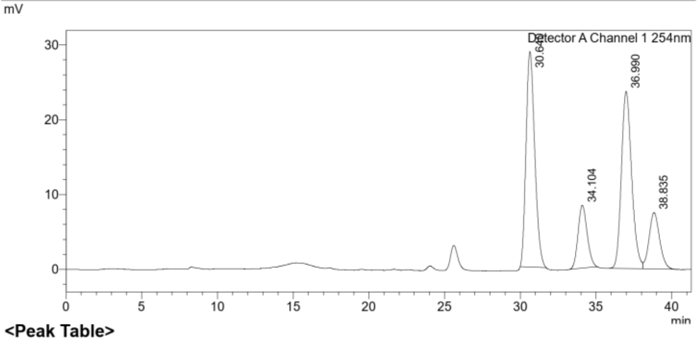

<Peak Table>

Detector A Channel 1 254nm

Peak#	Ret. Time	Area	Height	Conc.
1	30.640	1153443	28817	38.528
2	34.104	354498	8414	11.841
3	36.990	1117255	23695	37.319
4	38.835	368604	7547	12.312
Total		2993800	68472	

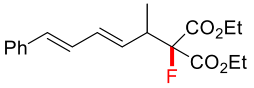

rac-**6x**

Analysis Report

<Sample Information>

Sample Name : LIL-LE137-ASY-ADH-99-1-254-230-0.5-2
Sample ID :
Data Filename : LIL-LE137-ASY-ADH-99-1-254-230-0.5-2.lcd
Method Filename : xsl-230-254-1.0.lcm
Batch Filename :
Vial # : 1-1 Sample Type : Unknown
Injection Volume : 20 uL
Date Acquired : 2021/12/21 11:33:30 Acquired by : System Administrator
Date Processed : 2021/12/21 12:15:06 Processed by : System Administrator

<Chromatogram>

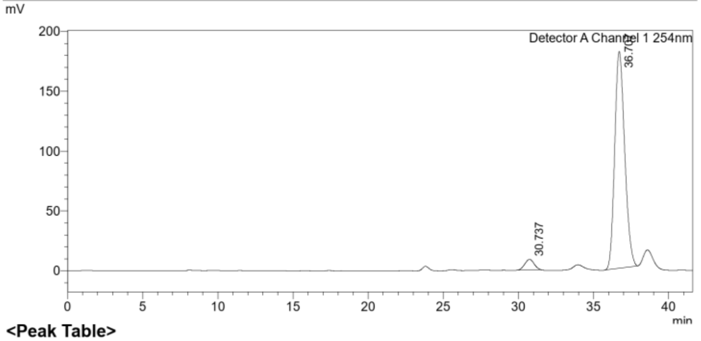

<Peak Table>

Detector A Channel 1 254nm

Peak#	Ret. Time	Area	Height	Conc.
1	30.737	385345	8876	4.398
2	36.707	8375960	181071	95.602
Total		8761305	189947	

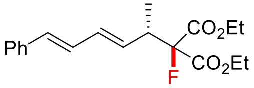

6x

Analysis Report

<Sample Information>

Sample Name : lil-ld-67-rac-adh-99-1-1.0-190-210-2
Sample ID :
Data Filename : lil-ld-67-rac-adh-99-1-1.0-190-210-1.lcd
Method Filename : xsl-230-254-1.0.lcm
Batch Filename :
Vial # : 1-1 Sample Type : Unknown
Injection Volume : 20 uL
Date Acquired : 2021/11/18 10:22:32 Acquired by : System Administrator
Date Processed : 2021/11/18 10:37:48 Processed by : System Administrator

<Chromatogram>

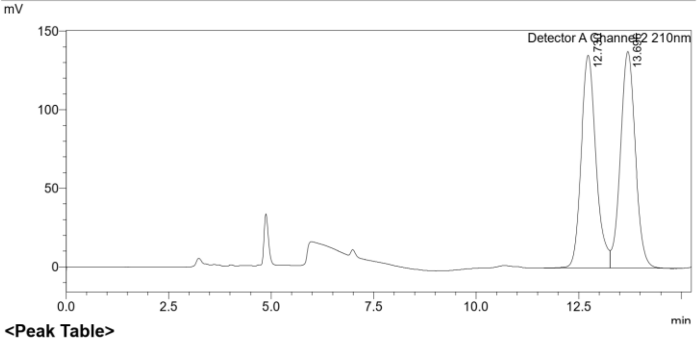

<Peak Table>

Detector A Channel 2 210nm

Peak#	Ret. Time	Area	Height	Conc.
1	12.730	3411504	135502	49.598
2	13.696	3466826	137998	50.402
Total		6878330	273500	

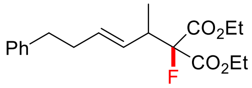

rac-**6y**

Analysis Report

<Sample Information>

Sample Name : lil-ld-67-asy-adh-99-1-1.0-190-210-2
Sample ID :
Data Filename : lil-ld-67-asy-adh-99-1-1.0-190-210-2.lcd
Method Filename : xsl-230-254-1.0.lcm
Batch Filename :
Vial # : 1-1 Sample Type : Unknown
Injection Volume : 20 uL
Date Acquired : 2021/11/18 10:00:26 Acquired by : System Administrator
Date Processed : 2021/11/18 10:21:31 Processed by : System Administrator

<Chromatogram>

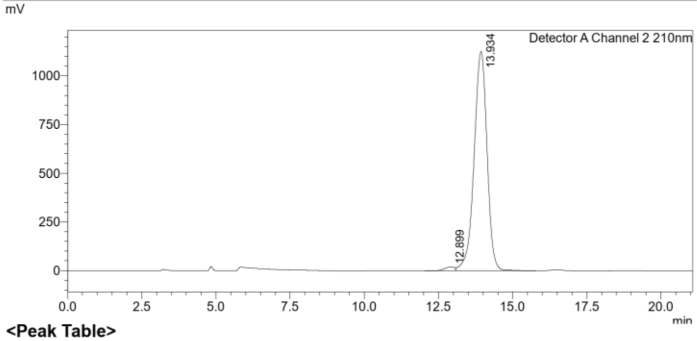

<Peak Table>

Detector A Channel 2 210nm

Peak#	Ret. Time	Area	Height	Conc.
1	12.899	491597	19861	1.410
2	13.934	34378095	1126912	98.590
Total		34869692	1146773	

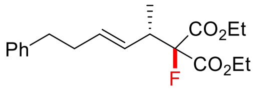

6y

Analysis Report

<Sample Information>

Sample Name	: lil-ld-150-rac-IF-99-1-0.5-210-230-1		
Sample ID	:		
Data Filename	: lil-ld-150-rac-IF-99-1-0.5-210-230-1.lcd		
Method Filename	: xsl-230-254-1.0.lcm		
Batch Filename	:		
Vial #	: 1-1	Sample Type	: Unknown
Injection Volume	: 20 uL		
Date Acquired	: 2021/12/6 14:52:50	Acquired by	: System Administrator
Date Processed	: 2021/12/6 15:19:36	Processed by	: System Administrator

<Chromatogram>

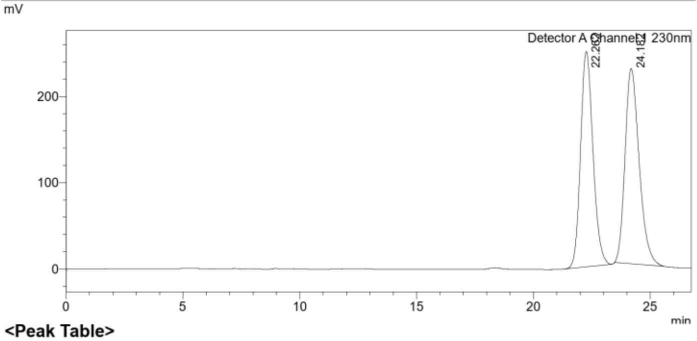

<Peak Table>

Detector A Channel 1 230nm

Peak#	Ret. Time	Area	Height	Conc.
1	22.262	9199120	249656	49.212
2	24.182	9493693	226296	50.788
Total		18692813	475952	

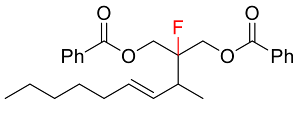

rac-**6z'**

Analysis Report

<Sample Information>

Sample Name	: lil-ld-150-ASY-IF-99-1-0.5-210-230-1		
Sample ID	:		
Data Filename	: lil-ld-150-ASY-IF-99-1-0.5-210-230-1.lcd		
Method Filename	: xsl-230-254-1.0.lcm		
Batch Filename	:		
Vial #	: 1-1	Sample Type	: Unknown
Injection Volume	: 20 uL		
Date Acquired	: 2021/12/6 15:23:13	Acquired by	: System Administrator
Date Processed	: 2021/12/6 15:51:12	Processed by	: System Administrator

<Chromatogram>

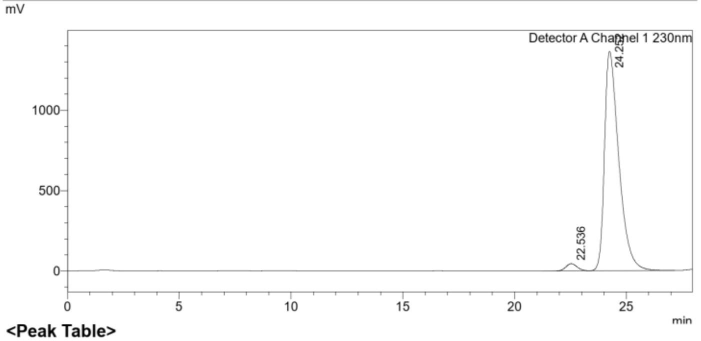

<Peak Table>

Detector A Channel 1 230nm

Peak#	Ret. Time	Area	Height	Conc.
1	22.536	1636446	45706	2.618
2	24.252	60860726	1364211	97.382
Total		62497172	1409918	

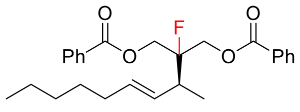

6z'

Analysis Report

<Sample Information>

Sample Name	: lil-ld-111-rac-ozh-99-1-254-230-4		
Sample ID	:		
Data Filename	: lil-ld-111-rac-ozh-99-1-254-230-4.lcd		
Method Filename	: xsl-230-254-1.0.lcm		
Batch Filename	:		
Vial #	: 1-1	Sample Type	: Unknown
Injection Volume	: 20 uL		
Date Acquired	: 2021/12/4 20:03:33	Acquired by	: System Administrator
Date Processed	: 2021/12/4 20:20:57	Processed by	: System Administrator

<Chromatogram>

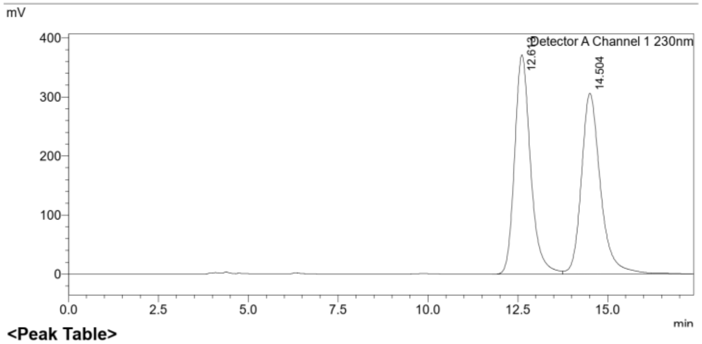

<Peak Table>

Detector A Channel 1 230nm

Peak#	Ret. Time	Area	Height	Conc.
1	12.613	11468574	371266	50.274
2	14.504	11343744	305817	49.726
Total		22812318	677084	

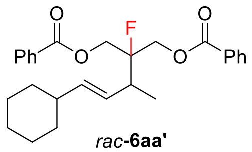

rac-**6aa'**

Analysis Report

<Sample Information>

Sample Name	: lil-ld-111-asy-ozh-99-1-254-230-2		
Sample ID	:		
Data Filename	: lil-ld-111-asy-ozh-99-1-254-230-2.lcd		
Method Filename	: xsl-230-254-1.0.lcm		
Batch Filename	:		
Vial #	: 1-1	Sample Type	: Unknown
Injection Volume	: 20 uL		
Date Acquired	: 2021/12/4 19:46:47	Acquired by	: System Administrator
Date Processed	: 2021/12/4 20:02:37	Processed by	: System Administrator

<Chromatogram>

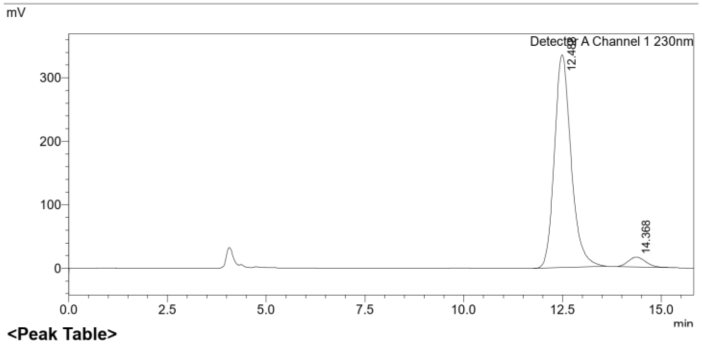

<Peak Table>

Detector A Channel 1 230nm

Peak#	Ret. Time	Area	Height	Conc.
1	12.488	9836233	334909	95.298
2	14.368	485309	15682	4.702
Total		10321543	350591	

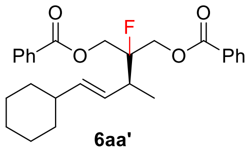

6aa'

Operator:dell Timebase:U-3000 Sequence:XSL

Page 1-1
2008-10-25 6:59 上午

103 lil-II-wanji-RAC-IF-230-1.0-3

Sample Name:	**lil-II-wanji-RAC-IF-230-1.0-3**	*Injection Volume:*	**20.0**
Vial Number:	**465**	*Channel:*	**UV_VIS_1**
Sample Type:	**standard**	*Wavelength:*	**230**
Control Program:	**XSL**	*Bandwidth:*	**n.a.**
Quantif. Method:	**XSL**	*Dilution Factor:*	**1.0000**
Run Time (min):	**9.91**	*Sample Amount:*	**1.0000**

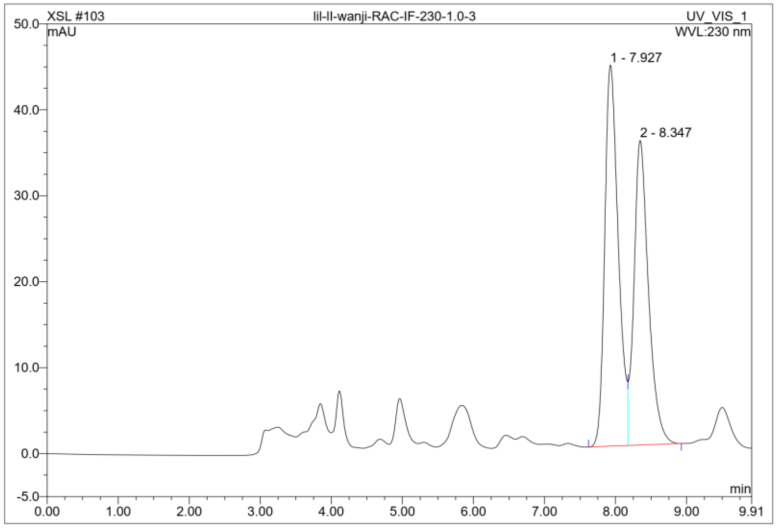

No.	Ret.Time min	Peak Name	Height mAU	Area mAU*min	Rel.Area %	Amount	Type
1	7.93	n.a.	44.350	9.759	53.74	n.a.	BM
2	8.35	n.a.	35.453	8.400	46.26	n.a.	MB
Total:			79.803	18.159	100.00	0.000	

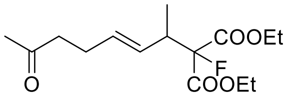

rac-**6ab**

Mu/Integration

Chromeleon (c) Dionex 1996-2006
Version 6.80 SR8a Build 2643 (158225)

Operator:dell Timebase:U-3000 Sequence:XSL

Page 1-1
2008-10-24 6:40 上午

101 lil-II-wanji-ASY-IF-230-1.0-2

Sample Name:	**lil-II-wanji-ASY-IF-230-1.0-2**	*Injection Volume:*	**20.0**
Vial Number:	**463**	*Channel:*	**UV_VIS_1**
Sample Type:	**standard**	*Wavelength:*	**230**
Control Program:	**XSL**	*Bandwidth:*	**n.a.**
Quantif. Method:	**XSL**	*Dilution Factor:*	**1.0000**
Run Time (min):	**10.57**	*Sample Amount:*	**1.0000**

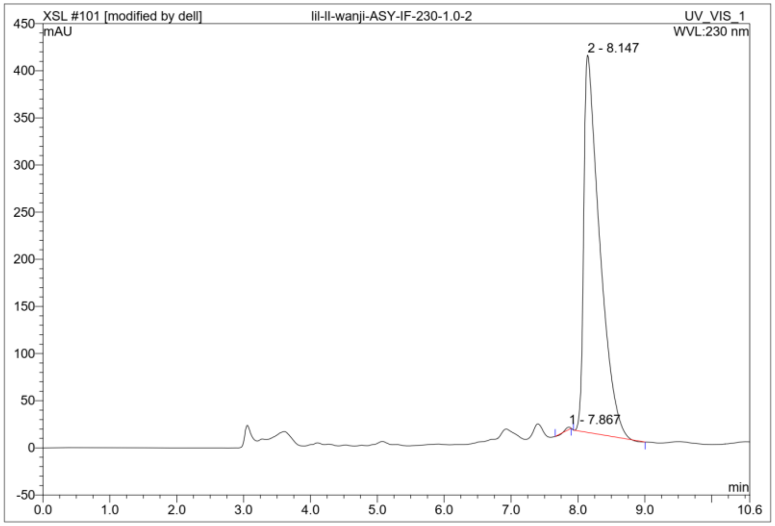

No.	Ret.Time min	Peak Name	Height mAU	Area mAU*min	Rel.Area %	Amount	Type
1	7.87	n.a.	2.409	0.155	0.14	n.a.	BMB*
2	8.15	n.a.	400.304	109.948	99.86	n.a.	BMB*
Total:			402.714	110.103	100.00	0.000	

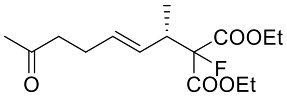

6ab

Mu/Integration

Chromeleon (c) Dionex 1996-2006
Version 6.80 SR8a Build 2643 (158225)

Analysis Report

<Sample Information>

Sample Name : LIL-LEMCPBA--ADH-98-2-254-230-1.0-2
Sample ID :
Data Filename : LIL-LEMCPBA--ADH-98-2-254-230-1.0-2.lcd
Method Filename : xsl-230-254-1.0.lcm
Batch Filename :
Vial # : 1-1 Sample Type : Unknown
Injection Volume : 20 uL
Date Acquired : 2021/12/21 13:25:40 Acquired by : System Administrator
Date Processed : 2021/12/21 21:07:30 Processed by : System Administrator

<Chromatogram>

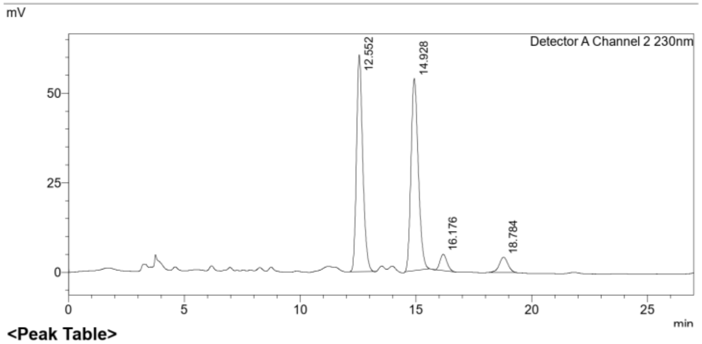

<Peak Table>

Detector A Channel 2 230nm

Peak#	Ret. Time	Area	Height	Conc.
1	12.552	1137819	60493	45.055
2	14.928	1179993	53660	46.726
3	16.176	95602	4565	3.786
4	18.784	111959	4261	4.433
Total		2525372	122979	

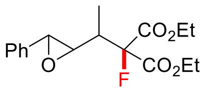

rac-**9**

Analysis Report

<Sample Information>

Sample Name : LIL-LEMCPBA-ASY--ADH-98-2-254-230-1.0-2
Sample ID :
Data Filename : LIL-LEMCPBA-ASY-ADH-98-2-254-230-1.0-2.lcd
Method Filename : xsl-230-254-1.0.lcm
Batch Filename :
Vial # : 1-1 Sample Type : Unknown
Injection Volume : 20 uL
Date Acquired : 2021/12/21 13:53:18 Acquired by : System Administrator
Date Processed : 2021/12/21 14:14:47 Processed by : System Administrator

<Chromatogram>

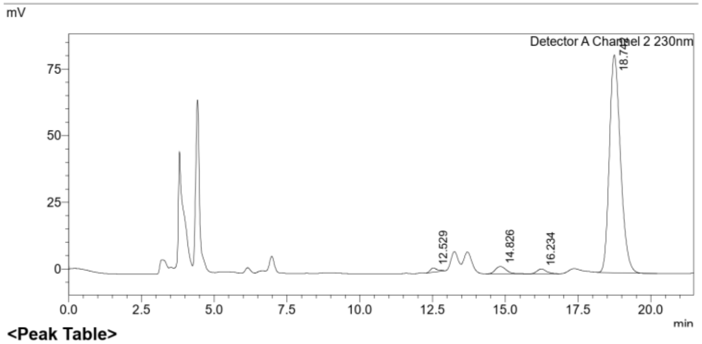

<Peak Table>

Detector A Channel 2 230nm

Peak#	Ret. Time	Area	Height	Conc.
1	12.529	26819	1585	1.160
2	14.826	72359	2727	3.129
3	16.234	41047	1801	1.775
4	18.742	2172366	81691	93.936
Total		2312591	87805	

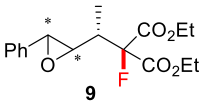

9

Analysis Report

<Sample Information>

Sample Name : LIL-LE-103-RAC-ODH-98-2-254-230-1
Sample ID :
Data Filename : LIL-LE-103-RAC-ODH-98-2-254-230-1.lcd
Method Filename : xsl-230-254-1.0.lcm
Batch Filename :
Vial # : 1-1 Sample Type : Unknown
Injection Volume : 20 uL
Date Acquired : 2021/12/20 9:49:03 Acquired by : System Administrator
Date Processed : 2021/12/20 10:57:27 Processed by : System Administrator

<Chromatogram>

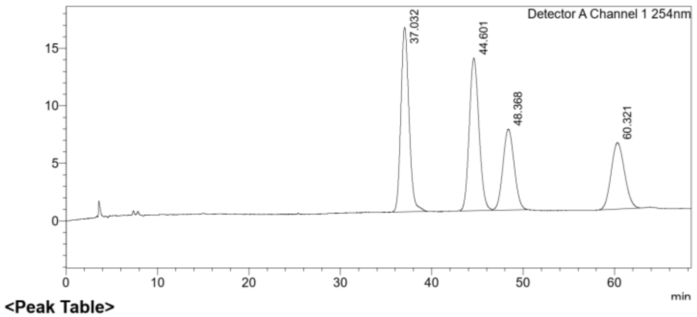

<Peak Table>

Detector A Channel 1 254nm

Peak#	Ret. Time	Area	Height	Conc.
1	37.032	995084	16026	31.365
2	44.601	983052	13280	30.986
3	48.368	606000	7047	19.101
4	60.321	588430	5785	18.547
Total		3172566	42138	

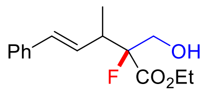

rac-**10**

Analysis Report

<Sample Information>

Sample Name : LIL-LE-103-ASY-ODH-98-2-254-230-1
Sample ID :
Data Filename : LIL-LE-103-ASY-ODH-98-2-254-230-1.lcd
Method Filename : xsl-230-254-1.0.lcm
Batch Filename :
Vial # : 1-1 Sample Type : Unknown
Injection Volume : 20 uL
Date Acquired : 2021/12/20 11:06:08 Acquired by : System Administrator
Date Processed : 2021/12/20 12:05:31 Processed by : System Administrator

<Chromatogram>

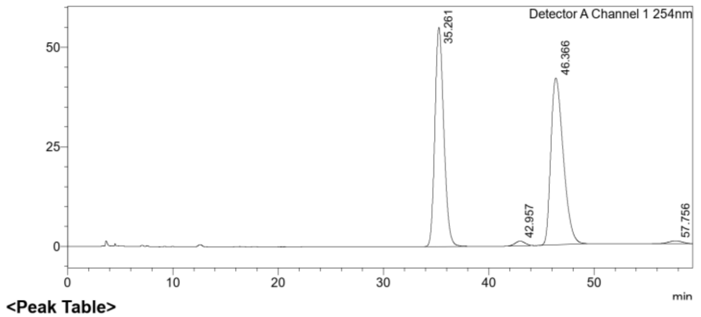

<Peak Table>

Detector A Channel 1 254nm

Peak#	Ret. Time	Area	Height	Conc.
1	35.261	3238785	55014	47.460
2	42.957	81052	1200	1.188
3	46.366	3436364	41868	50.356
4	57.756	67970	731	0.996
Total		6824171	98813	

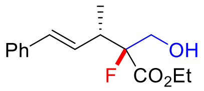

10

Analysis Report

<Sample Information>

Sample Name	: LIL-LF-17-RAC-OJH-75-25-1.0-254-230-1		
Sample ID	:		
Data Filename	: LIL-LF-17-RAC-OJH-75-25-1.0-254-230-1.lcd		
Method Filename	: gay-1.0-254-1.lcm		
Batch Filename	:		
Vial #	: 1-1	Sample Type	: Unknown
Injection Volume	: 10 uL		
Date Acquired	: 2022/1/10 11:43:07	Acquired by	: System Administrator
Date Processed	: 2022/1/10 12:11:20	Processed by	: System Administrator

<Chromatogram>

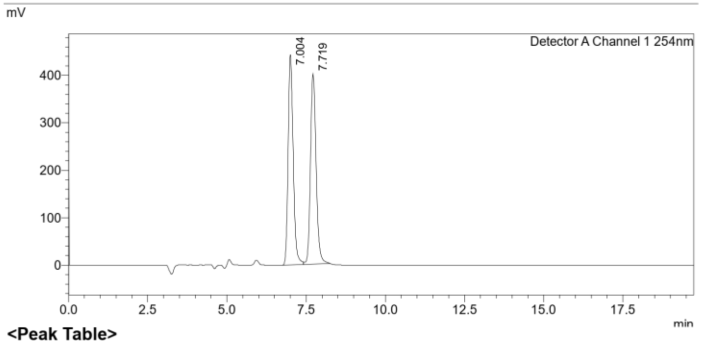

<Peak Table>

Detector A Channel 1 254nm

Peak#	Ret. Time	Area	Height	Conc.
1	7.004	4945536	442571	49.895
2	7.719	4966330	400920	50.105
Total		9911866	843491	

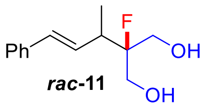

***rac*-11**

Analysis Report

<Sample Information>

Sample Name	: LIL-LF-17-ASY-OJH-75-25-1.0-254-230-1		
Sample ID	:		
Data Filename	: LIL-LF-17-ASY-OJH-75-25-1.0-254-230-1.lcd		
Method Filename	: gay-1.0-254-1.lcm		
Batch Filename	:		
Vial #	: 1-1	Sample Type	: Unknown
Injection Volume	: 10 uL		
Date Acquired	: 2022/1/10 12:14:49	Acquired by	: System Administrator
Date Processed	: 2022/1/10 12:25:34	Processed by	: System Administrator

<Chromatogram>

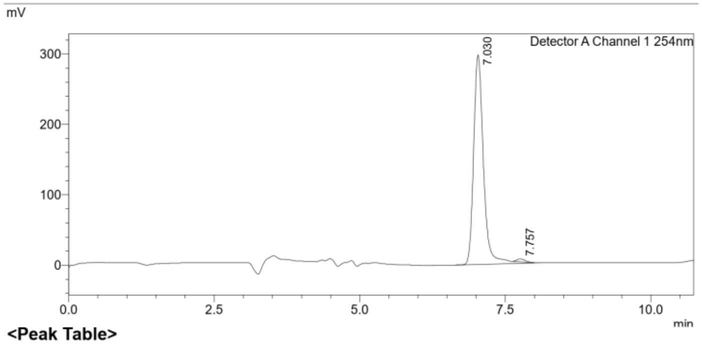

<Peak Table>

Detector A Channel 1 254nm

Peak#	Ret. Time	Area	Height	Conc.
1	7.030	3498146	297666	98.847
2	7.757	40791	4100	1.153
Total		3538938	301766	

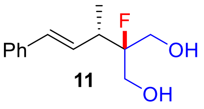

11

2022/1/1 19:49:26 Page 1 / 1

Analysis Report

<Sample Information>

Sample Name	: LIL-LF-12-RAC-OXH-95-5-254-230-1
Sample ID	:
Data Filename	: LIL-LF-12-RAC-OXH-95-5-254-230-1.lcd
Method Filename	: 1.0.lcm
Batch Filename	:
Vial #	: 1-1
Injection Volume	: 10 uL
Date Acquired	: 2022/1/1 19:12:05
Date Processed	: 2022/1/1 19:48:21
Sample Type	: Unknown
Acquired by	: System Administrator
Processed by	: System Administrator

<Chromatogram>

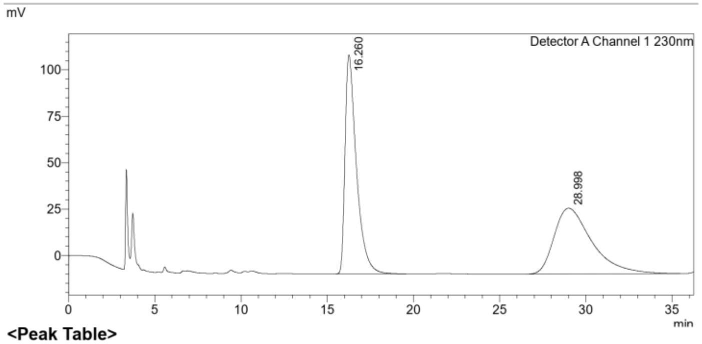

<Peak Table>

Detector A Channel 1 230nm

Peak#	Ret. Time	Area	Height	Conc.
1	16.260	5415448	118013	49.941
2	28.998	5428244	35485	50.059
Total		10843692	153498	

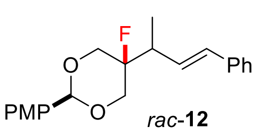

rac-**12**

D:\Data\lil\LE\LIL-LF-12-RAC-OXH-95-5-254-230-1.lcd

2022/1/9 21:43:56 Page 1 / 1

Analysis Report

<Sample Information>

Sample Name	: LIL-LF-28-ASY-OXH-90-10-1.0-254-230-1
Sample ID	:
Data Filename	: LIL-LF-28-ASY-OXH-90-10-1.0-254-230-1.lcd
Method Filename	: gay-1.0-254-1.lcm
Batch Filename	:
Vial #	: 1-1
Injection Volume	: 10 uL
Date Acquired	: 2022/1/6 12:10:24
Date Processed	: 2022/1/6 15:38:16
Sample Type	: Unknown
Acquired by	: System Administrator
Processed by	: System Administrator

<Chromatogram>

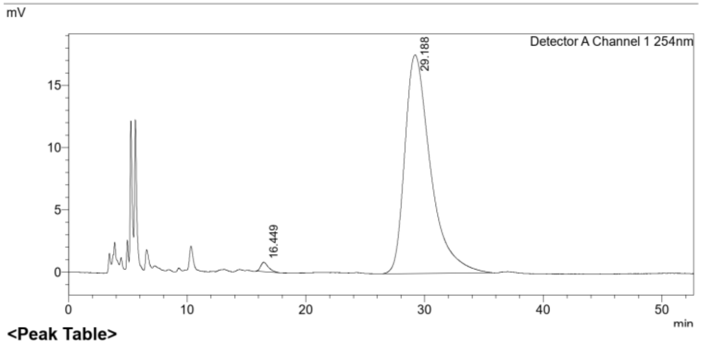

<Peak Table>

Detector A Channel 1 254nm

Peak#	Ret. Time	Area	Height	Conc.
1	16.449	35086	764	1.307
2	29.188	2649181	17565	98.693
Total		2684267	18328	

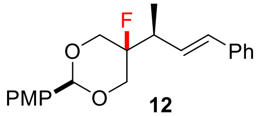

12

D:\Data\lil\LF\LIL-LF-28-ASY-OXH-90-10-1.0-254-230-1.lcd

Analysis Report

<Sample Information>

Sample Name	: yjs-ya-18-rac-adh-70-30-1.0-254-230-1		
Sample ID	:		
Data Filename	: yjs-ya-18-rac-adh-70-30-1.0-254-230-3.lcd		
Method Filename	: gay-1.0-254-1.lcm		
Batch Filename	:		
Vial #	: 1-1	Sample Type	: Unknown
Injection Volume	: 20 uL		
Date Acquired	: 2021/11/17 21:31:00	Acquired by	: System Administrator
Date Processed	: 2021/11/17 23:23:06	Processed by	: System Administrator

<Chromatogram>

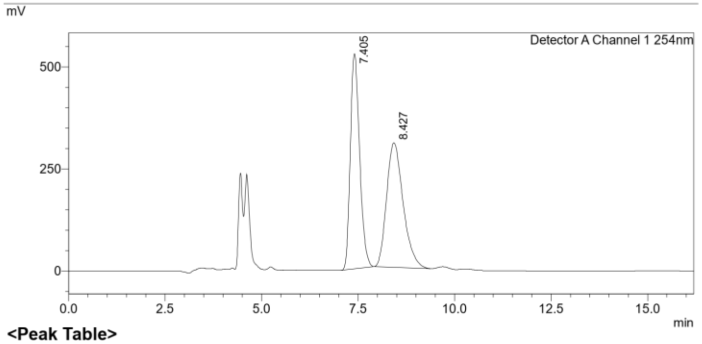

<Peak Table>

Detector A Channel 1 254nm

Peak#	Ret. Time	Area	Height	Conc.
1	7.405	8851023	526678	49.396
2	8.427	9067593	304711	50.604
Total		17918617	831389	

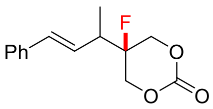

rac-**13**

Analysis Report

<Sample Information>

Sample Name	: yjs-ya-33-asy-adh-70-30-1.0-254-230-1		
Sample ID	:		
Data Filename	: yjs-ya-33-asy-adh-70-30-1.0-254-230-1.lcd		
Method Filename	: gay-1.0-254-1.lcm		
Batch Filename	:		
Vial #	: 1-1	Sample Type	: Unknown
Injection Volume	: 20 uL		
Date Acquired	: 2021/11/17 21:10:23	Acquired by	: System Administrator
Date Processed	: 2021/11/17 21:48:01	Processed by	: System Administrator

<Chromatogram>

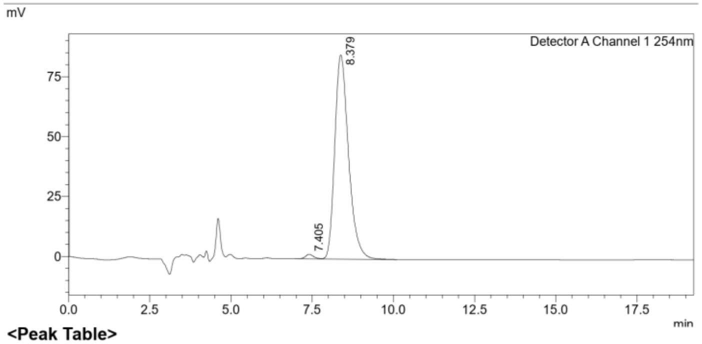

<Peak Table>

Detector A Channel 1 254nm

Peak#	Ret. Time	Area	Height	Conc.
1	7.405	33615	1879	1.318
2	8.379	2517647	85269	98.682
Total		2551262	87148	

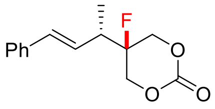

13

Analysis Report

<Sample Information>

Sample Name : LIL-LF-22-RAC-ODH-90-10-1.0-254-230-1
Sample ID :
Data Filename : LIL-LF-22-RAC-ODH-90-10-1.0-254-230-1.lcd
Method Filename : gay-1.0-254-1.lcm
Batch Filename :
Vial # : 1-1 Sample Type : Unknown
Injection Volume : 10 uL
Date Acquired : 2022/1/6 11:14:12 Acquired by : System Administrator
Date Processed : 2022/1/6 11:25:45 Processed by : System Administrator

<Chromatogram>

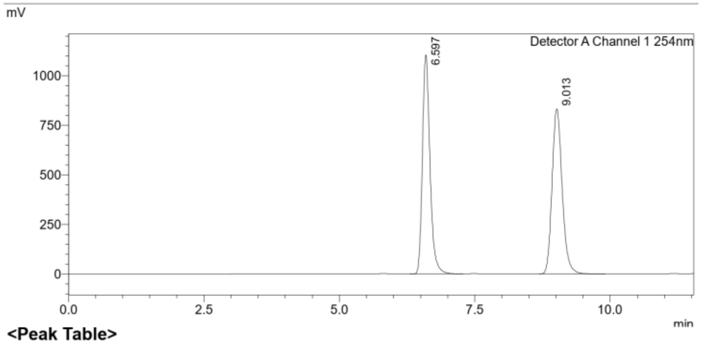

<Peak Table>

Detector A Channel 1 254nm

Peak#	Ret. Time	Area	Height	Conc.
1	6.597	10725439	1104460	49.825
2	9.013	10800746	831966	50.175
Total		21526185	1936425	

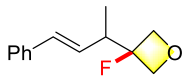

14

Analysis Report

<Sample Information>

Sample Name : LIL-LF-26-ASY-ODH-90-10-1.0-254-230-1
Sample ID :
Data Filename : LIL-LF-26-ASY-ODH-90-10-1.0-254-230-1.lcd
Method Filename : gay-1.0-254-1.lcm
Batch Filename :
Vial # : 1-1 Sample Type : Unknown
Injection Volume : 10 uL
Date Acquired : 2022/1/6 11:27:52 Acquired by : System Administrator
Date Processed : 2022/1/6 11:42:17 Processed by : System Administrator

<Chromatogram>

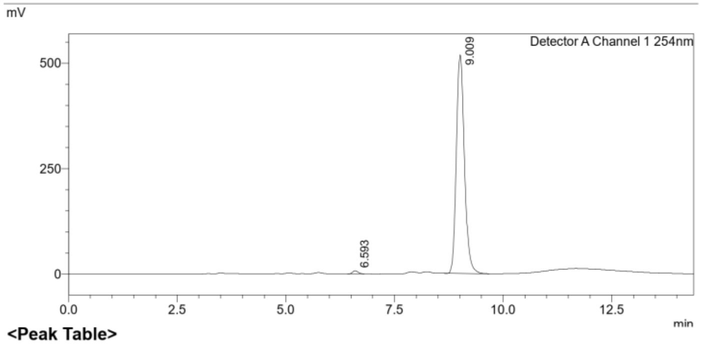

<Peak Table>

Detector A Channel 1 254nm

Peak#	Ret. Time	Area	Height	Conc.
1	6.593	68034	7537	1.006
2	9.009	6697946	518127	98.994
Total		6765980	525664	

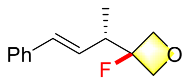

14

Analysis Report

<Sample Information>

Sample Name : LIL-LE-43-RAC-ADH-90-10--0.5-254-230-1
Sample ID :
Data Filename : LIL-LE-43-RAC-ADH-90-10--0.5-254-230-1.lcd
Method Filename : ZXX-ZH-84.lcm
Batch Filename :
Vial # : 1-1 Sample Type : Unknown
Injection Volume : 20 uL
Date Acquired : 2021/12/16 9:57:03 Acquired by : System Administrator
Date Processed : 2021/12/16 11:17:14 Processed by : System Administrator

<Chromatogram>

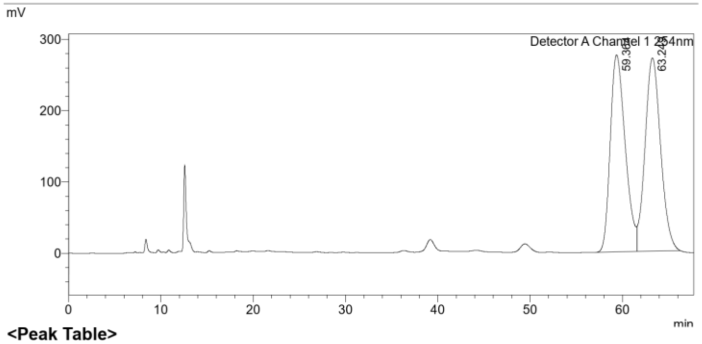

<Peak Table>

Detector A Channel 1 254nm

Peak#	Ret. Time	Area	Height	Conc.
1	59.364	31195589	276411	49.528
2	63.249	31790064	271197	50.472
Total		62985654	547607	

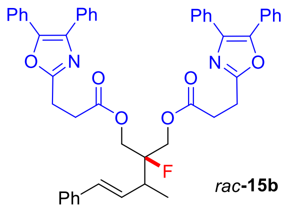

rac-**15b**

Analysis Report

<Sample Information>

Sample Name : LIL-LE-43-ASY-ADH-90-10--0.5-254-230-1
Sample ID :
Data Filename : LIL-LE-43-ASY-ADH-90-10--0.5-254-230-1.lcd
Method Filename : ZXX-ZH-84.lcm
Batch Filename :
Vial # : 1-1 Sample Type : Unknown
Injection Volume : 20 uL
Date Acquired : 2021/12/16 11:15:46 Acquired by : System Administrator
Date Processed : 2021/12/16 12:38:58 Processed by : System Administrator

<Chromatogram>

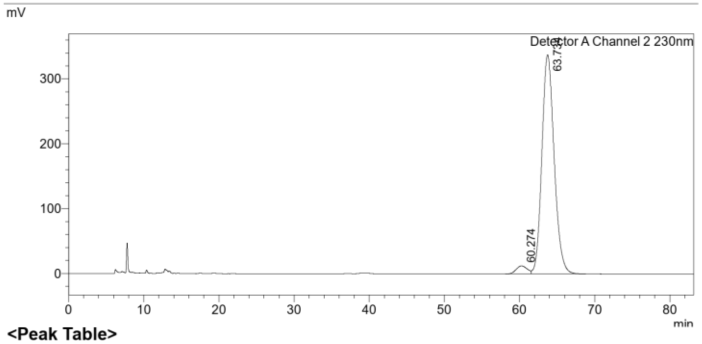

<Peak Table>

Detector A Channel 1 254nm

Peak#	Ret. Time	Area	Height	Conc.
1	60.277	417446	5365	1.686
2	63.733	24344353	218778	98.314
Total		24761800	224143	

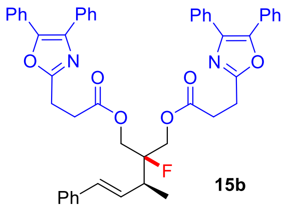

15b

Analysis Report

<Sample Information>

Sample Name : lil-le-45-rac-adh-90-10-1.0-210-230-1
Sample ID :
Data Filename : lil-le-45-rac-adh-90-10-1.0-210-230-1.lcd
Method Filename : xsl-230-254-1.0.lcm
Batch Filename :
Vial # : 1-1 Sample Type : Unknown
Injection Volume : 20 uL
Date Acquired : 2021/12/7 21:45:18 Acquired by : System Administrator
Date Processed : 2021/12/13 9:17:43 Processed by : System Administrator

<Chromatogram>

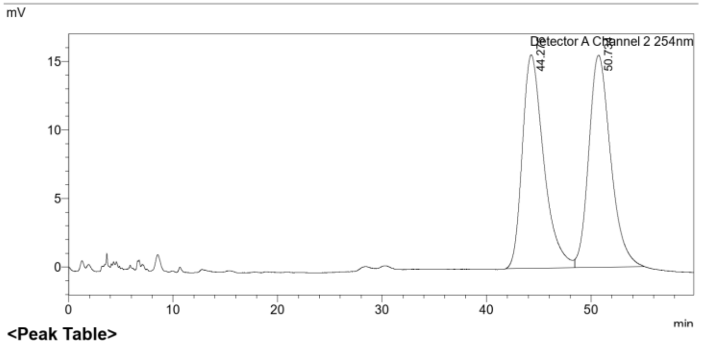

<Peak Table>

Detector A Channel 2 254nm

Peak#	Ret. Time	Area	Height	Conc.
1	44.276	2219413	15558	49.740
2	50.734	2242609	15472	50.260
Total		4462022	31030	

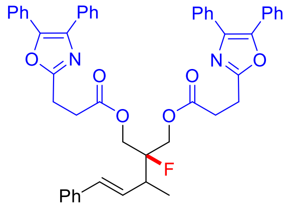

rac-**15c**

Analysis Report

<Sample Information>

Sample Name : lil-le-45-asy-adh-90-10-1.0-254-230-2
Sample ID :
Data Filename : lil-le-45-asy-adh-90-10-1.0-254-230-2.lcd
Method Filename : xsl-230-254-1.0.lcm
Batch Filename :
Vial # : 1-1 Sample Type : Unknown
Injection Volume : 20 uL
Date Acquired : 2021/12/14 18:19:22 Acquired by : System Administrator
Date Processed : 2021/12/14 19:21:01 Processed by : System Administrator

<Chromatogram>

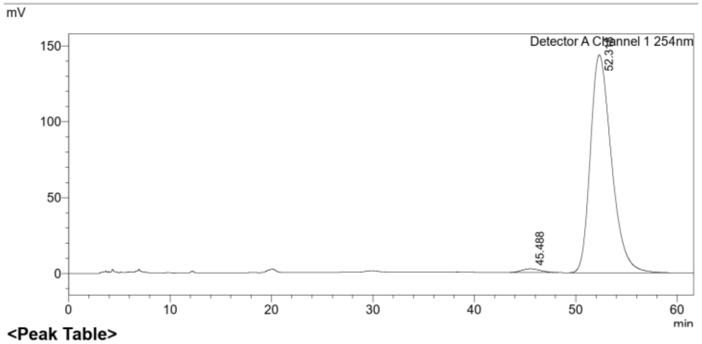

<Peak Table>

Detector A Channel 1 254nm

Peak#	Ret. Time	Area	Height	Conc.
1	45.488	312855	2433	1.488
2	52.318	20716469	143497	98.512
Total		21029324	145930	

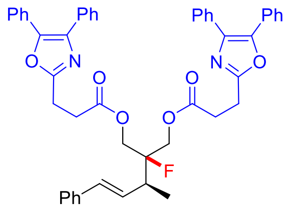

15c
